# Enantioselective Olefin 1,2-Arylamination Catalyzed by a Planar Chiral Indenyl-Rhodium(III) Complex

**DOI:** 10.1021/acscatal.5c07589

**Published:** 2026-01-16

**Authors:** Patrick Gross, Hyoju Choi, Wesley A. Pullara, Hoyoung Im, Khang Ung, Seunguk Kang, Mu-Hyun Baik, Simon B. Blakey

**Affiliations:** a Department of Chemistry, 1371 Emory University, Atlanta, Georgia 30322, United States; b Department of Chemistry, Korea Advanced Institute of Science and Technology (KAIST), Daejeon 34141, Korea; c Center for Catalytic Hydrocarbon Functionalizations, Institute for Basic Science (IBS), Daejeon 34141, Korea

**Keywords:** rhodium, cyclization, stereoselective, nitrene, mechanism

## Abstract

We report an enantioselective 1,2-arylamination of unactivated alkenes catalyzed by a chiral indenyl-Rh­(III) complex using Troc-protected hydroxylamine derivatives. This method enables efficient access to structurally diverse 2-aminotetralin scaffolds and amino-substituted carbospirocycles via a 6-endo cyclization involving electrophilic aromatic substitution (EAS). Experimental and computational studies reveal that the electronic asymmetry of the chiral indenyl ligand plays a pivotal role in enhancing catalytic activity compared to cyclopentadienyl-based analogs. Furthermore, the identity of the N-protecting group on hydroxylamine significantly influences the reaction pathway, distinguishing this transformation from previously reported aziridination strategies. The synthetic utility and versatility of this catalytic system are further demonstrated through streamlined syntheses of biologically relevant molecules, highlighting its strong potential for applications in medicinal chemistry.

## Graphical abstract

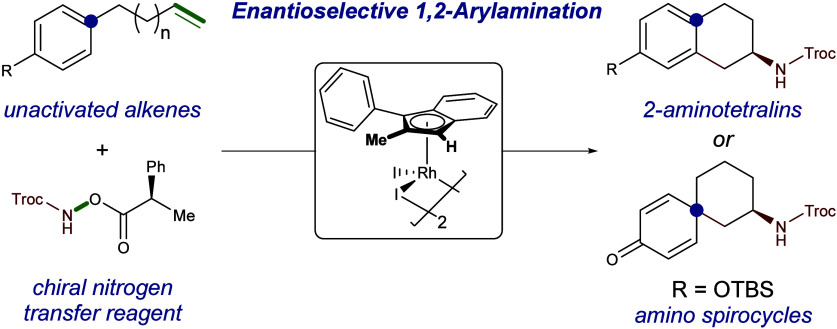

## Introduction

The 2-aminotetralin scaffold is a rigidified phenethylamine analogue that shares many of the biological activities associated with other phenethylamines. Compounds containing 2-aminotetralins, such as the 5-HT_1A_/5-HT_7_ agonist 8-OH-DPAT (**1**), are frequently employed in neurological receptor studies (Figure 1).^1^ In recent years, modern medicinal chemistry has leveraged the neurological activity of the 2-aminotetralin framework to develop treatments for disorders such as depression and addiction.^2^ This includes the successful development of pharmaceuticals like rotogotine (**2**), used to alleviate symptoms of Parkinson’s disease.^3^

**Figure 1:**
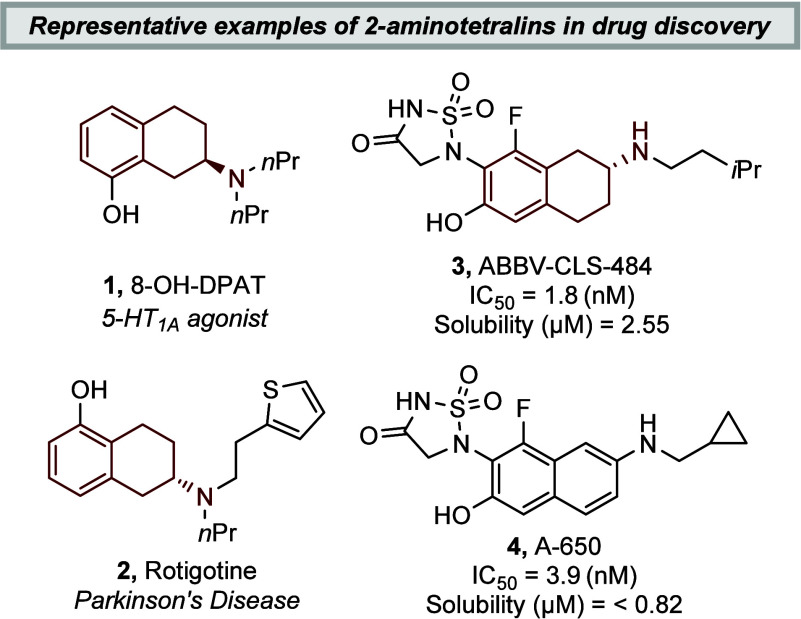
2-Aminotetralins in drug discovery.

More recently, interest in the 2-aminotetralin scaffold has grown due to its enhanced sp^3^ character, aligning with current medicinal chemistry efforts to “escape from flatland” and improve both biological and physicochemical properties of drug candidates.^4^ Consequently, 2-aminotetralin-containing compounds have found applications beyond neurological disorders.^5^ One prominent example is ABBV-CLS-484 (**3**), where partial saturation of the amino naphthalene core in lead compound A-650 (**4**) to generate the 2-aminotetralin-containing derivative improved aqueous solubility and enabled the development of a first-in-class dual-action cancer immunotherapy.^5^ Stereoselective synthesis of 2-aminotetralins is typically achieved via reductive amination of β-tetralones;^6^ however, β-tetralones are often difficult to access and have limited commercial availability. Thus, a direct enantioselective synthesis of the 2-aminotetralin core would constitute a valuable advance in synthetic and medicinal chemistry. Transition metal catalysis has emerged as a powerful strategy to access these challenging sp^3^-rich motifs with high levels of stereocontrol.

Our group has a long-standing interest in developing methods to access sp^3^-rich, nitrogen-containing scaffolds.^7^ To enable asymmetric catalysis, we previously developed a planar chiral indenyl-Rh­(III) catalyst (**5**), which can be conveniently synthesized from commercially available materials (Figure 2).^8^ This complex exhibits reactivity distinct from that of cyclopentadienyl-based systems due to the well-known “indenyl effect”, which involves facile η^5^-to-η^3^ ring slippage.^9,10^ This effect originates from the intrinsic electronic asymmetry of the indenyl ligand, resulting from its extended π-system.^11^ As illustrated in Figure 2a, Rh­(III)-complexes bearing a cyclopentadienyl ligand typically adopt a symmetric η^5^ binding mode through effective orbital overlap between the ligand’s 2π orbital and the metal’s d_*yz*_ orbital. In contrast, indenyl ligands feature inherently asymmetric π-MOs–particularly the 2π and 4π orbitals–that prevent full η^5^ coordination. Notably, the 2π MO of the indenyl ligand exhibits reduced orbital coefficients at the fused-ring junction (η^2^-carbon in the five-membered ring), which weakens the Rh–C bonding interaction at the η^2^ face. Simultaneously, the d_*yz*_ orbital of the Rh­(III) center becomes electronically asymmetric due to differential interactions with the indenyl π-system, in stark contrast to the behavior observed in cyclopentadienyl-Rh­(III) complexes. This intrinsic asymmetry structurally manifests as a coordination mode that is better characterized as η^2^+η^3^. Functionally, this unique electronic feature facilitates haptotropic shifts and alters the electronic environment of the metal center, ultimately enhancing catalytic selectivity.

**Figure 2:**
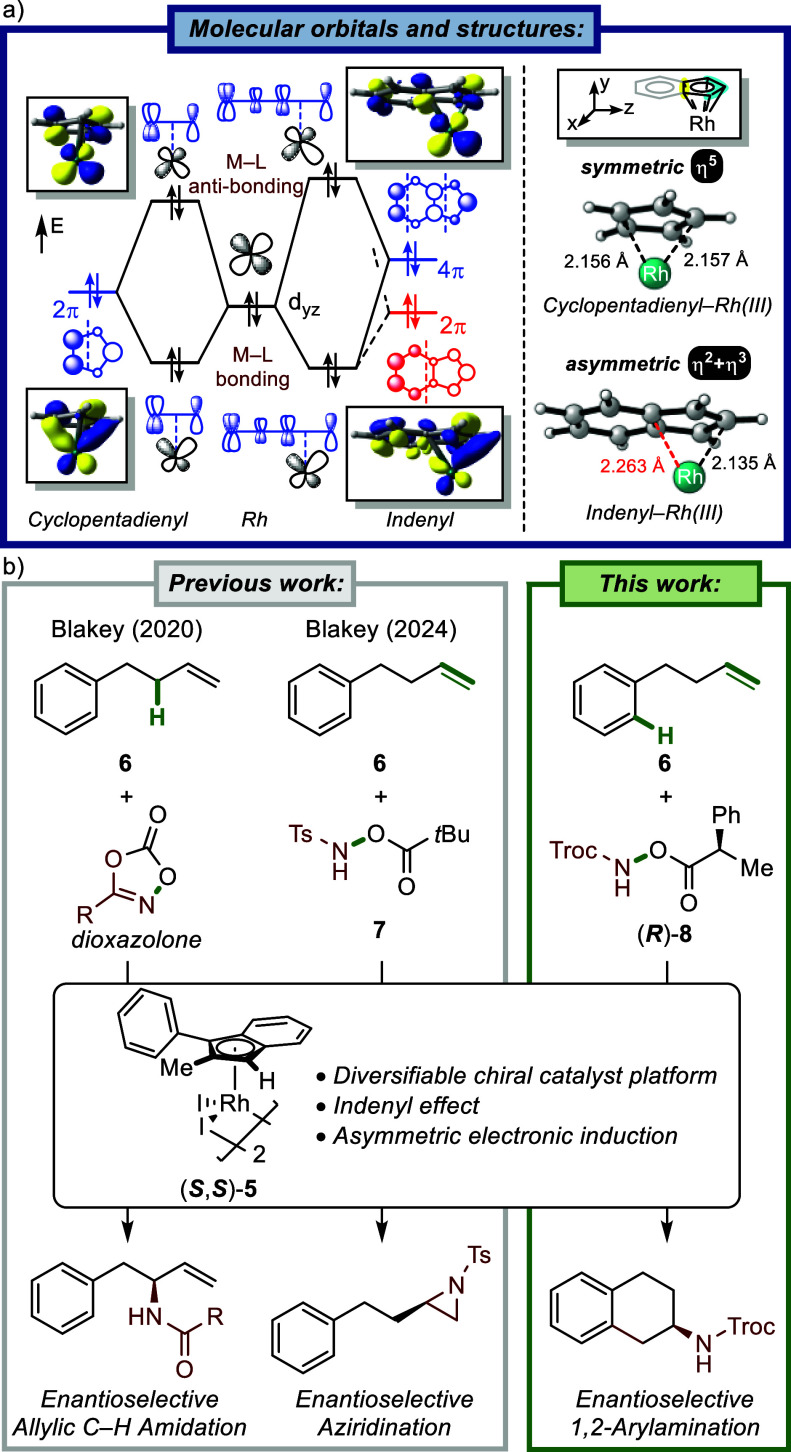
(a) Schematic molecular orbital (MO) diagram and structures of cyclopentadienyl- and indenyl-Rh­(III) complexes. (b) Enantioselective synthesis of nitrogen-containing compounds.

Utilizing this asymmetric indenyl catalyst (**5**), we initially demonstrated regio- and enantioselective allylic C–H amidation of unactivated alkenes such as 4-phenylbutene (**6**) with dioxazolone-derived nitrene precursors (Figure 2b).^8a^ More recently, we expanded the scope of this reaction to enable divergent reactivity, including enantioselective aziridination using a tosyl-protected hydroxylamine (**7**) as a nitrogen source.^8b^ In this work, we further showcase the versatility of this catalyst by achieving enantioselective 1,2-arylamination of unactivated alkenes using a Troc-protected hydroxylamine nitrogen source (**8**). This transformation affords direct access to valuable 2-aminotetralins and nitrogen-substituted spirocycles. Computational studies highlight the crucial role of the indenyl scaffold in enabling this transformation and clarify the divergent chemoselectivity observed with different N-protecting groups, which favors 1,2-arylamination over previously reported aziridination. Finally, analysis of the electrophilic aromatic substitution (EAS) pathway accounts for the observed ortho/ipso selectivity via substituent-controlled directing effects.

## Results and Discussion

### Reaction Discovery and Optimization

During our investigation of alternative nitrogen substituents in the enantioselective aziridination reaction,^8b^ we observed that replacing the standard N-Tosyl group in compound (**7**) with the Troc-protecting group (**9**) led to an unexpected arylamination reaction. When the unactivated alkene (**6**) was subjected to **9** in the presence of [Ind*RhCl_2_]_2_ using the standard aziridination conditions, the formation of 2-aminotetralin (**10**) (58% yield) via a 6-endo-trig cyclization was observed (Figure 3). Notably, no allylic amination (**11**), aziridination (**12**), or 5-exo-trig cyclization products (**13**) were observed. In an initial optimization, we found that the addition of AgNTf_2_ as a halide scavenger and the use of CsOAc enabled the formation of **10** in 83% yield. During the optimization studies, we were surprised to observe no reaction when [Cp*RhCl_2_]_2_ was used as the catalyst, indicating the importance of the indenyl ligand in enabling this transformation. The use of HFIP as the solvent was also important, with other fluorinated solvents such as TFE providing **10** in only 37% yield, while nonfluorinated solvents were ineffective. Other carbamate protected hydroxylamine nitrogen sources were found to be less effective than **9** (see Page S13 in Supporting Information for full details).

**Figure 3:**
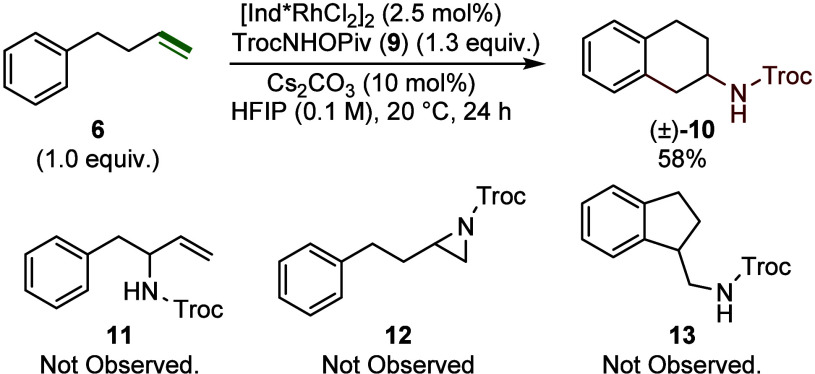
Discovery of the 1,2-arylamination.

Building on our initial understanding of the reaction, we developed an asymmetric transformation using our planar chiral Rh­(III) indenyl catalyst platform. The first-generation catalyst, (***S,S***)-**5**, led to 2-aminotetralin *R*-(**10**)^12^ in an excellent 94% yield with an 86:14 e.r. (Figure 4, entry 1). Substitution of the indenyl ligand with electron-withdrawing trifluoromethyl groups ((***S,S***)-**14**) significantly decreased the yield to 45%, with a negligible impact on enantiocontrol (86:14 e.r.; entry 2). In contrast, the electron-donating substituents such as methoxy ((***S,S***)-**15**), and *tert*-butyl ((***S,S***)-**16**) both afforded high yields (∼90%; entries 3 and 4). However, while the methoxy-substituted catalyst maintained comparable enantioselectivity (87:13 e.r.), the *tert*-butyl group led to a noticeable drop in stereocontrol (81:19 e.r.). The electron-rich pentamethylated catalyst, (***S,S***)-**17**, enhanced enantioselectivity to 91:9 e.r., but decreased the yield to 76% (entry 5).

**Figure 4:**
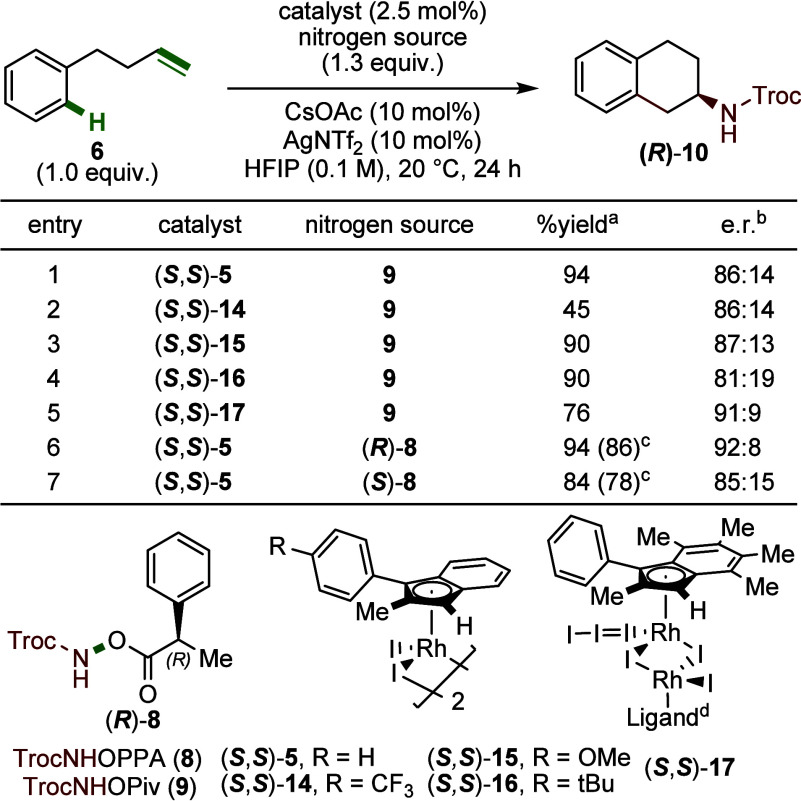
Optimization of the enantioselective 1,2-arylamination reaction. Reactions were performed on a 0.1 mmol scale. ^a^Yields were determined by ^1^H NMR spectroscopy using dibromomethane (0.1 mmol) as an NMR standard. ^b^Enantiomeric ratios were determined by chiral HPLC on a Chiralpak IJ column (5% isopropanol in hexanes). ^c^Isolated yields. ^d^Ligand = 1-phenyl-2,4,5,6,7-pentamethyl indenyl.

Although (***S,S***)-**17** offered improved enantioselectivity, it is more difficult to access than (***S,S***)-**5**. Additionally, there were challenges associated with purifying the products away from nitrogen source **9**. To address these limitations, we replaced the original pivalate leaving group in **9** with a chiral 2-phenylpropionic acid (**8**). Pairing the resulting chiral nitrogen source (***R***)-**8** with the (***S,S***)-**5** significantly improved enantioselectivity, affording product **10** in 94% yield and 92:8 e.r. (entry 6).^13^ In contrast, a mismatched pairing using (***S***)-**8** reduced the yield to 84% and e.r. to 85:15, highlighting a cooperative effect between the catalyst and nitrogen source (entry 7).

With the optimized enantioselective conditions, we performed control experiments to gain insight into the cyclization mechanism. Para-methoxy-substituted alkene **18** afforded two regioisomeric products, 7-substituted 2-aminotetralin **19a** and 6-substituted 2-aminotetralin **19b** (Figure 5a). The combined yield of these regioisomers was 65%, with **19b** predominating in a 4:1 ratio. Both regioisomers were formed with high enantioselectivity (92:8 e.r. for **19a**, 91:9 e.r. for **19b**). These results suggest the presence of two competing cyclization pathways that account for the observed regioselectivity (Figure 5b). A direct 6-endo-trig cyclization at the ortho-position would generate **19a**. Alternatively, a 5-endo-trig cyclization at the ipso-position forms a spirocyclic intermediate **18′**, stabilized by resonance with the para-methoxy substituent. A subsequent 1,2-alkyl-shift at C4 then yields the major regioisomer **19b**. A competing 1,2-shift at C1 is also conceivable and would lead to **19a**, though this appears less favorable under the reaction conditions. Similar rearrangement-based regioselectivity has been reported by the Chang group, in which spirocyclic γ-lactams underwent selective 1,2-shifts at electronically deactivated carbons.^14^ Furthermore, a para-trifluoromethyl-substituted alkene showed no reactivity under identical conditions, consistent with an electrophilic aromatic substitution (EAS) mechanism.

**Figure 5:**
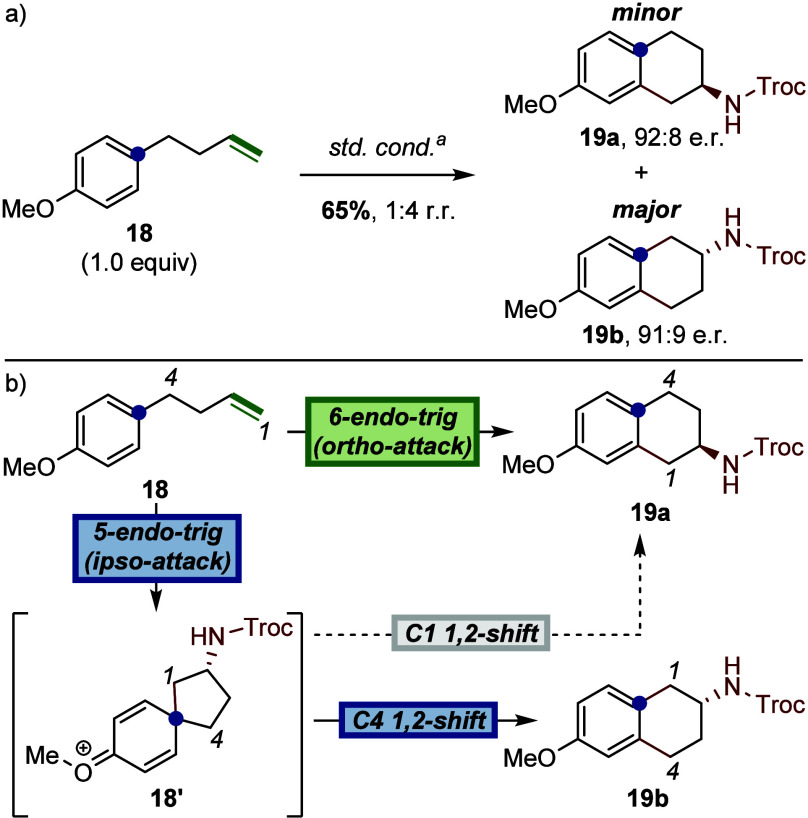
a) Observation of 2-aminotetralin regioisomers. b) Proposed divergent cyclization pathways. Isolated yield. Regiomeric ratios were determined via ^1^H NMR spectroscopy of the crude reaction material. Enantiomeric ratios were determined by chiral SFC on a Chiralcel OJ-3 column (5% Methanol in isopropanol with 0.2% formic acid). ^a^Standard conditions: (*S,S*)-5 (2.5 mol %), (*R*)-8 (1.3 equiv), CsOAc (10 mol %), AgNTf_2_ (10 mol %), HFIP (0.1 M), 20 °C, 24 h.

### Scope

We next explored the scope of the cyclization reaction by varying the substituents on the aromatic ring. Substrates bearing strong electron-donating groups that activate the ipso position toward electrophilic aromatic substitution (EAS) predominantly generated the rearranged products via ipso-attack (**19–21**, Figure 6). In contrast, substrates with more modestly activating groups (**22**–**24**) or those favoring activation at the ortho position (**25**–**27**) gave products in which the major regioisomer was formed by direct 6-endo attack from the ortho position. Notably, the phenolic allyl derivative failed to undergo cyclization, likely due to inductive deactivation of the alkene by the oxygen substituent. In the case of the butenyl olefin (**21**), the reaction proceeded with excellent chemoselectivity, affording the desired product in 54% yield with a 4:1 regioisomeric ratio and 91:9 e.r.

**Figure 6:**
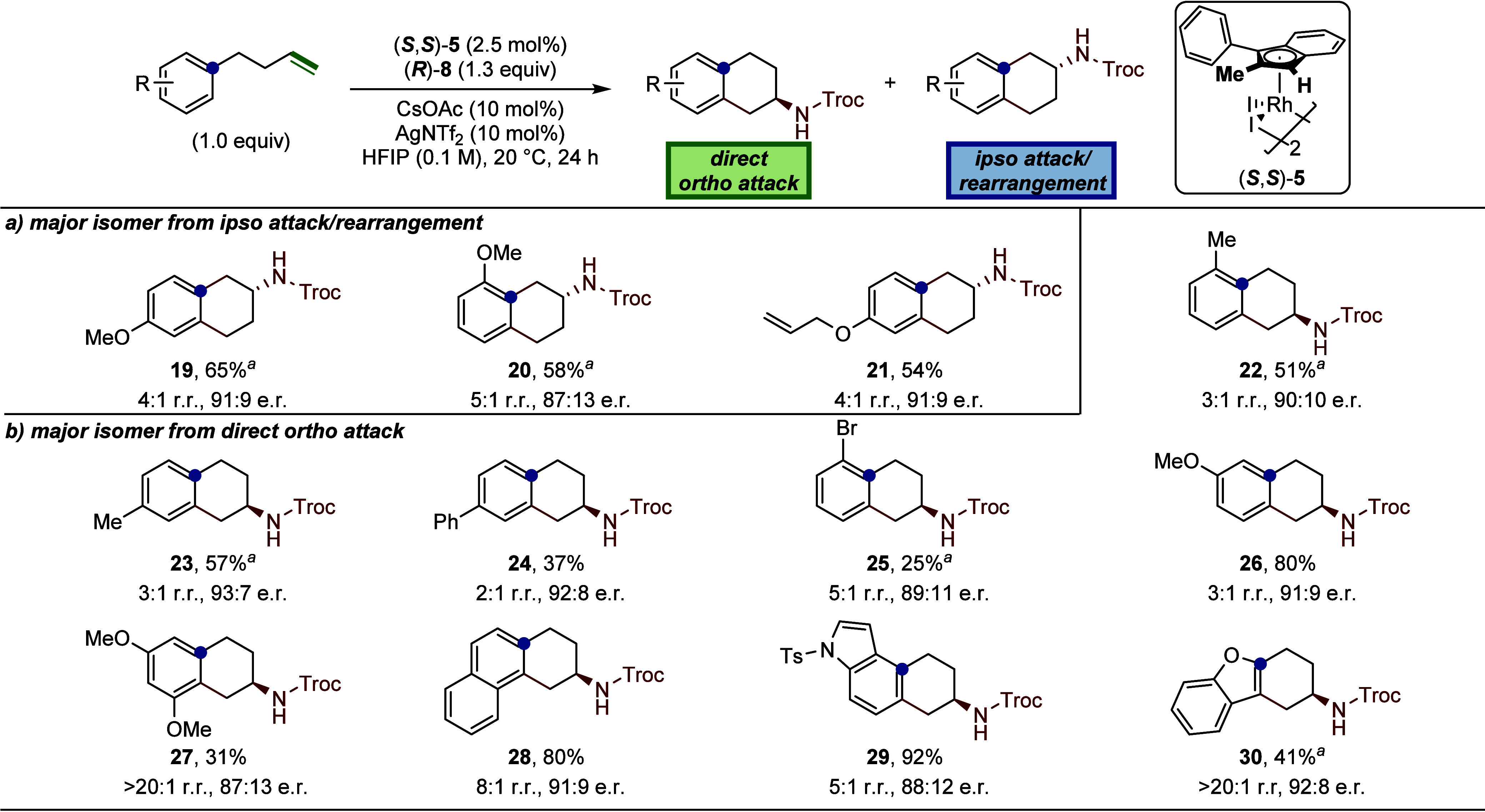
Scope of 2-aminotetralins. Reactions were run on a 0.1 mmol scale. Isolated yields are reported. Major regioisomer products are shown and the regiomeric ratios were determined via ^1^H NMR spectroscopy of the crude reaction material. Enantiomeric ratios were determined by chiral HPLC and SFC (see Section 13 in Supporting Information for details). ^a^Reaction was run for 48 h. a) substrates in which the major product arises from a 5-endo cyclization at the ipso position followed by rearrangement. b) substrates in which the major product arises from a direct 6-endo cyclization at the ortho position.

A substrate bearing moderately electron-withdrawing substituent, such as bromine (**25**), led to reduced reactivity in 25% yield (5:1 r.r., 89:11 e.r.). As previously noted, strongly electron-withdrawing groups such as trifluoromethyl completely suppressed the reaction (see Page S24 in Supporting Information for details). The meta-methoxy substrate provided **26** as the major regioisomer in 80% yield with a 3:1 r.r. and 91:9 e.r. In this case, the formation of regioisomers reflects the presence of nonequivalent ortho positions in the meta-substituted aromatic ring. Cyclization of a 2-substituted-naphthyl substrate provided the bent product **28** in an 80% yield with 8:1 r.r. and 91:9 e.r.. The minor regioisomer in this substrate was identified as the rearranged bent product via 5-endo-trig cyclization. Notably, no linear products were observed under these conditions. Nucleophilic heterocycles were also evaluated. The 4-substituted indole underwent regioselective cyclization at the C5 position to generate product **29** in 92% yield with 5:1 r.r. and 88:12 e.r. In contrast, attempts to direct cyclization to the more nucleophilic C3 position were unsuccessful (see Page S24 in Supporting Information for details). Finally, the benzofuran-derived substrate afforded product **30** in 41% yield as a single regioisomer (>20:1 r.r.) with a 92:8 e.r., demonstrating the compatibility of oxygen-containing heterocycles under the optimized conditions.

We targeted the dearomatized spirocyclic intermediates to isolate these species as stable, nitrogen-containing three-dimensional synthons (Figure 7). Cyclization of a TMS-protected para-phenol afforded the [4.5]-spirocycle **31** in a 63% yield with 89:11 e.r., accompanied by trace amounts of the corresponding 2-aminotetralin product. A 2-naphthol-derived substrate, incapable of undergoing 6-endo-trig cyclization, provided exclusive access to the [4.5]-spirocycle **32** in 73% yield with 1.9:1 d.r. and 88:12 e.r., representing a rare example of asymmetric dearomatization. Extending the carbon linker by one methylene unit enabled access to [5.5]-spirocycles. The para-phenol substrate yielded **33** in 82% yield and 91:9 e.r. via 6-endo-trig cyclization at the ipso position. The 2-naphthol-derived [5.5]-spirocycle **34** was obtained in 43% yield with a 4:1 d.r. and 88:12 e.r. The 8-methoxy-substituted substrate afforded spirocycle **35** in a 37% yield as a single diastereomer (>20:1 d.r.) with 89:11 e.r. In contrast, the 7-methoxy variant **36** gave the major diastereomer in a 32% yield, exhibiting reduced stereoselectivity (1.2:1 d.r. and 81:19 e.r.).

**Figure 7:**
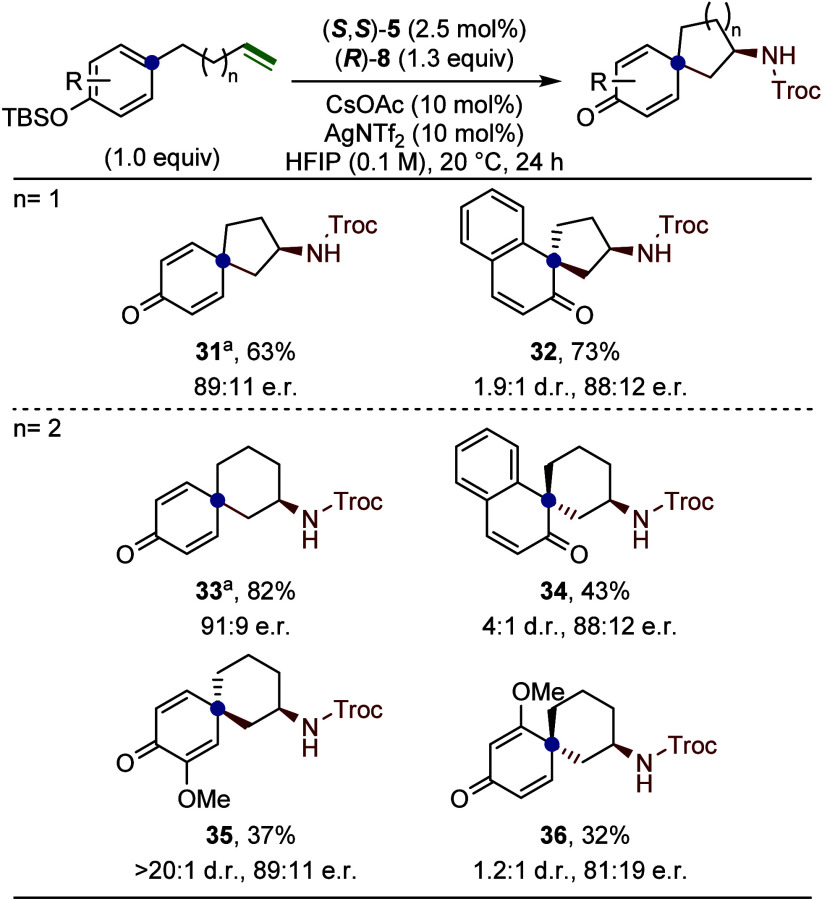
Synthesis of amino substituted spirocycles. Reactions were run on a 0.1 mmol scale. Isolated yields are reported. Diastereomeric ratios were determined via ^1^H NMR spectroscopy of the crude reaction material. Enantiomeric ratios were determined by chiral HPLC (see Section 13 in Supporting Information for details). ^a^TMS was used instead of TBS as the silyl ether protecting group.

When the methoxy-substituted compound **37** was subjected to the standard reaction conditions, the expected tetrahydroazulene product **38**–resulting from ipso-attack and subsequent rearrangement–was not observed. Instead, the bridged bicyclic product **39** was isolated in 15% yield with 89:11 e.r. (Figure 8a). This transformation is proposed to proceed via a 1,4-Michael-type addition to the activated spirocyclic intermediate **37′**. To validate this hypothesis, we synthesized spirocycle **33** on a 1.0 mmol scale using **S14** as the substrate, obtaining it in 73% yield and 91:9 e.r. (Figure 8b). Treatment of **33** under acidic conditions led to **39** in 70% yield, supporting the proposed pathway. Finally, to demonstrate the synthetic utility of this 1,2-arylamination platform, we applied this strategy to the preparation of the 5-HT_1A_ agonist 8-OH-DPAT (**1**). Scale-up of the 1,2-arylamination on a 1.00 mmol scale provided **20** in a 66% yield and 88:12 e.r. (Figure 8c). Subsequent Troc deprotection using Zn/AcOH generated free amine **40** in 72% yield. Reductive amination with propionic acid, followed by demethylation, afforded the target compound **1** in 36% yield, completing a concise four-step synthesis from alkene **S1.**

**Figure 8:**
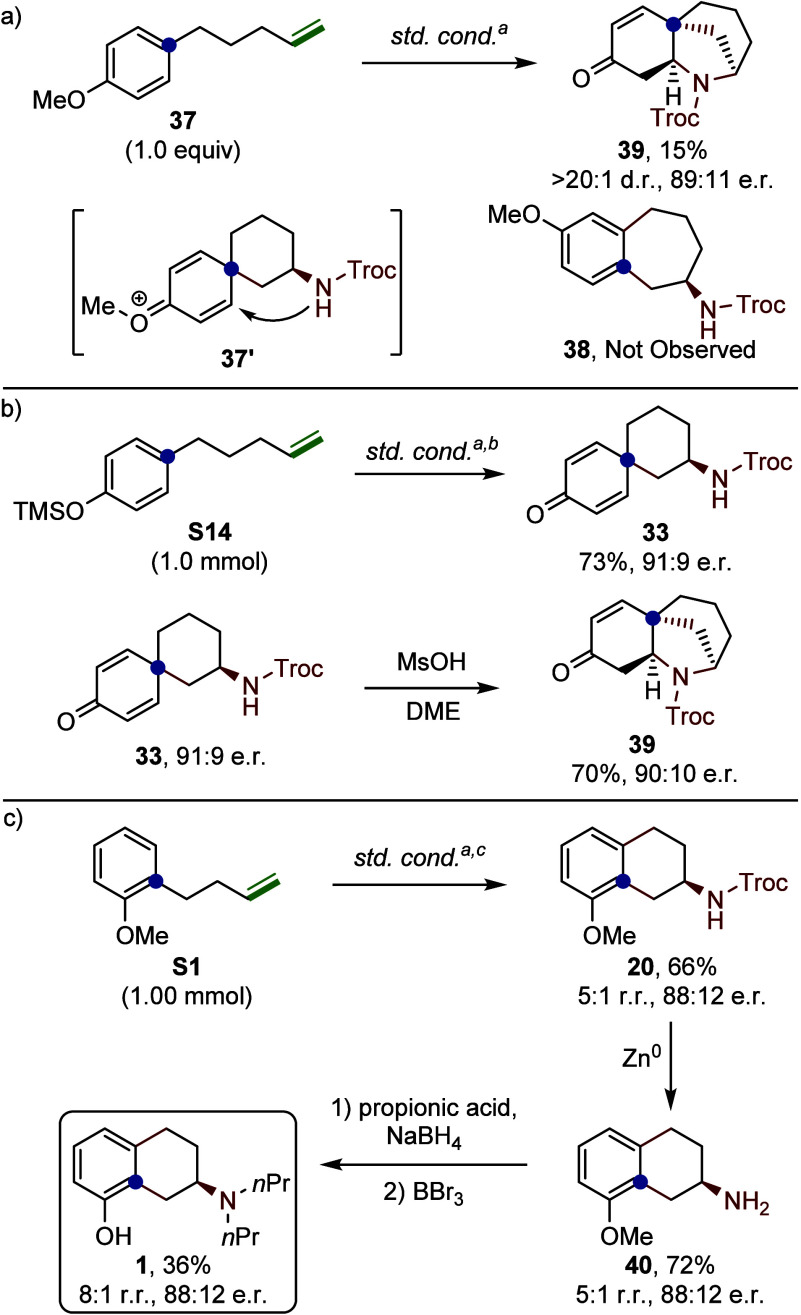
Synthesis of bridged azepine and synthetic utility of the 1,2-arylamination. Isolated yields are reported. Diastereomeric ratios were determined via ^1^H NMR spectroscopy of the crude reaction material. Enantiomeric ratios were determined by chiral HPLC (see Section 15 in Supporting Information for details). ^a^Standard conditions: (*S,S*)-5 (2.5 mol %), (*R*)-8 (1.3 equiv), CsOAc (10 mol %), AgNTf_2_ (10 mol %), HFIP (0.1 M), 20 °C, 24 h. ^b^48 h ^c^(S,S)-5 used in 5 mol %, 72 h.

### Reaction Mechanism

Having explored the scope of 2-aminotetralin and spirocycle synthesis, we next sought to investigate the reaction mechanism in greater detail. Based on the observed reactivity preference for electron-rich aromatic rings and regioselectivity between electron-rich ipso- and ortho-positions, we propose that the C–C bond formation proceeds via EAS mechanism. This is supported by the lack of deuterium scrambling when **6-** ***d***_***5***_ is subjected to the reaction conditions, affording **10-** ***d***_***4***_ in 63% yield, which indicates that C–H cleavage is irreversible (Figure 9a). Further support comes from the absence of a significant kinetic isotope effect (KIE = 1.00 ± 0.05) in an intermolecular competition experiment between **6** and **6-** ***d***_***5***_, suggesting that C–H cleavage occurs after the rate limiting step (Figure 9b).^15^ To identify the electrophilic intermediate undergoing nucleophilic attack from the pendant aryl group, we considered an aziridine intermediate–motivated by the mechanistic similarities to our previously reported enantioselective aziridination.^8^ Additionally, EAS ring-opening of aziridines to generate 2-aminotretralins has precedent in the literature.^16^ However, no aziridine intermediates or products were observed when either **7** or 1-nonene were subjected to the reaction conditions, indicating that aziridination does not occur. Moreover, independently synthesized aziridine **12** failed to generate **10** under standard conditions (Figure 9c). A π-allyl intermediate was also considered but deemed unlikely, as no deuterium scrambling was observed when the allylic deuterated substrate **6-** ***d***_***2***_ was subjected to the reaction conditions, forming **10-** ***d***_***2***_ in 95% yield (Figure 9d). When monodeuterated alkene *E* **-6-** ***d***_***1***_ was subjected to the standard reaction conditions, the product retained nearly complete deuterium stereochemistry (95:5, anti:syn), indicating antiaddition of the aryl and nitrogen groups across the olefin. This result, along with the absence of stereochemical scrambling, argues against a pathway involving a carbocation intermediate at C1 (Figure 9e).

**Figure 9:**
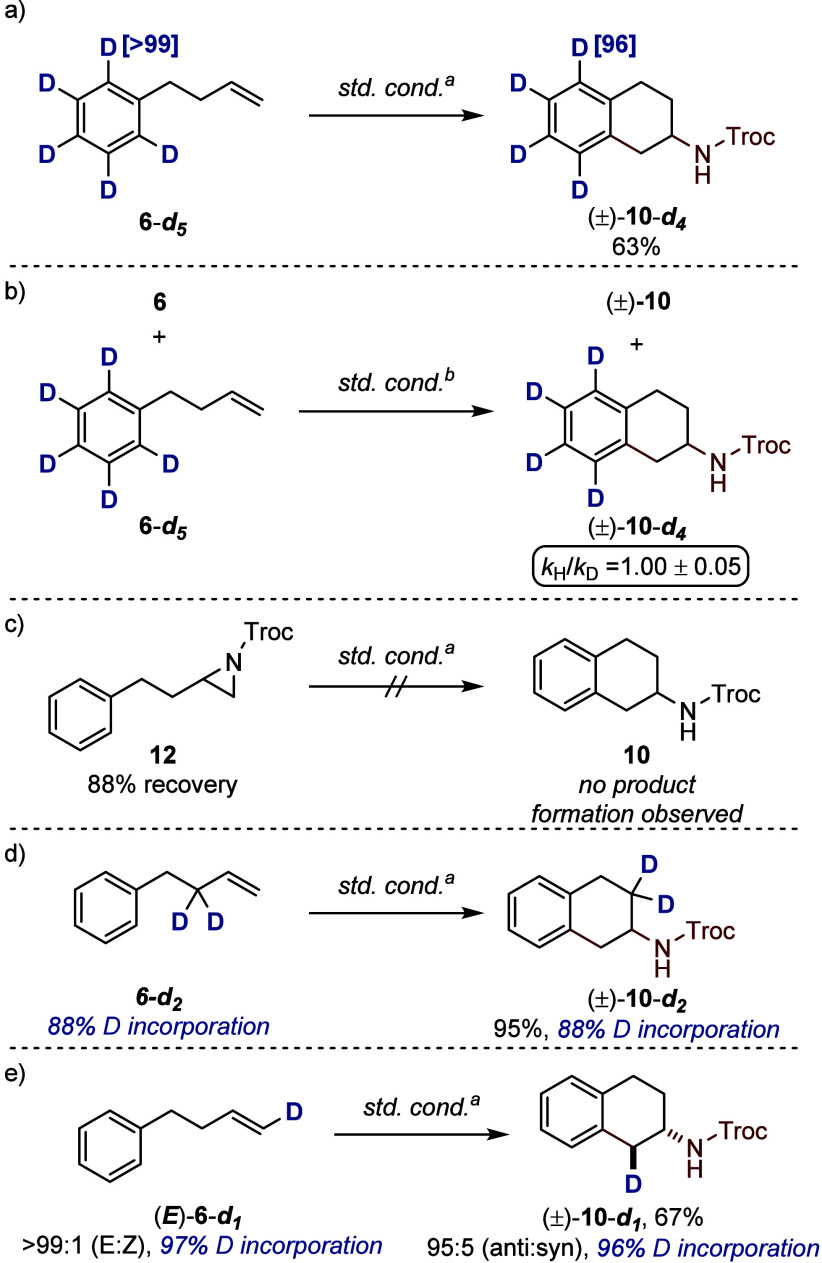
Mechanistic investigations. Reactions were run on a 0.1 mmol scale. Isolated yields are reported. ^a^Standard conditions: [Ind*RhCl_2_]_2_ (2.5 mol %), (±)-8 (1.3 equiv), CsOAc (10 mol %), AgNTf_2_ (10 mol %), HFIP (0.1 M), 20 °C, 24 h. ^b^Reaction was run for 6 h.

### Computational Study

To establish a plausible reaction mechanism and gain insights into tetralin formation catalyzed by the chiral indenyl rhodium complex **(** ***S***, ***S*** **)**-**5**, denoted as **IndRh** hereafter, density functional theory (DFT) calculations were conducted at the B3LYP-D3/def2-TZVPP//B3LYP-D3/def2-SVP­(def2-TZVP for Rh) level of theory with CPCM solvation in HFIP (see Section 19 in Supporting Information for computational details).^17^ As illustrated in Figure 10, the catalytic cycle starts with the 16-electron complex **A1**, formed upon activation of the dimeric precatalyst with 4-phenylbutene (**6**) and CsOAc, which serve as the olefin substrate and base, respectively. Subsequent addition of Troc-protected hydroxylamine (**9**) and concomitant release of acetic acid yields the rhodium-amide complex **A2**, a resting intermediate found at – 13.4 kcal/mol.

**Figure 10:**
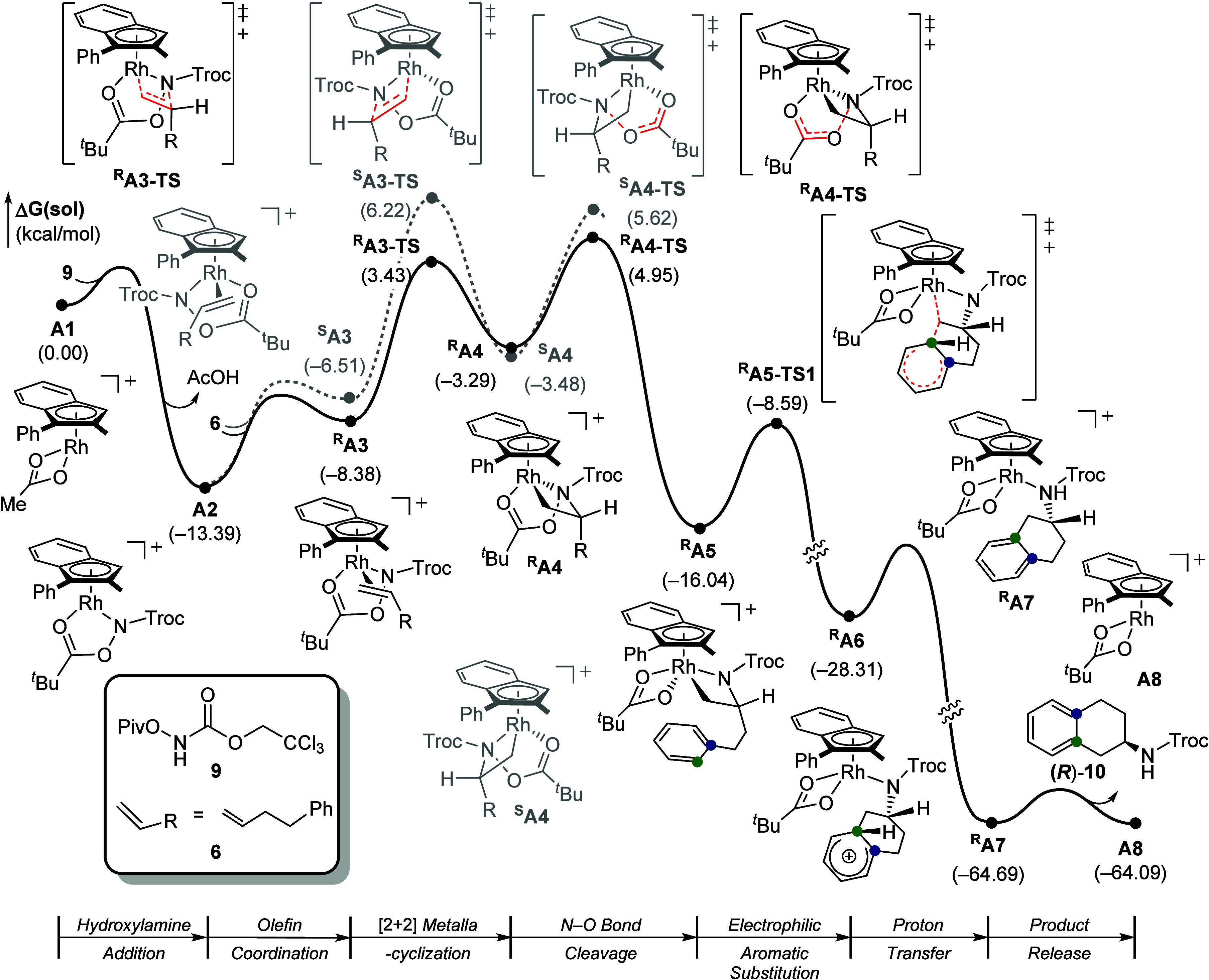
DFT-calculated energy profile of IndRh-catalyzed enantioselective 1,2-arylamination.

Olefin coordination to **A2** forms two diastereomeric intermediates, ^**R**^ **A3** and ^**S**^ **A3**, with relative energies of – 8.4 and – 6.5 kcal/mol, respectively. This coordination step defines the divergence between the (*R*)- and (*S*)-reaction pathways. Each pathway subsequently undergoes [2 + 2] metallacyclization through its corresponding transition state, ^**R**^ **A3-TS** or ^**S**^ **A3-TS**. The transition state ^**R**^ **A3-TS** is favored by 2.8 kcal/mol over ^**S**^ **A3-TS**. As previously noted, this disfavor on (*S*)-pathway originates from steric repulsion between the phenyl substituent on the chiral indenyl ligand and Troc group in ^**S**^ **A3-TS** (see Figure S1 for a detailed discussion).^8b^

The resulting Rh­(III) 18-electron intermediates ^**R**^ **A4** and ^**S**^ **A4** undergo N–O bond cleavage through ^**R**^ **A4-TS** and ^**S**^ **A4-TS**, respectively. In the (*R*)-pathway, ^**R**^ **A4-TS** represents the turnover-limiting step with an associated barrier of 18.3 kcal/mol. In contrast, in the (*S*)-pathway, ^**S**^ **A4-TS** is higher in energy than ^**R**^ **A4-TS** by 0.7 kcal/mol but remains below ^**S**^ **A3-TS**. Consequently, the highest point on the (*S*)-pathway is ^**S**^ **A3-TS** at 19.6 kcal/mol. Accordingly, the enantioselectivity is governed by the difference between the highest-energy transition states of the two pathways–^**R**^ **A4-TS** for the (*R*)-pathway and ^**S**^ **A3-TS** for the (*S*)-pathway. This 1.3 kcal/mol energy difference renders the (*R*)-pathway more favorable, consistent with the experimentally observed (*R*)-selectivity.

This transformation generates the intermediate ^**R**^ **A5**, bearing an electron-deficient Rh­(V) center, which withdraws electron density from adjacent ligands. This polarization renders the alkyl carbon directly bound to Rh highly electrophilic and susceptible to intramolecular attack by the aryl group, facilitating a 6-endo cyclization through ^**R**^ **A5-TS1**. The barrier for this C–C bond-forming step is 7.4 kcal/mol. The resulting arenium intermediate ^**R**^ **A6** undergoes rapid deprotonation, transferring the proton to the Troc-protected amido group to yield the final Troc-protected tetralin. Dissociation of the tetralin product from ^**R**^ **A7** regenerates the active catalyst species ^**R**^ **A8**, completing the catalytic cycle with an overall exergonic energy change of – 64.1 kcal/mol.

Notably, [Cp*RhCl_2_]_2_ exhibited no catalytic activity under otherwise identical conditions, underscoring the critical role of the indenyl ligand. To elucidate the ligand effect, we investigated an analogous reaction pathway using [Cp*RhCl_2_]_2_, denoted as Cp*Rh hereafter, as the catalyst precursor. Although both Cp*Rh and **IndRh** follow the same general mechanistic sequence, significant energetic differences emerge during the [2 + 2] metallacyclization and N–O bond cleavage steps (Figure 11). For Cp*Rh, the [2 + 2] metallacyclization via ^**R**^ **B3-TS** and the N–O bond cleavage via ^**R**^ **B4-TS** proceed with high activation barriers of 26.7 and 28.7 kcal/mol, respectively. These substantially elevated barriers suggest that Cp*Rh is unlikely to facilitate the reaction efficiently under standard reaction conditions, consistent with the experimentally observed lack of reactivity. The poor catalytic performance of Cp*Rh can be attributed to the instability of the key intermediate ^**R**^ **B4**, which lies 18.9 kcal/mol above the resting state **B2**. In contrast, the corresponding intermediate ^**R**^ **A4** in the **IndRh** system is only 10.1 kcal/mol higher in energy than **A2**, making the **IndRh** pathway considerably more energetically favorable.

**Figure 11:**
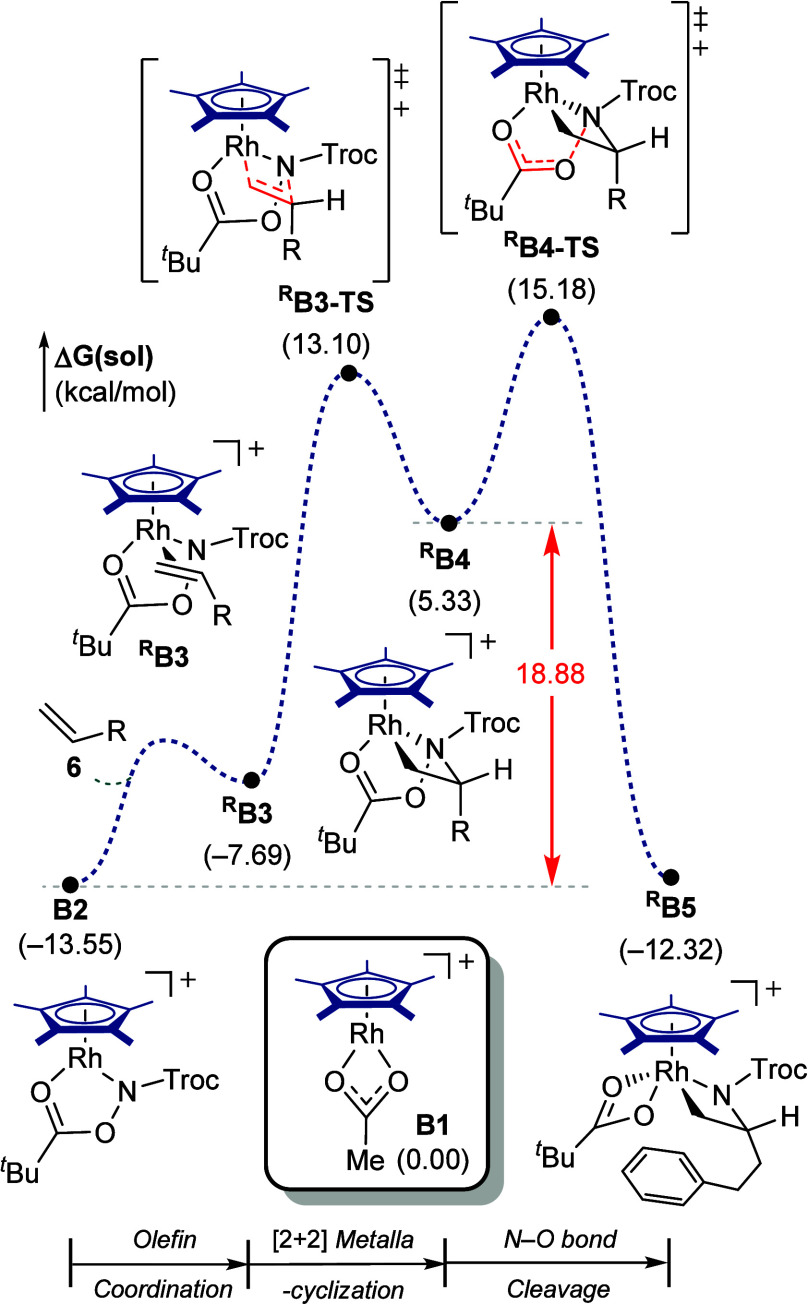
DFT-calculated energy profile of Cp*Rh-catalyzed 1,2-arylamination.

To clarify the origin of these energetic differences, we analyzed how the indenyl and Cp* ligands respond structurally during the transformation from **X2** to ^**R**^ **X4** (**X** = **A** for **IndRh**, and **B** for Cp*Rh). As shown in Figure 12a, this transformation involves the conversion of the 16-electron complex **X2** to the 18-electron complex ^**R**^ **X4** via [2 + 2] metallacyclization. Introduction of the alkyl ligand induces pronounced structural distortions, particularly elongating the Rh–C bonds at the η^2^ site opposite to the alkyl group, resulting in an asymmetric η^2^+η^3^ binding mode in ^**R**^ **X4**. For Cp*Rh, the initial complex **B2** displays nearly symmetric η^5^ coordination, with average Rh–C bond lengths of 2.178 Å (η^2^) and 2.163 Å (η^3^). In ^**R**^ **B4**, the η^2^-Rh–C bonds elongate by ∼ 0.12 Å. In contrast, the **IndRh** complex **A2** already shows inherent asymmetry even before metallacyclization, with η^2^-Rh–C and η^3^-Rh–C bond lengths of 2.254 and 2.155 Å, respectively. This asymmetry further increases in ^**R**^ **A4**, with an even greater elongation of 0.160 Å at the η^2^ site. Remarkably, despite the larger structural distortion in **IndRh**, the associated energy penalty is smaller than in Cp*Rh, suggesting that indenyl ligands confer greater structural flexibility and reduced energetic sensitivity to hapticity changes.

**Figure 12:**
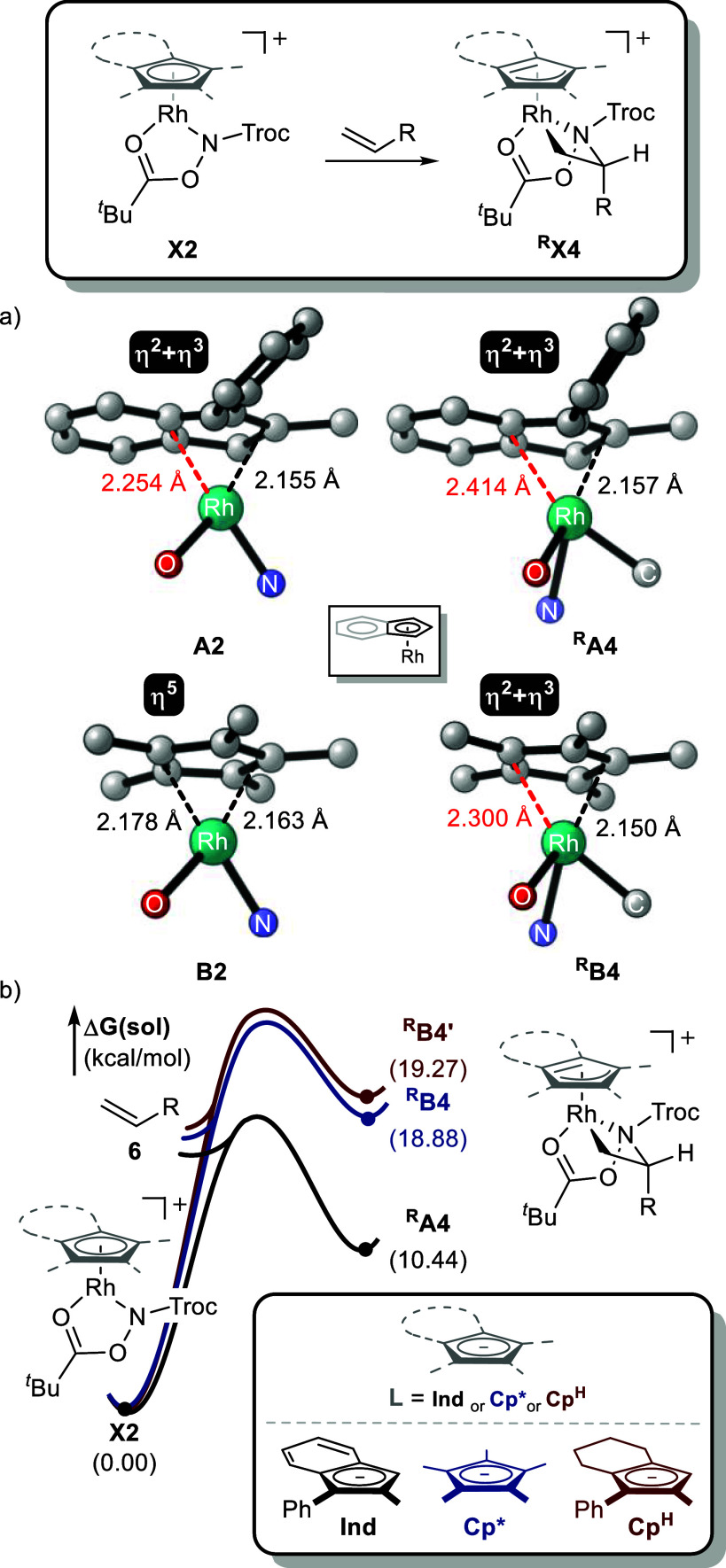
Structural and energetic comparison of IndRh and Cp*Rh complexes. (a) DFT-optimized structures^a,b^ and (b) relative free energy differences of X2 and ^R^X4 (X = A for IndRh, B for Cp*Rh, and B’ for Cp^H^Rh). ^a^Unnecessary atoms are omitted for clarity. ^b^Average η^2^- and η^3^-Rh–C lengths are shown.

As discussed in Figure 2a, this flexibility originates from the intrinsic asymmetry of the indenyl ligand, conferred by its extended π-system. This asymmetry weakens the initial metal–ligand (M–L) interaction, thereby lowering the energetic cost of structural rearrangement during η^5^ → η^2^+η^3^ hapticity shift (see Figure S2 for distortion-interaction analysis). To isolate the contributions of π-conjugation, we evaluated a model ligand (**Cp** ^**H**^) that mimics the steric properties of indenyl but lacks the extended π-conjugation, achieved by saturating the sp^2^ carbons (C4–C7) of the six-membered ring (Figure 12b). The energy cost associated with **Cp** ^**H**^ **Rh** (19.3 kcal/mol) was comparable to Cp*Rh but significantly higher than for **IndRh**, highlighting the critical role of π-conjugation in reducing the energetic penalty associated with structural distortion. Thus, the inherently weaker M–L interactions in **IndRh** facilitate energetically favorable hapticity shifts,enabling more efficient transitions from **A2** to ^**R**^ **A4**. This structural adaptability stabilizes reactive intermediates and lowers activation barriers, ultimately endowing **IndRh** complexes with superior electronic and structural properties that enhance catalytic performance.

Experimental results demonstrated that the choice of the *N*-protecting group has a profound influence on reaction chemoselectivity. Troc-protected hydroxylamine **9**, denoted as *N* **-Troc** hereafter, predominantly affords tetralin products, whereas tosyl-protected hydroxylamine **7**, denoted as *N* **-Ts** hereafter, favors aziridine formation.^8b^ To understand this divergence, we computationally examined two competing intramolecular pathways originating from Rh­(V) intermediates ^**R**^ **X5** (**X** = **A** for *N* **-Troc**, and **C** for *N* **-Ts**), involving either electrophilic aromatic substitution via ^**R**^ **X5-TS1** or intramolecular nucleophilic attack by nitrogen via ^**R**^ **X5-TS2** (Figure 13a). In the *N*-Troc system, EAS is favored with a lower activation barrier of 7.5 kcal/mol compared to aziridination, which is associated with a barrier of 9.7 kcal/mol. Conversely, the *N* **-Ts** substrate ^**R**^ **C5** favors aziridination, which proceeds with a barrier of 6.1 kcal/mol, lower than the barrier of 8.9 kcal/mol required for EAS. To rationalize these differences, we analyzed the electronic properties of ^**R**^ **A5** and ^**R**^ **C5**, hypothesizing that the inductive effect of the *N*-protecting group modulates the nitrogen nucleophilicity. Natural bond orbital (NBO) population analysis supports this hypothesis: the nitrogen in ^**R**^ **C5** bears a more negative charge of – 0.721 compared to ^**R**^ **A5** (−0.490), indicating increased nucleophilicity in the *N* **-Ts** system, which promotes aziridination. The electron-withdrawing Troc group, by contrast, reduces nitrogen nucleophilicity in ^**R**^ **A5**, thereby disfavoring aziridination and shifting the selectivity toward the more electrophilically driven EAS pathway. Further molecular orbital analysis of ^**R**^ **X5** corroborates this trend, showing that the Troc group stabilizes the LUMO of the Rh­(V) intermediates, enhancing its electrophilicity and favoring arylation over nitrogen attack (see Figure S4). These computational findings align closely with experimental observations, establishing the *N*-protecting group as a key determinant of chemoselectivity in this system.

**Figure 13:**
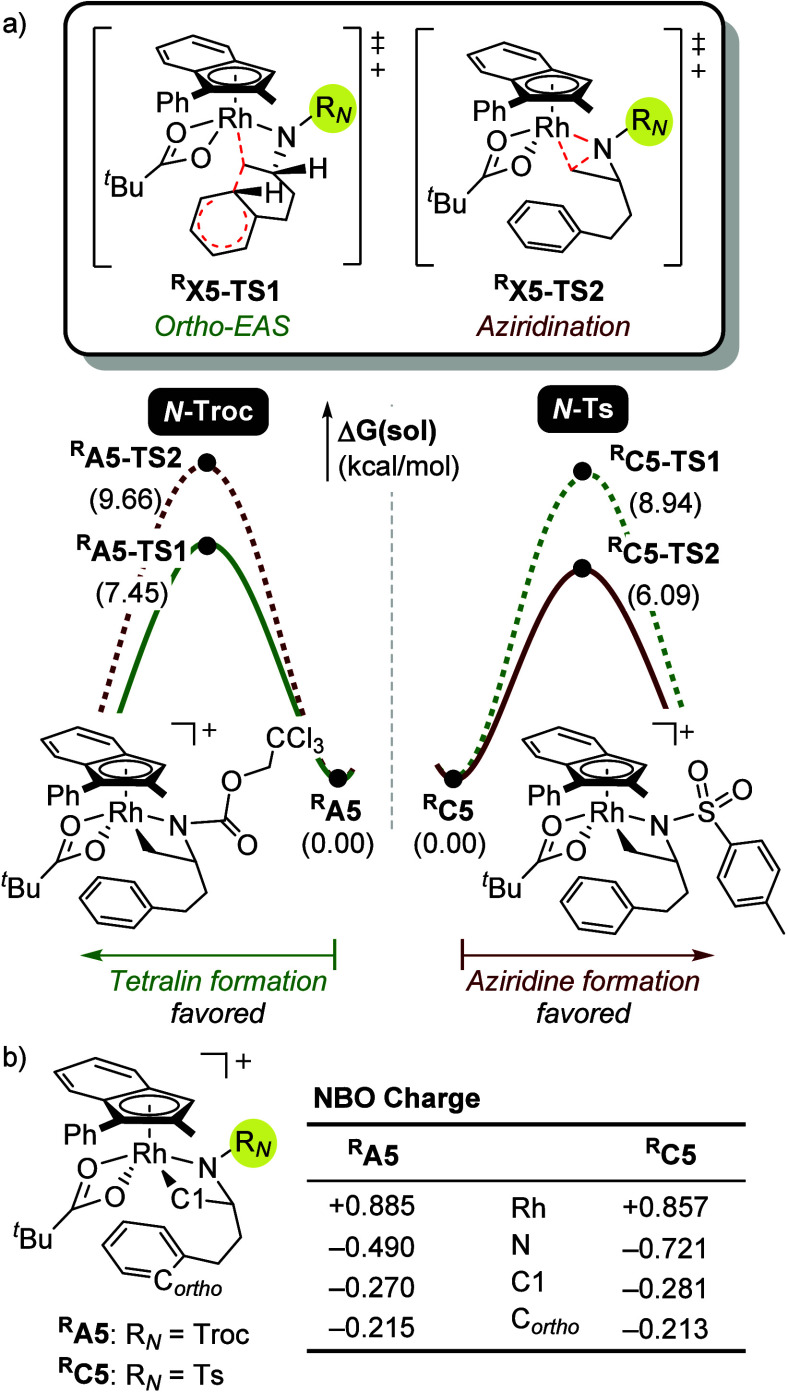
Comparison of tetralin and aziridine formation depending on the *N*-protecting groups. (a) relative free energy barriers of ^R^X5-TS1 and ^R^X5-TS2, and (b) natural bonding orbital charge analysis of ^R^X5 (X = A for *N*-Troc, and C for *N*-Ts).

To investigate regioselectivity along the EAS pathway, we compared the parent complex ^**R**^ **A5** (*p*-H) with the para-silyloxy analogue ^**R**^ **D5** (*p*-OTMS). Figure 14 summarizes the relative stabilities of the ortho- and ipso-arenium intermediates (^**R**^ **X6** and ^**R**^ **X10**; **X** = **A** for H, **D** for OTMS). In ^**R**^ **A5**, the ipso-alkyl substituent drives an ortho/para-directing behavior by electronically favoring substitution at C_*ortho*_ relative to C_*ipso*_. Accordingly, the ortho-arenium ^**R**^ **A6** is more stable than the ipso-arenium ^**R**^ **A10** by 10.7 kcal/mol. In contrast, introduction of the stronger electron-donating silyloxy group in ^**R**^ **D5** overrides this bias, yielding an ipso/meta-directing effect. The ipso-arenium ^**R**^ **D10** is more stable than ^**R**^ **D6** by 4.9 kcal/mol. This inversion is attributed to resonance effect of the silyloxy substituent, which redistributes reactivity across the aryl ring. As shown in Figure S6, ^**R**^ **A5** undergoes exclusively ortho-selective C–C bond formation via a 6-endo cyclization. By comparison, the silyloxy-substituted ^**R**^ **D5** can evolve into ^**R**^ **D10** through two routes: (i) direct ipso-selective arylation via a 5-endo cyclization or (ii) ortho-selective arylation via a 6-endo cyclization followed by a rearrangement to the spirocyclic intermediate. These findings are consistent with established EAS trends and underscore the role of substituent electronics in modulating regioselectivity. Additional calculations on carbospirocycle formation and the regioselective 1,2-shift pathways are provided in Figure S7 and S8.

**Figure 14:**
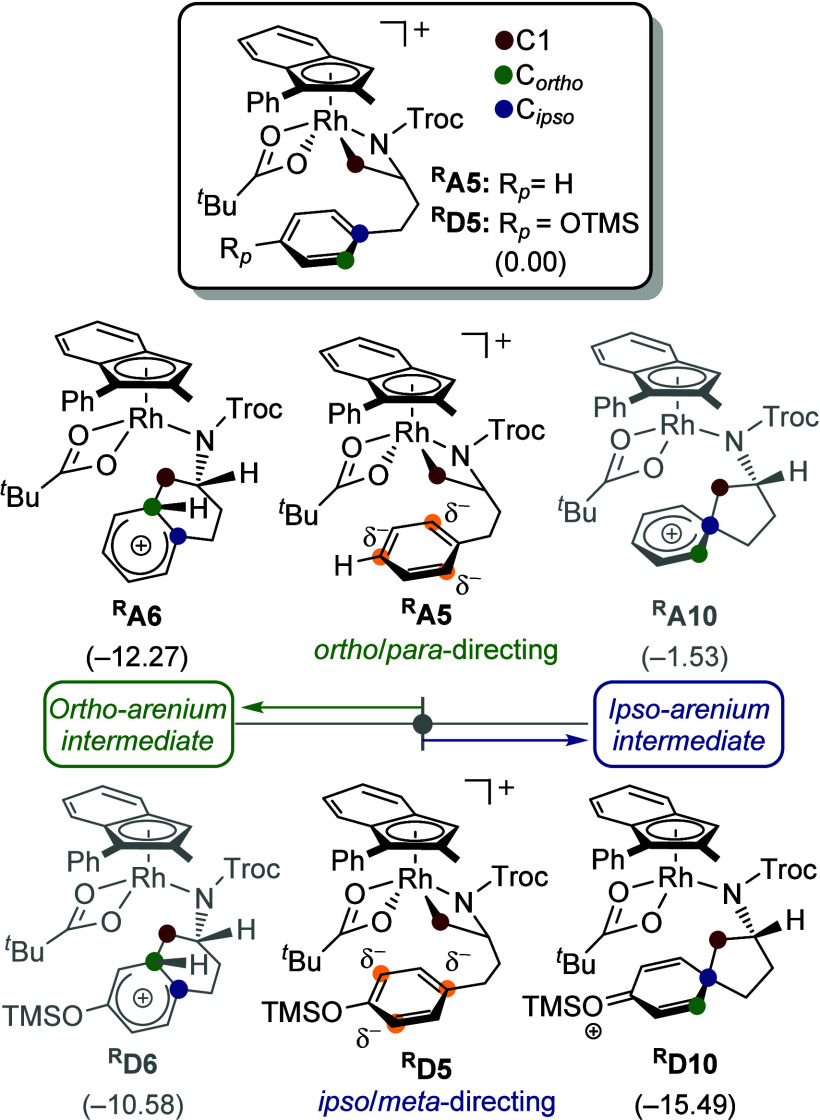
Comparison stabilities of ortho/ipso-arenium intermediates depending on the para-substitution groups of phenyl group. All Gibbs energies are given in kcal/mol.

## Conclusion

We have developed an enantioselective 1,2-arylamination reaction of unactivated alkenes catalyzed by a chiral indenyl-Rh­(III) catalyst. This method enables efficient access to structurally diverse 2-aminotetralins and spirocycles. Mechanistic investigations, complemented by DFT calculations, highlight the critical role of the indenyl ligand’s electronic asymmetry in enabling the reaction. In particular, we elucidate an electrophilic aromatic substitution pathway from a Rh­(V) intermediate and demonstrate how the nature of the *N*-protecting group governs chemoselectivity. Comparative studies with Cp*Rh complexes further elucidate the essential contribution of the indenyl ligand in alleviating the instability of the 18-electron Rh­(III) intermediate formed after [2 + 2] metallacyclization, wherein the new Rh–C­(sp^3^) bond is formed. The ability of the indenyl ligand to accommodate the strongly σ-donating C­(sp^3^) ligand through η^5^ → η^2^+η^3^ ring slippage plays a central role in reducing the activation barrier. This mechanistic insight provides a framework for rational ligand selection and will be valuable in guiding future developments in Rh-catalyzed transformations, particularly in evaluating when an indenyl ligand is likely to outperform its cyclopentadienyl counterpart.

## Methods

### General Procedure for the Enantioselective 1,2-Arylamination

In an oven-dried 4 mL reaction vial, with Teflon tape wrapped threads, and equipped with an oven-dried stir bar was brought into the glovebox. To the vial, CsOAc (0.01 mmol, 0.1 equiv), AgNTf_2_ (0.01 mmol, 0.1 equiv), and (***S,S***)-**5** (2.5 mol %) were added to the reaction vial. The vial was sealed with a Teflon septum screw cap and brought out of the box to complete the reaction. Under an N_2_ atmosphere outside of the glovebox, nitrogen source (***R***)-**8** was transferred to the reaction as stock solution in HFIP (0.5 mL, 0.13 mmol, 1.3 equiv). The olefin substrate (0.10 mmol 1.0 equiv) was added to the reaction vial using HFIP washing the vial three times (0.2 mL + 0.2 mL + 0.1 mL) to ensure complete transfer of the olefin. The reaction was left to stir at room temperature under an N_2_ balloon for 24 or 48 h. After completion, the crude reaction was filtered through a Celite pipet plug using DCM to flush. The solvent was removed under reduced pressure and the crude material purified via preparative TLC using the indicated eluent (see Supporting Information) to yield the corresponding 2-aminotetralin or spirocycle product.

## Supplementary Material





## Notes

Detailed experimental procedures, characterization data, spectra, and details of computational studies (PDF)
Cartesian coordinate file (XYZ)

## †

Department of Chemistry and Biochemistry, The Ohio State University, Columbus, Ohio 43210, United States

## §

Department of Chemistry, University of California, Irvine, Irvine, California 92697, United States
