# Supplementary material for: Enantioselective Olefin 1,2-Arylamination Catalyzed by a Planar Chiral Indenyl-Rhodium(III) Complex
Source: ACS Catal. 2026 Jan 16;16(4):3453–63. doi: 10.1021/acscatal.5c07589 (PMC12930352; doi:10.1021/acscatal.5c07589)

Supporting Information

Enantioselective Olefin 1,2-Arylamination Catalyzed by a Planar Chiral Indenyl-Rhodium(III) Complex

Patrick Gross,^{a, ||} Hyoju Choi,^{b,c, ||} Wesley A. Pullara,^{a, ||} Hoyoung Im,^{b,c} Khang Ung,^a Seunguk Kang,^{b,c} Mu-Hyun Baik,^{c,b,*} and Simon B. Blakey^{a,*}

^aDepartment of Chemistry, Emory University, Atlanta, Georgia 30322, USA

^bDepartment of Chemistry, Korea Advanced Institute of Science and Technology (KAIST),
Daejeon 34141, Republic of Korea

^cCenter for Catalytic Hydrocarbon Functionalizations, Institute for Basic Sciences (IBS),
Daejeon 34141, Republic of Korea

^{||} These authors contributed equally.

*email: sblakey@emory.edu

*email: mbaik2805@kaist.ac.kr

Table of contents

1. General Information.....	S3
2. Experimental Procedures and Tabulated Data	S4
3. General Procedure A – Nitrogen Source Synthesis.....	S5
4. General Procedure B – Grignard Addition to Benzyl Bromides	S6
5. General Procedure C – Grignard Addition to Benzaldehydes	S6
6. General Procedure D – Deoxygenation.....	S8
7. General Procedure E – Optimization of 1,2-Arylamination Procedure..	S11
8. Catalyst and Solvent Optimization (Table S1)	S12
9. Base and Silver Additive Optimization (Table S2)	S13
10. Nitrogen Source Optimization (Table S3)	S13
11. General Procedure F – Enantioselective 1,2-Arylamination Procedure	S14
12. Regioisomer Structural Assignments.....	S15
13. Arylamination Products.....	S15
14. Failed Substrates.....	S24
15. Synthetic Application	S24
16. Confirmation of Product Stereochemistry	S26
17. Synthesis of Mechanistic Substrates	S28
18. Mechanistic Studies.....	S30
19. Computational Details	S34
20. Supplementary Calculation.....	S35
20.1 Analysis of Enantioselective [2+2] Metallacyclization Steps	S35
20.2 Analysis of Ligand Effect: Indenyl vs. Cp*	S36
20.3 Effect of <i>N</i> -Protecting Group on Chemoselectivity.....	S38
20.4 Substituent Effects on Regioselectivity	S40
21. Energy Components for Optimized Structures	S44
22. Vibrational Frequencies of Optimized Structures.....	S47
23. Supplementary Information References	S66
24. NMR, HPLC, and SFC Data	S69

1. General Information

All reactions were conducted under nitrogen atmosphere with anhydrous solvents in oven- or flame-dried glassware using standard Schlenk technique, unless otherwise stated. Anhydrous dichloromethane (DCM), diethyl ether (Et₂O), tetrahydrofuran (THF), and were obtained by passage through activated alumina using a *Glass Contours* solvent purification system. 1,1,1,3,3,3-hexafluoroisopropanol (HFIP) was distilled over activated 4Å molecular sieves and stored over activated 4Å molecular sieves. Solvents for workup, extraction, and column chromatography were used as received from commercial suppliers without further purification. All catalysts were stored and weighed in a nitrogen-filled glovebox. All other chemicals were purchased from Millipore Sigma, Strem Chemicals, Oakwood Chemicals, Alfa Aesar, TCI, Combi Blocks, or Ambeed and used as received without further purification, unless otherwise stated.

¹H and ¹³C nuclear magnetic resonance (NMR) spectra were recorded on a Varian Inova 600 spectrometer (600 MHz ¹H, 151 MHz ¹³C), a Varian Inova 500 spectrometer (500 MHz ¹H, 126 MHz ¹³C), a Bruker 400 spectrometer (400 MHz ¹H, 126 MHz), a Varian Inova 400 spectrometer (400 MHz ¹H, 126 MHz ¹³C), and a. Chemical shifts δ values were reported in parts per million (ppm) relative to CHCl₃ (7.26 ppm for ¹H, 77.16 ppm for ¹³C) for CDCl₃, relative to C₆H₆ ((7.16 ppm for ¹H, 128.06 ppm for ¹³C) for C₆D₆, relative to DMSO (2.50 ppm for ¹H, 39.52 ppm for ¹³C) for DMSO-*d*₆. Coupling constants (*J* values) were reported in Hz and multiplicities were indicated using the following abbreviations: s = singlet, d = doublet, t = triplet, q = quartet, qn = quintet, m = multiplet, br = broad. High resolution mass spectra (HRMS) were obtained using a Thermo Electron Corporation Finigan LTQFTMS (at the Mass Spectrometry Facility, Emory University) or on an Agilent 5977C GC/MSD. High Pressure Liquid Chromatography (HPLC) was performed on an Agilent 1260 Infinity II series HPLC utilizing CHIRALPAK® IA, IB, IH, IJ, and IK 4.6 x 150 mm analytical columns. Semi preparative HPLC was performed on an Agilent 1260 Infinity II series preparative HPLC using a CHIRALCEL® OD-H 20 x 250 mm column. Supercritical Fluid Chromatography (SFC) was performed on a Waters Acquity UPC2 system using methanol/isopropanol with 0.2% formic acid in supercritical carbon dioxide utilizing CHIRALCEL® OJ3 3 μm particle size, 150 mm x 3 mm analytical columns. Optical rotations were measured on a PerkinElmer 341 polarimeter. Analytical thin layer chromatography (TLC) was performed on precoated glass-backed Silicycle SiliaPureR 0.25 mm silica gel 60 plates and visualized with UV light or ethanolic *p*-anisaldehyde. Silica gel column chromatography was performed using Silicycle SiliaFlashR F60 silica gel (40- 63 μm). Flash column chromatography was performed using Silicycle SiliaFlashR F60 silica gel (40- 63 μm) on a Biotage Isolera One system. Preparatory TLC was performed on precoated glass backed Silicycle SiliaPureR 1.0 mm silica gel 60 plates.

2. Experimental Procedures and Tabulated Data

Preparation of Starting Materials

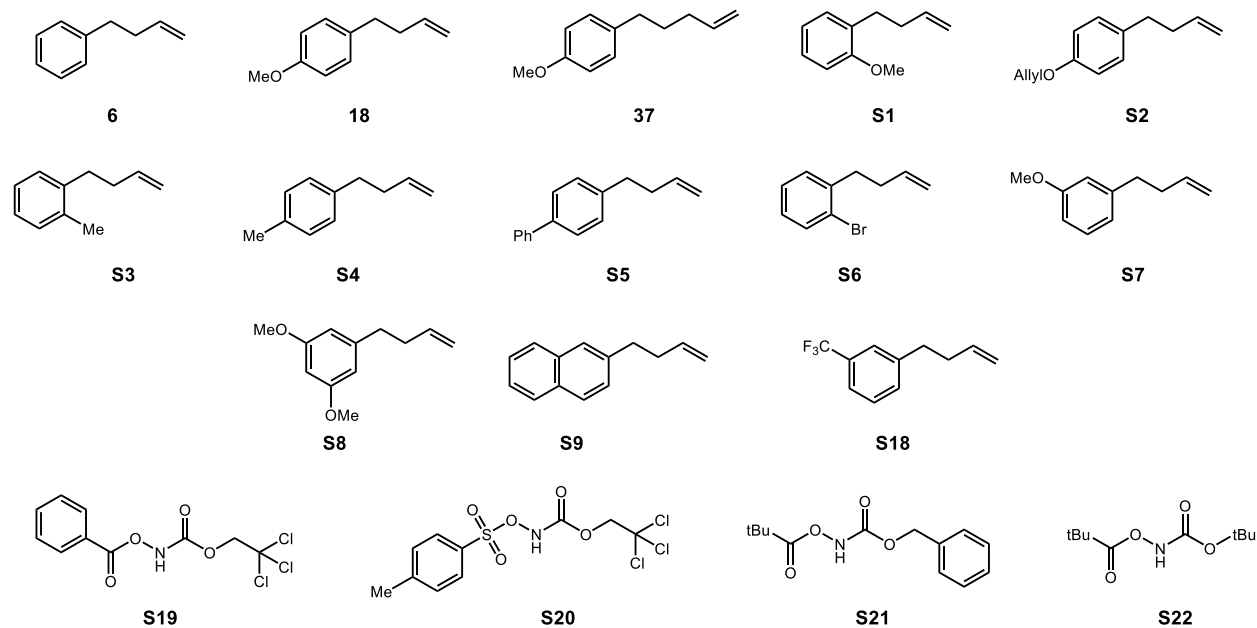

6 was obtained commercially and used without further purification.

S19,¹ **S20**,² **S21**,³ **S22**,⁴ **18**,⁵ **S2**,⁶ **S4**,⁵ **S5**,⁷ **S1**,⁸ **S6**,⁹ **S3**,¹⁰ **S7**,⁵ **S18**,⁷ **S8**,¹¹ **S9**,¹² and **37**¹³ were synthesized following reported literature procedures.

[Ind*RhCl₂]₂ catalyst was synthesized following a reported literature procedure.¹⁴

Catalysts **(S,S)-5**, **(S,S)-14**, **(S,S)-15**, **(S,S)-16**, and **(S,S)-17**¹⁵ were all synthesized using a method previously reported by us.¹⁶

3. General Procedure A: Nitrogen Source Synthesis

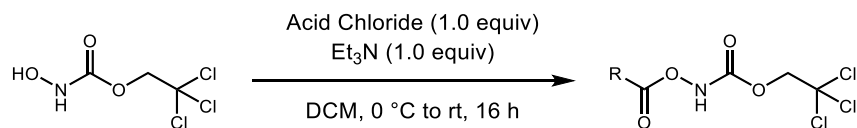

Under a N_2 atmosphere, Et_3N (1.0 equiv) was slowly added to a solution of 2,2,2-trichloroethyl hydroxycarbamate¹⁷ (1.0 equiv) in Et_2O (60mL) at 0 °C. Afterwards, acid chloride (1.0 equiv) was added dropwise leading to the formation of a white precipitate which was left to stir at room temperature overnight. The reaction was filtered through a Celite plug flushing with extra Et_2O . The solvent was removed under reduced pressure to provide the nitrogen source as an oil.

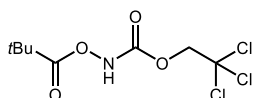

2,2,2-trichloroethyl (pivaloyloxy)carbamate (9): Prepared using General Procedure A, using 2,2,2-trichloroethyl hydroxycarbamate¹⁷ (8.0 g, 39 mmol) and pivaloyl chloride (4.7mL, 39 mmol) to provide **9** which was used without further purification (11.3 g, quant.) **¹H NMR** (400 MHz, $CDCl_3$) δ 8.22 (s, 1H), 4.80 (s, 2H), 1.32 (s, 9H). **¹³C NMR** (101 MHz, $CDCl_3$) δ 177.4, 154.9, 94.5, 75.3, 38.4, 27.1. **HRMS** (+APCI) calculated for $C_8H_{11}O_4NCl_3$ $[M-H]^-$ 289.9759, found 289.9767.

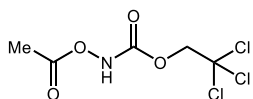

2,2,2-trichloroethyl acetoxycarbamate (S23): Prepared using General Procedure A, using 2,2,2-trichloroethyl hydroxycarbamate¹⁷ (2.1 g, 10 mmol) and acetyl chloride (0.7 mL, 10 mmol) to provide **S23** which was used without further purification (2.51 g, quant.) **¹H NMR** (400 MHz, $CDCl_3$) δ 8.41 (s, 1H), 4.80 (s, 2H), 2.24 (s, 3H). **¹³C NMR** (101 MHz, $CDCl_3$) δ 169.6, 154.7, 94.5, 75.2, 18.3. **HRMS** (+APCI) calculated for $C_5H_5O_4NCl_3$ $[M-H]^-$ 247.9290, found 247.9288.

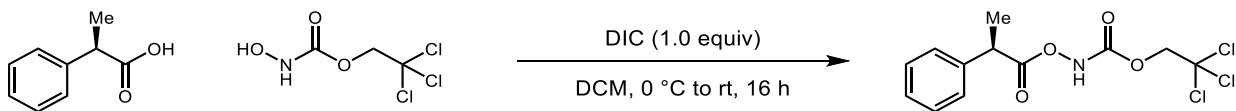

2,2,2-trichloroethyl (R)-((2-phenylpropanoyl)oxy)carbamate ((R)-8): Using an adapted procedure by Meggers.¹⁷ Under an N_2 atmosphere, in a 250 mL RBF equipped with a stir bar, 2,2,2-trichloroethyl hydroxycarbamate¹⁷ (4.17 g, 20.0 mmol, 1.0 equiv) was dissolved in DCM (124 mL) and the reaction vessel placed in an ice bath. Once cool, (R)-2-phenylpropanoic acid (2.7 mL, 20.0 mmol, 1.0 equiv) was added followed by the addition of a DIC (3.1 mL, 200 mmol 1.0 equiv). The reaction was stirred at room temperature overnight. Once complete, the solvent was removed under reduced pressure and the crude residue was immediately columned using flash column chromatography (5% EtOAc in Hexanes) to afford **(R)-8** as a slow crystallizing colorless solid. (4.84 g, 72%) **¹H NMR** (400 MHz, $CDCl_3$) δ 8.15 (s, 1H), 7.38 – 7.27 (m, 5H), 4.80 (d, J = 11.9 Hz, 1H), 4.76 (d, J = 11.9 Hz, 1H), 3.93 (q, J = 7.2 Hz, 1H), 1.61 (d, J = 7.2 Hz, 3H). **¹³C NMR**

(101 MHz, CDCl₃) δ 173.4, 154.6, 138.5, 129.0, 128.0, 127.7, 94.5, 75.3, 43.4, 18.6. Spectroscopic data for (*R*)-**8** matches those previously reported in the literature. ($\pm$)-**8** and (*S*)-**8** were synthesized in the same manner.¹⁷

4. General Procedure B: Allyl Magnesium Bromide Addition to Benzyl bromides

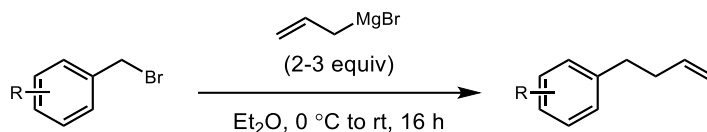

Under an N₂ atmosphere and at 0 °C, allyl magnesium bromide (1.0M in Et₂O, 2-3 equiv) was slowly added to a solution of the benzyl bromide substrate (1.0 equiv) in Et₂O (0.25M). Once the addition was completed the reaction was left to stir at room temperature overnight. The reaction was placed in an ice bath and slowly quenched with sat. NH₄Cl. The resulting biphasic solution was extracted with Et₂O (3x) and the combined organic layers washed with brine 1x. The organic layers were dried over MgSO₄ and the solvent removed under reduced pressure. The crude product was purified on silica gel column chromatography (100% Hexanes) to provide the final product.

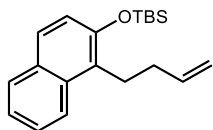

((1-(but-3-en-1-yl)naphthalen-2-yl)oxy)(tert-butyl)dimethylsilane (**S13**): Prepared using General Procedure B and ((1-(bromomethyl)naphthalen-2-yl)oxy)(tert-butyl)dimethylsilane¹⁸ (1.76g, 5.0 mmol). **S13** colorless oil (0.968 g, 62% yield). ¹H NMR (400 MHz, CDCl₃) δ 7.95 (dd, *J* = 8.6, 1.0 Hz, 1H), 7.78 (dd, *J* = 8.2, 1.5 Hz, 1H), 7.62 (d, *J* = 8.8 Hz, 1H), 7.48 (ddd, *J* = 8.4, 6.8, 1.4 Hz, 1H), 7.34 (ddd, *J* = 8.0, 6.7, 1.1 Hz, 1H), 7.10 (d, *J* = 8.8 Hz, 1H), 5.99 (ddt, *J* = 16.9, 10.2, 6.6 Hz, 1H), 5.12 (dq, *J* = 17.1, 1.7 Hz, 1H), 5.02 (ddd, *J* = 10.1, 2.1, 1.1 Hz, 1H), 3.20 – 3.10 (m, 2H), 2.43 – 2.32 (m, 2H), 1.08 (s, 9H), 0.28 (s, 6H). ¹³C NMR (101 MHz, CDCl₃) δ 150.6, 138.8, 133.4, 129.6, 128.6, 127.3, 126.2, 125.2, 123.4, 123.3, 120.4, 114.6, 34.2, 26.0, 25.3, 18.5, -3.8.

HRMS (+APCI) calculated for C₂₀H₂₈OSi [M+H]⁺ 313.1982, found 313.1980.

5. General Procedure C: Grignard Addition to Benzaldehydes

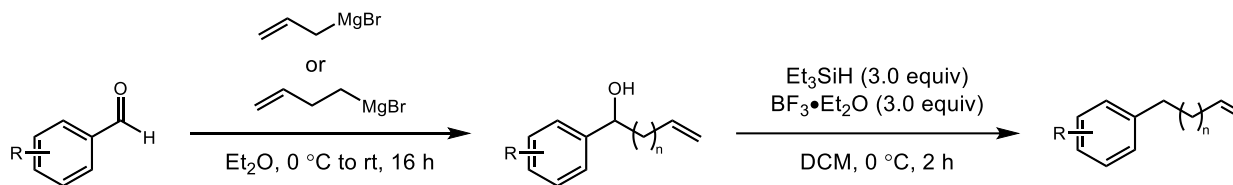

Under an N₂ atmosphere and at 0 °C, either allyl magnesium bromide or freshly prepared 3-butenylmagnesium bromide (1.0M in Et₂O, 2-3 equiv) was slowly added to a solution of the benzaldehyde substrate (1.0 equiv) in THF (0.25M). Once the addition was completed the reaction was left to stir at room temperature overnight. The reaction was placed in an ice bath and slowly quenched with sat. NH₄Cl. The resulting biphasic solution was extracted with Et₂O (3x) and the combined organic layers washed with brine (1x). The organic layers were dried over MgSO₄ and the solvent removed under reduced pressure. The crude product was purified on silica gel column chromatography (0-10% EtOAc in Hexanes) to provide the benzyl alcohol product.

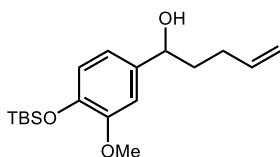

1-(4-((tert-butyldimethylsilyl)oxy)-3-methoxyphenyl)pent-4-en-1-ol (S24): Prepared using General Procedure C, 4-((tert-butyldimethylsilyl)oxy)-3-methoxybenzaldehyde¹⁹ (2.04g, 7.67 mmol) and 3-butenylmagnesium bromide (20 mL, 1.0M in Et₂O, 2.6 equiv). **S24** colorless oil (1.951 g, 79% yield). **¹H NMR** (400 MHz, CDCl₃) δ 6.86 (d, *J* = 2.0 Hz, 1H), 6.80 (d, *J* = 8.0 Hz, 1H), 6.76 (dd, *J* = 8.2, 2.0 Hz, 1H), 5.83 (ddt, *J* = 16.9, 10.2, 6.6 Hz, 1H), 5.03 (dq, *J* = 17.1, 1.7 Hz, 1H), 4.97 (ddt, *J* = 10.2, 2.2, 1.3 Hz, 1H), 4.61 (t, *J* = 6.7 Hz, 1H), 3.81 (s, 3H), 2.21 – 1.99 (m, 2H), 1.95 – 1.71 (m, 3H), 0.99 (s, 9H), 0.15 (s, 6H). **¹³C NMR** (101 MHz, CDCl₃) δ 151.0, 144.4, 138.3, 138.2, 120.6, 118.3, 114.9, 109.7, 74.0, 55.5, 38.0, 30.2, 25.7, 18.5, -4.6. **HRMS** (+APCI) calculated for C₁₈H₃₁O₃Si [M+H]⁺ 323.2037, found 323.2040

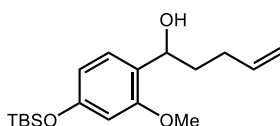

1-(4-((tert-butyldimethylsilyl)oxy)-2-methoxyphenyl)pent-4-en-1-ol (S25): Prepared using General Procedure C, 4-((tert-butyldimethylsilyl)oxy)-2-methoxybenzaldehyde²⁰ (2.04 g, 7.67 mmol), and 3-butenylmagnesium bromide (20 mL, 1.0M in Et₂O, 2.6 equiv). **S25** colorless oil (1.651 g, 67% yield). **¹H NMR** (400 MHz, CDCl₃) δ 7.10 (d, *J* = 8.2 Hz, 1H), 6.41 (dd, *J* = 8.1, 2.2 Hz, 1H), 6.38 (d, *J* = 2.2 Hz, 1H), 5.85 (ddt, *J* = 16.9, 10.2, 6.5 Hz, 1H), 5.04 (ddt, *J* = 17.2, 2.1, 1.6 Hz, 1H), 4.96 (ddt, *J* = 10.2, 2.2, 1.3 Hz, 1H), 4.80 (dt, *J* = 7.9, 5.9 Hz, 1H), 3.80 (s, 3H), 2.47 (d, *J* = 6.3 Hz, 1H), 2.27 – 2.15 (m, 1H), 2.15 – 2.04 (m, 1H), 1.98 – 1.75 (m, 2H), 0.98 (s, 9H), 0.20 (s, 6H). **¹³C NMR** (101 MHz, CDCl₃) δ 157.6, 156.0, 138.7, 127.5, 125.2, 114.6, 111.6, 103.6, 70.4, 55.3, 36.3, 30.4, 25.7, 18.2, -4.4. **HRMS** (+APCI) calculated for C₁₈H₃₁O₃Si [M+H]⁺ 323.2037, found 323.2042

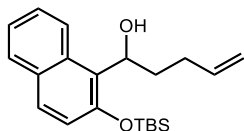

1-(2-((tert-butyldimethylsilyl)oxy)naphthalen-1-yl)pent-4-en-1-ol (S26): Prepared using General Procedure C, 2-((tert-butyldimethylsilyl)oxy)-1-naphthaldehyde²¹ (3.10 g, 10.8 mmol), and 3-butenylmagnesium bromide (22 mL, 1.0M in Et₂O, 2.0 equiv). **S26** colorless oil (2.16 g, 58% yield). **¹H NMR** (400 MHz, CDCl₃) δ 8.27 (d, *J* = 8.7 Hz, 1H), 7.77 (d, *J* = 7.9 Hz, 1H), 7.67 (d, *J* = 8.9 Hz, 1H), 7.47 (ddd, *J* = 8.5, 6.8, 1.5 Hz, 1H), 7.36 (ddd, *J* = 8.0, 6.8, 1.1 Hz, 1H), 7.09 (d, *J* = 8.9 Hz, 1H), 5.90 (ddt, *J* = 16.6, 10.1, 6.4 Hz, 1H), 5.61 (dd, *J* = 8.9, 5.1 Hz, 1H), 5.07 (dq, *J* = 17.0, 1.3 Hz, 1H), 4.99 (ddt, *J* = 10.1, 2.2, 1.1 Hz, 1H), 3.39 (s, 1H), 2.50 – 2.32 (m, 1H), 2.33 – 2.11 (m, 2H), 2.03 – 1.85 (m, 1H), 1.08 (s, 9H), 0.38 (s, 3H), 0.33 (s, 3H). **¹³C NMR** (101 MHz, CDCl₃) δ 150.6, 138.6, 132.3, 129.8, 129.0, 128.7, 126.4, 126.1, 124.0, 123.8, 120.1, 114.9, 69.4, 36.6, 30.8, 26.1, 18.5, -3.3, -3.8. **HRMS** (+APCI) calculated for C₂₁H₂₉O₂Si [M-H]⁻ 341.1942, found 341.1937

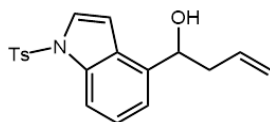

1-(1-tosyl-1H-indol-4-yl)but-3-en-1-ol (S27): Prepared using General Procedure C, 1-tosyl-1H-indole-4-carbaldehyde²² (1.61 g, 5.4 mmol), and allylmagnesium bromide (12 mL, 1.0M in Et₂O, 2.2 equiv). **S27** colorless oil (1.57 g, 86% yield). **¹H NMR** (400 MHz, CDCl₃) δ 7.91 (dt, *J* = 8.1, 1.0 Hz, 1H), 7.76 (d, *J* = 8.4 Hz, 2H), 7.58 (d, *J* = 3.7 Hz, 1H), 7.29 (t, *J* = 7.8 Hz, 1H), 7.25 – 7.18 (m, 3H), 6.83 (dd, *J* = 3.7, 0.8 Hz, 1H), 5.80 (ddt, *J* = 17.1, 10.1, 7.1 Hz, 1H), 5.19 – 5.09 (m, 2H), 5.02 (td, *J* = 6.6, 1.9 Hz, 1H), 2.56 (tt, *J* = 6.0, 1.4 Hz, 2H), 2.33 (s, 3H), 2.17 (d, *J* = 2.4 Hz, 1H). **¹³C NMR** (101 MHz, CDCl₃) δ 145.1, 136.6, 135.3, 135.1, 134.5, 130.0, 128.2, 127.0, 126.3, 124.7, 120.2, 118.6, 112.9, 107.4, 72.0, 43.0, 21.7. **HRMS** (+ACPI) calculated for C₁₉H₂₀O₃N³²S [M+H]⁺ 342.1158, found 342.1161

6. General Procedure D: Deoxygenation

Under an N₂ atmosphere, the benzyl alcohol (1.0 equiv) was dissolved in a DCM (0.1M) and placed in an ice bath to cool to 0 °C. Once cool, Et₃SiH (3.0 equiv) was added followed by the dropwise addition of BF₃•Et₂O (3.0 equiv). The reaction was left to stir at 0 °C and product formation was monitored by TLC. Once consumption of starting material was observed (~2 h) sat. NaHCO₃ was slowly added, and the reaction was stirred for an additional 20 min at 0 °C. The reaction was extracted with DCM (3X) and the combined organic washed with brine (1x). The organic layers were dried over Na₂SO₄ and solvent removed under reduced pressure. The crude product was purified on silica gel column chromatography (0-5% EtOAc in Hexanes) to provide the final product.

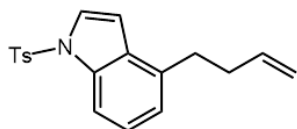

4-(but-3-en-1-yl)-1-tosyl-1H-indole (S10): Prepared using General Procedure D and **S27** (1.00 g, 2.92 mmol). **S10** colorless oil (.358 g, 38% yield). **¹H NMR** (400 MHz, CDCl₃) δ 7.84 (d, *J* = 8.4 Hz, 1H), 7.77 (d, *J* = 8.4 Hz, 2H), 7.57 (d, *J* = 3.7 Hz, 1H), 7.27 – 7.18 (m, 4H), 7.04 (dd, *J* = 7.4, 0.8 Hz, 1H), 6.70 (dd, *J* = 3.7, 0.9 Hz, 1H), 5.85 (ddt, *J* = 16.9, 10.2, 6.6 Hz, 1H), 5.04 (dq, *J* = 17.1, 1.6 Hz, 1H), 4.97 (ddt, *J* = 10.2, 2.2, 1.2 Hz, 1H), 2.95 – 2.82 (m, 2H), 2.40 (tdp, *J* = 7.9, 6.5, 1.3 Hz, 2H), 2.34 (s, 3H). **¹³C NMR** (101 MHz, CDCl₃) δ 145.0, 138.1, 135.5, 134.9, 134.9, 130.0, 130.0, 127.0, 125.9, 124.7, 122.8, 115.2, 111.4, 107.3, 34.8, 32.6, 21.7. **HRMS** (+APCI) calculated for C₁₉H₂₀O₂N³²S [M+H]⁺ 326.1209, found 326.1205

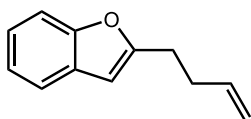

2-(but-3-en-1-yl)benzofuran (S11): Prepared using General Procedure D and 1-(benzofuran-2-yl)but-3-en-1-ol²³ (0.941 g, 5.00 mmol). **S11** colorless oil (0.179 g, 21% yield). Spectroscopic data for **S10** matches those previously reported in the literature.²⁴

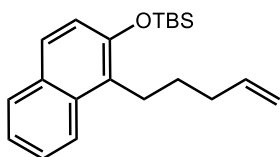

tert-butyl dimethyl((1-(pent-4-en-1-yl)naphthalen-2-yl)oxy)silane (S15): Prepared using General Procedure D and **S26** (2.163 g, 6.31 mmol). **S15** colorless oil (1.72 g, 66% yield). **¹H NMR** (400 MHz, CDCl₃) δ 7.94 (d, *J* = 8.6 Hz, 1H), 7.78 (d, *J* = 8.3 Hz, 1H), 7.61 (d, *J* = 8.8 Hz, 1H), 7.47 (ddd, *J* = 8.4, 6.7, 1.4 Hz, 1H), 7.33 (ddd, *J* = 8.0, 6.7, 1.1 Hz, 1H), 7.09 (d, *J* = 8.8 Hz, 1H), 5.91 (ddt, *J* = 16.9, 10.1, 6.7 Hz, 1H), 5.07 (dq, *J* = 17.1, 1.7 Hz, 1H), 4.99 (ddd, *J* = 10.2, 2.3, 1.1 Hz, 1H), 3.13 – 2.99 (m, 2H), 2.23 (q, *J* = 7.4 Hz, 2H), 1.79 – 1.63 (m, 2H), 1.08 (s, 9H), 0.28 (s, 6H). **¹³C NMR** (101 MHz, CDCl₃) δ 150.6, 139.0, 133.5, 129.6, 128.6, 127.2, 126.1, 125.8, 123.5, 123.3, 120.5, 114.8, 34.4, 29.4, 26.0, 25.4, 18.5, -3.7. **HRMS** (+APCI) calculated for C₂₁H₃₁OSi [M+H]⁺ 327.2139, found 327.2141

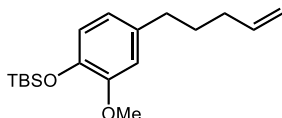

tert-butyl (2-methoxy-4-(pent-4-en-1-yl)phenoxy)dimethylsilane (S16): Prepared using General Procedure D and **S24** (0.847 g, 2.63 mmol). **S16** colorless oil (0.535 g, 66% yield). **¹H NMR** (400 MHz, CDCl₃) δ 6.76 (d, *J* = 8.0 Hz, 1H), 6.67 (d, *J* = 2.0 Hz, 1H), 6.62 (dd, *J* = 8.0, 2.1 Hz, 1H), 5.84 (ddt, *J* = 16.9, 10.2, 6.6 Hz, 1H), 5.09 – 4.93 (m, 2H), 3.79 (s, 3H), 2.60 – 2.49 (m, 2H), 2.14

– 2.03 (m, 2H), 1.76 – 1.64 (m, 2H), 1.00 (s, 9H), 0.15 (s, 5H). ^{13}C NMR (101 MHz, CDCl_3) δ 150.6, 142.9, 138.8, 136.0, 120.6, 120.5, 114.7, 112.5, 55.5, 35.0, 33.3, 30.8, 25.8, 18.4, –4.6.

HRMS (+APCI) calculated for $\text{C}_{18}\text{H}_{31}\text{O}_2\text{Si}$ $[\text{M}+\text{H}]^+$ 307.2088, found 307.2090

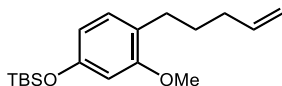

tert-butyl(3-methoxy-4-(pent-4-en-1-yl)phenoxy)dimethylsilane (**S17**): Prepared using General Procedure D and **S25** (0.837 g, 2.60 mmol). **S17** colorless oil (0.3474 g, 43% yield). ^1H NMR (400 MHz, CDCl_3) δ 6.93 (d, J = 8.7 Hz, 1H), 6.39 – 6.32 (m, 2H), 5.85 (ddt, J = 16.9, 10.2, 6.6 Hz, 1H), 5.02 (dq, J = 17.2, 1.8 Hz, 1H), 4.98 – 4.91 (m, 1H), 3.77 (s, 3H), 2.58 – 2.49 (m, 2H), 2.08 (td, J = 7.6, 5.9 Hz, 2H), 1.70 – 1.58 (m, 2H), 0.99 (s, 9H), 0.20 (s, 6H). ^{13}C NMR (101 MHz, CDCl_3) δ 158.3, 154.9, 139.3, 130.0, 123.7, 114.4, 111.3, 103.4, 55.4, 33.7, 29.3, 29.3, 25.9, 18.4, –4.2. **HRMS** (+APCI) calculated for $\text{C}_{18}\text{H}_{31}\text{O}_2\text{Si}$ $[\text{M}+\text{H}]^+$ 307.2088, found 307.2090

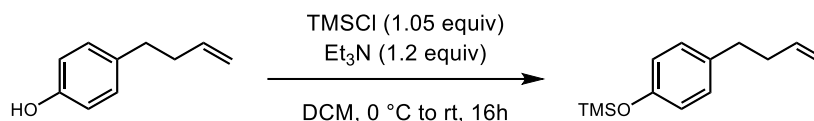

(4-(but-3-en-1-yl)phenoxy)trimethylsilane (**S12**): Under an N_2 atmosphere, in a 24 mL reaction vial equipped with a stir bar, 4-(but-3-en-1-yl)phenol (0.65 g, 4.4 mmol, 1.0 equiv) was dissolved in DCM (13.7 mL) and the reaction vial placed in an ice bath. Once cool, Et_3N (0.73 mL, 5.3 mmol, 1.2 equiv) was added followed by the addition of a TMSCl (0.54 mL, 4.6 mmol 1.2 equiv). The reaction was stirred at room temperature overnight. Once complete, the solvent was removed under reduced pressure and pentane was added to precipitate the amine chloride salt which was removed by filtration over Celite. Removal of the pentane filtrate provided an oil which was purified on silica gel column chromatography (0-5% EtOAc in Hexanes) to provide the final product **S12** as a clear oil (0.32 g, 33%). ^1H NMR (400 MHz, CDCl_3) δ 7.07 – 7.01 (m, 2H), 6.78 – 6.73 (m, 2H), 5.86 (ddt, J = 16.9, 10.2, 6.6 Hz, 1H), 5.03 (dq, J = 17.2, 1.6 Hz, 1H), 4.97 (ddt, J = 10.2, 2.2, 1.2 Hz, 1H), 2.64 (dd, J = 9.1, 6.6 Hz, 2H), 2.38 – 2.29 (m, 2H), 0.25 (d, J = 1.0 Hz, 9H). ^{13}C NMR (101 MHz, CDCl_3) δ 153.3, 138.4, 134.9, 129.4, 120.0, 114.9, 77.7, 35.9, 34.7, 0.4. **HRMS** (+APCI) calculated for $\text{C}_{13}\text{H}_{21}\text{O}^+\text{Si}$ $[\text{M}+\text{H}]^+$ 221.1356, found 221.1355.

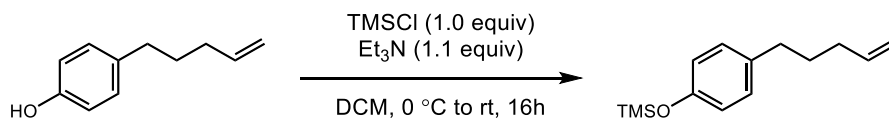

trimethyl(4-(pent-4-en-1-yl)phenoxy)silane (**S14**): Under an N_2 atmosphere, in a 24 mL reaction vial equipped with a stir bar, 4-(pent-4-en-1-yl)phenol (0.36 g, 2.2 mmol, 1.0 equiv) was dissolved in DCM (8 mL) and the reaction vial placed in an ice bath. Once cool, Et_3N (0.33 mL, 2.4 mmol, 1.1 equiv) was added followed by the addition of a TMSCl (0.29 mL, 2.2 mmol 1.0 equiv). The reaction was stirred at room temperature overnight. Once complete, the solvent was removed under reduced pressure and pentane was added to precipitate the amine chloride salt which was

removed by filtration over Celite. Removal of the pentane filtrate provided **S14** as an oil which did not require further purification (0.51 g, 97%). **¹H NMR** ¹H NMR (400 MHz, CDCl₃) δ 7.04 (d, *J* = 8.4 Hz, 2H), 6.76 (d, *J* = 8.5 Hz, 2H), 5.84 (ddt, *J* = 16.9, 10.2, 6.6 Hz, 1H), 5.02 (dq, *J* = 17.2, 1.6 Hz, 1H), 4.97 (ddt, *J* = 10.1, 2.3, 1.2 Hz, 1H), 2.56 (t, *J* = 7.6 Hz, 2H), 2.09 (qt, *J* = 7.8, 7.0, 1.3 Hz, 2H), 1.69 (p, *J* = 7.6 Hz, 2H), 0.26 (s, 9H). **¹³C NMR** (101 MHz, CDCl₃) δ 153.2, 138.9, 135.4, 129.4, 119.9, 114.8, 34.6, 33.4, 30.9, 0.4. **HRMS** (+APCI) calculated for C₁₄H₂₃OSi [M+H]⁺ 235.1513, found 235.1511.

7. General Procedure E: Optimization of Enantioselective 1,2-Arylation using 4-phenylbutene (**6**)

In an oven-dried 4 mL reaction vial, with Teflon tape wrapped threads, and equipped with an oven dried stir bar was brought into the glovebox. Base (10 mol%), silver salt additive (10 mol%), and catalyst (2.5 mol%) were added to the reaction vial. The vial was sealed with a Teflon septum screw cap and brought out of the box to complete the reaction. Under an N₂ atmosphere, HFIP (0.5 mL) was added directly to the reaction vial followed by the nitrogen source as a stock solution in HFIP (0.5 mL, 0.13 mmol, 1.3 equiv) and 4-phenylbutene **6** (15.0 μL, 0.10 mmol, 1.0 equiv.) using a micro syringe. The reaction was left to stir at room temperature under a N₂ balloon for 24 hours. After which, the crude reaction was filtered through a Celite pipette plug using DCM. The solvent was removed under reduced pressure and a crude NMR sample was prepared using dibromomethane as an NMR standard. The crude material purified via preparative TLC (5% EtOAc in hexanes) to yield the corresponding 2-aminotetralin product **10** which was analyzed via chiral HPLC (IJ column 5% 2-propanol in hexanes, 1.0 mL/min) to determine the enantiomeric ratio.

8. Catalyst and Solvent Optimization (Table S1)

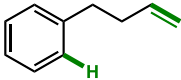 **6**<br>(1.0 equiv) **catalyst (2.5 mol%)**<br>**9 (1.3 equiv)** CsOAc (10 mol%)<br>AgNTf₂ (10 mol%)<br>**Solvent (0.1 M), 20 °C, 24 h** 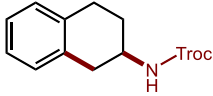 **(±) - 10**			
entry	catalyst	Solvent	% yield ^a
1	[Ind*RhCl ₂] ₂	HFIP	83
2	[Ind*RhCl ₂] ₂	TFE	37
3	[Ind*RhCl ₂] ₂	MeOH	0
4	[Ind*RhCl ₂] ₂	DCM	0
5	[Ind*RhCl ₂] ₂	THF	0
6	[Ind*RhCl ₂] ₂	Toluene	0
7	[Ind*RhCl ₂] ₂	DMF	0
8	Cp*Co(CO)I ₂ ^b	HFIP	0
9	[Cp*RhCl ₂] ₂	HFIP	0
10	[Cp*IrCl ₂] ₂	HFIP	0

^aDetermined by ¹H-NMR using dibromomethane as an internal standard

^bCp*Co(CO)I₂ (5 mol%)

9. Base and Silver Additive Optimization (Table S2)

$[Ind^*RhCl_2]_2$ (2.5 mol%)
9 (1.3 equiv)
base (10 mol%)
Ag Salt (10 mol%)
 HFIP (0.1 M), 20 °C, 24 h

entry	base	Ag Salt	% yield ^a
1	CsCO ₃	none	58
2	CsCO ₃	Ag ₂ CO ₃ ^b	50
3	CsCO ₃	AgSbF ₆	72
4	CsCO ₃	AgNTf ₂	77
5	CsOPiv	AgNTf ₂	74
6	CsOAc	AgNTf ₂	83
7	LiOAc	AgNTf ₂	76
8	NaOAc	AgNTf ₂	78
9	KOAc	AgNTf ₂	79
10	none	none	31
11	none	AgNTf ₂	78
12	CsOAc	none	51

^aDetermined by ¹H-NMR using 1,3,5-trimethoxybenzene as an internal standard

^bAg₂CO₃ (5 mol%)

10. Nitrogen Source Optimization (Table S3)

$[Ind^*RhCl_2]_2$ (2.5 mol%)
Nitrogen Source (1.3 equiv)
 CsOAc (10 mol%)
 AgNTf₂ (10 mol%)
 HFIP (0.1 M), 20 °C, 24 h

 **TrocNHOBz (S19)**<br>63%	 **TrocNHOTs (S20)**<br>0%	 **CbzNHOPiv (S21)**<br>56%
 **BocNHOPiv (S22)**<br>trace	 **TrocNHOOAc (S23)**<br>26%	

Yields determined by ¹H-NMR using dibromomethane as an internal standard

11. General Procedure F: Enantioselective 1,2-Arylation Procedure

In an oven-dried 4 mL reaction vial, with Teflon tape wrapped threads, and equipped with an oven dried stir bar was brought into the glovebox. To the vial, CsOAc (0.01 mmol, 0.1 equiv.), AgNTf₂ (0.01 mmol, 0.1 equiv.), and (**S,S**)-**5** (2.5 mol%) were added to the reaction vial. The vial was sealed with a Teflon septum screw cap and brought out of the box to complete the reaction. Under an N₂ atmosphere outside of the glovebox, nitrogen source (**R**)-**8** was transferred to the reaction as stock solution in HFIP (0.5 mL, 0.13 mmol, 1.3 equiv). The olefin substrate (0.10 mmol 1.0 equiv.) was added to the reaction vial using HFIP washing the vial three times (0.2 mL + 0.2 mL + 0.1 mL) to ensure complete transfer of the olefin. The reaction was left to stir at room temperature under an N₂ balloon for 24 or 48 hours. After completion, the crude reaction was filtered through a Celite pipette plug using DCM to flush. The solvent was removed under reduced pressure and the crude material purified via preparative TLC using the indicated eluent to yield the corresponding 2-aminotetralin or spirocycle product.

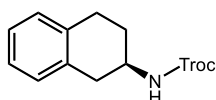

2,2,2-trichloroethyl (R)-(1,2,3,4-tetrahydronaphthalen-2-yl)carbamate (10): Prepared using **General Procedure E** (24 hours) using 4-phenylbutene **6**, (**R**)-**8**, and (**S,S**)-**5**. Purified via preparative TLC using (20% Acetone in Hexanes) to provide **10** (0.0277 g, 86% yield, 92:8 e.r.) as a colorless oil. ¹H NMR (400 MHz, CDCl₃) δ 7.19 – 7.04 (m, 4H), 5.06 (d, *J* = 7.4 Hz, 1H), 4.76 (d, *J* = 12.0 Hz, 2H), 4.72 (d, *J* = 12.0 Hz, 2H), 4.09 (qdd, *J* = 8.2, 5.1, 3.1 Hz, 1H), 3.18 (dd, *J* = 16.3, 5.2 Hz, 1H), 2.91 (t, *J* = 6.1 Hz, 2H), 2.72 (dd, *J* = 16.3, 8.1 Hz, 1H), 2.20 – 2.04 (m, 1H), 1.92 – 1.76 (m, 1H). ¹³C NMR (101 MHz, CDCl₃) δ 154.0, 135.4, 133.8, 129.6, 129.0, 126.4, 126.2, 95.7, 74.6, 47.3, 35.8, 28.8, 27.0. HRMS (+APCI) calculated for C₁₃H₁₅O₂NCl₃ [M+H]⁺ 322.0163, found 322.0168. HPLC (IJ column 5% 2-propanol in hexanes, 1.0 mL/min) *t*_M = 13.7 min *t*_m = 16.1 min, 92:8 e.r.

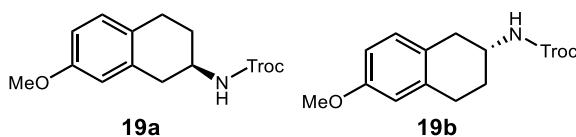

2,2,2-trichloroethyl (R)-(7-methoxy-1,2,3,4-tetrahydronaphthalen-2-yl)carbamate (19a) and **2,2,2-trichloroethyl (R)-(6-methoxy-1,2,3,4-tetrahydronaphthalen-2-yl)carbamate (19b):** Prepared using **General Procedure F** (48 hours) using **18**, (**R**)-**8**, and (**S,S**)-**5**. Purified via preparative TLC using (20% Acetone in Hexanes) to provide **19a** and **19b** as a colorless oil and mixture of inseparable regioisomers (0.0235 g, 65% yield, 1:4 r.r., 91:9 e.r.). ¹H NMR (800 MHz, CDCl₃) δ 7.02 (d, *J* = 8.4 Hz, 1H, minor regioisomer), 6.99 (d, *J* = 8.4 Hz, 1H), 6.72 (td, *J* = 8.9, 8.5, 2.7 Hz, 1H), 6.64 (d, *J* = 2.7 Hz, 1H), 6.61 (d, *J* = 2.7 Hz, 1H, minor regioisomer), 5.05 (d, *J* = 8.1 Hz, 1H), 4.75 (d, *J* = 12.1 Hz, 1H), 4.72 (d, *J* = 12.0 Hz, 1H), 4.10 – 4.01 (m, 1H), 3.78 (d, *J* = 4.0 Hz, 3H), 3.14 (dd, *J* = 16.4, 5.0 Hz, 1H, minor regioisomer), 3.10 (dd, *J* = 15.9, 5.1 Hz, 1H), 2.94 – 2.80 (m, 2H), 2.69 (dd, *J* = 16.3, 7.9 Hz, 1H, minor regioisomer), 2.64 (dd, *J* = 15.9, 8.0 Hz, 1H), 2.13 – 2.01 (m, 1H), 1.87 – 1.74 (m, 1H). ¹³C NMR (201 MHz, CDCl₃) δ 158.2, 158.0, 154.0, 136.5, 134.9, 130.5, 129.9, 127.4, 125.8, 114.0, 113.5, 112.9, 112.6, 95.8, 74.6, 55.4, 55.4, 47.5, 47.2, 36.1, 35.1, 29.0, 28.7, 27.3, 26.1. HRMS (+APCI) calculated for C₁₄H₁₇O₃NCl₃ [M+H]⁺ 352.0269,

found 352.0270. **SFC** (ChiralCel OJ-3 5% MeOH/IPA with 0.2% Formic Acid, 2.5 mL/min) (**19a**): $t_M = 6.05$ min $t_m = 4.43$ min, 92:8 e.r. (**19b**): $t_M = 4.84$ min $t_m = 3.82$ min, 91:9 e.r.

12. Structural Assignment of 2-aminotetralins via NOESY Correlations

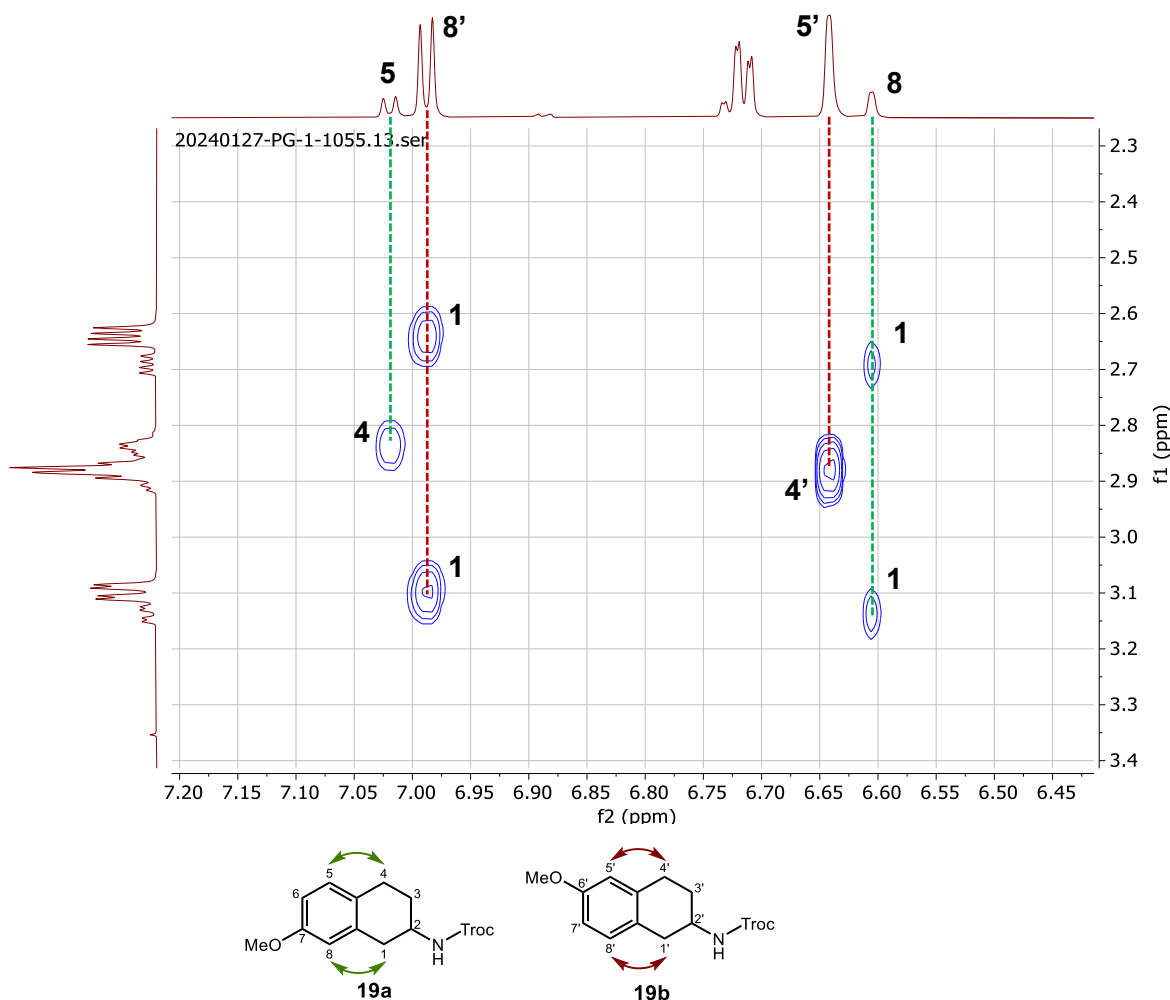

The identity of the major and minor regioisomers for all 2-aminotetralins was determined via the NOESY correlations between C1-C8 and between C4-C5.

13. Arylamination Products

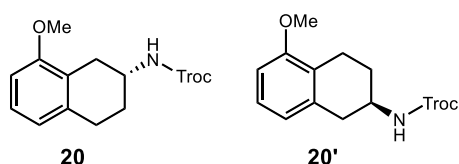

2,2,2-trichloroethyl (*R*)-(5-methoxy-1,2,3,4-tetrahydronaphthalen-2-yl)carbamate (**20'**) and 2,2,2-trichloroethyl (*R*)-(8-methoxy-1,2,3,4-tetrahydronaphthalen-2-yl)carbamate (**20**): Prepared using **General Procedure F** (48 hours) using **S1**, (*R*)-**8**, and (*S,S*)-**5**. Purified via preparative TLC using (15% Acetone in Hexanes) to provide **20** and **20'** as a colorless oil and mixture of inseparable

regioisomers (0.0203 g, 58% yield, 1:5 r.r., 86:13 e.r.). **¹H NMR** (600 MHz, CDCl₃) δ 7.12 (t, *J* = 7.9 Hz, 1H), 6.73 (d, *J* = 7.7 Hz, 1H), 6.72 – 6.65 (m, 1H), 5.04 (d, *J* = 7.9 Hz, 1H), 4.78 – 4.67 (m, 2H), 4.06 (ddt, *J* = 10.9, 8.3, 4.3 Hz, 1H), 3.82 (s, 3H, minor regioisomer), 3.81 (s, 3H), 3.18 – 3.08 (m, 1H), 2.95 – 2.66 (m, 2H), 2.52 (dd, *J* = 17.2, 8.0 Hz, 1H), 2.17 – 2.03 (m, 1H), 1.89 – 1.74 (m, 1H). **¹³C NMR** (151 MHz, CDCl₃) δ 157.6, 157.4, 154.1, 154.0, 136.8, 135.0, 126.8, 126.7, 124.4, 122.8, 121.69, 121.1, 107.6, 107.2, 95.8, 74.6, 55.4, 47.2, 46.9, 35.8, 29.9, 28.5, 28.2, 27.3, 21.0. **HRMS** (+APCI) calculated for C₁₄H₁₇O₃NCl₃ [M+H]⁺ 352.0269, found 352.0270. **HPLC** (IB column 10% 2-propanol in hexanes, 1.0 mL/min) (**20'**): *t*_M = 10.7 min *t*_m = 8.7 min, 88:12 e.r. (**20**): *t*_M = 9.38 min *t*_m = 11.8 min, 86:13 e.r.

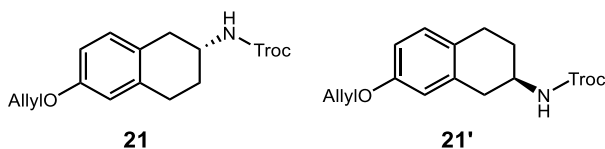

2,2,2-trichloroethyl (*R*)-(7-(allyloxy)-1,2,3,4-tetrahydronaphthalen-2-yl)carbamate (**21'**) and 2,2,2-trichloroethyl (*R*)-(6-(allyloxy)-1,2,3,4-tetrahydronaphthalen-2-yl)carbamate (**21**): Prepared using **General Procedure F** (24 hours) using **S2**, (*R*)-**8**, and (*S,S*)-**5**. Purified via preparative TLC using (20% Acetone in Hexanes) to provide **21** and **21'** as a colorless oil and mixture of inseparable regioisomers (0.0227 g, 54% yield, 1:4 r.r., 91:9 e.r.). **¹H NMR** (800 MHz, CDCl₃) δ 7.01 (d, *J* = 8.4 Hz, 1H, minor regioisomer), 6.98 (d, *J* = 8.4 Hz, 1H), 6.76 – 6.71 (m, 1H), 6.66 (d, *J* = 2.7 Hz, 1H), 6.62 (d, *J* = 2.7 Hz, 1H, minor regioisomer), 6.05 (ddtd, *J* = 18.8, 10.5, 5.3, 3.0 Hz, 1H), 5.40 (dp, *J* = 17.2, 1.5 Hz, 1H), 5.28 (dq, *J* = 10.5, 1.4 Hz, 1H), 5.03 (d, *J* = 8.0 Hz, 1H), 4.75 (d, *J* = 12.0 Hz, 1H), 4.72 (d, *J* = 12.0 Hz, 1H), 4.56 – 4.48 (m, 2H), 4.11 – 4.00 (m, 1H), 3.18 – 3.04 (m, 1H), 2.93 – 2.80 (m, 2H), 2.68 (dd, *J* = 16.2, 8.0 Hz, 1H, minor regioisomer), 2.64 (dd, *J* = 15.9, 8.0 Hz, 1H), 2.17 – 2.00 (m, 1H), 1.82 (dtd, *J* = 12.7, 8.7, 6.3 Hz, 1H). **¹³C NMR** (201 MHz, CDCl₃) δ 157.2, 157.0, 154.0, 136.5, 134.9, 133.5, 130.5, 129.9, 127.6, 126.0, 117.7, 115.1, 114.5, 113.6, 113.3, 95.8, 74.6, 69.0, 47.5, 47.2, 36.1, 35.1, 29.0, 28.7, 27.3, 26.2. **HRMS** (+APCI) calculated for C₁₈H₁₉O₃NCl₃ [M+H]⁺ 378.0425, found 378.0427. **SFC** (ChiralCel OJ-3 5% MeOH/IPA with 0.2% Formic Acid, 2.5 mL/min) (**21'**): *t*_M = 8.70 min *t*_m = 5.44 min, 92:8 e.r. (**21**): *t*_M = 6.02 min *t*_m = 5.05 min, 91:9 e.r.

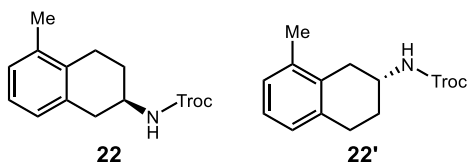

2,2,2-trichloroethyl (*R*)-(5-methyl-1,2,3,4-tetrahydronaphthalen-2-yl)carbamate (**22**) and 2,2,2-trichloroethyl (*R*)-(8-methyl-1,2,3,4-tetrahydronaphthalen-2-yl)carbamate (**22'**): Prepared using **General Procedure F** (48 hours) using **S3**, (*R*)-**8**, and (*S,S*)-**5**. Purified via preparative TLC using (20% Acetone in Hexanes) to provide **22** and **22'** as a colorless oil and mixture of inseparable regioisomers (0.0178 g, 60% yield, 3:1 r.r., 90:10 e.r.). **¹H NMR** (800 MHz, CDCl₃) δ 7.09 – 7.05 (m, 1H), 7.05 – 7.00 (m, 1H), 6.98 (d, *J* = 7.4 Hz, 1H, minor regioisomer), 6.94 (d, *J* = 7.4 Hz, 1H), 5.08 (d, *J* = 8.0 Hz, 1H, minor regioisomer), 5.04 (d, *J* = 8.0 Hz, 1H), 4.79 – 4.69 (m, 2H), 4.14 – 4.02 (m, 1H), 3.16 (dd, *J* = 16.0, 4.9 Hz, 1H), 3.08 (dd, *J* = 16.6, 5.5 Hz, 1H, minor regioisomer),

2.96 – 2.87 (m, 1H), 2.82 – 2.70 (m, 2H), 2.51 (dd, J = 16.6, 8.2 Hz, 1H, minor regioisomer), 2.24 (s, 2H), 2.22 (s, 1H), 2.18 – 2.07 (m, 1H), 1.93 – 1.84 (m, 1H), 1.84 – 1.76 (m, 1H, minor regioisomer). ^{13}C NMR (201 MHz, CDCl_3) δ 154.1, 137.0, 136.7, 135.4, 133.9, 133.6, 132.5, 127.98, 127.7, 127.5, 126.8, 126.1, 126.0, 95.8, 74.6, 74.6, 47.7, 46.9, 36.3, 33.5, 28.8, 28.6, 27.7, 24.6, 19.7. **HRMS** (+APCI) calculated for $\text{C}_{14}\text{H}_{17}\text{O}_2\text{NCl}_3$ $[\text{M}+\text{H}]^+$ 336.0319, found 336.0321. **SFC** (ChiralCel OJ-3 1% MeOH/IPA with 0.2% Formic Acid, 2.5 mL/min) (**22**): t_{M} = 4.64 min t_{m} = 7.03 min, 90:10 e.r. (**22'**): inseparable

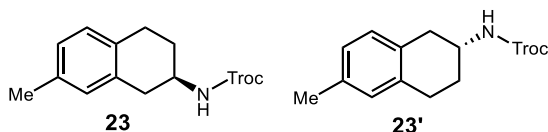

2,2,2-trichloroethyl (*R*)-(7-methyl-1,2,3,4-tetrahydronaphthalen-2-yl)carbamate (**23**) and 2,2,2-trichloroethyl (*R*)-(6-methyl-1,2,3,4-tetrahydronaphthalen-2-yl)carbamate (**23'**): Prepared using **General Procedure F** (48 hours) using **S4**, (*R*)-**8**, and (*S,S*)-**5**. Purified via preparative TLC using (15% Acetone in Hexanes) to provide **23** and **23'** as a colorless oil and mixture of inseparable regioisomers (0.0123 g, 37% yield, 2:1 r.r., 92:8 e.r.). ^1H NMR (800 MHz, CDCl_3) δ 7.00 (d, J = 7.7 Hz, 1H), 6.99 – 6.94 (m, 1H), 6.93 (s, 1H, minor regioisomer), 6.90 (s, 1H), 5.03 (d, J = 8.0 Hz, 1H), 4.75 (d, J = 12.0 Hz, 1H), 4.72 (d, J = 12.0 Hz, 1H), 4.12 – 4.02 (m, 1H), 3.13 (dd, J = 16.2, 5.2 Hz, 1H), 2.91 – 2.82 (m, 2H), 2.68 (dd, J = 16.2, 7.8 Hz, 1H), 2.29 (s, 3H), 2.14 – 2.05 (m, 1H), 1.83 (dq, J = 14.6, 8.6, 8.2 Hz, 1H). ^{13}C NMR (201 MHz, CDCl_3) δ 154.0, 136.0, 135.7, 135.2, 133.6, 132.3, 130.6, 130.1, 129.6, 129.5, 128.9, 127.3, 127.1, 95.8, 74.6, 47.4, 47.3, 35.8, 35.5, 28.9, 28.8, 26.9, 26.6, 21.1, 21.1. **HRMS** (+APCI) calculated for $\text{C}_{14}\text{H}_{17}\text{O}_2\text{NCl}_3$ $[\text{M}+\text{H}]^+$ 336.0319, found 336.0321. **SFC** (ChiralCel OJ-3 5% MeOH/IPA with 0.2% Formic Acid, 2.5 mL/min) (**23**): t_{M} = 7.77 min t_{m} = 4.42 min, 92:8 e.r. (**23'**): t_{M} = 5.48 min t_{m} = 4.08 min, 87:13 e.r.

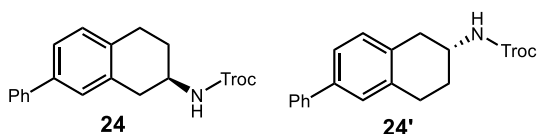

2,2,2-trichloroethyl (*R*)-(7-phenyl-1,2,3,4-tetrahydronaphthalen-2-yl)carbamate (**24**) and 2,2,2-trichloroethyl (*R*)-(6-phenyl-1,2,3,4-tetrahydronaphthalen-2-yl)carbamate (**24'**): Prepared using **General Procedure F** (24 hours) using **S5**, (*R*)-**8**, and (*S,S*)-**5**. Purified via preparative TLC using (20% Acetone in Hexanes) to provide **24** and **24'** as a colorless oil and mixture of inseparable regioisomers (0.0238 g, 57% yield, 3:1 r.r., 93:7 e.r.). ^1H NMR (800 MHz, CDCl_3) δ 7.57 (d, J = 7.4 Hz, 2H), 7.43 (t, J = 7.6 Hz, 2H), 7.41 – 7.36 (m, 1H), 7.34 (t, J = 7.2 Hz, 1H), 7.31 (s, 1H), 7.19 (d, J = 7.9 Hz, 1H), 7.16 (d, J = 7.9 Hz, 1H, minor regioisomer), 5.08 (d, J = 6.9 Hz, 1H), 4.77 (d, J = 12.1 Hz, 1H), 4.74 (d, J = 12.0 Hz, 1H), 4.13 (dp, J = 12.6, 4.3 Hz, 1H), 3.28 – 3.18 (m, 1H), 3.01 – 2.90 (m, 2H), 2.83 – 2.72 (m, 1H), 2.21 – 2.10 (m, 1H), 1.89 (dq, J = 14.9, 7.9 Hz, 1H). ^{13}C NMR (201 MHz, CDCl_3) δ 154.3, 154.0, 141.0, 141.0, 139.6, 139.3, 135.8, 134.6, 134.2, 132.9, 130.1, 129.5, 128.9, 128.2, 127.7, 127.3, 127.3, 127.1, 127.1, 125.4, 125.1, 95.8, 74.9, 74.6, 48.0, 47.4, 47.3, 36.0, 35.6, 29.5, 28.9, 28.8, 27.2, 26.8. **HRMS** (+APCI) calculated for $\text{C}_{19}\text{H}_{19}\text{O}_2\text{NCl}_3$ $[\text{M}+\text{H}]^+$ 398.0476, found 398.0480. **HPLC** (IJ column 20% 2-propanol in

hexanes, 1.0 mL/min) (**24**): $t_M = 24.9$ min $t_m = 37.0$ min, 93:7 e.r. (**24'**): $t_M = 20.0$ min $t_m = 17.9$ min, 89:11 e.r.

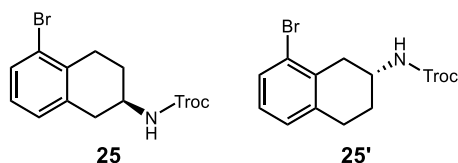

2,2,2-trichloroethyl (*R*)-(5-bromo-1,2,3,4-tetrahydronaphthalen-2-yl)carbamate (**25**) and 2,2,2-trichloroethyl (*R*)-(8-bromo-1,2,3,4-tetrahydronaphthalen-2-yl)carbamate (**25'**): Prepared using **General Procedure F** (48 hours) using **S6**, (*R*)-**8**, and (*S,S*)-**5**. Purified via preparative TLC using (15% Acetone in Hexanes) to provide **25** and **25'** as a colorless oil and mixture of inseparable regioisomers (0.0100 g, 25% yield, 5:1 r.r., 88:11 e.r.). **¹H NMR** (800 MHz, CDCl₃) δ 7.44 – 7.39 (m, 1H), 7.07 (d, $J = 7.5$ Hz, 1H, minor regioisomer), 7.05 – 6.98 (m, 2H), 5.01 (d, $J = 7.9$ Hz, 1H), 4.73 (s, 2H), 4.13 – 3.99 (m, 1H), 3.22 (dd, $J = 17.1, 5.6$ Hz, 1H, minor regioisomer), 3.17 (dd, $J = 16.3, 4.9$ Hz, 1H), 2.98 – 2.89 (m, 1H), 2.88 – 2.80 (m, 1H), 2.75 (dd, $J = 16.3, 8.0$ Hz, 1H), 2.62 (dd, $J = 17.1, 8.3$ Hz, 1H, minor regioisomer), 2.21 – 2.06 (m, 1H), 1.88 (td, $J = 14.6, 8.5$ Hz, 1H). **¹³C NMR** (201 MHz, CDCl₃) δ 154.0, 136.2, 135.0, 130.8, 130.4, 128.8, 128.2, 127.6, 127.5, 125.9, 125.7, 95.7, 74.7, 74.7, 47.7, 46.8, 36.7, 36.3, 29.9, 28.8, 28.2, 28.0. **HRMS** (+APCI) calculated for C₁₄H₁₇O₃NCl₃ [M+H]⁺ 352.0269, found 352.0270. **SFC** (ChiralCel OJ-3 5% MeOH/IPA with 0.2% Formic Acid, 2.5 mL/min) (**25**): $t_M = 16.9$ min $t_m = 11.6$ min, 88:11 e.r. (**25'**): inseparable

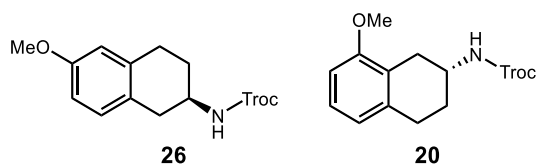

2,2,2-trichloroethyl (*R*)-(6-methoxy-1,2,3,4-tetrahydronaphthalen-2-yl)carbamate (**26**) and 2,2,2-trichloroethyl (*R*)-(8-methoxy-1,2,3,4-tetrahydronaphthalen-2-yl)carbamate (**20**): Prepared using **General Procedure F** (24 hours) using **S7**, (*R*)-**8**, and (*S,S*)-**5**. Purified via preparative TLC using (20% Acetone in Hexanes) to provide **26** and **20** as a colorless oil and mixture of inseparable regioisomers (0.0297 g, 80% yield, 3:1 r.r., 91:9 e.r.). **¹H NMR** (800 MHz, CDCl₃) δ 7.12 (t, $J = 7.9$ Hz, 1H, minor regioisomer), 6.99 (d, $J = 8.4$ Hz, 1H), 6.75 – 6.69 (m, 1H), 6.68 (d, $J = 8.1$ Hz, 1H, minor regioisomer), 6.64 (d, $J = 2.7$ Hz, 1H), 5.05 (d, $J = 7.6$ Hz, 1H), 4.76 – 4.73 (m, 1H), 4.72 (d, $J = 12.1$ Hz, 1H), 4.06 (tq, $J = 8.3, 4.2, 3.3$ Hz, 1H), 3.81 (s, 3H, minor regioisomer), 3.78 (s, 3H), 3.16 – 3.06 (m, 1H), 2.94 – 2.83 (m, 2H), 2.64 (dd, $J = 15.9, 8.0$ Hz, 1H), 2.52 (dd, $J = 17.1, 8.1$ Hz, 1H, minor regioisomer), 2.14 – 2.01 (m, 1H), 1.89 – 1.75 (m, 1H). **¹³C NMR** (201 MHz, CDCl₃) δ 158.2, 157.6, 154.0, 136.8, 136.5, 130.5, 126.7, 125.8, 122.8, 121.0, 113.5, 112.6, 107.2, 95.8, 74.6, 55.4, 47.5, 47.2, 35.5, 35.1, 29.9, 28.7, 28.5, 27.3, 27.3. **HRMS** (+APCI) calculated for C₁₄H₁₇O₃NCl₃ [M+H]⁺ 352.0269, found 352.0270. **HPLC** (IA column 5% 2-propanol in hexanes, 1.0 mL/min) (**26**): $t_M = 10.8$ min $t_m = 11.7$ min, 91:9 e.r. (**20**): $t_M = 9.1$ min $t_m = 17.5$ min, 92:8 e.r.

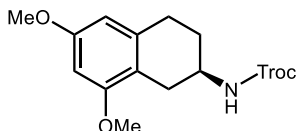

2,2,2-trichloroethyl (R)-(6,8-dimethoxy-1,2,3,4-tetrahydronaphthalen-2-yl)carbamate (27): Prepared using **General Procedure F** (24 hours) using **S8**, (**R**)-**8**, and (**S,S**)-**5**. Purified via preparative TLC using (20% Et₂O in Pentane) to provide **27** (0.0120 g, 31% yield, 87:13 e.r.) as a colorless oil. ¹H NMR (400 MHz, CDCl₃) δ 6.29 (d, *J* = 2.4 Hz, 1H), 6.24 (d, *J* = 2.4 Hz, 1H), 5.03 (d, *J* = 7.9 Hz, 1H), 4.75 (d, *J* = 11.9 Hz, 2H), 4.71 (d, *J* = 12.0 Hz, 2H), 4.03 (dddd, *J* = 16.7, 8.3, 5.7, 2.9 Hz, 1H), 3.78 (s, 6H), 3.03 (dd, *J* = 16.8, 5.5 Hz, 1H), 2.85 (td, *J* = 6.7, 3.0 Hz, 2H), 2.44 (dd, *J* = 16.8, 7.9 Hz, 1H), 2.15 – 1.98 (m, 1H), 1.88 – 1.72 (m, 1H). ¹³C NMR (201 MHz, CDCl₃) δ 159.0, 158.6, 154.0, 137.3, 115.1, 104.1, 96.3, 95.8, 74.6, 55.5, 55.4, 47.3, 29.5, 28.5, 27.6. **HRMS** (+APCI) calculated for C₁₅H₁₉O₄NC₃ [M+H]⁺ 382.0374, found 382.0379. **HPLC** (IA column 10% 2-propanol in hexanes, 1.0 mL/min) *t*_M = 8.5 min *t*_m = 13.0 min, 87:13 e.r.

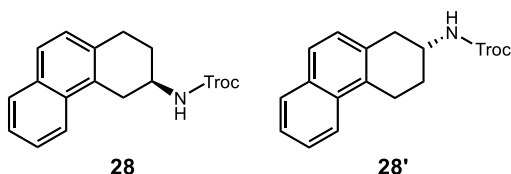

2,2,2-trichloroethyl (R)-(1,2,3,4-tetrahydrophenanthren-3-yl)carbamate (28) and **2,2,2-trichloroethyl (R)-(1,2,3,4-tetrahydrophenanthren-2-yl)carbamate (28')**: Prepared using **General Procedure F** (24 hours) using **S9**, (**R**)-**8**, and (**S,S**)-**5**. Purified via preparative TLC using (20% Acetone in Hexanes) to provide **28** and **28'** as an off white solid and mixture of inseparable regioisomers (0.0309 g, 80% yield, 8:1 r.r., 91:9 e.r.). ¹H NMR (400 MHz, CDCl₃) δ 7.96 (d, *J* = 8.4 Hz, 1H, minor regioisomer), 7.91 (d, *J* = 8.4 Hz, 1H), 7.82 (dd, *J* = 8.0, 1.5 Hz, 1H), 7.67 (d, *J* = 8.4 Hz, 1H), 7.52 (ddd, *J* = 8.4, 6.7, 1.6 Hz, 1H), 7.47 (ddd, *J* = 8.0, 6.7, 1.3 Hz, 1H), 7.23 (d, *J* = 8.4 Hz, 1H), 7.18 (d, *J* = 8.4 Hz, 1H, minor regioisomer), 5.17 (d, *J* = 8.0 Hz, 1H), 4.79 (d, *J* = 12.0 Hz, 1H), 4.74 (d, *J* = 12.0 Hz, 1H), 4.32 – 4.07 (m, 1H), 3.57 (dd, *J* = 16.6, 5.5 Hz, 1H), 3.15 – 2.93 (m, 3H), 2.87 (dd, *J* = 16.5, 7.7 Hz, 1H, minor regioisomer), 2.30 – 2.12 (m, 1H), 2.07 – 1.84 (m, 1H). ¹³C NMR (201 MHz, CDCl₃) δ 154.1, 132.9, 132.4, 132.3, 128.7, 128.4, 127.7, 126.7, 126.4, 125.4, 125.3, 123.0, 122.7, 95.8, 74.7, 74.6, 47.5, 46.9, 32.5, 28.5, 28.1. **HRMS** (+APCI) calculated for C₁₇H₁₇O₂NC₃ [M+H]⁺ 372.0319, found 372.0322. **SFC** (ChiralCel OJ-3 5% MeOH/IPA with 0.2% Formic Acid, 2.5 mL/min) (**28**): *t*_M = 8.77 min *t*_m = 10.35 min, 91:9 e.r. (**28'**): *t*_M = 5.22 min *t*_m = 7.76 min, 95:5 e.r.

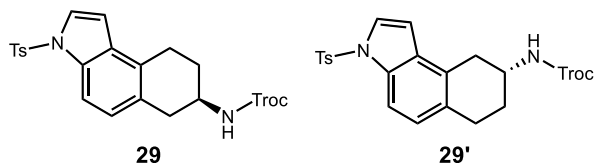

2,2,2-trichloroethyl (R)-(3-tosyl-6,7,8,9-tetrahydro-3H-benzo[e]indol-7-yl)carbamate (29) and **2,2,2-trichloroethyl (R)-(3-tosyl-6,7,8,9-tetrahydro-3H-benzo[e]indol-8-yl)carbamate (29')**: Prepared using **General Procedure F** (24 hours) using **S10**, (**R**)-**8**, and (**S,S**)-**5**. Purified via

preparative TLC using (20% Acetone in Hexanes) to provide **29** and **29'** as a colorless oil and mixture of regioisomers (0.0502 g, 92% yield, 88:12 e.r.). **¹H NMR** (800 MHz, CDCl₃) δ 7.76 (m, 3H), 7.61 – 7.49 (m, 1H), 7.21 (d, *J* = 8.1 Hz, 2H), 7.04 (d, *J* = 8.5 Hz, 1H, minor regioisomer), 7.01 (d, *J* = 8.6 Hz, 1H, major regioisomer), 6.62 (d, *J* = 3.7 Hz, 1H, major regioisomer), 6.59 (d, *J* = 3.7 Hz, 1H, minor regioisomer), 5.09 (m, 1H), 4.86 – 4.68 (m, 2H), 4.10 (m, 1H), 3.28 (dd, *J* = 16.6, 5.3 Hz, 1H, minor regioisomer), 3.19 (dd, *J* = 16.1, 4.8 Hz, 1H, major regioisomer), 2.97 (m, 2H), 2.75 (m, *J* = 16.0, 7.9 Hz, 1H), 2.33 (s, 3H), 2.13 (m, 1H), 1.90 (m, 1H). **¹³C NMR** (201 MHz, CDCl₃) δ 154.0, 145.0, 135.5, 133.2, 133.0, 130.3, 130.0, 129.9, 128.1, 127.8, 126.9, 126.4, 126.2, 126.2, 125.8, 111.8, 111.7, 106.9, 106.8, 95.7, 74.6, 74.6, 47.2, 47.0, 35.5, 32.8, 28.8, 28.1, 26.8, 24.0, 21.7. **HRMS** (+APCI) calculated for C₂₂H₂₂O₄N₂³⁵Cl₃³²S [M+H]⁺ 515.0360, found 515.0370 **HPLC** (IA column 20% 2-propanol in hexanes, 1.0 mL/min) (**29**): *t*_M = 14.0 min *t*_m = 13.0 min, 88:12 e.r. (**29'**): inseparable

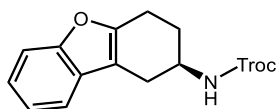

2,2,2-trichloroethyl (R)-(1,2,3,4-tetrahydrodibenzo[b,d]furan-2-yl)carbamate (30): Prepared using **General Procedure F** (48 hours) using **S11**, (**R**)-**8**, and (**S,S**)-**5**. Purified via preparative TLC using (20% Acetone in Hexanes) to provide **30** (0.0154 g, 41% yield, 92:8 e.r.) as a colorless oil. **¹H NMR** (800 MHz, CDCl₃) δ 7.42 (d, *J* = 8.0 Hz, 1H), 7.39 (d, *J* = 7.5 Hz, 1H), 7.24 (t, *J* = 7.5 Hz, 1H), 7.21 (d, *J* = 14.4 Hz, 1H), 5.12 (d, *J* = 8.2 Hz, 1H), 4.76 (d, *J* = 12.1 Hz, 1H), 4.73 (d, *J* = 12.0 Hz, 1H), 4.25 – 4.16 (m, 1H), 3.09 (dd, *J* = 15.6, 5.1 Hz, 1H), 2.94 – 2.81 (m, 2H), 2.65 – 2.58 (m, 1H), 2.21 – 2.14 (m, 1H), 2.09 (dq, *J* = 13.5, 7.0 Hz, 1H). **¹³C NMR** (201 MHz, CDCl₃) δ 155.0, 154.1, 152.5, 128.4, 123.8, 122.6, 118.5, 111.2, 110.5, 95.7, 74.7, 46.9, 28.2, 27.5, 21.0. **HRMS** (+APCI) calculated for C₁₅H₁₅O₃N³⁵Cl₃ [M+H]⁺ 362.0112, found 362.0116. **HPLC** (IH column 5% 2-propanol in hexanes, 1.0 mL/min) *t*_M = 17.9 min *t*_m = 16.6 min, 92:8 e.r.

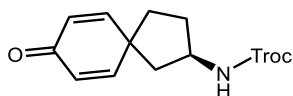

2,2,2-trichloroethyl (R)-(8-oxospiro[4.5]deca-6,9-dien-2-yl)carbamate (31): Prepared using **General Procedure F** using **S12**, (**R**)-**8**, and (**S,S**)-**5**. Purified via preparative TLC using (20% Acetone in Hexanes) to provide **31** (0.0089 g, 26% yield, 89:11 e.r.) as a colorless oil. **¹H NMR** (800 MHz, CDCl₃) δ 6.88 (t, *J* = 8.3 Hz, 2H), 6.22 (dd, *J* = 9.7, 8.0 Hz, 2H), 5.15 (d, *J* = 17.1 Hz, 1H), 4.73 (s, 2H), 4.36 (h, *J* = 7.4 Hz, 1H), 2.40 (q, *J* = 11.9, 9.5 Hz, 1H), 2.27 (dd, *J* = 13.8, 7.6 Hz, 1H), 2.04 – 1.95 (m, 1H), 1.86 (td, *J* = 12.8, 7.8 Hz, 2H), 1.80 (dd, *J* = 13.8, 7.8 Hz, 1H). **¹³C NMR** (201 MHz, CDCl₃) δ 185.9, 154.3, 154.1, 153.2, 127.8, 127.6, 95.6, 74.7, 52.8, 47.1, 43.8, 35.9, 32.6. **HRMS** (+APCI) calculated for C₁₃H₁₅O₃NCl₃ [M+H]⁺ 338.0112, found 338.0118. **HPLC** (IA column 10% 2-propanol in hexanes, 1.0 mL/min) *t*_M = 15.4 min *t*_m = 19.8 min, 89:11 e.r.

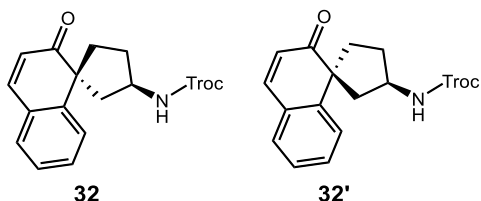

2,2,2-trichloroethyl ((1*R*,3*R*)-2'-oxo-2'*H*-spiro[cyclopentane-1,1'-naphthalen]-3-yl)carbamate (**32**) and 2,2,2-trichloroethyl ((1*S*,3*R*)-2'-oxo-2'*H*-spiro[cyclopentane-1,1'-naphthalen]-3-yl)carbamate (**32'**): Prepared using **General Procedure F** using **S13**, (*R*)-**8**, and (*S,S*)-**5**. Purified via preparative TLC using (20% EtOAc in Hexanes) to provide **32** and **32'** separable diastereomers (0.0293g, 73% yield, 1.9:1 d.r.).

32: Colorless oil (0.0187 g, 46% yield, 89:11 e.r.). **¹H NMR** (800 MHz, CDCl₃) δ 7.48 (d, *J* = 7.8 Hz, 1H), 7.46 – 7.39 (m, 2H), 7.33 – 7.26 (m, 2H), 6.15 (d, *J* = 9.7 Hz, 1H), 5.13 (s, 1H), 4.78 (d, *J* = 11.8 Hz, 1H), 4.70 (d, *J* = 12.1 Hz, 1H), 4.43 (q, *J* = 7.4 Hz, 1H), 2.74 (dd, *J* = 13.8, 7.2 Hz, 1H), 2.46 – 2.34 (m, 2H), 2.09 – 1.99 (m, 1H), 1.97 (dd, *J* = 13.7, 8.1 Hz, 1H), 1.94 – 1.85 (m, 1H). **¹³C NMR** (201 MHz, CDCl₃) δ 204.1, 154.0, 147.5, 145.1, 130.6, 129.6, 129.3, 127.0, 126.5, 124.3, 95.8, 74.6, 57.5, 53.6, 45.6, 39.1, 33.1. **HRMS** (+APCI) calculated for C₁₇H₁₇O₃NCl₃ [M+H]⁺ 388.0269, found 388.0272. **HPLC** (IH column 20% 2-propanol in hexanes, 1.0 mL/min) (**32**): *t*_M = 13.4 min *t*_m = 15.5 min, 89:11 e.r.

32': Colorless oil (0.0106 g, 26% yield, 86:14 e.r.). **¹H NMR** (800 MHz, CDCl₃) δ 7.49 (d, *J* = 9.8 Hz, 1H), 7.44 (t, *J* = 7.5 Hz, 1H), 7.39 (d, *J* = 7.9 Hz, 1H), 7.33 (d, *J* = 7.4 Hz, 1H), 7.30 (t, *J* = 7.3 Hz, 1H), 6.43 (d, *J* = 8.6 Hz, 1H), 6.21 (dd, *J* = 9.7, 1.2 Hz, 1H), 4.75 (s, 2H), 4.59 (dh, *J* = 9.0, 4.9 Hz, 1H), 2.49 (dd, *J* = 14.6, 7.8 Hz, 1H), 2.36 (dt, *J* = 14.1, 7.4 Hz, 1H), 2.33 – 2.23 (m, 2H), 2.02 (dq, *J* = 12.7, 6.4 Hz, 1H), 1.89 (dt, *J* = 13.7, 6.8 Hz, 1H). **¹³C NMR** (201 MHz, CDCl₃) δ 205.9, 154.3, 147.9, 146.2, 130.9, 129.7, 128.9, 127.1, 126.2, 124.3, 95.9, 74.6, 57.9, 54.4, 45.4, 42.3, 34.6. **HRMS** (+APCI) calculated for C₁₇H₁₇O₃NCl₃ [M+H]⁺ 388.0269, found 388.0272. **HPLC** (IK column 20% 2-propanol in hexanes, 1.0 mL/min) (**32'**): *t*_M = 10.0 min *t*_m = 8.6 min, 86:14 e.r.

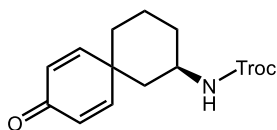

2,2,2-trichloroethyl (*R*)-(9-oxospiro[5.5]undeca-7,10-dien-2-yl)carbamate (**33**): Prepared using **General Procedure F** using **S14**, (*R*)-**8**, and (*S,S*)-**5**. Purified via preparative TLC using (20% Acetone in Hexanes) to provide **33** (0.0299 g, 82% yield, 91:9 e.r.) as a colorless oil. **¹H NMR** (800 MHz, CDCl₃) δ 7.31 (dd, *J* = 10.4, 3.0 Hz, 1H), 6.67 (dd, *J* = 10.0, 3.0 Hz, 1H), 6.31 (d, *J* = 10.3 Hz, 1H), 6.23 (d, *J* = 9.9 Hz, 1H), 4.96 (s, 1H), 4.69 (s, 2H), 3.87 (tdt, *J* = 12.1, 8.1, 4.0 Hz, 1H), 2.20 (d, *J* = 12.4 Hz, 1H), 1.88 (dt, *J* = 14.0, 3.6 Hz, 1H), 1.83 (d, *J* = 12.4 Hz, 1H), 1.74 (qt, *J* = 13.5, 4.1 Hz, 1H), 1.59 – 1.50 (m, 2H), 1.44 (t, *J* = 12.4 Hz, 1H), 1.27 (qd, *J* = 12.7, 4.0 Hz, 1H). **¹³C NMR** (201 MHz, CDCl₃) δ 185.8, 156.2, 153.8, 150.5, 129.2, 128.4, 95.6, 74.6, 46.8, 41.8, 41.2, 34.6, 32.6, 21.2. **HRMS** (+APCI) calculated for C₁₄H₁₇O₃NCl₃ [M+H]⁺ 352.0269, found 352.0272. **HPLC** (IB column 20% 2-propanol in hexanes, 1.0 mL/min) *t*_M = 9.1 min *t*_m = 10.4 min, 91:9 e.r.

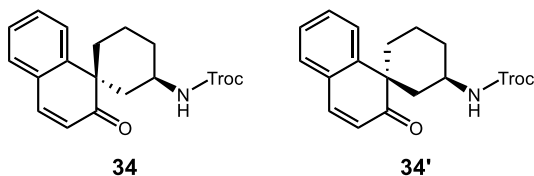

2,2,2-trichloroethyl ((1*S*,3*R*)-2'-oxo-2'-*H*-spiro[cyclohexane-1,1'-naphthalen]-3-yl)carbamate (**34**) and 2,2,2-trichloroethyl ((1*R*,3*R*)-2'-oxo-2'-*H*-spiro[cyclohexane-1,1'-naphthalen]-3-yl)carbamate (**34'**): Prepared using **General Procedure F** using **S15**, (*R*)-**8**, and (*S,S*)-**5**. Purified via preparative TLC using (20% EtOAc in Hexanes) to provide **34** and **34'** as a colorless oil and mixture of inseparable diastereomers (0.0177 g, 43% yield, 4:1 d.r., 88:12 e.r.). **¹H NMR** (800 MHz, CDCl₃) δ 7.59 (d, *J* = 8.0 Hz, 1H), 7.49 (d, *J* = 8.0 Hz, 1H, minor), 7.43 (t, *J* = 7.3 Hz, 1H), 7.38 (d, *J* = 9.8 Hz, 1H), 7.33 – 7.27 (m, 3H), 6.86 (d, *J* = 8.5 Hz, 1H), 6.12 (d, *J* = 9.7 Hz, 1H), 6.06 (d, *J* = 9.8 Hz, 1H, minor), 4.75 (d, *J* = 12.0 Hz, 2H), 4.73 (d, *J* = 12.0 Hz, 2H), 4.69 (d, *J* = 4.4 Hz, 2H, minor), 4.49 (tdt, *J* = 11.8, 8.3, 4.3 Hz, 1H, minor), 4.26 (dq, *J* = 11.0, 5.5 Hz, 1H), 2.32 (dd, *J* = 14.9, 5.9 Hz, 1H), 2.19 – 2.14 (m, 1H, minor), 2.09 (dd, *J* = 14.9, 5.9 Hz, 1H), 1.94 (ddd, *J* = 14.1, 7.4, 3.5 Hz, 1H), 1.89 – 1.81 (m, 1H), 1.76 (tdd, *J* = 12.6, 9.4, 3.2 Hz, 1H), 1.74 – 1.67 (m, 2H), 1.66 – 1.58 (m, 1H, minor), 1.55 (dt, *J* = 13.6, 6.8, 3.3 Hz, 1H). **¹³C NMR** (201 MHz, CDCl₃) δ 206.1, 154.2, 146.7, 144.9, 130.3, 130.2, 129.7, 127.2, 126.9, 124.2, 96.0, 74.5, 52.5, 46.0, 37.3, 35.3, 31.2, 17.9. **HRMS** (+APCI) calculated for C₁₈H₁₉O₃NC₃ [M+H]⁺ 402.0425, found 402.0432. **HPLC** (IK column 5% 2-propanol in hexanes, 1.0 mL/min) (**34**): *t*_M = 38.5 min *t*_m = 29.8 min, 88:12 e.r. (**34'**): *t*_M = 52.9 min *t*_m = 42.6 min, 76:24 e.r.

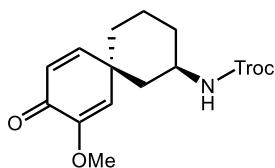

2,2,2-trichloroethyl ((2*R*,6*R*)-8-methoxy-9-oxospiro[5.5]undeca-7,10-dien-2-yl)carbamate (**35**): Prepared using **General Procedure F** using **S16**, (*R*)-**8**, and (*S,S*)-**5**. Purified via preparative TLC using (50% EtOAc in Hexanes) to provide **35** as a colorless oil (0.0142 g, 37% yield, >20:1 d.r., 89:11 e.r.). **¹H NMR** (800 MHz, CDCl₃) δ 6.69 (dd, *J* = 9.9, 2.6 Hz, 1H), 6.28 (d, *J* = 9.9 Hz, 1H), 6.15 (d, *J* = 2.7 Hz, 1H), 4.87 (s, 1H), 4.75 – 4.65 (m, 2H), 3.88 (tdt, *J* = 12.1, 8.1, 4.0 Hz, 1H), 3.71 (s, 3H), 2.23 (d, *J* = 13.0 Hz, 1H), 1.90 (dt, *J* = 14.2, 3.8 Hz, 1H), 1.80 (d, *J* = 12.3 Hz, 1H), 1.74 (qt, *J* = 13.7, 3.7 Hz, 1H), 1.59 (td, *J* = 13.4, 4.0 Hz, 1H), 1.53 – 1.40 (m, 2H), 1.29 – 1.21 (m, 2H). **¹³C NMR** (201 MHz, CDCl₃) δ 180.9, 156.5, 153.8, 151.3, 127.7, 116.4, 95.6, 74.6, 55.1, 47.1, 42.3, 42.0, 35.4, 32.7, 21.5. **HRMS** (+APCI) calculated for C₁₅H₁₉O₄NC₃ [M+H]⁺ 382.0374, found 382.0377. **HPLC** (IB column 20% 2-propanol in hexanes, 1.0 mL/min) (**4.60**): *t*_M = 8.6 min *t*_m = 10.0 min, 89:11 e.r.

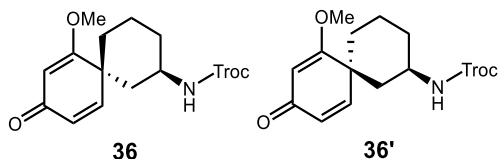

2,2,2-trichloroethyl ((2*R*,6*R*)-7-methoxy-9-oxospiro[5.5]undeca-7,10-dien-2-yl)carbamate (**36**) and 2,2,2-trichloroethyl ((2*R*,6*S*)-7-methoxy-9-oxospiro[5.5]undeca-7,10-dien-2-yl)carbamate (**36'**): Prepared using **General Procedure F** using **S17**, (*R*)-**8**, and (*S,S*)-**5**. Purified via preparative TLC using (20% Acetone in Hexanes) to provide **36** and **36'** as a colorless oil and mixture of inseparable diastereomers (0.0182 g, 47% yield, 1.2:1 d.r., 81:19 e.r.). ¹H NMR (800 MHz, CDCl₃) δ 7.10 (d, *J* = 10.2 Hz, 1H), 6.44 (d, *J* = 9.9 Hz, 1H, minor), 6.23 (dd, *J* = 10.2, 1.6 Hz, 1H), 6.09 (dd, *J* = 9.9, 1.6 Hz, 1H, minor), 5.58 (d, *J* = 1.6 Hz, 1H), 5.49 (d, *J* = 1.7 Hz, 1H, minor), 4.87 (dd, *J* = 16.5, 8.1 Hz, 1H), 4.71 – 4.66 (m, 2H), 4.21 (ddp, *J* = 10.9, 6.9, 4.1 Hz, 1H, minor), 3.91 (tdt, *J* = 12.4, 8.5, 4.2 Hz, 1H), 3.77 (s, 3H, minor), 3.73 (s, 4H), 2.21 – 2.14 (m, 1H), 2.11 – 2.05 (m, 2H), 2.04 – 1.94 (m, 2H), 1.91 – 1.82 (m, 2H), 1.76 (dq, *J* = 17.6, 6.6, 5.3, 2.2 Hz, 3H), 1.52 (ddd, *J* = 14.7, 12.1, 4.8 Hz, 1H, minor), 1.47 – 1.43 (m, 1H), 1.41 (dd, *J* = 13.7, 10.6 Hz, 1H, minor), 1.31 – 1.19 (m, 3H). ¹³C NMR (201 MHz, CDCl₃) δ 187.8, 187.0, 180.8, 178.4, 153.8, 153.7, 152.1, 146.8, 127.8, 126.7, 102.4, 101.1, 95.7, 95.7, 74.6, 74.5, 55.9, 55.8, 46.7, 46.6, 44.1, 43.4, 39.9, 39.4, 34.0, 32.9, 32.6, 31.6, 20.9, 20.8. HRMS (+APCI) calculated for C₁₅H₁₉O₄NCl₃ [M+H]⁺ 382.0374, found 382.0380. HPLC (IK column 30% 2-propanol in hexanes, 1.0 mL/min) (**36**): *t*_M = 18.5 min *t*_m = 12.4 min, 81:19 e.r. (**36'**): *t*_M = 8.7 min *t*_m = 10.9 min, 80:20 e.r.

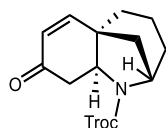

2,2,2-trichloroethyl (2*R*,5*aR*,9*aS*)-8-oxo-2,3,4,5,9,9*a*-hexahydro-2,5*a*-methanobenzo[*b*]azepine-1(8*H*)-carboxylate (**39**): Prepared using **General Procedure F** using **37**, (*R*)-**8**, and (*S,S*)-**5**. Purified via preparative TLC using (40% EtOAc in Hexanes) to provide **39** as a colorless oil and mixture of two conformers (0.0058 g, 15% yield,). ¹H NMR (800 MHz, CDCl₃) δ 6.57 (t, *J* = 10.2 Hz, 2H), 5.96 (d, *J* = 5.8 Hz, 1H), 5.94 (d, *J* = 5.7 Hz, 1H), 4.96 (d, *J* = 12.0 Hz, 1H), 4.81 (d, *J* = 12.0 Hz, 1H), 4.75 (d, *J* = 12.0 Hz, 1H), 4.55 (d, *J* = 12.0 Hz, 1H), 4.37 (t, *J* = 5.3 Hz, 1H), 4.34 (t, *J* = 5.3 Hz, 1H), 4.12 (d, *J* = 6.3 Hz, 1H), 4.10 (d, *J* = 6.3 Hz, 1H), 3.27 (ddd, *J* = 23.2, 16.4, 6.3 Hz, 2H), 2.41 (dd, *J* = 16.4, 11.5 Hz, 1H), 2.33 (dd, *J* = 16.3, 11.6 Hz, 1H), 2.27 – 2.23 (m, 1H), 2.23 – 2.19 (m, 1H), 2.19 – 2.13 (m, 2H), 1.75 (dt, *J* = 13.6, 5.5 Hz, 2H), 1.73 – 1.54 (m, 8H), 1.44 (td, *J* = 12.9, 5.4 Hz, 1H), 1.39 (td, *J* = 13.0, 5.6 Hz, 1H). ¹³C NMR (201 MHz, CDCl₃) δ 197.1, 197.0, 152.3, 152.4, 151.8, 151.5, 127.9, 127.7, 95.8, 95.7, 74.8, 74.7, 61.0, 60.6, 56.9, 56.8, 43.8, 43.0, 42.0, 40.9, 40.2, 39.5, 37.1, 29.2, 27.6, 18.5, 18.4. HRMS (+APCI) calculated for C₁₄H₁₇O₃N³⁵Cl₃ [M+H]⁺ 352.0269, found 352.0271. SFC (ChiralCel OJ-3 5% MeOH/IPA with 0.2% Formic Acid, 2.5 mL/min) (**39**): *t*_M = 7.42 min *t*_m = 5.36 min, 89:11 e.r.

Preparation of **39** via acidic conditions:

2,2,2-trichloroethyl (2*R*,5*aR*,9*aS*)-8-oxo-2,3,4,5,9,9*a*-hexahydro-2,5*a*-methanobenzo[*b*]azepine-1(8*H*)-carboxylate (**39**): Dissolved **33** (35.3 mg, 0.100 mmol) in DME (1.00 mL) under inert atmosphere. Made addition of methanesulfonic acid (7.4 μL, 1.1 equiv) and heated to 80 °C. After 8 hours continuous stirring made second addition of methanesulfonic acid (7.4 μL, 1.1 equiv) and stirred additional 8 hours. Quenched reaction with water (2 mL) and extracted with EtOAc (3x with 3 mL). Purified via preparative TLC using (20% Acetone in Hexanes) to provide **39** as a colorless oil (0.0246 g, 70%), matches previously reported spectra. SFC (ChiralCel OJ-3 5% MeOH/IPA with 0.2% Formic Acid, 2.5 mL/min) (**39**): *t*_M = 7.55 min *t*_m = 5.43 min, 90:10 e.r.

14. Failed Substrates/Limitations

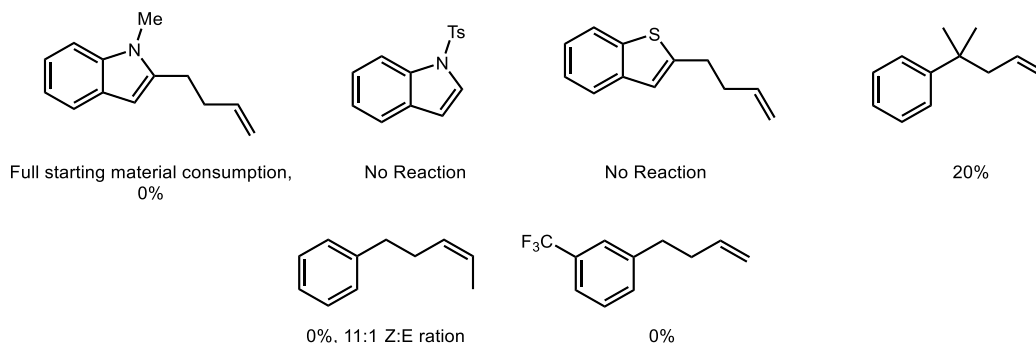

15. Synthetic Application

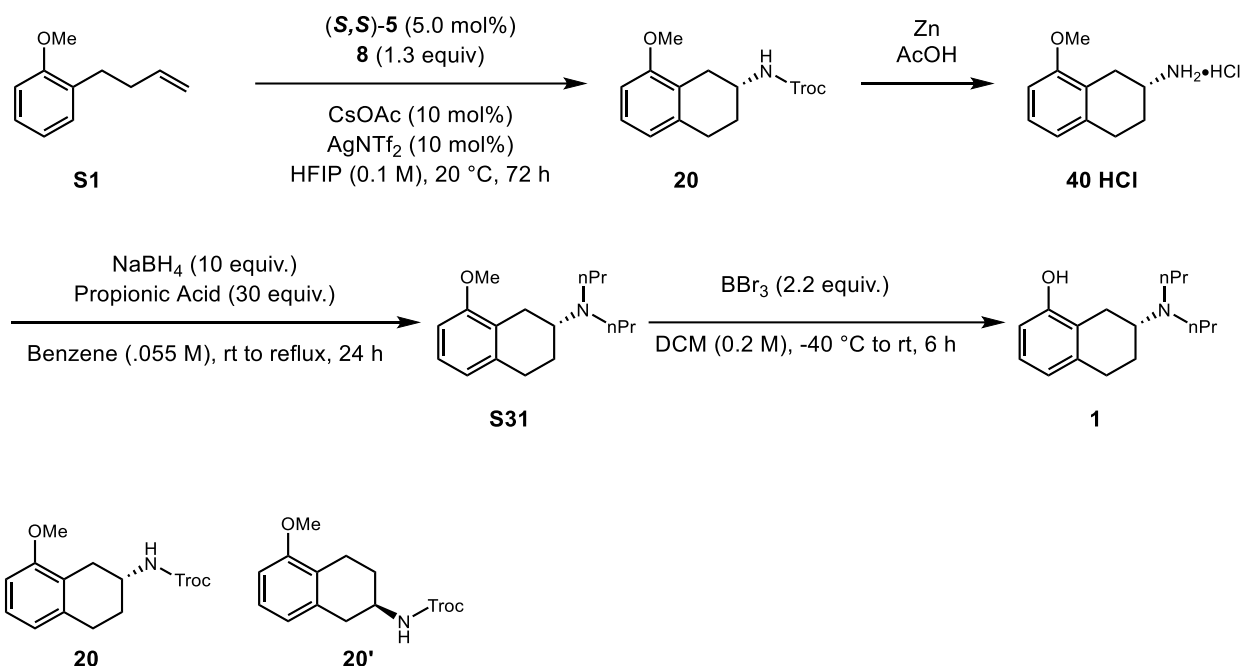

2,2,2-trichloroethyl (R)-(8-methoxy-1,2,3,4-tetrahydronaphthalen-2-yl)carbamate (20) and **2,2,2-trichloroethyl (R)-(5-methoxy-1,2,3,4-tetrahydronaphthalen-2-yl)carbamate (20')**: Prepared using a modified version of **General Procedure F** on a 1.00 mmol scale, where **S1** was added in 5.0 mL of HFIP via syringe pump over the course of 24 hours to a stirring solution of **(R)-8**, **(S,S)-5** (5.0 mol% catalyst loading), CsOAc, and AgNTf₂. 48 hours of additional stirring was performed after the addition of **S1**, at which point the crude reaction was filtered through a Celite syringe plug using DCM to flush. The solvent was removed under reduced pressure and the crude material purified using flash chromatography with 5% acetone in hexanes to provide **20** and **20'** as a slowly solidifying white solid and mixture of inseparable regioisomers (0.2338 g, 66% yield, 5:1 r.r., 88:12 e.r.). ¹H NMR (400 MHz, CDCl₃) δ 7.12 (t, *J* = 7.9 Hz, 1H), 6.76 – 6.70 (m, 1H), 6.68 (d, *J* = 8.0 Hz, 1H), 5.14 (d, *J* = 10.0 Hz, 1H), 4.79 – 4.68 (m, 2H), 4.12 – 3.99 (m, 1H), 3.82 (s, 3H, minor regioisomer), 3.81 (s, 3H, major regioisomer), 3.13 (dd, *J* = 17.2, 5.6 Hz, 1H), 2.89 (t, *J* = 6.3 Hz, 2H), 2.51 (dd, *J* = 17.3, 8.1 Hz, 1H), 2.13 – 2.01 (m, 1H), 1.88 – 1.73 (m, 1H). ¹³C NMR (101 MHz, CDCl₃) δ 157.6, 154.0, 136.8, 126.7, 122.8, 121.7, 121.1, 107.5, 107.2, 95.8, 74.6, 55.4, 47.2, 46.9, 35.8, 29.9, 28.4, 28.2, 27.2, 21.0. HPLC (IB column 10% 2-propanol in

hexanes, 1.0 mL/min) (**20**): $t_M = 9.6$ min $t_m = 12.0$ min, 88:12 e.r (**20'**): $t_M = 11.0$ min $t_m = 9.1$ min, 89:11 e.r.

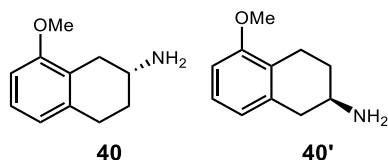

(*R*)-8-methoxy-1,2,3,4-tetrahydronaphthalen-2-amine hydrochloride (**40**) and (*R*)-5-methoxy-1,2,3,4-tetrahydronaphthalen-2-amine hydrochloride (**40'**): To a 7 mL vial was added a mixture of **20** and **20'** (0.190 g, 0.539 mmol, 1.0 equiv). Atmosphere was exchanged to an N₂ atmosphere, followed by the addition of AcOH (3.00 mL, 52.3 mmol, 97.0 equiv). Vial was briefly opened to air and zinc dust (0.352 g, 5.39 mmol, 10 equiv) was added in one portion. Vial was placed back under N₂ atmosphere and allowed to stir at room temperature for 4 hours. After reaction was completed, it was the reaction with methanol, passed through a celite plug, and concentrated under vacuum. The crude material was basified with NH₄OH (10 mL) and extracted with EtOAc (3x). This solution was concentrated under vacuum and then treated with 5 mL of 2.0 M HCl in Et₂O. This material was washed with EtOAc and filtered through a celite plug. Once vessel containing material adequately washed with EtOAc, remaining material in reaction vial was dissolved in MeOH and passed through same plug as EtOAc was pushing all MeOH soluble material into a clean vial. This was then concentrated under vacuum to collect product **40** and **40'** HCl as a white solid and mixture of inseparable regioisomers. Washed with ammonium hydroxide to yield **40** and **40'** as free amine (.0689 g, 72% yield). **¹H NMR** (400 MHz, CDCl₃) δ 7.09 (t, $J = 7.9$ Hz, 1H), 6.75 – 6.69 (m, 1H), 6.66 (d, $J = 8.1$ Hz, 1H), 3.81 (s, 3H), 3.14 (dddd, $J = 10.3, 9.0, 5.4, 3.0$ Hz, 1H), 3.05 (dd, $J = 17.1, 4.8$ Hz, 1H, major regioisomer), 3.01 – 2.94 (m, 1H, minor regioisomer), 2.93 – 2.77 (m, 2H), 2.65 – 2.49 (m, 1H, minor regioisomer), 2.28 (dd, $J = 16.8, 9.2$ Hz, 1H, major regioisomer), 2.06 – 1.91 (m, 1H), 1.63 – 1.48 (m, 3H). **¹³C NMR** (101 MHz, CDCl₃) δ 157.5, 137.4, 136.8, 126.3, 126.2, 124.9, 124.3, 121.6, 121.0, 107.2, 107.0, 55.4, 47.4, 47.1, 39.6, 33.5, 32.5, 28.5, 22.3. **HRMS** (+APCI) calculated for C₁₁H₁₆ON [M+H]⁺ 178.1226, found 178.1228.

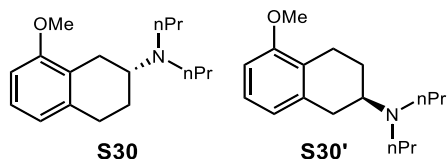

(*R*)-8-methoxy-*N,N*-dipropyl-1,2,3,4-tetrahydronaphthalen-2-amine (**S30**) and (*R*)-5-methoxy-*N,N*-dipropyl-1,2,3,4-tetrahydronaphthalen-2-amine (**S30'**): Prepared using **40** and **40'** in the free amine form. In a 7 mL vial, to a solution of propionic acid (0.210 mL, 30 equiv) in benzene (0.85 mL) sodium borohydride (0.0354 mg, 10 equiv) was added in one portion and stirred at room temperature until gas evolution ceased. Amine was added as a stock solution (16.6 mg, 0.094 mmol, 0.11 M) to bring total reaction volume to 1.7 mL. Reaction was heated to reflux for 20 hours, cooled to room temperature, basified using NaOH, and extracted from H₂O with DCM (3x). Purified via preparatory TLC using 5% EtOAc and 5% triethylamine in hexanes to collect product **S30** and **S30'** as a colorless oil and mixture of inseparable regioisomers. (0.014 g, 57%). **¹H NMR** (800 MHz, CDCl₃) δ 7.08 (t, $J = 7.8$ Hz, 1H), 6.71 (d, $J = 7.6$ Hz, 1H), 6.66 (d, $J = 7.9$ Hz, 1H), 3.82 (s, 3H, major regioisomer), 3.81 (s, 3H, minor regioisomer), 3.02 – 2.91 (m,

2H), 2.88 (ddd, $J = 16.6, 5.3, 2.5$ Hz, 1H), 2.83 (ddd, $J = 16.8, 12.0, 5.3$ Hz, 1H), 2.50 (m, 4H), 2.43 (dd, $J = 18.2, 12.4$ Hz, 1H), 2.08 – 1.95 (m, 1H), 1.60 (qd, $J = 12.0, 5.1$ Hz, 1H, major regioisomer), 1.56 (dd, $J = 12.2, 5.7$ Hz, 1H, minor regioisomer), 1.48 (m, 4H), 0.89 (m, 6H). **^{13}C NMR** (201 MHz, CDCl_3) δ 157.7, 138.1, 126.2, 126.1, 121.8, 121.0, 107.0, 106.9, 57.0, 55.38, 55.4, 52.9, 52.8, 30.5, 26.1, 25.8, 25.5, 24.1, 22.5, 22.3, 12.1. **HRMS** (+APCI) calculated for $\text{C}_{17}\text{H}_{28}\text{ON}$ $[\text{M}+\text{H}]^+$ 262.2165, found 262.2164.

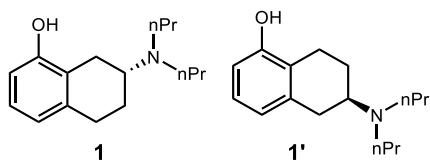

(*R*)-7-(dipropylamino)-5,6,7,8-tetrahydronaphthalen-1-ol (**1**) and (*R*)-6-(dipropylamino)-5,6,7,8-tetrahydronaphthalen-1-ol (**1'**): Prepared using **S30** and **S30'** mixture and an adapted procedure by Lautens.²⁵ In a 4 mL vial under inert atmosphere **S30/S30'** mixture (0.0072 g, 0.028 mmol) was dissolved in DCM (.092 mL) at -40 °C. To stirring solution, BBr_3 (0.061 mL, 2 equiv) was added, and reaction was stirred for 2h at -40 °C and then warmed to room temperature to stir for an additional 3h. Reaction was quenched with saturated NaHCO_3 , extracted with DCM (3x) and concentrated under vacuum. Purified via preparatory TLC using 10% EtOAc and 5% triethylamine in hexanes to collect products **1** and **1'** as a colorless oil and mixture of inseparable regioisomers. (0.0043, 63% yield). **^1H NMR** (800 MHz, CDCl_3) δ 6.98 (m, 1H), 6.70 – 6.66 (m, 1H), 6.61 (d, $J = 7.9$ Hz, 1H, major regioisomer), 6.59 (d, $J = 7.8$ Hz, 1H, minor regioisomer), 3.00 (tdd, $J = 11.6, 5.3, 2.7$ Hz, 1H, major regioisomer), 2.93 (m, 1H, minor regioisomer), 2.90 – 2.85 (m, 2H), 2.81 (m, 1H), 2.53 – 2.45 (m, 5H), 2.03 – 1.99 (m, 1H), 1.60 (qd, $J = 12.3, 5.0$ Hz, 1H), 1.52 – 1.45 (m, 4H), 0.89 (m, 6H). **^{13}C NMR** (201 MHz, CDCl_3) δ 151.0, 138.5, 126.5, 126.4, 123.2, 122.0, 121.0, 112.0, 112.0, 57.1, 56.6, 52.8, 52.8, 30.4, 29.9, 25.8, 25.7, 25.5, 23.8, 22.3, 22.2, 12.1. **HRMS** (+APCI) calculated for $\text{C}_{16}\text{H}_{26}\text{ON}$ $[\text{M}+\text{H}]^+$ d 248.2009, found 248.2006.

16. Confirmation of Product Stereochemistry

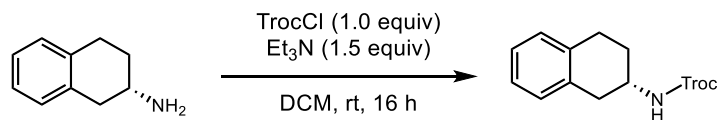

2,2,2-trichloroethyl (*S*)-(1,2,3,4-tetrahydronaphthalen-2-yl)carbamate ((**S**)-**10**): Under an N_2 atmosphere in a 7 mL vial equipped with stir bar, (*S*)-2-aminotetralin (0.075 g, 0.50 mmol, 1.0 equiv) and triethylamine (0.11 mL, 0.75 mmol, 1.5 equiv) were dissolved in DCM (2.5 mL). To this solution 2,2,2-trichloroethyl carbonochloridate (TrocCl) (0.08 mL, 0.50 mmol, 1.0 equiv) was added and the reaction was stirred at room temperature overnight. The reaction was quenched by the addition of 1M HCl followed by extraction with DCM (3x). The combined organic layers were washed with brine (1x) and dried over Na_2SO_4 before removing the solvent under reduced pressure. The crude product was purified using flash column chromatography with 10% EtOAc in hexanes to provide (**S**)-**10** as a colorless oil (0.097 g, 59% yield). **^1H NMR** (400 MHz, CDCl_3) δ 7.23 – 6.99 (m, 4H), 5.08 (d, $J = 7.6$ Hz, 1H), 4.76 (d, $J = 12.1$ Hz, 1H), 4.72 (d, $J = 12.0$ Hz, 1H), 4.09 (ddtd, $J = 13.1, 8.2, 5.1, 3.1$ Hz, 1H), 3.18 (dd, $J = 16.3, 5.1$ Hz, 1H), 2.99 – 2.83 (m, 2H),

2.72 (dd, $J = 16.3, 8.1$ Hz, 1H), 2.18 – 2.05 (m, 1H), 1.91 – 1.78 (m, 1H). ^{13}C NMR (101 MHz, CDCl_3) δ 154.0, 135.4, 133.8, 129.6, 129.0, 126.4, 126.2, 95.7, 74.6, 47.3, 35.8, 28.8, 27.0. **HRMS** (+APCI) calculated for $\text{C}_{13}\text{H}_{15}\text{O}_2\text{NCl}_3$ $[\text{M}+\text{H}]^+$ 322.0163, found 322.0168. **HPLC** (IJ column 5% 2-propanol in hexanes, 1.0 mL/min) $t_{\text{M}} = 15.2$ min

2,2,2-trichloroethyl (S)-(1,2,3,4-tetrahydronaphthalen-2-yl)carbamate ((S)-10):

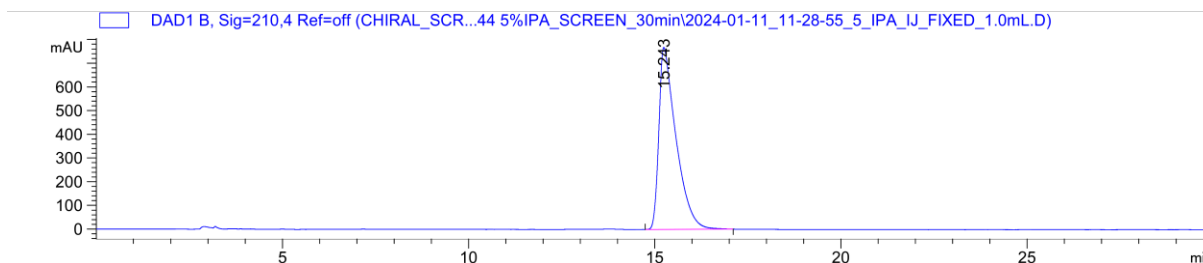

Signal 2: DAD1 B, Sig=210,4 Ref=off

Peak #	RetTime [min]	Type	Width [min]	Area [mAU*s]	Height [mAU]	Area %
1	15.243	BV R	0.3815	2.45226e4	767.96552	100.0000

Totals : 2.45226e4 767.96552

2,2,2-trichloroethyl (R)-(1,2,3,4-tetrahydronaphthalen-2-yl)carbamate ((R)-10):

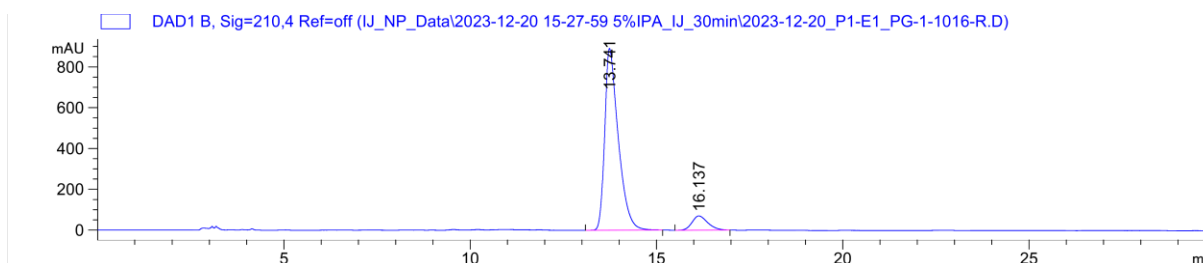

Signal 2: DAD1 B, Sig=210,4 Ref=off

Peak #	RetTime [min]	Type	Width [min]	Area [mAU*s]	Height [mAU]	Area %
1	13.741	VV R	0.3316	2.25000e4	891.12781	91.6616
2	16.137	VV R	0.3432	2046.81458	69.97881	8.3384

Totals : 2.45468e4 961.10662

Based on the comparison of the HPLC traces of (S)-10 with the sample of 10 prepared by using (R)-8, and (S,S)-5 the product stereochemistry of the 2-aminotetralins and the spirocycles has been assigned to be (R)

17. Synthesis of Substrates for Mechanistic Studies

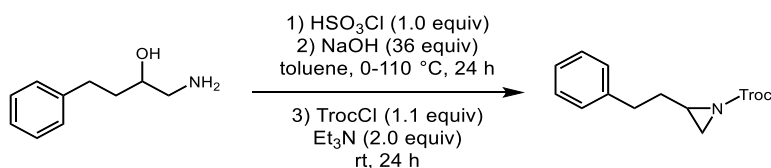

2,2,2-trichloroethyl 2-phenethylaziridine-1-carboxylate (12): Using an adapted procedure by Doyle.²⁶ Under an N_2 atmosphere, in a 100 mL RBF equipped with a stir bar, 1-amino-4-phenylbutan-2-ol²⁷ (0.500 g, 3.0 mmol, 1.0 equiv) was dissolved in Toluene (20 mL) and the reaction vessel placed in an ice bath. Once cool, chlorosulfonic acid (0.20 mL, 3.0 mmol, 1.0 equiv) was added and the reaction stirred at room temperature. After two hours NaOH (18 mL, 6M, 36 equiv) was added, a condenser attached, and the reaction was refluxed for 24 h. The reaction was cooled to room temperature and extracted with Et_2O (3x) and washed with brine (1x). The combined organic layers were dried over MgSO_4 and the Et_2O removed under reduced pressure. The remaining toluene solution containing the crude unprotected aziridine was placed in a 100 mL RBF and the reaction vessel was placed in an ice bath. Once cool, Et_3N (0.85 mL, 6.0 mmol, 2.0 equiv) was added followed by TrocCl (0.46 mL, 3.3 mmol, 1.1 equiv) and the reaction was stirred at room temperature overnight. The reaction was quenched by adding sat. NaHCO_3 and then extracting with DCM (3X). The combined organic layers were dried over MgSO_4 and the solvent removed under reduced pressure. The crude product was first purified by silica gel column chromatography using 10% EtOAc in Hexanes followed by Kugelrohr distillation to provide **12** as a colorless oil (0.412 g, 42%). **^1H NMR** (400 MHz, CDCl_3) δ 7.34 – 7.27 (m, 2H), 7.25 – 7.16 (m, 3H), 4.79 (d, J = 12.0 Hz, 1H), 4.75 (d, J = 12.0 Hz, 1H), 2.97 – 2.76 (m, 2H), 2.61 (dddd, J = 7.2, 6.1, 5.2, 3.8 Hz, 1H), 2.45 (d, J = 6.1 Hz, 1H), 2.10 (d, J = 3.8 Hz, 1H), 1.94 – 1.76 (m, 2H). **^{13}C NMR** (101 MHz, CDCl_3) δ 161.8, 141.2, 128.6, 128.6, 126.2, 95.2, 75.5, 38.6, 34.2, 33.3, 32.4. **HRMS** (+APCI) calculated for $\text{C}_{13}\text{H}_{15}\text{O}_2\text{NCl}_3$ $[\text{M}+\text{H}]^+$ 322.0163, found 322.0167.

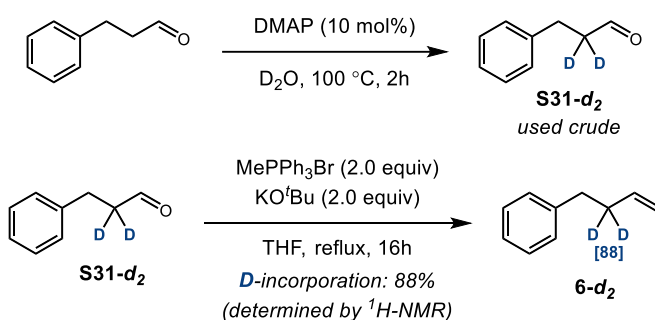

3-phenylpropanal-2,2- d_2 (S31- d_2): Using an adapted procedure by Zhang.⁷ In a 24 mL reaction equipped with a stir bar, 3-phenylpropanal (3.30 mL, 25.0 mmol, 1.0 equiv) and DMAP (0.31 g, 2.5 mmol, 0.1 equiv) were dissolved in D_2O (5.0 mL). The reaction vessel was sealed, placed in an aluminum heating block and heated at 100 °C. After two hours, the reaction vessel was cooled to room temperature and the reaction was extracted with DCM (3x10 mL). The combined organic layers were washed with 1M HCl (10 mL), sat. NaHCO_3 (10 mL) and brine (10 mL) before drying over Na_2SO_4 . The solvent was removed, and the crude material was used directly in the next step.

(*but-3-en-1-yl-2,2-d₂*)benzene (**6-d₂**): Under an N₂ atmosphere, in a 250 RBF equipped with a stir bar and condenser methyltriphenylphosphonium bromide was dissolved in THF (125 mL) and the reaction vessel placed in an ice bath. Once cool, the vessel was quickly opened and potassium tert-butoxide (5.66 g, 50 mmol, 2.0 equiv) was added quickly. The reaction was sealed, and the yellow solution was stirred for one hour. Crude **S32-d₂** was dissolved in THF (10 mL) and added to the reaction before placing in heating block and refluxed overnight. The reaction was cooled to room temperature and placed in an ice bath before quenching with sat. NH₄Cl. The reaction was extracted with Et₂O (3x) and washed with brine (1x). The combined organic layers were dried over MgSO₄ and the solvent removed under reduced pressure. The crude reaction mixture was purified using silica gel column chromatography using Pentane to provide **6-d₂** as colorless oil (2.25 g, 67%) **¹H NMR** (400 MHz, CDCl₃) δ 7.36 – 7.27 (m, 2H), 7.21 (dd, *J* = 7.1, 1.8 Hz, 3H), 5.88 (dd, *J* = 17.1, 10.3 Hz, 1H), 5.07 (dt, *J* = 17.2, 1.9 Hz, 1H), 5.00 (dt, *J* = 10.2, 1.8 Hz, 1H), 2.72 (s, 2H). Spectroscopic data for **6-d₂** matches those previously reported in the literature.²⁸

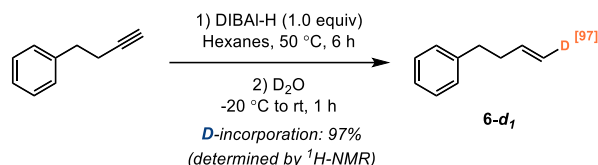

(*E*)-(but-3-en-1-yl-4-d)benzene (**E-6-d₁**): Using an adapted procedure by Hirino and Murana.²⁹ Under an N₂ atmosphere, 3-butenylbenzene (0.70 mL, 5.00mmol, 1.0 equiv) was dissolved in Hexanes (27 mL) followed by the dropwise addition of DIBAL-H (14.4 mL, 1.0M in toluene) at room temperature. The reaction was placed in an alumina heating block and stirred at 50 °C for 6 hours. Afterwards, the reaction was cooled to -20 °C and D₂O (0.70 mL, 5.00mmol, 1.0 equiv) was added and the reaction was stirred for an additional hour at room temperature. To the reaction sat. NH₄Cl was added and the resulting suspension was filtered through alumina. The filtrate was dried over Na₂SO₄ and the solvent removed under reduced pressure to afford the crude product which was purified via flash column chromatography (100% Pentane) to afford **E-6-d₁** as a colorless oil (1.0 g, 60%). **¹H NMR** (800 MHz, CDCl₃) δ 7.32 – 7.27 (m, 2H), 7.23 – 7.17 (m, 3H), 5.92 – 5.81 (m, 1H), 5.08 – 5.01 (m, 1H), 2.72 (t, *J* = 7.8 Hz, 2H), 2.42 – 2.35 (m, 2H). **¹³C NMR** (201 MHz, CDCl₃) δ 142.0, 138.1, 128.6, 128.4, 126.0, 114.8 (t, *J* = 24.2 Hz), 35.6, 35.5. **HRMS** (+APCI) calculated for C₁₀H₁₂D [M+H]⁺ 134.1075, found 134.1076.

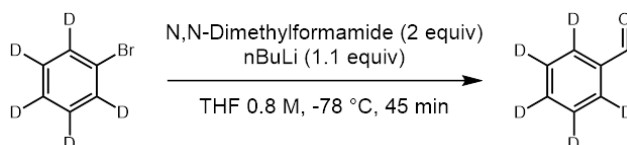

benzaldehyde-2,3,4,5,6-d₅ (**S32-d₅**): Using an adapted procedure by Liu.³⁰ Under N₂ atmosphere, to a solution of d₅-bromobenzene (2.40 g, 14.8 mmol) in anhydrous THF (18.1 mL) was added a solution n-BuLi in hexane (6.85 mL, 2.5 M, 1.1 equiv) dropwise at -78 °C. The mixture was stirred at this temperature for 30 minutes and then N,N dimethylformamide (2.32 mL, 2 equiv) was added and allowed to stir until reaching ambient temperature. The reaction was then quenched with H₂O, extracted with EtOAc (3x), dried over MgSO₄ and concentrated under vacuum. Used crude in following step.

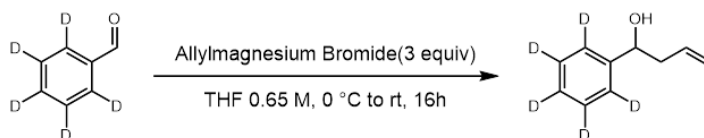

1-(phenyl-*d*₅)but-3-en-1-ol (S33-*d*₅): Prepared using General Procedure C, **S32-*d*₅** (1.65g, 14.8 mmol) and allylmagnesium bromide (46.9 mL, 1.0M in Et₂O, 3 equiv). Purified via flash chromatography with 5% acetone in hexanes to produce **S33-*d*₅** as a yellow oil (1.373 g, 60% yield). **¹H NMR** (400 MHz, CDCl₃) δ 5.82 (dddd, *J* = 17.0, 10.2, 7.6, 6.6 Hz, 1H), 5.21 – 5.12 (m, 2H), 4.79 – 4.70 (m, 1H), 2.59 – 2.44 (m, 2H), 2.14 – 2.03 (m, 1H). **¹³C NMR** (101 MHz, CDCl₃) δ 143.8, 134.6, 128.6 – 127.7 (m), 127.6 – 126.5 (m), 126.1 – 124.9 (m), 118.6, 73.3, 44.0. **HRMS** (+APCI) calculated for C₁₀H₄²H₅O [M-H]⁺ 289.9759, found 289.9767.

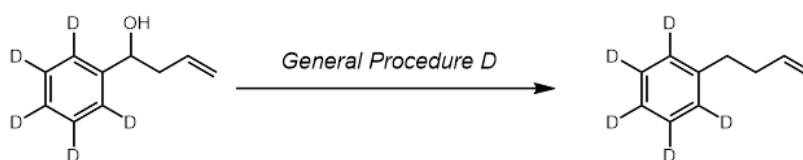

1-(but-3-en-1-yl)benzene-2,3,4,5,6-*d*₅ (6-*d*₅): Prepared using General Procedure D and **S33-*d*₅** (0.300 g, 1.96 mmol). Purified via flash chromatography with 100% pentane as eluent to yield **6-*d*₅** as a colorless oil (0.0313 g, 12% yield). **¹H NMR** (400 MHz, CDCl₃) δ 5.87 (ddt, *J* = 16.8, 10.2, 6.5 Hz, 1H), 5.05 (dq, *J* = 17.1, 1.7 Hz, 1H), 5.00 – 4.94 (m, 1H), 2.75 – 2.67 (m, 2H), 2.43 – 2.34 (m, 2H). **¹³C NMR** (101 MHz, CDCl₃) δ 141.8, 138.3, 128.3 (d, *J* = 23.3 Hz), 127.8 (d, *J* = 23.6 Hz), 125.2, 115.0, 35.7, 35.4. **MS data note: substrate too nonpolar to ionize via ESI, spectra taken using Agilent 5977C GC/MSD.** Expected mass of 137.1, detected 137.1

18. Mechanistic Studies

Intermediate Aziridine

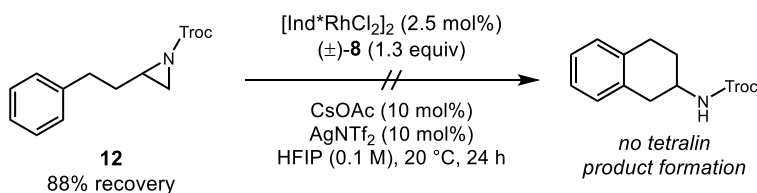

In an oven-dried 4 mL reaction vial, with Teflon tape wrapped threads, and equipped with an oven dried stir bar was brought into the glovebox. To the vial, CsOAc (0.01mmol, 0.1 equiv.), AgNTf₂ (0.01 mmol, 0.1 equiv.), and [Ind*RhCl₂]₂ (2.5 mol%) were added to the reaction vial. The vial was sealed with a Teflon septum screw cap and brought out of the box to complete the reaction. Under an N₂ atmosphere outside of the glovebox, (±)-**8** was transferred to the reaction as stock solution in HFIP (0.5 mL, 0.13 mmol, 1.3 equiv). The aziridine **12** (0.10 mmol 1.0 equiv.) was added to the reaction vial using HFIP washing the vial three times (0.2 mL + 0.2 mL + 0.1mL) to ensure complete transfer of the olefin. The reaction was left to stir at room temperature under an N₂ balloon for 24 hours. After 24 hours, the crude reaction was filtered through a Celite pipette plug using DCM to flush. The solvent was removed under reduced pressure, and the crude reaction

was analyzed by ^1H -NMR using dibromomethane as an NMR standard. No 2-aminotetralin product formation was observed and the aziridine **12** was recovered in a 88% yield.

Deuterium Labeling Studies

d_5 KIE Study

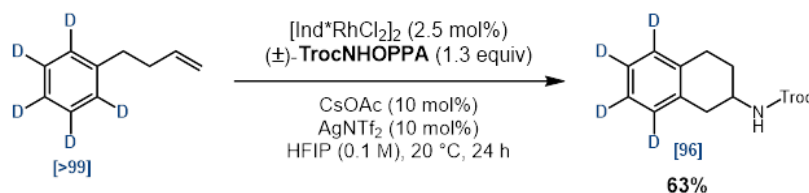

2,2,2-trichloroethyl (1,2,3,4-tetrahydronaphthalen-2-yl-5,6,7,8- d_4)carbamate (10- d_4**)** In an oven-dried 4 mL reaction vial, with Teflon tape wrapped threads, and equipped with an oven dried stir bar was brought into the glovebox. To the vial, CsOAc (0.01 mmol, 0.1 equiv.), AgNTf₂ (0.01 mmol, 0.1 equiv.), and [Ind*RhCl₂]₂ (2.5 mol%) were added to the reaction vial. The vial was sealed with a Teflon septum screw cap and brought out of the box to complete the reaction. Under an N₂ atmosphere outside of the glovebox, (±)-**8** was transferred to the reaction as stock solution in HFIP (0.5 mL, 0.13 mmol, 1.3 equiv). The deuterated substrate **6- d_5** (0.10 mmol 1.0 equiv.) was added to the reaction vial using HFIP washing the vial three times (0.2 mL + 0.2 mL + 0.1 mL) to ensure complete transfer of the olefin. The reaction was left to stir at room temperature under an N₂ balloon for 24 hours. After 24 hours, the crude reaction was filtered through a Celite pipette plug using DCM to flush. The solvent was removed under reduced pressure and the crude material purified via preparative TLC using the indicated eluent to yield the corresponding deuterated 2-aminotetralin (±)-**10- d_4** . The yield was determined to be 63% from the isolated product with <5% loss of deuterium incorporation. (0.0206 g, 63% yield) ^1H NMR (800 MHz, CDCl₃) δ 5.04 (d, J = 7.9 Hz, 1H), 4.74 (q, J = 12.0 Hz, 2H), 4.09 (dddt, J = 11.1, 8.2, 5.2, 3.2 Hz, 1H), 3.18 (dd, J = 16.2, 5.2 Hz, 1H), 2.96 – 2.87 (m, 2H), 2.72 (dd, J = 16.2, 8.1 Hz, 1H), 2.15 – 2.08 (m, 1H), 1.85 (dtd, J = 12.8, 8.6, 6.2 Hz, 1H). ^{13}C NMR (201 MHz, CDCl₃) δ 154.0, 135.3, 133.7, 129.4 – 128.9 (m), 128.6 (d, J = 23.9 Hz), 126.2 – 125.8 (m), 125.7 (d, J = 24.3 Hz), 74.6, 47.3, 35.8, 28.8, 27.0. HRMS (+APCI) calculated for C₁₃H₁₁²H₄O₂N³⁵Cl₃ [M+H]⁺ 326.0414, found 326.0412.

d_5 KIE Competition Study

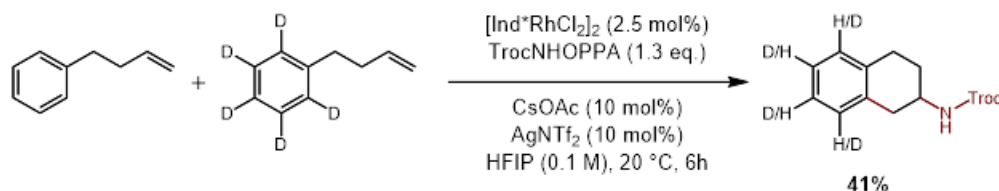

The deuterated substrate **6- d_5** and substrate **6** (0.065 mmol **6- d_5** and 0.068 mmol **6**, 18.2 mg total) were added to an oven dried 4 mL vial. Portion of mixture was taken for NMR sample, a total remaining mass of 15.8 mg **6- d_5** and **6** was left in the 4 mL vial. By NMR sample ratio is 1.00:1.03 **6- d_5** :**6** corresponding to 0.057 mmol **6- d_5** and 0.059 mmol **6**. Total mmol of reaction sample was 0.116 mmol and equivalents of other reagents were based on this value.

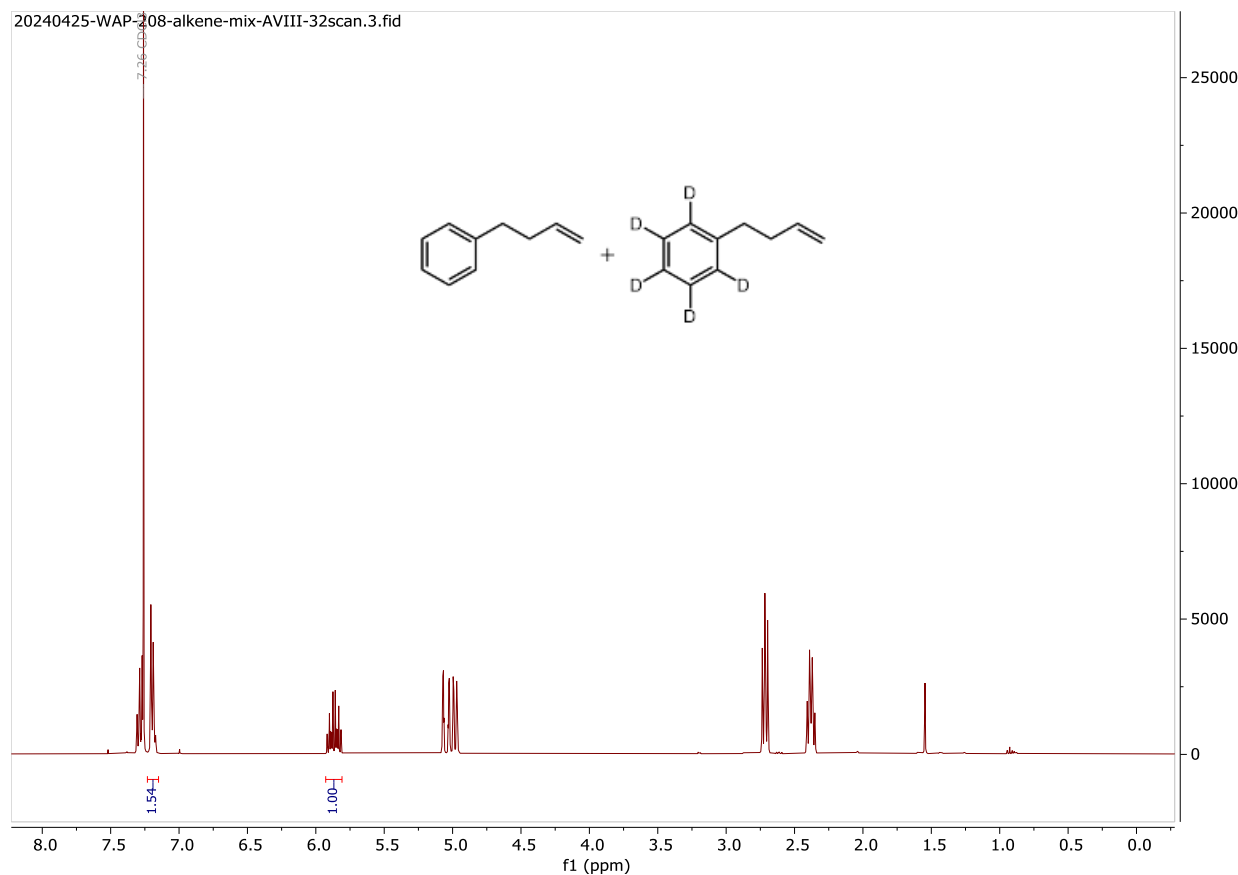

An oven-dried 4 mL reaction vial, with Teflon tape wrapped threads, and equipped with an oven dried stir bar was brought into the glovebox. CsOAc (0.0116 mmol, 0.1 equiv.), AgNTf₂ (0.0116 mmol, 0.1 equiv.), and [Ind*RhCl₂]₂ (2.5 mol%) were added to the reaction vial. The vial was sealed with a Teflon septum screw cap and brought out of the box to complete the reaction. Under an N₂ atmosphere outside of the glovebox, (±)-**8** was transferred to the reaction as stock solution in HFIP (0.580 mL, 0.13 mmol, 1.3 equiv). **6-d₅** and **6** sample mixture was added to reaction vial using HFIP washing the vial three times (0.2 mL + 0.2 mL + 0.18 mL) to ensure complete transfer of the olefin. The reaction was left stirring at room temperature under an N₂ balloon for 6 hours. After 6 hours, the crude reaction was filtered through a Celite pipette plug using DCM to flush. The solvent was removed under reduced pressure and the crude material purified via preparative TLC using the indicated eluent to yield the corresponding deuterated 2-aminotetralin product mixture (±)-**10-d₄**. The yield was determined to be 41% from the isolated product of mixed sample.

General Procedure G: 1,2-Arylation Procedure for Deuterium Labeling Studies

In an oven-dried 4 mL reaction vial, with Teflon tape wrapped threads, and equipped with an oven dried stir bar was brought into the glovebox. To the vial, CsOAc (0.01mmol, 0.1 equiv.), AgNTf₂ (0.30 mmol, 0.3 equiv.), and [Ind*RhCl₂]₂ (2.5 mol%) were added to the reaction vial. The vial was sealed with a Teflon septum screw cap and brought out of the box to complete the reaction. Under an N₂ atmosphere outside of the glovebox, (±)-**8** was transferred to the reaction as stock solution in HFIP (0.5 mL, 0.13 mmol, 1.3 equiv). The deuterated olefin substrate (0.10 mmol 1.0 equiv.) was added to the reaction vial using HFIP washing the vial three times (0.2 mL + 0.2 mL + 0.1mL) to ensure complete transfer of the olefin. The reaction was left to stir at room temperature under an N₂ balloon for 24 hours. After 24 hours, the crude reaction was filtered through a Celite pipette

plug using DCM to flush. The solvent was removed under reduced pressure and the crude material purified via preparative TLC using the indicated eluent to yield the corresponding deuterated 2-aminotetralin or spirocycle product. Deuterium incorporation was determined by analysis of the ^1H -NMR.

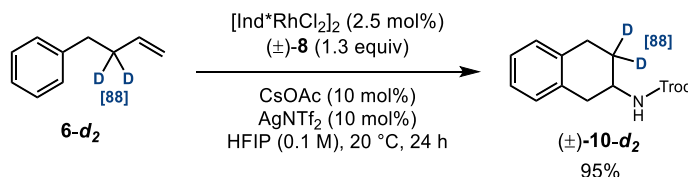

2,2,2-trichloroethyl (1,2,3,4-tetrahydronaphthalen-2-yl-3,3-d₂)carbamate ((±)-10-d₂): Prepared using **General Procedure G** using **6-d₂**. Purified via preparative TLC using (20% Acetone in Hexanes) to provide (±)-10-d₂ as a colorless oil (0.0355 g, 95% yield). ^1H NMR (400 MHz, CDCl_3) δ 7.20 – 7.04 (m, 4H), 5.04 (d, J = 6.9 Hz, 1H), 4.76 (d, J = 12.1 Hz, 1H), 4.72 (d, J = 12.1 Hz, 1H), 4.08 (td, J = 8.0, 5.0 Hz, 1H), 3.17 (dd, J = 16.3, 5.1 Hz, 1H), 2.90 (s, 2H), 2.72 (dd, J = 16.3, 8.0 Hz, 1H). ^{13}C NMR (201 MHz, CDCl_3) δ 154.0, 135.4, 133.8, 129.6, 129.0, 126.5, 126.2, 95.8, 74.6, 47.2, 35.8, 28.6 – 27.8 (m), 26.8. HRMS (+APCI) calculated for $\text{C}_{13}\text{H}_{13}\text{D}_2\text{O}_2\text{NCl}_3$ $[\text{M}+\text{H}]^+$ 324.0288, found 324.0293.

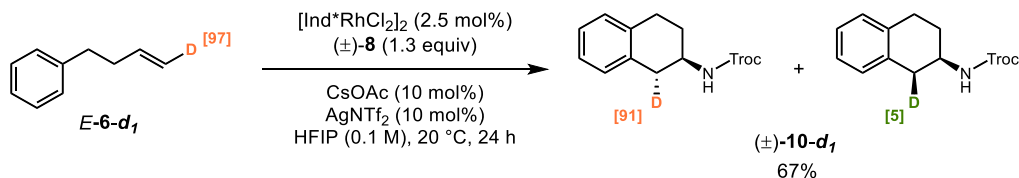

2,2,2-trichloroethyl (1,2,3,4-tetrahydronaphthalen-2-yl-1-d)carbamate ((±)-10-d₁): Prepared using **General Procedure G** using **E-6-d₁**. Purified via preparative TLC using (20% Acetone in Hexanes) to provide (±)-10-d₁ as a colorless oil (0.0227 g, 67% yield). ^1H NMR (400 MHz, CDCl_3) δ 7.23 – 7.06 (m, 4H), 5.08 (d, J = 7.8 Hz, 1H), 4.78 (d, J = 12.1 Hz, 1H), 4.74 (d, J = 12.0 Hz, 1H), 4.10 (qd, J = 8.2, 3.1 Hz, 1H), 2.94 (t, J = 6.1 Hz, 2H), 2.72 (d, J = 8.2 Hz, 1H), 2.14 (dtd, J = 12.2, 6.0, 3.1 Hz, 1H), 1.86 (ddd, J = 15.8, 12.8, 8.1 Hz, 1H). ^{13}C NMR (201 MHz, CDCl_3) δ 154.0, 135.4, 133.7, 129.6, 129.0, 126.5, 126.2, 95.8, 74.6, 47.3, 35.5 (t, J = 19.9 Hz), 28.8, 27.1. HRMS (+APCI) calculated for $\text{C}_{13}\text{H}_{14}\text{DO}_2\text{NCl}_3$ $[\text{M}+\text{H}]^+$ 323.0226, found 323.0218.

19. Computational Details

All calculations were conducted using density functional theory (DFT) implemented in ORCA 4.2.0 with B3LYP including Grimme's D3 dispersion correction.³¹ Geometry optimizations and analytical vibrational frequency calculations were carried out with Ahlrichs's double- ζ def2-SVP basis set for main group atoms and def2-TZVP for Rh.³² Vibrational frequency analysis confirmed that each optimized structure corresponds to either a minimum with no imaginary frequencies or a transition state with one imaginary frequency. Single-point energy calculations were performed at the def2-TZVPP level on the optimized geometries to obtain the gas-phase electronic energies ($E(\text{SCF})$) and the solution-phase electronic energies ($E(\text{sol})$) using the solvation model. The conductor-like polarizable continuum model (CPCM) was employed with a dielectric constant of 16.7 to simulate HFIP.³³ The RIJCOSX approximation was used with the def2/J auxiliary basis set.³⁴ Thermochemical corrections, including the zero-point energy (ZPE), thermal enthalpy ($H(\text{gas})$), and entropy contributions ($S(\text{gas})$), were obtained from gas-phase vibrational frequency calculations. The thermal enthalpy correction ($E_{\text{thermal\_correction}}$) includes translational, rotational, and vibrational contributions at $T = 298.15$ K under the ideal gas approximation. The solution-phase Gibbs free energy ($G(\text{sol})$) was computed using the following expressions.

$$H(\text{gas}) = E(\text{SCF}) + \text{ZPE} + E_{\text{thermal\_correction}}$$

$$G_{\text{solv}} = E(\text{sol}) - E(\text{SCF})$$

$$G(\text{sol}) = H(\text{gas}) - TS(\text{gas}) + G_{\text{solv}}$$

$$\Delta G(\text{sol}) = \Sigma G(\text{sol}) \text{ for products} - \Sigma G(\text{sol}) \text{ for reactants}$$

Additionally, we used the Amsterdam Modeling Suite (AMS) for energy decomposition analysis (EDA), which was performed at the B3LYP-D3/TZ2P level under the scalar relativistic approximation using the zeroth-order regular approximation (ZORA).³⁵ The total bonding energies ($E_{\text{interaction}}$) were computed with the following components: Pauli repulsion (E_{Pauli}), electrostatic interaction (E_{elsta}), orbital interaction (E_{orbital}), and dispersion contributions (E_{disp}).³⁶

$$E_{\text{interaction}} = E_{\text{Pauli}} + E_{\text{elstat}} + E_{\text{orbital}} + E_{\text{disp}}$$

Natural bond orbital (NBO) analysis was performed using NBO 6.0 to evaluate charge distribution.³⁷ Intrinsic bond orbital (IBO) analysis was carried out with the IBOView module to visualize localized bonding patterns, using IBO2015 (exponent 2).³⁸ The buried volume ($\%V_{\text{Buried}}$) was calculated using the SambVca 2.1 web tool with a sphere radius of 3.5 Å and a mesh spacing of 0.1 Å.³⁹ All molecular visualizations and structural representations were generated using CYLview20.⁴⁰

20. Supplementary Calculation

20.1. Analysis of Enantioselective [2+2] Metallacyclization Steps

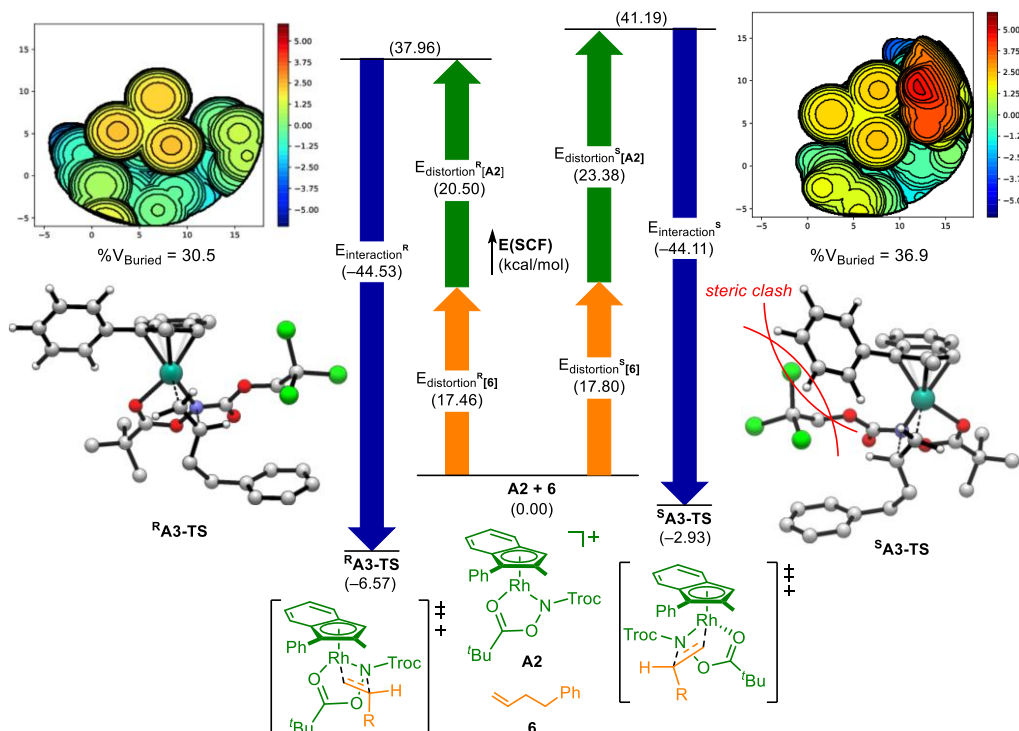

Figure S1. Distortion interaction analysis of $^R\text{A3-TS}$ and $^S\text{A3-TS}$.

Figure S1 shows a distortion–interaction analysis comparing the transition states $^R\text{A3-TS}$ and $^S\text{A3-TS}$. Both pathways originate from the same fragments **A2** and **6**, and the relative activation barriers are decomposed into distortion energies and interaction energies. $^R\text{A3-TS}$ exhibits a slightly lower total distortion energy (38.0 kcal/mol) than $^S\text{A3-TS}$ (41.2 kcal/mol), primarily due to reduced fragment strain. Despite similar interaction energies (−44.5 vs −44.1 kcal/mol), the larger distortion penalty in $^S\text{A3-TS}$ contributes to its higher energy. Notably, the steric clash between the bulky Troc group and the aryl moiety of the ligand in $^S\text{A3-TS}$ increases the distortion of fragment **A2**, as reflected in its higher $E_{\text{distortion}}^S[\text{A2}]$ value (23.4 kcal/mol) than $E_{\text{distortion}}^R[\text{A2}]$ (20.5 kcal/mol). Buried volume ($\%V_{\text{Buried}}$) analysis also indicates a more congested environment in $^S\text{A3-TS}$ (36.9%) compared to $^R\text{A3-TS}$ (30.5%), supporting the observed steric repulsion between the Troc-protecting group and the chiral ligand.

20.2. Analysis of Ligand Effect: Indenyl vs. Cp*

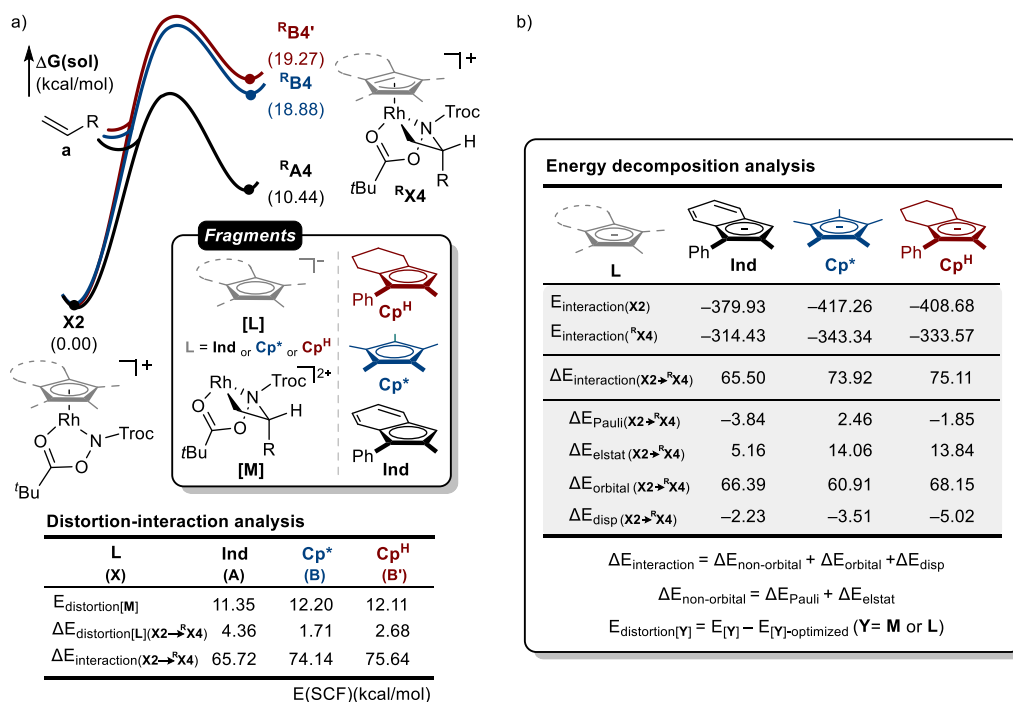

Figure S2. (a) Distortion-interaction analysis and (b) energy decomposition analysis for the haptotropic shift from **X2** and **R4** (**X** = **A** for **IndRh**, **B** for **Cp*Rh**, and **B'** for **Cp^HRh**), performed using ORCA and AMS, respectively.

Relative free energies for intermediate **R4** with three ligands are plotted relative to **X2**. Among the three systems, **R4** is significantly less unstable in **IndRh** (10.4 kcal/mol) than in **Cp*Rh** (18.9 kcal/mol) and **Cp^HRh** (19.3 kcal/mol), highlighting the enhanced thermodynamic favorability of haptotropic rearrangement in the presence of an indenyl ligand.

To investigate the origin of this energetic preference, we performed a distortion–interaction analysis using model fragments **[L]** and **[M]** (Figure S2a). While both the distortion energies for metal fragments ($E_{\text{distortion}}[\text{M}]$) and distortion energy differences of ligand fragments ($\Delta E_{\text{distortion}}[\text{L}](\text{X2} \rightarrow \text{R4})$) are relatively similar across the systems, the interaction energy change associated with the rearrangement from **X2** to **R4** ($\Delta E_{\text{interaction}}(\text{X2} \rightarrow \text{R4})$) is substantially smaller in **Ind** (65.7 kcal/mol) than in **Cp*** (74.1 kcal/mol) and **Cp^H** (75.6 kcal/mol). This indicates that the major contributor to the energetic stabilization of **R4** is the reduced interaction energy penalty during the hapticity shift.

Energy decomposition analysis (EDA) further supports this conclusion (Figure S2b). The absolute interaction energies ($\Delta E_{\text{interaction}}$) are markedly stronger for **Cp*** (–417.3 kcal/mol) and **Cp^H** (–408.7 kcal/mol) compared to **Ind** (–379.9 kcal/mol), reflecting tighter initial metal–ligand (M–L) binding in the Cp-based ligands. However, this stronger initial interaction leads to a greater energetic loss during the haptotropic shift. A detailed breakdown of the EDA components reveals that the primary difference arises from non-orbital terms—most notably, electrostatic interactions. When comparing **Ind** and **Cp^H** (an analog to **Cp*** with steric features similar to **Ind**), the change in electrostatic interaction (ΔE_{elstat}) is 5.1 kcal/mol for **Ind** versus 13.8 kcal/mol for **Cp^H**, indicating a significant difference of 8.7 kcal/mol. In contrast, the change in orbital interaction energy ($\Delta E_{\text{orbital}}$)

is small: 66.4 kcal/mol (**Ind**) vs 68.2 kcal/mol (**Cp^H**). These findings suggest that the more diffuse π -electron density in the indenyl ligand renders it less sensitive to changes in M–L bonding rearrangements. The comparison with **Cp^H** further confirms that the extended π -conjugation in the indenyl ligand plays a decisive role in minimizing the energy cost of the rearrangement. Despite their steric difference, **Cp^HRh** and **Cp^{*}Rh** show nearly identical energetic profiles, reinforcing the conclusion that the energetic advantage of **IndRh** arises primarily from electronic, rather than steric factors.

In conclusion, this analysis supports the notion that the diffuse and asymmetric π -system in the indenyl ligand allows for greater flexibility in M–L coordination, thereby reducing the energetic penalty associated with $\eta^5 \rightarrow \eta^2+\eta^3$ hapticity shifts and contributing to the superior thermodynamic stabilization of key intermediates.

20.3. Effect of *N*-Protecting Group on Chemoselectivity: Tetralin vs. Aziridine Formation

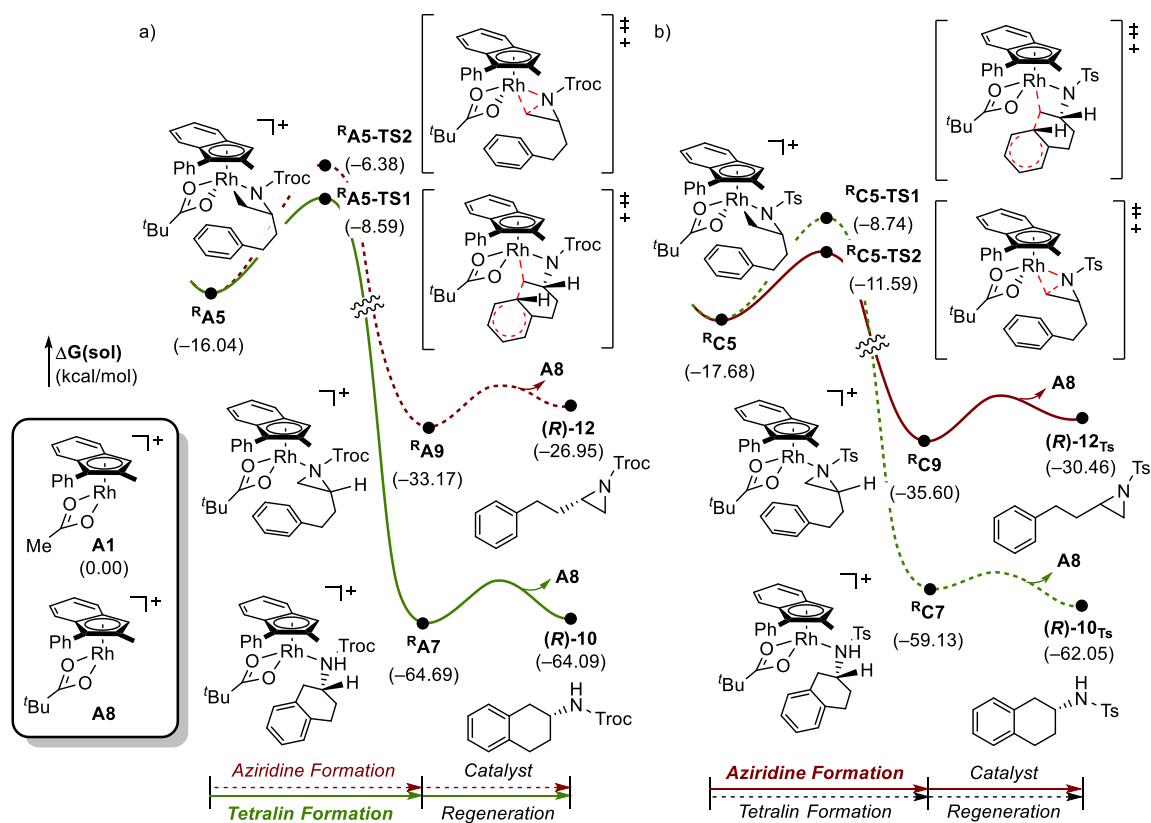

Figure S3. Full energy profiles of tetralin and aziridine formation depending on the *N*-protecting groups. (a) *N*-Troc and (b) *N*-Ts.

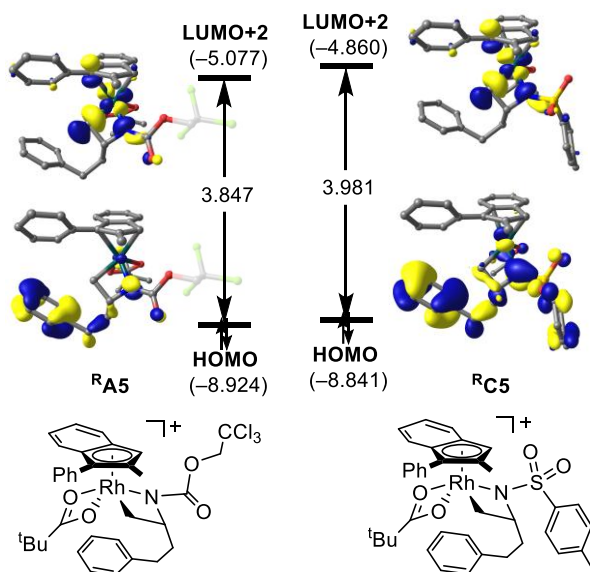

Figure S4. Molecular orbital analysis of **R_X5** (X = A for *N*-Troc, and C for *N*-Ts).

Molecular orbital analysis of **R^X5** reveals the electronic influence of the *N*-protecting groups on tetralin formation (Figure S4). We identified that the π -orbital of the aryl ring and the σ -antibonding orbitals of the Rh–C and Rh–N bonds—primarily localized on the HOMO and LUMO+2 of **R^X5**, respectively—are responsible for the formation of the new C–C bond. While both orbitals involve contributions from the nitrogen atom, the LUMO+2 shows a greater coefficient at the nitrogen than the HOMO, making it more sensitive to the electronic properties of the *N*-protecting group. In **R^X5** bearing more electron-withdrawing Troc group, the LUMO+2 level is 0.22 eV lower than that of **R^C5** with Ts group. In contrast, the HOMO levels are only marginally influenced by the *N*-protecting group, with a small energy difference of 0.08 eV between **R^A5** and **R^C5**. As a result, the HOMO and LUMO+2 levels are closer in **R^A5**, enhancing orbital interaction and thereby facilitating tetralin formation—consistent with the experimentally observed chemoselectivity of the *N*-Troc substrate.

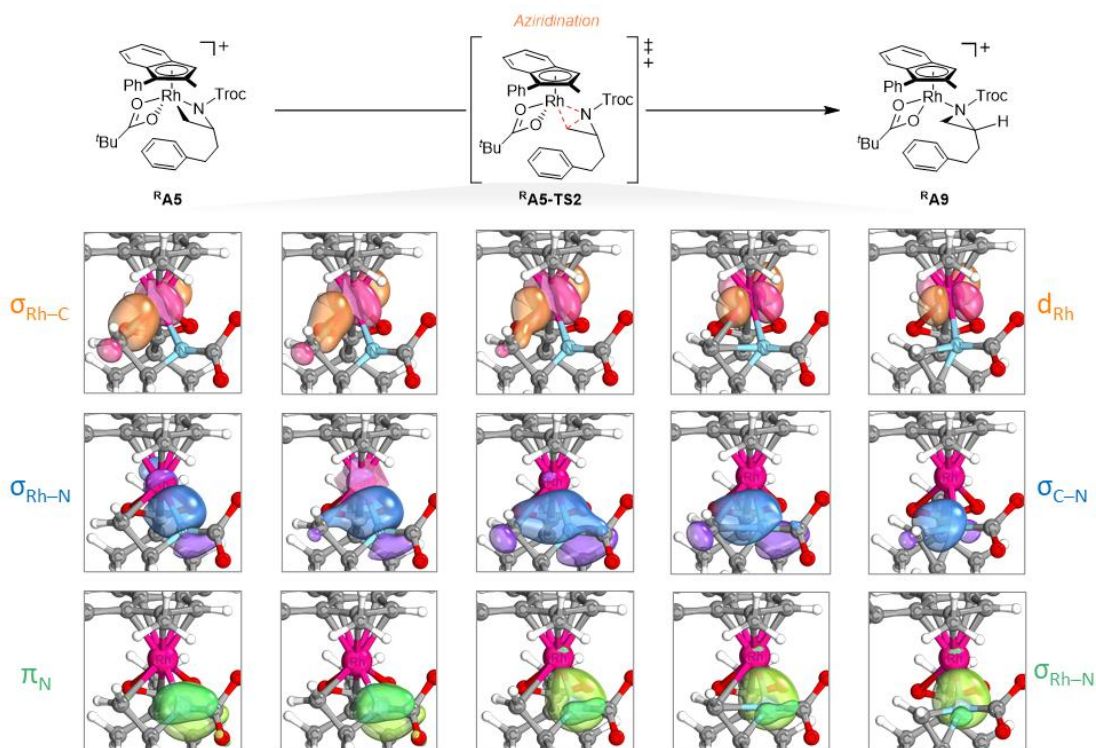

Figure S5. IBO analysis: Electronic flow of aziridination of **R^A5**.

20.4. Substituent Effects on Regioselectivity

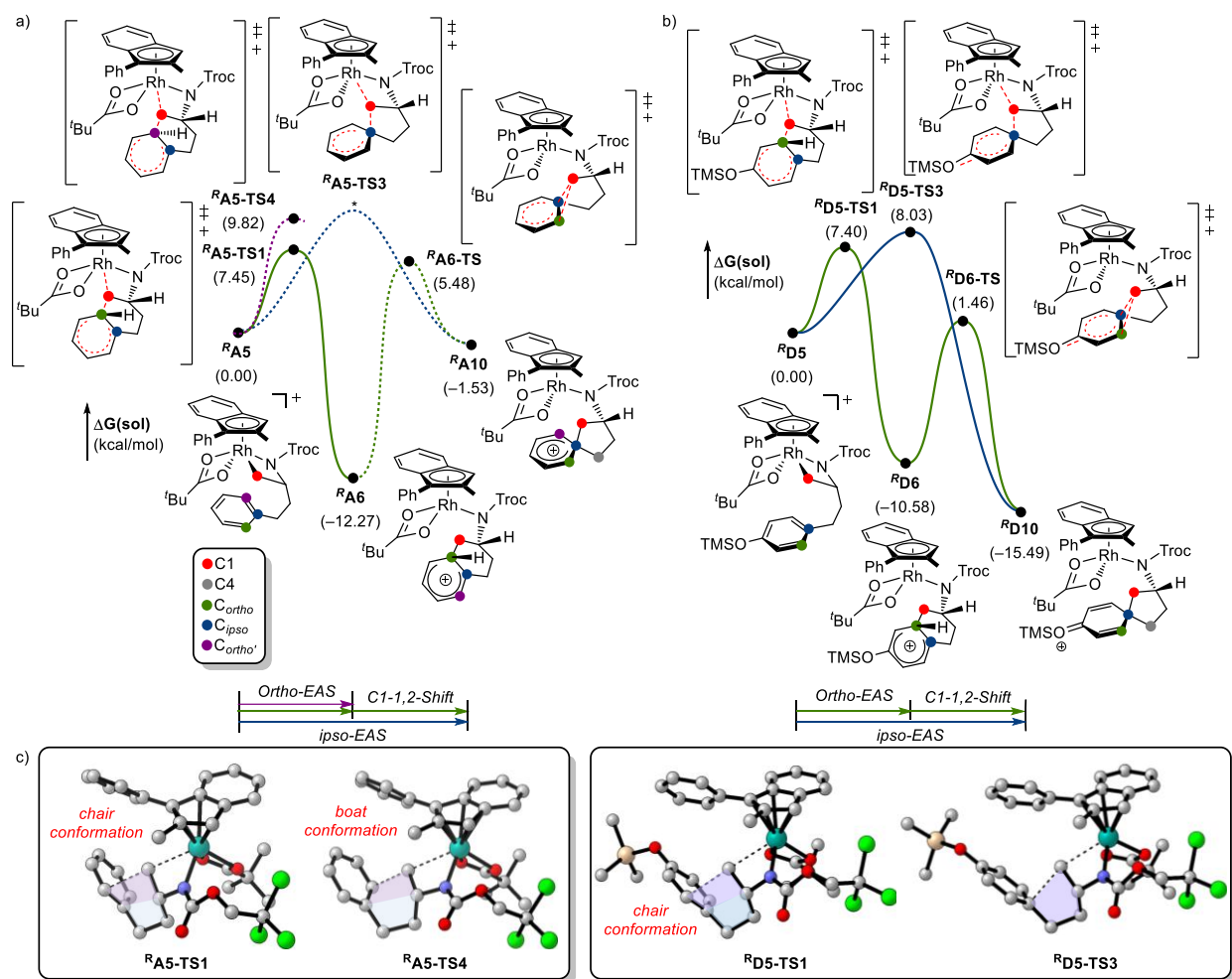

Figure S6. Energy profile electrophilic aromatic substitutions of (a) R^A5 (p-H) and (b) R^D5 (p-OTMS). (c) DFT-optimized structures of key transition states. Hydrogen atoms are omitted for clarity.

Figure S6a depicts the energy profile for competing electrophilic aromatic substitution (EAS) pathways starting from R^A5 . Ortho-EAS preferentially occurs at the C_{ortho} position (green dot) via R^A5-TS1 , which has a barrier of 7.5 kcal/mol, rather than at the alternative $C_{ortho'}$ position (purple dot) through R^A5-TS4 with a barrier of 9.8 kcal/mol. The observed regioselectivity is attributed to the favored chair-like cyclohexene motif in R^A5-TS1 , whereas R^A5-TS4 adopts a less favorable boat-like conformation. Attempts to identify an ipso-EAS pathway were unsuccessful, indicating that the direct ipso attack to C1 is disfavored. In line with this finding, the ortho-arenium R^A6 is thermodynamically more stable than the ipso-arenium R^A10 by 10.7 kcal/mol. Although R^A10 can be generated from R^A6 by C1-1,2-shift via R^A6-TS with an activation barrier of 17.8 kcal/mol relative to R^A6 , the pronounced stability of R^A6 suggests that it is the resting state after the EAS step.

In contrast, the EAS profile starting from R^D5 exhibits a different trend. As shown in Figure S6b, R^D5 is a Rh(V) intermediate bearing a para-OTMS substituent on the aryl ring. C-C bond

formation can occur either at C_{ortho} via R_{D5-TS1} with a barrier of 7.4 kcal/mol or at C_{ipso} (blue dot) through R_{D5-TS3} with a barrier of 8.0 kcal/mol. The small gap of 0.6 kcal/mol indicates that both pathways are kinetically accessible. From a thermodynamic perspective, however, the ipso-arenium R_{D10} is located at -15.5 kcal/mol, which is substantially lower in energy than R_{D6} at -10.6 kcal/mol. As a result, R_{D10} serves as the resting state after EAS step. Consistent with this finding, R_{D6} can rearrange to R_{D10} by a C1-1,2-shift via R_{D6-TS} , requiring a barrier of 12.0 kcal/mol relative to R_{D6} . This rearrangement is easier than the analogous shift from R_{A6} (17.8 kcal/mol) by 5.8 kcal/mol, highlighting the stronger thermodynamic driving force toward R_{D10} .

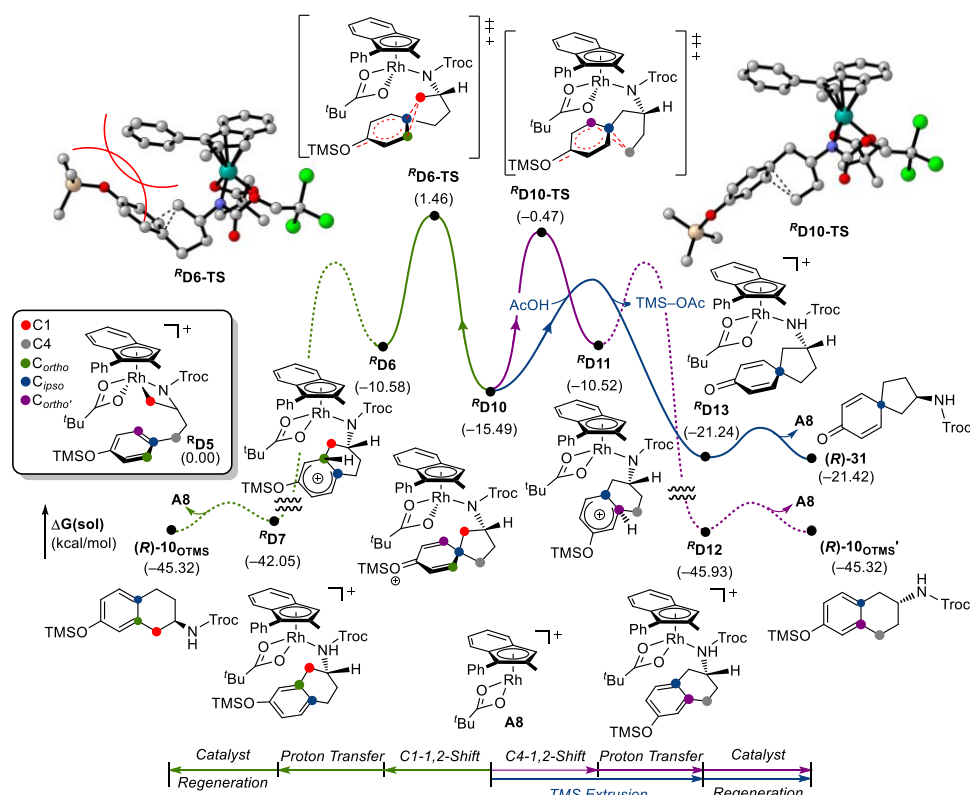

Figure S7. Energy profile from R_{D10} bearing p -OTMS. DFT-optimized structures of key transition states are drawn. Hydrogen atoms are omitted for clarity.

The resulting spirocyclic oxocarbenium intermediate R_{D10} can follow three distinct pathways: 1) C4-1,2-shift, 2) C1-1,2-shift, and 3) TMS extrusion. The C4-1,2-shift proceeds through R_{D10-TS} with a barrier of 15.0 kcal/mol and is favored over the competing C1-1,2-shift through R_{D6-TS} at 17.0 kcal/mol. This preference is consistent with the experimentally observed regioselectivity (see Figure 6). Both transition states adopt chair-like cyclohexene geometries, but the higher barrier in the C1-pathway originates from steric clash between the phenyl substitute on the indenyl ligand and the substrate's phenyl ring.

After the 1,2-shift from R_{D10} , the C4- and C1-pathways furnish R_{D11} and R_{D6} , respectively. Subsequent proton transfer restores aromaticity to give R_{D12} from R_{D11} and R_{D7} from R_{D6} . Then the products $(R)-10_{OTMS'}$ and $(R)-10_{OTMS}$ could be released, giving the active catalyst. This stage is strongly exergonic, with an overall energy release of approximately 35 kcal/mol relative to R_{D5} .

Alternatively, the positive oxocarbenium character of **^RD10 enhances the electrophilicity at the spiro center, promoting TMS extrusion. In this pathway, nucleophilic attack by acetate at the OTMS substituent, followed by proton transfer and release of TSM–OAc, affords the spirocyclic product (**^R31). This transformation is exergonic by 21.4 kcal/mol relative to **^RD5.******

Taken together, proton transfer from **^RD6 and **^RD11 can yield tetralin products, while **^RD10 can undergo TMS extrusion to form a carbospirocyclic product. All of these pathways are expected to be solvent- or acid-assisted and therefore proceed through relatively slow bimolecular processes. However, **^RD6, **^RD10, and **^RD11 are interconverted by rapid intramolecular 1,2-shifts, establishing a equilibrium among the three intermediates. Within this equilibrium, **^RD10 is the most stable species and thus dominates the population, which makes it the most exposed to subsequent bimolecular reactions.**************

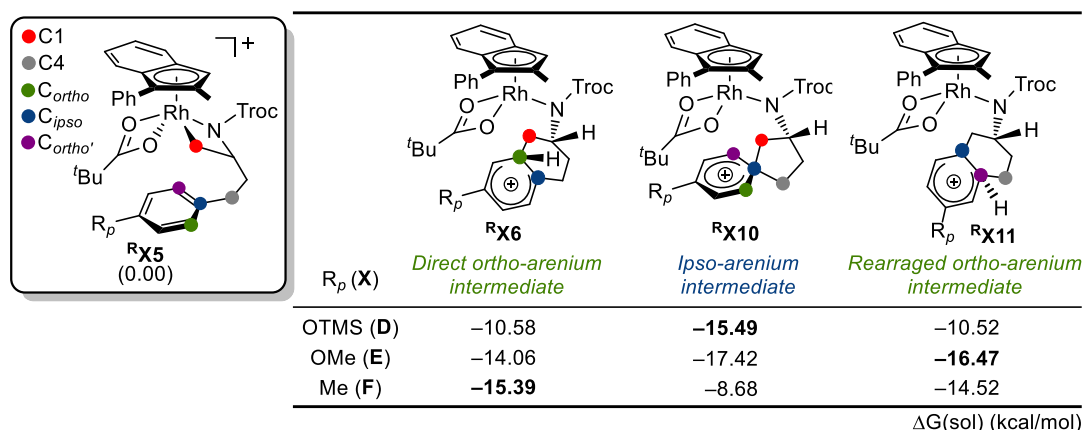

Figure S8. Relative free energies of arenium intermediates **^RX6**, **^RX10**, and **^RX11** (**X** = **D** for OTMS, **E** for OMe, and **F** for Me).

The formation of both the direct and rearranged 2-aminotetralin products is governed by a different mechanistic feature. Figure S8 illustrates the relative energies of the arenium intermediates formed after the electrophilic cyclization step. **^RX6**, **^RX10**, and **^RX11 are connected through C1- and C4-1,2-shift processes, allowing rapid equilibration among these intermediates (see Figure S7 for the corresponding reaction pathways). The computed free energies indicate that the relative stability of these intermediates governs their steady-state populations and, consequently, the distribution of products.**

For substrates bearing *p*-OMe or *p*-OTMS substituents, the ipso-arenium **^RX10 is preferentially stabilized through resonance. In the OTMS case, facile loss of the TMS moiety enables **^RD10 to follow the spirocyclization pathway.****

In contrast, the *p*-OMe substituent has a much poorer leaving group. Although **^RE10 is stabilized by the resonance effect (ΔG = –17.4 kcal/mol, making it the most stable arenium intermediate), it cannot undergo Si–O cleavage as seen in the OTMS case. Instead, **^RE10 interconverts with the ortho-arenium species through 1,2-shift. **^RE11 is calculated to lie 2.4 kcal/mol below **^RE6, an energy difference that originates from steric interactions present in **^RE6 between the indenyl phenyl group and the substrate's *para*-substituent. As a result, the equilibrium is shifted toward **^RE11, but not exclusively; both **^RE6 and **^RE11 remain accessible. Consequently, the reaction****************

furnishes a biased mixture of direct and rearranged products. Calculations and experiments are in good agreement on a ratio of 1:4.

For *p*-Me substrates, the substituent provides weaker stabilization of the ipso-arenium intermediate, making the ortho-arenium species **^RF6** and **^RF11** thermodynamically preferred. **^RF6** is calculated to be only 1.1 kcal/mol more stable than **^RF11**, leading to a mild thermodynamic preference for **^RF6**. However, the small energy difference results in appreciable populations of both intermediates, giving rise to a biased but mixed distribution of products, in agreement with the experimentally observed 3:1 ratio.

Overall, these data show that the arenium intermediates form a rapid equilibrium whose populations are dictated by their relative free energies. This analysis highlights how the relative stability of arenium intermediates governs the distribution of final products.

21. Energy components for optimized structures

Table S4. DFT-optimized structure's energy components

Label	E(SCF)<br>(eV)<br>B3LYP-D3<br>/def2-TZVPP	E(sol)<br>(eV)<br>B3LYP-D3<br>/def2-TZVPP	ZPE<br>(kcal/mol)<br>B3LYP-D3<br>/def2-<br>SVP(def2-<br>TZVP for Rh)	E _{thermal_correction}<br>(kcal/mol)<br>B3LYP-D3<br>/def2-<br>SVP(def2-<br>TZVP for Rh)	S(gas)<br>(kcal/mol)<br>B3LYP-D3<br>/def2-<br>SVP(def2-<br>TZVP for Rh)
Indenyl–Rh(III)	-12433.800		81.89	4.80	87.98
Cyclopentadienyl–Rh(III)	-8246.794		52.54	3.05	73.99
9		-55732.557	129.05	11.34	134.97
7		-33230.091	175.80	11.96	138.19
6		-10562.879	119.07	5.93	94.52
HOAc		-6233.778	38.65	2.89	68.32
HOPIv		-9442.206	91.84	5.12	85.73
TMSOAc		-17353.887	101.51	7.69	105.42
(R)-10		-56856.194	159.22	11.29	136.84
(R)-12		-56854.511	157.75	11.64	138.72
(R)-10 _{TS}		-34353.674	206.18	11.81	137.75
(R)-12 _{TS}		-34352.200	204.59	12.31	142.21
(R)-10 _{OTMS}		-70023.459	224.84	16.92	175.65
(R)-10 _{OTMS} '		-70023.456	224.75	16.97	174.17
(R)-31		-58902.255	161.55	11.89	139.33
A1		-26022.150	182.90	11.84	136.44
A2	-75519.786	-75521.595	273.52	20.46	197.69
[Ind]-A2	-16800.663				
[M]-A2	-58702.611				
^R A3		-86085.055	394.34	26.74	236.59
^R A3-TS	-86082.790	-86084.558	393.88	26.34	232.47
[A2]- ^R A3-TS	-10561.962				
[6]- ^R A3-TS	-75518.897				
^R A4	-86083.138	-86084.882	395.32	26.45	235.18
[Ind]- ^R A4	-16800.474				
[M]- ^R A4	-69269.002				
^R A4-TS		-86084.497	394.04	26.14	231.96
^R A5		-86085.441	394.91	26.44	233.27
^R A5-TS1		-86085.132	394.60	26.06	229.82
^R A5-TS2		-86084.958	393.96	26.56	235.49
^R A5-TS4		-86085.013	394.4	26.11	230.56
^R A6		-86086.024	395.74	26.18	231.23
^R A6-TS		-86085.219	394.82	26.18	230.86
^R A7		-86087.704	397.83	26.06	229.88

**A8**		-29230.601	235.90	14.24	151.43
**^RA9**		-86086.250	396.02	26.41	231.76
**^RA10**		-86085.541	395.55	26.36	232.53
**^SA3**		-86084.973	394.38	26.89	237.30
**^SA3-TS**	-86082.632	-86084.440	393.68	26.33	231.53
**[A2]-^SA3-TS**	-10561.947				
**[6]-^SA3-TS**	-75518.772				
**^SA4**		-86084.945	395.77	26.26	231.76
**^SA4-TS**		-86084.457	394.11	26.17	233.14
**^SA5**		-86085.391	395.55	26.41	232.13
**^RA5-TS4**		-86085.013	394.4	26.11	230.56
**B1**		-19835.136	170.44	11.43	133.56
**B2**	-69332.725	-69334.633	261.68	20.09	193.53
[Cp*]-B2	-10612.064				
**[M]-B2**	-58702.563				
**^RB3**		-79898.052	382.86	26.58	234.68
**^RB3-TS**		-79897.116	382.09	26.33	233.94
**^RB4**	-79895.838	-79897.562	384.02	25.91	230.49
[Cp*]-^RB4	-10611.990				
**[M]-^RB4**	-69268.965				
**^RB4-TS**		-79897.036	382.30	25.85	232.20
**^RB5**		-79898.305	383.40	26.06	230.66
**B2'**	-75585.673	-75587.482	302.15	21.17	201.48
**[Cp^H]-B2'**	-16865.439				
**[M]-B2'**	-58702.596				
**^RB4'**	-86148.650	-86150.374	424.2	27.07	239.31
**[Cp^H]-^RB4'**	-16865.323				
**[M]-^RB4'**	-69268.969				
**^RC5**		-63583.056	442.07	27.19	237.56
**^RC5-TS1**		-63582.681	441.14	26.62	231.46
**^RC6**		-63583.598	443.25	26.82	235.69
**^RC7**		-63585.005	444.33	26.32	230.39
**^RC5-TS2**		-63582.717	440.91	27.26	239.75
**^RC9**		-63583.885	442.63	26.71	233.74
**^RD5**		-99252.802	460.64	32.23	273.18
**^RD5-TS1**		-99252.496	460.17	31.75	268.76
**^RD5-TS3**		-99252.465	459.99	32.03	269.39
**^RD6**		-99253.288	460.95	31.99	271.27
**^RD6-TS**		-99252.782	460.56	31.87	268.29
**^RD7**		-99254.799	463.09	31.52	265.47
**^RD10**		-99253.523	461.47	32.15	271.84

**^RD10-TS**	-99252.802	459.97	31.92	271.44
**^RD11**	-99253.290	461.18	32.05	271.91
**^RD12**	-99254.924	463.11	31.86	270.10
**^RD13**	-88133.688	399.66	26.81	234.55

---

22. Vibrational Frequencies of Optimized Structures

Table S5. Vibrational frequencies (in cm^{-1}) of optimized structures

=====	1398.59 1421.55 1428.96 1448.63 1455.80 1460.51
**Indenyl-Rh(III)**	1462.41 1469.33 1473.98 1477.17 1498.47 1523.85
=====	1627.57 1653.55 1850.81 3024.15 3027.28 3031.40
	3039.04 3093.28 3112.32 3113.09 3120.68 3125.96
	3126.24 3135.09 3135.61 3172.06 3177.69 3208.78
	3210.58 3341.27
=====	=====
107.96 126.20 285.25 346.82 393.28 395.00 449.56	**6**
465.34 532.82 541.23 593.86 684.54 737.12 791.19	=====
848.12 866.74 895.45 929.47 969.24 998.32 1002.53	
1025.59 1043.07 1053.62 1061.14 1140.06 1194.88	
1203.05 1210.13 1270.30 1363.42 1375.78 1405.89	
1436.32 1500.71 1503.13 1546.61 1580.61 3194.10	
3194.73 3197.70 3199.68 3206.31 3208.00 3210.42	
=====	
**Cyclopentadienyl-Rh(III)**	35.20 63.95 91.66 108.51 264.12 287.86 350.15
=====	420.14 445.81 513.31 587.94 633.98 654.01 722.92
	773.79 787.64 832.95 864.02 928.45 943.12 949.13
	988.61 1000.66 1012.55 1024.89 1030.65 1036.59
	1051.51 1085.39 1124.05 1176.85 1196.98 1213.52
	1225.59 1272.63 1296.44 1313.21 1345.97 1363.74
	1370.69 1431.48 1460.48 1476.39 1482.49 1526.96
	1638.05 1661.60 1718.33 3013.42 3029.93 3061.46
	3082.06 3115.89 3126.17 3156.36 3158.61 3170.58
	3180.07 3191.14 3216.92
=====	=====
	**HOAc**
	=====
	46.91 434.33 539.93 596.55 692.16 874.66 1001.52
	1051.05 1213.18 1336.95 1398.91 1441.31 1454.33
	1867.29 3045.21 3129.79 3175.24 3739.41
=====	=====
**9**	**HOPiv**
=====	=====
11.61 19.71 32.29 59.42 71.67 72.44 97.40 174.13	
191.75 206.91 220.53 234.51 243.56 264.93 280.03	
281.70 300.22 328.40 342.84 352.88 373.93 382.90	
394.58 439.00 477.90 532.20 565.46 588.58 637.63	
698.03 738.55 761.33 782.42 792.71 829.79 898.52	
936.66 947.73 954.85 971.92 1037.61 1047.53	
1074.01 1075.92 1098.23 1132.25 1161.40 1226.80	
1251.85 1286.98 1295.94 1350.28 1387.54 1393.72	
1417.54 1421.41 1446.51 1449.98 1458.35 1460.84	
1473.16 1474.64 1477.81 1496.87 1862.81 1871.90	
3030.79 3034.33 3038.72 3067.75 3109.53 3118.77	
3120.68 3134.14 3138.45 3143.10 3143.21 3570.76	
=====	
**7**	
=====	=====
12.39 14.23 21.85 44.15 55.15 66.23 88.50 157.78	
169.97 205.25 220.26 239.61 261.92 282.78 285.44	
296.95 311.68 317.63 337.60 361.62 378.84 392.60	
411.14 422.07 440.64 483.81 515.16 525.10 574.60	
609.75 629.25 644.11 677.02 730.98 765.15 816.05	
819.11 834.17 858.31 880.14 947.80 955.10 963.39	
972.10 987.52 1001.76 1007.63 1026.32 1031.42	
1044.29 1053.55 1053.83 1091.32 1135.99 1138.95	
1154.77 1201.12 1227.07 1233.07 1255.46 1293.67	
1314.98 1340.80 1360.96 1385.35 1392.25 1396.84	
	**TMSOAc**
	=====
	58.92 79.42 98.84 116.96 138.37 155.19 158.62
	185.59 196.61 210.04 246.58 281.03 372.29 584.28
	598.13 633.92 682.81 692.86 695.47 727.18 767.85
	772.39 864.95 867.05 869.29 958.49 1022.19 1050.82
	1265.93 1267.80 1276.17 1297.65 1384.57 1418.54
	1429.24 1429.69 1430.08 1435.66 1437.34 1447.05

1448.87 1816.49 3022.54 3024.34 3026.00 3044.13
3108.90 3110.01 3111.90 3118.25 3121.96 3131.53
3140.68 3176.09

=====

(R)-10

=====

5.28 16.51 27.85 48.20 65.54 73.87 123.06 140.48
163.24 204.61 218.04 248.16 263.58 267.48 287.98
302.10 339.05 391.94 397.95 412.31 447.03 471.51
477.56 492.00 525.06 555.60 571.25 602.18 663.97
689.29 721.82 731.46 751.76 764.22 773.24 806.06
833.45 858.14 895.55 911.10 929.78 980.20 983.28
1019.48 1027.39 1053.58 1067.01 1083.15 1088.31
1103.64 1133.32 1142.55 1156.16 1180.47 1187.38
1203.89 1217.78 1227.89 1245.70 1270.58 1290.94
1305.05 1324.99 1342.87 1356.29 1374.18 1383.87
1391.57 1416.38 1446.13 1450.98 1454.85 1470.95
1485.29 1526.59 1545.80 1633.91 1664.00 1847.60
2985.09 3008.53 3017.23 3059.99 3070.87 3073.12
3073.39 3089.55 3145.02 3156.63 3161.02 3176.00
3190.21 3615.64

=====

(R)-12

=====

14.06 19.34 29.94 41.33 54.30 61.58 87.21 91.93
137.78 173.49 186.98 210.17 237.89 267.65 280.26
300.66 318.79 361.93 382.96 391.49 396.86 421.05
449.59 510.76 561.94 574.14 578.29 633.70 663.59
703.12 723.69 771.41 775.26 786.04 796.14 811.26
837.92 865.78 910.59 930.08 953.81 985.40 1002.28
1008.66 1012.82 1027.37 1039.53 1050.42 1062.57
1068.06 1083.29 1094.60 1119.15 1131.23 1136.96
1176.85 1185.42 1198.18 1212.52 1224.43 1225.19
1257.07 1311.31 1324.00 1344.94 1358.79 1365.56
1369.69 1410.93 1448.49 1454.76 1461.67 1474.41
1483.09 1518.08 1527.39 1638.41 1661.90 1850.01
3031.82 3039.17 3072.22 3076.17 3087.22 3104.07
3109.70 3144.39 3158.05 3159.98 3173.27 3181.01
3193.55 3208.84

=====

(R)-10_{TS}

=====

9.78 22.26 31.03 42.36 65.54 79.75 117.81 145.85
162.58 173.67 234.23 237.15 262.97 277.18 300.31
333.72 353.21 392.31 411.07 422.92 446.53 456.26
460.78 483.98 502.62 516.45 527.10 553.12 577.06
603.10 634.62 645.74 657.69 724.31 729.95 746.32
763.99 812.03 830.65 837.88 849.11 862.11 874.35
896.36 914.90 934.84 982.30 987.60 1002.96 1008.72
1018.82 1027.94 1029.59 1049.42 1065.20 1082.22
1090.19 1104.40 1131.11 1134.89 1137.10 1145.30
1155.67 1179.81 1200.22 1203.16 1216.10 1230.05
1233.74 1256.59 1277.77 1302.49 1314.24 1331.95

1336.70 1353.16 1357.31 1371.46 1375.81 1388.24
1395.81 1425.46 1442.54 1449.76 1450.23 1461.93
1464.45 1474.97 1484.10 1522.60 1526.27 1624.60
1632.74 1652.24 1663.79 3002.86 3004.11 3010.59
3023.19 3056.36 3064.90 3084.34 3094.18 3095.23
3123.16 3155.06 3161.42 3171.29 3171.97 3176.94
3191.29 3202.57 3206.75 3557.70

=====

(R)-12_{TS}

=====

10.22 18.50 29.47 35.73 41.80 42.80 77.11 97.34
141.23 152.58 167.69 202.75 238.87 266.61 285.70
318.98 350.12 364.80 365.36 389.39 419.18 420.25
429.08 490.17 495.16 518.11 537.45 565.60 586.27
634.02 644.17 653.80 675.83 723.56 736.24 770.41
779.27 810.88 817.99 835.96 859.52 864.22 880.99
918.99 935.60 951.44 987.98 998.43 1001.62 1005.86
1006.95 1012.78 1026.60 1028.76 1049.41 1050.53
1051.15 1074.10 1083.20 1091.36 1115.19 1127.96
1133.01 1144.19 1170.76 1176.30 1198.02 1199.01
1207.39 1226.00 1231.70 1244.81 1282.66 1311.95
1323.08 1336.53 1353.41 1358.47 1359.49 1369.34
1395.69 1426.33 1430.11 1462.96 1463.32 1464.31
1469.97 1481.99 1490.81 1524.20 1527.20 1628.54
1638.46 1653.64 1662.10 3022.91 3027.41 3035.80
3075.34 3088.36 3093.60 3102.06 3108.01 3123.66
3154.36 3160.33 3170.99 3172.24 3172.54 3180.03
3192.01 3206.11 3207.58 3208.02

=====

(R)-10_{OTMS}

=====

2.89 13.15 18.67 23.30 43.41 47.89 56.20 80.91
103.77 107.42 128.70 135.30 144.83 146.25 160.09
171.40 181.60 186.90 205.99 215.71 227.37 242.22
252.98 261.46 277.82 283.01 287.46 330.19 341.85
355.68 391.69 406.48 437.21 456.28 473.66 495.15
542.44 563.00 571.67 579.18 593.76 611.39 667.74
677.48 681.31 683.83 684.86 691.19 722.17 726.81
757.65 762.42 769.76 783.27 807.85 820.49 830.92
850.54 860.52 862.05 885.07 894.29 921.36 939.33
972.79 977.17 1002.99 1030.18 1044.63 1081.54
1085.02 1101.02 1132.79 1138.51 1157.61 1180.69
1190.42 1215.18 1224.64 1235.61 1270.35 1270.92
1272.37 1277.78 1289.78 1306.30 1307.20 1329.55
1339.06 1345.87 1371.05 1378.32 1390.65 1407.21
1420.91 1426.67 1428.36 1433.46 1434.35 1444.09
1446.04 1451.72 1454.75 1466.98 1471.31 1538.10
1543.42 1618.75 1672.74 1854.03 2987.59 3000.83
3014.60 3020.39 3021.35 3023.36 3064.96 3067.03
3073.07 3079.00 3091.96 3106.77 3107.86 3111.92
3119.47 3120.23 3123.73 3145.16 3156.72 3176.39
3201.01 3594.66

=====

(R)-10_{OTMS}'

=====

11.62 20.62 21.71 28.21 38.79 47.98 58.61 71.17
89.39 109.44 122.33 136.94 144.08 152.13 154.82
170.53 175.04 186.01 200.91 213.96 227.21 242.93
257.87 264.63 273.43 280.76 283.93 324.00 345.57
373.76 388.88 404.70 414.27 454.91 475.63 495.80
509.47 530.18 563.27 572.21 597.82 619.66 662.95
675.79 685.55 687.71 688.51 694.74 716.25 728.70
760.84 762.21 768.59 777.40 796.69 820.34 831.84
851.46 859.83 860.53 880.81 898.41 912.80 921.62
938.58 975.16 994.25 1039.42 1054.81 1076.99
1081.50 1107.47 1132.90 1151.89 1159.59 1166.09
1183.21 1212.48 1240.35 1259.86 1270.93 1271.86
1277.53 1283.02 1296.99 1311.95 1329.23 1336.33
1347.27 1351.48 1364.37 1380.17 1402.90 1419.89
1420.91 1427.01 1427.64 1433.59 1434.07 1443.74
1447.82 1451.11 1461.01 1467.38 1470.35 1477.15
1539.96 1619.98 1671.79 1854.65 2978.90 3004.80
3021.18 3022.99 3023.38 3025.28 3047.97 3064.52
3070.17 3075.23 3109.10,3109.83 3114.25 3116.26
3122.45 3123.67 3128.40 3141.34 3154.78 3181.73
3191.76 3620.91

=====

(R)-31

=====

17.12 20.13 32.95 43.35 50.39 91.66 105.60 130.03
176.89 190.81 195.39 216.87 234.18 257.78 271.03
283.27 323.55 357.74 368.42 394.93 399.03 434.76
447.62 468.42 488.81 510.86 554.12 566.12 594.83
600.41 625.25 686.96 702.76 727.57 754.86 778.59
782.87 794.75 803.84 813.81 860.63 887.53 910.24
938.45 959.54 979.56 1017.73 1031.68 1037.81
1043.54 1047.77 1057.09 1083.67 1087.05 1096.27
1109.34 1170.31 1177.38 1196.68 1214.13 1230.79
1252.96 1259.81 1287.47 1296.81 1303.51 1321.47
1333.18 1373.14 1386.86 1396.19 1409.13 1415.63
1454.96 1457.11 1462.51 1479.03 1544.82 1667.94
1707.57 1774.06 1835.85 3053.49 3063.49 3068.24
3078.13 3079.05 3120.41 3124.05 3129.48 3142.83
3143.38 3149.99 3187.19 3188.72 3623.67

=====

A1

=====

41.65 54.93 71.04 74.45 89.07 93.60 99.09 123.95
148.66 165.03 199.17 221.79 229.92 234.97 271.19
288.41 325.47 337.08 355.18 410.90 422.65 455.87
472.49 485.12 506.34 509.97 557.47 564.23 609.87
613.24 626.35 656.06 676.56 707.78 716.80 727.56
749.90 779.99 790.15 856.28 876.62 891.27 919.47
928.02 972.29 972.94 990.00 1005.33 1010.34
1014.62 1015.35 1020.12 1032.34 1037.22 1046.43
1047.17 1055.98 1062.00 1114.20 1134.17 1162.43
1182.08 1187.85 1207.58 1212.51 1239.50 1317.99
1324.46 1347.15 1367.26 1373.48 1392.74 1408.07
1409.34 1410.77 1438.52 1446.83 1453.03 1471.28

1482.82 1489.76 1494.06 1520.52 1532.52 1542.22
1589.21 1624.68 1642.04 1651.15 2989.54 3041.15
3077.59 3125.68 3126.53 3169.35 3182.08 3186.51
3193.74 3194.07 3201.35 3203.45 3208.41 3212.39
3221.79 3222.43

=====

A2

=====

2.85 6.94 19.25 25.04 32.85 53.91 57.39 60.57 67.14
77.22 86.57 91.47 109.07 116.76 126.29 148.01
174.03 186.64 202.97 206.16 223.54 227.47 235.49
239.02 244.75 266.21 271.22 276.56 280.21 287.21
290.38 306.59 315.68 333.24 335.51 342.14 353.58
365.51 394.71 403.13 409.27 418.94 427.02 450.45
455.91 479.36 489.09 498.99 503.35 559.19 561.78
565.32 610.50 622.28 627.27 640.40 657.20 668.03
681.54 709.57 722.86 747.92 756.80 767.18 774.26
782.10 791.51 821.66 830.81 856.32 870.31 887.00
922.76 934.05 940.70 945.15 951.77 959.13 965.79
975.74 987.91 1006.37 1010.89 1016.64 1017.90
1027.98 1037.83 1040.39 1043.11 1044.94 1048.12
1052.02 1056.89 1068.89 1087.83 1114.71 1138.18
1165.37 1167.28 1180.33 1187.32 1207.55 1216.31
1219.49 1238.56 1239.41 1251.17 1256.72 1303.46
1320.42 1327.62 1351.25 1368.86 1382.13 1389.97
1390.99 1395.79 1399.86 1409.36 1424.34 1432.22
1447.04 1451.00 1455.97 1459.28 1461.57 1463.04
1473.42 1476.09 1477.73 1482.84 1496.21 1498.98
1516.48 1536.81 1580.92 1609.54 1626.58 1651.60
1655.77 1857.04 3033.58 3035.89 3044.52 3045.07
3081.59 3122.05 3123.28 3127.32 3132.69 3133.22
3136.33 3139.91 3154.27 3180.15 3188.76 3190.19
3194.32 3196.07 3199.78 3203.62 3210.94 3212.45
3217.37 3238.56

=====

^RA3

=====

2.38 13.41 23.54 30.08 31.72 35.21 40.31 46.88 48.55
52.69 62.62 63.03 65.02 74.06 75.24 91.44 102.46
110.95 112.16 133.33 145.89 151.38 159.43 176.41
178.13 185.76 193.27 202.14 207.94 213.06 220.89
241.75 251.17 252.96 264.46 274.46 276.13 276.96
284.04 290.42 310.38 316.63 324.87 327.77 334.40
341.61 350.41 366.41 367.93 391.99 397.67 404.11
406.61 410.00 419.68 421.22 440.76 442.76 446.76
479.21 488.13 492.63 498.31 501.55 526.25 560.99
561.27 564.88 591.36 607.41 617.62 628.34 631.75
633.65 662.09 670.01 678.93 711.45 724.21 727.84
737.74 751.64 765.95 769.29 773.66 781.28 783.34
792.12 794.95 815.27 826.64 851.26 862.92 864.81
874.97 897.94 920.59 923.91 927.44 931.88 940.65
941.53 944.79 958.72 962.32 967.54 984.13 987.33
994.38 1001.46 1002.70 1008.23 1010.16 1012.36
1012.54 1016.84 1018.69 1028.37 1033.22 1037.25
1042.92 1043.62 1044.90 1046.72 1051.19 1051.43
1056.73 1066.43 1082.40 1090.89 1111.22 1123.55

1134.07 1155.91 1164.64 1179.12 1179.74 1184.54
1198.32 1206.64 1207.17 1209.62 1216.66 1227.47
1239.18 1239.95 1243.87 1245.10 1277.16 1291.77
1302.95 1320.76 1326.75 1329.23 1344.99 1351.06
1364.42 1365.21 1367.58 1376.64 1389.19 1391.52
1401.64 1403.91 1405.34 1422.88 1431.01 1433.14
1449.21 1452.63 1457.76 1461.23 1462.82 1464.37
1467.35 1469.91 1472.04 1476.26 1478.43 1479.50
1482.35 1496.11 1501.38 1511.33 1526.97 1540.79
1560.05 1589.19 1629.41 1637.33 1645.49 1650.41
1657.43 1660.46 1846.49 3003.18 3007.50 3013.17
3039.31 3041.31 3065.42 3069.73 3088.24 3089.87
3092.02 3095.23 3113.98 3119.41 3122.30 3126.08
3135.81 3142.52 3148.56 3155.87 3158.96 3159.39
3161.28 3177.81 3178.54 3184.68 3186.08 3187.57
3193.46 3197.75 3198.89 3202.52 3209.33 3210.98
3235.59 3253.20 3259.30

=====

R_{A3-TS}

=====

-205.25 8.51 16.32 20.08 29.22 30.76 35.06 41.04
49.50 54.71 60.93 65.27 70.01 72.82 81.57 99.30
106.29 108.91 114.60 128.59 134.04 153.03 163.95
171.64 177.14 189.59 194.60 202.51 209.28 215.06
226.10 230.01 235.84 243.19 247.89 255.97 269.86
272.82 279.16 285.35 295.51 301.80 302.63 317.33
338.57 348.59 348.97 358.85 364.37 372.96 385.49
394.00 410.00 418.91 424.77 425.92 430.70 445.54
466.13 475.12 483.87 488.90 490.41 503.48 521.28
561.77 563.17 571.38 586.45 603.76 614.38 629.19
629.97 633.59 661.16 674.94 678.63 713.21 716.13
724.98 733.50 745.72 763.69 764.90 766.36 771.18
780.20 785.82 791.40 813.90 825.19 840.96 860.69
870.74 875.52 893.22 923.40 925.56 927.82 933.38
941.38 946.39 950.05 954.26 959.97 964.32 970.58
973.96 985.28 999.89 1010.74 1013.18 1015.40
1016.01 1016.97 1030.04 1032.14 1035.27 1038.13
1040.90 1040.97 1041.56 1046.28 1048.22 1056.43
1057.29 1058.13 1068.46 1092.01 1109.32 1112.21
1131.32 1149.05 1164.05 1169.97 1176.11 1179.44
1182.59 1184.64 1205.05 1206.05 1209.61 1211.28
1218.43 1227.13 1231.47 1243.60 1248.26 1265.94
1274.95 1292.45 1313.89 1315.84 1325.04 1340.15
1343.66 1343.82 1365.03 1365.82 1379.94 1387.57
1388.39 1397.61 1402.05 1403.68 1411.15 1424.94
1429.35 1429.65 1450.71 1451.07 1458.51 1460.17
1466.10 1470.26 1473.97 1474.50 1475.82 1478.13
1479.42 1486.68 1498.47 1499.36 1508.61 1514.09
1531.10 1542.94 1591.40 1629.57 1639.84 1650.79
1658.45 1661.51 1684.23 1829.78 2994.94 3031.43
3038.95 3040.42 3043.65 3044.16 3068.61 3080.84
3091.70 3115.74 3119.34 3122.57 3124.73 3128.18
3131.23 3137.23 3142.55 3150.18 3158.09 3159.46
3169.99 3172.81 3178.85 3182.38 3186.80 3187.47
3188.35 3196.19 3197.18 3197.76 3198.77 3204.90
3208.46 3214.84 3220.32 3244.25

=====

R_{A4}

=====

4.77 13.90 19.98 23.28 27.67 31.16 35.08 40.61 45.91
52.10 53.87 57.51 64.81 69.05 71.20 84.36 90.87
101.48 105.05 132.92 150.92 158.59 165.05 175.50
183.02 200.15 204.10 208.47 218.87 226.37 232.70
236.81 243.36 254.13 263.74 271.28 273.06 278.16
287.42 291.97 302.93 315.93 321.30 339.42 347.23
354.85 361.05 371.59 386.05 403.34 409.03 416.16
420.44 422.41 427.93 452.06 461.37 463.80 486.70
489.10 504.60 509.73 523.14 562.27 566.83 578.67
587.87 605.18 611.21 617.30 629.08 633.23 651.43
661.68 680.13 688.21 714.79 720.71 724.02 724.64
768.35 773.37 774.64 777.66 778.75 784.05 790.94
801.55 805.56 826.87 834.95 854.23 860.57 863.47
870.26 894.16 920.20 922.58 927.34 938.47 941.79
947.97 955.77 961.85 967.53 977.50 987.34 988.56
990.03 1002.42 1009.53 1010.73 1012.73 1013.88
1018.90 1030.87 1031.71 1038.30 1039.85 1041.69
1043.06 1043.96 1050.87 1051.98 1058.44 1071.34
1080.52 1087.76 1100.01 1107.88 1122.17 1124.03
1132.85 1155.30 1158.44 1175.82 1177.76 1179.43
1184.00 1197.70 1203.51 1207.54 1222.11 1224.34
1226.37 1236.58 1247.03 1253.83 1282.84 1292.22
1302.22 1303.45 1309.15 1321.88 1337.15 1346.19
1351.14 1361.49 1363.07 1363.88 1383.25 1385.70
1391.36 1392.84 1394.47 1401.75 1426.99 1428.46
1449.70 1451.05 1451.70 1460.99 1462.55 1465.27
1466.41 1467.01 1470.42 1476.15 1477.72 1479.51
1480.38 1482.32 1497.55 1499.32 1507.09 1526.08
1542.90 1610.20 1631.46 1636.79 1649.45 1658.62
1658.80 1732.75 1878.76 3025.26 3032.52 3037.18
3039.78 3043.02 3045.74 3060.35 3072.78 3091.73
3094.15 3107.17 3113.94 3119.21 3129.25 3131.23
3136.46 3139.52 3145.06 3148.51 3155.95 3156.30
3168.66 3170.69 3176.97 3177.72 3177.76 3182.60
3185.94 3189.36 3191.89 3196.52 3197.03 3198.41
3203.44 3206.04 3212.08

=====

R_{A4-TS}

=====

-82.28 8.81 13.27 14.33 21.55 28.31 37.84 41.20
42.99 49.24 51.97 54.81 66.43 76.57 77.83 84.67
89.92 104.96 108.02 123.74 150.03 162.18 167.06
177.80 183.23 194.82 200.63 202.66 209.47 223.92
234.45 241.35 243.60 247.79 254.61 272.74 275.86
278.18 279.28 288.18 296.94 312.72 318.63 333.75
340.42 344.80 359.94 368.43 386.57 388.69 398.84
408.94 411.39 420.14 424.08 433.73 440.69 447.02
461.52 485.85 488.64 504.76 507.33 546.20 561.38
569.27 569.37 572.71 587.61 613.37 618.75 629.72
633.29 661.60 662.37 676.06 689.42 715.11 724.62
726.30 754.32 767.50 772.36 774.24 776.27 780.60
783.27 787.58 794.41 811.80 822.65 857.51 861.63
867.21 871.74 887.63 889.70 901.82 906.26 925.71
942.66 948.34 949.28 964.27 967.00 973.13 982.61
986.28 988.66 1004.76 1007.92 1012.01 1012.34

1015.89 1019.81 1022.69 1029.60 1033.21 1034.32
1039.64 1041.20 1044.39 1049.19 1052.66 1060.10
1063.98 1079.79 1082.27 1094.21 1113.15 1118.08
1127.54 1129.82 1152.77 1158.07 1174.71 1179.82
1184.78 1198.85 1205.41 1205.69 1209.53 1214.18
1224.61 1231.03 1235.70 1241.57 1244.59 1255.15
1292.04 1303.54 1311.22 1319.12 1321.32 1338.89
1339.84 1342.46 1362.71 1366.51 1368.35 1379.59
1383.03 1384.34 1393.42 1395.79 1404.53 1423.50
1428.56 1440.65 1446.15 1452.30 1456.95 1458.77
1460.05 1462.01 1466.00 1466.54 1470.16 1471.54
1475.78 1477.68 1482.58 1495.88 1506.41 1507.94
1526.74 1529.16 1545.47 1607.19 1630.42 1637.35
1648.67 1657.65 1659.83 1848.47 3010.88 3030.53
3036.53 3037.46 3039.11 3044.87 3071.17 3085.30
3090.73 3104.86 3114.79 3114.86 3117.60 3129.73
3130.05 3132.52 3139.98 3144.21 3148.82 3154.93
3157.34 3170.29 3176.91 3179.78 3180.27 3184.41
3188.30 3193.77 3196.10 3197.60 3199.11 3200.32
3208.03 3209.87 3213.84 3224.85

=====

RA5

=====

6.51 11.35 19.09 34.36 37.57 39.41 42.51 45.35 48.39
53.45 58.42 64.33 76.76 81.47 87.82 92.51 98.71
113.53 121.14 131.94 140.66 164.36 170.05 175.12
180.91 200.24 209.33 213.07 217.60 229.86 241.28
247.19 251.42 258.87 270.42 273.82 277.71 280.24
287.00 296.83 313.94 321.27 321.66 324.60 336.08
348.46 362.44 369.88 380.44 391.36 395.56 400.88
408.49 418.84 423.31 429.92 437.84 456.40 462.81
474.07 491.42 499.18 507.36 530.91 549.50 557.85
560.61 567.77 571.71 614.28 627.76 628.03 632.43
658.70 660.28 666.00 681.21 712.55 723.85 725.88
731.85 761.10 764.33 769.47 774.25 780.14 781.76
792.64 795.60 815.65 825.04 862.73 864.90 868.70
870.90 894.32 906.34 925.52 928.77 931.07 944.51
952.74 955.71 967.29 981.81 983.63 990.69 995.85
1003.37 1004.02 1010.05 1011.68 1011.97 1016.81
1017.70 1028.79 1034.15 1040.84 1041.93 1043.14
1044.67 1048.38 1050.07 1053.47 1055.86 1076.29
1081.21 1089.86 1096.43 1112.01 1124.17 1131.87
1134.65 1171.24 1176.56 1177.24 1181.42 1186.55
1198.61 1203.97 1216.46 1216.80 1218.11 1224.36
1237.74 1241.78 1243.24 1250.72 1296.83 1310.87
1314.60 1322.93 1327.52 1347.52 1348.11 1359.08
1362.96 1366.87 1375.26 1383.93 1392.56 1394.32
1401.22 1406.62 1414.76 1416.16 1420.88 1438.75
1450.46 1452.67 1459.43 1461.54 1463.14 1464.39
1467.14 1469.55 1471.16 1474.11 1476.08 1476.70
1481.17 1481.67 1495.30 1506.74 1517.03 1524.06
1531.56 1538.36 1595.15 1625.22 1633.24 1641.97
1651.96 1657.33 1787.97 3000.17 3008.40 3019.75
3023.13 3036.84 3038.91 3059.05 3075.93 3080.83
3083.69 3089.27 3102.81 3113.13 3116.53 3118.05
3121.18 3122.08 3140.65 3148.18 3158.85 3159.06
3166.61 3173.82 3184.78 3184.97 3189.60 3192.36
3194.15 3197.17 3203.19 3206.21 3213.72 3218.09
3220.82 3248.87 3255.52

=====

RA5-TS1

=====

-236.88 11.06 18.49 23.78 30.10 36.21 36.68 43.91
48.19 49.01 60.99 63.99 81.41 85.29 89.07 94.26
104.51 108.06 112.19 133.29 142.61 148.94 164.54
170.17 180.97 187.71 195.45 206.83 208.47 223.81
236.20 240.71 251.39 255.99 266.46 271.77 272.70
277.72 282.05 287.46 303.69 313.91 321.07 323.76
334.12 348.80 355.57 361.11 385.73 390.97 397.63
400.41 405.75 419.28 420.42 426.15 433.22 444.63
463.37 473.34 481.35 491.86 505.78 513.05 549.06
558.58 562.23 571.41 572.43 614.92 625.79 628.86
629.49 657.59 660.77 665.08 681.99 712.25 730.97
736.13 738.19 762.48 766.52 770.79 779.71 782.24
792.68 794.76 797.94 815.39 817.59 840.83 862.67
876.62 883.45 888.08 906.70 912.57 925.64 927.56
944.29 944.83 952.59 956.14 967.37 981.49 982.16
989.74 990.91 999.47 1007.33 1011.89 1013.19
1017.69 1019.22 1025.18 1034.95 1036.72 1040.40
1042.50 1042.78 1044.76 1048.82 1050.03 1054.46
1058.07 1072.57 1083.04 1107.75 1110.12 1116.30
1133.74 1135.07 1167.74 1178.86 1182.77 1184.32
1189.36 1195.68 1200.93 1204.35 1213.36 1216.33
1223.59 1238.81 1242.84 1244.87 1251.98 1301.77
1316.04 1321.80 1322.80 1329.22 1345.67 1347.78
1350.39 1363.59 1363.75 1379.00 1384.38 1389.93
1394.81 1399.03 1405.56 1415.28 1416.61 1427.31
1437.64 1450.99 1452.20 1459.46 1460.64 1462.32
1463.43 1467.62 1471.78 1472.25 1473.75 1475.65
1477.88 1478.94 1480.54 1494.55 1511.15 1514.93
1522.76 1537.56 1548.05 1592.77 1616.63 1628.57
1647.57 1648.86 1656.30 1778.67 2997.56 3000.63
3006.90 3008.17 3038.54 3050.16 3068.76 3078.36
3078.49 3081.35 3089.90 3106.36 3109.87 3112.53
3116.50 3120.62 3131.11 3150.80 3160.14 3175.80
3178.40 3179.06 3181.80 3185.99 3187.77 3187.91
3194.65 3195.72 3199.06 3202.70 3205.15 3208.56
3214.42 3217.05 3255.11 3321.40

=====

RA5-TS2

=====

-268.70 7.34 9.97 14.34 20.80 26.74 34.99 35.75
40.24 53.40 55.80 58.72 72.19 77.20 79.63 90.16
98.39 99.02 112.09 118.20 138.58 142.57 156.65
161.73 172.46 181.76 184.85 200.97 205.08 207.81
222.39 228.63 234.18 240.99 248.63 257.68 260.39
275.78 280.33 282.10 290.34 294.32 300.96 320.67
326.14 331.30 344.10 353.25 366.68 381.34 383.64
397.01 398.32 408.16 417.80 423.31 429.51 446.91
456.87 473.10 482.15 489.80 500.21 504.42 540.83
557.89 561.86 567.31 570.39 612.77 622.62 628.02
629.68 632.66 660.55 664.43 681.74 710.67 711.76
725.88 732.39 743.46 757.28 768.05 773.40 780.83
782.50 791.01 797.19 818.32 821.72 844.75 862.57
867.92 879.62 885.98 913.07 916.79 924.48 926.94
936.65 946.68 953.93 955.84 963.29 970.71 974.20

989.32 1000.63 1004.46 1006.14 1011.71 1012.43
 1013.98 1014.70 1023.31 1028.43 1031.98 1034.40
 1040.41 1041.79 1042.82 1044.32 1046.43 1050.18
 1057.88 1067.49 1080.73 1085.25 1110.91 1120.98
 1138.17 1146.74 1169.28 1179.57 1180.06 1185.42
 1194.67 1199.03 1205.65 1208.46 1213.24 1222.91
 1223.62 1241.19 1243.71 1248.57 1252.01 1253.50
 1297.02 1306.35 1322.04 1330.84 1333.66 1348.30
 1350.59 1361.76 1366.11 1375.09 1382.50 1387.12
 1391.76 1392.40 1408.71 1411.19 1414.62 1432.97
 1449.37 1452.39 1459.63 1460.27 1460.83 1462.22
 1464.69 1469.65 1470.92 1472.20 1474.48 1475.76
 1476.11 1481.88 1483.81 1494.72 1509.59 1513.23
 1523.88 1538.07 1553.84 1584.17 1628.69 1632.01
 1652.64 1654.76 1656.82 1788.56 3027.98 3031.37
 3036.02 3038.77 3041.56 3067.72 3079.70 3083.52
 3088.62 3111.99 3115.84 3117.70 3118.70 3124.17
 3133.08 3136.97 3145.73 3156.77 3160.91 3165.98
 3168.94 3173.59 3175.60 3178.72 3181.14 3188.14
 3191.13 3191.74 3197.71 3200.67 3202.01 3216.96
 3218.28 3220.48 3283.38 3308.54

RA5-TS4

-226.35 7.63 16.46 24.77 29.42 31.84 40.41 43.33
 51.06 57.14 59.61 62.64 74.61 84.59 92.64 96.64
 98.73 103.63 113.37 130.86 143.40 154.26 158.01
 170.26 181.75 187.40 202.28 206.88 213.79 218.80
 230.26 240.05 247.58 255.01 258.82 270.59 274.80
 279.47 284.21 291.58 308.79 314.28 317.30 322.08
 326.45 332.81 348.72 354.06 379.55 388.20 395.85
 401.17 403.32 418.52 420.77 427.67 438.74 441.68
 467.27 474.09 479.26 490.60 500.95 508.10 547.35
 558.46 565.32 572.12 578.16 614.21 625.97 626.69
 628.79 660.34 662.44 666.83 687.47 710.66 722.09
 732.16 747.96 761.83 762.16 767.80 777.29 782.44
 793.97 794.62 814.41 815.74 822.23 844.16 861.42
 877.50 882.31 886.30 897.67 919.02 925.46 925.81
 933.50 944.41 955.27 956.78 967.71 981.42 986.28
 987.56 997.41 998.06 1002.09 1010.91 1014.75
 1015.61 1019.23 1033.94 1035.55 1038.60 1039.48
 1042.01 1042.19 1043.55 1048.36 1049.51 1056.53
 1057.52 1067.18 1081.46 1092.00 1109.96 1118.07
 1134.86 1135.60 1166.58 1178.29 1183.68 1185.70
 1188.06 1197.37 1198.13 1205.22 1212.32 1216.10
 1219.13 1230.78 1242.32 1245.51 1251.69 1301.73
 1309.07 1317.76 1321.07 1333.14 1337.89 1345.30
 1354.47 1359.99 1362.65 1383.51 1384.65 1389.71
 1395.01 1399.28 1405.02 1410.47 1417.19 1428.48
 1436.72 1450.93 1451.60 1454.70 1460.53 1460.82
 1462.74 1466.58 1470.13 1472.01 1473.80 1474.84
 1477.86 1478.80 1479.98 1494.37 1510.49 1516.02
 1519.11 1535.88 1542.43 1591.05 1612.76 1626.79
 1644.07 1650.50 1655.26 1769.50 2997.86 3004.10
 3011.85 3014.02 3039.49 3045.58 3051.54 3074.59
 3078.66 3083.50 3095.70 3106.57 3108.75 3113.06
 3118.50 3118.59 3147.47 3148.29 3152.76 3174.69
 3175.46 3180.51 3182.32 3182.78 3188.55 3189.83

3194.82 3196.91 3199.77 3204.14 3204.35 3213.61
 3216.15 3219.36 3254.67 3318.72

RA6

11.24 17.47 20.76 24.06 30.74 31.79 41.44 44.81
 45.89 53.30 70.05 80.23 83.45 92.71 99.37 103.13
 109.54 119.68 143.24 147.94 153.75 161.71 164.71
 176.18 185.50 202.09 209.96 212.27 222.29 227.83
 234.20 239.19 242.78 257.62 260.67 270.17 276.30
 282.64 283.45 287.51 299.02 313.12 327.07 332.66
 344.71 349.33 355.03 375.89 392.35 394.93 398.52
 411.53 418.83 428.92 431.56 432.51 447.51 459.45
 472.80 482.76 490.45 506.88 512.03 545.84 551.53
 560.61 564.73 574.05 574.96 613.96 626.16 629.08
 660.55 664.09 667.41 683.83 710.64 716.57 733.84
 737.80 756.80 762.85 774.27 777.68 782.45 794.23
 796.52 802.35 814.75 822.44 860.10 863.57 871.95
 882.84 890.02 894.44 907.03 924.96 925.34 932.41
 946.70 955.80 967.70 971.42 973.84 987.47 993.56
 998.16 999.93 1010.55 1015.46 1022.19 1023.57
 1032.28 1034.75 1037.02 1038.53 1042.23 1042.93
 1046.15 1050.53 1056.88 1057.51 1066.47 1071.46
 1080.35 1092.78 1108.99 1123.10 1129.53 1134.47
 1153.69 1162.11 1174.63 1177.20 1181.27 1183.11
 1194.10 1206.78 1210.50 1212.59 1222.89 1232.11
 1242.88 1251.54 1255.45 1258.10 1273.55 1302.16
 1303.42 1318.51 1322.32 1331.40 1343.05 1345.03
 1356.44 1362.31 1375.50 1381.20 1386.30 1390.60
 1400.81 1404.72 1409.05 1413.39 1423.47 1429.42
 1438.39 1448.74 1450.67 1453.36 1456.00 1457.39
 1460.62 1463.66 1465.48 1467.88 1470.86 1474.03
 1475.79 1480.06 1488.63 1495.16 1496.82 1510.42
 1514.35 1538.96 1561.39 1565.94 1590.50 1624.95
 1654.35 1654.78 1656.75 1762.00 2896.13 2961.76
 3001.89 3024.40 3027.96 3035.98 3037.93 3065.73
 3072.47 3102.78 3104.20 3106.71 3111.57 3114.60
 3120.32 3121.36 3127.04 3143.74 3144.30 3145.63
 3153.14 3170.33 3172.59 3182.74 3183.47 3186.02
 3190.13 3190.47 3196.35 3197.76 3199.54 3202.95
 3211.42 3211.81 3214.15 3259.83

RA6-TS

-196.43 13.53 18.12 23.45 26.26 31.35 36.69 39.41
 42.35 44.68 52.16 62.45 82.07 83.94 96.92 100.12
 106.27 109.77 114.26 128.85 145.11 154.04 159.35
 167.86 179.74 183.85 197.81 207.63 213.35 216.31
 230.91 234.08 242.06 257.23 259.79 266.32 270.03
 277.73 281.10 283.91 286.27 301.21 306.43 322.34
 328.27 347.75 350.24 371.88 378.69 384.60 389.59
 400.38 403.19 411.90 420.04 428.46 431.86 448.96
 460.41 471.51 474.14 483.91 491.21 511.11 544.21
 560.34 564.20 573.46 593.35 614.81 620.18 624.53
 630.01 661.25 665.41 670.38 680.06 686.13 710.97
 731.77 736.66 751.09 762.51 767.78 775.83 777.13

792.56 797.13 812.96 821.03 840.49 849.64 861.68
 877.17 879.07 884.24 897.97 918.31 924.22 925.36
 946.95 951.93 954.75 967.09 973.11 980.21 989.01
 990.30 996.10 997.44 1008.88 1011.83 1018.91
 1021.18 1025.45 1034.43 1036.94 1039.12 1039.16
 1042.11 1045.27 1045.95 1050.83 1051.27 1059.75
 1066.13 1081.53 1101.33 1109.49 1116.62 1127.93
 1134.63 1158.50 1164.25 1173.73 1178.40 1182.21
 1189.07 1194.45 1199.73 1205.89 1211.19 1211.50
 1223.68 1231.00 1242.87 1250.07 1253.83 1301.80
 1316.84 1317.19 1321.96 1322.99 1328.87 1345.21
 1351.95 1355.01 1362.68 1381.58 1387.49 1389.25
 1397.37 1404.36 1407.74 1410.26 1412.47 1428.78
 1437.89 1449.05 1454.08 1456.15 1459.14 1460.87
 1463.49 1464.90 1466.90 1471.41 1472.72 1474.72
 1476.15 1479.54 1481.46 1496.10 1496.54 1510.97
 1512.16 1540.13 1569.83 1591.36 1602.64 1627.94
 1651.95 1653.76 1657.88 1767.58 3020.36 3024.29
 3026.74 3033.80 3038.22 3059.81 3078.51 3090.30
 3104.36 3111.58 3113.05 3113.32 3122.09 3124.33
 3127.21 3142.80 3150.31 3154.45 3161.75 3167.66
 3173.03 3181.97 3185.54 3187.72 3189.75 3192.14
 3197.03 3198.07 3200.22 3207.26 3209.33 3211.44
 3214.60 3214.62 3258.75 3285.95

=====

RA7

=====

16.44 19.46 25.15 25.41 33.59 35.38 45.68 49.53
 54.70 58.89 64.58 71.63 80.76 86.39 98.98 106.02
 115.13 126.77 130.96 143.59 152.59 153.20 162.83
 176.77 183.15 193.13 207.04 210.56 220.91 226.70
 230.90 241.75 251.46 262.36 263.66 270.80 271.41
 279.06 288.97 291.47 299.94 316.24 326.41 329.01
 333.17 351.06 369.90 392.05 392.78 394.96 406.01
 417.80 425.51 429.64 442.01 444.13 448.44 474.83
 483.06 491.06 502.66 514.15 529.14 543.12 558.04
 566.59 570.27 577.65 600.52 610.78 626.04 628.29
 642.50 659.31 667.63 681.86 710.02 722.24 730.12
 733.27 755.25 759.25 767.56 779.05 791.00 793.01
 798.98 813.71 823.32 838.99 856.17 858.21 876.54
 882.96 899.28 901.28 915.92 924.54 925.10 929.65
 946.90 948.74 957.59 971.64 973.85 985.18 989.45
 1003.56 1011.35 1011.47 1019.22 1022.83 1028.23
 1029.02 1032.88 1040.24 1040.96 1043.68 1044.49
 1047.50 1056.67 1060.49 1068.04 1072.56 1077.40
 1104.13 1115.70 1128.50 1138.32 1138.93 1145.68
 1163.19 1168.23 1181.01 1183.89 1186.21 1207.92
 1209.07 1214.70 1215.50 1220.65 1224.15 1242.63
 1248.18 1251.15 1254.76 1274.31 1290.52 1313.13
 1323.22 1325.56 1334.79 1337.66 1349.15 1362.05
 1368.08 1378.23 1381.36 1382.36 1390.92 1392.91
 1393.60 1403.92 1407.21 1414.94 1431.58 1445.85
 1448.98 1450.55 1454.84 1456.62 1458.82 1459.00
 1461.48 1470.03 1471.80 1474.21 1475.41 1476.52
 1479.66 1485.12 1487.85 1497.39 1509.77 1518.52
 1530.47 1530.72 1542.08 1579.46 1628.12 1636.88
 1654.99 1657.88 1665.17 1868.43 3014.23 3018.65
 3022.24 3027.73 3035.02 3036.26 3039.62 3077.59
 3083.45 3094.41 3116.76 3117.97 3118.18 3120.90

3122.54 3127.34 3134.35 3137.72 3142.18 3161.74
 3166.87 3170.82 3177.39 3182.66 3190.45 3190.85
 3191.88 3197.99 3199.22 3201.52 3204.21 3208.88
 3216.09 3220.35 3238.59 3463.74

=====

A8

=====

37.41 42.36 59.55 71.02 76.80 88.92 92.09 101.54
 110.73 132.19 157.01 170.36 218.64 230.37 237.52
 241.27 262.36 270.91 282.11 309.53 316.87 324.40
 338.52 348.19 394.31 402.99 423.40 433.04 447.39
 461.30 475.41 487.19 507.34 550.74 557.53 562.77
 610.11 626.64 636.84 656.56 677.11 708.11 728.13
 749.73 779.67 790.60 791.50 817.25 855.87 876.58
 890.12 918.76 923.80 927.38 943.84 957.63 971.96
 981.92 990.67 1004.75 1011.14 1013.78 1019.38
 1032.34 1036.59 1038.53 1045.22 1046.51 1047.84
 1056.62 1114.29 1134.57 1162.31 1182.27 1187.04
 1208.31 1209.70 1212.88 1238.72 1239.77 1247.96
 1318.71 1325.59 1348.10 1368.02 1384.40 1392.06
 1395.28 1406.93 1410.22 1416.05 1446.59 1450.96
 1452.82 1454.09 1461.63 1466.98 1471.55 1475.10
 1476.74 1481.27 1484.68 1493.10 1521.33 1525.14
 1533.70 1588.41 1625.53 1643.17 1652.24 3002.51
 3010.16 3014.21 3040.38 3084.59 3093.25 3095.18
 3112.92 3117.21 3119.74 3125.03 3171.61 3182.59
 3187.71 3193.26 3196.20 3202.58 3203.97 3208.35
 3212.06 3222.00 3224.07

=====

RA9

=====

18.31 21.56 23.63 31.56 35.37 40.85 43.98 49.31
 52.50 62.15 62.45 72.82 77.84 84.71 87.90 89.82
 104.29 111.51 119.17 126.28 147.95 158.68 166.19
 176.23 189.13 191.70 197.81 200.11 206.94 215.72
 222.95 239.78 243.04 247.95 261.48 269.50 275.95
 286.21 291.21 294.73 295.84 303.50 322.39 329.16
 331.51 349.03 352.32 363.80 390.46 395.07 397.66
 399.79 414.14 421.36 423.32 425.80 435.56 450.24
 475.73 483.50 487.37 501.61 510.99 543.44 559.56
 560.33 566.26 574.55 591.39 611.66 625.68 628.12
 634.09 660.17 671.04 676.85 699.52 710.47 726.44
 730.90 756.40 772.89 778.79 779.40 790.86 793.02
 797.10 804.79 823.42 824.30 855.09 861.47 870.21
 877.32 882.81 897.00 904.65 925.42 926.14 927.81
 938.26 947.56 954.90 957.47 972.09 974.63 986.64
 987.37 1002.06 1006.80 1012.07 1013.44 1017.76
 1019.41 1021.67 1027.66 1033.88 1039.67 1042.47
 1043.77 1045.51 1047.96 1051.68 1059.02 1060.07
 1067.14 1076.85 1100.43 1108.31 1110.98 1134.24
 1135.98 1153.77 1161.44 1178.94 1179.58 1184.96
 1192.73 1200.71 1207.25 1209.72 1210.91 1223.67
 1223.76 1237.83 1243.44 1250.33 1256.13 1266.40
 1292.34 1319.05 1323.03 1328.79 1332.93 1345.97
 1353.99 1360.85 1365.89 1371.81 1382.12 1390.35
 1392.00 1404.08 1405.56 1415.29 1433.09 1434.45

1448.95 1455.47 1456.34 1458.62 1459.33 1462.21
 1469.62 1472.52 1472.82 1475.16 1476.16 1477.63
 1483.08 1484.09 1495.78 1499.27 1509.51 1520.39
 1527.14 1537.51 1547.88 1580.59 1628.67 1637.51
 1654.48 1656.75 1660.19 1843.57 3028.40 3032.45
 3038.55 3041.40 3041.43 3071.06 3088.18 3093.83
 3113.58 3118.05 3122.24 3123.05 3124.71 3132.83
 3135.46 3142.87 3147.56 3149.96 3163.11 3167.80
 3170.36 3175.00 3177.15 3181.20 3183.70 3188.80
 3191.48 3191.67 3197.77 3199.74 3200.57 3203.38
 3213.25 3216.02 3238.96 3256.83

=====

^RA10

=====

12.29 16.06 18.75 22.96 28.79 32.50 39.32 40.30
 44.38 48.63 58.30 72.66 83.74 84.53 88.88 101.68
 108.11 111.94 118.51 144.28 154.55 159.99 166.83
 175.35 185.17 205.85 208.54 212.62 223.73 233.61
 237.91 245.24 249.47 260.31 263.05 266.89 280.26
 283.05 284.85 289.69 308.46 314.25 322.25 328.64
 347.66 349.99 354.87 368.41 382.57 385.38 397.11
 399.26 418.59 421.79 427.59 430.19 433.62 448.13
 471.36 478.26 490.89 502.15 510.77 543.56 561.21
 565.28 572.00 573.80 600.79 614.70 621.78 623.50
 630.07 661.03 665.72 676.55 686.15 710.86 711.40
 732.90 736.84 762.63 770.05 776.77 780.46 791.19
 797.71 810.04 821.49 835.23 851.76 861.94 871.19
 877.08 883.62 892.00 899.28 901.09 923.37 925.59
 946.80 954.40 960.29 967.12 972.02 976.03 989.63
 993.80 994.97 1012.08 1017.70 1020.75 1021.49
 1031.22 1034.11 1034.34 1036.64 1038.42 1041.75
 1046.00 1046.68 1051.31 1052.66 1059.97 1066.80
 1069.43 1074.49 1082.29 1109.03 1110.57 1126.90
 1133.01 1134.01 1164.28 1176.80 1180.64 1181.97
 1189.54 1197.54 1205.03 1211.19 1215.35 1219.87
 1225.01 1243.11 1248.67 1255.03 1275.81 1290.78
 1303.30 1311.01 1316.83 1321.34 1324.26 1345.21
 1346.43 1349.95 1363.27 1381.93 1387.30 1388.87
 1399.63 1406.79 1410.92 1413.28 1423.24 1428.39
 1449.73 1453.67 1454.73 1459.10 1460.70 1460.79
 1462.90 1466.15 1467.63 1470.11 1471.64 1475.66
 1475.85 1479.48 1481.63 1487.32 1496.60 1510.74
 1512.11 1541.00 1572.86 1581.31 1592.57 1629.64
 1644.87 1653.66 1658.62 1758.48 3021.77 3025.61
 3025.64 3032.81 3036.76 3069.09 3077.19 3084.51
 3092.71 3102.36 3109.08 3111.16 3120.57 3122.65
 3127.23 3136.67 3143.30 3148.77 3151.18 3161.17
 3169.67 3178.35 3181.70 3182.26 3184.57 3187.35
 3187.61 3196.32 3196.58 3205.85 3206.66 3207.98
 3210.07 3210.63 3213.08 3257.59

=====

^SA3

=====

10.60 10.83 17.68 22.81 25.75 30.31 36.29 38.53
 41.99 47.47 60.47 71.42 78.15 81.97 91.25 93.98
 96.65 110.88 124.02 135.72 141.64 148.96 153.60

167.50 174.57 178.55 185.58 198.97 206.56 212.80
 218.52 233.42 237.56 242.49 251.69 270.62 271.77
 274.50 283.84 287.77 293.37 308.10 317.76 318.29
 339.66 343.60 346.51 367.75 373.30 384.28 392.68
 398.49 405.57 414.59 416.68 419.31 437.40 439.43
 446.08 479.38 484.29 490.05 498.01 501.17 518.75
 558.69 566.56 570.15 591.11 609.85 615.17 628.92
 632.25 633.57 654.89 663.73 686.21 713.97 719.75
 724.77 742.88 762.71 766.89 771.08 772.16 781.51
 786.14 788.44 796.36 808.46 823.85 852.38 857.95
 863.64 866.26 892.46 907.62 923.59 923.72 924.68
 938.01 942.94 946.05 954.14 955.56 971.39 975.20
 984.39 992.03 1001.83 1003.89 1006.43 1009.25
 1012.59 1014.88 1018.59 1021.08 1032.02 1033.59
 1035.81 1037.75 1039.06 1042.30 1045.94 1051.41
 1052.68 1056.67 1061.00 1084.72 1091.21 1109.82
 1125.29 1136.72 1152.11 1160.28 1179.16 1180.36
 1183.59 1198.43 1202.08 1208.22 1216.91 1224.18
 1227.82 1238.32 1240.20 1247.99 1257.41 1280.08
 1284.13 1318.67 1324.34 1325.01 1335.34 1351.94
 1356.30 1363.65 1364.10 1370.18 1383.69 1388.18
 1392.81 1396.70 1407.71 1413.74 1425.17 1428.41
 1431.14 1443.14 1450.65 1450.79 1454.10 1457.72
 1460.20 1461.53 1470.40 1473.05 1475.60 1477.06
 1482.47 1486.08 1491.63 1498.24 1514.62 1527.15
 1539.20 1567.64 1588.12 1630.98 1637.79 1640.13
 1650.62 1659.34 1660.01 1837.20 3029.93 3031.75
 3040.22 3042.13 3045.15 3076.70 3079.90 3092.48
 3116.80 3118.16 3122.44 3129.90 3131.80 3134.61
 3135.41 3138.67 3152.30 3154.63 3155.65 3156.47
 3162.18 3165.97 3177.64 3178.80 3184.10 3187.56
 3188.62 3192.69 3198.85 3199.81 3203.56 3209.34
 3220.45 3221.52 3227.32 3254.77

=====

^SA3-TS

=====

-209.56 11.02 18.57 23.15 30.08 36.30 46.46 51.86
 56.90 66.17 68.85 77.76 80.52 83.81 89.39 95.49
 104.79 108.41 114.34 128.26 130.02 138.29 152.17
 165.83 170.27 176.05 187.10 192.63 209.68 214.94
 225.92 229.51 233.33 239.78 247.81 257.24 267.29
 270.61 278.31 286.63 299.73 301.53 303.72 317.08
 337.18 345.31 351.81 358.73 366.06 370.24 384.88
 394.73 405.70 413.61 416.28 423.88 427.69 449.24
 466.02 474.98 483.66 484.67 487.58 501.64 524.47
 560.99 565.29 570.27 583.77 603.86 611.01 628.27
 629.08 632.91 654.03 668.22 689.32 710.73 712.70
 719.55 728.29 748.57 755.72 762.05 764.50 769.36
 777.19 779.60 786.16 810.32 822.25 834.88 856.47
 862.50 871.30 889.68 915.94 922.40 926.49 930.25
 939.66 942.97 948.87 953.10 955.53 957.55 970.44
 974.23 990.84 997.57 1004.71 1010.04 1013.03
 1013.36 1015.14 1030.54 1032.06 1034.02 1035.81
 1037.96 1038.62 1041.25 1045.36 1048.67 1055.59
 1056.83 1057.82 1063.31 1100.56 1107.10 1109.01
 1132.72 1145.75 1162.03 1163.25 1176.26 1182.70
 1183.12 1184.41 1201.28 1205.82 1209.25 1213.77
 1218.67 1226.65 1235.46 1239.36 1248.99 1269.12
 1280.58 1303.52 1313.73 1316.75 1323.43 1338.52

1347.14 1348.70 1363.05 1365.91 1386.30 1387.78
1390.13 1397.65 1404.92 1408.34 1411.45 1417.10
1425.20 1429.47 1450.73 1451.51 1459.83 1460.29
1460.45 1471.36 1473.47 1474.46 1476.36 1478.73
1483.79 1486.60 1491.66 1499.29 1511.74 1520.67
1531.83 1537.67 1589.70 1630.61 1639.95 1650.97
1658.56 1661.55 1682.21 1823.25 2993.79 3031.90
3036.49 3039.12 3041.42 3043.26 3068.87 3088.98
3095.69 3119.54 3120.67 3122.45 3126.85 3128.45
3130.29 3138.98 3142.52 3160.47 3162.77 3163.32
3174.09 3176.47 3178.61 3181.31 3186.56 3187.07
3189.28 3193.99 3196.07 3199.69 3200.13 3204.16
3204.92 3213.11 3215.81 3217.66

=====

^sA4

=====

9.61 19.22 23.50 24.49 37.22 38.40 41.41 45.49 52.87
58.62 68.90 74.71 79.20 82.08 87.02 97.64 102.32
106.41 120.96 129.46 146.76 163.14 168.91 182.45
185.46 193.77 194.14 208.13 217.82 221.53 227.55
237.57 240.82 250.18 254.57 262.19 275.14 281.38
286.65 289.46 304.07 308.37 327.85 334.85 345.48
358.50 363.69 371.61 379.49 397.82 404.55 418.62
421.97 422.78 427.56 446.23 463.23 470.78 484.32
488.00 506.41 514.07 529.44 559.46 568.76 569.54
578.05 608.88 614.60 618.13 630.72 632.77 651.38
659.11 673.54 693.66 712.11 719.45 724.08 731.76
768.42 770.57 770.77 777.11 779.85 782.72 789.62
792.06 817.30 837.26 844.31 860.01 868.97 874.69
890.91 893.48 901.89 916.63 925.32 942.15 945.09
950.83 955.42 964.20 976.02 977.51 987.55 989.95
999.35 1004.85 1008.30 1012.39 1013.19 1015.40
1017.61 1028.50 1030.28 1031.56 1037.70 1039.54
1042.00 1045.25 1047.81 1050.01 1051.22 1059.48
1084.71 1091.02 1100.85 1111.06 1121.54 1126.52
1141.87 1148.22 1159.72 1168.01 1178.30 1178.97
1183.14 1197.70 1205.52 1210.77 1220.73 1223.15
1228.21 1239.39 1243.30 1245.76 1275.15 1284.10
1299.39 1306.58 1315.68 1326.27 1342.88 1344.80
1351.12 1360.64 1363.05 1373.42 1383.67 1385.76
1390.04 1398.20 1402.57 1404.61 1417.63 1425.71
1448.48 1451.31 1452.58 1458.29 1460.00 1461.25
1466.61 1467.65 1469.26 1470.10 1475.34 1478.82
1482.16 1483.33 1493.74 1497.62 1510.34 1525.64
1536.85 1608.50 1629.22 1635.02 1650.46 1658.25
1658.89 1743.90 1874.57 3034.10 3036.39 3040.43
3042.92 3044.35 3047.11 3070.27 3084.61 3090.99
3098.24 3120.60 3121.31 3121.42 3131.11 3134.60
3136.91 3138.34 3144.12 3160.72 3163.03 3163.17
3164.68 3178.56 3179.05 3183.46 3185.36 3187.04
3187.72 3192.02 3195.82 3199.26 3202.60 3203.98
3207.74 3216.43 3221.88

=====

^sA4-TS

=====

-227.06 7.34 8.38 10.42 21.01 27.94 35.15 36.39
42.63 48.77 51.04 54.27 67.13 74.12 75.89 79.78
91.44 100.42 113.19 118.24 145.75 156.56 164.13
181.80 183.83 194.83 203.54 205.20 209.89 229.20
234.83 237.62 241.57 249.11 261.65 270.40 272.56
276.26 281.86 291.42 296.31 311.90 327.69 334.03
340.75 345.36 352.92 359.77 382.74 389.77 391.37
403.03 414.58 420.33 426.77 444.45 445.00 448.86
462.27 487.75 488.59 506.44 514.25 544.21 559.88
569.63 571.31 576.15 588.73 616.83 623.99 630.21
633.26 660.90 666.55 671.30 690.03 710.72 724.42
730.96 754.10 768.51 769.75 775.46 776.31 781.19
782.55 791.48 792.97 816.74 824.74 858.10 863.49
867.09 874.36 892.17 901.85 907.08 918.11 923.04
942.54 947.79 950.97 963.84 966.72 973.55 979.62
986.98 994.98 1004.42 1007.32 1011.72 1012.28
1015.02 1016.42 020.99 1029.19 1032.66 1039.59
1040.69 1042.16 1044.14 1047.80 1052.48 1057.46
1069.11 1079.64 1084.47 1096.17 1112.21 1117.32
1126.55 1134.92 1149.22 1163.50 1175.04 1179.67
1184.04 1199.17 1204.30 1208.20 1210.59 1218.50
1224.24 1233.26 1238.20 1238.84 1245.42 1250.50
1304.99 1308.69 1310.85 1316.61 1322.63 1328.99
1341.21 1341.98 1361.95 1363.92 1372.03 1379.91
1385.01 1387.93 1394.18 395.51 1404.88 1420.18
1423.69 1441.59 1447.52 1455.95 1457.74 1459.14
1460.64 1461.46 1462.96 1467.04 1472.58 1472.98
1476.35 1478.24 1482.32 1494.63 1494.89 1519.97
1526.60 1535.63 1545.28 1607.38 1627.40 1636.83
1648.69 1654.96 1659.60 1841.22 3019.52 3030.04
3036.31 3037.66 3039.59 3043.62 3065.72 3083.11
3091.97 3097.03 3115.27 3115.43 3124.53 3126.11
3128.88 3133.27 3139.41 3144.94 3152.82 3157.16
3159.54 3170.13 3176.88 3179.44 3181.05 3184.16
3187.18 3192.14 3194.89 3195.03 3197.43 3202.12
3203.97 3207.63 3213.25 3221.09

=====

^sA5

=====

12.14 19.78 24.85 28.27 33.30 37.45 39.29 47.51
53.65 59.30 67.82 68.83 79.75 81.74 87.93 89.29
105.68 119.15 120.23 136.72 146.50 156.15 171.01
176.26 179.27 189.37 202.53 207.64 216.05 228.79
230.42 245.84 252.78 260.43 271.33 277.18 277.96
280.96 285.12 287.65 289.81 302.01 321.93 327.65
335.89 348.67 362.46 373.32 384.09 394.58 399.47
401.15 411.68 421.65 422.51 427.53 438.16 457.12
468.98 484.50 488.10 499.62 512.50 523.31 550.81
557.31 564.93 568.79 572.94 614.06 627.91 630.26
632.64 657.02 661.18 663.33 681.84 713.68 720.99
725.25 761.25 764.46 766.65 773.20 779.85 781.68
792.96 794.49 796.33 819.58 821.74 864.99 869.02
870.19 874.01 898.24 907.13 923.41 927.66 946.20
952.85 955.66 967.90 969.51 974.50 976.70 986.43
995.25 1000.94 1002.31 1006.80 1008.27 1012.09
1019.56 1022.26 1030.47 1037.59 1040.38 1040.91
1044.71 1045.61 1046.09 1049.72 1052.77 1059.70
1076.75 1082.12 1092.19 1103.81 1115.99 1118.06
1132.87 1152.48 1168.41 1178.64 1180.16 1187.87

1195.05 1198.29 1206.60 1206.89 1215.54 1220.47
 1222.88 1233.51 1246.23 1250.96 1251.57 1309.65
 1315.12 1322.03 1322.56 1337.19 1345.00 1346.93
 1352.35 1358.67 1367.67 1370.71 1386.49 1390.37
 1395.85 1406.81 1408.62 1413.61 1415.39 1426.09
 1440.77 1448.91 1452.34 1456.71 1457.47 1459.75
 1461.79 1467.45 1471.31 1472.54 1473.76 1474.94
 1479.38 1482.25 1482.49 1497.11 1508.43 1512.20
 1523.82 1524.22 1539.09 1601.29 1623.98 1632.56
 1641.54 1652.56 1656.82 1767.18 3033.33 3035.37
 3036.33 3038.58 3038.78 3046.48 3047.61 3091.65
 3096.97 3113.90 3114.42 3118.27 3118.43 3120.41
 3130.04 3134.71 3149.44 3152.28 3153.55 3162.05
 3164.61 3166.12 3176.37 3184.72 3185.24 3191.79
 3196.37 3196.63 3200.99 3206.13 3211.32 3216.35
 3225.98 3233.67 3235.12 3250.81

=====

B1

=====

32.76 43.95 77.46 94.57 95.91 102.61 107.52 112.83
 138.55 141.29 141.69 157.66 203.38 205.54 298.44
 306.04 310.75 314.57 326.53 354.87 449.75 463.96
 476.88 520.33 535.45 538.72 569.28 597.21 605.53
 626.50 628.27 679.69 715.87 812.02 815.52 958.45
 960.70 971.56 1011.49 1018.49 1021.00 1031.78
 1034.68 1088.55 1091.96 1105.56 1116.27 1130.43
 1181.83 1185.47 1373.87 1379.28 1386.06 1395.84
 1398.24 1413.72 1415.55 1418.53 1421.35 1422.78
 1429.69 1438.44 1444.86 1445.02 1451.35 1456.77
 1457.87 1466.33 1472.47 1480.05 1481.25 1495.50
 1518.75 1537.28 1544.24 2570.91 2654.48 2791.86
 3025.19 3030.85 3032.75 3036.87 3037.85 3114.60
 3117.23 3118.32 3122.02 3122.96 3154.21 3157.00
 3157.99 3160.61 3162.66

=====

B2

=====

7.83 16.87 24.84 41.72 56.34 61.40 65.92 87.96 99.73
 104.98 107.61 109.27 113.97 119.74 123.74 126.38
 139.00 146.87 162.00 163.80 179.46 205.95 208.61
 235.13 240.60 252.46 266.77 272.90 280.15 289.22
 307.36 308.56 311.06 314.59 315.51 332.60 336.86
 341.45 355.21 372.89 400.24 406.58 422.32 445.21
 452.45 459.19 463.58 501.76 538.14 539.85 567.10
 570.42 598.64 617.83 629.16 634.21 636.37 685.28
 756.18 768.89 775.01 812.79 817.33 819.52 819.86
 929.02 941.30 950.03 958.93 960.15 965.06 984.12
 1018.12 1021.98 1026.89 1034.81 1037.73 1040.08
 1040.89 1053.72 1064.69 1083.34 1090.88 1093.32
 1118.51 1153.56 1182.98 1184.95 1214.70 1235.21
 1241.05 1249.95 1315.50 1376.70 1382.37 1382.60
 1390.31 1391.54 1394.55 1398.40 1399.46 1404.47
 1417.38 1419.18 1424.31 1425.69 1433.53 1433.79
 1444.82 1447.83 1450.54 1453.20 1454.10 1458.62
 1460.49 1462.57 1464.05 1476.68 1476.92 1479.11
 1480.44 1492.28 1499.76 1510.40 1535.50 1609.51

1859.03 3005.78 3008.56 3017.16 3032.10 3032.28
 3036.57 3037.17 3039.35 3086.95 3089.64 3093.13
 3097.39 3111.80 3114.29 3118.63 3119.89 3121.28
 3125.06 3128.59 3130.45 3149.60 3153.96 3154.66
 3162.28 3166.94 3189.09

=====

RB3

=====

6.91 16.89 25.45 29.43 41.28 44.73 48.14 55.69 56.40
 72.07 77.92 86.12 89.50 96.87 97.55 97.79 104.58
 118.23 119.10 126.92 130.41 139.04 141.16 149.20
 164.59 173.30 175.06 184.99 186.01 193.32 200.17
 208.10 228.16 236.27 240.74 247.12 264.00 275.80
 277.04 280.86 294.84 306.39 309.68 310.61 313.32
 315.68 324.06 328.90 341.12 349.22 364.10 377.96
 401.81 402.55 409.54 424.36 427.94 433.60 439.09
 441.87 447.51 475.17 498.81 508.36 537.85 540.95
 568.66 570.82 590.78 599.18 610.19 631.82 632.24
 636.88 638.16 680.96 728.61 741.10 752.53 765.14
 771.79 777.26 784.42 811.89 817.57 821.60 825.42
 838.78 878.92 922.03 927.22 941.61 946.49 949.12
 955.55 960.01 964.37 974.57 980.48 986.56 998.16
 1007.82 1014.73 1021.25 1022.06 1026.98 1032.92
 1035.70 1037.06 1039.14 1039.59 1040.77 1046.19
 1051.85 1063.23 1081.41 1090.58 1093.29 1104.58
 1120.08 1139.49 1143.10 1179.91 1182.20 1183.90
 1185.96 1205.38 1218.20 1224.13 1237.99 1255.51
 1262.51 1290.74 1302.43 1315.65 1331.54 1351.19
 1354.48 1365.09 1379.32 1381.28 1381.79 1385.24
 1392.70 1395.23 1396.26 1397.35 1409.58 1415.65
 1419.34 1423.95 1430.41 1430.85 1431.55 1438.83
 1448.85 1449.42 1450.75 1451.62 1455.49 1459.26
 1461.18 1463.79 1464.00 1464.68 1475.65 1476.66
 1480.13 1482.67 1484.11 1486.75 1493.90 1500.02
 1505.43 1522.93 1533.96 1613.95 1622.35 1639.69
 1652.72 1820.54 3031.99 3032.44 3033.23 3034.37
 3035.11 3037.48 3038.64 3039.39 3047.19 3060.79
 3089.29 3103.66 3113.84 3116.20 3118.56 3119.85
 3120.15 3121.94 3130.19 3131.10 3134.48 3134.81
 3134.86 3135.39 3137.11 3140.46 3140.64 3150.38
 3152.29 3154.22 3154.32 3157.27 3166.28 3167.91
 3180.63 3185.44 3193.71 3259.19

=====

RB3-TS

=====

-322.72 10.76 19.88 21.55 24.20 31.97 35.33 40.73
 46.45 48.22 51.29 58.38 65.87 81.75 87.95 100.28
 107.12 112.24 117.48 119.24 122.38 127.23 129.25
 146.61 149.43 157.59 163.66 174.17 179.97 182.79
 197.51 209.92 220.69 235.65 236.47 240.51 253.29
 273.71 277.88 289.73 296.56 300.57 303.50 309.54
 311.76 319.58 324.04 337.98 358.93 363.13 366.78
 399.56 401.32 402.39 414.35 418.87 424.72 441.36
 450.98 455.34 484.25 493.36 501.77 538.56 540.69
 549.74 568.28 570.05 591.79 597.55 609.46 629.09
 633.29 634.38 640.46 681.78 723.59 733.02 748.03

769.36 770.73 778.14 786.84 811.81 812.54 818.45
824.21 859.41 865.01 918.33 920.10 924.10 945.08
946.81 954.37 957.20 960.02 966.13 977.55 987.57
993.67 1002.82 1012.57 1022.32 1024.83 1029.31
1032.66 1033.40 1040.82 1042.44 1044.50 1049.93
1050.55 1055.95 1080.02 1089.09 1090.12 1092.17
1108.87 1118.60 1126.42 1139.97 1178.11 1180.29
1182.56 1188.77 1197.84 1211.74 1222.84 1228.52
1230.67 1248.49 1256.86 1266.83 1321.33 1324.05
1338.24 1350.32 1362.10 1369.94 1378.47 1381.92
1387.93 1394.67 1397.24 1397.79 1398.64 1402.35
1414.39 1417.24 1423.99 1425.65 1432.69 1435.06
1439.88 1444.62 1448.50 1449.92 1452.55 1454.52
1458.92 1459.77 1460.68 1462.37 1464.95 1472.45
1473.94 1475.67 1478.42 1483.22 1483.47 1491.67
1493.16 1500.11 1511.86 1526.82 1548.15 1638.53
1660.59 1697.55 1845.89 3028.88 3030.47 3032.65
3032.75 3034.79 3038.89 3040.41 3041.08 3042.10
3049.33 3086.64 3090.73 3093.34 3102.74 3110.68
3115.11 3116.61 3116.73 3116.92 3119.96 3121.09
3127.99 3131.29 3134.24 3140.15 3142.07 3148.84
3151.71 3157.95 3158.51 3162.69 3163.46 3175.78
3178.14 3187.74 3188.16 3189.79 3198.34

R_{B4}

12.70 17.15 22.91 23.76 29.38 33.25 38.25 46.92
57.02 58.86 69.10 73.65 87.67 96.67 103.07 111.97
122.50 132.19 136.27 145.83 147.75 151.57 158.70
170.31 171.64 181.36 189.42 195.48 199.77 208.37
214.62 228.32 238.10 254.42 256.33 270.55 274.14
283.86 297.67 298.47 303.36 310.75 313.70 317.69
323.71 333.57 338.48 356.29 356.97 367.53 380.21
392.67 401.52 408.95 418.73 427.84 443.02 456.67
461.05 492.38 506.84 518.32 534.93 539.86 564.69
569.00 584.80 593.87 600.58 613.23 633.76 635.32
645.81 653.32 682.33 719.40 723.47 770.99 775.94
781.27 785.78 800.72 804.53 810.08 814.77 822.04
836.68 856.23 863.89 920.05 936.90 942.58 948.29
955.89 957.28 961.72 962.61 980.88 987.32 1002.97
1012.52 1013.81 1025.04 1030.33 1031.61 1033.52
1039.92 1042.20 1043.51 1044.98 1051.95 1052.12
1063.54 1082.70 1086.02 1088.05 1090.79 1094.32
1117.57 1122.36 1133.68 1153.09 1175.51 1179.35
1180.24 1180.42 1199.27 1220.19 1223.69 1233.56
1248.93 1259.21 1284.91 1293.32 1298.31 1315.55
1344.92 1350.82 1367.63 1371.95 1374.04 1375.69
1387.37 1390.55 1395.71 1396.11 1399.11 1399.55
1403.55 1417.15 1421.55 1427.16 1431.10 1437.69
1442.42 1448.72 1450.17 1452.56 1454.33 1457.29
1462.05 1463.15 1464.56 1465.07 1466.03 1466.47
1468.56 1477.17 1478.11 1478.52 1481.41 1484.49
1500.79 1501.84 1504.64 1529.00 1581.80 1640.70
1664.03 1736.38 1863.39 3023.35 3025.65 3028.44
3028.67 3030.55 3030.84 3032.33 3036.02 3037.18
3037.34 3054.22 3061.12 3070.49 3090.20 3102.35
3103.45 3105.74 3111.54 3112.06 3112.59 3117.00
3122.46 3123.38 3128.96 3131.40 3135.99 3136.74

3140.79 3143.78 3146.02 3147.90 3151.26 3154.12
3156.02 3157.65 3178.12 3185.14 3199.21

R_{B4-TS}

-196.29 4.31 7.76 14.83 15.99 20.12 31.27 45.10
53.91 55.69 59.28 73.05 85.23 97.15 103.24 110.59
115.74 126.83 132.83 138.58 148.58 152.20 161.22
163.28 165.04 168.49 171.10 180.18 188.09 199.98
214.51 221.21 235.74 243.09 253.22 255.72 271.51
278.63 291.64 295.30 298.80 307.94 309.93 319.40
320.53 325.55 330.34 347.79 350.87 362.17 374.46
389.22 393.66 396.66 404.93 423.38 431.10 442.91
445.34 480.18 485.34 509.82 534.51 538.57 540.74
556.83 567.62 571.43 588.00 594.96 598.66 622.31
633.30 638.83 642.87 688.13 726.35 735.02 753.06
771.82 780.05 782.73 785.48 814.91 815.26 815.82
822.07 841.01 870.43 901.57 920.91 936.30 945.04
951.94 953.78 956.61 965.49 975.31 979.25 996.08
1006.49 1009.31 1014.15 1021.16 1029.07 1032.32
1034.10 1038.33 1040.35 1046.44 1050.46 1052.73
1069.63 1075.67 1080.60 1087.15 1090.37 1101.82
1115.33 1118.44 1126.84 1162.85 1174.05 1179.67
1179.76 1188.64 1202.60 1215.86 1217.99 1231.86
1233.53 1246.20 1257.10 1274.42 1309.66 1312.82
1337.33 1339.58 1362.90 1367.23 1372.82 1378.41
1379.05 1383.79 1393.30 1394.64 1395.47 1399.55
1400.48 1419.13 1424.20 1425.31 1427.98 1434.81
1439.35 1441.98 1445.68 1447.46 1448.19 1451.80
1454.70 1459.86 1461.12 1461.47 1462.59 1465.30
1466.11 1469.16 1472.92 1477.36 1480.50 1482.87
1491.99 1497.80 1510.30 1526.77 1536.04 1564.64
1633.68 1657.50 1847.82 2983.35 3029.57 3029.90
3032.59 3034.28 3034.86 3036.04 3038.88 3045.47
3045.98 3049.70 3075.14 3091.08 3106.13 3108.15
3110.16 3111.16 3113.44 3114.75 3115.41 3119.87
3122.58 3130.84 3131.54 3142.85 3143.38 3145.80
3146.78 3146.93 3149.42 3150.01 3156.34 3160.02
3165.98 3172.91 3183.14 3195.97 3219.48

R_{B5}

12.13 15.52 17.67 21.64 29.43 37.28 38.84 49.91
55.04 61.25 67.97 73.02 85.92 97.28 109.47 117.24
132.28 138.00 140.74 148.61 164.38 168.43 170.77
172.50 181.14 184.26 190.45 194.84 204.18 214.00
222.55 225.52 238.10 242.33 249.51 260.16 274.50
280.54 286.18 297.23 299.00 299.49 307.96 311.75
314.80 323.62 327.27 344.63 361.37 372.78 374.00
390.40 395.60 403.48 411.26 420.57 426.93 436.61
451.60 464.41 470.24 510.80 514.16 533.39 539.69
557.30 563.50 573.37 576.58 598.65 623.25 631.22
632.53 638.47 661.23 682.25 724.66 748.25 761.73
772.57 776.51 781.40 794.84 815.51 818.39 822.57
825.59 865.14 871.40 905.92 928.98 947.47 952.85
956.77 958.02 971.47 977.21 984.57 988.18 1004.81

1005.94 1011.60 1019.54 1023.51 1028.48 1031.02
1038.69 1044.61 1046.31 1046.79 1049.18 1050.51
1078.15 1083.77 1084.79 1087.44 1095.97 1101.12
1119.17 1120.69 1134.37 1173.26 1176.67 1178.31
1182.55 1198.14 1213.48 1223.14 1224.81 1237.16
1242.36 1252.77 1306.57 1315.02 1330.14 1349.47
1356.11 1363.87 1366.68 1376.96 1381.96 1385.27
1391.58 1392.50 1395.80 1397.68 1401.44 1406.06
1414.62 1415.13 1418.59 1423.50 1435.35 1441.00
1442.06 1448.51 1449.18 1450.54 1453.48 1456.04
1457.01 1457.98 1460.96 1461.44 1466.95 1471.31
1473.53 1475.37 1478.67 1479.76 1480.05 1483.99
1489.08 1494.77 1509.91 1520.22 1522.78 1524.83
1630.26 1654.80 1788.61 3012.43 3016.35 3022.96
3023.44 3025.02 3030.48 3033.19 3033.90 3036.35
3041.31 3042.16 3072.15 3091.35 3096.72 3097.08
3101.66 3105.08 3108.43 3112.44 3117.91 3121.28
3123.20 3126.46 3130.48 3131.51 3142.21 3144.42
3149.24 3149.26 3152.90 3154.80 3160.62 3164.98
3171.29 3175.91 3183.47 3195.29 3274.21

=====

B2'

=====

3.95 11.98 21.78 26.26 33.97 36.08 53.94 58.09 69.57
75.26 83.34 85.45 99.03 107.15 120.75 133.60 157.61
172.73 181.98 189.19 202.00 209.30 226.21 233.82
241.08 247.17 249.73 266.33 269.97 280.82 285.38
293.53 304.26 314.62 324.91 338.81 344.01 355.93
366.30 395.57 402.18 413.01 414.93 420.18 433.77
438.53 453.25 459.95 500.02 503.32 517.13 560.63
562.72 585.19 620.61 624.48 629.74 640.88 659.34
679.91 690.98 711.92 723.29 748.96 771.19 775.04
775.97 821.58 821.69 824.05 829.28 868.21 871.35
909.02 932.70 939.87 943.02 943.23 950.27 957.10
958.45 967.83 975.03 988.74 1011.01 1011.53
1020.93 1036.56 1040.88 1042.33 1047.13 1050.82
1054.69 1068.14 1079.22 1083.04 1087.09 1108.10
1131.19 1153.92 1157.85 164.04 1182.52 1185.63
1201.92 1220.38 1233.22 1235.47 1250.06 1254.55
1257.13 1277.15 1304.26 1314.47 1325.04 1338.17
1362.07 1363.39 1376.66 1383.60 1385.32 1387.24
1393.30 1396.06 1400.57 1424.04 1428.19 1432.18
1437.74 1443.49 1450.16 1451.21 1458.79 1461.01
1461.19 1466.71 1471.57 1475.58 1476.47 1476.80
1482.83 1485.21 1498.56 1515.46 1537.84 1605.84
1629.40 1655.57 1861.45 3024.69 3027.93 3033.85
3035.61 3036.33 3043.15 3044.29 3046.44 3073.14
3083.18 3100.87 3103.60 3110.28 3122.00 3123.04
3124.29 3131.11 3134.74 3136.48 3138.81 3156.20
3176.82 3180.47 3186.92 3188.86 3198.52 3206.55
3234.40

=====

R^{B4}

=====

4.10 11.12 17.84 23.99 26.12 31.70 32.30 38.58 47.06
52.31 57.06 65.73 69.52 70.87 73.27 76.95 85.29

100.84 105.90 129.15 140.58 153.51 166.17 173.34
178.65 186.33 199.60 203.44 208.41 217.15 224.72
230.66 237.55 239.74 255.81 259.23 270.08 276.91
282.32 289.91 302.88 309.75 310.95 320.35 337.84
339.54 355.43 363.91 372.05 376.74 388.30 404.11
412.06 414.28 417.38 420.56 426.59 439.83 451.42
464.99 490.42 500.67 502.66 511.57 521.44 562.90
567.38 586.38 596.23 599.28 612.16 617.14 628.87
633.27 649.57 658.49 687.13 702.41 714.87 720.45
723.84 723.96 767.97 773.16 774.89 778.47 780.53
800.48 803.10 818.95 826.30 829.21 834.85 851.69
863.32 868.16 871.79 904.40 919.49 921.55 933.62
939.71 941.30 947.62 955.82 956.13 961.12 963.65
978.02 989.52 991.82 1002.16 1005.45 1009.14
1012.17 1012.74 1020.68 1031.40 1039.29 1041.14
1042.13 1042.67 1050.37 1051.63 1055.74 1073.84
1077.63 1079.20 1082.91 1088.72 1097.41 1105.28
1123.37 1127.88 1132.46 1148.76 1152.03 1155.76
1179.28 1182.78 1183.65 1187.18 1197.34 1198.87
1223.27 1223.62 1225.57 1227.28 1246.51 1251.98
1259.18 1270.25 1285.49 1295.57 1303.08 1304.55
1310.38 1314.60 1332.75 1345.32 1350.86 1357.07
1358.49 1361.58 1362.79 1374.23 1383.84 1385.98
1392.59 1394.76 1395.88 1397.65 1427.41 1430.74
1435.54 1438.90 1445.05 1451.31 1451.95 1453.59
1460.91 1462.04 1462.99 1465.19 1470.30 1473.24
1475.14 1476.40 1476.74 1477.66 1480.61 1482.54
1490.88 1499.75 1525.80 1537.78 1569.57 1631.28
1636.45 1658.47 1659.94 1727.05 1880.63 3025.92
3026.00 3033.08 3035.24 3035.60 3039.09 3041.31
3041.55 3045.33 3047.55 3053.33 3075.08 3083.66
3088.24 3090.17 3093.51 3098.35 3106.23 3106.70
3113.73 3120.16 3128.73 3131.81 3137.23 3140.26
3152.76 3153.71 3155.19 3155.90 3157.17 3166.22
3173.02 3177.18 3178.51 3185.47 3187.64 3194.66
3197.81 3202.94 3212.68

=====

R^{C5}

=====

9.90 14.25 19.70 20.05 21.36 30.52 36.90 38.84 43.23
45.10 55.80 56.98 70.55 80.31 89.00 98.94 103.83
118.16 121.11 129.07 137.89 147.46 165.04 171.44
181.41 186.63 200.17 216.20 218.71 229.64 232.49
240.06 249.28 253.59 264.52 272.89 279.78 284.72
286.43 292.01 301.91 305.36 313.04 323.79 331.15
349.85 352.35 374.08 385.68 392.61 395.81 403.41
418.67 418.87 425.31 431.73 442.25 451.25 469.65
476.97 486.34 492.02 498.76 500.89 516.31 543.58
548.72 556.15 564.59 567.06 584.44 613.59 625.45
627.49 629.00 632.60 641.65 657.78 659.05 663.39
712.03 724.21 724.53 726.85 759.42 762.91 775.72
778.10 781.52 792.49 797.32 812.42 820.07 832.74
837.11 856.38 860.80 862.18 866.98 872.46 891.14
906.50 913.89 924.08 929.32 948.07 952.96 954.19
967.87 974.09 987.24 991.29 992.52 1003.51 1004.04
1004.33 1007.84 1010.90 1012.21 1015.48 1016.71
1020.83 1026.47 1029.70 1034.10 1041.95 1042.95
1043.90 1045.50 1045.76 1050.35 1051.03 1056.75
1068.24 1078.77 1091.47 1100.22 1112.38 1126.99

1131.46 1134.43 1136.85 1140.95 1175.79 1177.64
 1181.73 1186.30 1200.13 1203.31 1204.55 1217.83
 1219.07 1227.14 1228.24 1233.46 1237.54 1244.29
 1253.26 1255.11 1288.81 1315.57 1317.21 1320.16
 1323.65 1331.46 1347.80 1355.31 1357.78 1360.58
 1366.86 1376.58 1383.41 1390.86 1396.60 1397.16
 1398.67 1411.73 1412.89 1417.89 1428.58 1435.71
 1449.85 1455.02 1458.28 1458.83 1459.34 1461.64
 1465.86 1466.13 1467.69 1471.31 1473.29 1474.74
 1476.28 1479.29 1482.06 1495.34 1506.23 1515.71
 1519.67 1525.16 1533.23 1547.79 1593.27 1618.43
 1625.69 1633.95 1640.65 1645.54 1652.64 1657.91
 3025.28 3026.02 3030.51 3032.14 3037.12 3038.42
 3040.76 3060.14 3076.80 3097.78 3112.41 3116.42
 3120.47 3124.69 3124.91 3130.10 3131.17 3134.26
 3134.30 3145.65 3161.21 3163.04 3167.60 3174.51
 3175.53 3181.98 3184.09 3185.38 3187.61 3192.16
 3194.91 3196.45 3202.42 3202.94 3205.21 3208.83
 3215.76 3217.44 3219.63 3248.95 3253.91

=====

RC5-TS1

=====

-216.56 16.91 22.27 27.46 30.24 35.70 43.66 47.72
 52.49 58.86 62.00 77.67 78.58 88.07 92.98 97.70
 105.30 118.37 121.95 126.96 138.44 142.03 145.17
 159.19 170.56 181.17 188.67 196.08 200.56 218.00
 223.46 229.62 247.83 255.61 270.62 272.49 281.45
 283.96 290.49 305.47 313.31 314.82 319.01 339.53
 345.86 349.26 354.58 358.61 383.77 388.20 397.55
 402.50 406.76 419.14 420.76 423.16 430.07 446.19
 448.47 457.28 478.23 489.39 500.33 504.25 512.78
 528.10 542.66 557.00 559.02 568.62 578.20 613.20
 615.36 626.98 628.33 633.95 642.74 655.37 660.62
 665.18 710.76 726.51 732.42 741.03 757.67 777.21
 778.98 781.80 789.51 792.63 796.18 807.60 816.62
 822.69 832.92 839.37 862.97 863.57 878.09 884.22
 886.42 898.04 909.37 924.11 925.23 940.45 946.08
 948.37 955.03 970.50 986.74 990.02 992.15 993.05
 1001.32 1004.26 1009.58 1010.61 1011.58 1012.73
 1014.38 1022.87 1028.32 1030.77 1030.85 1039.00
 1040.70 1044.33 1044.74 1046.35 1048.22 1048.67
 1053.59 1057.44 1070.27 1079.14 100.68 1110.60
 1113.19 1127.96 1137.36 1141.22 1143.27 1177.59
 1180.74 1183.77 1185.08 1194.04 1200.35 1205.30
 1207.34 1214.42 1214.98 1222.23 1232.16 1233.45
 1239.36 1244.72 1248.93 1284.37 1303.80 1321.20
 1324.00 1331.37 1334.21 1347.85 1350.79 1356.24
 1358.64 1365.45 1373.44 1387.94 1393.83 1397.79
 1397.94 1398.62 1411.70 1415.84 1429.94 1430.47
 1437.66 1451.90 1455.76 1457.60 1461.14 1462.93
 1465.94 1467.97 1470.43 1471.56 1473.33 1474.90
 1475.29 1477.94 1478.14 1481.35 1494.33 1511.20
 1515.51 1520.86 1524.20 1537.87 1557.59 1588.72
 1613.65 1623.42 1627.24 1642.84 1649.27 1652.37
 1657.31 2966.80 2969.96 2979.18 3006.69 3020.19
 3034.11 3039.36 3042.89 3046.21 3048.11 3049.23
 3092.18 3092.47 3104.34 3104.94 3109.23 3113.27
 3120.64 3123.23 3156.44 3159.37 3166.76 3170.46
 3177.91 3179.13 3181.09 3184.29 3185.47 3185.95

3189.69 3193.71 3196.59 3200.13 3200.45 3202.86
 3204.57 3207.95 3212.95 3218.57 3271.04 3326.23

=====

RC6

=====

5.15 12.34 16.89 19.70 25.79 31.70 33.00 38.40 47.55
 62.00 67.24 73.86 84.98 88.84 95.90 105.39 113.13
 122.92 126.88 140.00 143.84 158.55 165.94 173.50
 175.80 186.64 207.81 209.30 225.71 228.48 240.14
 244.26 259.66 265.15 269.99 281.70 283.95 289.38
 292.89 298.99 310.56 311.03 319.30 321.56 346.13
 351.33 360.12 374.07 381.08 396.24 405.95 410.42
 420.26 421.14 421.67 429.28 445.32 448.68 469.85
 474.79 486.13 490.43 502.60 511.03 530.04 542.01
 545.87 557.50 560.13 572.41 591.97 613.69 620.60
 629.57 644.34 651.59 657.19 661.43 667.21 703.07
 710.93 727.32 733.82 760.61 763.87 776.03 776.45
 794.54 795.93 810.07 817.83 822.59 834.22 855.91
 859.64 862.93 868.89 878.19 884.23 893.86 901.52
 907.89 923.69 924.46 933.06 948.19 952.59 967.61
 970.65 980.54 985.90 989.33 991.29 995.83 999.30
 1006.13 1010.28 1011.29 1017.26 1022.74 1029.62
 1033.51 1034.87 1036.32 1038.55 1044.26 1045.76
 1046.70 1047.35 1052.07 1060.02 1062.84 1067.47
 1077.63 1087.41 1107.12 1112.24 1125.67 1128.32
 1135.52 1139.60 1164.02 1168.79 1174.79 1177.42
 1182.28 1194.44 1203.26 1206.39 1208.30 1213.04
 1229.90 1232.53 1234.42 1243.59 1247.44 1253.95
 1257.29 1279.79 1282.37 1314.69 1319.21 1319.92
 1322.81 1323.99 1346.51 1353.00 1356.22 1365.93
 1381.02 1383.34 1387.27 1388.73 1395.86 1397.83
 1405.08 1412.66 1425.91 1428.09 1428.67 1443.36
 1448.92 1453.96 1455.31 1457.84 1458.88 1463.06
 1464.73 1465.64 1468.13 1470.59 1473.17 1473.89
 1476.66 1481.36 1484.29 1493.69 1496.80 1511.32
 1514.14 1524.59 1541.03 1565.94 1568.44 1589.61
 1622.40 1628.05 1650.14 1652.28 1655.18 1658.86
 2982.07 3013.53 3023.92 3024.72 3025.09 3029.06
 3034.62 3036.92 3093.05 3103.02 3103.51 3110.16
 3111.42 3111.98 3116.84 3122.34 3126.11 3129.11
 3130.52 3130.76 3166.62 3170.41 3171.96 3172.63
 3176.07 3180.19 3182.03 3183.36 3185.93 3191.70
 3191.84 3196.89 3197.23 3198.13 3208.27 3209.74
 3212.49 3213.47 3218.23 3231.09 3264.83

=====

RC7

=====

8.30 16.28 22.36 29.17 33.95 41.30 44.85 57.76 61.94
 66.38 69.86 74.78 77.86 85.33 104.14 107.02 117.21
 132.13 141.73 149.01 158.53 163.08 172.92 179.07
 185.68 195.96 209.24 213.41 221.02 235.66 250.33
 261.51 265.66 268.99 273.23 282.99 290.38 305.72
 310.15 311.77 317.45 325.23 330.22 353.38 358.88
 366.90 384.39 398.85 401.79 414.45 417.35 422.65
 426.25 433.06 436.54 442.55 446.35 460.22 476.44
 481.53 489.97 496.23 502.39 511.72 520.57 542.06

557.92 564.53 566.34 583.76 600.26 610.71 621.17
628.46 638.16 642.59 659.15 663.46 667.69 670.79
710.60 727.74 729.37 730.44 756.36 761.31 768.70
778.73 791.84 800.82 815.59 817.59 832.03 847.26
858.74 863.45 866.83 876.21 882.85 896.44 907.04
911.11 924.68 925.72 927.47 945.37 953.93 971.11
979.80 985.92 986.62 992.47 998.38 1002.87 1011.62
1014.28 1018.58 1022.10 1023.70 1025.77 1028.65
1039.73 1039.96 1043.90 1044.31 1047.05 1047.53
1048.63 1061.04 1061.78 1067.33 1087.40 1097.06
1101.58 1115.62 1132.23 1136.06 1137.25 1146.22
1152.45 1169.06 117.19 1179.56 1183.29 1186.18
1206.51 1208.57 1213.73 1214.00 1222.08 1224.52
1233.67 1235.83 1242.30 1243.78 1252.47 1259.27
1286.15 1322.25 1324.07 1324.93 1329.57 1334.95
1337.71 1349.16 1363.83 1365.07 1369.84 1377.91
1379.16 1385.24 1388.81 1390.97 1394.42 1407.75
1413.72 1416.05 1430.66 1433.41 1438.53 1446.58
1450.17 1451.41 1456.55 1460.55 1463.79 1469.49
1471.59 1474.16 1474.48 1475.33 1477.64 1478.97
1483.03 1485.20 1487.44 1497.89 1512.05 1516.91
1521.28 1531.19 1540.51 1554.18 1579.99 1623.16
1629.92 1639.87 1647.34 1655.23 1658.46 1664.88
2816.84 2887.50 936.99 2995.59 3000.72 3009.00
3010.56 3028.81 3032.28 3032.86 3077.59 3079.48
3085.37 3089.30 3098.91 3109.46 3110.70 3114.84
3116.37 3122.55 3156.70 3158.06 3160.55 3165.72
3168.72 3181.25 3183.14 3183.91 3186.05 3189.60
3190.04 3196.74 3198.33 3200.81 3204.33 3205.13
3209.87 3213.65 3220.38 3254.40 3428.55

RC5-TS2

-251.70 3.83 10.22 18.73 20.63 25.14 26.34 30.35
37.59 39.71 42.37 51.28 60.22 65.00 74.78 89.21
98.93 106.43 112.63 119.91 133.11 140.97 152.42
154.29 173.70 178.08 181.28 188.48 204.53 204.80
219.34 223.82 231.86 242.86 246.90 262.38 270.72
277.24 280.09 284.32 285.76 300.64 303.92 320.00
324.00 335.61 353.14 362.01 370.89 381.45 386.66
396.25 409.68 417.11 418.97 425.85 429.22 442.38
448.75 451.56 483.14 487.49 492.93 499.92 501.82
521.89 544.41 553.00 556.85 565.10 573.00 607.46
612.23 624.17 628.00 632.61 641.03 651.20 660.40
663.73 702.62 712.48 724.50 726.07 731.37 757.40
767.68 774.60 780.91 782.77 790.50 797.81 815.91
821.14 834.94 843.19 859.10 859.41 866.41 878.21
885.02 903.43 919.40 924.85 927.32 946.60 948.54
955.38 963.30 967.98 973.30 990.08 991.12 995.22
1002.94 1004.47 1004.87 1009.43 1012.07 1012.40
1013.25 1016.12 1020.37 1026.22 1029.19 1031.97
1041.37 1041.55 1043.67 1044.78 1047.33 1050.73
1051.51 1052.82 1057.65 1079.68 1085.53 1110.00
1119.61 1132.15 1137.62 1142.17 1172.20 1179.47
1180.43 1185.00 1189.75 1198.88 1204.53 1204.69
1214.15 1214.38 1224.26 1224.93 1233.89 1241.02
1247.17 1250.13 1253.49 1297.25 1309.28 1321.23
1322.03 1329.71 1333.63 1348.39 1351.40 1359.31
1361.70 1365.36 1370.84 1381.85 1391.27 1397.09

1397.91 1398.55 1411.44 1412.79 1430.05 1430.84
1449.02 1450.45 1456.79 1457.43 1460.19 1460.50
1465.10 1467.30 1469.72 1471.11 1472.37 1473.76
1475.20 1476.66 1482.00 1483.44 1492.72 1510.35
1513.06 1519.99 1525.02 1538.15 1555.15 1583.97
1620.43 1628.92 1634.49 1646.30 1651.44 1657.03
1657.33 3026.80 3027.05 3030.31 3034.88 3037.84
3038.14 3055.98 3066.65 3086.34 3098.15 3111.50
3116.02 3117.35 3119.55 3121.02 3124.92 3132.14
3133.97 3135.43 3157.50 3164.52 3166.48 3175.38
3176.38 3179.33 3180.28 3181.63 3183.83 3188.43
3189.38 3191.32 3199.28 3200.18 3200.94 3202.01
3207.74 3212.85 3215.00 3217.52 3263.07 3319.87

RC9

6.76 15.47 18.33 22.71 27.92 37.26 42.67 45.28 48.61
53.20 69.11 70.39 80.37 82.26 95.00 105.47 117.27
124.12 135.53 147.65 158.36 162.61 174.42 178.12
186.09 194.50 201.11 206.33 216.26 229.45 234.11
247.00 250.50 267.69 279.02 282.44 286.52 288.61
299.33 303.21 311.54 322.52 327.65 335.01 350.22
356.64 374.01 376.65 385.93 395.78 406.93 420.31
422.02 423.87 424.29 432.69 446.94 452.89 482.98
486.27 492.37 498.36 503.35 517.40 521.92 537.55
543.66 560.01 564.64 568.89 583.24 612.30 623.96
628.29 629.09 634.40 642.73 660.43 661.73 672.38
710.32 723.96 728.73 728.99 757.88 773.96 779.54
779.98 788.22 795.50 798.04 819.29 822.63 842.80
856.02 858.54 862.75 866.41 876.29 883.49 885.67
905.29 925.18 929.09 932.76 941.45 946.33 954.73
968.72 972.48 986.40 989.48 994.20 1002.59 1002.96
1007.60 1012.12 1012.71 1017.92 1019.38 1025.30
1028.59 1029.11 1034.41 1038.97 1041.07 1041.83
1043.56 1046.85 1052.05 1059.00 1068.53 1082.65
1085.28 1100.26 1111.50 1116.20 1126.38 1135.21
1141.97 1145.96 1149.64 1167.05 1178.10 1178.84
1184.97 1189.19 1199.83 1207.16 1207.32 1210.77
1212.38 1223.05 1230.52 1235.41 1241.87 1250.47
1253.42 1256.51 1304.94 1319.86 1322.79 1329.20
1333.16 1340.46 1345.84 1353.80 1365.28 1366.57
1366.96 1368.80 1381.94 1389.88 1391.00 1405.51
1413.49 1427.54 1433.92 1436.11 1445.39 1449.01
1455.03 1457.51 1458.17 1465.22 1469.55 1471.74
1472.41 1473.64 1475.63 1477.92 1481.83 1482.31
1482.79 1490.64 1492.16 1495.62 1510.87 1519.21
1521.46 1527.71 1539.46 1555.90 1583.49 1622.14
1629.23 1637.66 1648.13 1653.90 1657.18 1661.44
2701.79 2797.54 2846.52 3026.89 3031.12 3034.45
3035.88 3039.23 3057.59 3088.45 3112.67 3115.76
3117.37 3121.42 3123.01 3129.54 3134.05 3135.04
3148.15 3157.74 3158.66 3161.83 3171.37 3176.05
3179.63 3182.63 3182.88 3183.60 3187.82 3188.51
3194.26 3198.17 3198.77 3200.63 3207.52 3209.23
3209.25 3216.72 3225.83 3240.54 3259.20

RD5

=====

2.18 10.62 18.30 19.34 20.76 24.07 29.08 31.77 38.31
41.41 45.59 48.49 55.26 64.38 70.27 73.41 79.61
82.96 95.73 105.75 107.71 115.98 127.16 132.96
145.95 150.05 158.04 166.09 168.23 175.52 176.92
178.80 183.58 190.05 200.58 206.81 216.92 219.68
224.14 234.41 236.56 240.84 244.02 252.69 257.35
258.56 273.96 275.18 280.35 285.81 288.33 296.55
304.68 307.79 319.35 335.45 339.41 351.06 357.43
367.67 383.94 386.55 389.41 393.81 403.89 421.78
426.30 431.65 434.95 452.02 456.72 467.28 471.05
493.21 499.52 510.25 523.89 548.28 556.68 559.18
560.78 566.78 601.67 615.50 627.57 628.43 652.17
658.08 659.83 662.60 671.06 677.00 681.74 688.06
694.92 712.07 721.76 737.19 754.61 759.58 762.01
765.76 766.71 779.54 782.00 783.01 792.92 794.11
821.47 830.88 847.33 856.87 858.21 861.23 866.21
867.82 870.69 877.78 894.01 908.29 920.49 921.46
925.82 928.02 945.07 954.71 964.92 972.54 982.91
987.61 990.58 992.55 995.41 1003.81 1004.71
1010.50 1014.08 1017.43 1026.01 1034.77 1038.78
1041.29 1042.41 1044.76 1045.82 1055.42 1057.03
1077.48 1085.20 1091.95 1100.37 1115.94 1129.69
1135.30 1137.33 1171.65 1179.71 1182.26 1183.18
1187.35 1205.73 1213.71 1218.26 1223.07 1225.79
1235.68 1242.30 1245.86 1252.80 1273.02 1273.87
1279.13 1293.44 1302.59 1310.09 1317.57 1318.84
1324.87 1329.41 1349.22 1352.74 1356.84 1363.56
1372.64 1383.86 1389.17 1392.92 1398.89 1406.36
1412.82 1414.80 1421.90 1422.75 1426.55 1427.52
1434.29 1434.85 1438.32 1443.63 1448.79 1451.46
1454.10 1456.35 1459.41 1463.11 1465.88 1469.38
1470.62 1472.58 1473.22 1476.13 1476.62 1480.93
1495.87 1507.40 1517.48 1529.99 1538.74 1543.24
1597.83 1616.79 1625.92 1644.02 1653.22 1663.22
1788.47 3019.85 3021.98 3023.28 3025.73 3026.60
3031.73 3037.55 3037.59 3041.44 3058.49 3068.96
3086.89 3110.84 3111.71 3113.23 3115.13 3116.00
3116.23 3116.79 3118.66 3119.97 3122.06 3123.27
3127.07 3130.44 3130.85 3141.08 3153.11 3161.02
3162.73 3163.31 3184.63 3187.92 3193.28 3193.89
3196.65 3204.71 3207.75 3212.87 3221.79 3224.03
3225.58 3258.63 3273.35

=====

^RD5-TS1

=====

-224.93 2.23 11.37 17.05 22.10 24.99 33.40 37.31
39.35 42.75 44.52 48.67 55.04 60.06 60.88 76.17
85.69 87.07 96.46 98.10 104.06 117.12 119.92 137.54
143.36 159.56 163.89 172.73 174.75 178.51 179.23
185.31 192.58 195.57 205.05 208.53 214.44 216.19
224.76 229.32 231.40 235.84 240.63 250.02 252.12
254.13 256.58 273.40 276.10 279.99 282.57 285.66
299.22 301.72 311.55 325.93 339.10 348.64 352.39
364.76 374.65 388.47 392.47 396.75 397.80 415.62
423.64 430.33 432.09 444.60 455.28 460.75 474.49
480.60 490.75 505.98 506.96 545.27 558.72 561.77
562.63 571.82 602.22 614.82 624.71 628.99 647.67

653.89 660.80 665.31 669.83 682.18 686.18 691.61
697.28 712.95 732.20 735.57 736.56 760.75 762.65
763.42 770.85 778.57 779.56 781.22 794.20 797.19
817.38 821.48 836.04 857.81 859.82 863.87 865.86
868.71 880.08 881.51 888.80 902.58 916.73 918.09
926.39 927.41 945.51 947.02 956.00 958.78 968.42
973.45 983.31 990.75 999.20 999.77 1006.90 1011.98
1016.50 1019.98 1021.59 1023.28 1033.38 1039.50
1042.07 1044.11 1046.19 1047.31 1055.08 1060.34
1076.19 1082.84 1106.79 1110.40 1127.68 1133.02
1135.09 1170.66 1178.30 1182.01 1187.62 1188.16
1198.81 1207.42 1215.40 1223.57 1224.37 1235.76
1244.06 1251.70 1253.09 1277.29 1279.15 1284.71
1297.82 1304.57 1309.57 1311.91 1318.29 1325.45
1330.88 1346.18 1347.40 1356.86 1364.11 1366.91
1381.82 1388.76 1390.84 1394.86 1404.54 1411.79
1414.12 1423.23 1427.43 1427.96 1428.65 1434.66
1436.88 1437.22 1444.30 1446.36 1448.95 1452.76
1458.24 1458.87 1460.82 1462.24 1468.29 1470.08
1472.39 1474.56 1476.46 1477.41 1481.88 1495.29
1511.90 1515.27 1538.47 1540.76 1557.95 1591.10
1595.19 1628.67 1648.20 1650.01 1656.63 1779.00
3004.21 3006.85 3007.93 3009.48 3026.59 3029.66
3036.15 3037.35 3050.26 3073.99 3082.51 3095.58
3095.96 3097.68 3100.89 3102.68 3103.21 3108.82
3110.84 3116.81 3117.92 3120.31 3120.73 3130.48
3139.85 3144.01 3146.47 3158.70 3175.98 3178.48
3181.24 3182.84 3184.26 3189.24 3191.70 3200.47
3201.85 3205.96 3208.11 3215.13 3217.44 3220.43
3266.48 3326.86

=====

^RD5-TS3

=====

-264.55 9.77 14.32 20.52 21.56 29.67 31.37 34.07
36.82 43.92 47.92 48.88 55.89 61.85 69.87 72.50
81.01 84.66 88.01 96.69 103.11 109.70 118.38 131.04
134.05 144.89 149.84 151.32 157.17 166.60 171.06
175.63 177.99 188.53 195.48 202.55 204.84 210.70
217.21 220.34 227.66 236.15 238.18 244.07 250.95
255.25 258.60 272.03 276.92 281.46 284.76 286.32
301.72 303.37 309.05 319.22 325.97 332.67 349.67
355.18 368.02 382.68 390.61 395.92 398.97 418.14
420.93 425.64 427.70 434.41 440.03 467.66 476.07
487.96 490.05 505.17 507.69 539.31 544.38 558.34
562.64 572.36 601.56 614.35 625.37 630.32 638.48
654.13 659.67 662.51 672.43 677.51 684.16 686.60
695.64 712.69 725.05 731.48 736.00 753.59 759.62
761.20 765.28 769.15 772.80 777.39 789.60 795.36
818.70 822.53 838.07 843.64 852.20 861.45 863.09
864.13 873.81 877.73 886.67 916.10 918.48 922.54
925.62 926.30 936.85 946.04 956.23 962.41 969.83
973.69 986.05 991.08 992.61 996.96 998.24 1002.55
1013.15 1016.97 1018.07 1019.34 1033.61 1035.72
1039.71 1042.94 1043.87 1047.21 1059.24 1060.45
1077.15 1084.56 1113.06 1114.78 1134.02 1135.97
1139.91 1170.10 1178.58 1185.64 1186.95 1191.48
1205.98 1207.64 1211.07 1214.46 1223.49 1232.07
1244.71 1245.88 1255.65 1277.32 1277.84 1284.01
1300.90 1311.99 1317.96 1321.04 1324.56 1329.24

1342.52 1347.10 1350.76 1362.92 1366.47 1370.54
 1382.54 1389.42 1392.30 1399.88 1406.26 1414.18
 1416.90 1420.71 1424.03 1425.85 1429.39 1432.14
 1437.64 1442.15 1446.69 1448.88 1453.88 1458.02
 1461.30 1463.96 1466.09 1468.33 1468.95 1472.00
 1472.91 1474.11 1477.88 1481.56 1483.57 1494.47
 1512.25 1515.80 1537.18 1537.95 1543.48 1578.09
 1591.39 1630.23 1650.20 1651.24 1659.31 1787.97
 3021.51 3025.41 3026.49 3028.51 3028.55 3030.80
 3035.48 3040.74 3054.05 3066.52 3069.58 3101.94
 3108.38 3112.01 3114.30 3114.90 3116.69 3117.18
 3121.90 3122.57 3123.16 3125.29 3127.93 3129.84
 3130.55 3141.04 3141.42 3159.82 3170.90 3176.49
 3183.24 3186.81 3189.93 3195.11 3199.19 3201.32
 3202.47 3208.30 3208.88 3218.27 3219.61 3221.90
 3264.88 3332.37

=====

R_{D6}

=====

5.58 11.61 15.28 16.50 20.16 24.97 28.77 29.37 36.15
 40.76 44.47 53.80 60.62 69.46 81.40 82.91 88.55
 95.77 103.35 106.33 112.65 123.95 127.53 137.25
 146.10 149.87 151.51 156.77 161.77 167.36 175.12
 184.70 190.03 201.19 202.10 209.11 212.47 222.33
 226.85 230.34 236.92 237.98 245.54 251.23 260.60
 266.40 270.47 275.30 281.42 281.90 283.90 293.70
 297.87 318.93 326.56 337.39 339.18 347.95 360.33
 364.17 390.67 394.84 398.36 398.74 416.78 425.68
 428.02 430.88 446.48 448.13 469.16 473.23 490.99
 498.60 511.70 517.62 543.68 544.62 561.21 564.51
 574.51 579.00 602.79 614.17 624.80 629.41 646.03
 660.67 667.44 676.93 680.72 683.86 690.28 697.90
 710.53 716.78 731.02 735.19 753.74 762.30 763.59
 770.44 774.47 778.22 789.71 792.19 796.69 809.42
 821.99 828.31 857.48 861.74 864.56 866.39 872.88
 879.53 881.93 891.11 898.60 909.13 924.27 924.71
 936.72 946.49 955.71 962.56 965.55 971.37 980.75
 988.37 993.31 1001.45 1008.03 1010.70 1017.04
 1021.44 1029.80 1032.25 1034.14 1037.16 1038.31
 1042.60 1044.27 1045.84 1053.73 1058.42 1081.06
 1082.97 1097.82 1109.39 1121.84 1130.73 1133.92
 1151.19 1162.45 1174.67 1177.00 1181.48 1184.30
 1206.45 1209.81 1211.75 1222.85 1233.00 1243.50
 1250.50 1251.48 1255.36 1274.83 1280.86 1281.68
 1287.85 1300.93 1303.96 1318.65 1323.31 1330.32
 1335.79 1342.80 1345.11 1354.29 1362.86 1369.24
 1381.23 1385.99 1389.52 1390.42 1400.56 1406.98
 1410.51 1412.72 1420.52 1422.38 1424.12 1427.50
 1428.30 1433.12 1435.69 1439.82 1443.14 1448.66
 1451.75 1456.67 1458.28 1460.63 1463.97 1465.60
 1467.94 1470.73 1473.94 1475.71 1480.28 1487.04
 1492.12 1495.52 1511.35 1513.77 1538.86 1563.71
 1577.16 1591.24 1626.26 1649.42 1654.42 1656.78
 1764.62 2847.60 2967.07 2997.02 3022.60 3023.87
 3025.84 3027.34 3028.00 3033.69 3036.92 3067.12
 3071.28 3094.15 3099.83 3105.79 3110.33 3110.75
 3112.18 3112.30 3114.17 3119.24 3120.94 3121.11
 3121.45 3123.72 3127.77 3140.90 3141.94 3142.66
 3155.23 3164.32 3171.98 3181.99 3185.41 3188.06

3190.81 3196.18 3197.43 3200.33 3203.17 3204.86
 3211.19 3214.14 3262.69

=====

R_{D6-TS}

=====

-259.66 8.36 15.49 20.32 23.47 26.02 32.52 36.09
 38.06 41.79 49.02 50.25 55.79 64.87 78.46 80.66
 85.57 86.70 97.21 101.28 104.57 112.23 114.72
 142.03 144.14 146.58 148.66 153.54 155.18 164.49
 171.91 176.67 180.93 186.68 203.60 204.35 210.80
 215.21 220.91 223.01 225.41 231.38 243.61 247.52
 249.46 254.85 258.68 273.42 278.04 279.77 283.85
 284.28 297.18 299.39 313.26 321.15 328.72 339.41
 351.68 359.02 372.48 382.36 393.40 397.09 406.18
 413.15 419.99 424.84 428.52 441.29 453.61 463.25
 473.20 480.80 490.14 491.36 509.16 541.03 553.87
 560.99 563.34 572.27 602.75 614.41 620.32 629.05
 636.41 659.90 661.24 668.54 676.94 681.73 685.41
 689.63 692.06 701.78 711.86 734.81 736.58 758.89
 762.58 763.40 773.70 775.19 775.96 791.39 795.53
 814.02 821.24 831.52 850.13 853.61 862.76 863.01
 865.03 869.63 879.00 884.81 886.62 904.19 907.44
 917.35 922.26 925.62 945.95 953.15 964.98 970.52
 971.19 983.45 989.87 992.59 996.44 1008.59 1012.09
 1018.07 1020.52 1023.34 1032.73 1034.29 1035.90
 1041.18 1041.92 1044.13 1045.56 1051.53 1059.40
 1077.63 1083.04 1099.93 1109.67 1135.39 1136.80
 1142.57 1162.47 1167.92 1172.52 1177.64 1181.94
 1192.67 1197.17 1206.63 1211.53 1216.32 1224.31
 1234.88 1243.49 1248.98 1252.02 1276.85 1278.62
 1284.76 1297.92 1312.59 1317.10 1319.68 1324.76
 1331.22 1335.45 1344.52 1354.60 1361.53 1364.34
 1381.05 1387.66 1388.72 1391.11 1397.10 1404.97
 1409.07 1412.42 1420.83 1424.66 428.29 1429.90
 1431.65 1436.14 1437.46 1443.78 1448.47 1453.37
 1457.23 1459.11 1459.72 1462.11 1465.43 1467.87
 1470.58 1473.22 1474.11 1474.79 1480.80 1481.24
 1496.82 1510.42 1511.27 1519.13 1540.92 1569.49
 1586.33 1590.41 1628.69 1654.57 1657.43 1658.41
 1767.58 3021.71 3024.53 3026.42 3027.92 3028.18
 3030.17 3036.40 3038.67 3054.90 3074.72 3097.05
 3108.62 3109.26 3110.17 3113.91 3114.57 3116.47
 3118.71 3122.13 3123.68 3124.33 3124.75 3125.85
 3130.21 3139.92 3145.61 3160.91 3162.88 3163.48
 3178.04 3181.39 3182.71 3186.31 3191.96 3197.73
 3199.88 3201.53 3205.99 3212.69 3215.14 3216.20
 3231.66 3271.95 3274.57

=====

R_{D7}

=====

10.84 14.92 18.25 27.83 32.18 32.43 39.74 41.60
 43.72 44.83 47.41 53.33 59.69 71.18 74.34 77.95
 83.29 102.06 105.01 112.49 120.61 125.56 132.71
 141.28 149.65 161.08 176.17 176.87 186.07 186.95
 190.20 200.16 202.77 207.12 210.95 215.22 217.70
 222.98 225.50 241.06 243.05 248.95 250.59 254.24

259.04 267.08 272.00 273.79 282.12 290.76 302.02
305.72 312.38 321.27 326.92 328.76 344.47 348.61
353.43 365.47 388.76 391.04 394.11 396.50 415.49
427.41 431.62 442.98 454.19 460.40 475.66 481.66
489.15 500.59 520.13 538.44 542.93 558.25 561.10
565.89 581.37 596.46 605.14 611.36 625.29 628.56
652.20 659.47 666.64 670.09 681.83 687.40 690.75
697.07 709.79 720.86 728.28 735.16 755.92 762.95
765.74 776.04 779.67 790.74 795.26 803.36 806.50
822.14 826.64 837.70 856.20 857.67 867.17 871.05
876.71 879.53 883.31 899.67 900.64 924.60 924.93
925.41 938.42 945.94 956.50 967.66 975.39 983.43
987.10 987.59 1004.23 1005.14 1010.64 1012.14
1017.20 1020.41 1027.92 1031.23 1038.70 1039.65
1042.77 1043.47 1047.94 1054.31 1059.90 1063.32
1075.58 1105.23 1116.20 1134.34 1136.92 1138.88
1151.57 1158.49 1164.65 1179.81 1187.30 1188.40
1208.07 1213.89 1218.42 1220.62 1226.90 1240.54
1243.34 1249.93 1253.04 1271.83 1276.42 1278.15
1283.81 1285.75 1299.99 1317.28 1322.21 1326.85
1332.05 1341.47 1349.11 1350.42 1368.65 1372.79
1378.09 1380.68 1387.70 1392.99 1393.68 1405.12
1407.88 1414.72 1425.47 1429.38 1430.66 1432.35
1436.13 1441.38 1443.95 1447.19 1449.21 1452.59
1455.09 1457.66 1460.93 1466.35 1468.64 1469.06
1475.09 1476.03 1477.11 1478.30 1480.09 1480.55
1486.14 1498.15 1510.12 1519.74 1533.09 1542.09
1542.99 1581.52 1617.40 1629.56 1654.92 1657.49
1671.08 1863.57 2991.84 2997.38 2999.79 3002.61
3005.10 3011.81 3022.97 3026.41 3026.86 3030.74
3035.83 3072.97 3077.03 3082.68 3082.88 3085.77
3087.53 3096.93 3097.53 3100.83 3100.98 3109.19
3113.23 3118.99 3120.14 3125.57 3125.82 3133.82
3149.78 3159.90 3167.13 3181.41 3188.20 3188.53
3196.05 3199.93 3201.57 3208.03 3210.64 3210.71
3214.76 3218.23 3234.12 3466.59

=====

R_D10

=====

5.44 13.21 16.92 19.66 23.28 27.13 30.18 36.50 38.83
45.26 45.73 52.91 57.30 62.45 70.67 82.10 84.16
94.44 97.26 100.10 111.75 126.60 130.96 137.31
146.47 147.25 151.59 154.30 157.46 164.55 168.10
180.38 183.64 191.62 195.14 208.27 210.51 214.50
219.73 228.32 230.28 233.27 241.98 245.57 250.42
256.71 263.21 277.92 280.08 281.82 288.43 292.60
299.86 305.34 317.92 327.60 334.93 349.44 351.80
359.50 378.29 386.50 390.90 399.00 417.90 427.22
427.97 430.95 447.11 451.54 464.62 470.89 492.00
503.07 510.46 521.03 542.33 544.67 561.57 563.87
574.63 608.60 614.98 616.48 623.44 629.72 660.22
662.09 665.26 666.85 685.05 686.92 698.13 701.47
712.07 715.47 732.08 733.45 755.53 764.68 767.48
770.89 774.71 775.30 791.93 794.96 796.30 818.18
820.24 823.39 846.39 862.97 867.97 870.13 871.50
878.11 881.90 882.24 884.16 898.72 920.67 923.79
925.51 943.36 946.44 953.91 965.43 970.97 973.37
990.03 992.42 993.57 1000.90 1011.59 1017.18
1018.15 1019.34 1027.20 1033.00 1035.41 1037.58

1040.51 1042.62 1044.38 1046.00 1053.11 1058.55
1059.04 1080.70 1093.16 1109.22 1126.02 1129.83
1134.55 1163.78 1164.92 1176.84 1180.60 1188.29
1205.24 1209.63 1210.97 1212.25 1224.75 1232.10
1245.33 1248.33 1253.54 1271.65 1280.50 1283.02
1287.24 1288.66 1299.04 1307.37 1317.86 1318.38
1323.34 1332.28 1341.02 1345.46 1362.79 1381.71
1386.20 1388.17 1390.02 1399.85 1406.08 1410.54
1412.96 1416.56 1418.06 1420.96 1422.48 1430.09
1430.59 1434.28 1438.63 1448.50 1452.22 1455.20
1460.03 1460.43 1461.88 1465.10 1466.10 1468.49
1470.41 1474.87 1475.47 1479.42 1482.21 1489.86
1497.42 1512.17 1513.67 1541.60 1558.60 1562.30
1590.52 1594.82 1629.04 1655.42 1658.92 1679.14
1777.96 3026.08 3026.92 3027.24 3027.57 3030.69
3032.05 3036.41 3038.02 3066.59 3066.92 3088.58
3095.14 3109.44 3111.23 3113.28 3114.43 3116.98
3122.72 3123.09 3123.22 3124.65 3124.99 3126.73
3127.70 3131.99 3137.64 3143.17 3148.22 3160.12
3168.25 3178.73 3179.99 3184.39 3185.63 3186.26
3188.23 3194.06 3196.11 3204.20 3209.85 3213.40
3213.78 3214.45 3264.38

=====

R_D10-TS

=====

-152.76 4.65 9.11 12.17 15.22 20.30 21.80 30.24
34.06 36.46 37.17 47.03 55.09 64.72 70.26 78.91
85.02 86.35 95.86 102.45 106.33 108.35 116.70
143.84 150.34 152.72 155.55 165.47 167.24 174.87
180.36 182.75 190.16 194.41 200.94 206.56 210.88
215.41 220.93 223.17 227.81 233.09 236.26 245.74
249.27 256.49 259.69 265.92 274.32 278.46 282.16
283.79 297.41 298.29 316.22 317.46 325.58 338.19
352.25 362.81 377.03 381.38 391.17 398.68 409.73
415.12 421.99 425.58 428.17 436.77 448.01 471.12
473.10 490.24 491.17 493.39 509.21 541.33 554.98
561.95 564.92 571.68 596.51 614.84 621.15 629.42
635.80 652.34 661.28 665.70 674.04 683.05 683.63
687.21 696.17 711.00 712.17 731.98 741.64 763.29
763.79 767.58 770.54 776.75 781.30 791.53 797.12
808.76 820.39 821.98 830.11 852.52 857.78 863.04
863.60 866.37 869.08 876.80 885.10 888.07 908.10
917.13 923.43 925.90 945.06 951.55 953.88 966.61
968.42 969.04 992.55 996.53 996.93 1009.04 1011.99
1016.52 1018.13 1020.42 1021.34 1033.28 1036.16
1039.65 1041.34 1044.29 1045.53 1051.44 1058.74
1061.67 1080.66 1101.99 1109.79 1119.52 1128.92
1134.60 1140.06 1170.20 1176.28 1177.22 1182.11
1188.29 1188.59 1204.96 1211.59 1214.41 1225.21
1237.85 1242.78 1245.35 1254.48 1275.35 1278.08
1284.10 1290.37 1300.55 1307.31 1318.13 1318.49
1326.18 1329.25 1345.84 1349.92 1363.21 1368.91
1383.65 1386.69 1387.88 1389.60 1400.76 1405.22
1410.63 1411.89 1417.02 1420.48 1424.34 1427.04
1430.94 1432.00 1438.77 1443.71 1448.64 1454.90
1455.25 1459.50 1461.28 1468.11 1470.14 1470.40
1471.57 1472.64 1475.96 1476.73 1482.38 1484.34
1496.46 1512.22 1513.63 1531.12 1541.38 1565.74
1585.75 1591.62 1629.26 1653.13 1655.56 1659.42

1746.97 2976.85 3005.69 3021.03 3022.78 3025.44
3026.75 3027.63 3032.66 3038.43 3072.22 3082.53
3103.89 3107.77 3108.72 3111.22 3112.10 3116.49
3117.62 3119.99 3122.87 3123.48 3129.44 3130.49
3136.16 3144.50 3150.25 3163.18 3165.04 3167.19
3172.82 3177.37 3181.80 3185.44 3185.76 3189.45
3196.37 3197.22 3206.64 3206.86 3211.10 3213.81
3237.32 3255.67 3266.04

=====

^RD11

=====

7.76 10.48 13.27 14.16 21.69 25.43 28.66 33.69 36.88
37.49 41.73 56.42 59.15 66.09 74.52 83.36 86.23
86.98 96.52 105.31 109.34 117.82 122.01 139.75
144.11 145.01 157.02 158.06 162.83 169.45 175.69
183.06 188.89 196.79 205.24 209.78 210.41 222.97
223.98 231.38 238.63 239.51 249.71 255.06 261.76
264.47 266.58 274.69 281.98 282.61 283.66 297.30
306.51 318.19 322.40 335.65 344.80 352.98 355.35
369.46 382.20 394.42 395.64 398.44 414.86 422.10
425.92 428.31 446.43 448.29 472.05 481.03 489.93
491.32 494.39 509.19 525.38 541.44 561.80 564.03
572.10 584.01 611.14 614.84 621.75 629.42 654.37
661.20 665.15 667.90 680.75 682.44 687.48 693.97
702.05 712.25 732.72 738.41 762.48 763.24 769.25
776.09 776.70 790.94 792.06 797.00 807.48 820.29
821.84 839.70 855.06 863.40 863.60 864.95 868.06
877.68 884.89 894.66 902.29 908.37 910.91 923.71
926.07 938.13 944.79 951.39 965.42 969.33 969.93
982.34 992.76 996.09 1011.93 1018.57 1020.22
1024.56 1033.11 1035.68 1035.78 1037.93 1040.09
1041.68 1044.25 1045.36 1054.23 1058.70 1079.88
1084.04 1091.57 1099.20 1109.53 1124.46 1134.54
1170.14 1175.38 1177.63 1181.45 1182.53 1189.40
1205.14 1211.56 1218.82 1225.13 1227.85 1242.36
1245.41 1249.60 1254.68 1272.82 1279.17 1279.66
1286.10 1298.00 1304.29 1318.62 1319.30 1321.54
1326.41 1327.57 1345.77 1347.01 1358.04 1363.24
1383.00 1383.53 1386.97 1387.97 1399.68 1403.67
1407.50 1412.09 1420.59 1423.38 1424.52 1430.90
1432.55 1435.76 1436.75 1440.70 1445.23 1452.56
1454.64 1455.28 1459.43 1461.44 1465.53 1467.63
1470.66 1471.90 1472.79 1476.60 1480.83 1483.48
1495.88 1497.12 1512.52 1513.45 1541.67 1563.64
1571.61 1591.54 1629.18 1653.36 1655.73 1659.66
1758.33 3002.94 3009.86 3022.10 3023.08 3027.13
3027.48 3028.68 3033.86 3037.59 3063.05 3070.05
3070.49 3072.83 3105.24 3107.40 3110.51 3110.60
3112.63 3116.13 3116.84 3118.78 3123.09 3124.40
3126.83 3129.34 3130.96 3136.24 3141.38 3142.21
3153.89 3163.70 3171.02 3180.24 3185.10 3188.11
3188.29 3194.87 3196.61 3200.01 3203.50 3206.32
3210.77 3213.28 3272.33

=====

^RD12

=====

7.65 11.63 16.48 19.07 22.81 26.67 28.58 35.22 35.96
38.46 49.29 51.02 54.48 64.10 69.50 73.82 85.53
89.37 107.00 117.53 122.48 126.54 133.55 137.44
145.53 150.82 154.38 158.78 164.08 172.37 174.72
179.64 183.94 189.48 191.61 209.65 211.12 218.44
222.35 228.43 237.63 243.82 244.58 253.85 259.23
267.06 270.34 274.11 284.50 289.02 293.12 299.78
308.66 325.78 326.40 326.90 332.23 346.06 351.56
391.75 392.21 395.40 397.58 416.48 425.27 429.76
439.27 444.45 448.85 465.24 476.83 483.97 490.14
502.64 513.78 542.36 543.34 559.27 567.10 569.77
574.47 585.64 610.58 611.25 625.54 628.58 648.97
658.78 668.23 676.61 679.94 683.73 688.67 697.36
710.01 721.20 730.29 735.68 754.72 756.69 767.78
773.35 779.27 791.23 793.05 802.16 811.86 823.95
824.67 841.32 855.87 859.82 860.98 867.06 876.15
877.71 882.98 898.75 900.36 913.66 924.88 928.31
939.62 946.31 949.19 956.20 971.01 972.61 982.57
986.27 987.27 1004.09 1008.09 1011.01 1018.48
1021.77 1028.70 1039.02 1039.96 1040.82 1043.22
1044.46 1048.07 1060.05 062.60 1067.99 1077.66
1109.82 1116.46 1125.71 1133.86 1138.05 1139.56
1164.40 1174.64 1181.14 1181.38 1186.14 1209.20
1210.40 1215.24 1218.15 1222.64 1240.36 1243.26
1250.68 1253.95 1268.84 1271.12 1272.59 1278.85
1289.15 1310.96 1313.86 1323.25 1326.52 1328.32
1338.69 1349.90 1354.97 1365.15 1368.97 1378.76
1380.63 1390.53 1392.56 1393.33 1403.35 1406.59
1414.38 1420.53 1425.50 1426.94 1431.12 1432.42
1434.05 1443.01 1443.95 1450.38 1452.27 1454.40
1457.39 1460.34 1461.66 1463.42 1465.07 1469.41
1473.30 1474.66 1476.89 1478.12 1479.49 1486.35
1497.29 1509.79 1520.79 1535.43 1540.56 1544.15
1579.39 1622.97 1628.84 1655.26 1658.13 1669.07
1871.26 3008.79 3017.53 3020.44 3020.79 3021.75
3026.75 3030.39 3031.54 3035.58 3037.82 3084.39
3089.14 3095.56 3103.38 3109.60 3111.47 3111.77
3112.73 3114.66 3115.37 3115.72 3117.59 3118.45
3121.50 3122.52 3131.03 3134.14 3140.13 3164.22
3168.17 3171.90 3181.61 3183.75 3189.46 3190.73
3199.02 3199.92 3200.31 3207.83 3210.23 3215.44
3220.83 3237.32 3463.81

=====

^RD13

=====

7.43 23.33 26.17 30.72 35.09 45.21 48.53 52.35 59.93
65.19 69.77 72.07 74.17 76.81 83.68 88.03 112.04
114.60 124.67 146.16 147.11 156.66 167.11 173.50
183.55 184.48 195.90 201.62 207.89 216.02 219.27
223.50 232.87 239.57 256.38 262.59 263.67 272.95
281.68 284.83 294.30 300.56 315.31 322.77 327.66
329.08 342.98 353.01 384.36 388.89 390.74 394.24
411.82 420.98 422.84 430.32 438.72 450.30 459.05
481.59 483.67 489.23 501.78 513.87 543.90 546.70
559.92 565.15 569.25 588.08 597.92 611.17 626.83
628.05 657.39 660.74 671.58 685.14 701.78 711.35
721.95 730.88 756.48 759.90 777.39 778.01 779.94
790.49 796.37 799.12 813.63 822.46 826.36 856.30
865.55 879.24 881.62 883.82 903.05 907.97 925.52

926.35 931.68 943.43 945.62 957.05 962.16 971.68
973.29 986.67 988.97 1004.06 1012.30 1015.24
1018.29 1023.17 1028.25 1029.53 1033.89 1037.91
1040.15 1041.38 1043.81 1044.65 1047.02 1054.93
1059.18 1073.50 1080.09 1101.76 1105.32 1113.06
1116.50 1121.32 1136.99 1165.54 1173.20 1180.81
1186.27 1194.70 1207.60 1211.54 1211.90 1214.82
1219.29 1241.90 1242.74 1247.25 1255.59 1257.22
1272.00 1307.36 1308.39 1314.77 1323.23 1329.85
1333.15 1346.71 1363.58 1367.97 1370.58 1381.82
1389.45 1390.81 1396.85 1406.41 1407.29 1413.76

1415.33 1434.81 1448.97 1449.58 1456.90 1459.38
1460.37 1461.36 1462.23 1464.64 1469.32 1472.51
1474.13 1477.31 1478.08 1483.73 1484.18 1500.33
1508.56 1518.63 1534.27 1545.29 1579.89 1630.64
1653.83 1657.98 1672.46 1712.86 1780.25 1869.18
3030.14 3032.69 3040.02 3049.16 3051.40 3055.02
3063.64 3092.06 3112.12 3115.14 3116.12 3120.15
3120.98 3128.32 3129.79 3137.71 3145.89 3148.68
3150.94 3155.25 3160.16 3168.19 3171.64 3179.55
3185.95 3191.55 3193.59 3195.73 3197.37 3200.47
3201.72 3205.46 3214.24 3217.00 3242.49 3470.82

23. Supplementary Information References

- (1) Lu, D.-F.; Zhu, C.-L.; Sears, J. D.; Xu, H. Iron(II)-Catalyzed Intermolecular Aminofluorination of Unfunctionalized Olefins Using Fluoride Ion. *J. Am. Chem. Soc.* **2016**, *138* (35), 11360-11367.
- (2) Masruri; Willis, A. C.; McLeod, M. D. Osmium-Catalyzed Vicinal Oxyamination of Alkenes by N-(4-Toluenesulfonyloxy)Carbamates. *J. Org. Chem.* **2012**, *77* (19), 8480-8491.
- (3) Jing, C.; Jones, B. T.; Adams, R. J.; Bower, J. F. Cyclopropane-Fused N-Heterocycles Via Aza-Heck-Triggered C(sp³)-H Functionalization Cascades. *J. Am. Chem. Soc.* **2022**, *144* (37), 16749-16754.
- (4) Falk, E.; Makai, S.; Delcaillau, T.; Gürtler, L.; Morandi, B. Design and Scalable Synthesis of N-Alkylhydroxylamine Reagents for the Direct Iron-Catalyzed Installation of Medicinally Relevant Amines**. *Angew. Chem. Int. Ed.* **2020**, *59* (47), 21064-21071.
- (5) Wang, Y.-F.; Gao, Y.-R.; Mao, S.; Zhang, Y.-L.; Guo, D.-D.; Yan, Z.-L.; Guo, S.-H.; Wang, Y.-Q. Wacker-Type Oxidation and Dehydrogenation of Terminal Olefins Using Molecular Oxygen as the Sole Oxidant without Adding Ligand. *Org. Lett.* **2014**, *16* (6), 1610-1613.
- (6) Ranu, B. C.; Banerjee, S.; Adak, L. Regioselective Cross-Coupling of Allylindium Reagents with Activated Benzylic Bromides—A Simple and Efficient Procedure for the Synthesis of Terminal Alkenes. *Tetrahedron Lett.* **2007**, *48* (41), 7374-7379.
- (7) Zhang, Q.; Wang, S.; Zhang, Q.; Xiong, T.; Zhang, Q. Radical Addition-Triggered Remote Migratory Isomerization of Unactivated Alkenes to Difluoromethylene-Containing Alkenes Enabled by Bimetallic Catalysis. *ACS Catal.* **2022**, *12* (1), 527-535.
- (8) Lu, Y.; Nakatsuji, H.; Okumura, Y.; Yao, L.; Ishihara, K. Enantioselective Halo-Oxy- and Halo-Azacyclizations Induced by Chiral Amidophosphate Catalysts and Halo-Lewis Acids. *J. Am. Chem. Soc.* **2018**, *140* (19), 6039-6043.
- (9) Lin, S.; Song, C.-X.; Cai, G.-X.; Wang, W.-H.; Shi, Z.-J. Intra/Intermolecular Direct Allylic Alkylation Via Pd(II)-Catalyzed Allylic C-H Activation. *J. Am. Chem. Soc.* **2008**, *130* (39), 12901-12903.
- (10) Liu, R.; Lu, Z.-H.; Hu, X.-H.; Li, J.-L.; Yang, X.-J. Monocarboxylation and Intramolecular Coupling of Butenylated Arenes Via Palladium-Catalyzed C-H Activation Process. *Org. Lett.* **2015**, *17* (6), 1489-1492.
- (11) Smith, M. J. S.; Tu, W.; Robertson, C. M.; Bower, J. F. Stereospecific Aminative Cyclizations Triggered by Intermolecular Aza-Prilezhaev Alkene Aziridination. *Angew. Chem. Int. Ed.* **2023**, *62* (48), e202312797.
- (12) Meng, Q.-Y.; Schirmer, T. E.; Katou, K.; König, B. Controllable Isomerization of Alkenes by Dual Visible-Light-Cobalt Catalysis. *Angew. Chem. Int. Ed.* **2019**, *58* (17), 5723-5728.
- (13) Murai, M.; Nishimura, K.; Takai, K. Palladium-Catalyzed Double-Bond Migration of Unsaturated Hydrocarbons Accelerated by Tantalum Chloride. *Chem. Commun.* **2019**, *55* (19), 2769-2772.
- (14) Semakul, N.; Jackson, K. E.; Paton, R. S.; Rovis, T. Heptamethylindenyl (Ind*) Enables Diastereoselective Benzamidation of Cyclopropenes Via Rh(III)-Catalyzed C-H Activation. *Chem. Sci.* **2017**, *8* (2), 1015-1020.
- (15) Heyboer, E. M. P.; Pullara, W. A.; Bacsá, J.; Blakey, S. B. Enantioselective 3,4-Oxyamination of 1,3-Dienes Enabled by an Electron-Rich Planar Chiral Rhodium Indenyl Catalyst. *Org. Lett.* **2025**, *27* (27), 7412-7416.
- (16) Gross, P.; Im, H.; Laws, D., III; Park, B.; Baik, M.-H.; Blakey, S. B. Enantioselective Aziridination of Unactivated Terminal Alkenes Using a Planar Chiral Rh(III) Indenyl Catalyst. *J. Am. Chem. Soc.* **2024**, *146* (2), 1447-1454.
- (17) Ye, C.-X.; Shen, X.; Chen, S.; Meggers, E. Stereocontrolled 1,3-Nitrogen Migration to Access Chiral α -Amino Acids. *Nat. Chem.* **2022**, *14* (5), 566-573.
- (18) Hilby, K. M.; Denmark, S. E. Lewis Base Catalyzed, Sulfenium Ion Initiated Enantioselective, Spiroketalization Cascade. *J. Org. Chem.* **2021**, *86* (21), 14250-14289.

- (19) Trost, B. M.; Yeh, V. S. C.; Ito, H.; Bremeyer, N. Effect of Ligand Structure on the Zinc-Catalyzed Henry Reaction. Asymmetric Syntheses of (–)-Denopamine and (–)-Arbutamine. *Org. Lett.* **2002**, *4* (16), 2621-2623.
- (20) Muranaka, K.; Ichikawa, S.; Matsuda, A. Development of the Carboxamide Protecting Group, 4-(Tert-Butyldimethylsiloxy)-2-Methoxybenzyl. *J. Org. Chem.* **2011**, *76* (22), 9278-9293.
- (21) Song, Z.; Jia, Y.; Zhang, D.; Wang, D. Dmap Mediated Efficient Construction of Functionalized Chromenes through One-Pot Reaction of Para-Quinone Methides with Allenates. *Eur. J. Org. Chem.* **2021**, *2021* (12), 1942-1948.
- (22) Kumar, A.; Li, Z.; Sharma, S. K.; Parmar, V. S.; Van der Eycken, E. V. Switching the Regioselectivity Via Indium(III) and Gold(I) Catalysis: A Post-Ugi Intramolecular Hydroarylation to Azepino- and Azocino-[C,D]Indolones. *Chem. Commun.* **2013**, *49* (60), 6803-6805.
- (23) Büyükdalı, N. N.; Aslan, N.; Gümüş, S.; Gümüş, A. Stereoselective Synthesis of Benzofuran and Benzothiophene Substituted Dihydropyran Derivatives Via Ring Closing Metathesis. *Tetrahedron: Asymmetry* **2016**, *27* (19), 954-959.
- (24) Wang, H.; Liu, C.-F.; Martin, R. T.; Gutierrez, O.; Koh, M. J. Directing-Group-Free Catalytic Dicarbofunctionalization of Unactivated Alkenes. *Nat. Chem.* **2022**, *14* (2), 188-195.
- (25) Webster, R.; Boyer, A.; Fleming, M. J.; Lautens, M. Practical Asymmetric Synthesis of Bioactive Aminotetralins from a Racemic Precursor Using a Regiodivergent Resolution. *Org. Lett.* **2010**, *12* (23), 5418-5421.
- (26) Williams, W. L.; Gutiérrez-Valencia, N. E.; Doyle, A. G. Branched-Selective Cross-Electrophile Coupling of 2-Alkyl Aziridines and (Hetero)Aryl Iodides Using Ti/Ni Catalysis. *J. Am. Chem. Soc.* **2023**, *145* (44), 24175-24183.
- (27) Seefeld, M. A.; Rouse, M. B.; Heerding, D. A.; Peace, S.; Yamashita, D. S.; McNulty, K. C. Preparation of Novel Heterocyclic Carboxamides as Inhibitors of AKT Activity. 2008.
- (28) Kohler, D. G.; Gockel, S. N.; Kennemur, J. L.; Waller, P. J.; Hull, K. L. Palladium-Catalysed Anti-Markovnikov Selective Oxidative Amination. *Nat. Chem.* **2018**, *10* (3), 333-340.
- (29) Xu, S.; Hirano, K.; Miura, M. Nickel-Catalyzed Regio- and Stereospecific C–H Coupling of Benzamides with Aziridines. *Org. Lett.* **2021**, *23* (14), 5471-5475.
- (30) Sasane, A. V.; Kuo, T.-C.; Cheng, M.-J.; Liu, R.-S. Gold-Catalyzed Rearrangement of α -Carbonyl Cyclopropanes to Form 3-(Cyclopenta-1,3-Dien-1-Ylmethyl)Oxindoles Via a Postulated 1,5-Enolate Shift. *Org. Lett.* **2022**, *24* (28), 5220-5225.
- (31) (a) Lee, C.; Yang, W.; Parr, R. G. Development of the Colle-Salvetti Correlation-Energy Formula into a Functional of the Electron Density. *Phys. Rev. B* **1988**, *37* (2), 785-789. (b) Grimme, S.; Antony, J.; Ehrlich, S.; Krieg, H. A Consistent and Accurate Ab Initio Parametrization of Density Functional Dispersion Correction (DFT-D) for the 94 Elements H-Pu. *J. Chem. Phys.* **2010**, *132* (15), 154104. (c) Neese, F. The Orca Program System. *Wiley Interdiscip. Rev. Comput. Mol. Sci.* **2012**, *2* (1), 73-78. (d) Parr, R. G. Density Functional Theory of Atoms and Molecules. In *Horizons of Quantum Chemistry*; Fukui, K., Pullman, B., Eds.; Springer Netherlands, 1980; pp 5-15. (e) Slater, J. C.; Phillips, J. C. Quantum Theory of Molecules and Solids Vol. 4: The Self-Consistent Field for Molecules and Solids. *Phys. Today* **1974**, *27* (12), 49-50.
- (32) Weigend, F.; Ahlrichs, R. Balanced Basis Sets of Split Valence, Triple Zeta Valence and Quadruple Zeta Valence Quality for H to Rn: Design and Assessment of Accuracy. *Phys. Chem. Chem. Phys.* **2005**, *7* (18), 3297-3305.
- (33) Marten, B.; Kim, K.; Cortis, C.; Friesner, R. A.; Murphy, R. B.; Ringnalda, M. N.; Sitkoff, D.; Honig, B. New Model for Calculation of Solvation Free Energies: Correction of Self-Consistent Reaction Field Continuum Dielectric Theory for Short-Range Hydrogen-Bonding Effects. *J. Phys. Chem.* **1996**, *100* (28), 11775-11788.

- (34) Neese, F.; Wennmohs, F.; Hansen, A.; Becker, U. Efficient, Approximate and Parallel Hartree–Fock and Hybrid DFT Calculations. A ‘Chain-of-Spheres’ Algorithm for the Hartree–Fock Exchange. *Chem. Phys.* **2009**, *356* (1), 98-109.
- (35) (a) te Velde, G.; Bickelhaupt, F. M.; Baerends, E. J.; Fonseca Guerra, C.; van Gisbergen, S. J. A.; Snijders, J. G.; Ziegler, T. Chemistry with ADF. *J. Comput. Chem.* **2001**, *22* (9), 931-967. (b) van Lenthe, E.; Ehlers, A.; Baerends, E.-J. Geometry Optimizations in the Zero Order Regular Approximation for Relativistic Effects. *J. Chem. Phys.* **1999**, *110* (18), 8943-8953.
- (36) (a) Bickelhaupt, F. M.; Houk, K. N. Analyzing Reaction Rates with the Distortion/Interaction-Activation Strain Model. *Angew. Chem. Int. Ed.* **2017**, *56* (34), 10070-10086. (b) Morokuma, K. Molecular Orbital Studies of Hydrogen Bonds. III. C=O···H–O Hydrogen Bond in H₂CO···H₂O and H₂CO···2H₂O. *J. Chem. Phys.* **1971**, *55* (3), 1236-1244.
- (37) Glendening, E. D.; Landis, C. R.; Weinhold, F. NBO 6.0: Natural Bond Orbital Analysis Program. *J. Comput. Chem.* **2013**, *34* (16), 1429-1437.
- (38) (a) Knizia, G.; Klein, J. E. M. N. Electron Flow in Reaction Mechanisms—Revealed from First Principles. *Angew. Chem. Int. Ed.* **2015**, *54* (18), 5518-5522. (b) Knizia, G. Intrinsic Atomic Orbitals: An Unbiased Bridge between Quantum Theory and Chemical Concepts. *J. Chem. Theory Comput.* **2013**, *9* (11), 4834-4843.
- (39) Falivene, L.; Cavallo, L.; Talarico, G. Buried Volume Analysis for Propene Polymerization Catalysis Promoted by Group 4 Metals: A Tool for Molecular Mass Prediction. *ACS Catal.* **2015**, *5* (11), 6815-6822.
- (40) CYLview, 1.0b; Legault, C. Y., Université de Sherbrooke, 2009 (<http://www.cylview.org>)

24. NMR, HPLC and SFC Data

2,2,2-trichloroethyl (pivaloyloxy)carbamate (**9**):

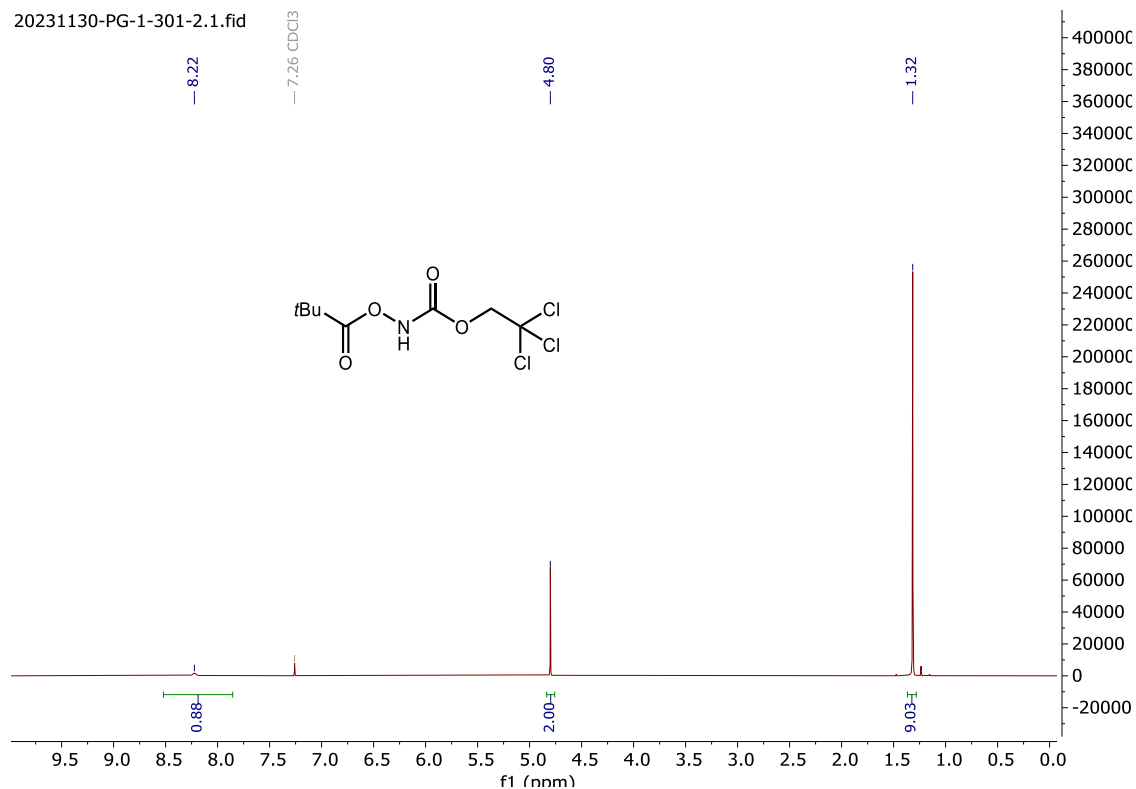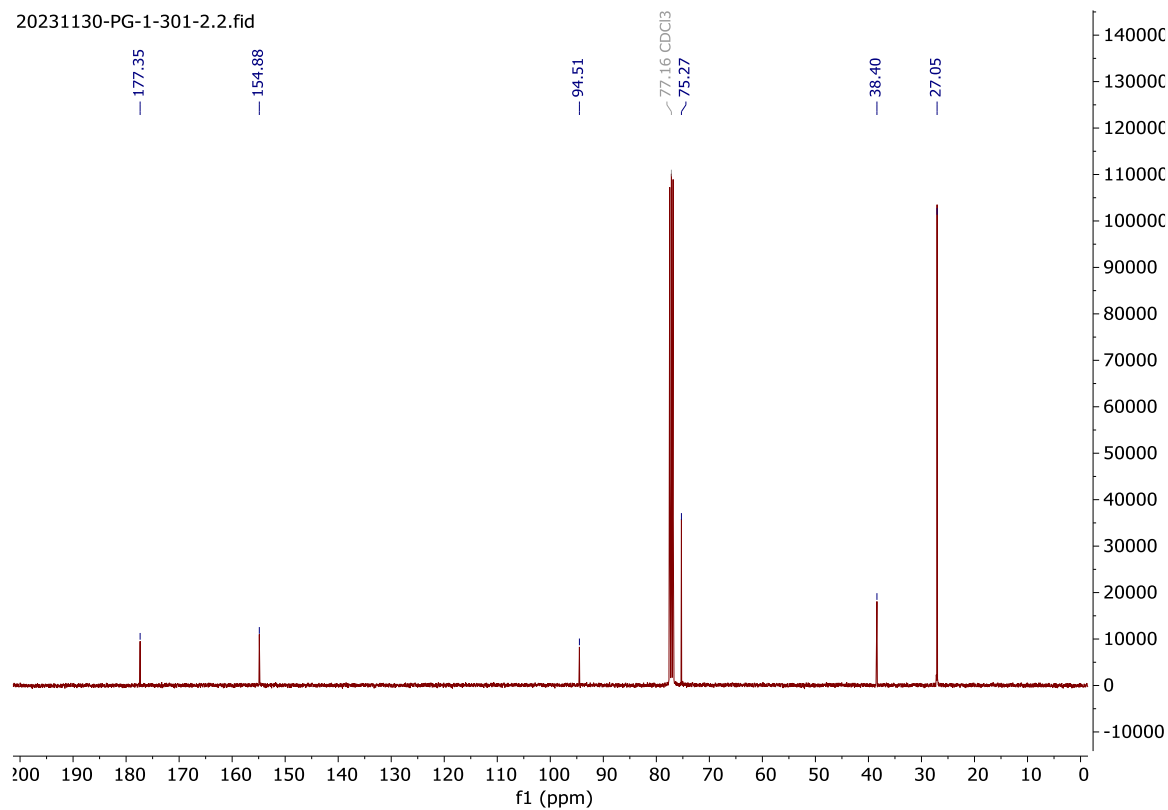

2,2,2-trichloroethyl (R)-(1,2,3,4-tetrahydronaphthalen-2-yl)carbamate ((R)-10):

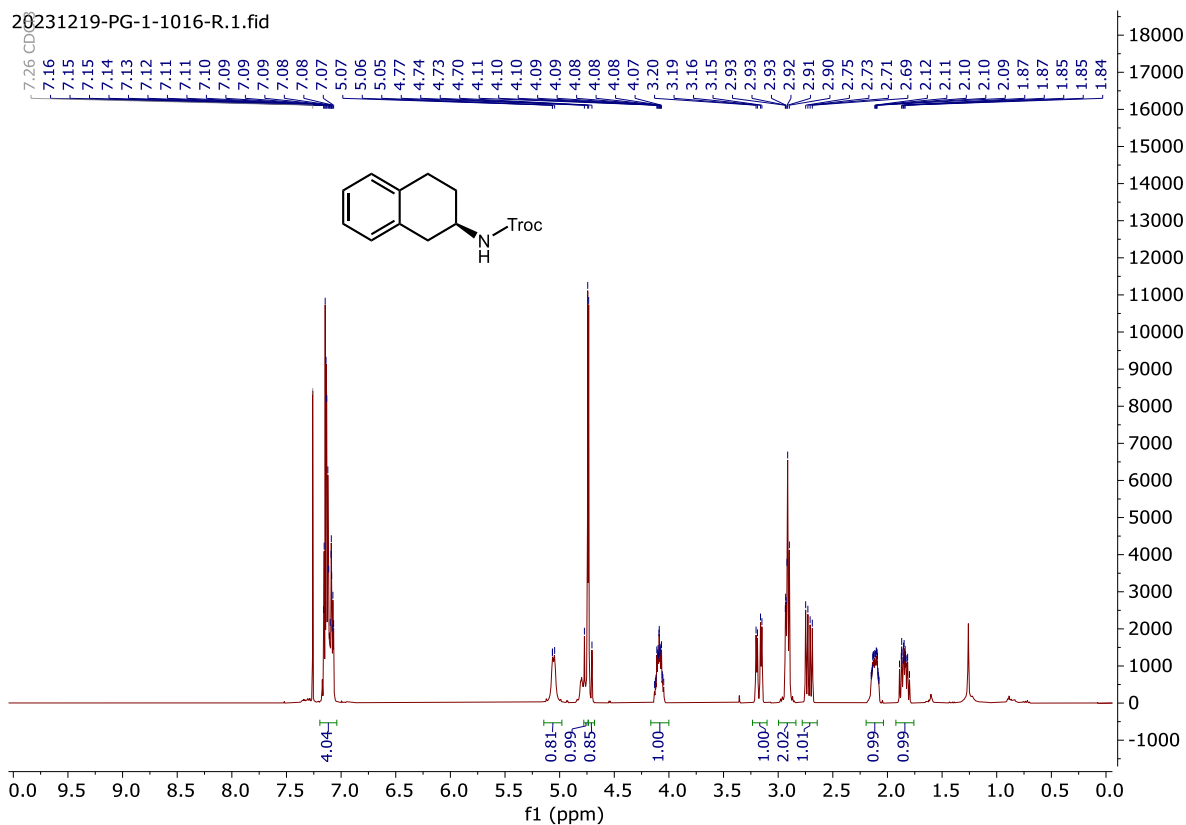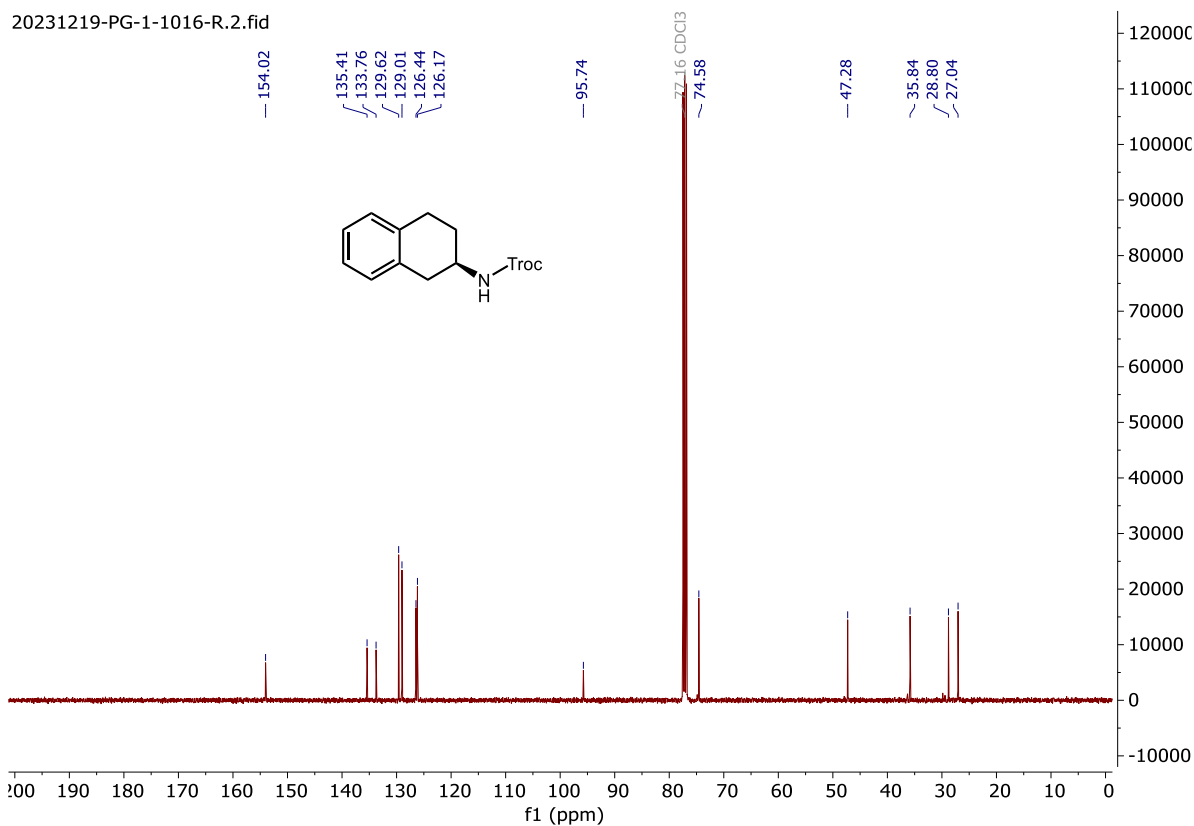

2,2,2-trichloroethyl (1,2,3,4-tetrahydronaphthalen-2-yl)carbamate ($\pm$ -10):

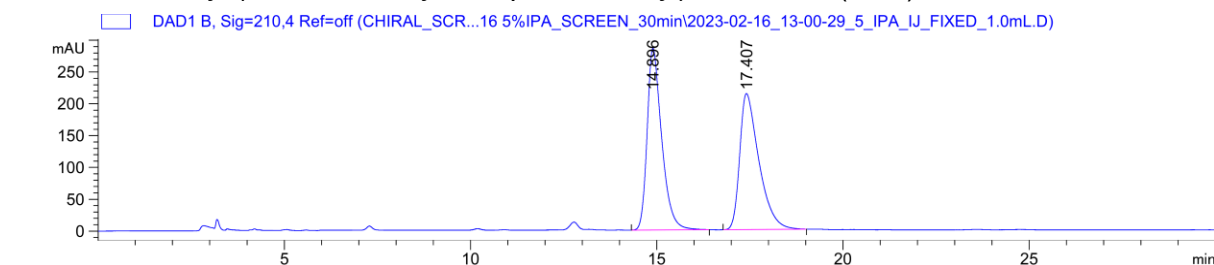

Signal 2: DAD1 B, Sig=210,4 Ref=off

Peak #	RetTime [min]	Type	Width [min]	Area [mAU*s]	Height [mAU]	Area %
1	14.896	BB	0.3731	7745.97803	285.46552	50.3063
2	17.407	BB	0.4476	7651.66211	214.02701	49.6937

Totals : 1.53976e4 499.49252

2,2,2-trichloroethyl (R)-(1,2,3,4-tetrahydronaphthalen-2-yl)carbamate (10):

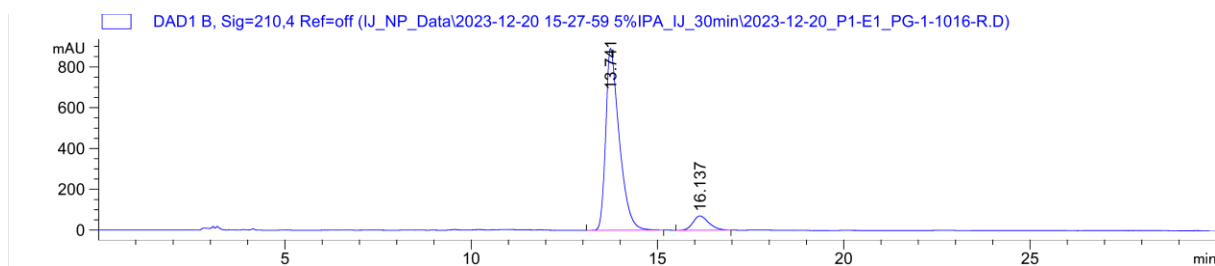

Signal 2: DAD1 B, Sig=210,4 Ref=off

Peak #	RetTime [min]	Type	Width [min]	Area [mAU*s]	Height [mAU]	Area %
1	13.741	VV R	0.3316	2.25000e4	891.12781	91.6616
2	16.137	VV R	0.3432	2046.81458	69.97881	8.3384

Totals : 2.45468e4 961.10662

2,2,2-trichloroethyl (R)-(7-methoxy-1,2,3,4-tetrahydronaphthalen-2-yl)carbamate (**19a**) and 2,2,2-trichloroethyl (R)-(6-methoxy-1,2,3,4-tetrahydronaphthalen-2-yl)carbamate (**19b**):

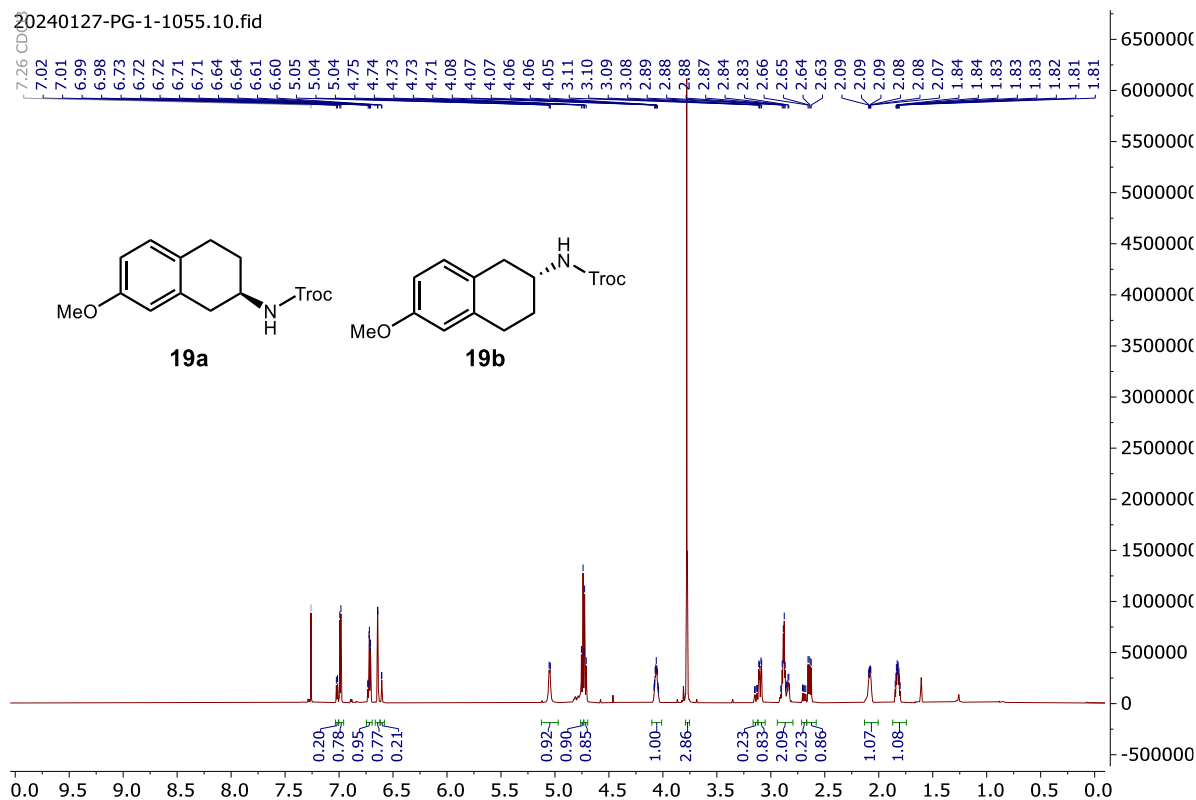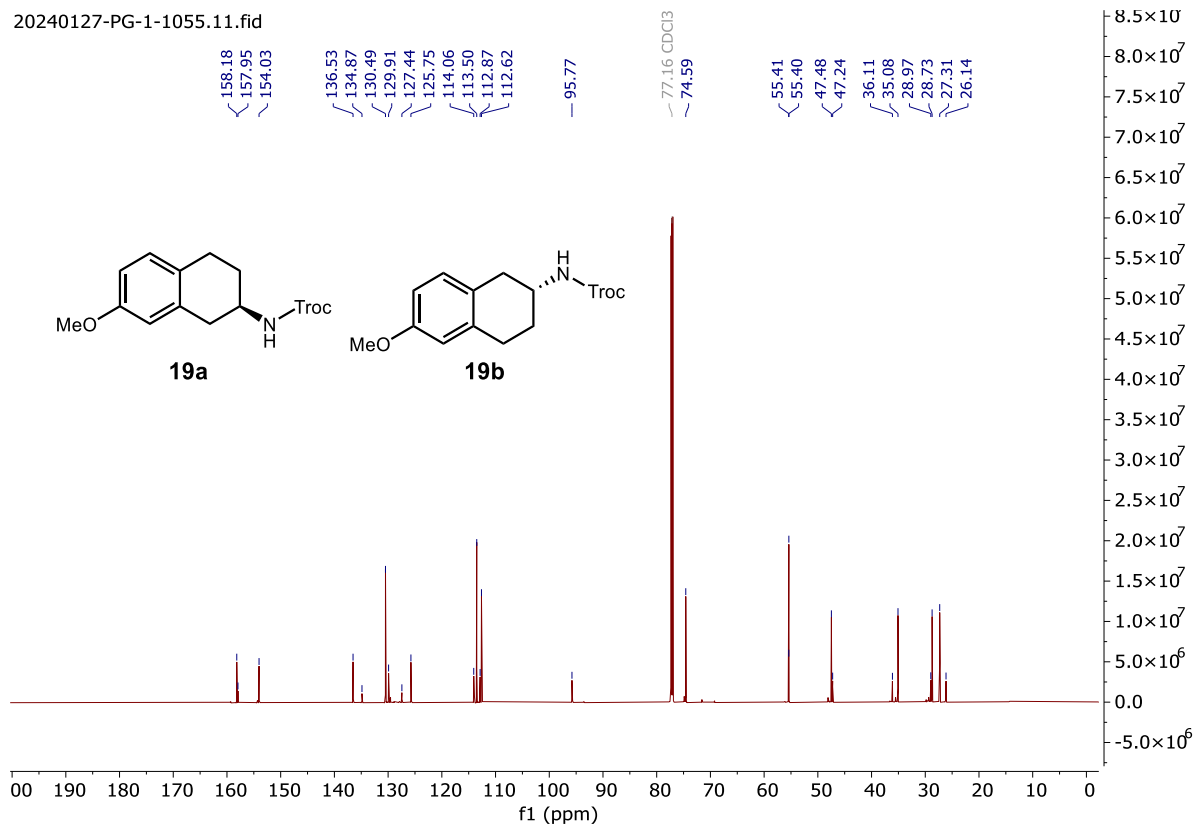

(19a) and (19b):

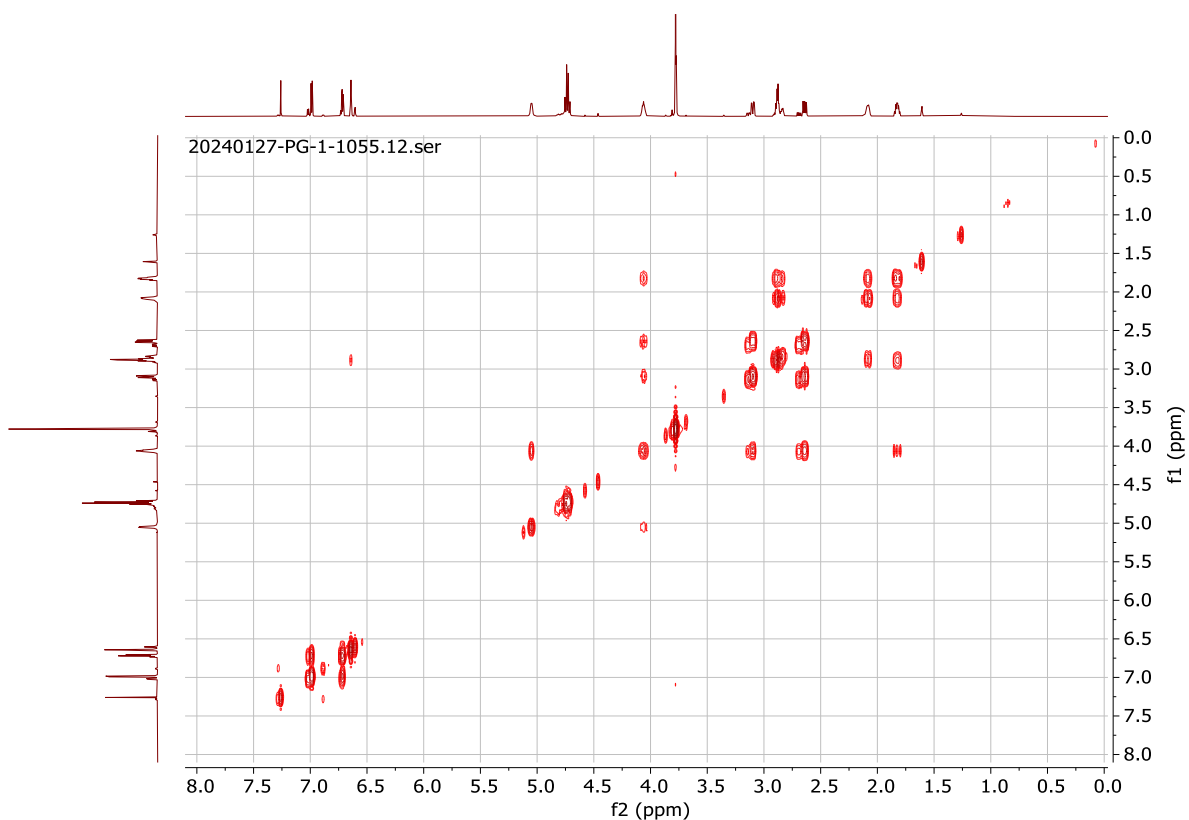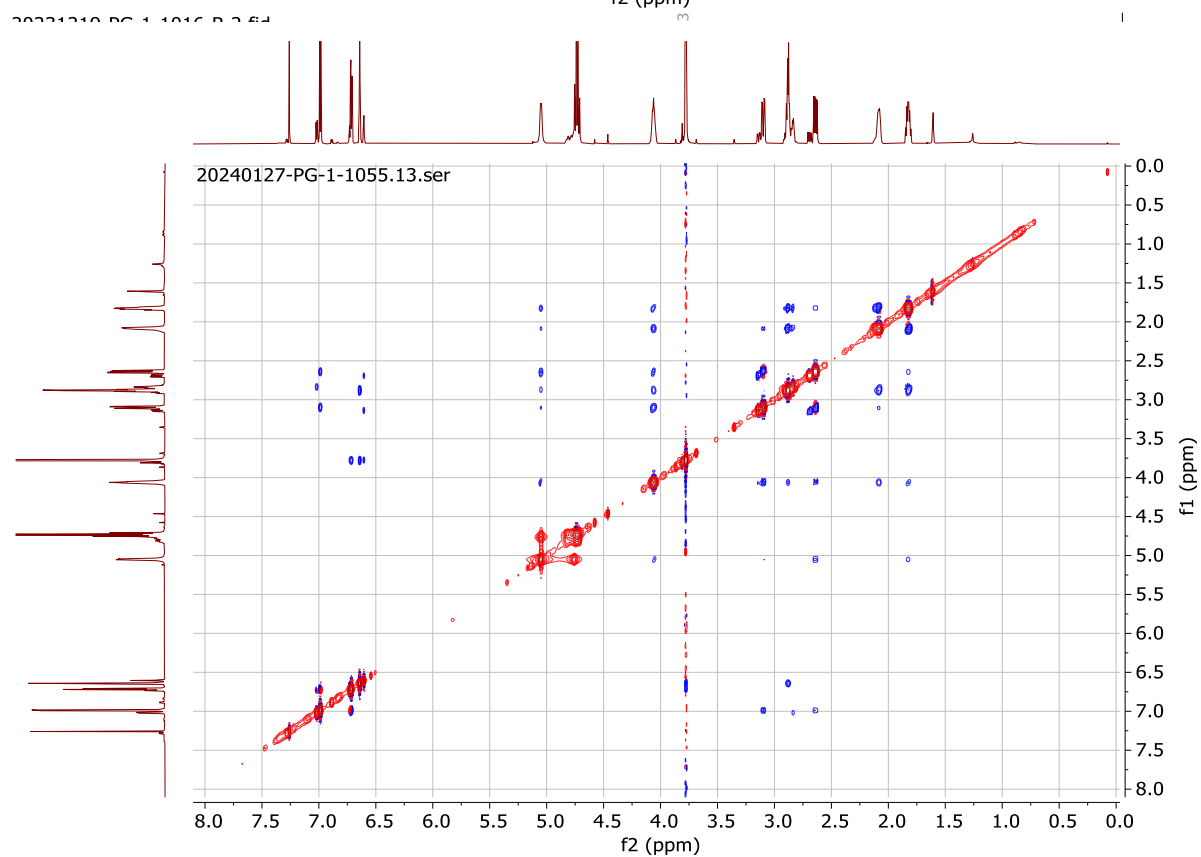

2,2,2-trichloroethyl (7-methoxy-1,2,3,4-tetrahydronaphthalen-2-yl)carbamate ($\pm$ -19a):

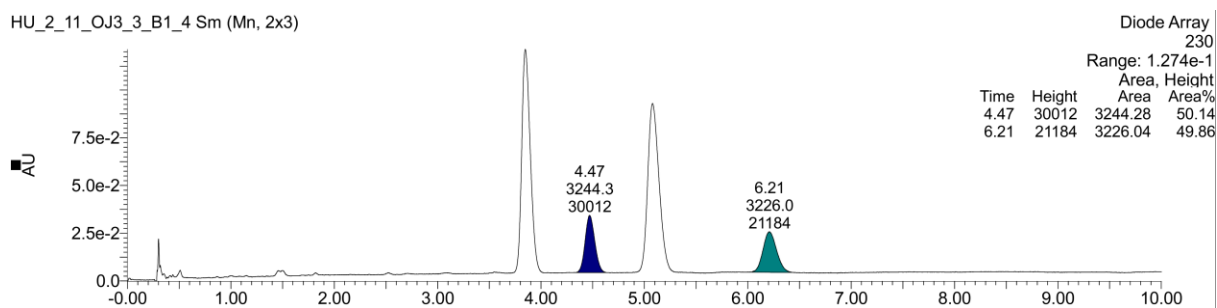

2,2,2-trichloroethyl (R)-(7-methoxy-1,2,3,4-tetrahydronaphthalen-2-yl)carbamate (19a):

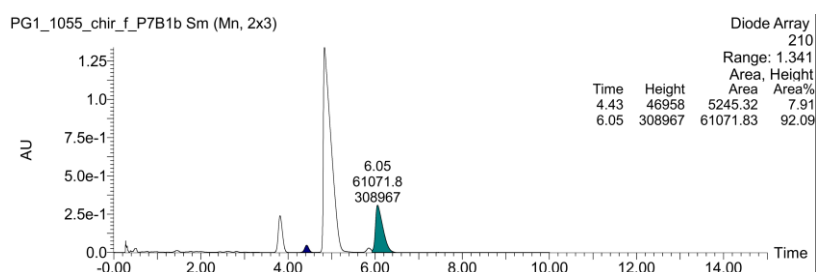

2,2,2-trichloroethyl (6-methoxy-1,2,3,4-tetrahydronaphthalen-2-yl)carbamate ($\pm$ -19b):

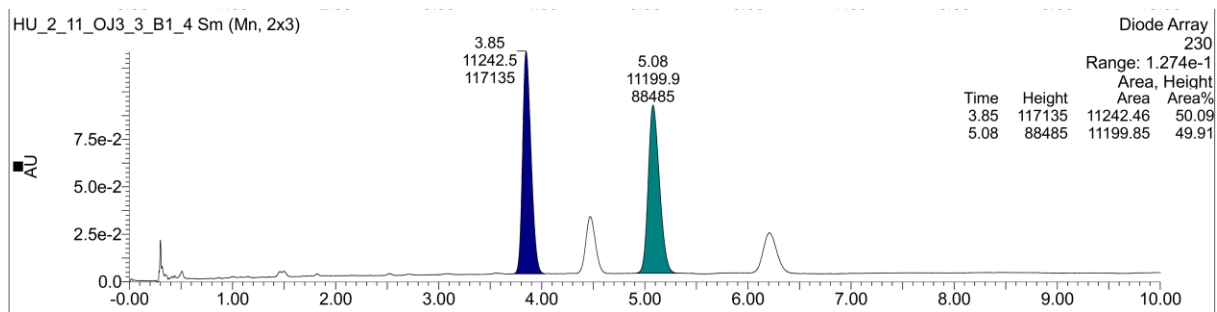

2,2,2-trichloroethyl (R)-(6-methoxy-1,2,3,4-tetrahydronaphthalen-2-yl)carbamate (19b):

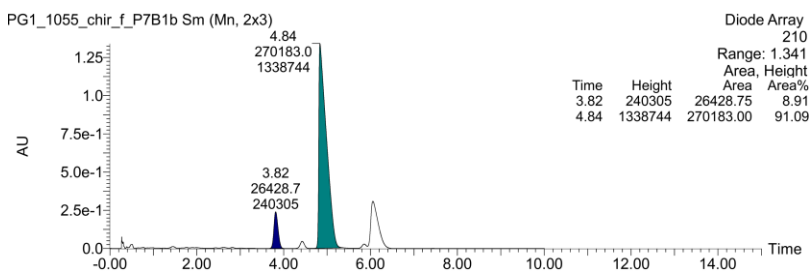

2,2,2-trichloroethyl (*R*)-(5-methoxy-1,2,3,4-tetrahydronaphthalen-2-yl)carbamate (**20'**) and 2,2,2-trichloroethyl (*R*)-(8-methoxy-1,2,3,4-tetrahydronaphthalen-2-yl)carbamate (**20**):

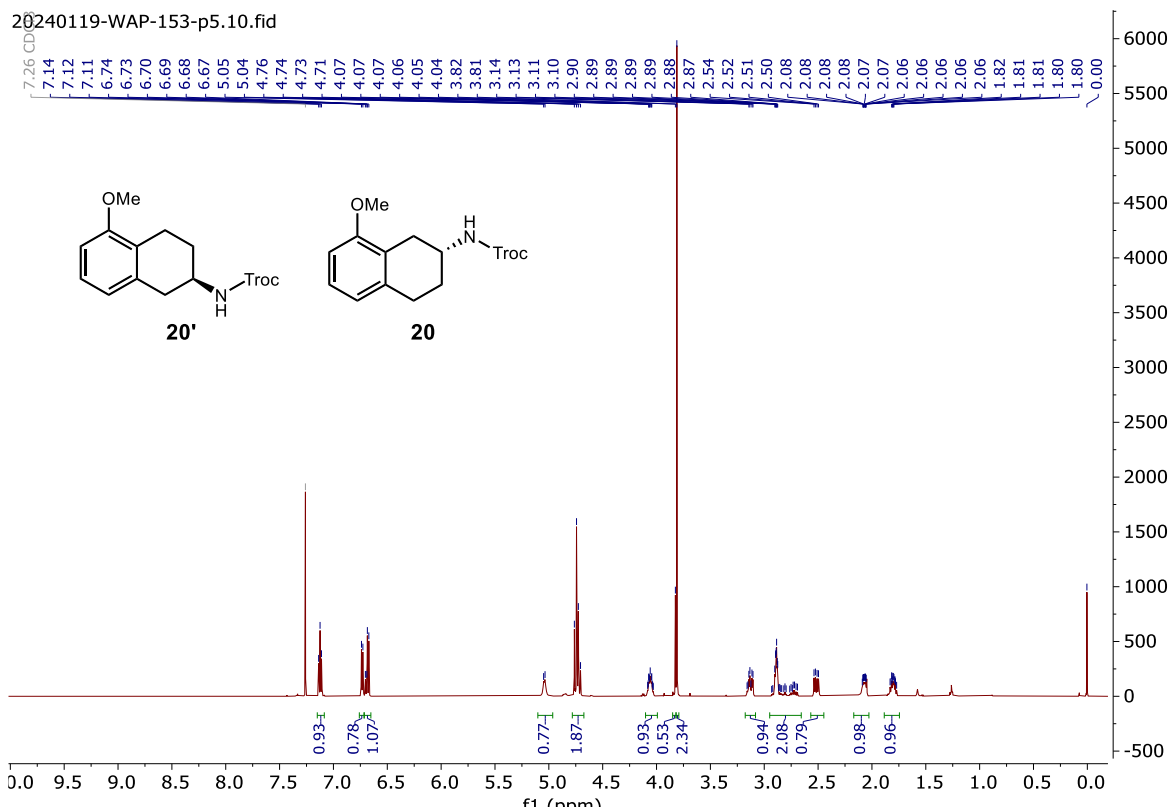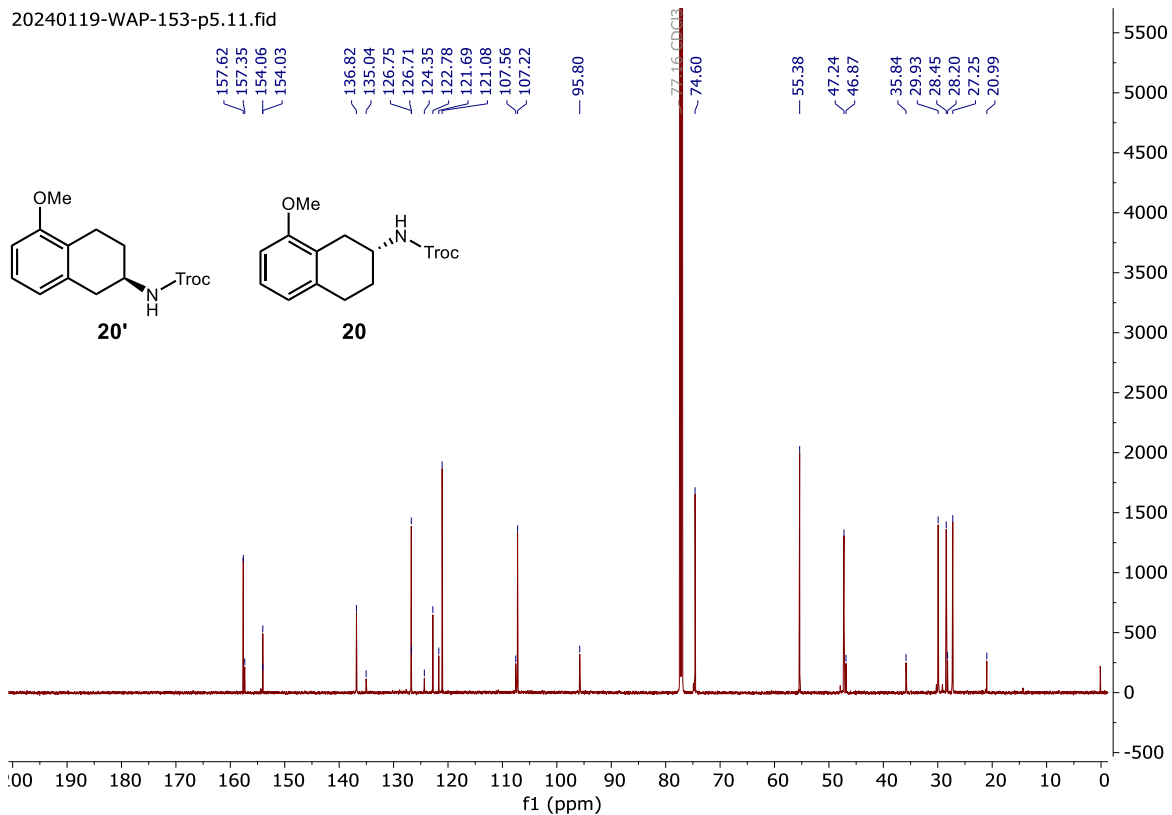

(20) and (20'):

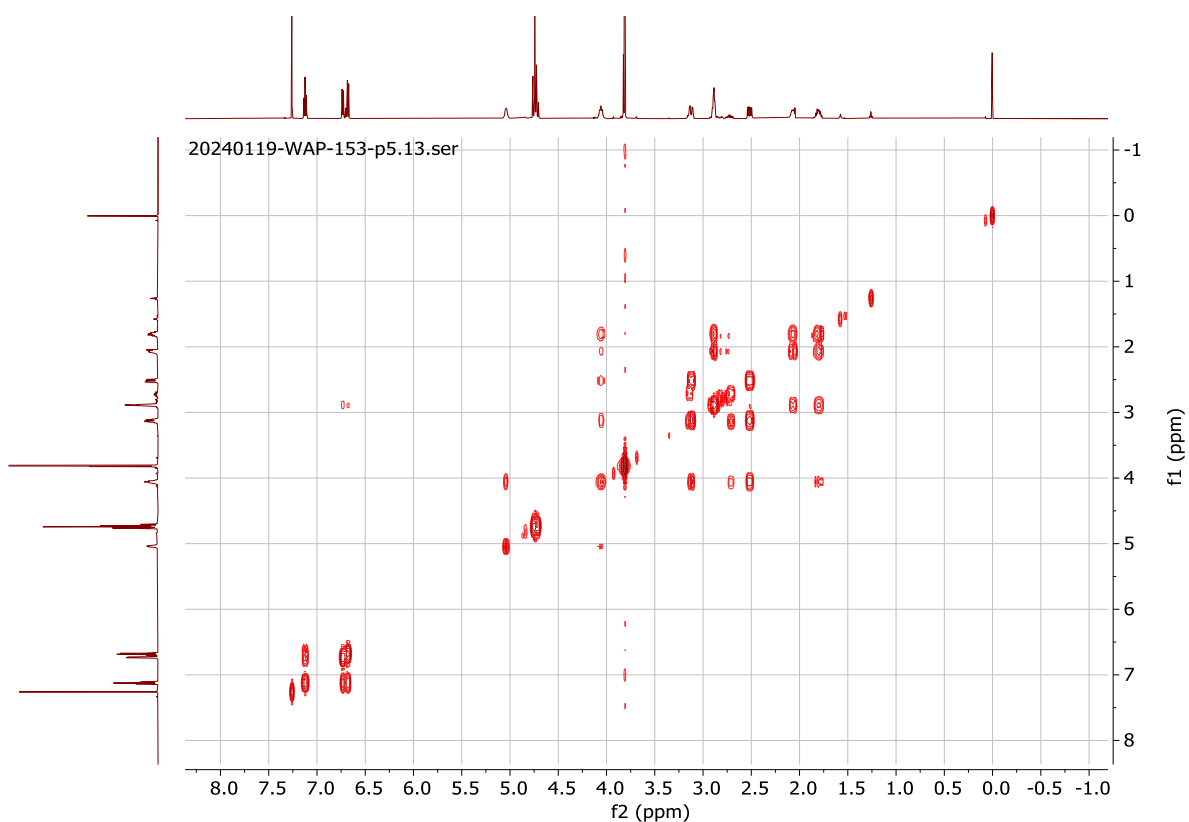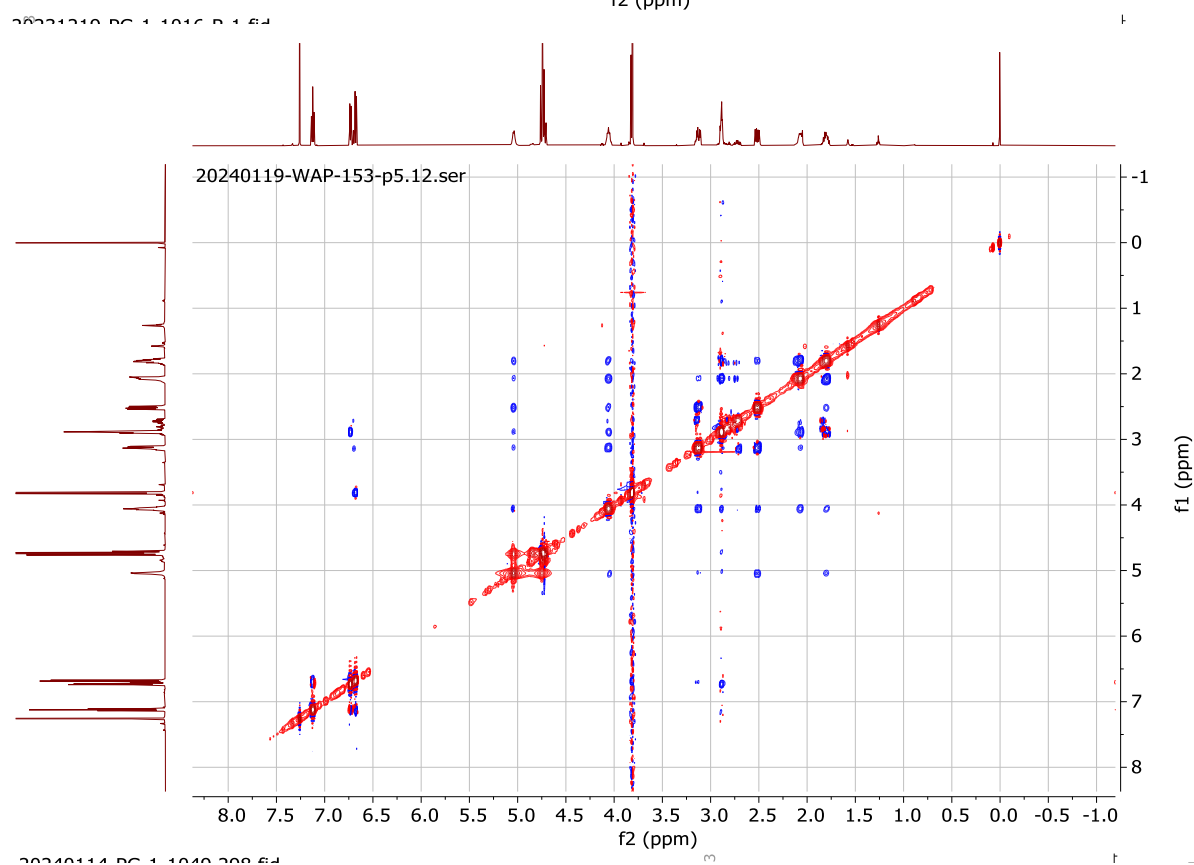

2,2,2-trichloroethyl (5-methoxy-1,2,3,4-tetrahydronaphthalen-2-yl)carbamate (20'):

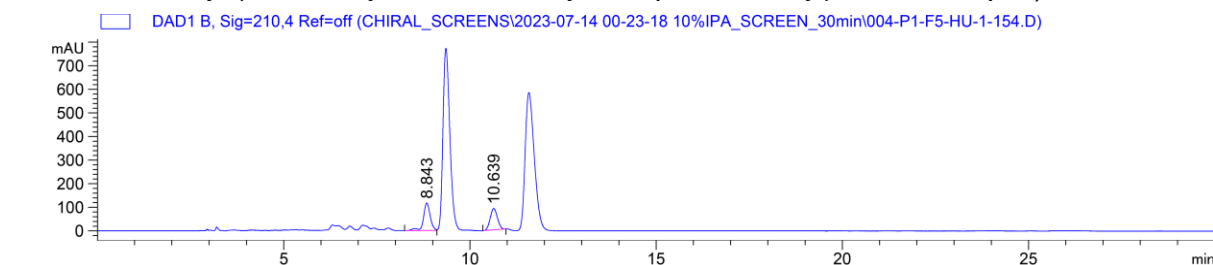

Signal 2: DAD1 B, Sig=210,4 Ref=off

Peak #	RetTime [min]	Type	Width [min]	Area [mAU*s]	Height [mAU]	Area %
1	8.843	VV R	0.1734	1462.58716	116.43881	54.5366
2	10.639	BV R	0.1864	1219.25684	90.63749	45.4634

Totals : 2681.84399 207.07630

2,2,2-trichloroethyl (R)-(5-methoxy-1,2,3,4-tetrahydronaphthalen-2-yl)carbamate (20'):

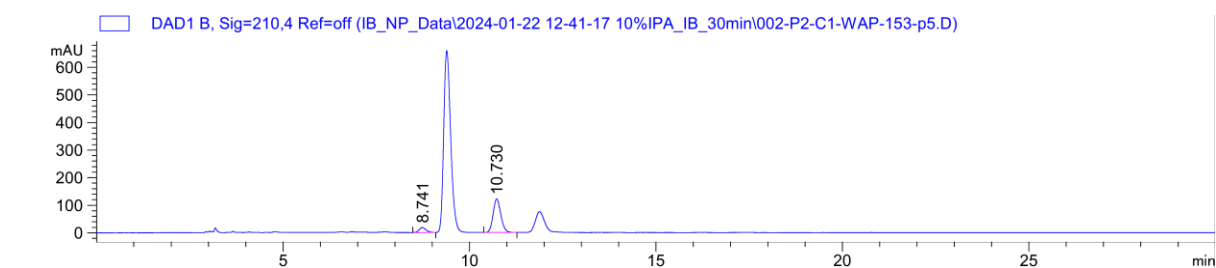

Signal 2: DAD1 B, Sig=210,4 Ref=off

Peak #	RetTime [min]	Type	Width [min]	Area [mAU*s]	Height [mAU]	Area %
1	8.741	BV R	0.1511	230.56030	18.10416	11.6884
2	10.730	VV R	0.1908	1741.99695	121.47370	88.3116

Totals : 1972.55725 139.57786

2,2,2-trichloroethyl (8-methoxy-1,2,3,4-tetrahydronaphthalen-2-yl)carbamate ($\pm$ -20):

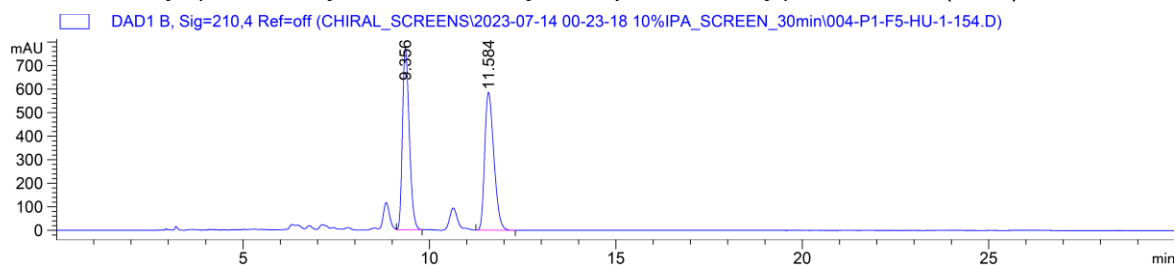

Signal 2: DAD1 B, Sig=210,4 Ref=off

Peak #	RetTime [min]	Type	Width [min]	Area [mAU*s]	Height [mAU]	Area %
1	9.356	VB	0.1939	9832.21582	770.42181	49.5640
2	11.584	BV R	0.2432	1.00052e4	586.10193	50.4360

Totals : 1.98374e4 1356.52374

2,2,2-trichloroethyl (R)-(8-methoxy-1,2,3,4-tetrahydronaphthalen-2-yl)carbamate (20):

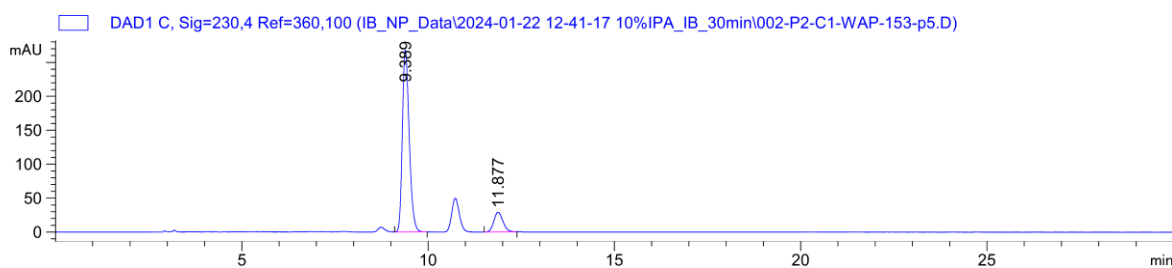

Signal 2: DAD1 B, Sig=210,4 Ref=off

Peak #	RetTime [min]	Type	Width [min]	Area [mAU*s]	Height [mAU]	Area %
1	9.389	VV R	0.1981	8540.25586	659.27283	86.7633
2	11.875	BV R	0.2057	1302.91284	75.37483	13.2367

Totals : 9843.16870 734.64766

2,2,2-trichloroethyl (R)-(7-(allyloxy)-1,2,3,4-tetrahydronaphthalen-2-yl)carbamate (**21'**) and 2,2,2-trichloroethyl (R)-(6-(allyloxy)-1,2,3,4-tetrahydronaphthalen-2-yl)carbamate (**21**):

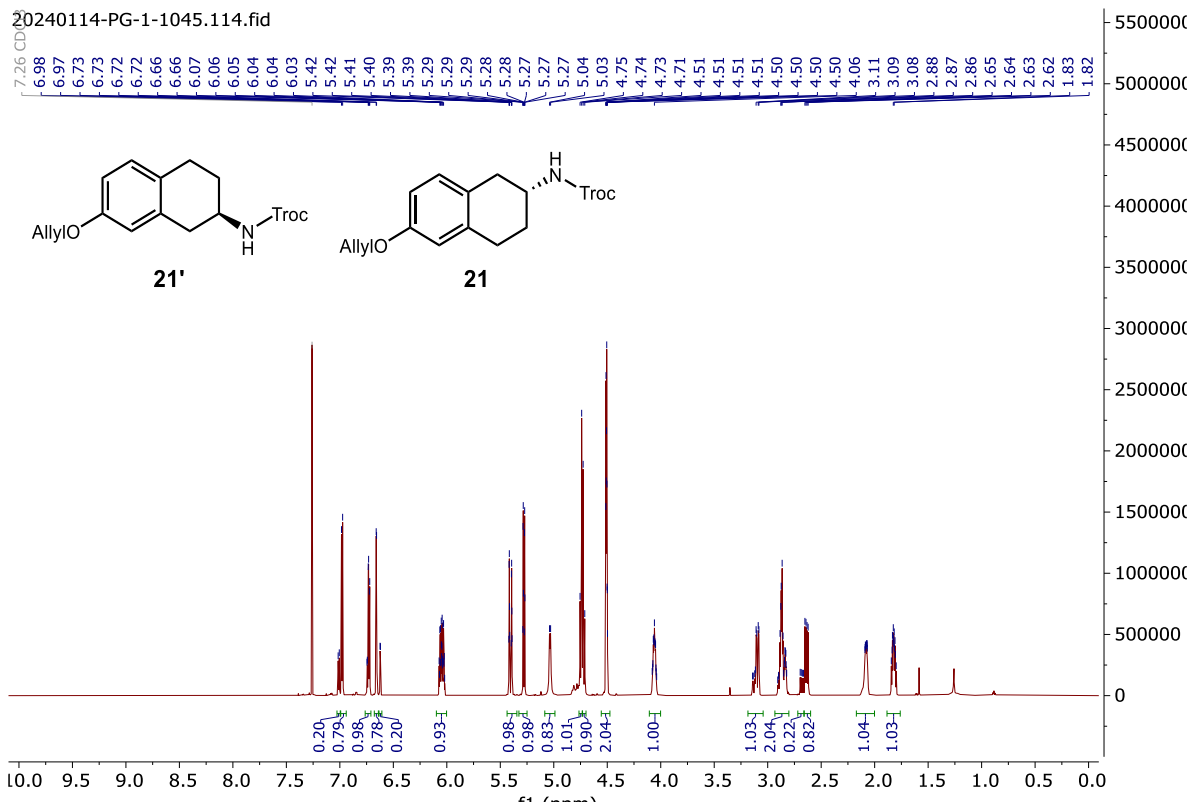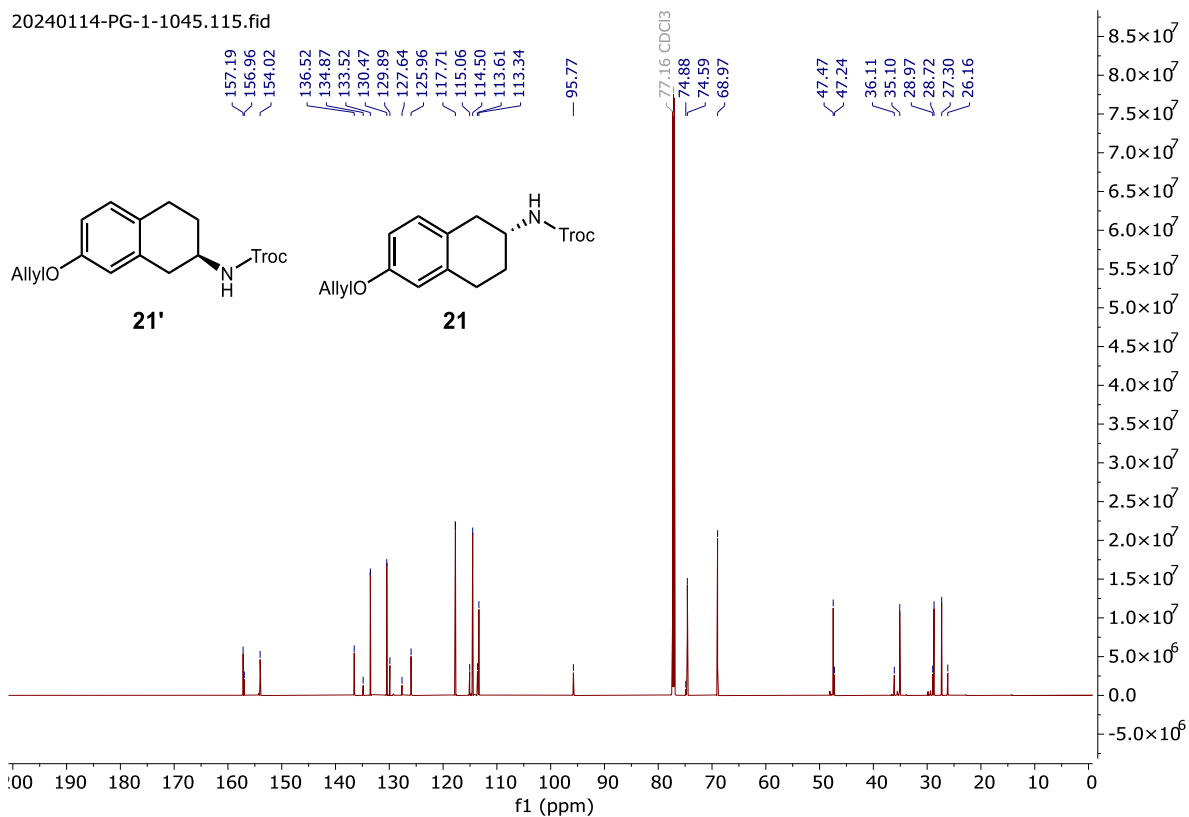

(21) and (21'):

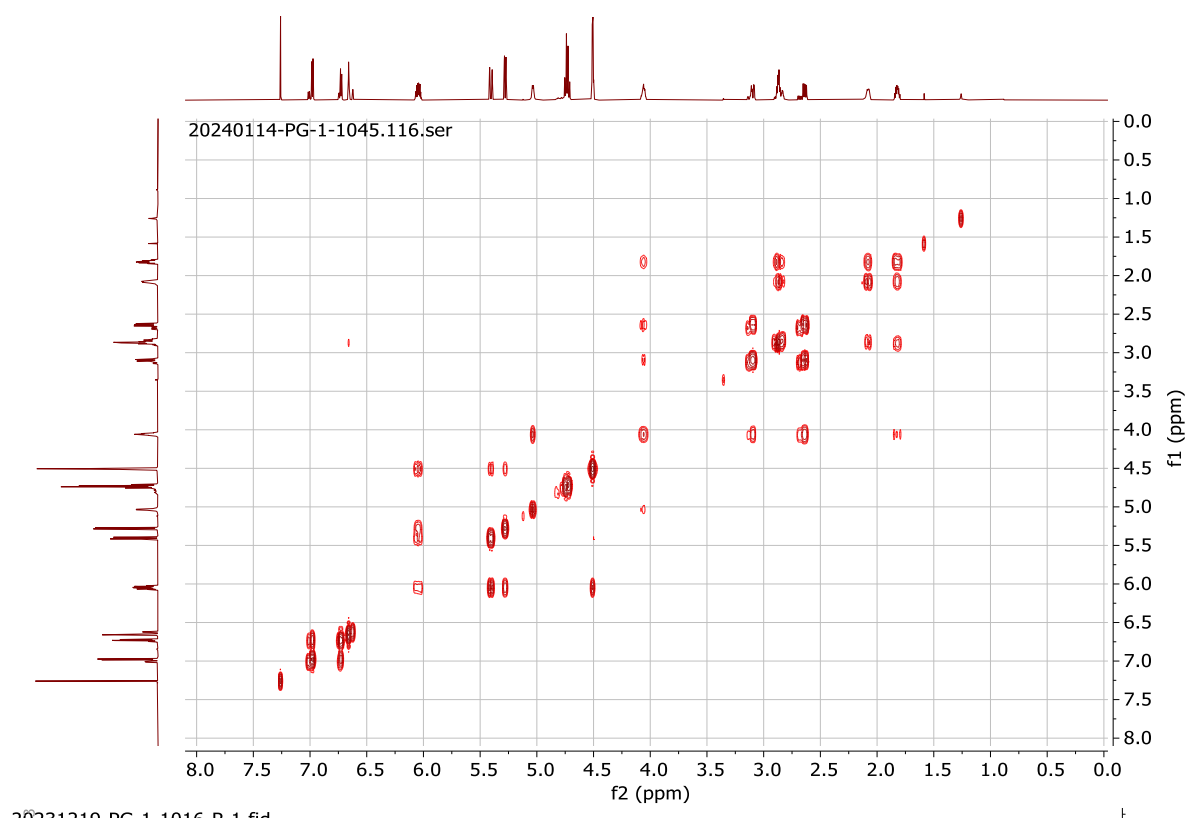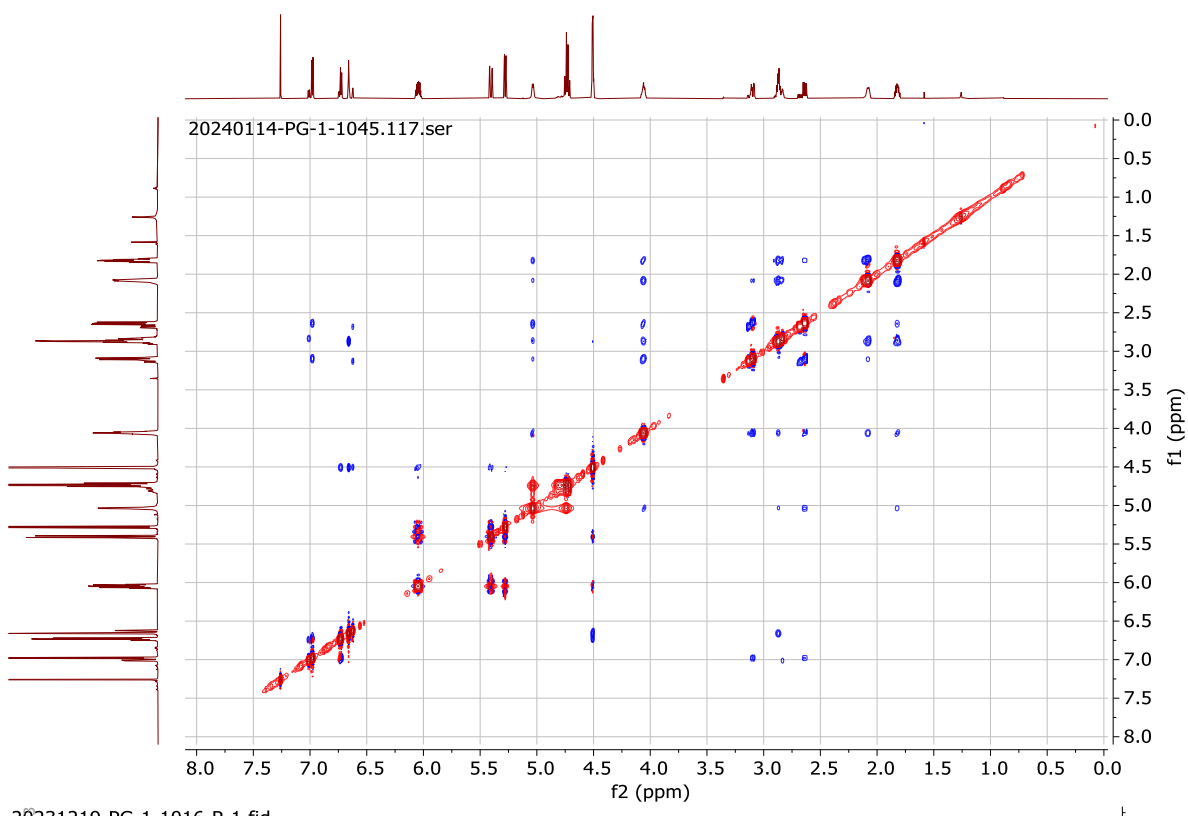

2,2,2-trichloroethyl (7-(allyloxy)-1,2,3,4-tetrahydronaphthalen-2-yl)carbamate ($\pm$ -21')

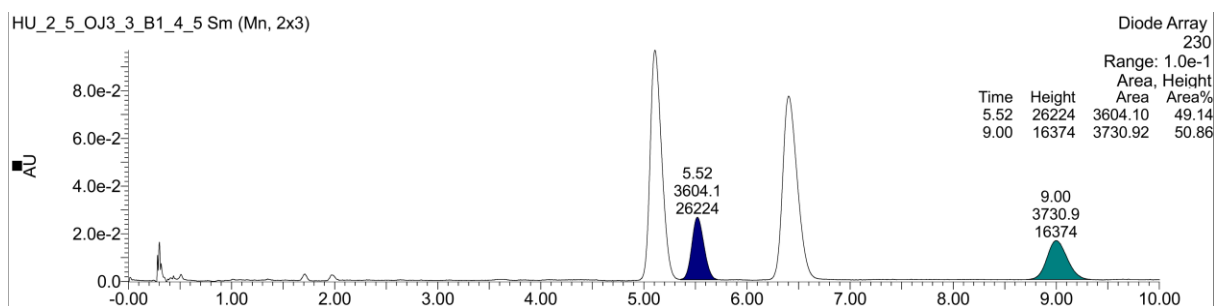

2,2,2-trichloroethyl (R)-(7-(allyloxy)-1,2,3,4-tetrahydronaphthalen-2-yl)carbamate (21')

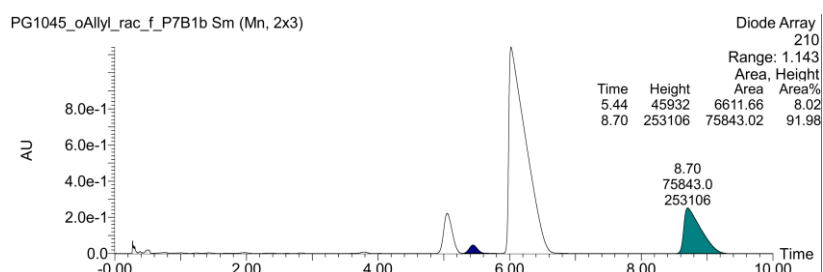

2,2,2-trichloroethyl (6-(allyloxy)-1,2,3,4-tetrahydronaphthalen-2-yl)carbamate ($\pm$ -21)

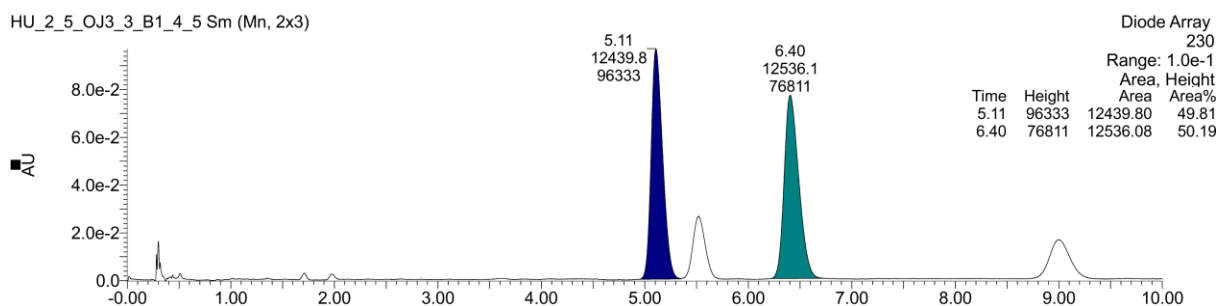

2,2,2-trichloroethyl (R)-(6-(allyloxy)-1,2,3,4-tetrahydronaphthalen-2-yl)carbamate (21)

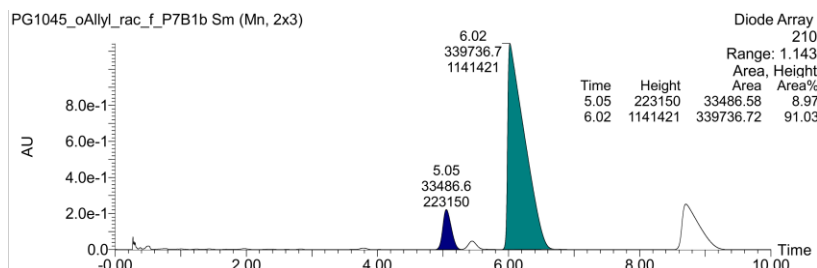

2,2,2-trichloroethyl (*R*)-(5-methyl-1,2,3,4-tetrahydronaphthalen-2-yl)carbamate (**22**) and 2,2,2-trichloroethyl (*R*)-(8-methyl-1,2,3,4-tetrahydronaphthalen-2-yl)carbamate (**22'**):

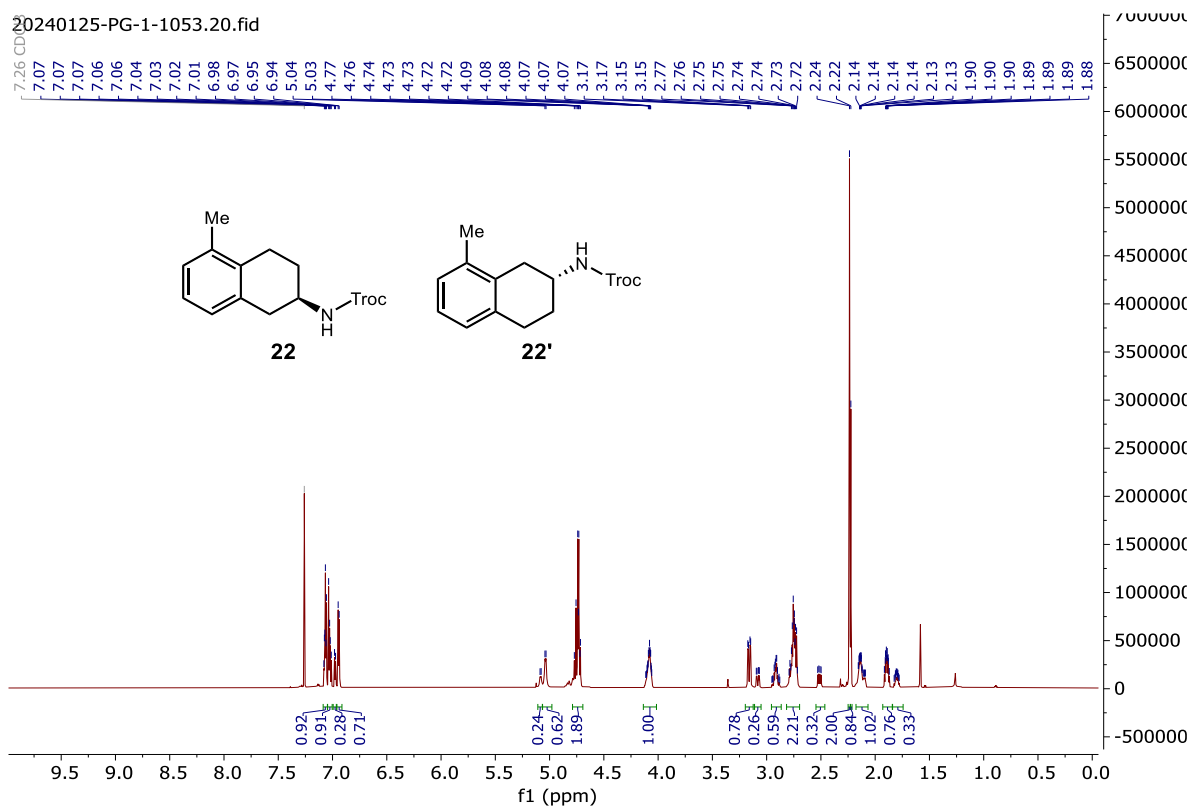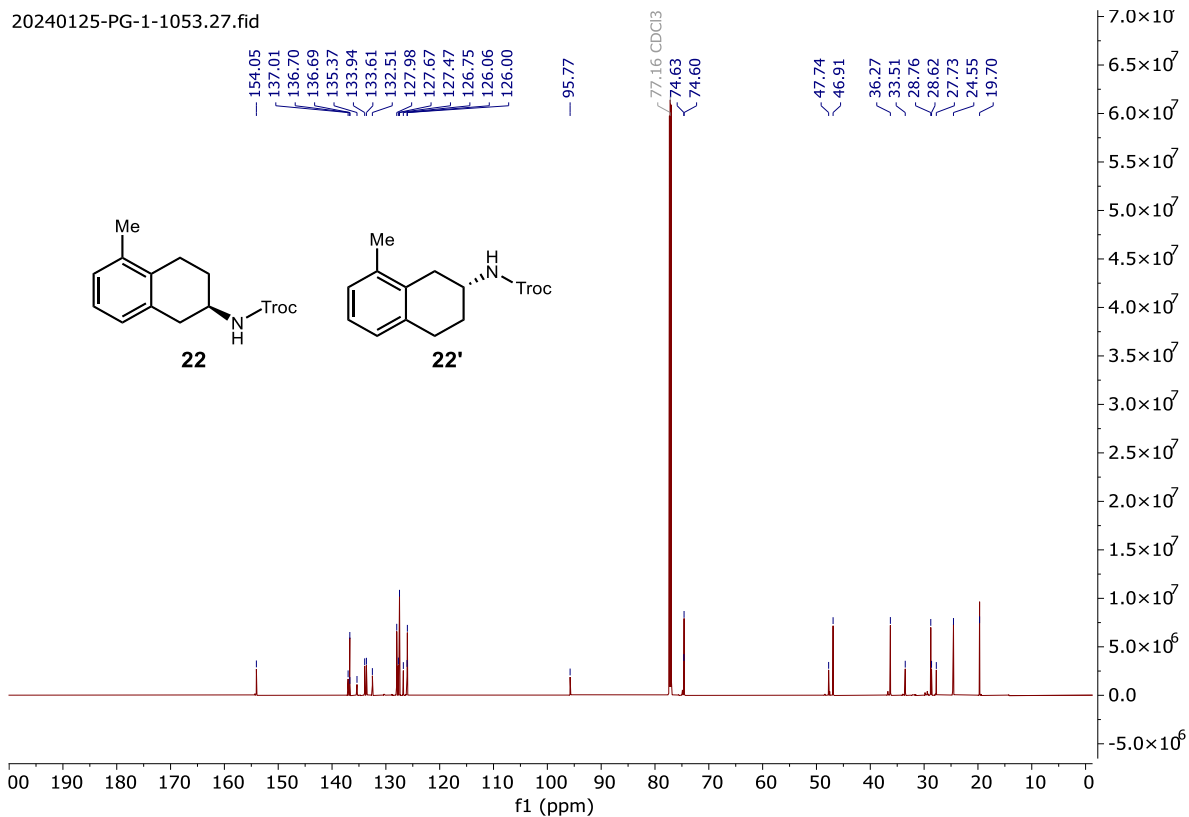

(22) and (22'):

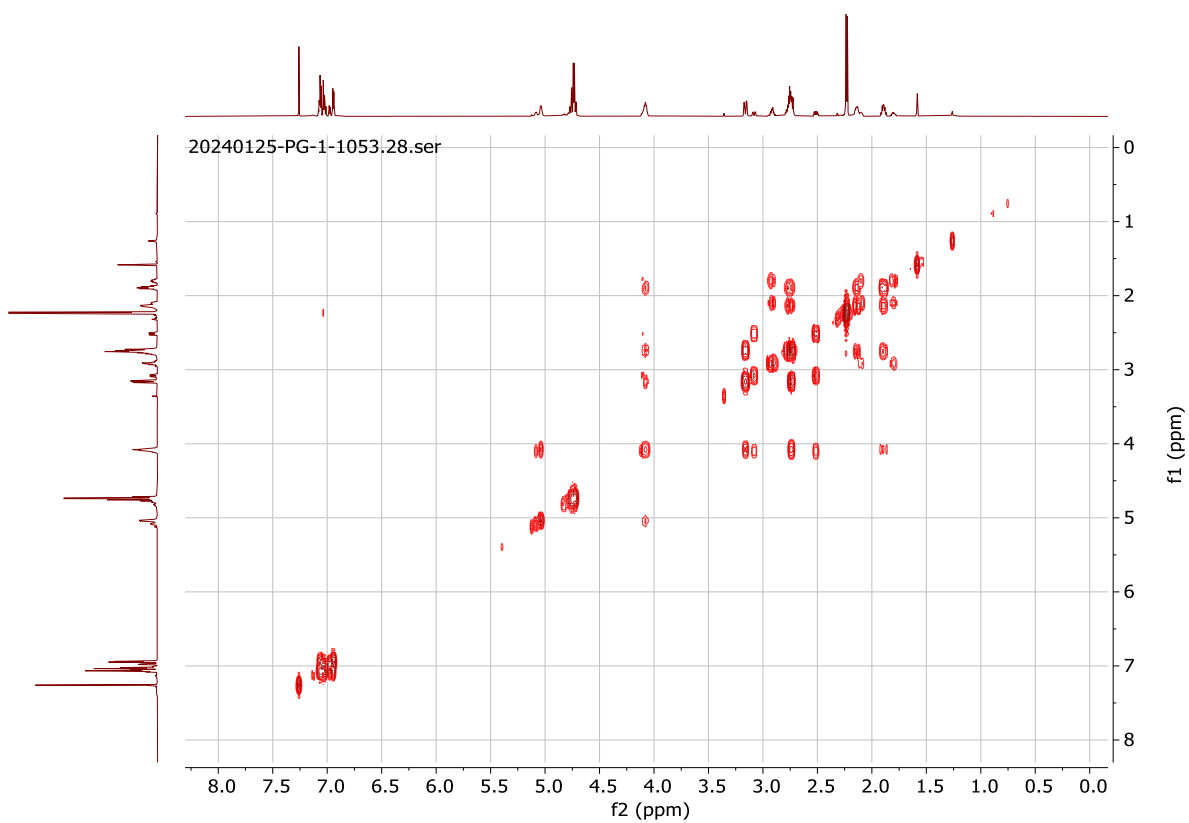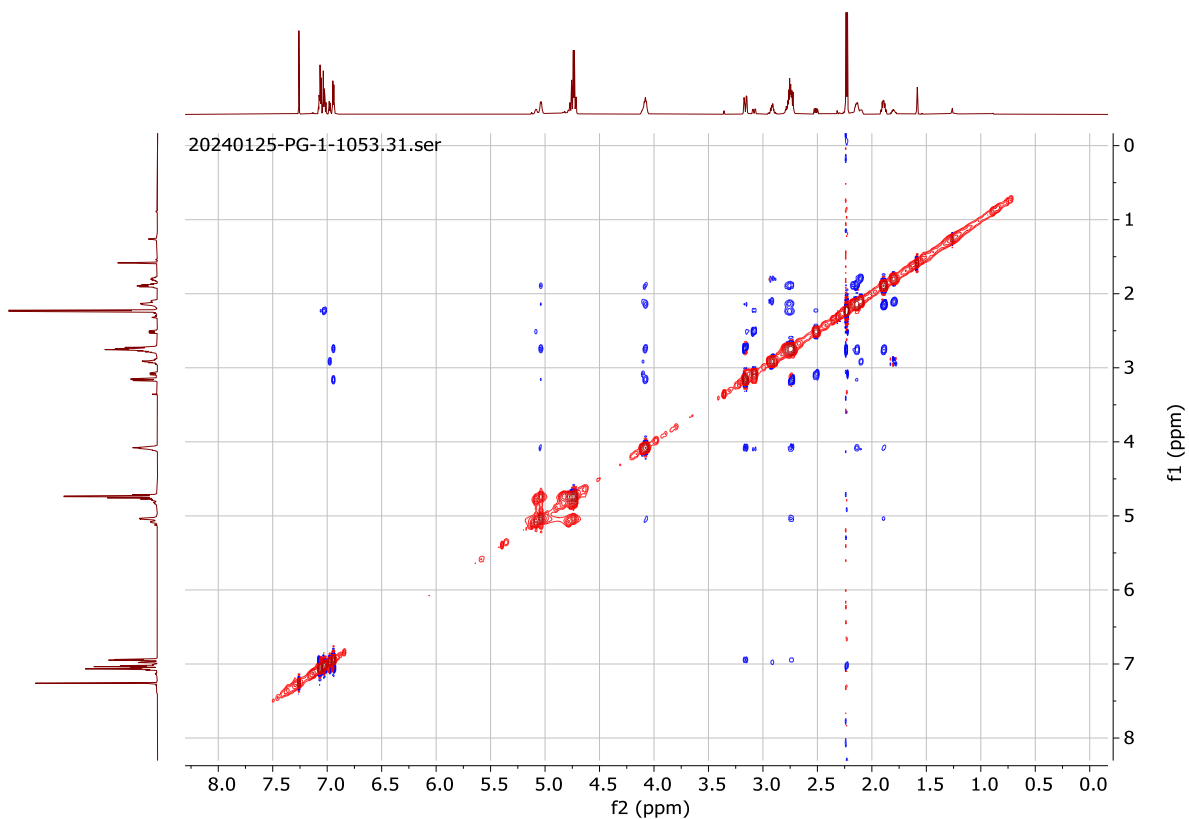

2,2,2-trichloroethyl (5-methyl-1,2,3,4-tetrahydronaphthalen-2-yl)carbamate ($\pm$ -22):

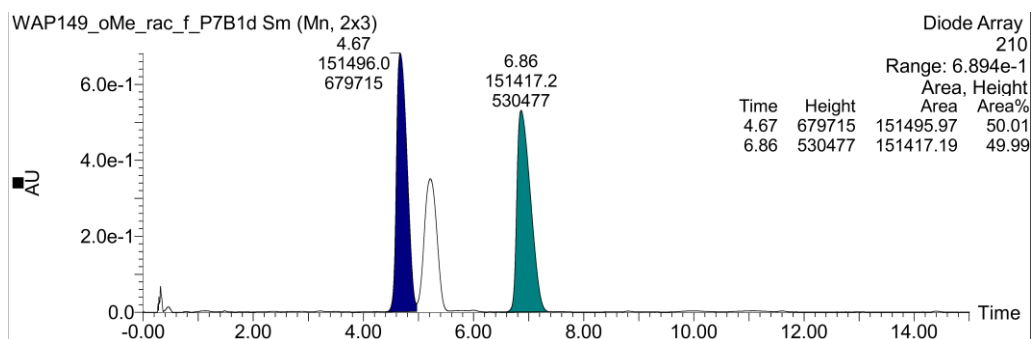

2,2,2-trichloroethyl (R)-(5-methyl-1,2,3,4-tetrahydronaphthalen-2-yl)carbamate (22):

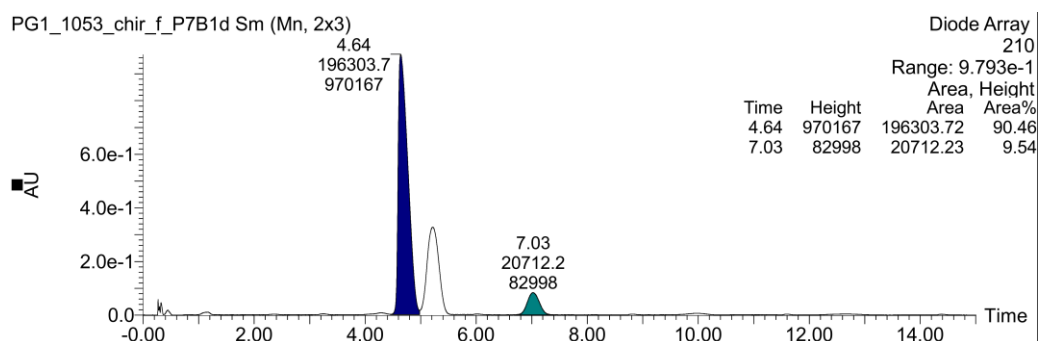

2,2,2-trichloroethyl (R)-(7-methyl-1,2,3,4-tetrahydronaphthalen-2-yl)carbamate (**23**) and 2,2,2-trichloroethyl (R)-(6-methyl-1,2,3,4-tetrahydronaphthalen-2-yl)carbamate (**23'**):

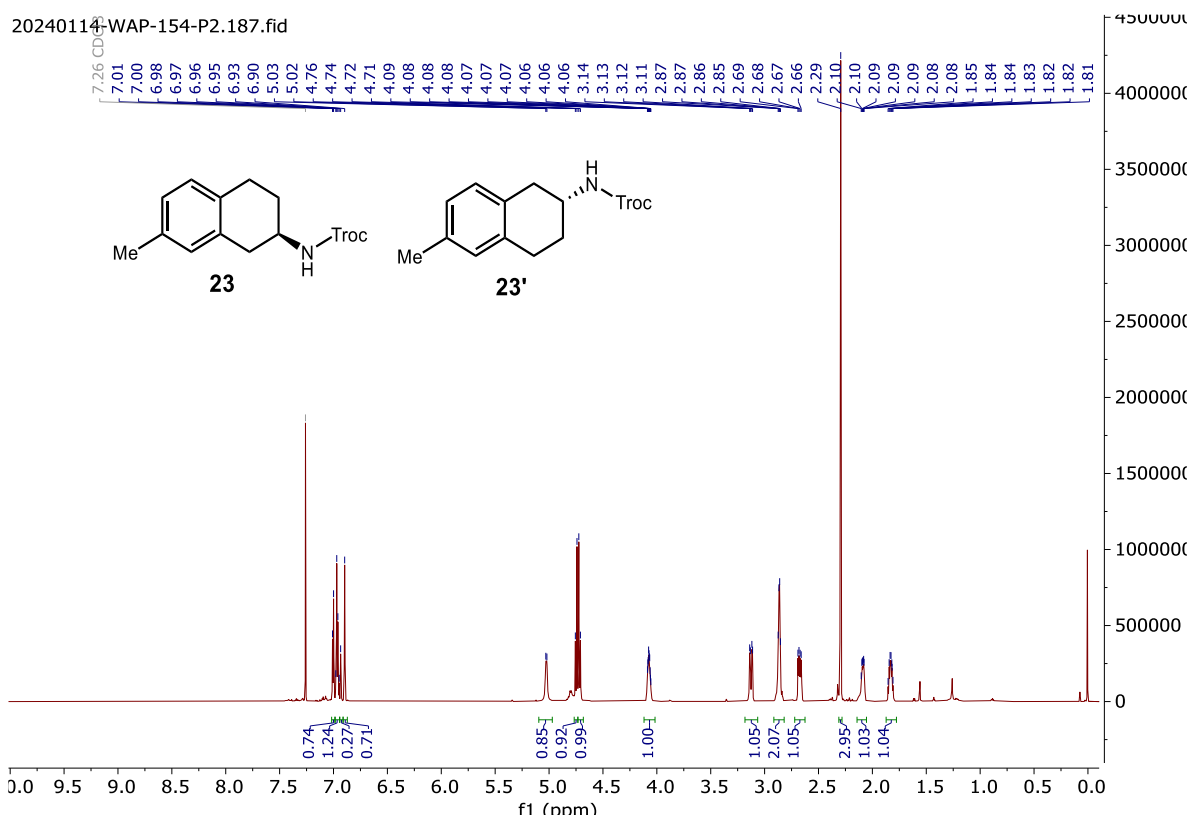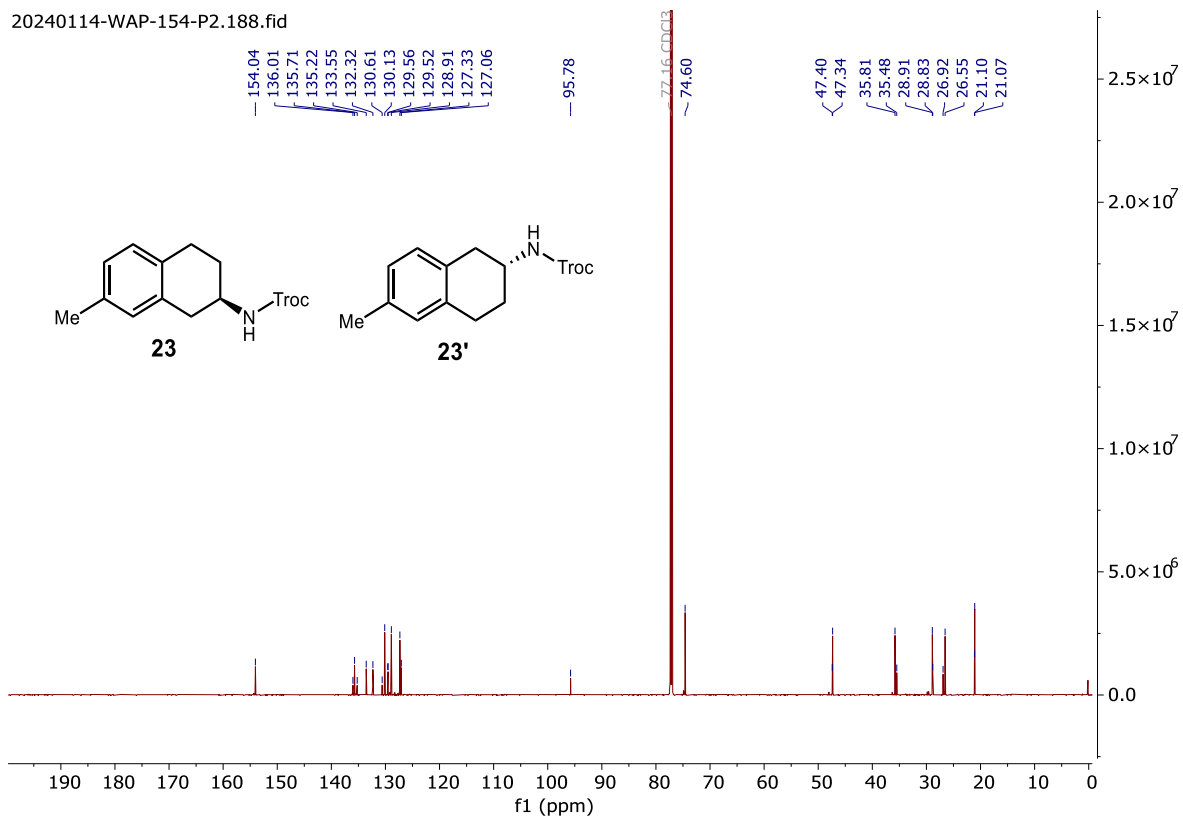

(23) and (23'):

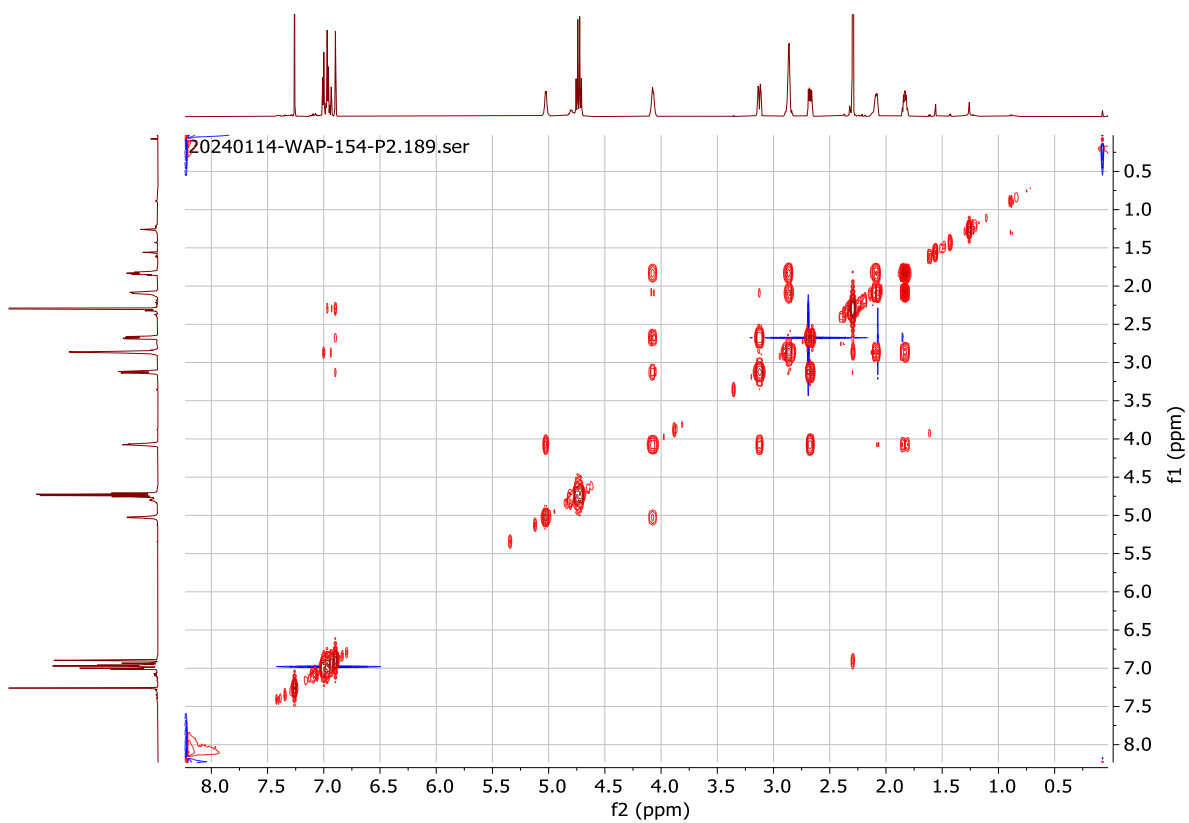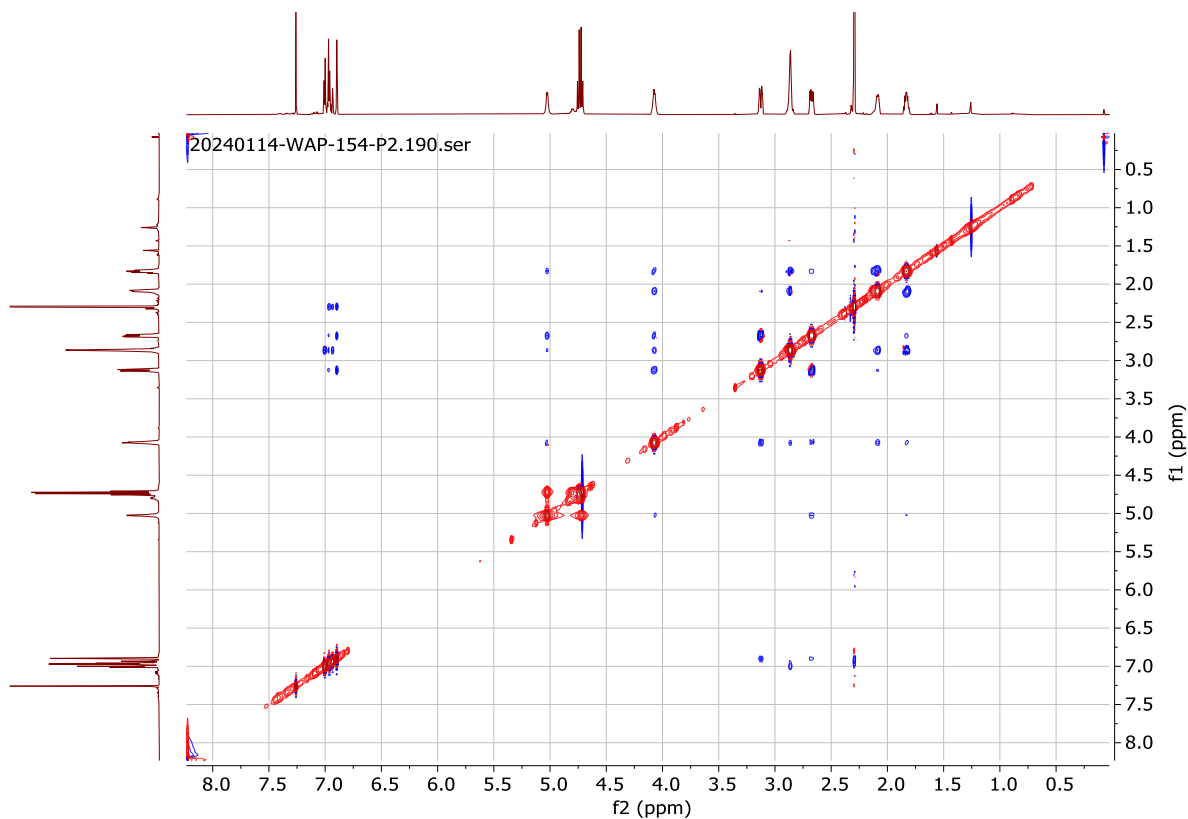

2,2,2-trichloroethyl (7-methyl-1,2,3,4-tetrahydronaphthalen-2-yl)carbamate ($\pm$ -23):

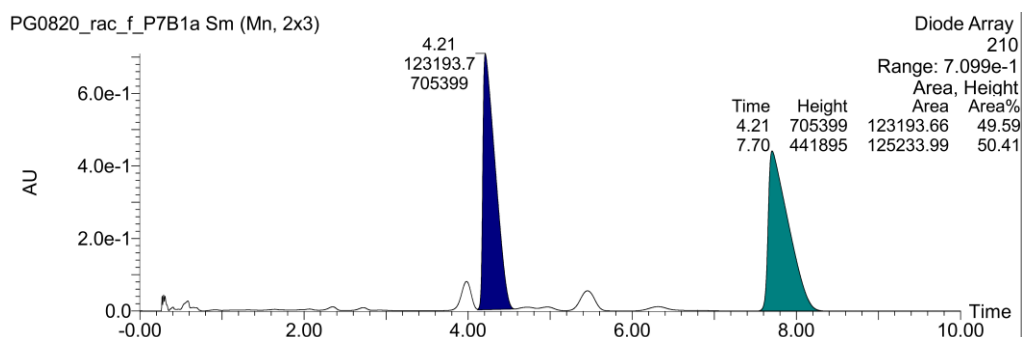

2,2,2-trichloroethyl (R)-(7-methyl-1,2,3,4-tetrahydronaphthalen-2-yl)carbamate (23):

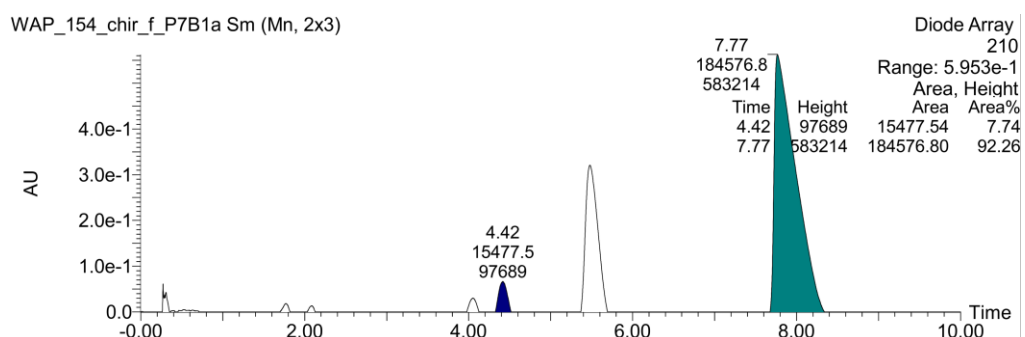

2,2,2-trichloroethyl (6-methyl-1,2,3,4-tetrahydronaphthalen-2-yl)carbamate ($\pm$ -23'):

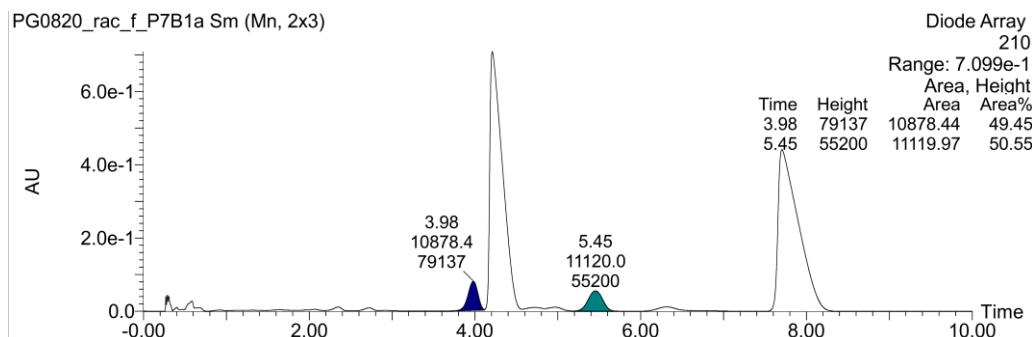

2,2,2-trichloroethyl (R)-(6-methyl-1,2,3,4-tetrahydronaphthalen-2-yl)carbamate (23'):

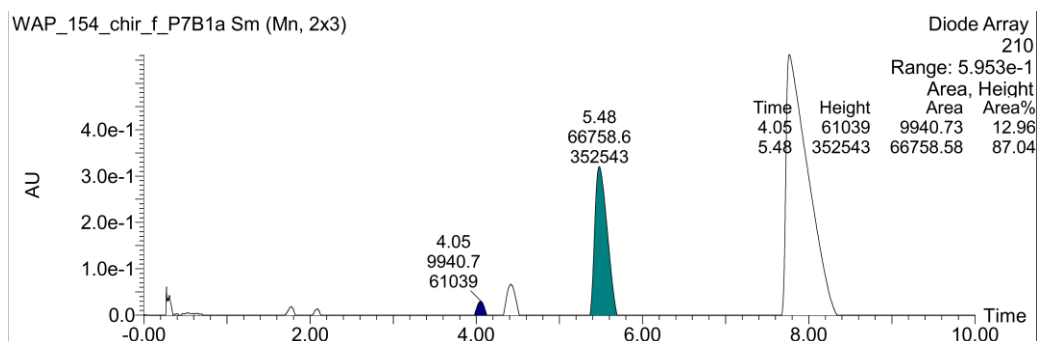

2,2,2-trichloroethyl (R)-(7-phenyl-1,2,3,4-tetrahydronaphthalen-2-yl)carbamate (24**) and 2,2,2-trichloroethyl (R)-(6-phenyl-1,2,3,4-tetrahydronaphthalen-2-yl)carbamate (**24'**):**

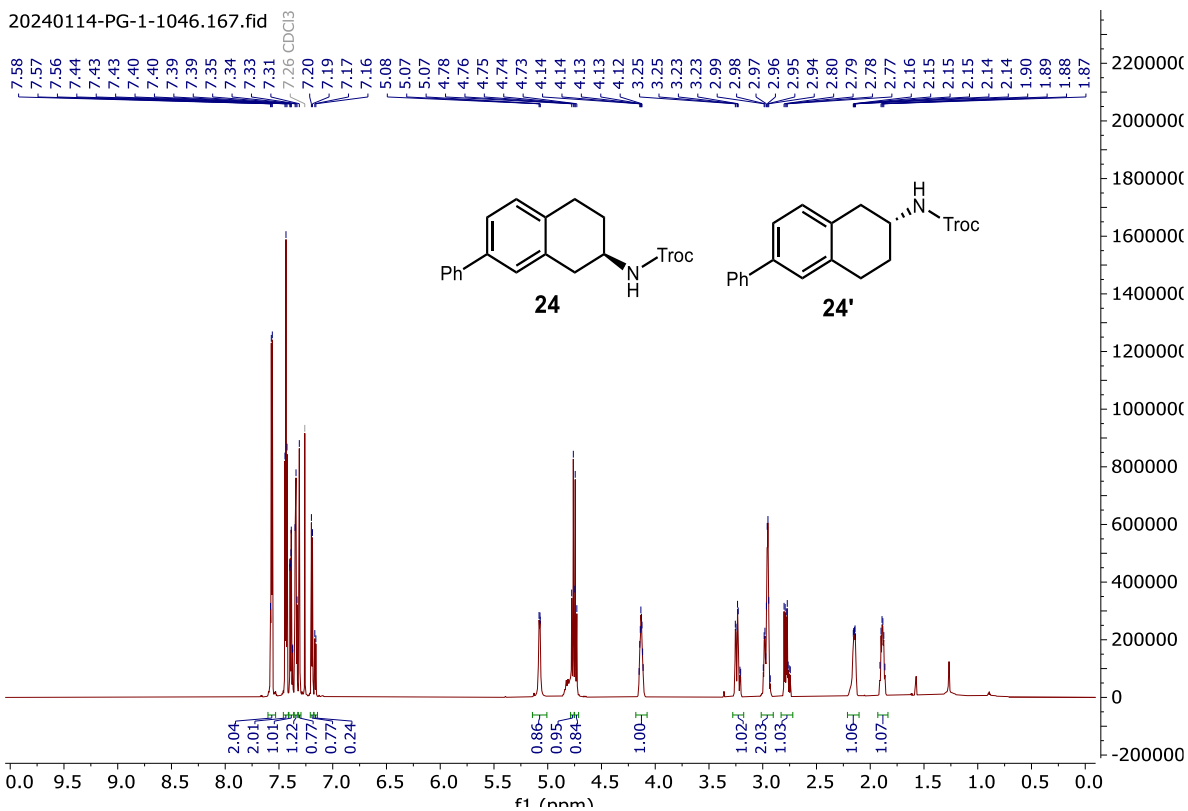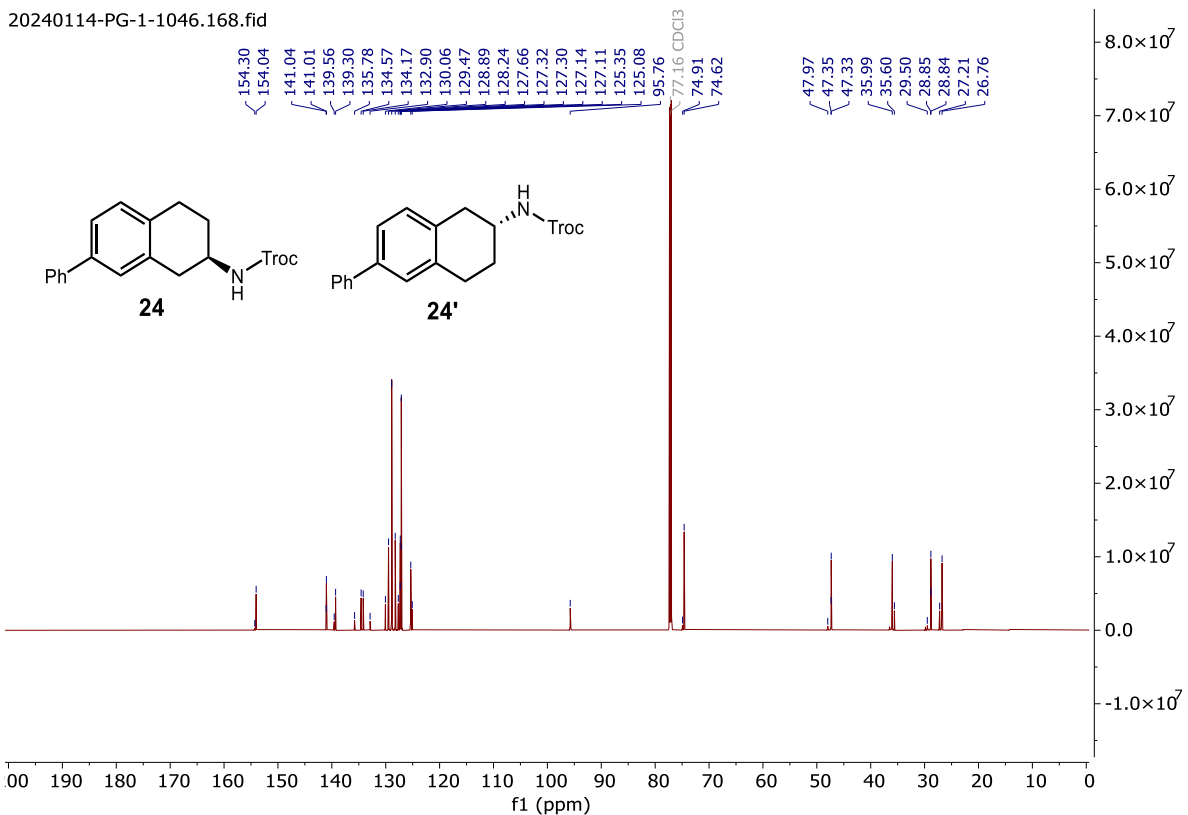

(24) and (24'):

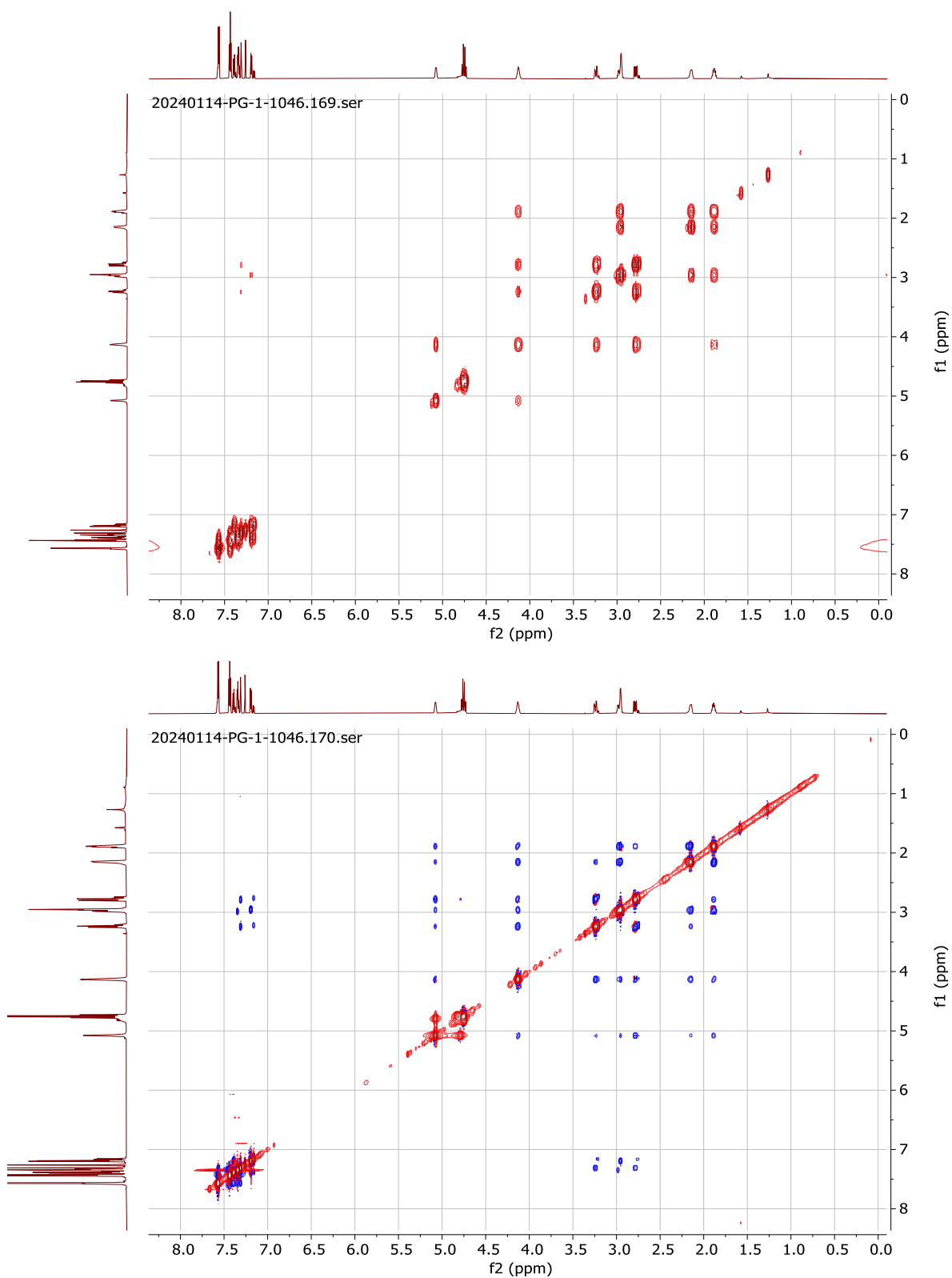

2,2,2-trichloroethyl (7-phenyl-1,2,3,4-tetrahydronaphthalen-2-yl)carbamate ($\pm$ -24)

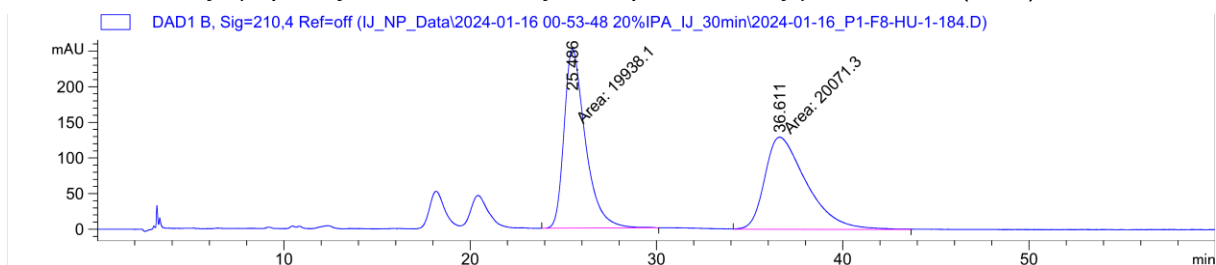

Signal 2: DAD1 B, Sig=210,4 Ref=off

Peak #	RetTime [min]	Type	Width [min]	Area [mAU*s]	Height [mAU]	Area %
1	25.486	MM T	1.3228	1.99381e4	251.21323	49.8335
2	36.611	MM T	2.5864	2.00713e4	129.33791	50.1665

Totals : 4.00095e4 380.55113

2,2,2-trichloroethyl (R)- (7-phenyl-1,2,3,4-tetrahydronaphthalen-2-yl)carbamate (24):

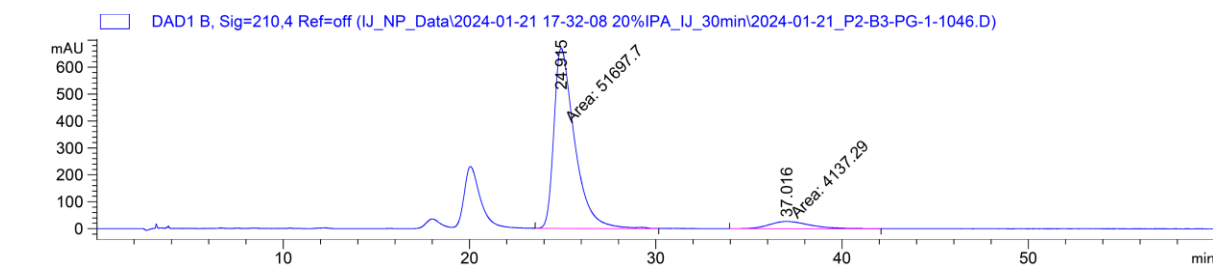

Signal 2: DAD1 B, Sig=210,4 Ref=off

Peak #	RetTime [min]	Type	Width [min]	Area [mAU*s]	Height [mAU]	Area %
1	24.915	MM T	1.2889	5.16977e4	668.48682	92.5901
2	37.016	MM T	2.5634	4137.29492	26.90002	7.4099

Totals : 5.58349e4 695.38684

2,2,2-trichloroethyl (6-phenyl-1,2,3,4-tetrahydronaphthalen-2-yl)carbamate ($\pm$ -24'):

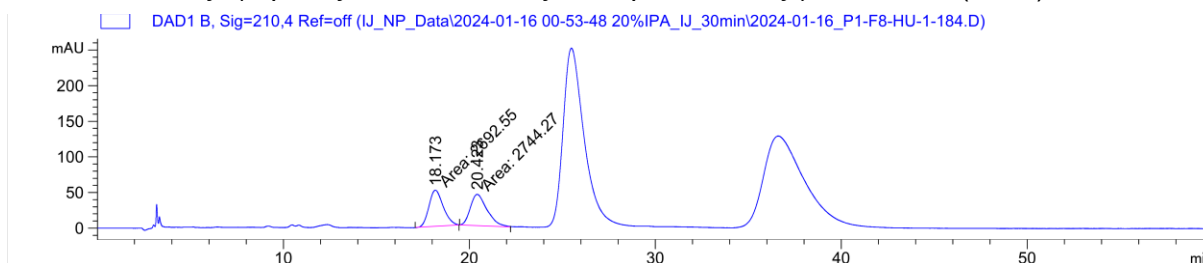

Signal 2: DAD1 B, Sig=210,4 Ref=off

Peak #	RetTime [min]	Type	Width [min]	Area [mAU*s]	Height [mAU]	Area %
1	18.173	MM	0.8862	2692.54590	50.63799	49.5243
2	20.423	MM T	1.0138	2744.27246	43.92320	50.4757

Totals : 5436.81836 94.56119

2,2,2-trichloroethyl (R)-(6-phenyl-1,2,3,4-tetrahydronaphthalen-2-yl)carbamate (24'):

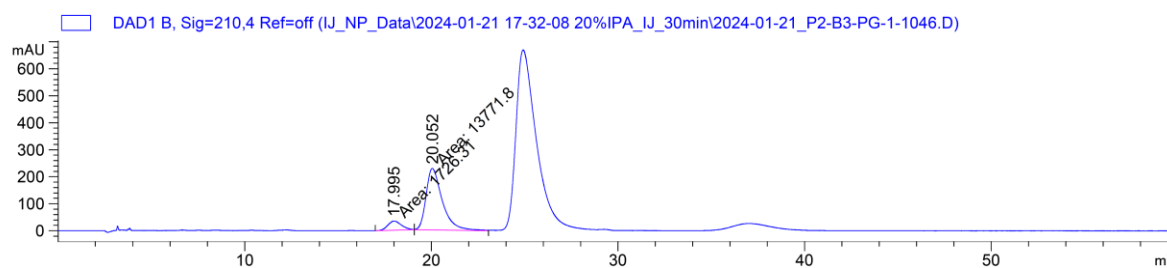

Signal 2: DAD1 B, Sig=210,4 Ref=off

Peak #	RetTime [min]	Type	Width [min]	Area [mAU*s]	Height [mAU]	Area %
1	17.995	MM T	0.8437	1726.30750	34.10238	11.1388
2	20.052	MM T	1.0072	1.37718e4	227.89500	88.8612

Totals : 1.54981e4 261.99738

2,2,2-trichloroethyl (R)-(5-bromo-1,2,3,4-tetrahydronaphthalen-2-yl)carbamate (**25**) and 2,2,2-trichloroethyl (R)-(8-bromo-1,2,3,4-tetrahydronaphthalen-2-yl)carbamate (**25'**):

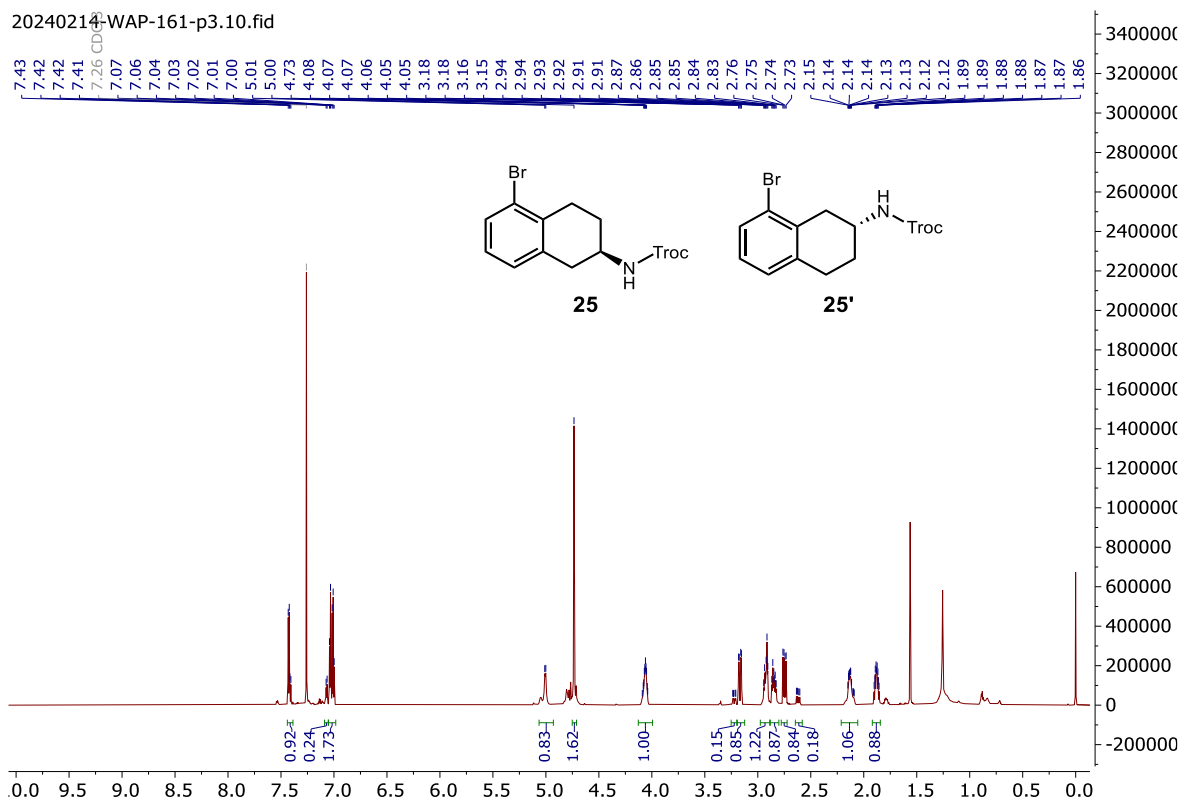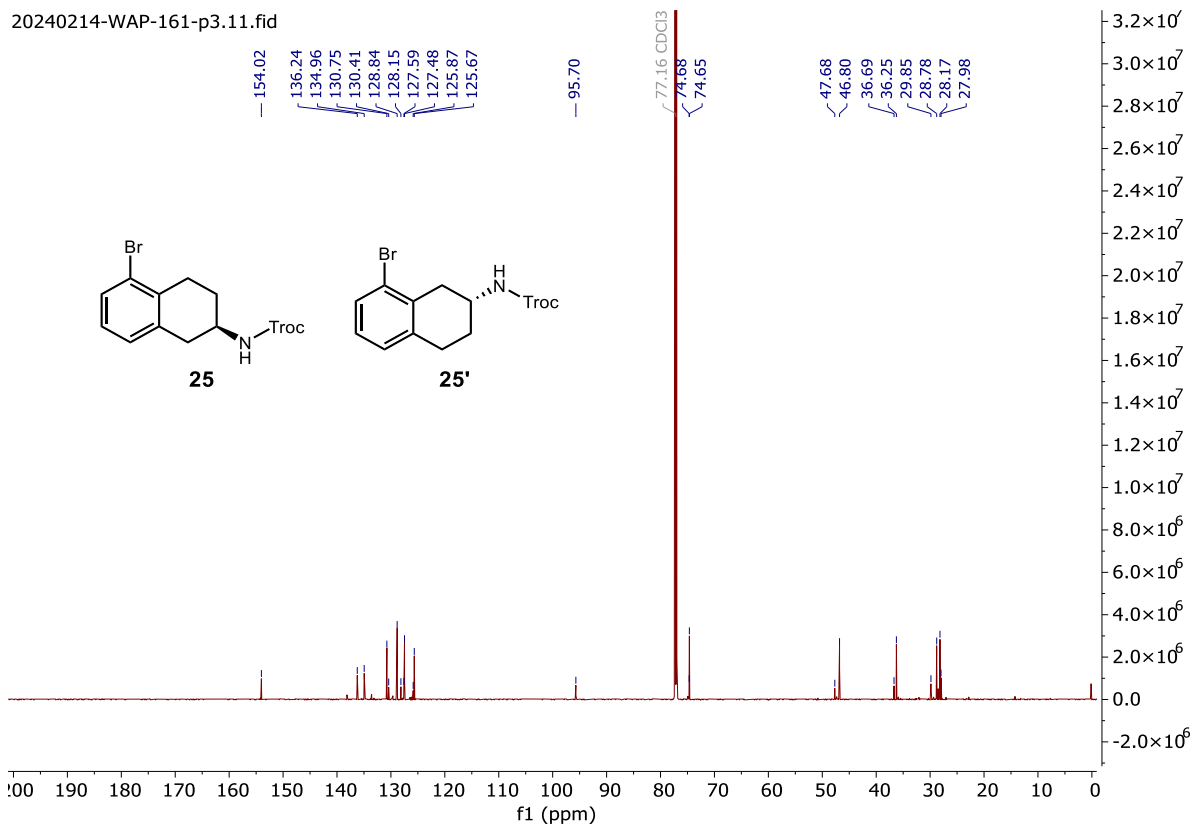

(25) and (25'):

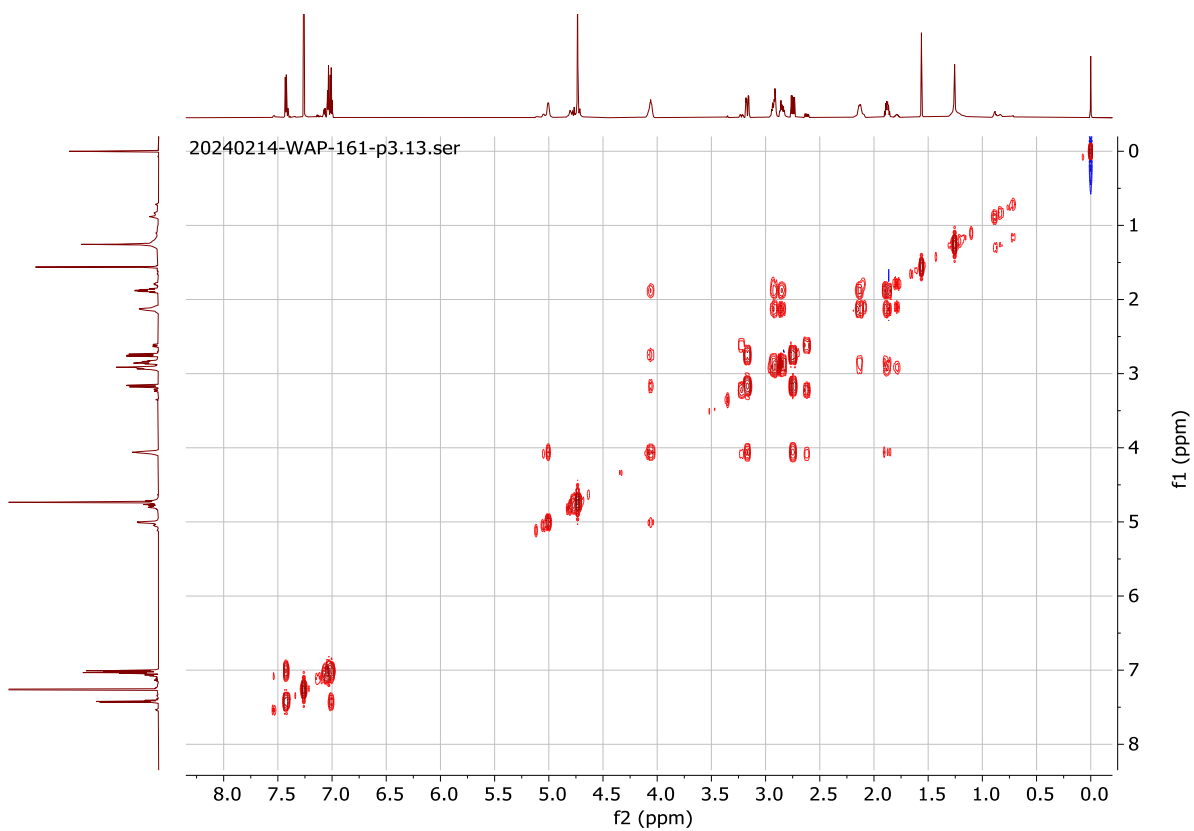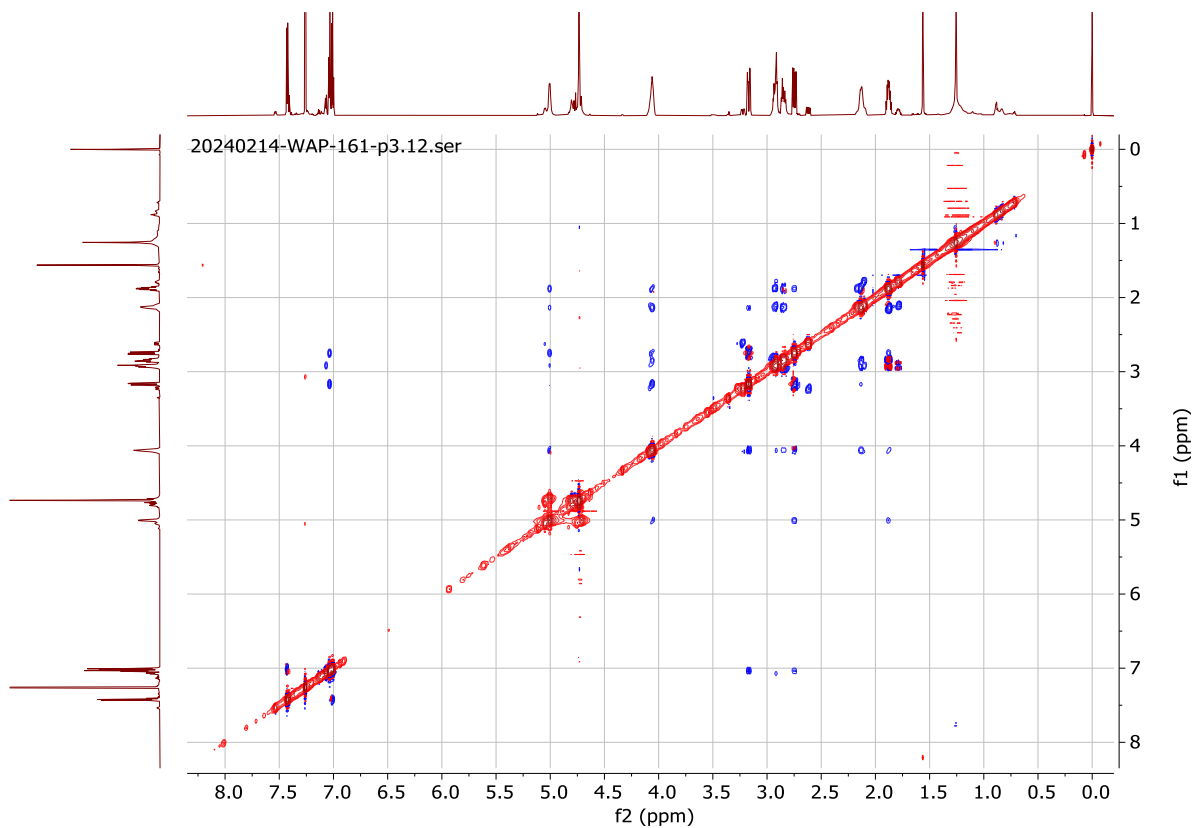

2,2,2-trichloroethyl (5-bromo-1,2,3,4-tetrahydronaphthalen-2-yl)carbamate ($\pm$ -25):

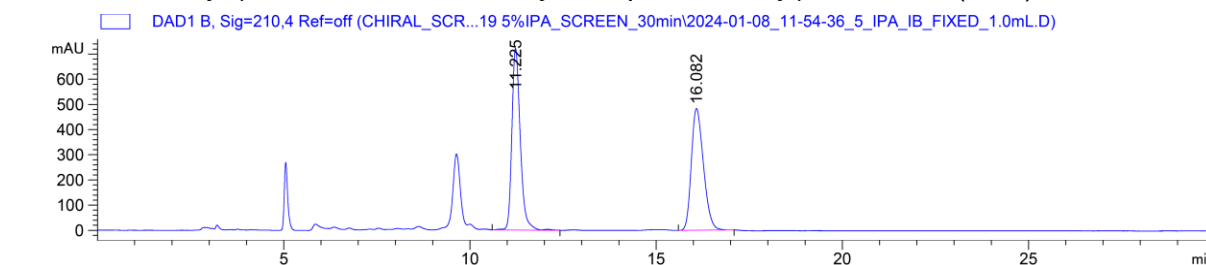

Signal 2: DAD1 B, Sig=210,4 Ref=off

Peak #	RetTime [min]	Type	Width [min]	Area [mAU*s]	Height [mAU]	Area %
1	11.225	VV R	0.2354	1.12944e4	721.33508	50.8184
2	16.082	BV R	0.2855	1.09306e4	483.89395	49.1816

Totals : 2.22250e4 1205.22903

2,2,2-trichloroethyl (R)-(5-bromo-1,2,3,4-tetrahydronaphthalen-2-yl)carbamate (25):

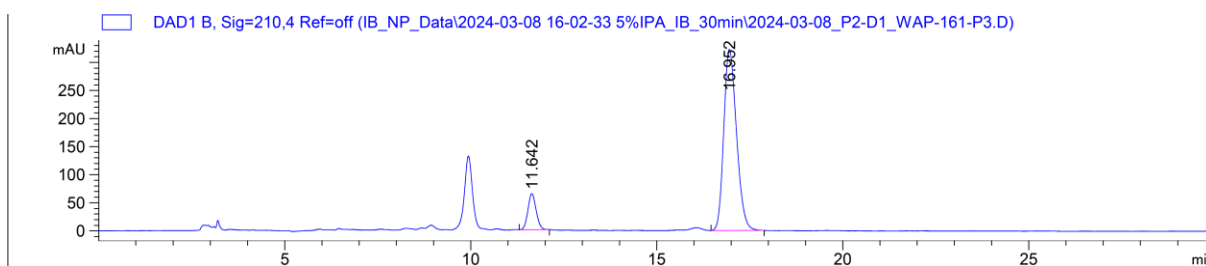

Signal 2: DAD1 B, Sig=210,4 Ref=off

Peak #	RetTime [min]	Type	Width [min]	Area [mAU*s]	Height [mAU]	Area %
1	11.642	BV R	0.1801	980.41797	64.21804	11.3562
2	16.952	BB	0.2793	7652.92725	322.51056	88.6438

Totals : 8633.34521 386.72860

2,2,2-trichloroethyl (R)-(6-methoxy-1,2,3,4-tetrahydronaphthalen-2-yl)carbamate (**26**) and 2,2,2-trichloroethyl (R)-(8-methoxy-1,2,3,4-tetrahydronaphthalen-2-yl)carbamate (**20**):

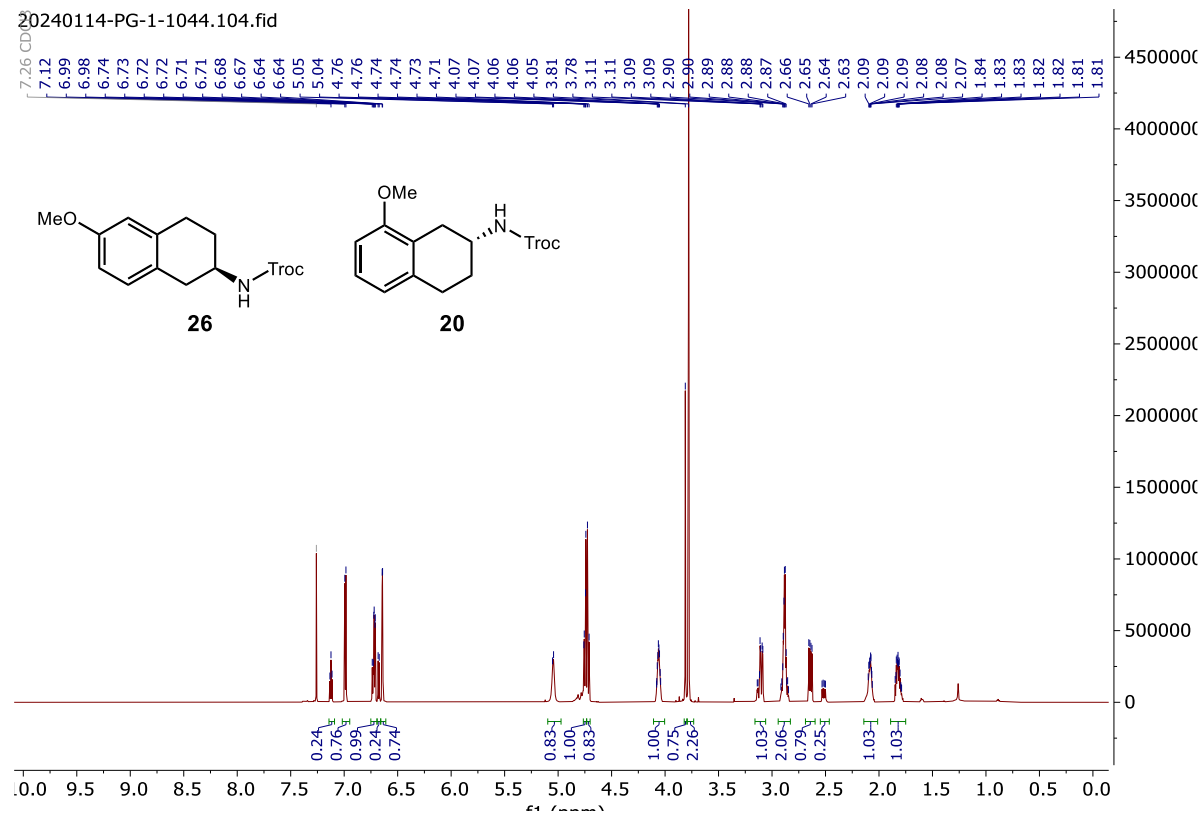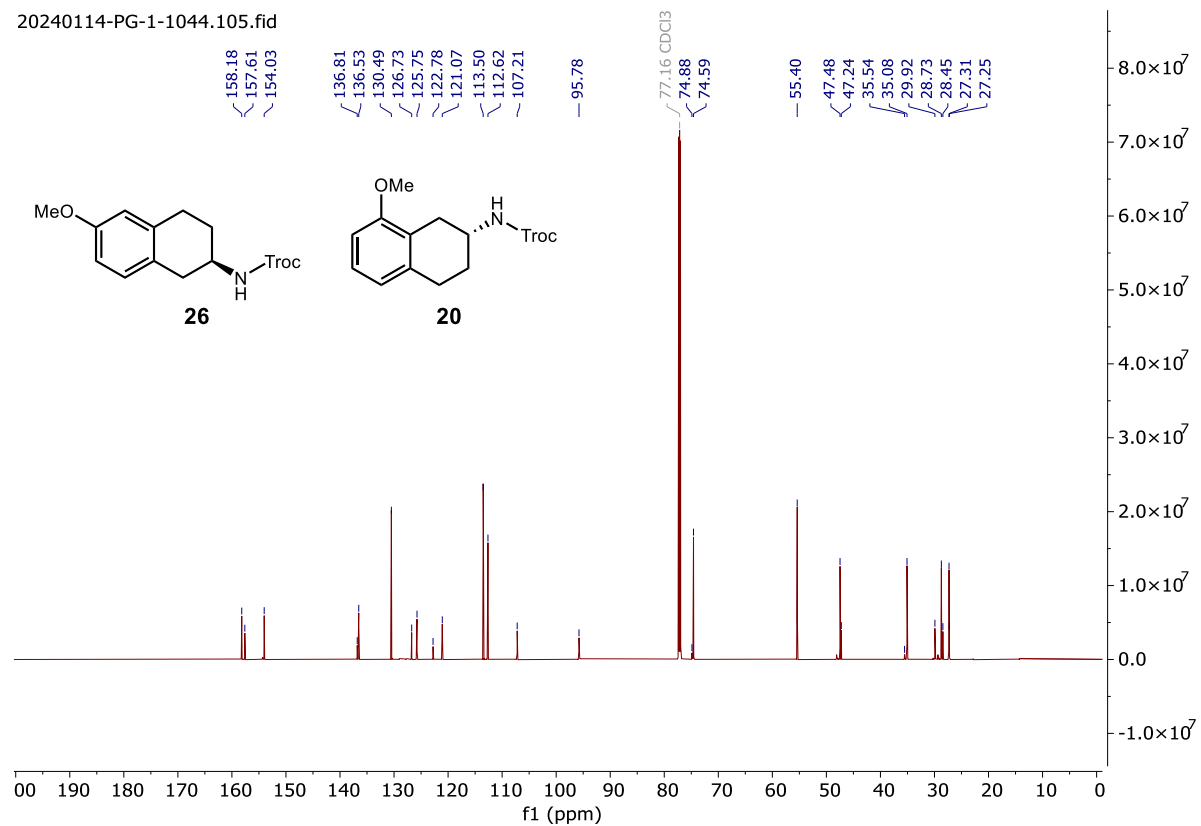

(26) and (20):

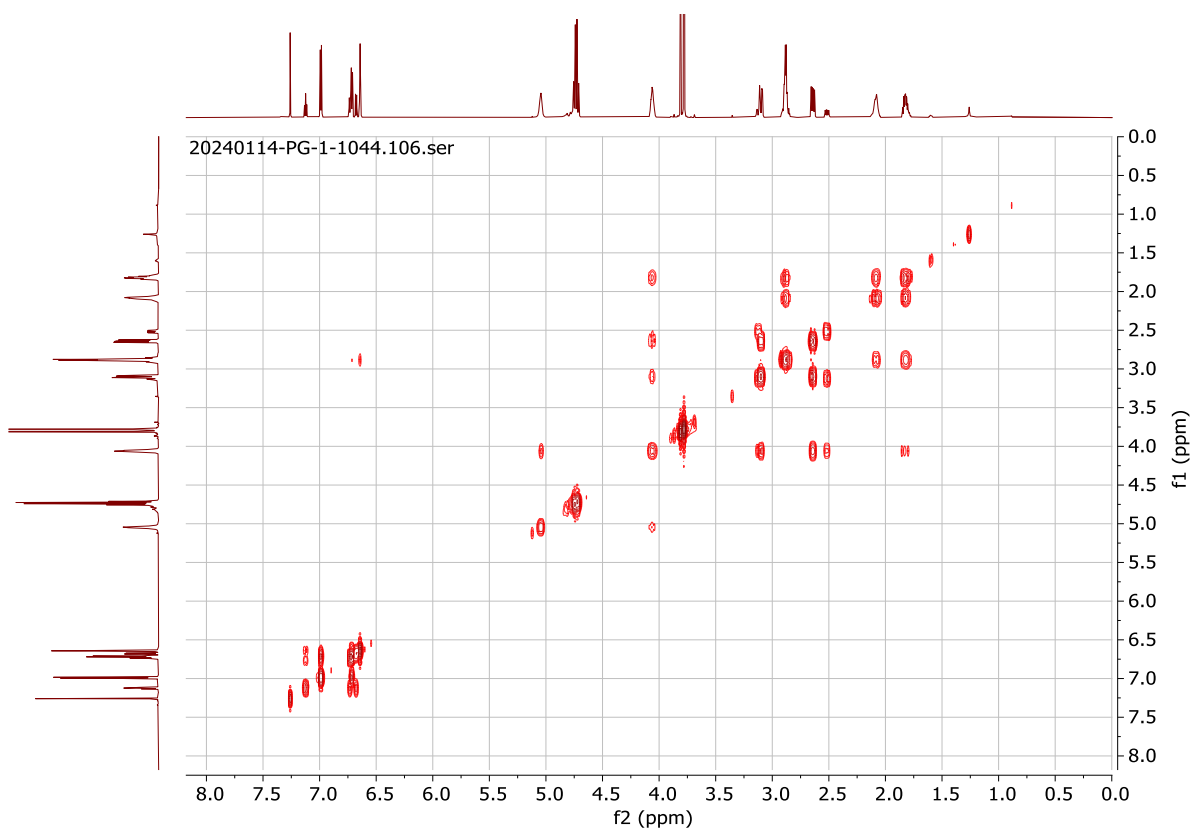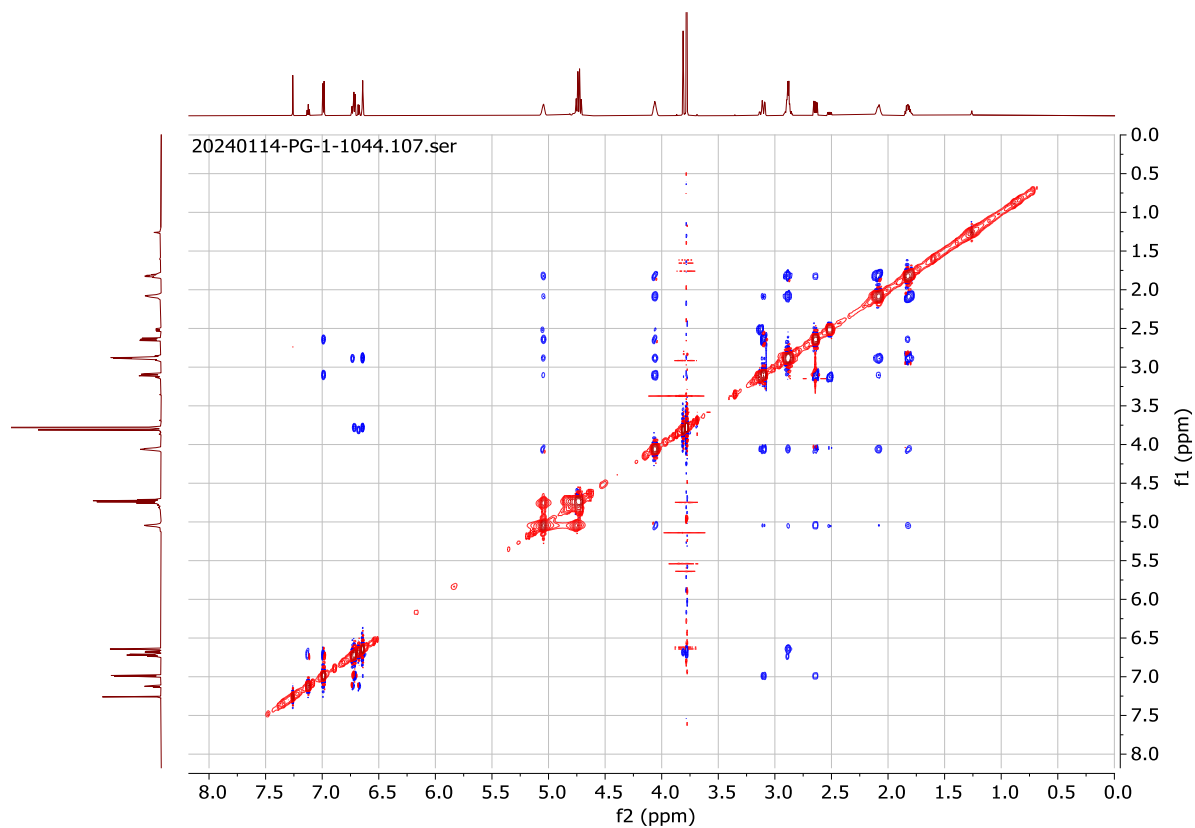

2,2,2-trichloroethyl (6-methoxy-1,2,3,4-tetrahydronaphthalen-2-yl)carbamate ($\pm$ -26):

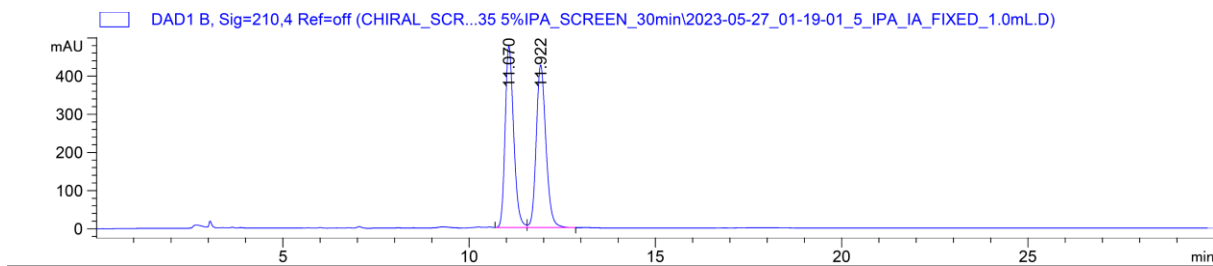

Signal 2: DAD1 B, Sig=210,4 Ref=off

Peak #	RetTime [min]	Type	Width [min]	Area [mAU*s]	Height [mAU]	Area %
1	11.070	BV	0.2391	7528.36133	474.48944	49.5182
2	11.922	VB	0.2683	7674.84766	425.82767	50.4818

Totals : 1.52032e4 900.31711

2,2,2-trichloroethyl (R)-(6-methoxy-1,2,3,4-tetrahydronaphthalen-2-yl)carbamate (26):

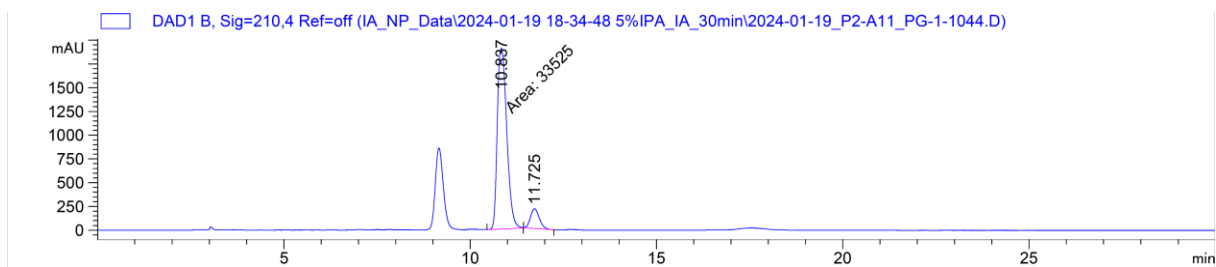

Signal 2: DAD1 B, Sig=210,4 Ref=off

Peak #	RetTime [min]	Type	Width [min]	Area [mAU*s]	Height [mAU]	Area %
1	10.837	MM T	0.2934	3.35250e4	1904.11755	90.7335
2	11.725	BB	0.2218	3423.86914	207.72342	9.2665

Totals : 3.69488e4 2111.84097

2,2,2-trichloroethyl (8-methoxy-1,2,3,4-tetrahydronaphthalen-2-yl)carbamate ($\pm$ -20):

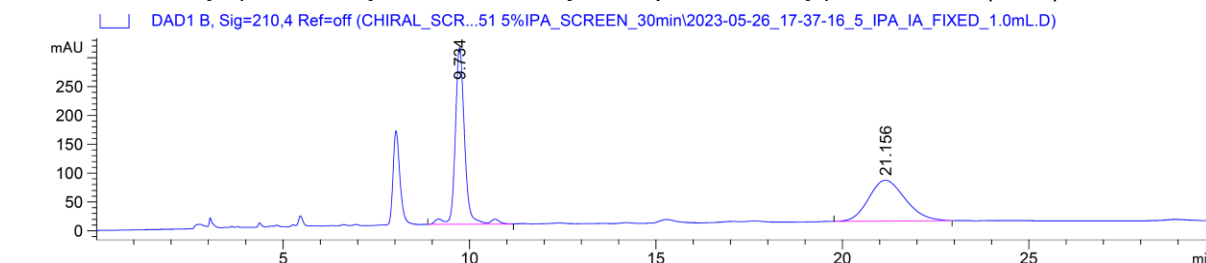

Signal 2: DAD1 B, Sig=210,4 Ref=off

Peak #	RetTime [min]	Type	Width [min]	Area [mAU*s]	Height [mAU]	Area %
1	9.734	VV R	0.2519	5403.88330	306.12808	53.0699
2	21.156	BV R	0.7940	4778.69287	70.52479	46.9301

Totals : 1.01826e4 376.65287

2,2,2-trichloroethyl (R)-(8-methoxy-1,2,3,4-tetrahydronaphthalen-2-yl)carbamate (20):

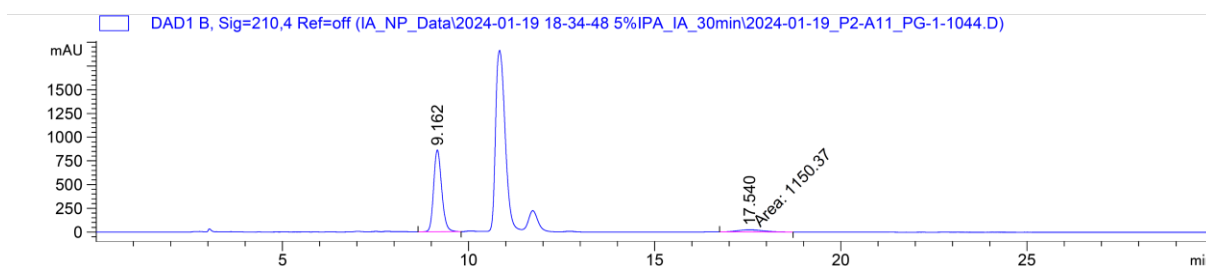

Signal 2: DAD1 B, Sig=210,4 Ref=off

Peak #	RetTime [min]	Type	Width [min]	Area [mAU*s]	Height [mAU]	Area %
1	9.162	BB	0.2231	1.29499e4	863.01361	91.8415
2	17.540	MM T	0.8418	1150.37000	22.77640	8.1585

Totals : 1.41003e4 885.79001

2,2,2-trichloroethyl (R)-(6,8-dimethoxy-1,2,3,4-tetrahydronaphthalen-2-yl)carbamate (**27**):

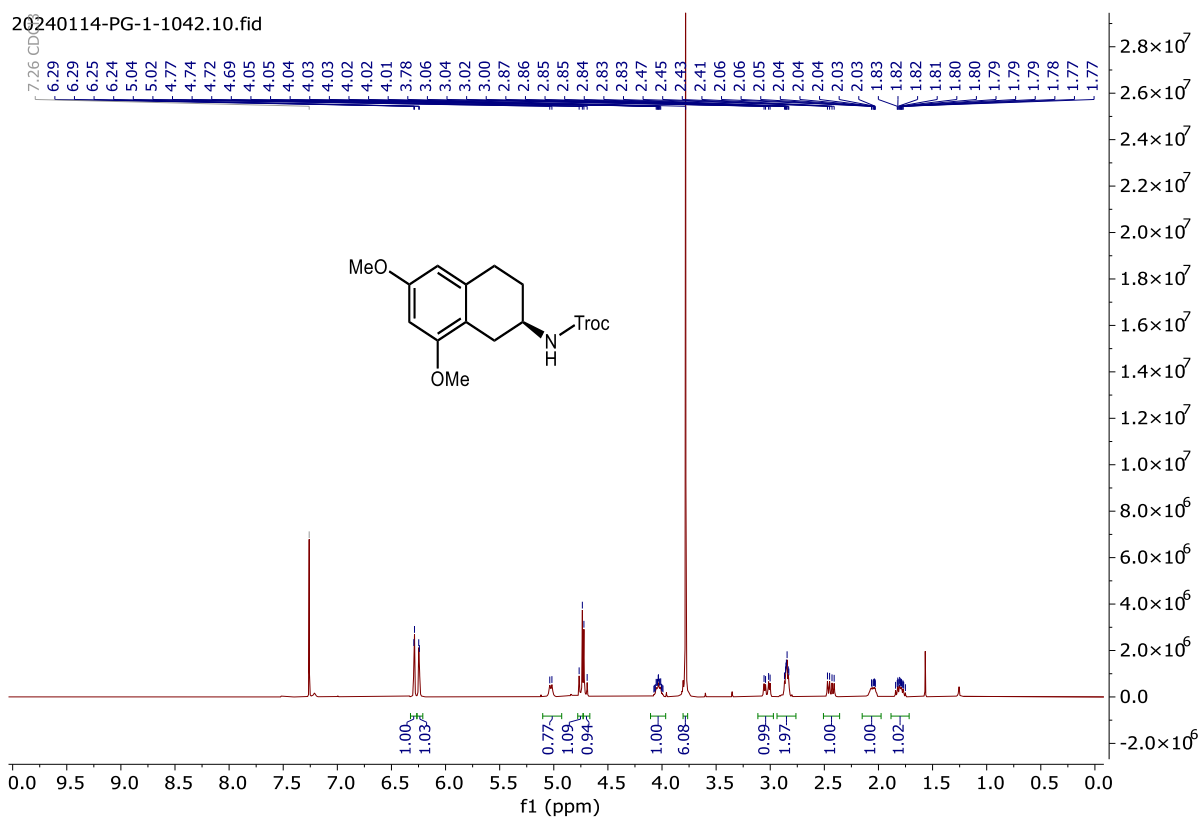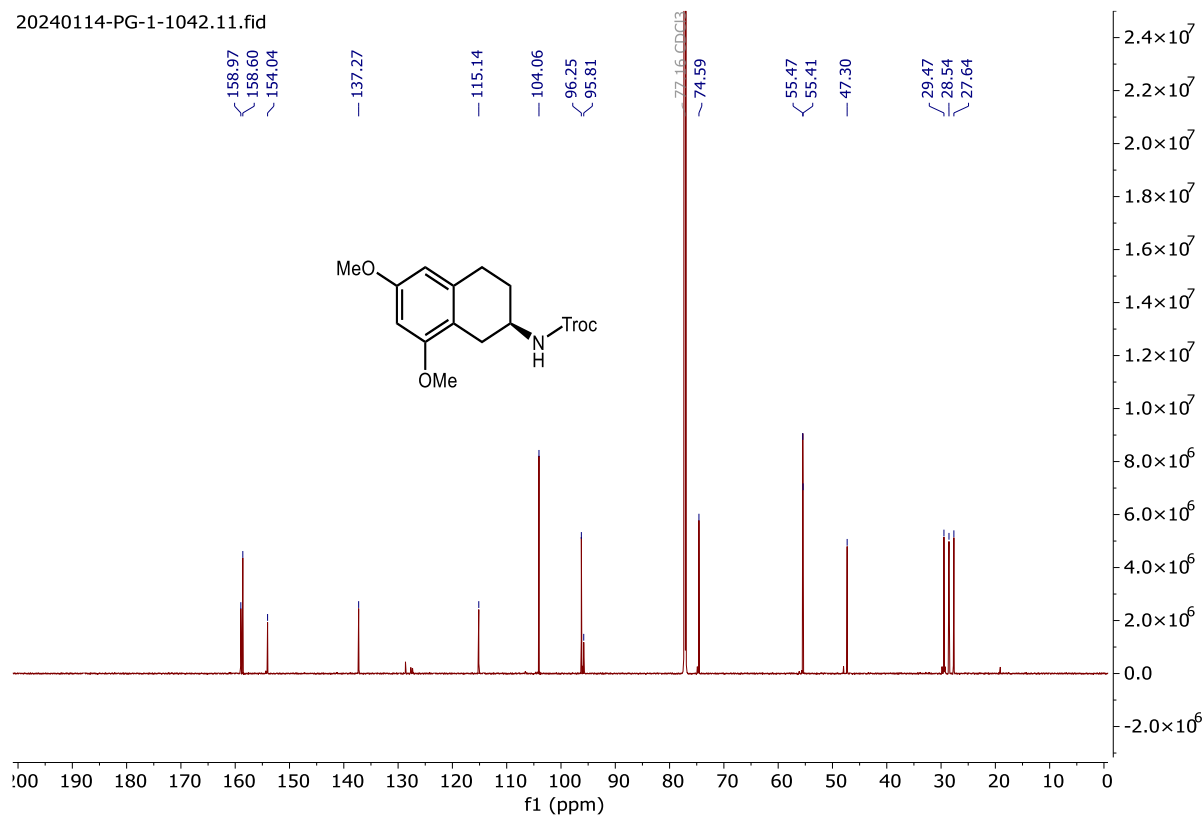

2,2,2-trichloroethyl (6,8-dimethoxy-1,2,3,4-tetrahydronaphthalen-2-yl)carbamate ($\pm$ -27):

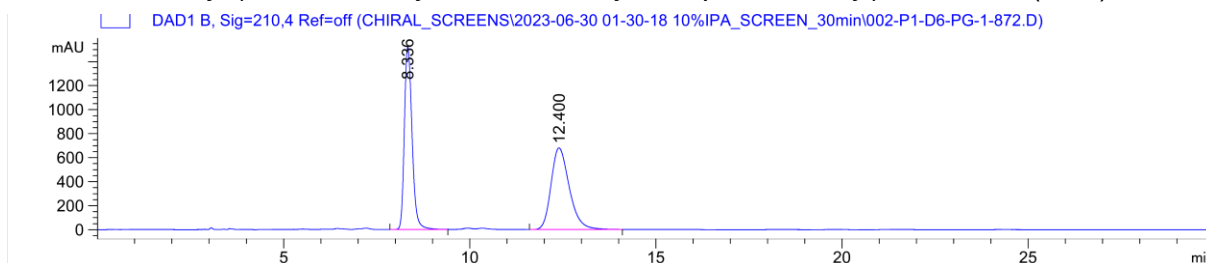

Signal 2: DAD1 B, Sig=210,4 Ref=off

Peak #	RetTime [min]	Type	Width [min]	Area [mAU*s]	Height [mAU]	Area %
1	8.336	BB	0.2056	2.18494e4	1516.03369	48.6000
2	12.400	BB	0.4063	2.31082e4	680.35248	51.4000

Totals : 4.49576e4 2196.38617

2,2,2-trichloroethyl (R)-(6,8-dimethoxy-1,2,3,4-tetrahydronaphthalen-2-yl)carbamate (27):

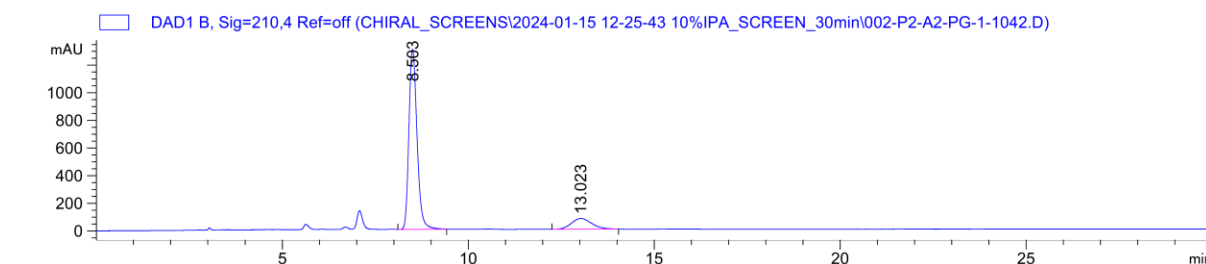

Signal 2: DAD1 B, Sig=210,4 Ref=off

Peak #	RetTime [min]	Type	Width [min]	Area [mAU*s]	Height [mAU]	Area %
1	8.503	BV R	0.1974	1.97550e4	1302.82568	87.0335
2	13.023	VV R	0.4447	2943.16187	77.56516	12.9665

Totals : 2.26982e4 1380.39084

2,2,2-trichloroethyl (R)-(1,2,3,4-tetrahydrophenanthren-3-yl)carbamate (**28**) and 2,2,2-trichloroethyl (R)-(1,2,3,4-tetrahydrophenanthren-2-yl)carbamate (**28'**):

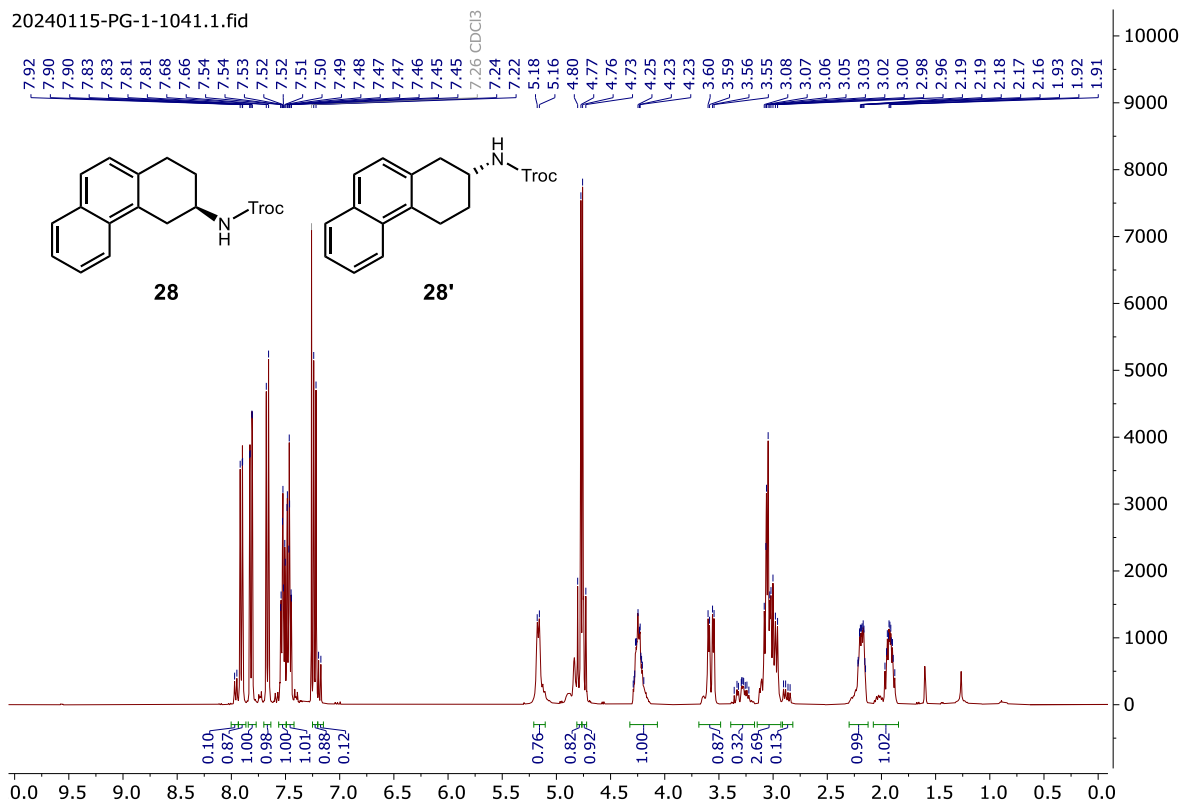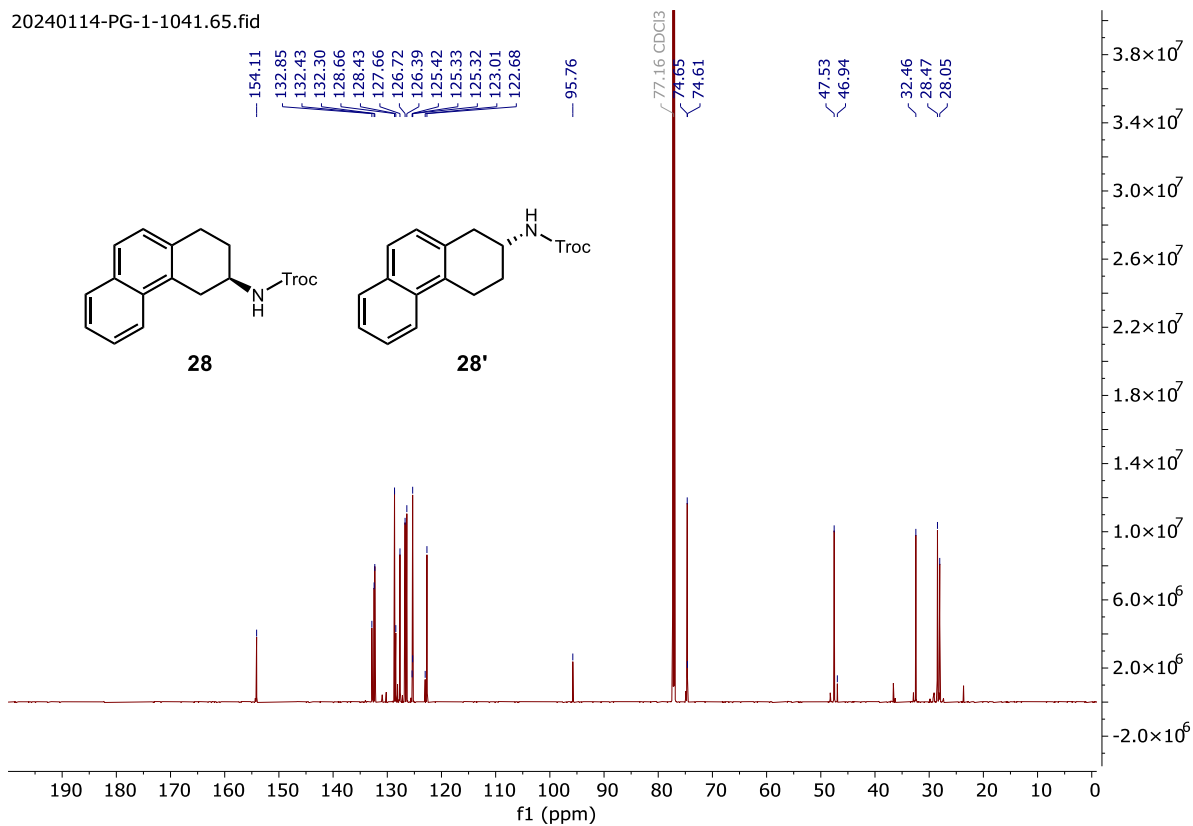

(28) and (28'):

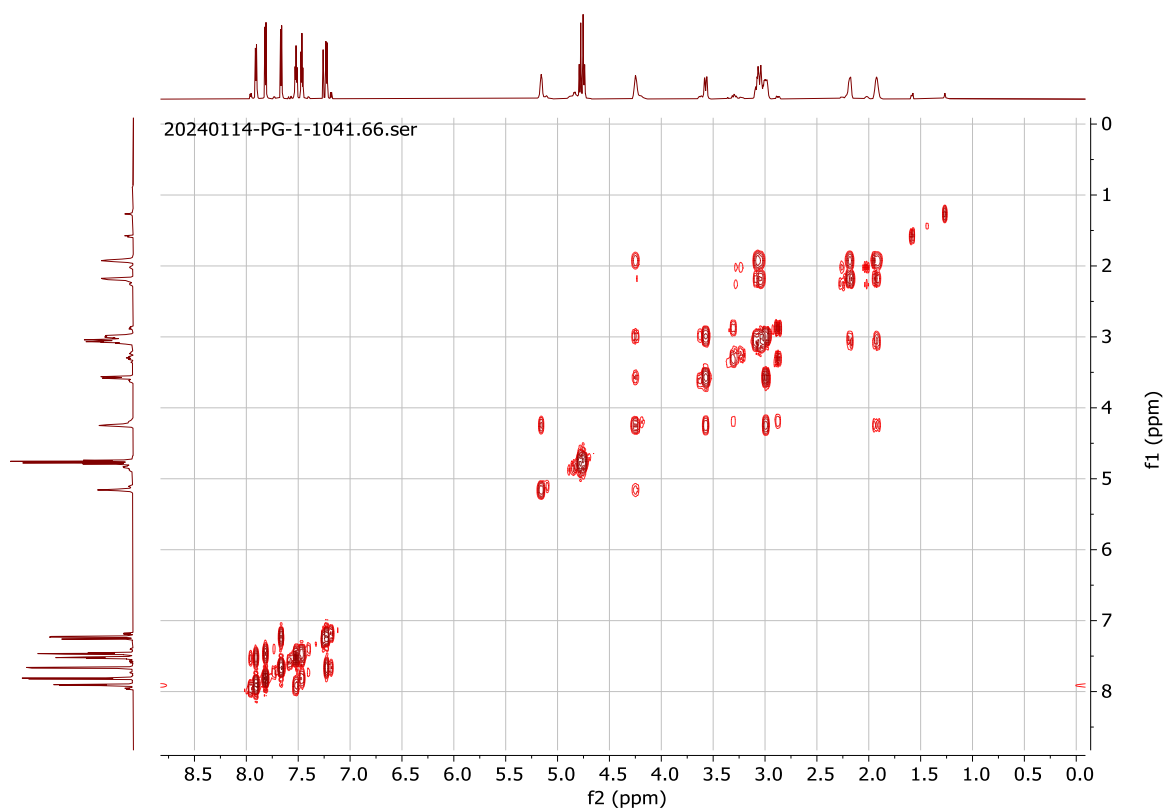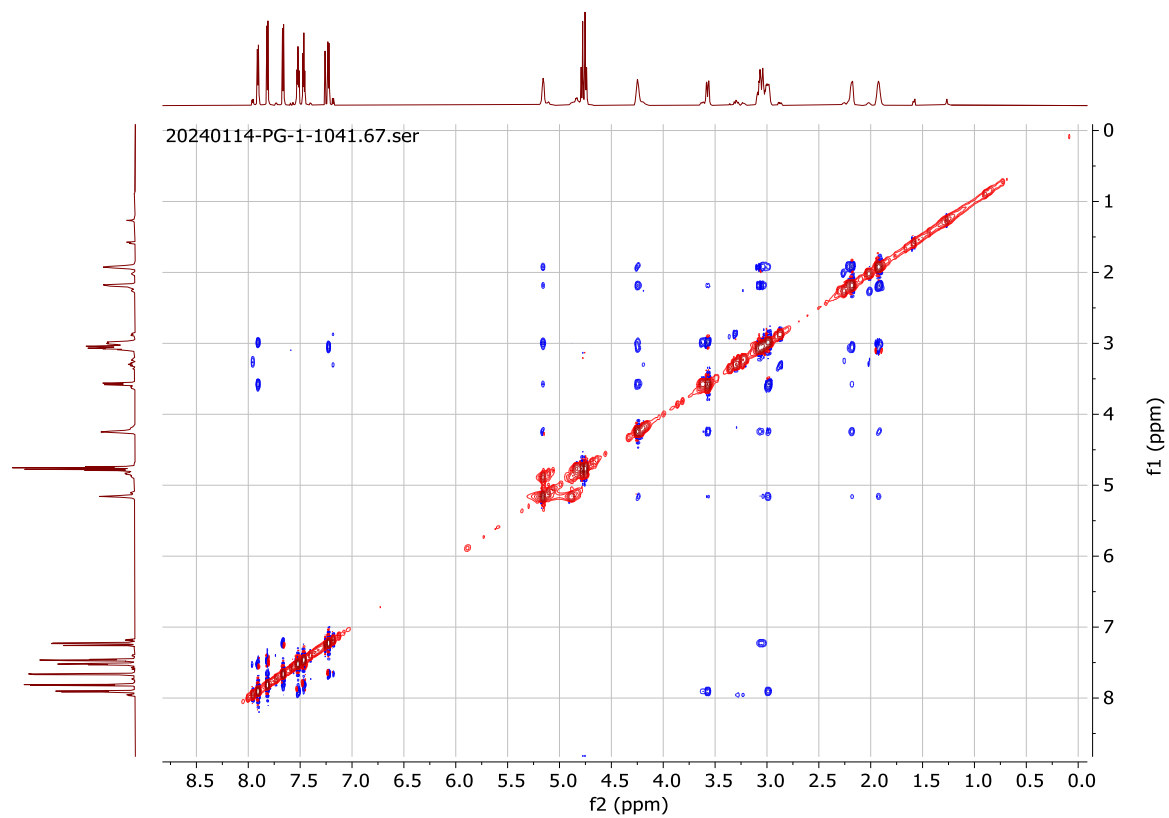

2,2,2-trichloroethyl (1,2,3,4-tetrahydrophenanthren-3-yl)carbamate ($\pm$ -28):

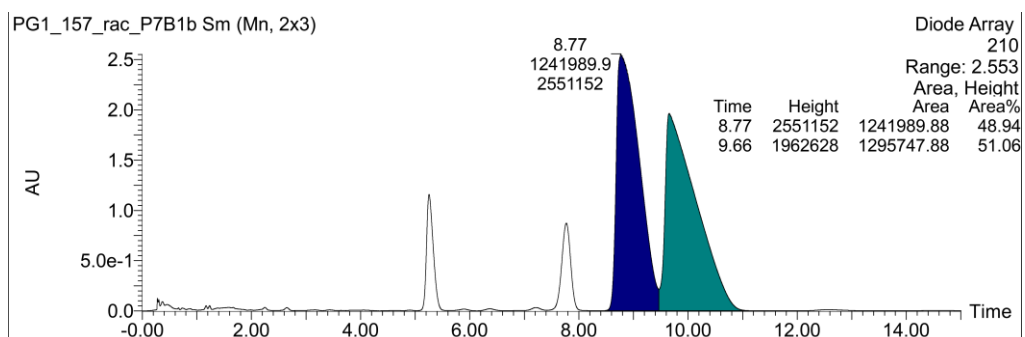

2,2,2-trichloroethyl (R)-(1,2,3,4-tetrahydrophenanthren-3-yl)carbamate (28):

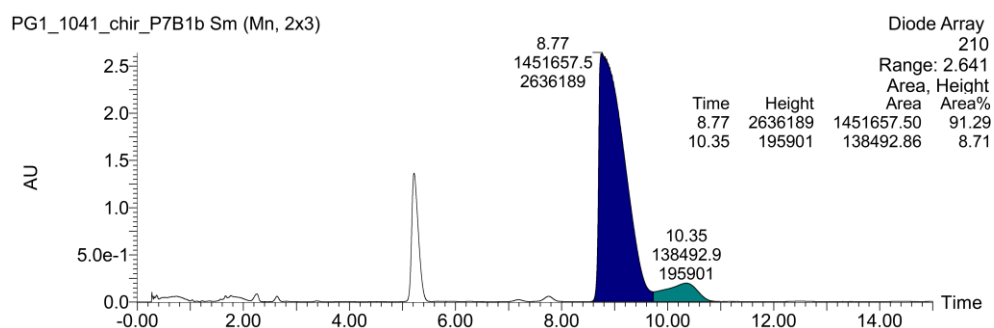

2,2,2-trichloroethyl (R)-(1,2,3,4-tetrahydrophenanthren-2-yl)carbamate ($\pm$ -28'):

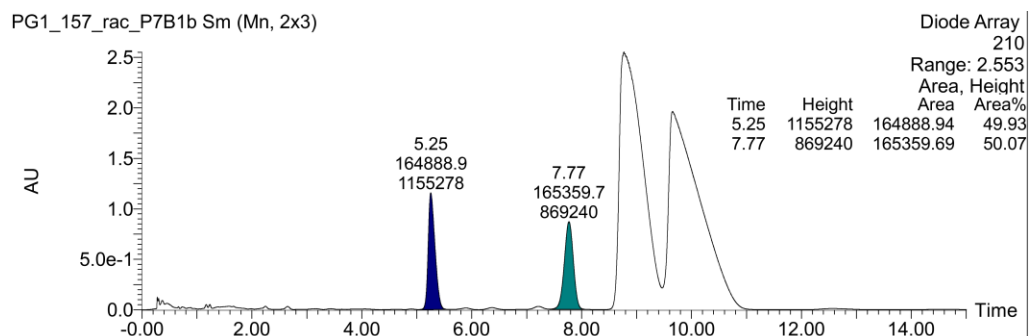

2,2,2-trichloroethyl (R)-(1,2,3,4-tetrahydrophenanthren-2-yl)carbamate (28'):

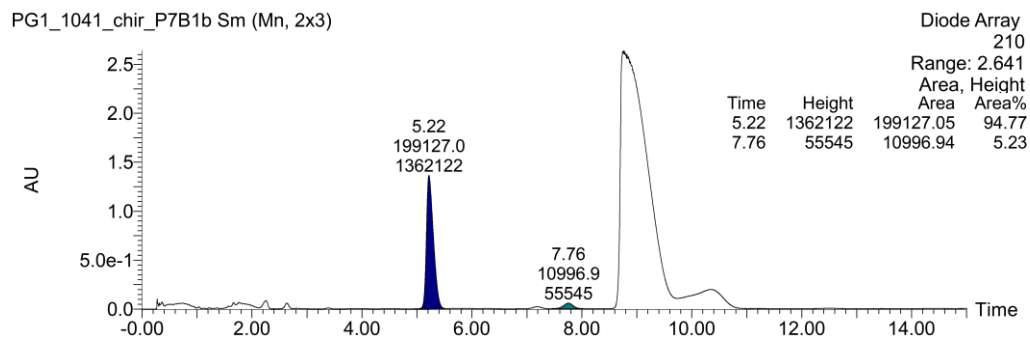

2,2,2-trichloroethyl (R)-(3-tosyl-6,7,8,9-tetrahydro-3H-benzo[e]indol-7-yl)carbamate (29) and 2,2,2-trichloroethyl (R)-(3-tosyl-6,7,8,9-tetrahydro-3H-benzo[e]indol-8-yl)carbamate (29')

20240123-PG-1-1049.1.fid

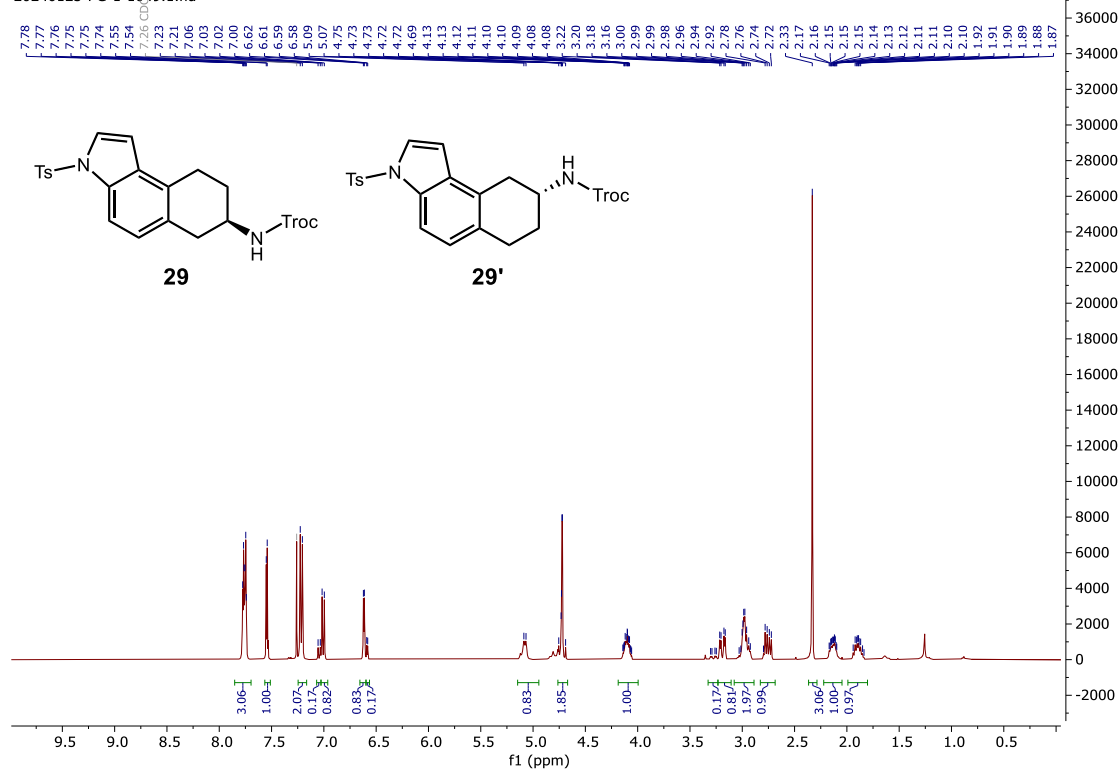

20240114-PG-1-1049.298.fid

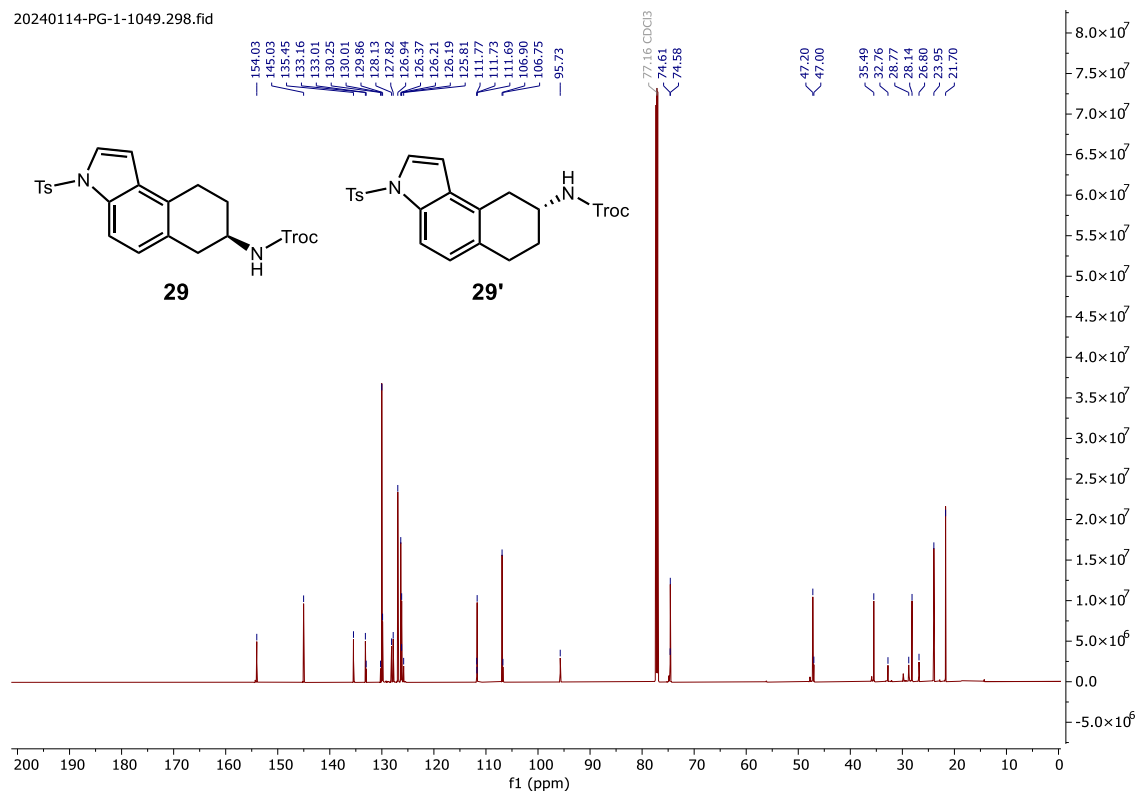

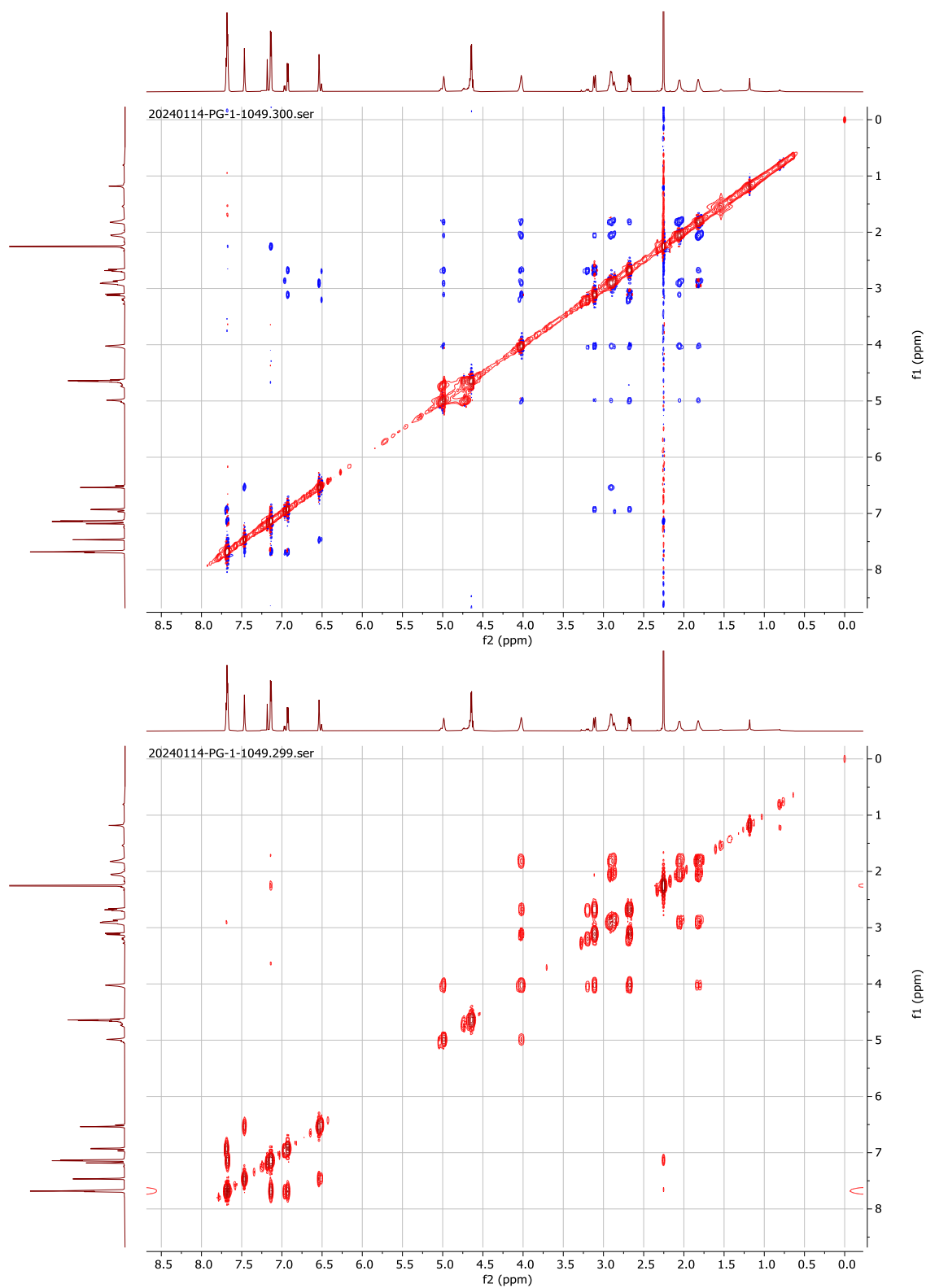

2,2,2-trichloroethyl (R)-(3-tosyl-6,7,8,9-tetrahydro-3H-benzo[e]indol-7-yl)carbamate ($\pm$ -29)

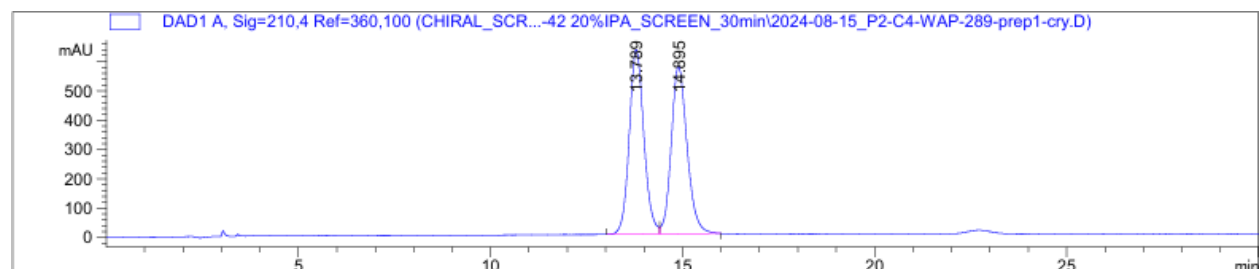

Signal 1: DAD1 A, Sig=210,4 Ref=360,100

Peak #	RetTime [min]	Type	Width [min]	Area [mAU*s]	Height [mAU]	Area %
1	13.789	VV R	0.3179	1.66882e4	629.32239	50.4955
2	14.895	VV R	0.3371	1.63607e4	570.50861	49.5045

mal Phase 8/20/2024 11:54:29 AM SYSTEM

a File C:\Users\P...5 09-00-42 20%IPA_SCREEN_30min\2024-08-
ple Name: WAP-289-prep1-cry

Totals : 3.30489e4 1199.83099

2,2,2-trichloroethyl (R)-(3-tosyl-6,7,8,9-tetrahydro-3H-benzo[e]indol-7-yl)carbamate (29)

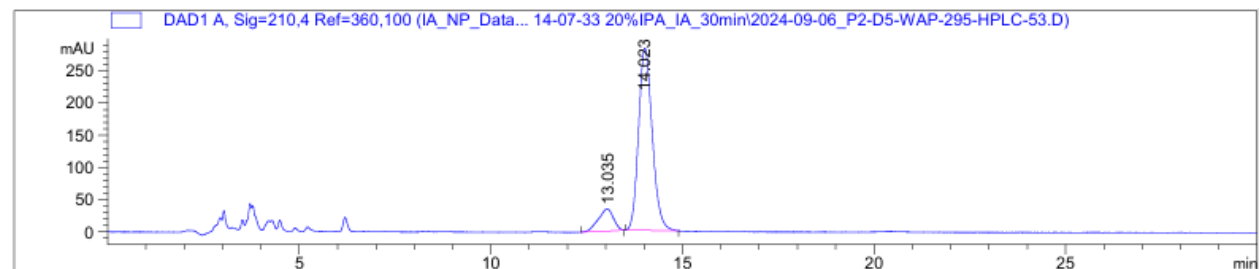

Signal 1: DAD1 A, Sig=210,4 Ref=360,100

Peak #	RetTime [min]	Type	Width [min]	Area [mAU*s]	Height [mAU]	Area %
1	13.035	VV R	0.3263	949.52911	34.41939	11.9215
2	14.023	VV R	0.2958	7015.34131	282.91034	88.0785

nal Phase 9/14/2024 3:41:34 PM SYSTEM

i File C:\Users\P...4-09-06 14-07-33 20%IPA_IA_30min\2024-0
ple Name: WAP-295-HPLC-53

Totals : 7964.87042 317.32973

2,2,2-trichloroethyl (R)-(1,2,3,4-tetrahydrodibenzo[b,d]furan-2-yl)carbamate (**30**):

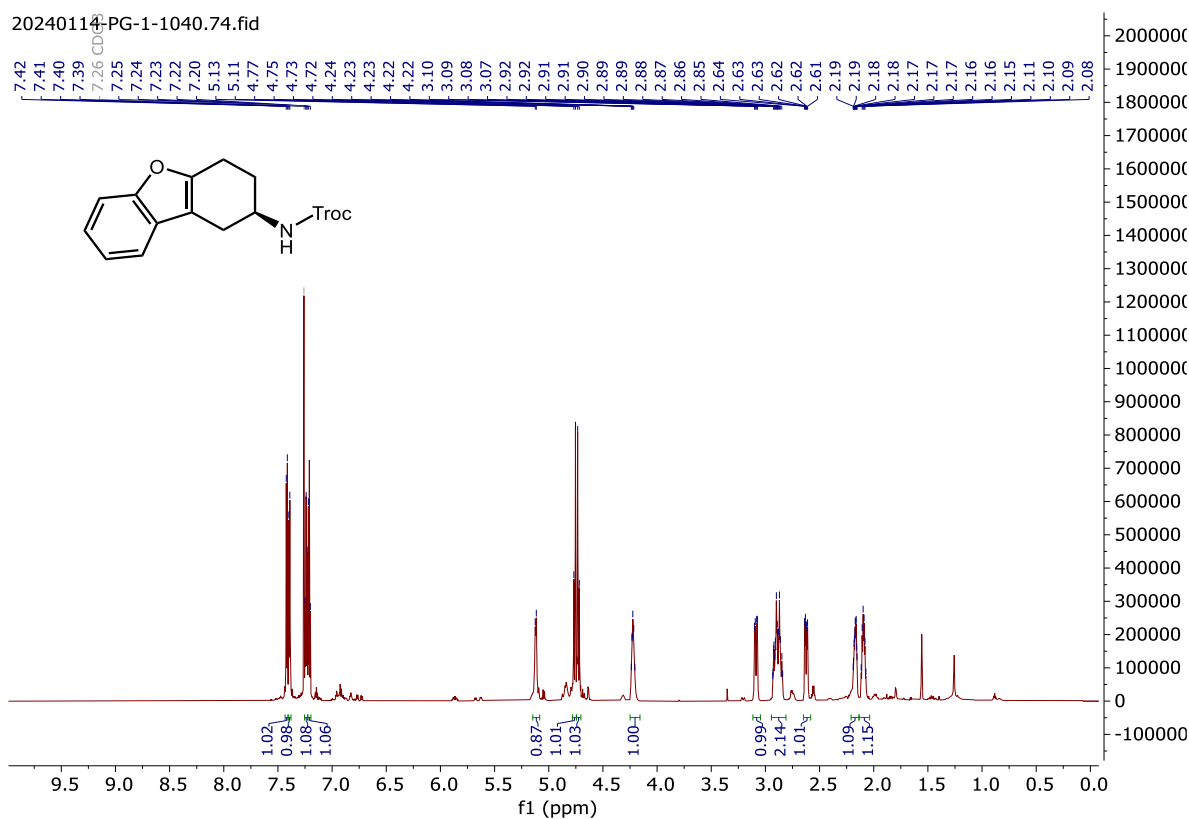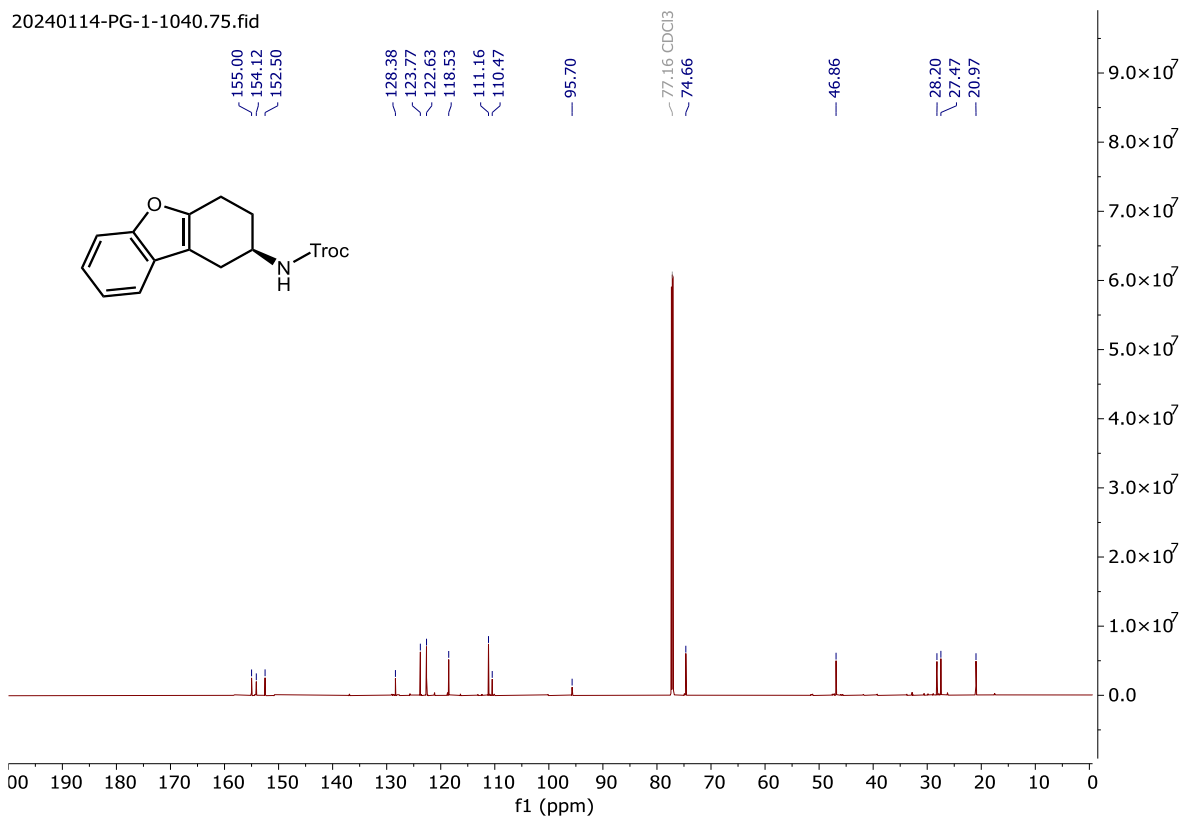

(30):

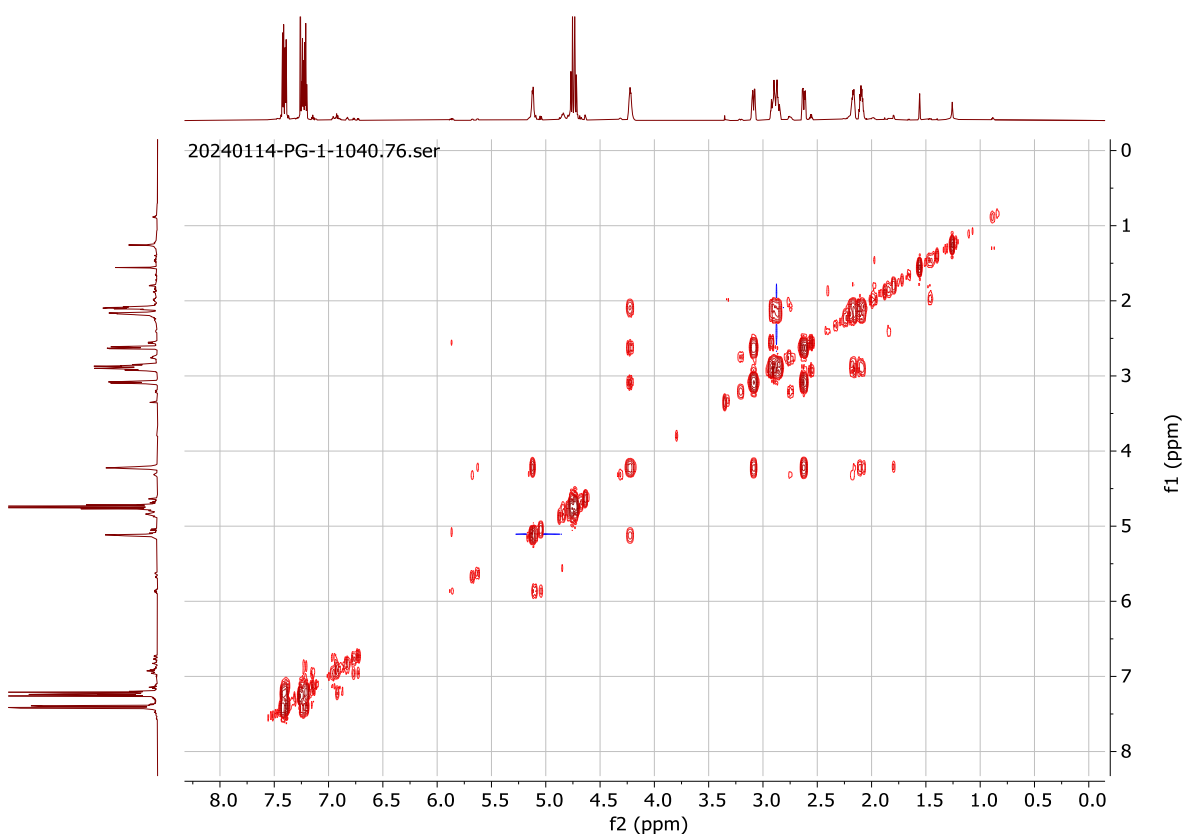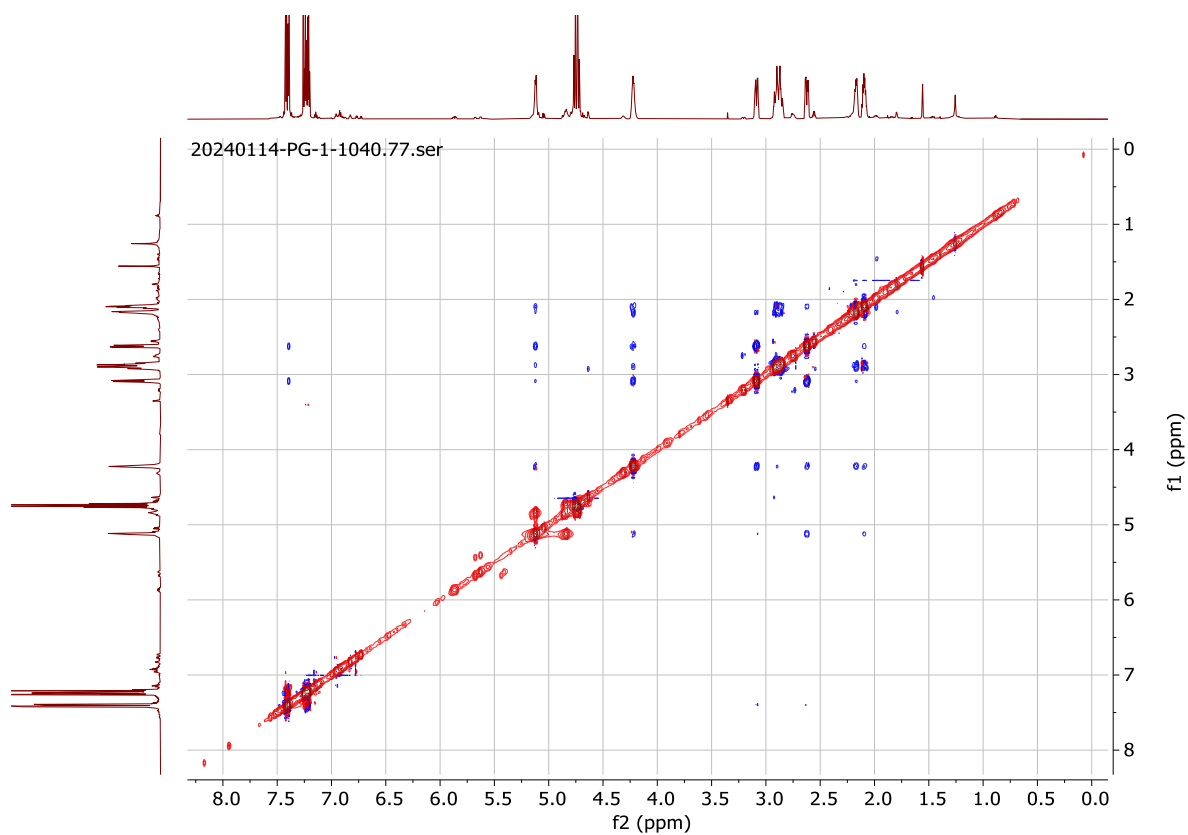

2,2,2-trichloroethyl (1,2,3,4-tetrahydrodibenzo[b,d]furan-2-yl)carbamate ($\pm$ -30):

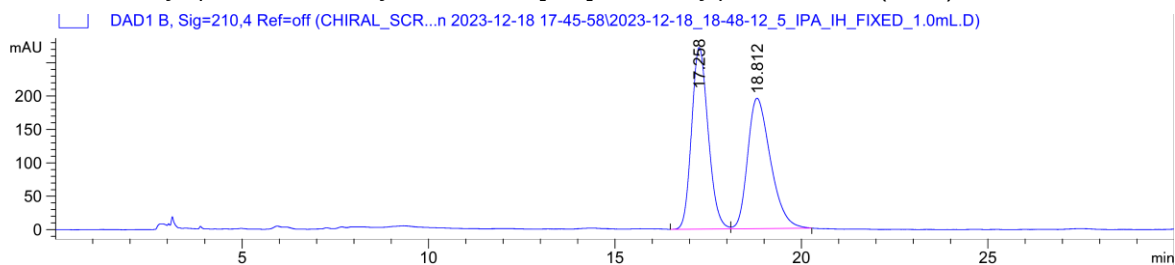

Signal 2: DAD1 B, Sig=210,4 Ref=off

Peak #	RetTime [min]	Type	Width [min]	Area [mAU*s]	Height [mAU]	Area %
1	17.258	VV R	0.3623	8411.32813	271.97485	50.2865
2	18.812	VV R	0.4986	8315.49414	195.37236	49.7135

Totals : 1.67268e4 467.34721

2,2,2-trichloroethyl (R)-(1,2,3,4-tetrahydrodibenzo[b,d]furan-2-yl)carbamate (30):

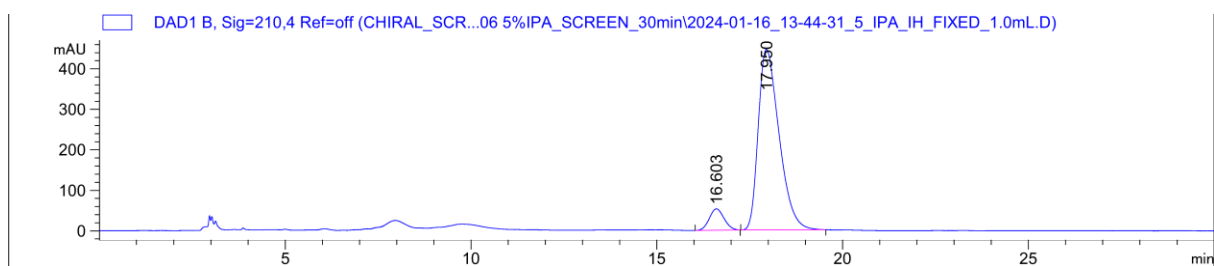

Signal 2: DAD1 B, Sig=210,4 Ref=off

Peak #	RetTime [min]	Type	Width [min]	Area [mAU*s]	Height [mAU]	Area %
1	16.603	BB	0.3248	1463.62378	52.97777	7.7664
2	17.950	BV R	0.4572	1.73819e4	445.93954	92.2336

Totals : 1.88455e4 498.91731

2,2,2-trichloroethyl (R)-(8-oxospiro[4.5]deca-6,9-dien-2-yl)carbamate (**31**):

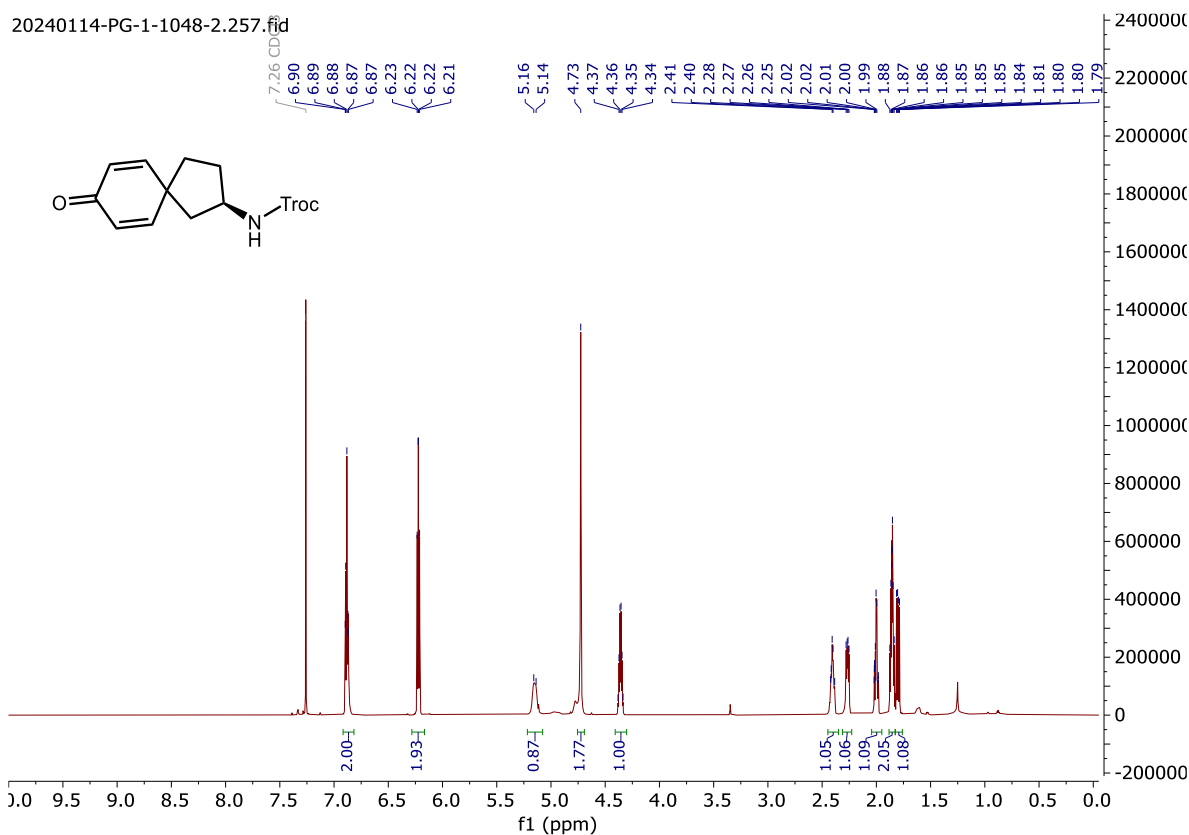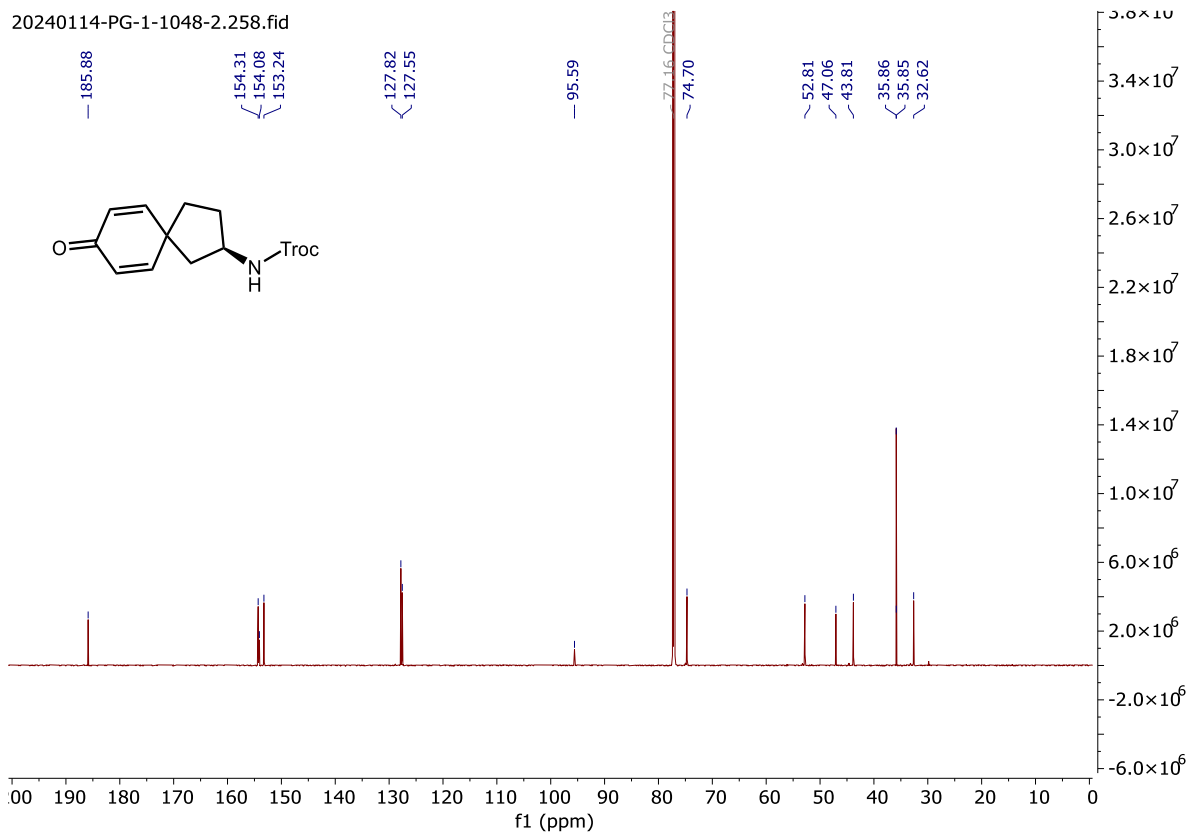

2,2,2-trichloroethyl (8-oxospiro[4.5]deca-6,9-dien-2-yl)carbamate ($\pm$ -31):

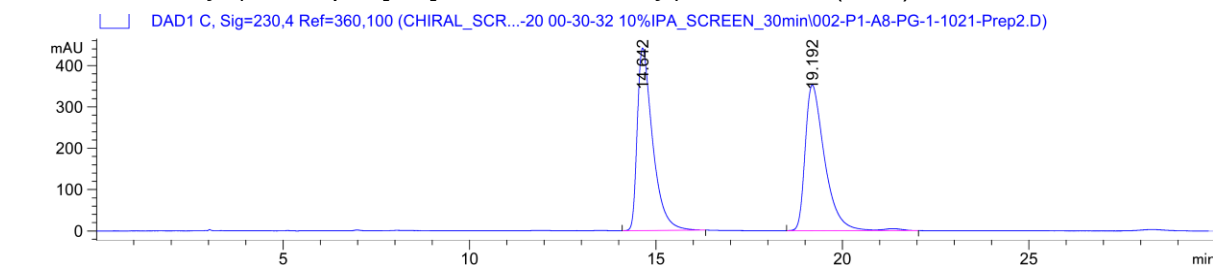

Signal 3: DAD1 C, Sig=230,4 Ref=360,100

Peak #	RetTime [min]	Type	Width [min]	Area [mAU*s]	Height [mAU]	Area %
1	14.642	BV R	0.3926	1.26219e4	441.36261	49.5520
2	19.192	VV R	0.4398	1.28501e4	352.37262	50.4480

Totals : 2.54720e4 793.73523

2,2,2-trichloroethyl (R)-(8-oxospiro[4.5]deca-6,9-dien-2-yl)carbamate (31):

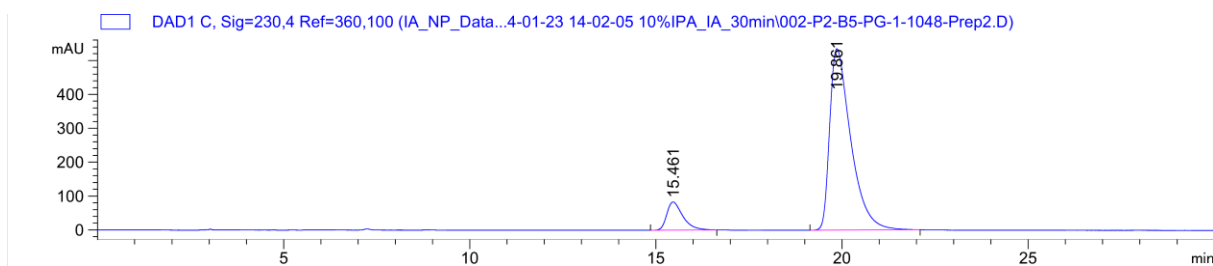

Signal 3: DAD1 C, Sig=230,4 Ref=360,100

Peak #	RetTime [min]	Type	Width [min]	Area [mAU*s]	Height [mAU]	Area %
1	15.461	BB	0.3499	2481.91553	83.11046	10.5321
2	19.861	BB	0.4951	2.10833e4	535.13440	89.4679

Totals : 2.35652e4 618.24486

2,2,2-trichloroethyl ((1R,3R)-2'-oxo-2'H-spiro[cyclopentane-1,1'-naphthalen]-3-yl)carbamate(**32**):

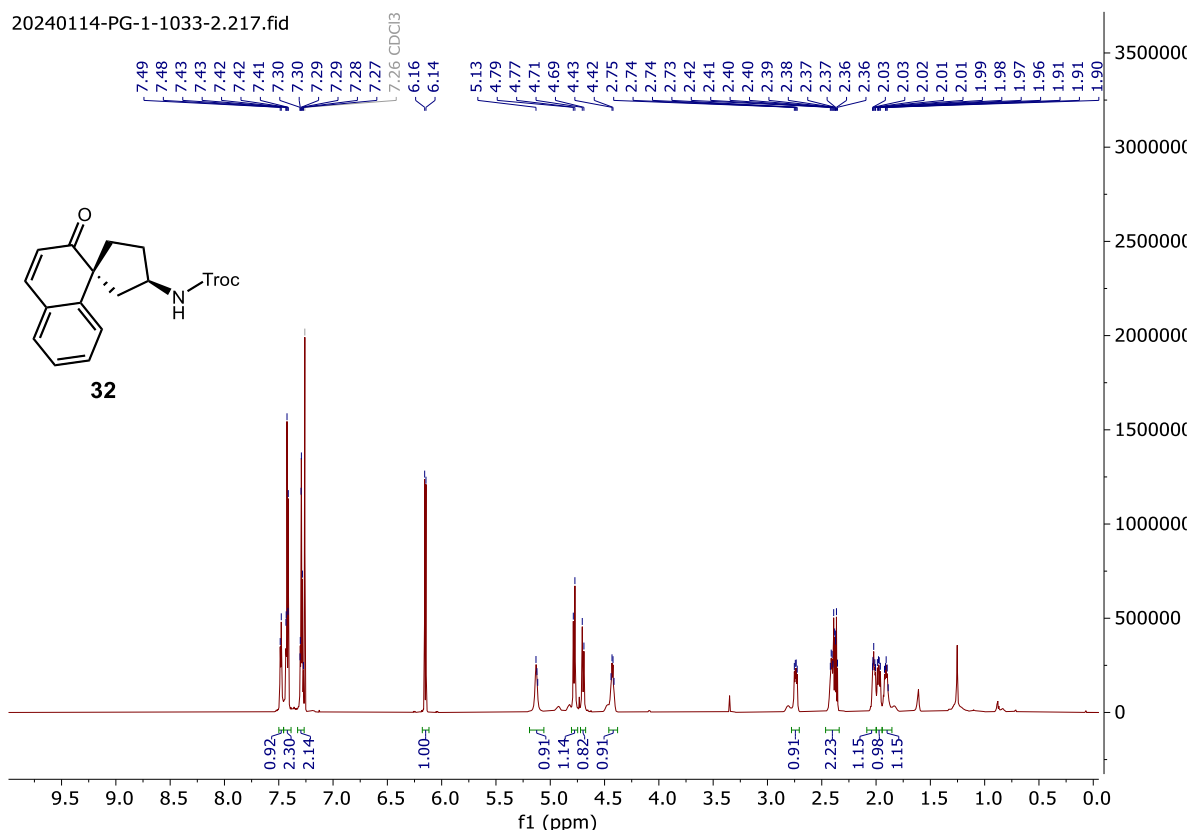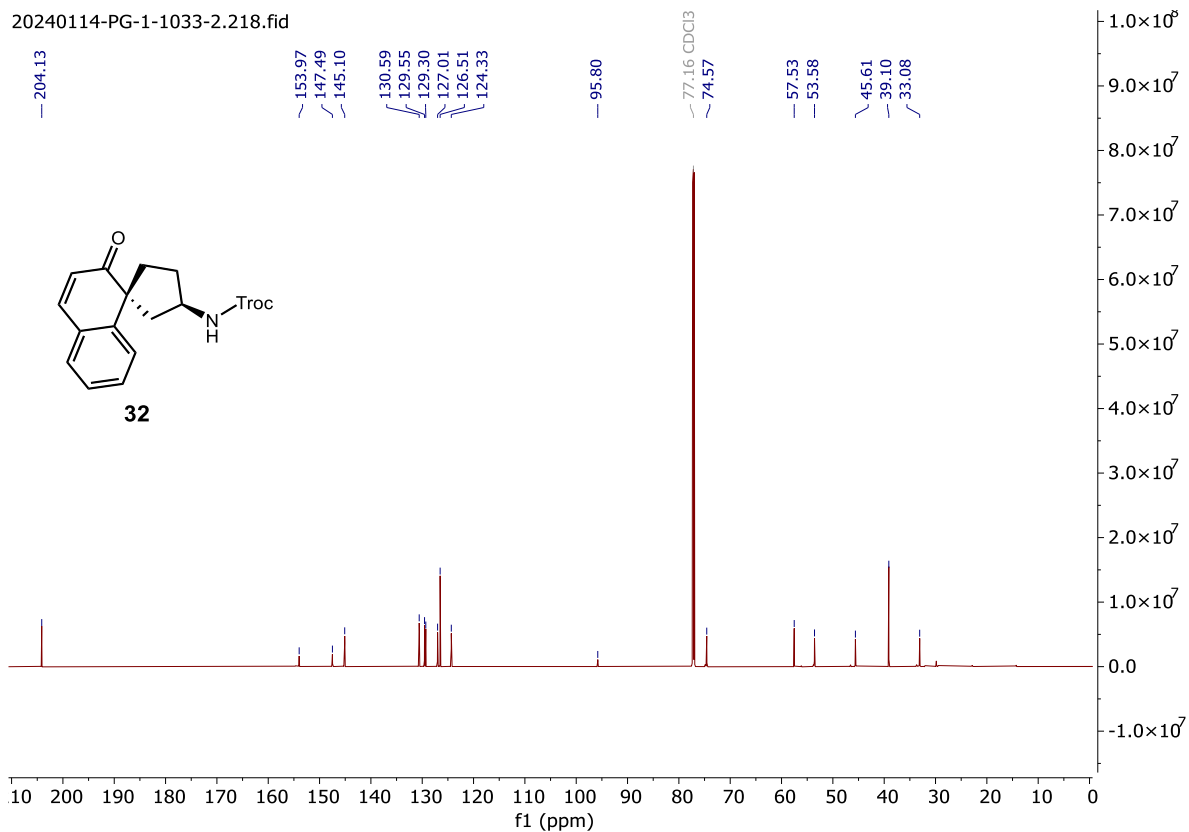

(32):

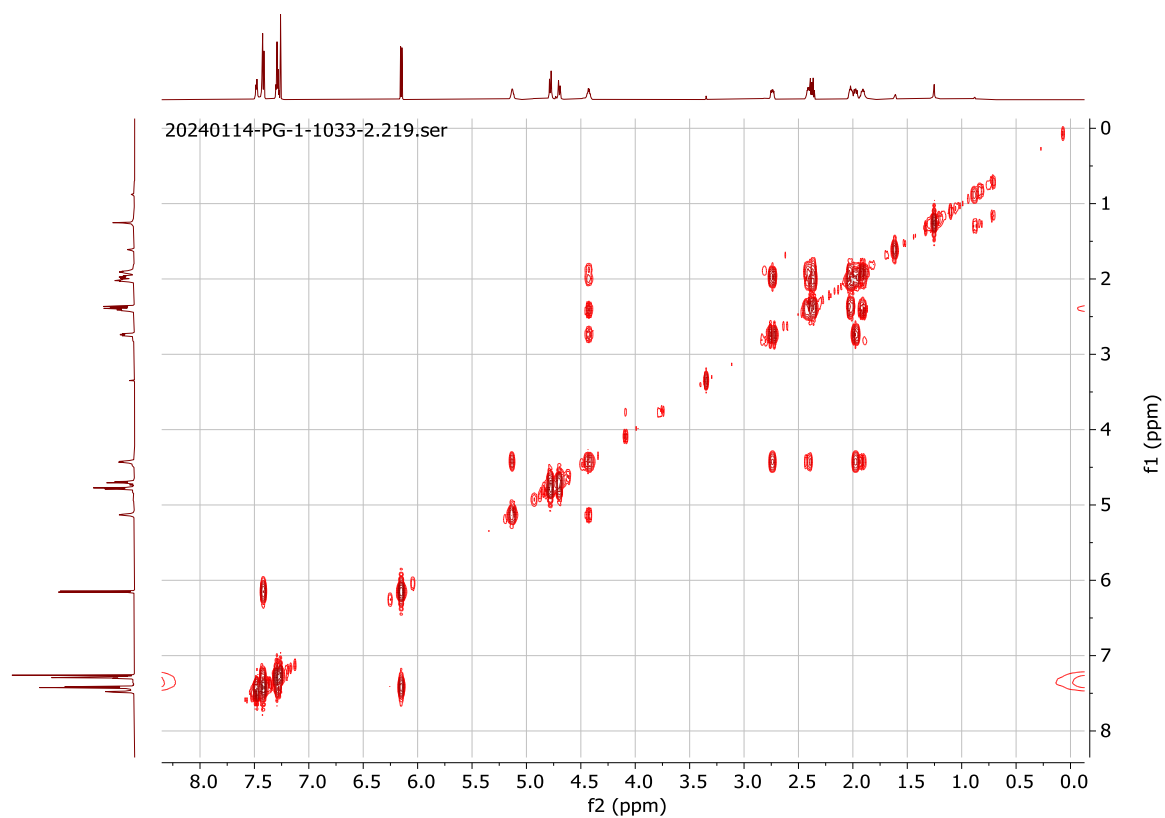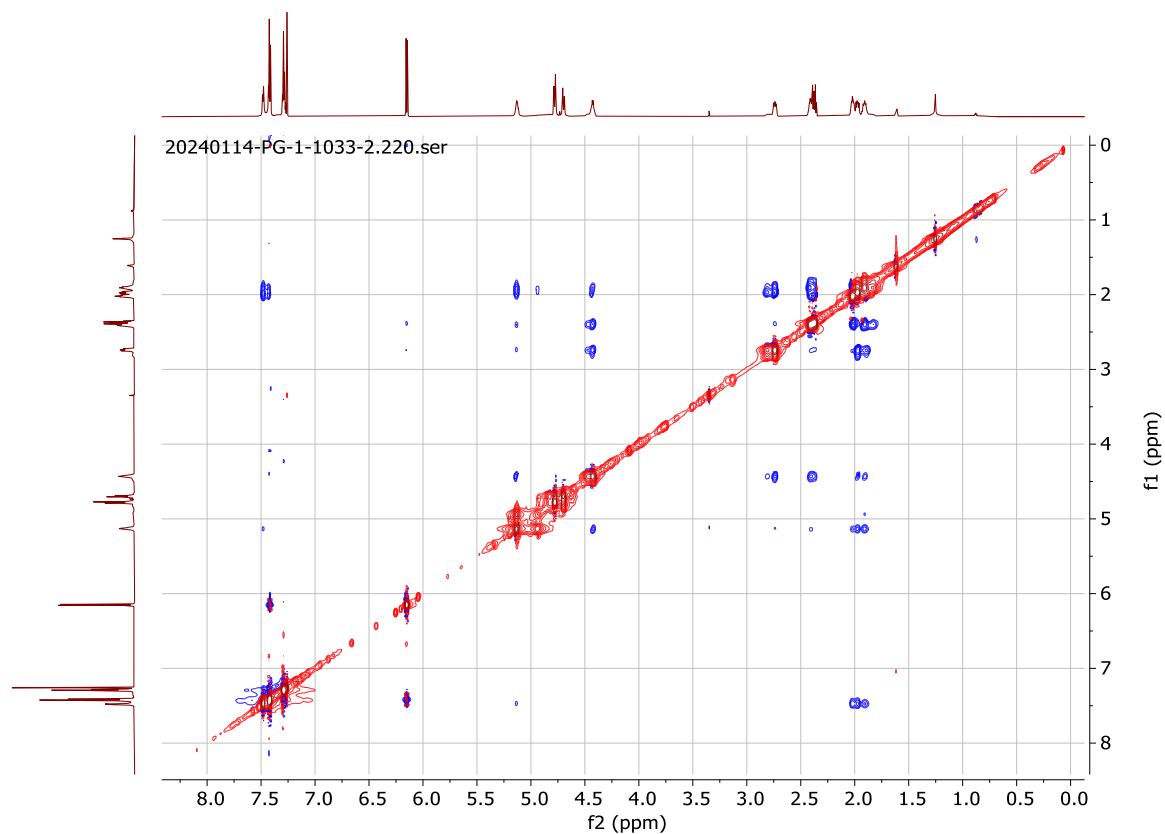

2,2,2-trichloroethyl ((1R,3R)-2'-oxo-2'H-spiro[cyclopentane-1,1'-naphthalen]-3-yl)carbamate (±32):

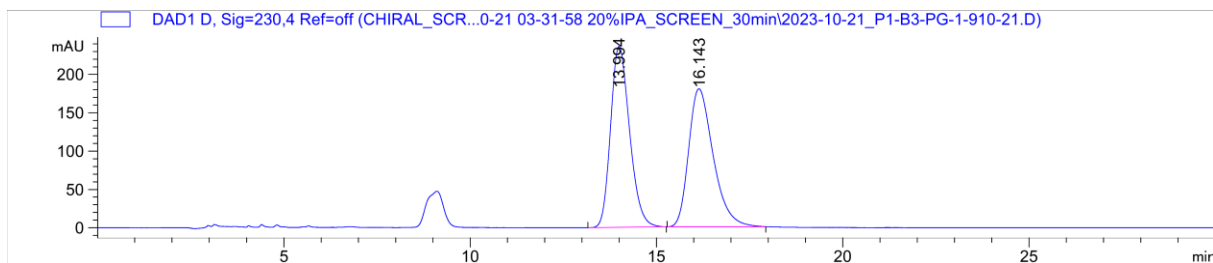

Signal 4: DAD1 D, Sig=230,4 Ref=off

Peak #	RetTime [min]	Type	Width [min]	Area [mAU*s]	Height [mAU]	Area %
1	13.994	BB	0.4373	8360.22461	236.01596	50.3765
2	16.143	BB	0.5380	8235.26074	179.92947	49.6235

Totals : 1.65955e4 415.94543

2,2,2-trichloroethyl ((1R,3R)-2'-oxo-2'H-spiro[cyclopentane-1,1'-naphthalen]-3-yl)carbamate (32):

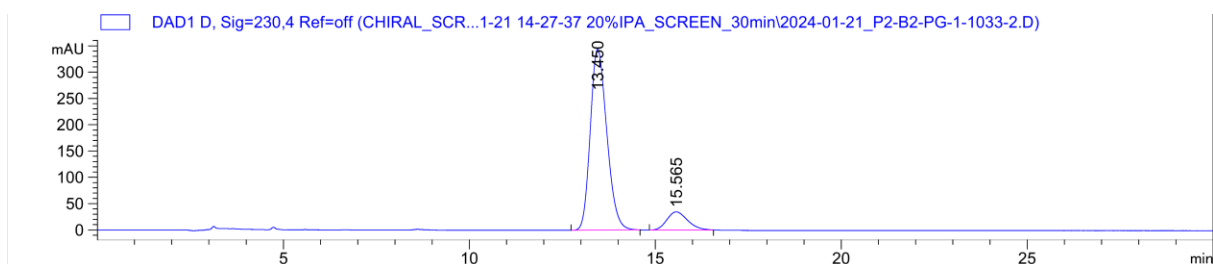

Signal 4: DAD1 D, Sig=230,4 Ref=off

Peak #	RetTime [min]	Type	Width [min]	Area [mAU*s]	Height [mAU]	Area %
1	13.450	BB	0.4065	1.06456e4	344.05215	88.8895
2	15.565	VV R	0.4525	1330.62378	34.45852	11.1105

Totals : 1.19762e4 378.51067

2,2,2-trichloroethyl ((1S,3R)-2'-oxo-2'H-spiro[cyclopentane-1,1'-naphthalen]-3-yl)carbamate (**32'**):

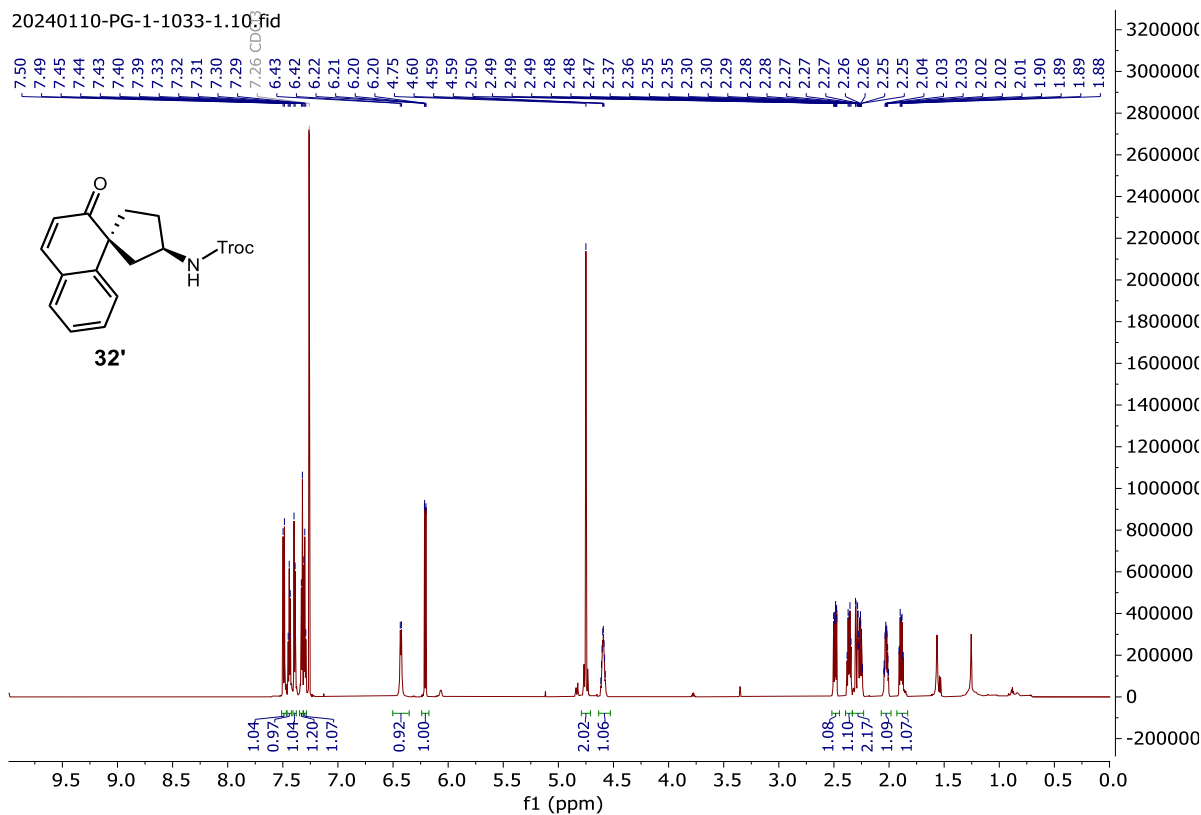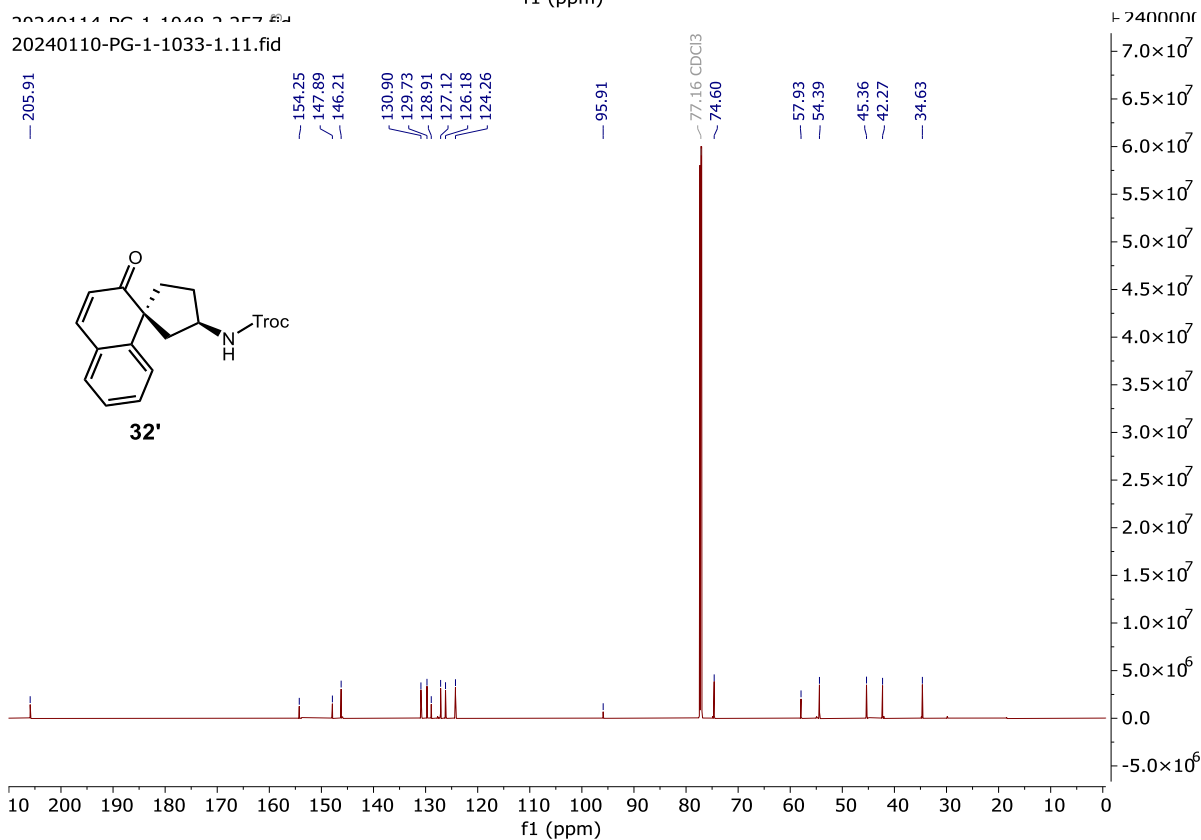

(32'):

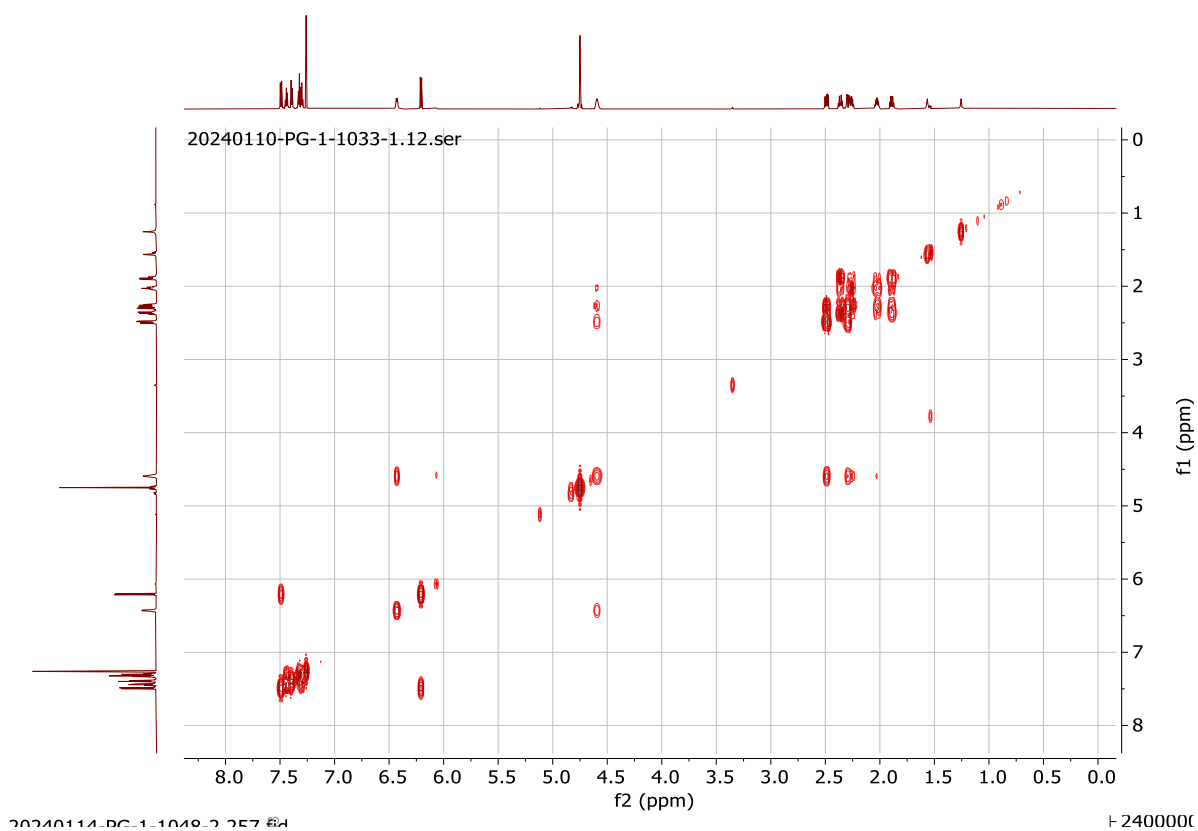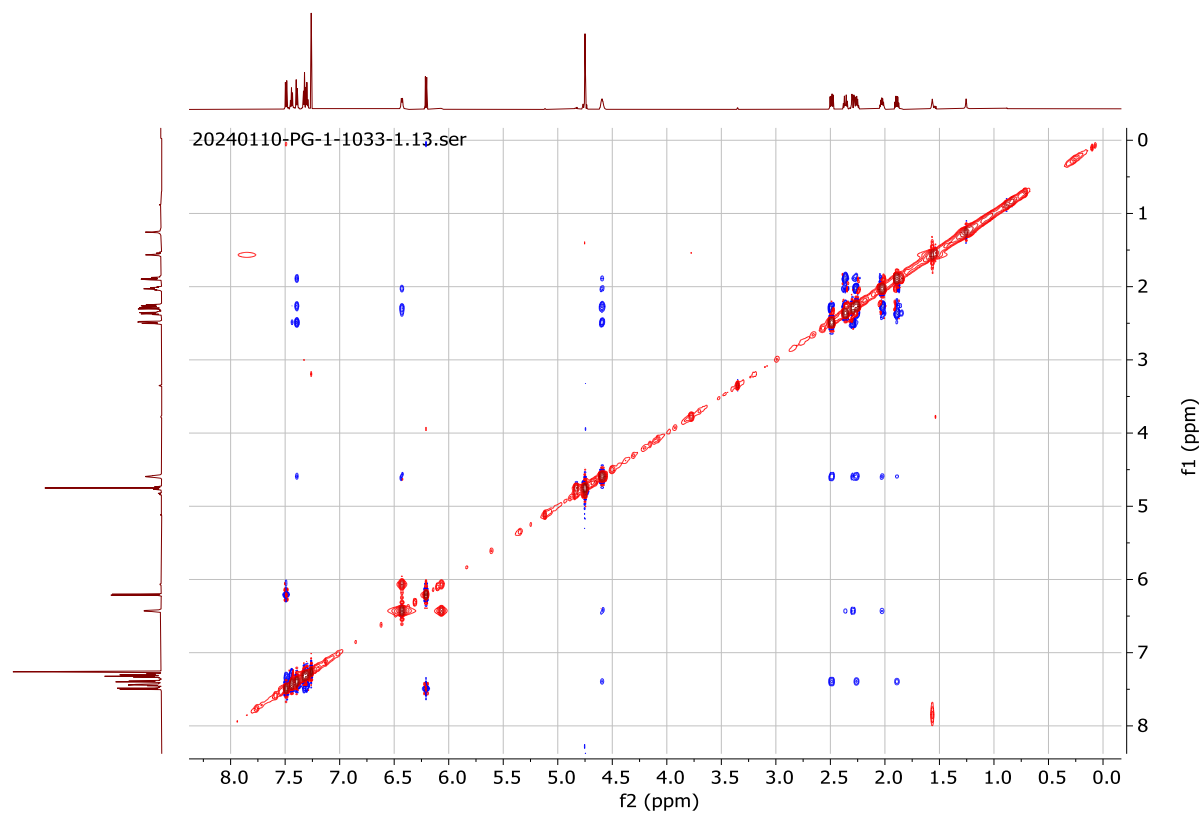

2,2,2-trichloroethyl ((1*S*,3*R*)-2'-oxo-2'-*H*-spiro[cyclopentane-1,1'-naphthalen]-3-yl)carbamate (±-32'):

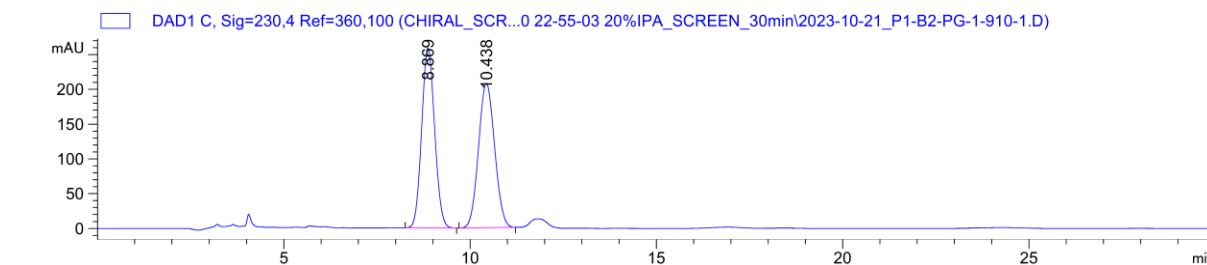

Signal 3: DAD1 C, Sig=230,4 Ref=360,100

Peak #	RetTime [min]	Type	Width [min]	Area [mAU*s]	Height [mAU]	Area %
1	8.869	BB	0.3284	6275.72217	258.78503	50.1898
2	10.438	BB	0.3588	6228.25781	207.75491	49.8102

Totals : 1.25040e4 466.53995

2,2,2-trichloroethyl ((1*S*,3*R*)-2'-oxo-2'-*H*-spiro[cyclopentane-1,1'-naphthalen]-3-yl)carbamate (32'):

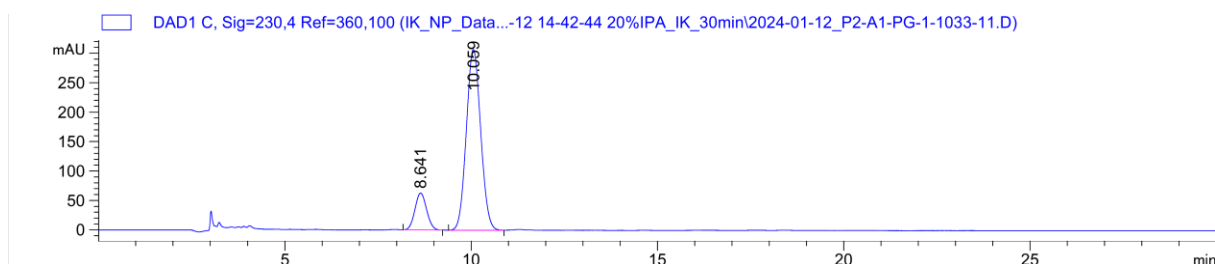

Signal 3: DAD1 C, Sig=230,4 Ref=360,100

Peak #	RetTime [min]	Type	Width [min]	Area [mAU*s]	Height [mAU]	Area %
1	8.641	BB	0.2550	1359.03430	62.55727	14.1970
2	10.059	BB	0.3244	8213.65332	306.76489	85.8030

Totals : 9572.68762 369.32216

2,2,2-trichloroethyl (R)-(9-oxospiro[5.5]undeca-7,10-dien-2-yl)carbamate (**33**):

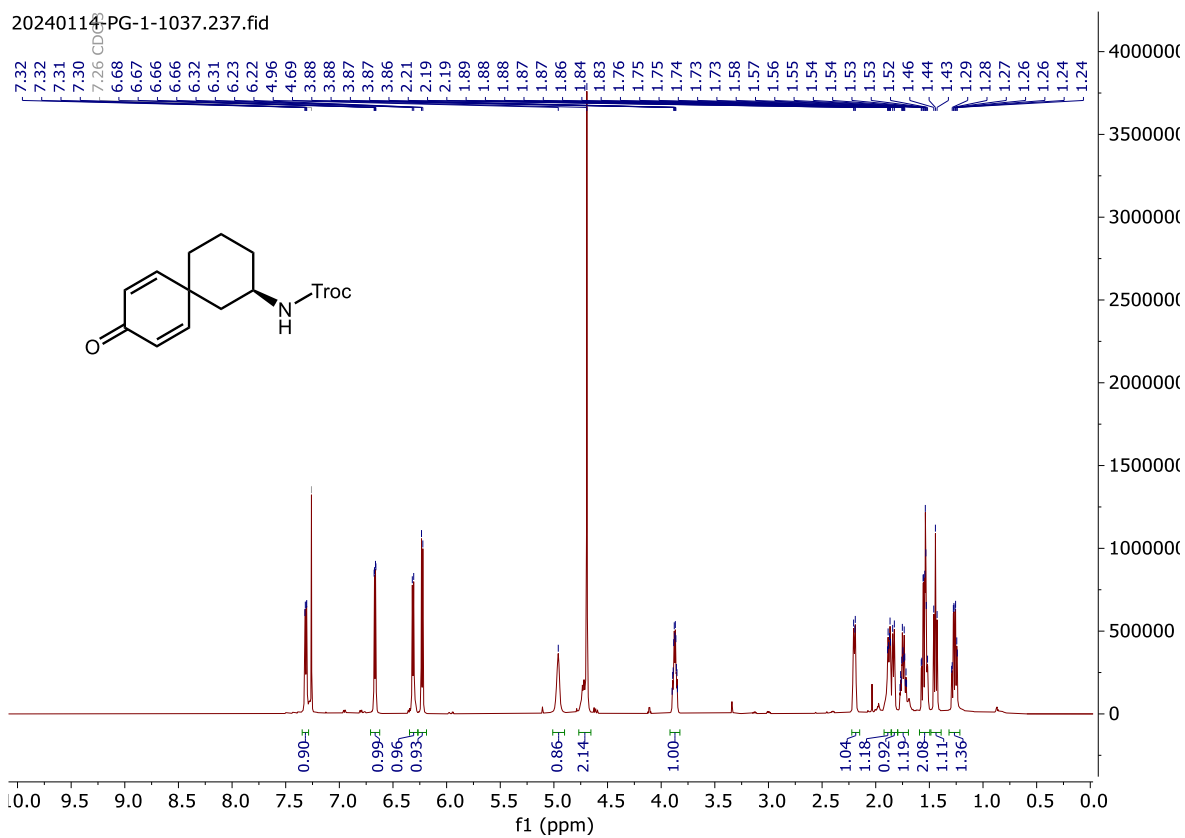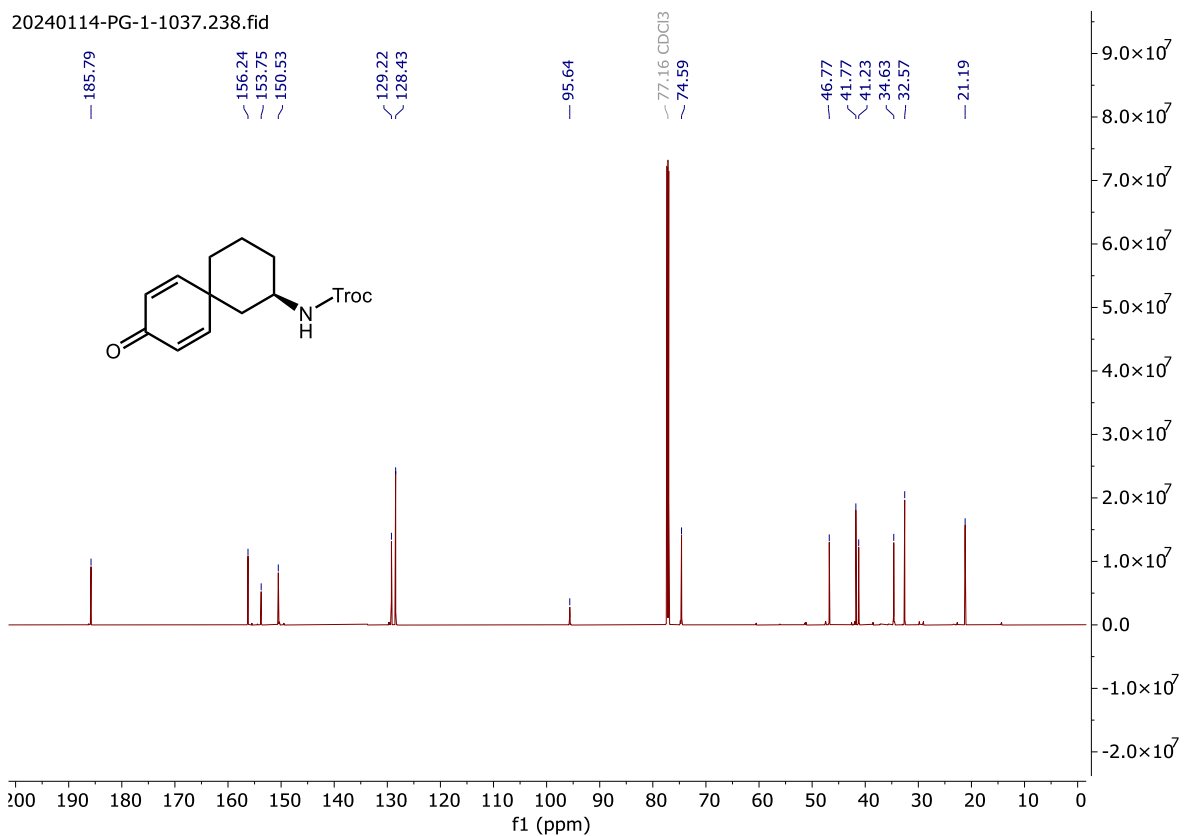

2,2,2-trichloroethyl (9-oxospiro[5.5]undeca-7,10-dien-2-yl)carbamate ($\pm$ -33):

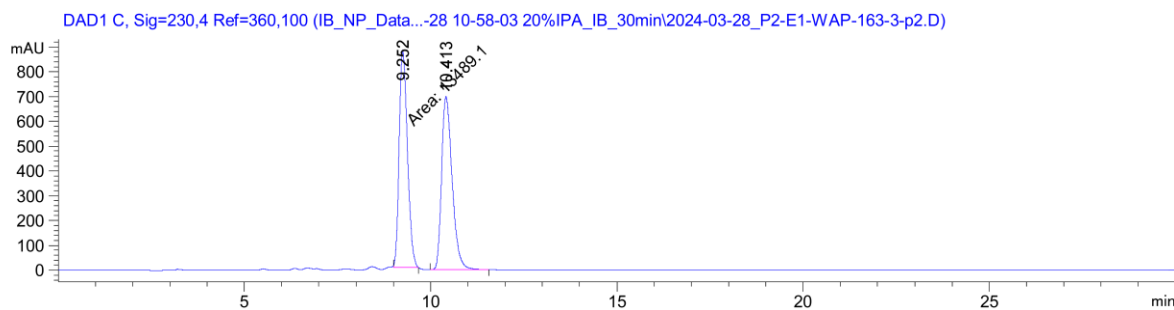

Signal 3: DAD1 C, Sig=230,4 Ref=360,100

Peak #	RetTime [min]	Type	Width [min]	Area [mAU*s]	Height [mAU]	Area %
1	9.252	MM	0.2565	1.34891e4	876.44525	49.5070
2	10.413	BV R	0.2945	1.37577e4	699.34204	50.4930

Totals : 2.72468e4 1575.78729

2,2,2-trichloroethyl (R)-(9-oxospiro[5.5]undeca-7,10-dien-2-yl)carbamate (33):

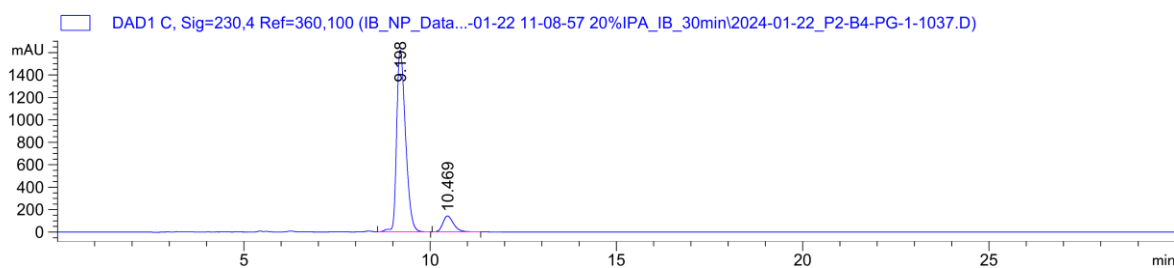

Signal 3: DAD1 C, Sig=230,4 Ref=360,100

Peak #	RetTime [min]	Type	Width [min]	Area [mAU*s]	Height [mAU]	Area %
1	9.198	VB R	0.2308	2.68146e4	1620.85364	90.4624
2	10.469	BB	0.2716	2827.10815	142.10719	9.5376

Totals : 2.96417e4 1762.96083

2,2,2-trichloroethyl ((1*S*,3*R*)-2'-oxo-2'-*H*-spiro[cyclohexane-1,1'-naphthalen]-3-yl)carbamate (**34**) and 2,2,2-trichloroethyl ((1*R*,3*R*)-2'-oxo-2'-*H*-spiro[cyclohexane-1,1'-naphthalen]-3-yl)carbamate (**34'**):

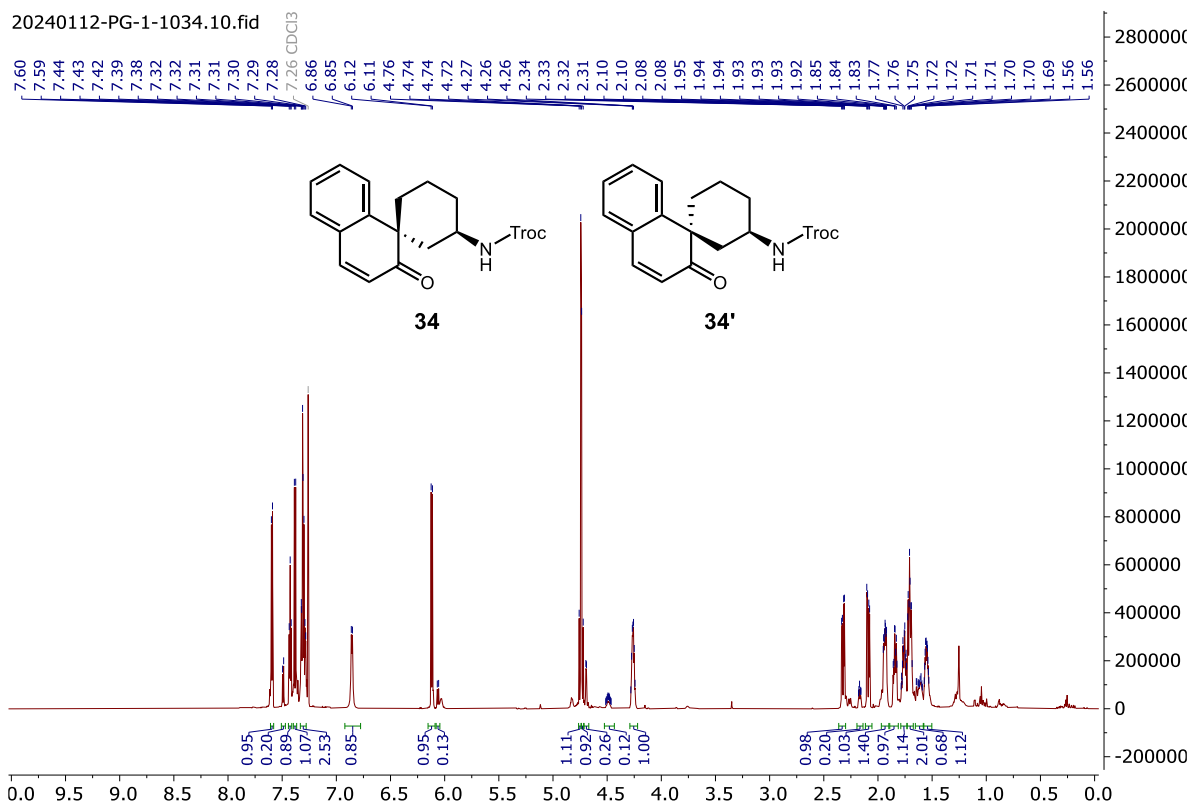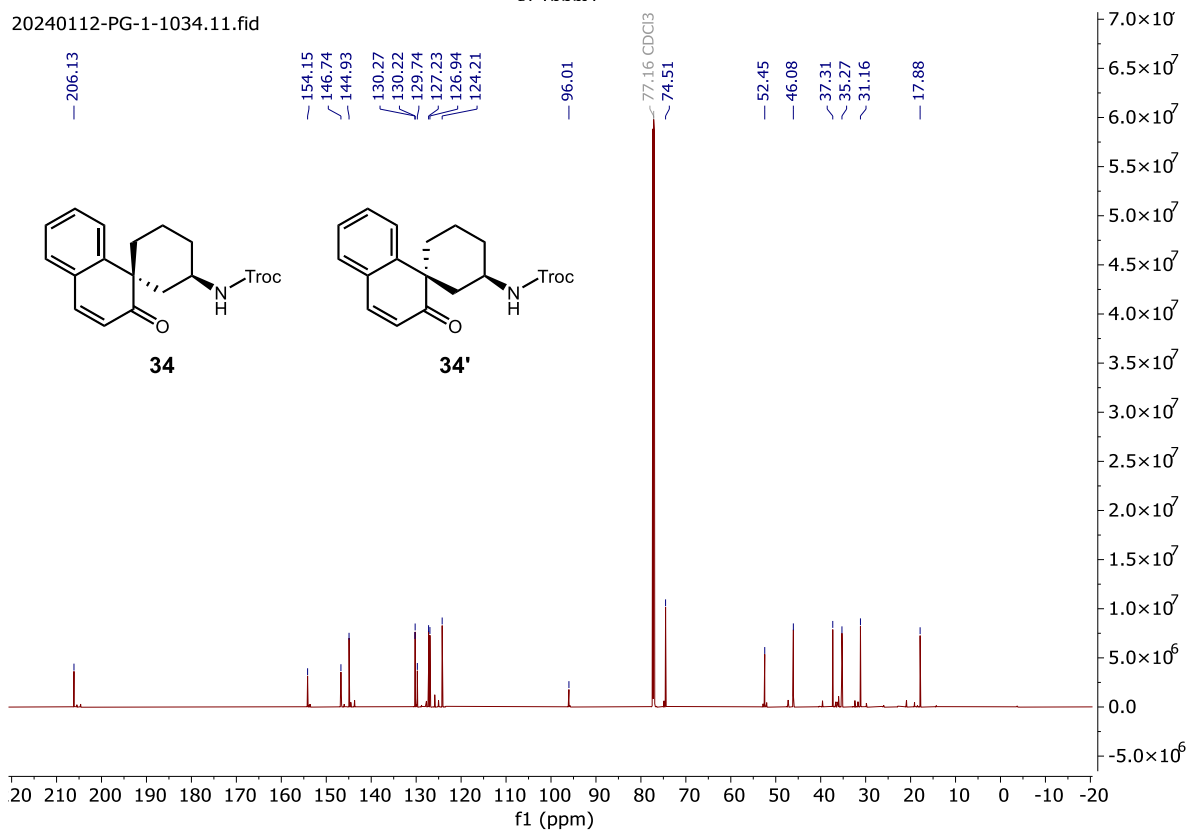

(34) and (34'):

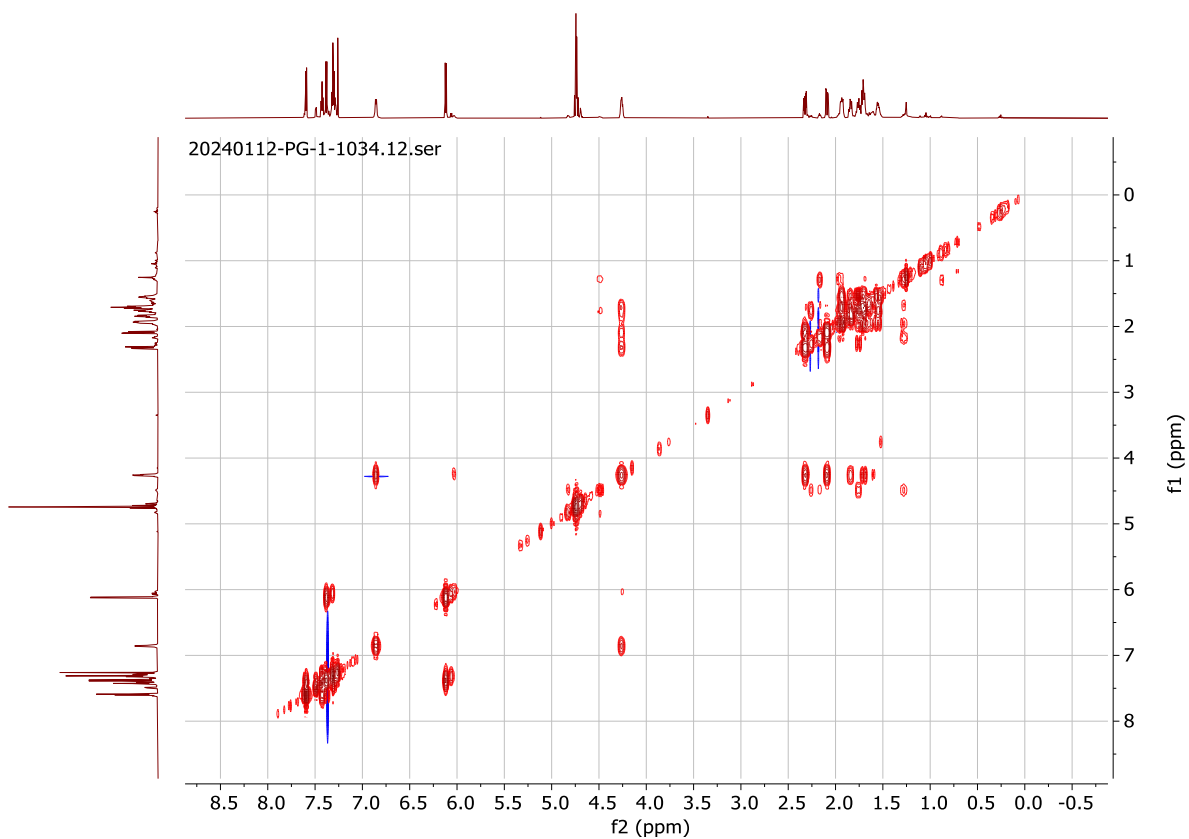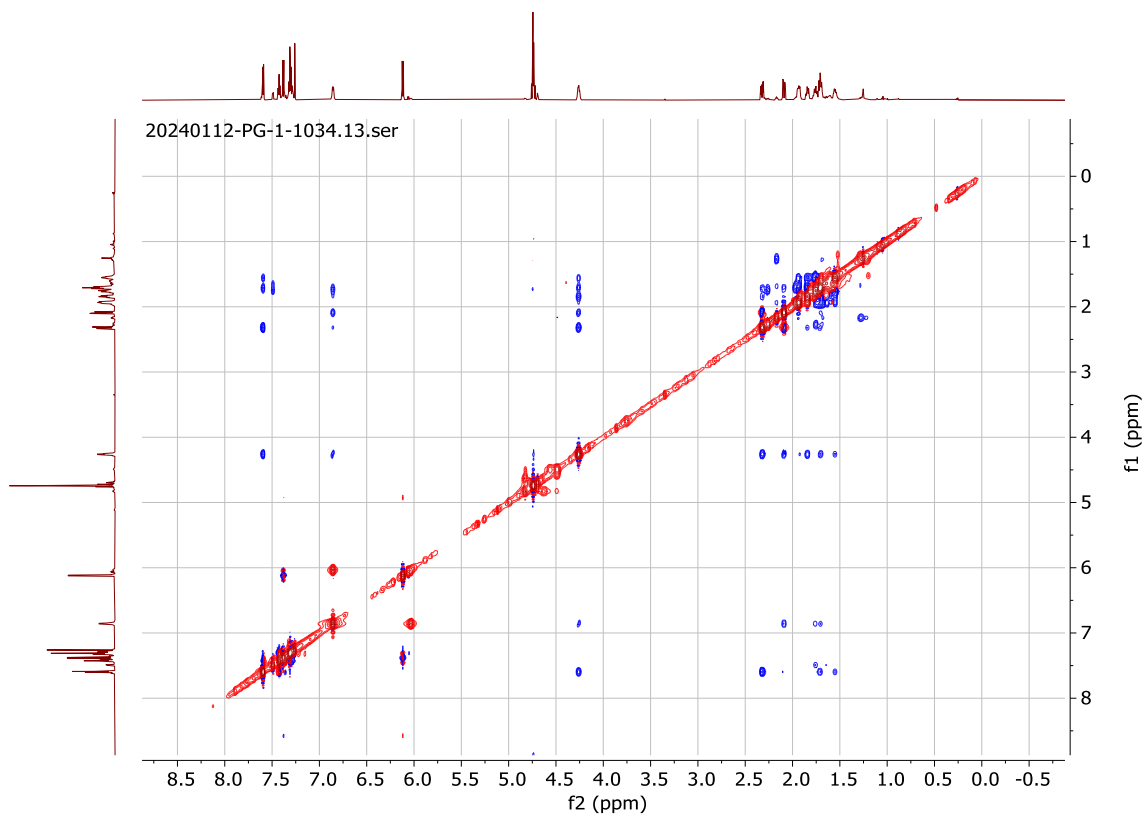

2,2,2-trichloroethyl ((1*S*,3*R*)-2'-oxo-2'*H*-spiro[cyclohexane-1,1'-naphthalen]-3-yl)carbamate ($\pm$ -34**):**

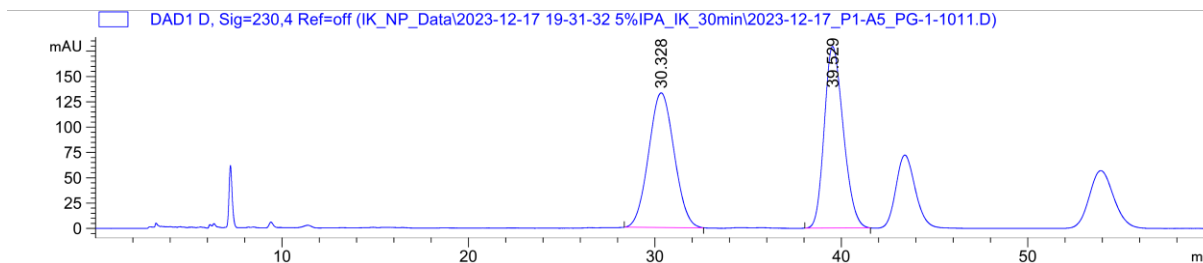

Signal 4: DAD1 D, Sig=230,4 Ref=off

Peak #	RetTime [min]	Type	Width [min]	Area [mAU*s]	Height [mAU]	Area %
1	30.328	BB	1.1113	1.26442e4	132.91623	49.7966
2	39.529	BB	0.8334	1.27475e4	179.31085	50.2034

Totals : 2.53917e4 312.22708

2,2,2-trichloroethyl ((1*S*,3*R*)-2'-oxo-2'*H*-spiro[cyclohexane-1,1'-naphthalen]-3-yl)carbamate (34**):**

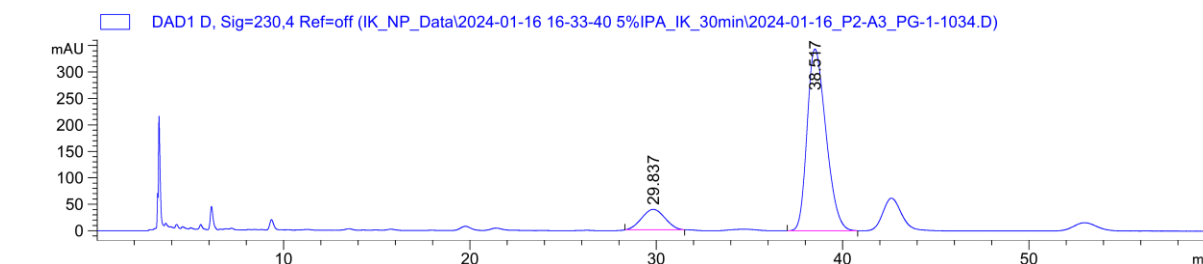

Signal 4: DAD1 D, Sig=230,4 Ref=off

Peak #	RetTime [min]	Type	Width [min]	Area [mAU*s]	Height [mAU]	Area %
1	29.837	BB	1.0125	3344.14697	38.59244	12.1706
2	38.517	VV R	0.8216	2.41332e4	343.51312	87.8294

Totals : 2.74774e4 382.10556

2,2,2-trichloroethyl ((1R,3R)-2'-oxo-2'H-spiro[cyclohexane-1,1'-naphthalen]-3-yl)carbamate (±-34'):

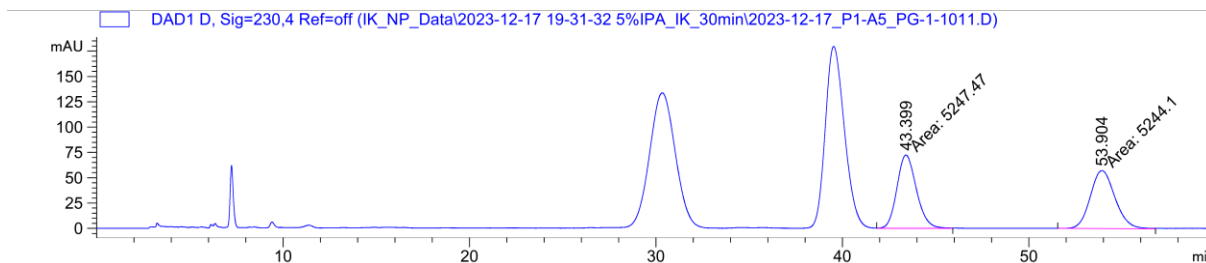

Signal 4: DAD1 D, Sig=230,4 Ref=off

Peak #	RetTime [min]	Type	Width [min]	Area [mAU*s]	Height [mAU]	Area %
1	43.399	MM	1.2078	5247.46826	72.41349	50.0161
2	53.904	MM	1.5274	5244.09717	57.22234	49.9839

Totals : 1.04916e4 129.63583

2,2,2-trichloroethyl ((1R,3R)-2'-oxo-2'H-spiro[cyclohexane-1,1'-naphthalen]-3-yl)carbamate (34'):

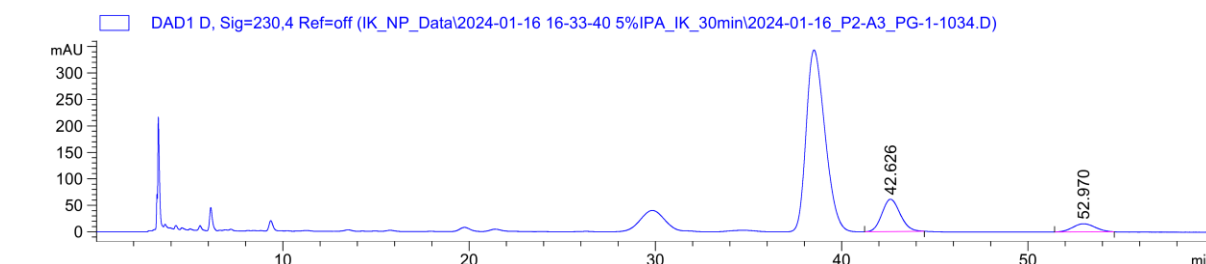

Signal 4: DAD1 D, Sig=230,4 Ref=off

Peak #	RetTime [min]	Type	Width [min]	Area [mAU*s]	Height [mAU]	Area %
1	42.626	BV R	0.7925	4138.69336	61.04314	76.2606
2	52.970	BB	0.9757	1288.34912	15.42955	23.7394

Totals : 5427.04248 76.47270

2,2,2-trichloroethyl ((2R,6R)-8-methoxy-9-oxospiro[5.5]undeca-7,10-dien-2-yl)carbamate (35):

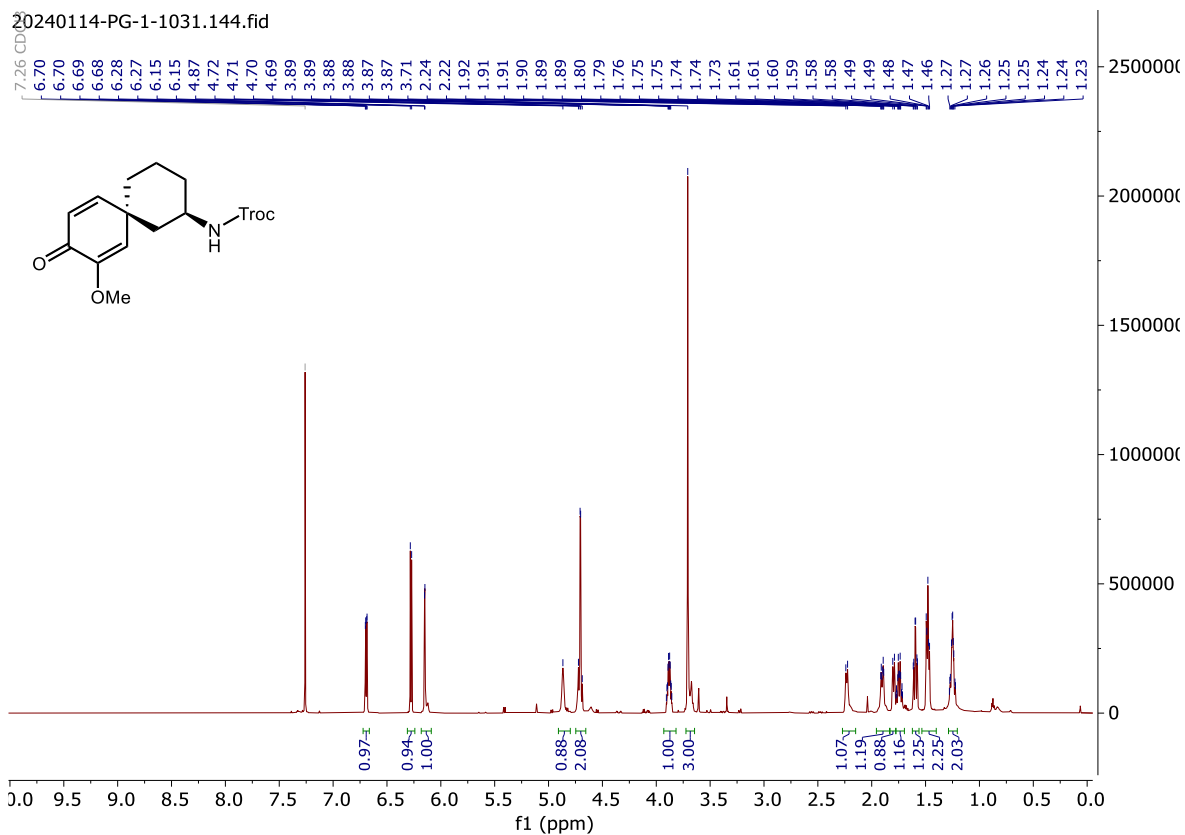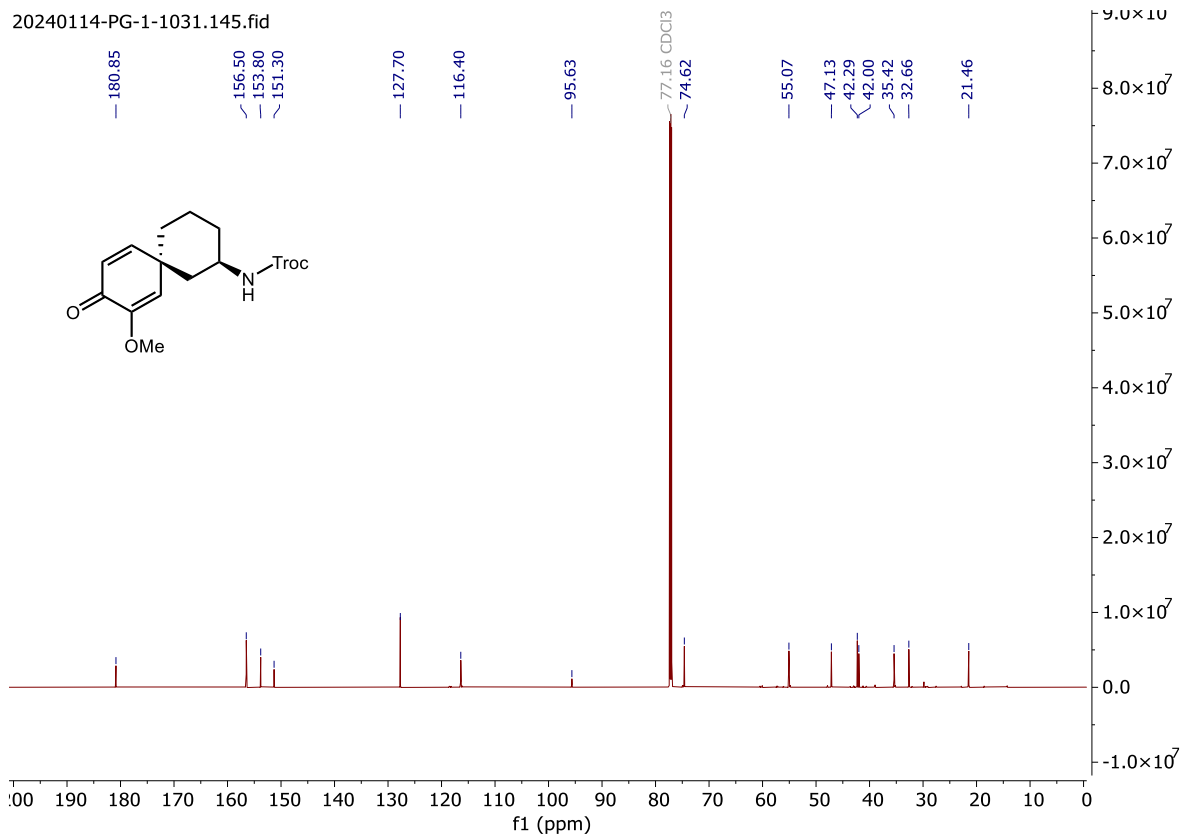

(35)

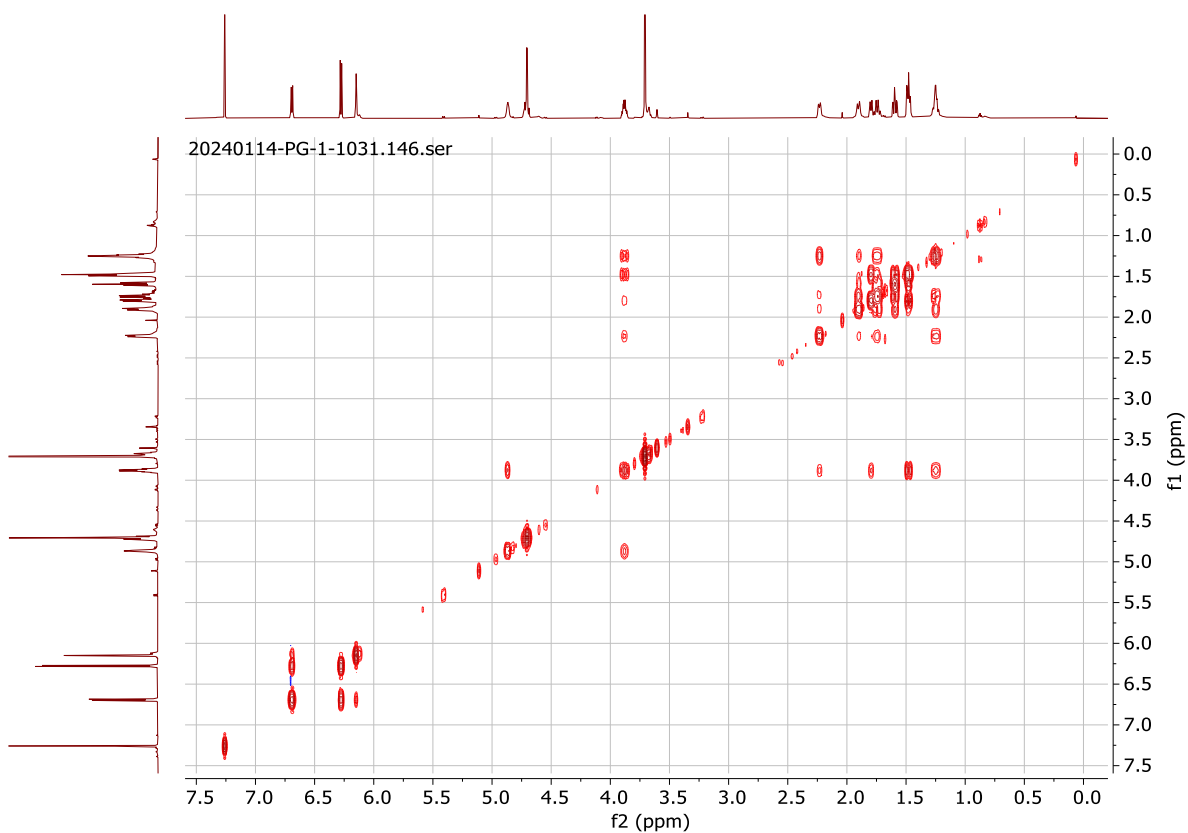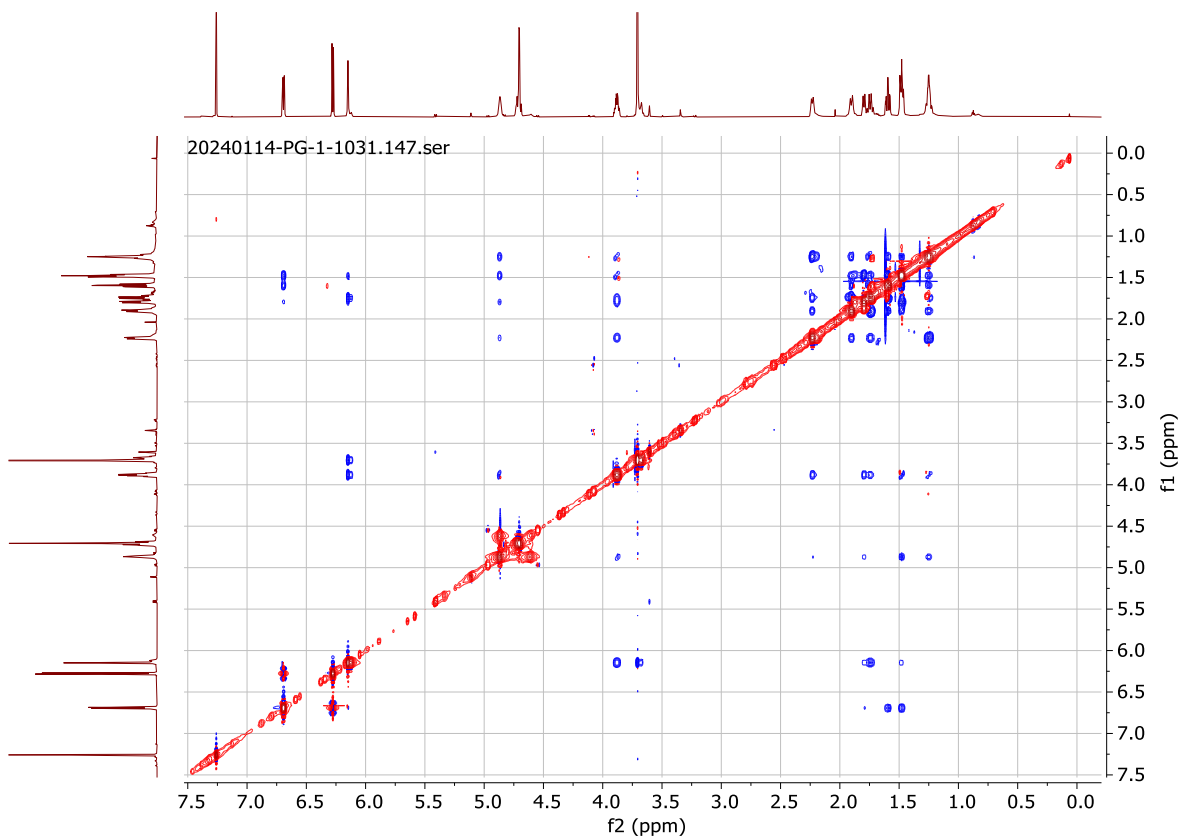

2,2,2-trichloroethyl ((2R,6R)-8-methoxy-9-oxospiro[5.5]undeca-7,10-dien-2-yl)carbamate ($\pm$ -35):

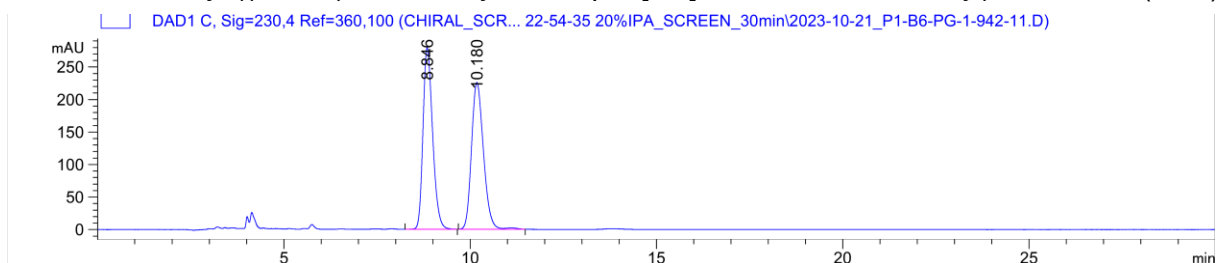

Signal 3: DAD1 C, Sig=230,4 Ref=360,100

Peak #	RetTime [min]	Type	Width [min]	Area [mAU*s]	Height [mAU]	Area %
1	8.846	BB	0.2710	5008.08105	278.82919	49.8599
2	10.180	BV R	0.3215	5036.21826	225.72603	50.1401

Totals : 1.00443e4 504.55522

2,2,2-trichloroethyl ((2R,6R)-8-methoxy-9-oxospiro[5.5]undeca-7,10-dien-2-yl)carbamate (35):

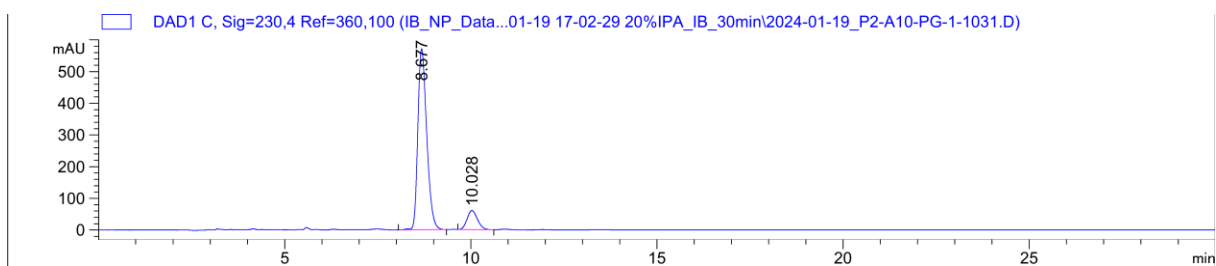

Signal 3: DAD1 C, Sig=230,4 Ref=360,100

Peak #	RetTime [min]	Type	Width [min]	Area [mAU*s]	Height [mAU]	Area %
1	8.677	VB R	0.2491	9455.07324	571.04541	89.0630
2	10.028	BV R	0.2463	1161.08948	59.98688	10.9370

Totals : 1.06162e4 631.03229

2,2,2-trichloroethyl ((2*R*,6*R*)-7-methoxy-9-oxospiro[5.5]undeca-7,10-dien-2-yl)carbamate (**36**) and 2,2,2-trichloroethyl ((2*R*,6*S*)-7-methoxy-9-oxospiro[5.5]undeca-7,10-dien-2-yl)carbamate (**36'**):

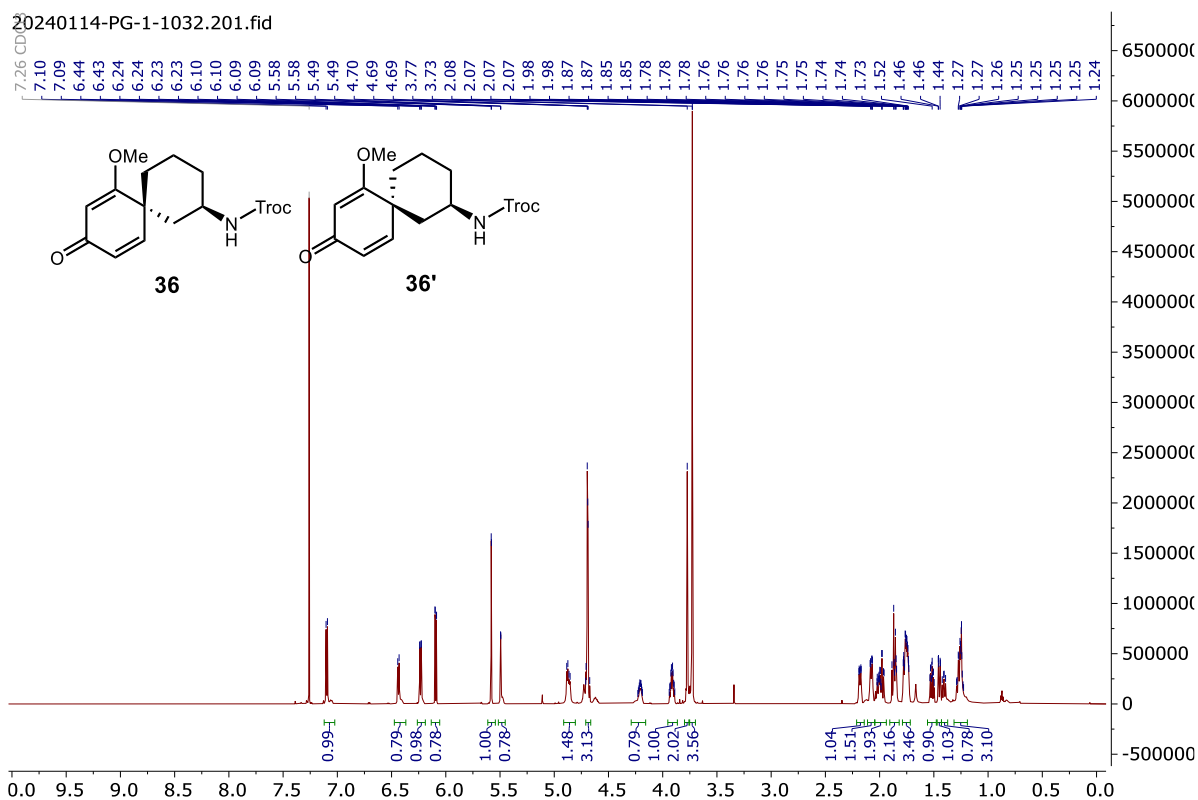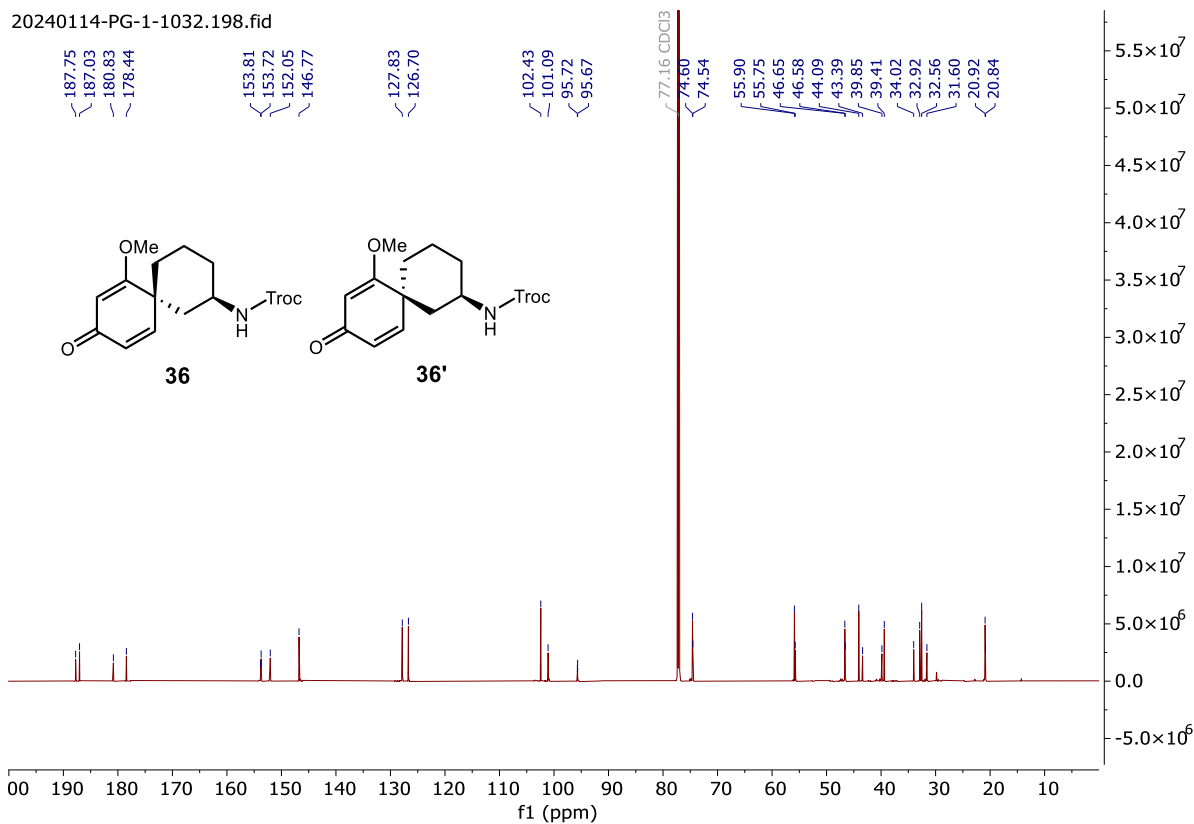

(36) and (36'):

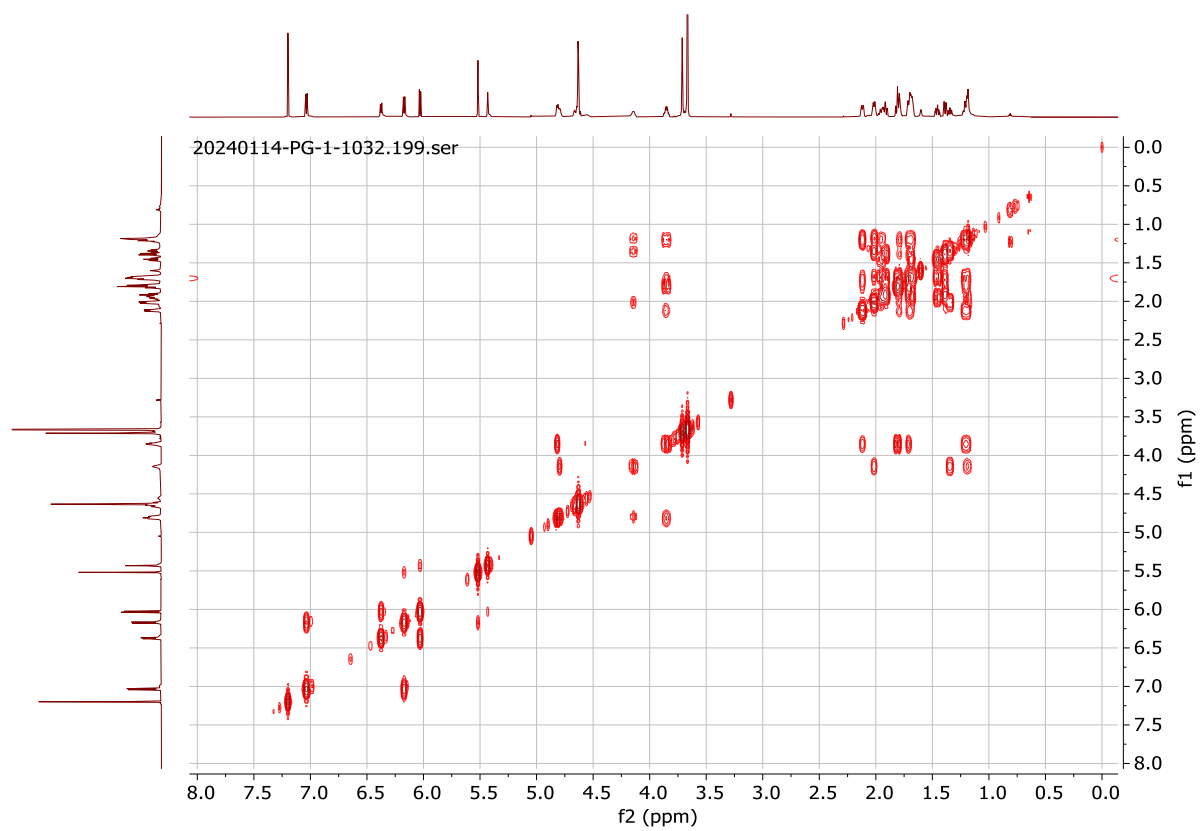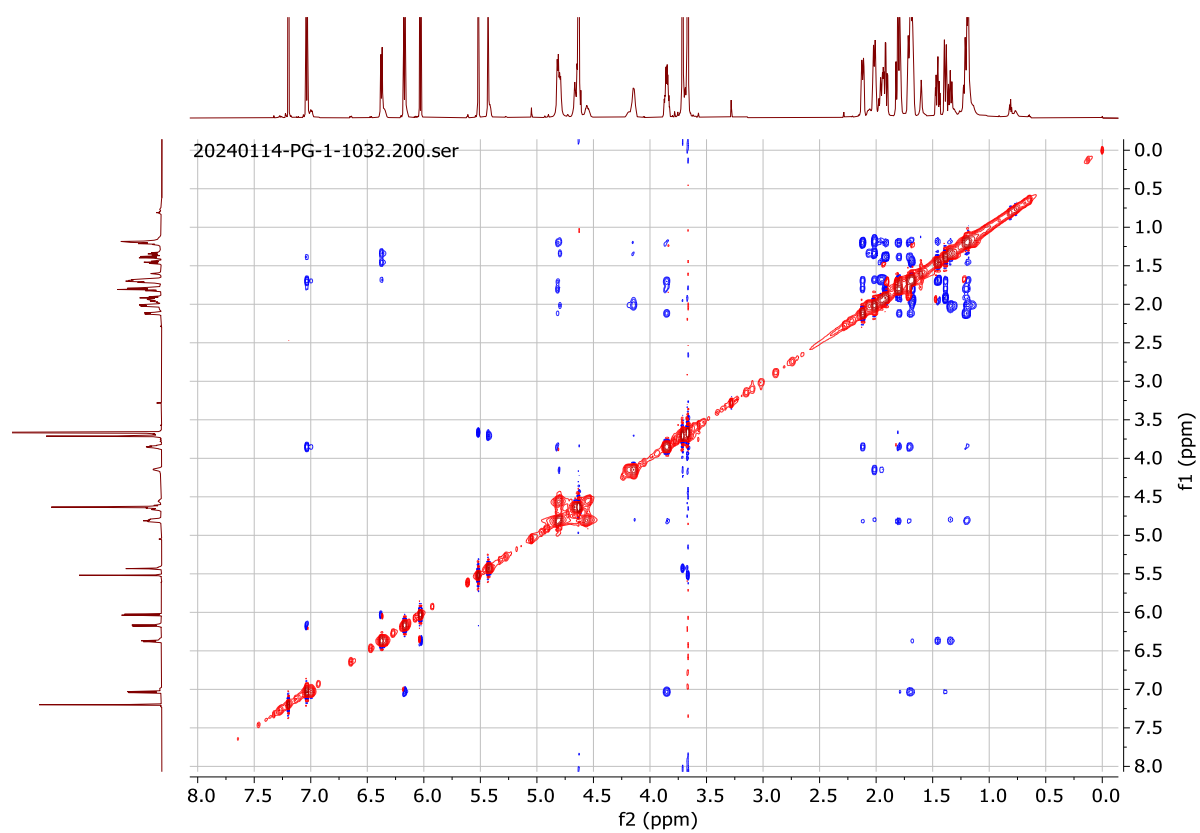

2,2,2-trichloroethyl ((2R,6R)-7-methoxy-9-oxospiro[5.5]undeca-7,10-dien-2-yl)carbamate ($\pm$ -36):

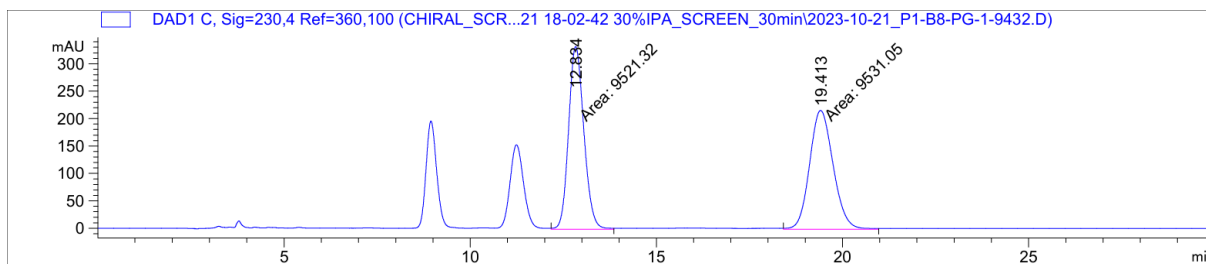

Signal 3: DAD1 C, Sig=230,4 Ref=360,100

Peak #	RetTime [min]	Type	Width [min]	Area [mAU*s]	Height [mAU]	Area %
1	12.834	MM	0.4763	9521.31836	333.19983	49.9745
2	19.413	MM	0.7328	9531.04688	216.77274	50.0255

Totals : 1.90524e4 549.97256

2,2,2-trichloroethyl ((2R,6R)-7-methoxy-9-oxospiro[5.5]undeca-7,10-dien-2-yl)carbamate (36):

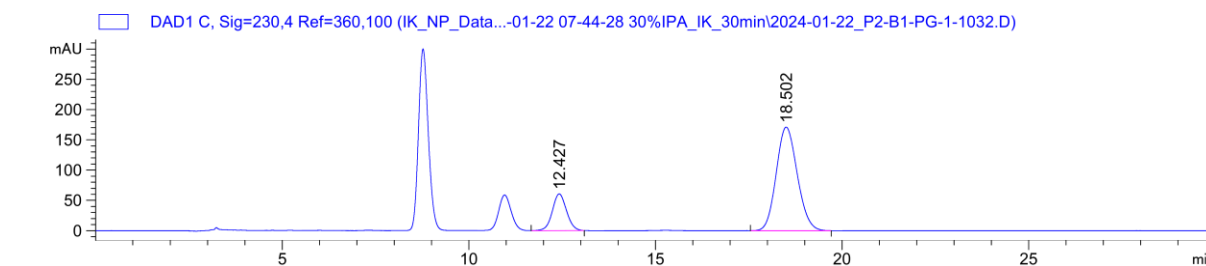

Signal 3: DAD1 C, Sig=230,4 Ref=360,100

Peak #	RetTime [min]	Type	Width [min]	Area [mAU*s]	Height [mAU]	Area %
1	12.427	BV R	0.3101	1592.13757	60.58779	19.2001
2	18.502	VB R	0.4610	6700.19141	171.05046	80.7999

Totals : 8292.32898 231.63825

2,2,2-trichloroethyl ((2R,6S)-7-methoxy-9-oxospiro[5.5]undeca-7,10-dien-2-yl)carbamate ($\pm$ -36'):

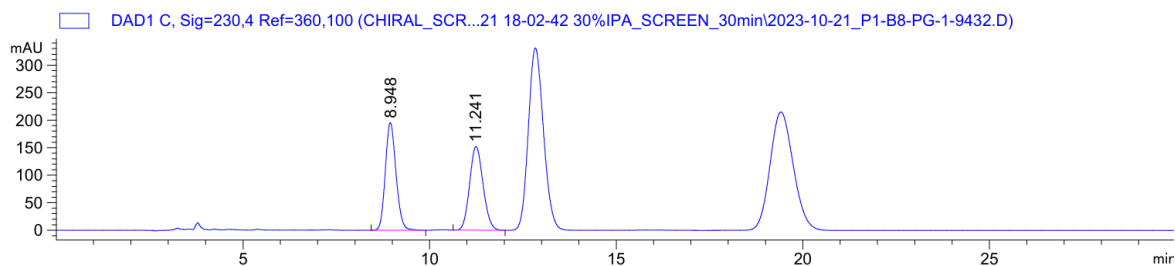

Signal 3: DAD1 C, Sig=230,4 Ref=360,100

Peak #	RetTime [min]	Type	Width [min]	Area [mAU*s]	Height [mAU]	Area %
1	8.948	BB	0.2813	3882.78125	195.42876	50.4193
2	11.241	BB	0.3405	3818.19800	152.29341	49.5807

2,2,2-trichloroethyl ((2R,6S)-7-methoxy-9-oxospiro[5.5]undeca-7,10-dien-2-yl)carbamate (36'):

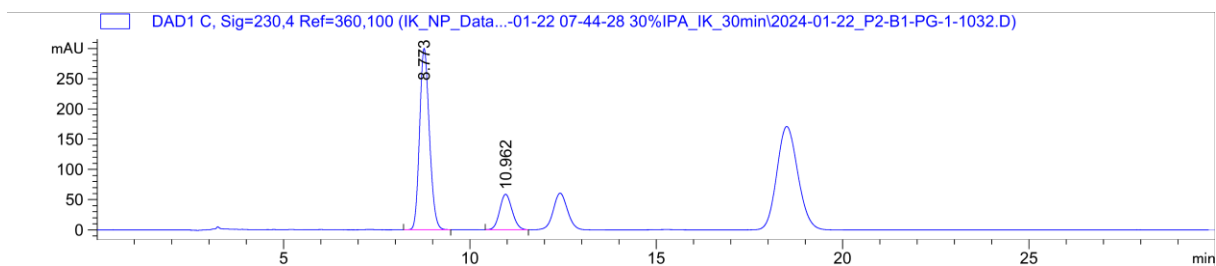

Signal 3: DAD1 C, Sig=230,4 Ref=360,100

Peak #	RetTime [min]	Type	Width [min]	Area [mAU*s]	Height [mAU]	Area %
1	8.773	BV R	0.2657	5483.44824	300.02490	80.1786
2	10.962	BV R	0.2733	1355.59644	58.72931	19.8214

Totals : 6839.04468 358.75422

2,2,2-trichloroethyl (2R,5aR,9aS)-8-oxo-2,3,4,5,9,9a-hexahydro-2,5a-methanobenzo[b]azepine-1(8H)-carboxylate ($\pm$ -39):

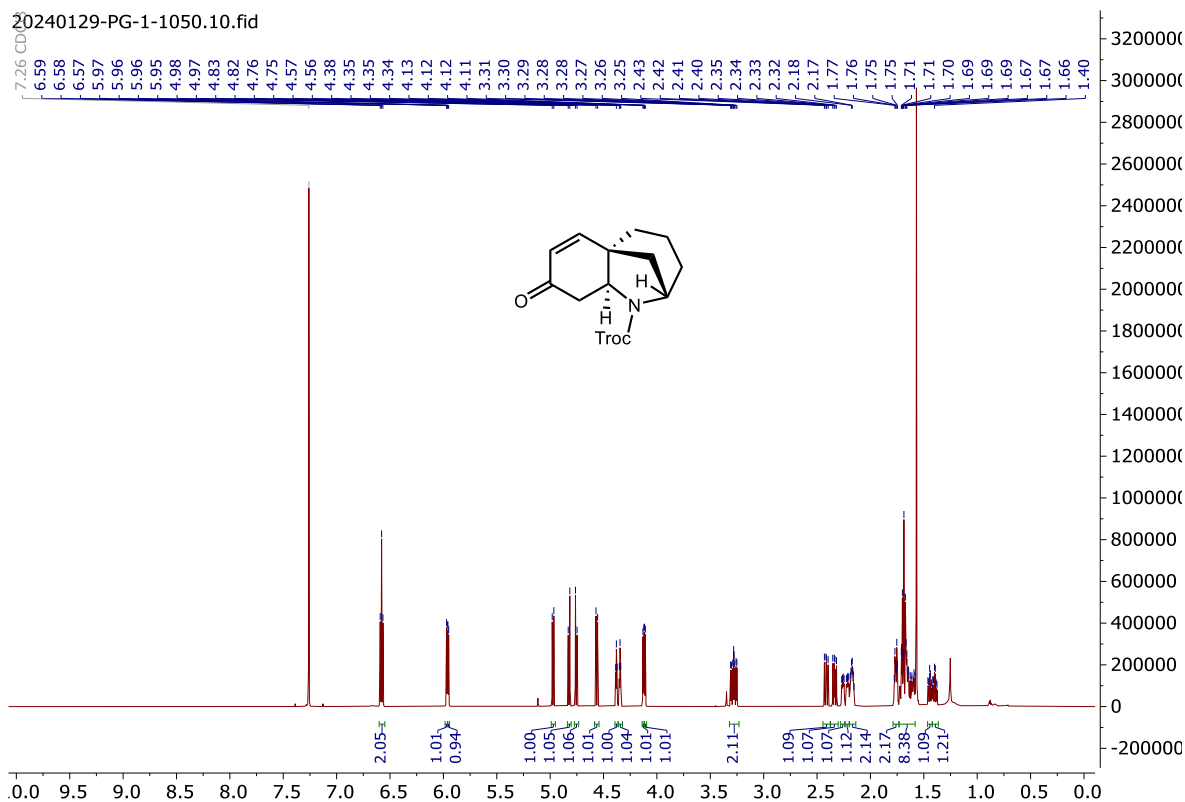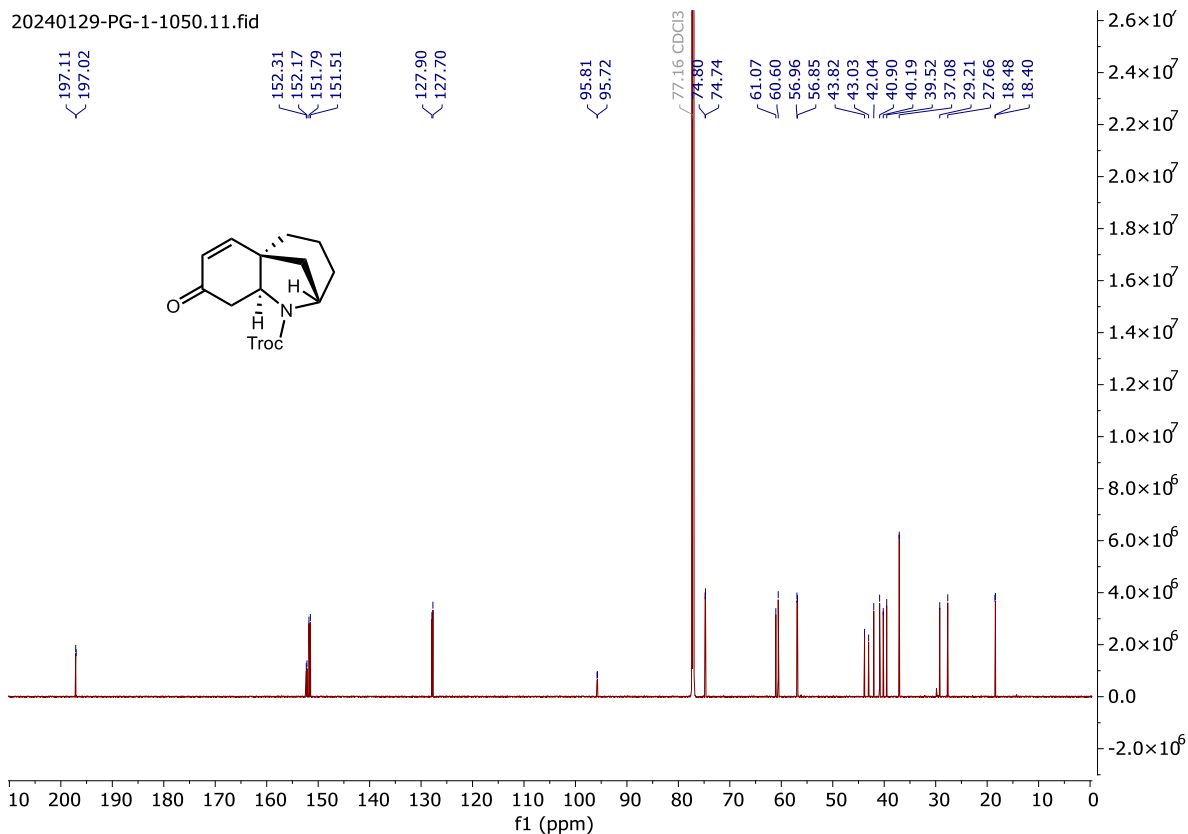

Key HMBC (^1H - ^{13}C) correlations used to assign the structure of ($\pm$ -**39**):

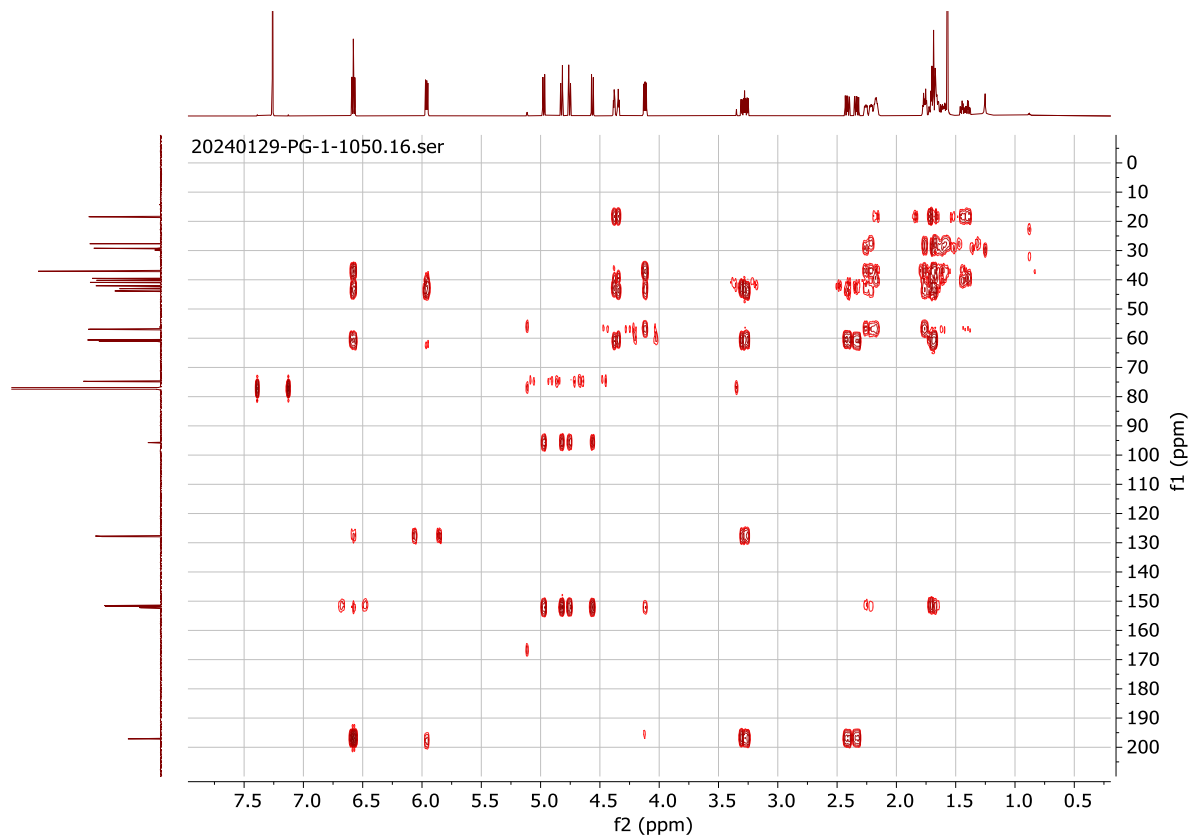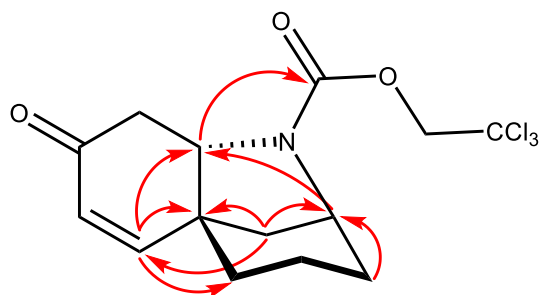

Key NOESY (^1H - ^1H) correlations used to assign the structure of **39**:

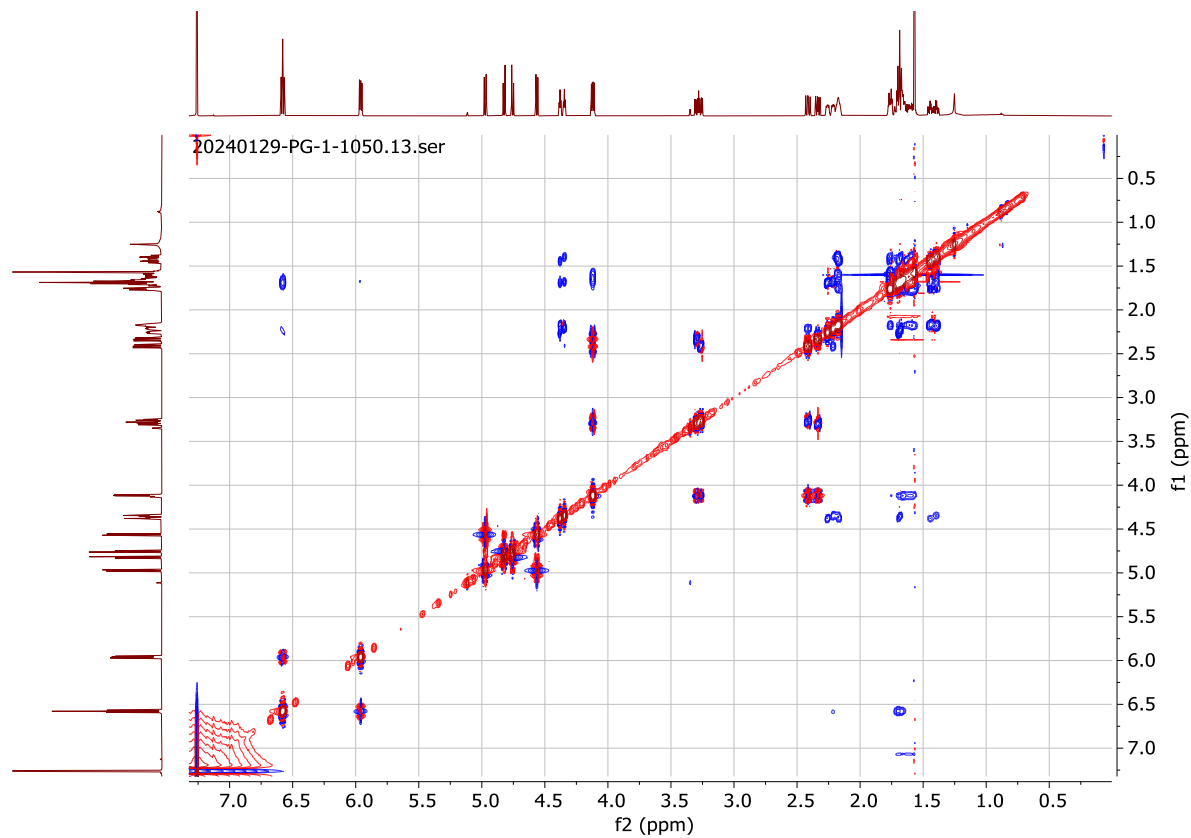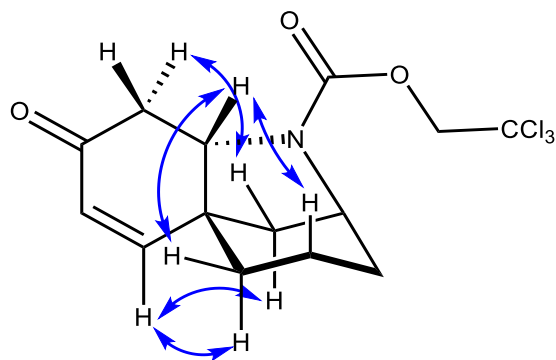

COSY and HSQC used to assign the structure of $\pm$ -39

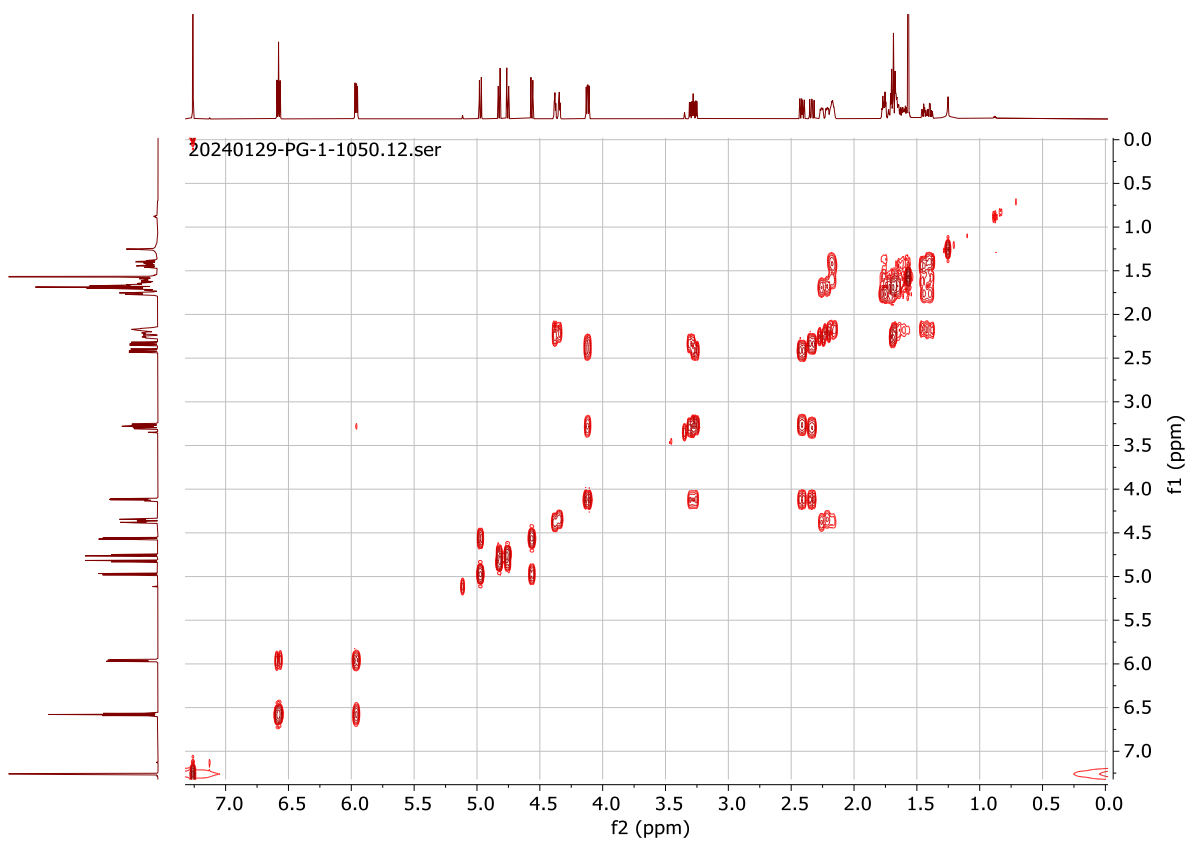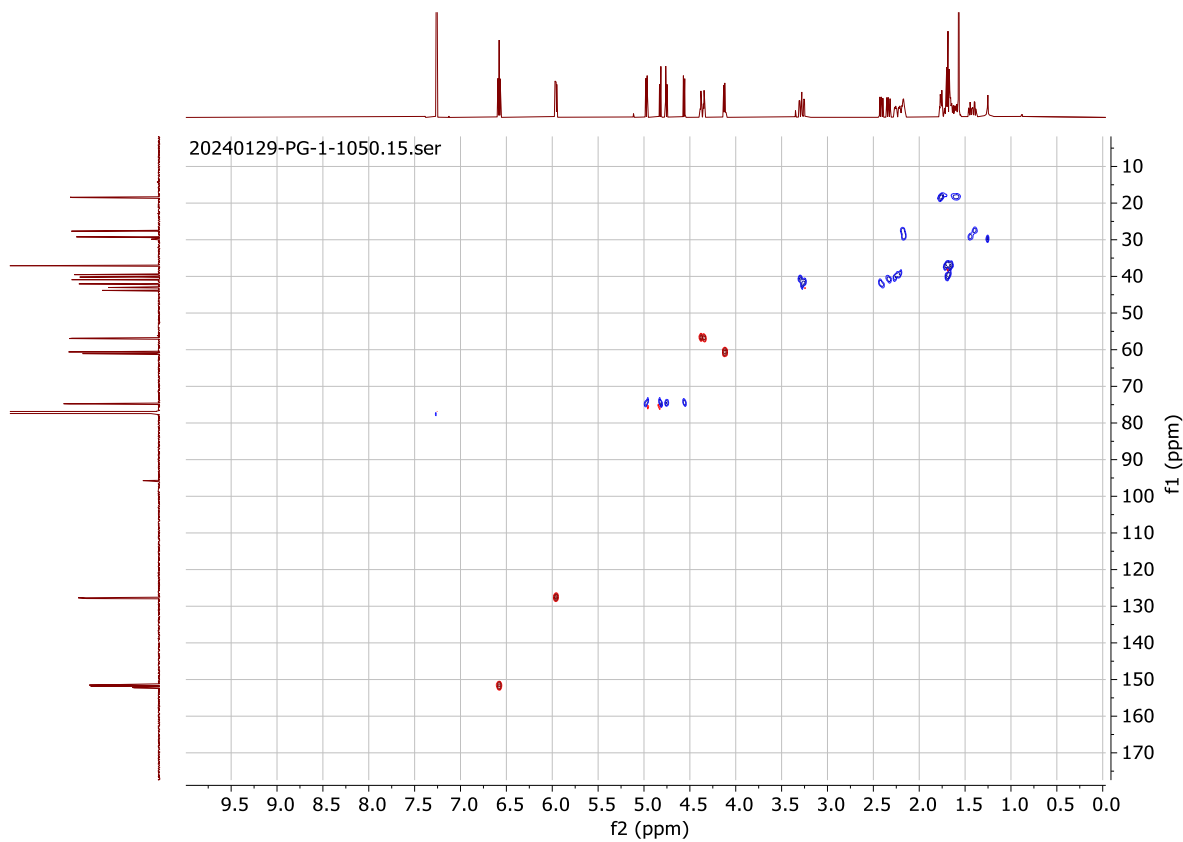

2,2,2-trichloroethyl (2R,5aR,9aS)-8-oxo-2,3,4,5,9,9a-hexahydro-2,5a-methanobenzo[b]azepine-1(8H)-carboxylate (39):

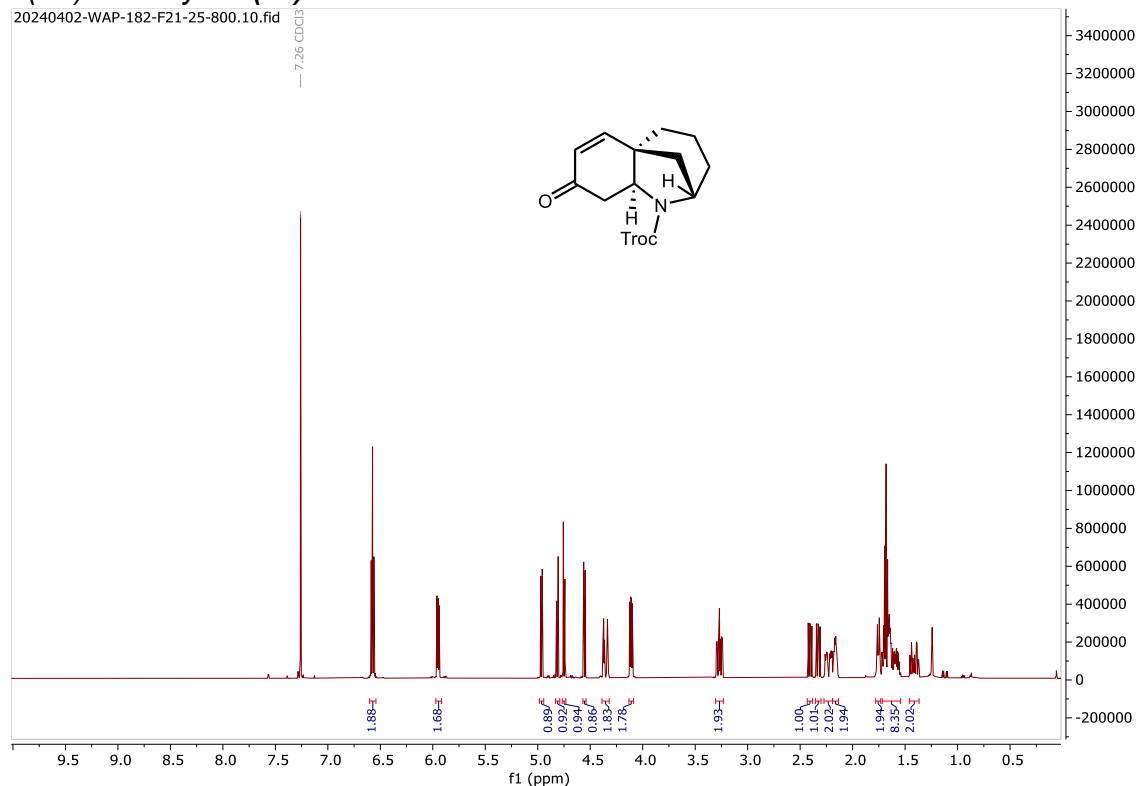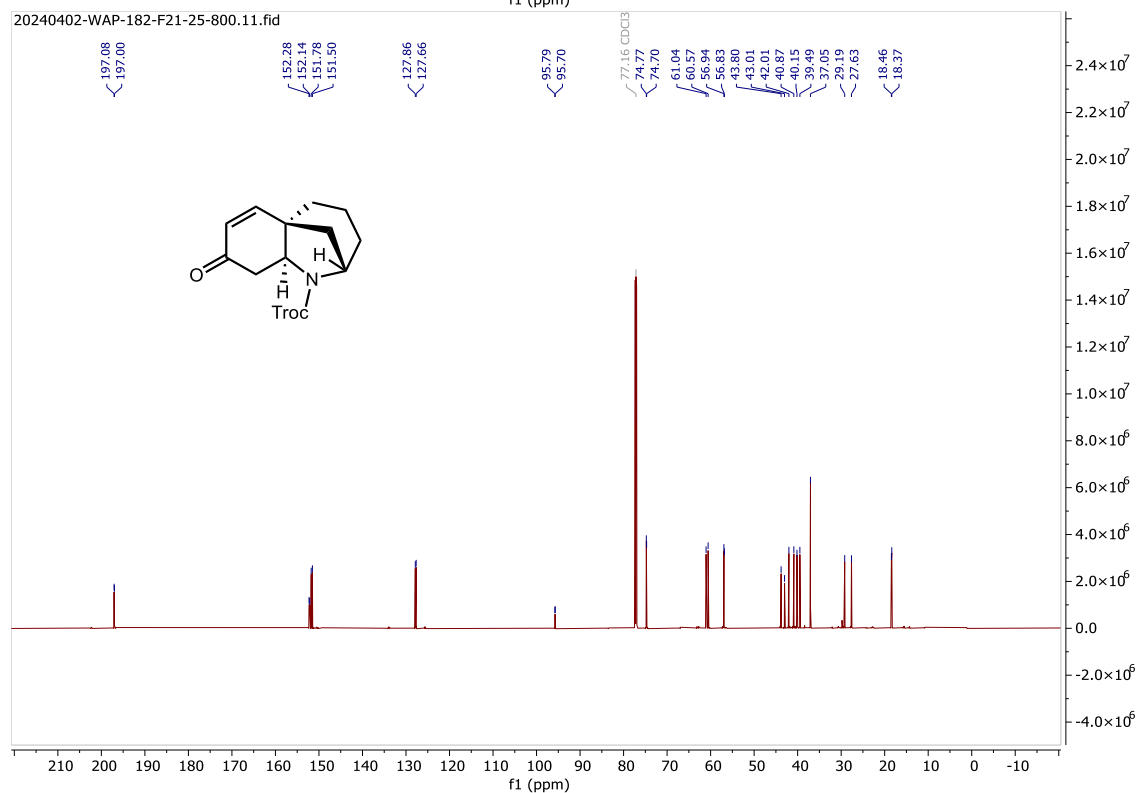

2,2,2-trichloroethyl (2R,5aR,9aS)-8-oxo-2,3,4,5,9,9a-hexahydro-2,5a-methanobenzo[b]azepine-1(8H)-carboxylate ($\pm$ -39) via Standard Conditions:

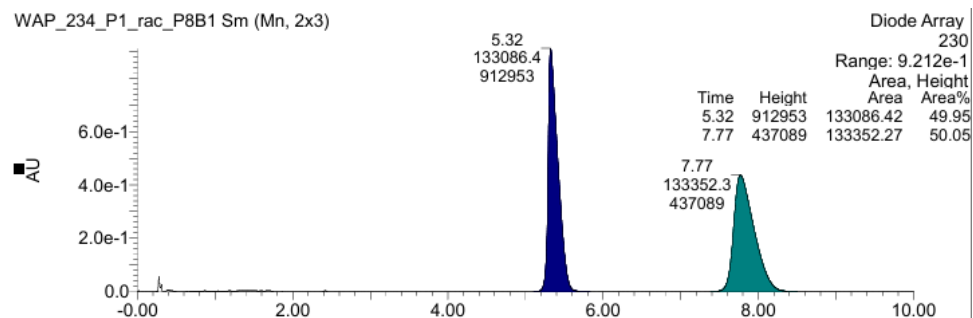

2,2,2-trichloroethyl (2R,5aR,9aS)-8-oxo-2,3,4,5,9,9a-hexahydro-2,5a-methanobenzo[b]azepine-1(8H)-carboxylate (39) via Standard Conditions:

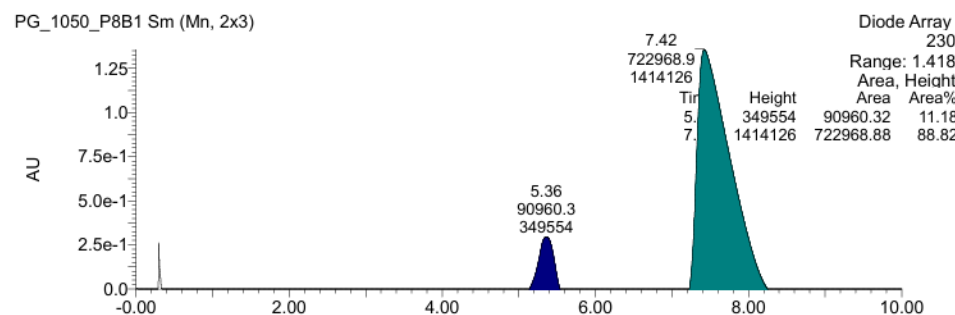

2,2,2-trichloroethyl (2R,5aR,9aS)-8-oxo-2,3,4,5,9,9a-hexahydro-2,5a-methanobenzo[b]azepine-1(8H)-carboxylate (39) via Acidic Conditions:

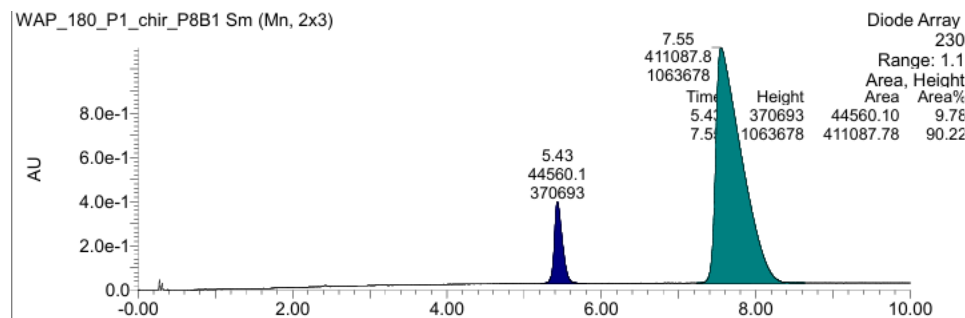

2,2,2-trichloroethyl (*R*)-(5-methoxy-1,2,3,4-tetrahydronaphthalen-2-yl)carbamate (**20'**) and 2,2,2-trichloroethyl (*R*)-(8-methoxy-1,2,3,4-tetrahydronaphthalen-2-yl)carbamate (**20**):

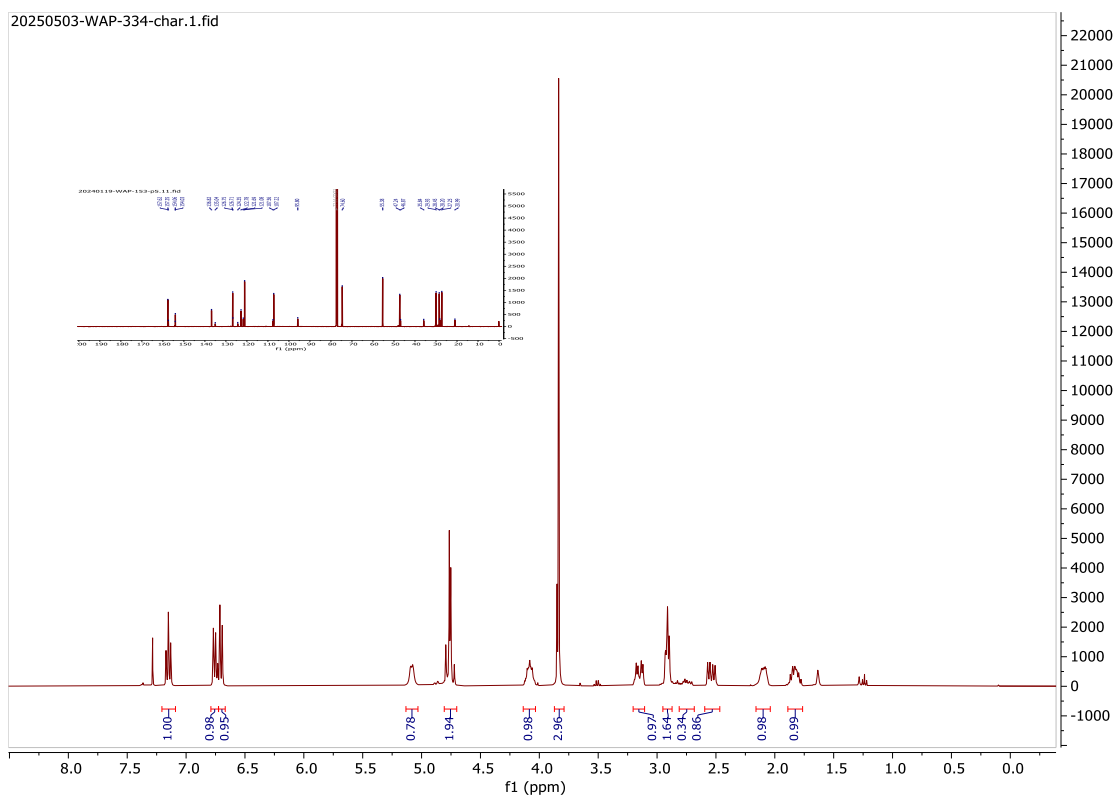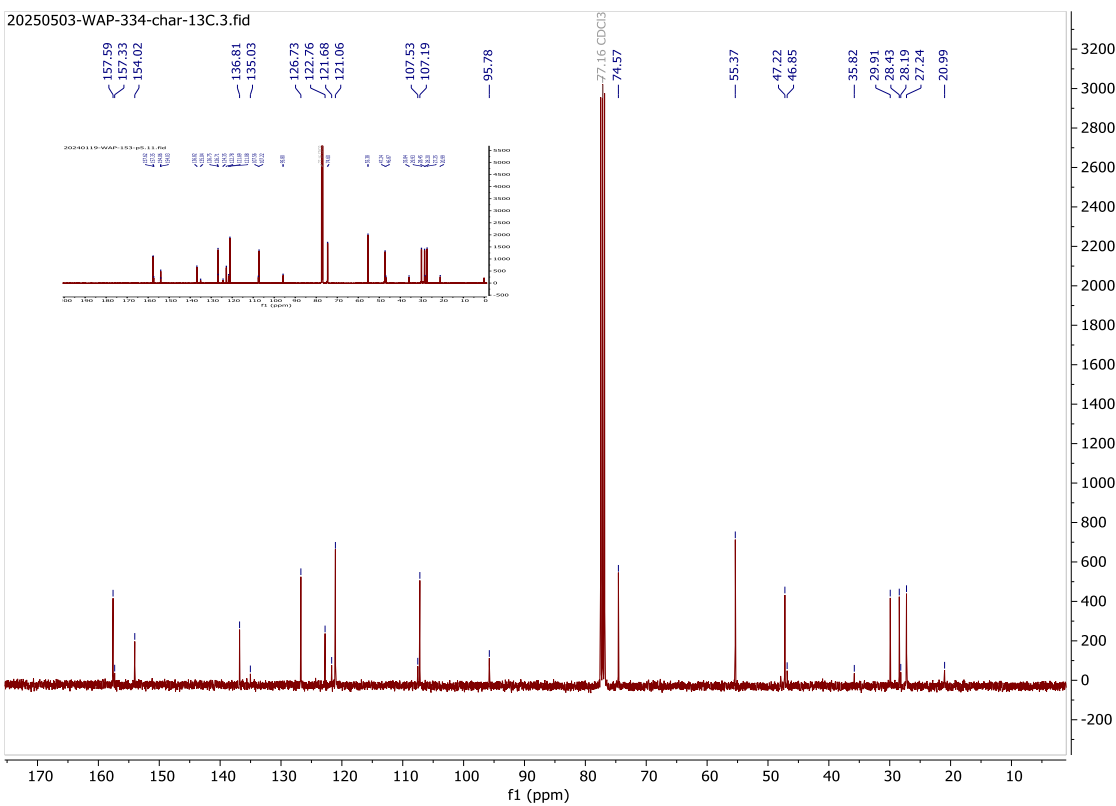

2,2,2-trichloroethyl (5-methoxy-1,2,3,4-tetrahydronaphthalen-2-yl)carbamate (20):

Signal 2: DAD1 B, Sig=210,4 Ref=off

Peak #	RetTime [min]	Type	Width [min]	Area [mAU*s]	Height [mAU]	Area %
1	9.356	VB	0.1939	9832.21582	770.42181	49.5640
2	11.584	BV R	0.2432	1.00052e4	586.10193	50.4360

Totals : 1.98374e4 1356.52374

Signal 2: DAD1 B, Sig=210,4 Ref=off

Peak #	RetTime [min]	Type	Width [min]	Area [mAU*s]	Height [mAU]	Area %
1	8.843	VV R	0.1734	1462.58716	116.43881	54.5366
2	10.639	BV R	0.1864	1219.25684	90.63749	45.4634

Totals : 2681.84399 207.07630

2,2,2-trichloroethyl (R)-(5-methoxy-1,2,3,4-tetrahydronaphthalen-2-yl)carbamate (20):

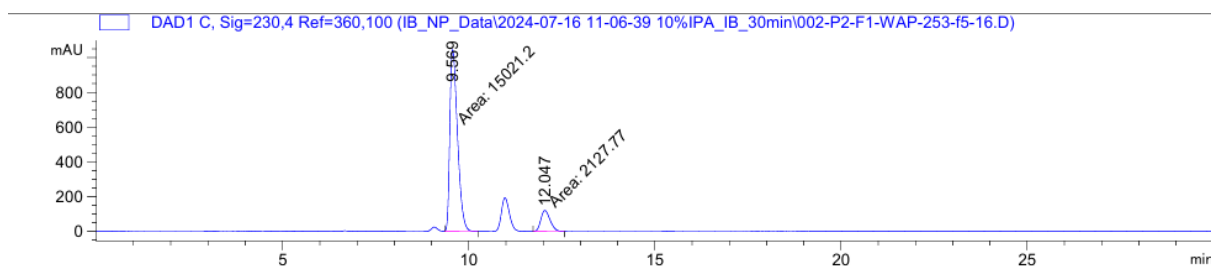

Signal 3: DAD1 C, Sig=230,4 Ref=360,100

Peak #	RetTime [min]	Type	Width [min]	Area [mAU*s]	Height [mAU]	Area %
1	9.569	MM	0.2392	1.50212e4	1046.75012	87.5924
2	12.047	MM	0.2927	2127.77393	121.16650	12.4076

Totals : 1.71489e4 1167.91662

DAD1 C, Sig=230,4 Ref=360,100 (IB_NP_Data\2024-07-16 11-06-39 10%IPA_IB_30min\002-P2-F1-WAP-253-f5-16.D)

2,2,2-trichloroethyl (8-methoxy-1,2,3,4-tetrahydronaphthalen-2-yl)carbamate ($\pm$ -20'):

Signal 2: DAD1 B, Sig=210,4 Ref=off

Peak #	RetTime [min]	Type	Width [min]	Area [mAU*s]	Height [mAU]	Area %
1	8.843	VV R	0.1734	1462.58716	116.43881	54.5366
2	10.639	BV R	0.1864	1219.25684	90.63749	45.4634

Totals : 2681.84399 207.07630

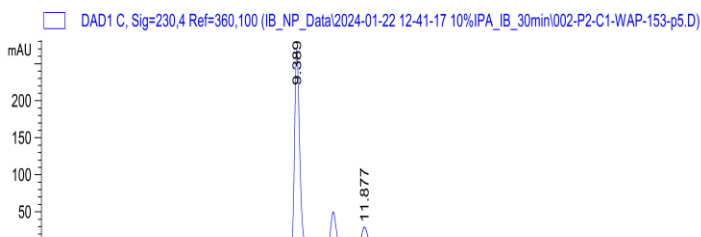

2,2,2-trichloroethyl (R)-(8-methoxy-1,2,3,4-tetrahydronaphthalen-2-yl)carbamate (20'):

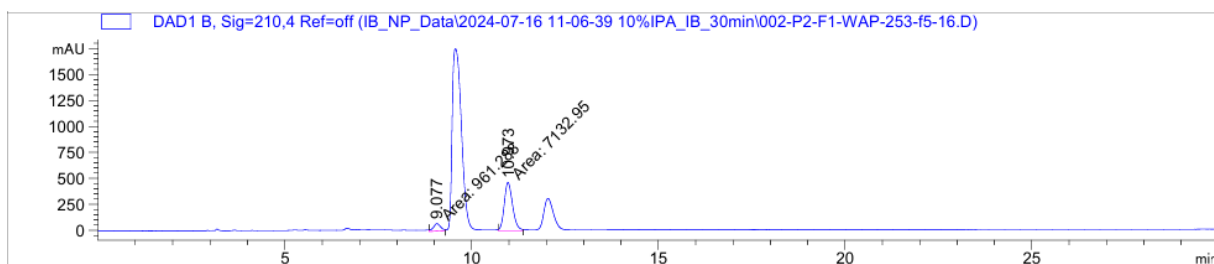

Signal 2: DAD1 B, Sig=210,4 Ref=off

Peak #	RetTime [min]	Type	Width [min]	Area [mAU*s]	Height [mAU]	Area %
1	9.077	MM	0.2267	961.28772	70.67665	11.8762
2	10.973	MM	0.2578	7132.95117	461.08185	88.1238

Totals : 8094.23889 531.75850

(*R*)-8-methoxy-1,2,3,4-tetrahydronaphthalen-2-amine (**40**) and (*R*)-5-methoxy-1,2,3,4-tetrahydronaphthalen-2-amine (**40'**):

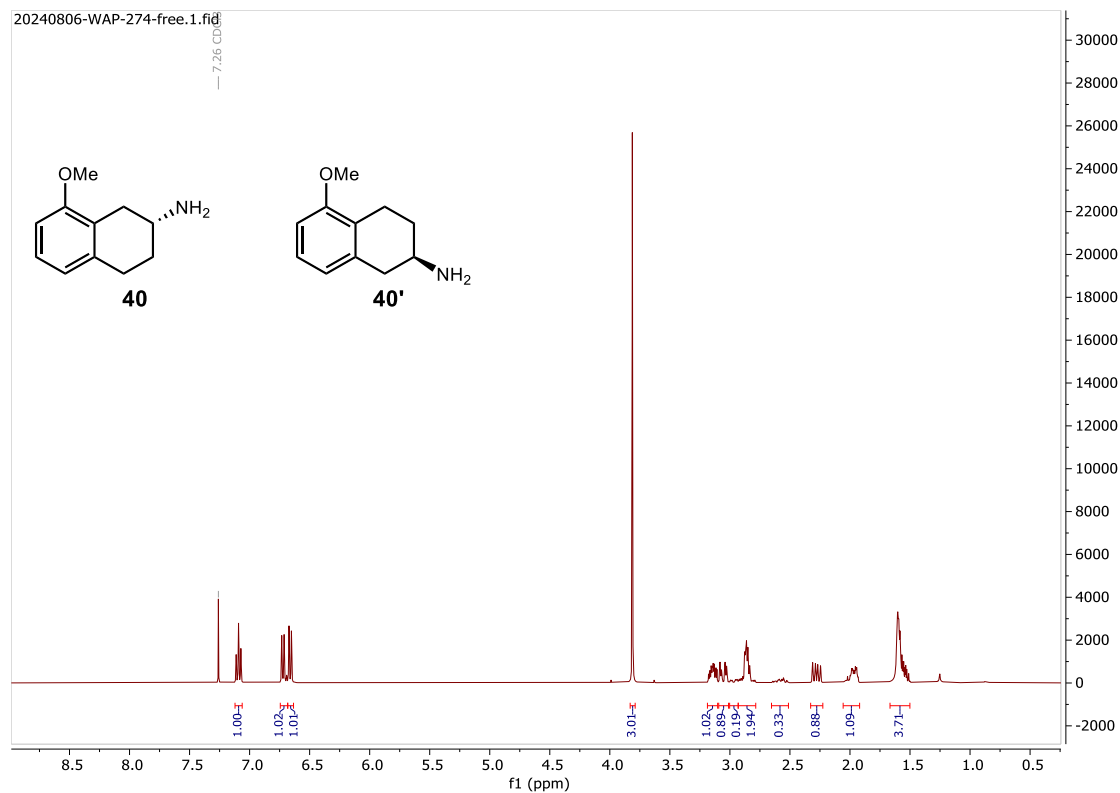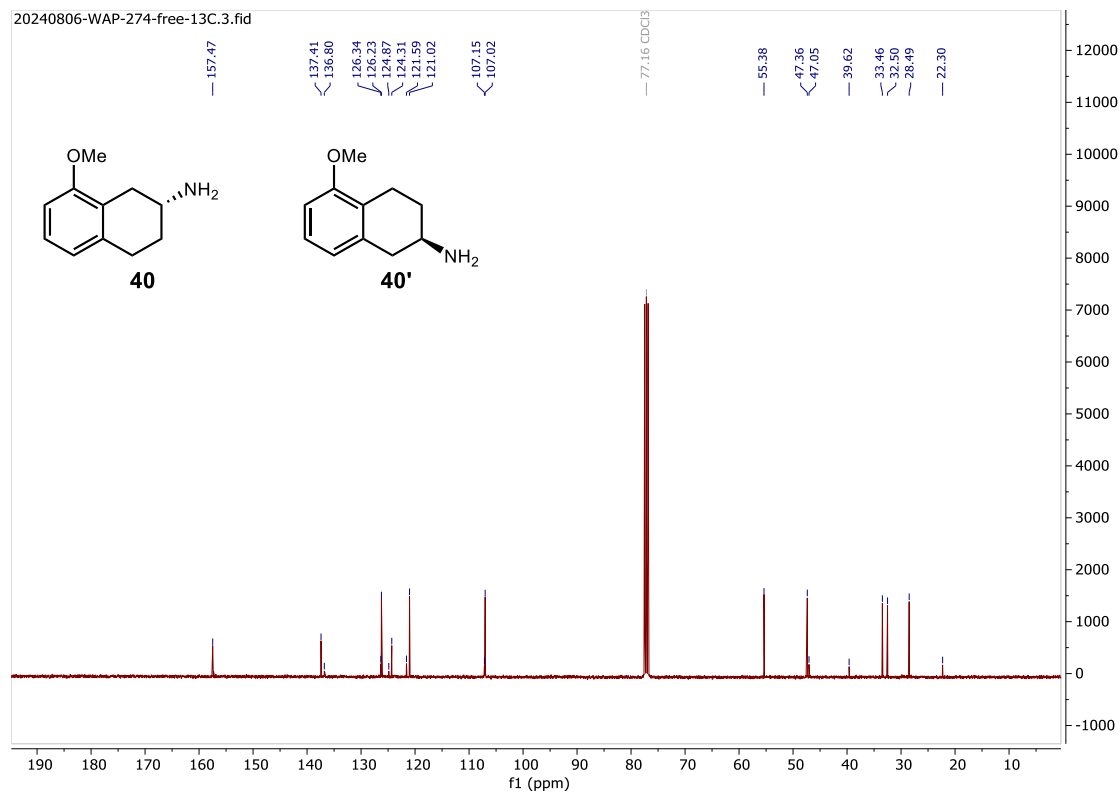

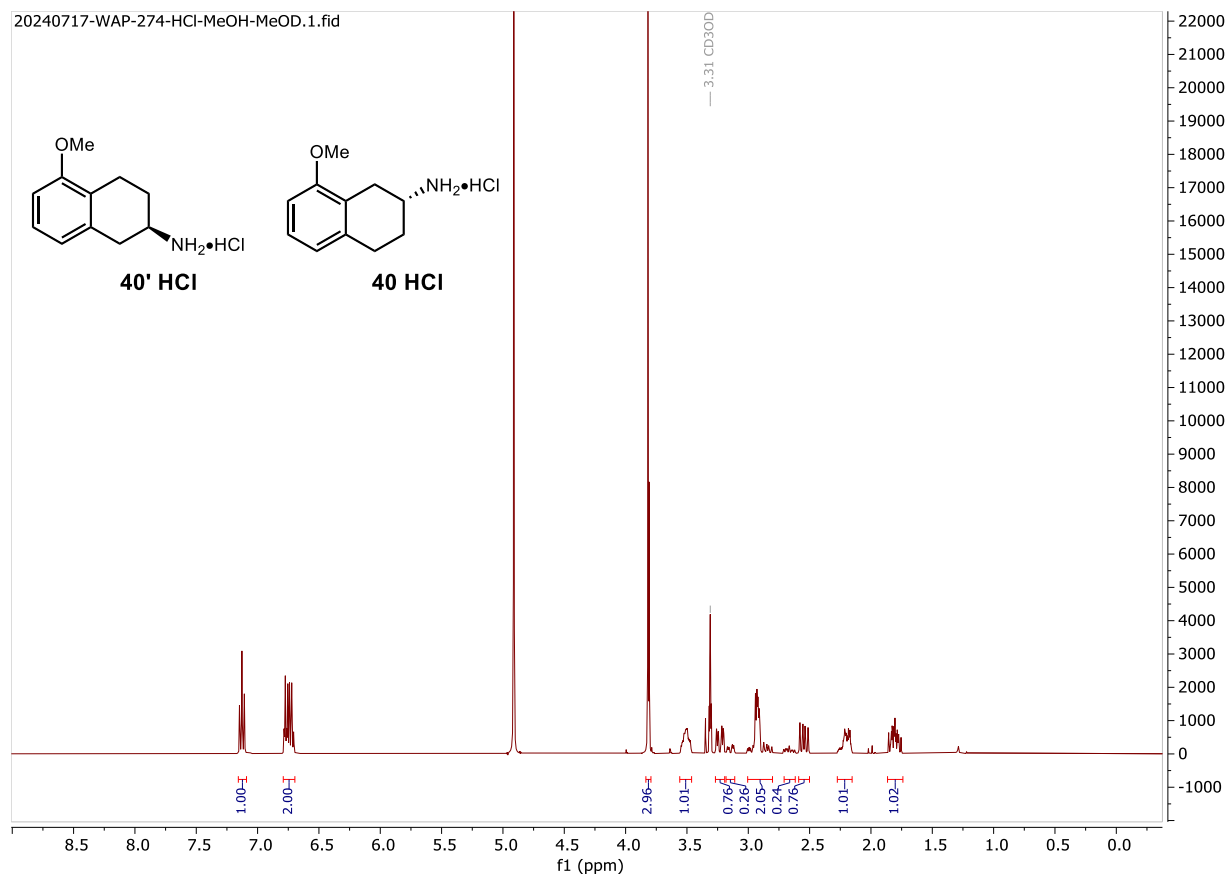

(R)-8-methoxy-N,N-dipropyl-1,2,3,4-tetrahydronaphthalen-2-amine (S30) and **(R)-5-methoxy-N,N-dipropyl-1,2,3,4-tetrahydronaphthalen-2-amine (S30')**:

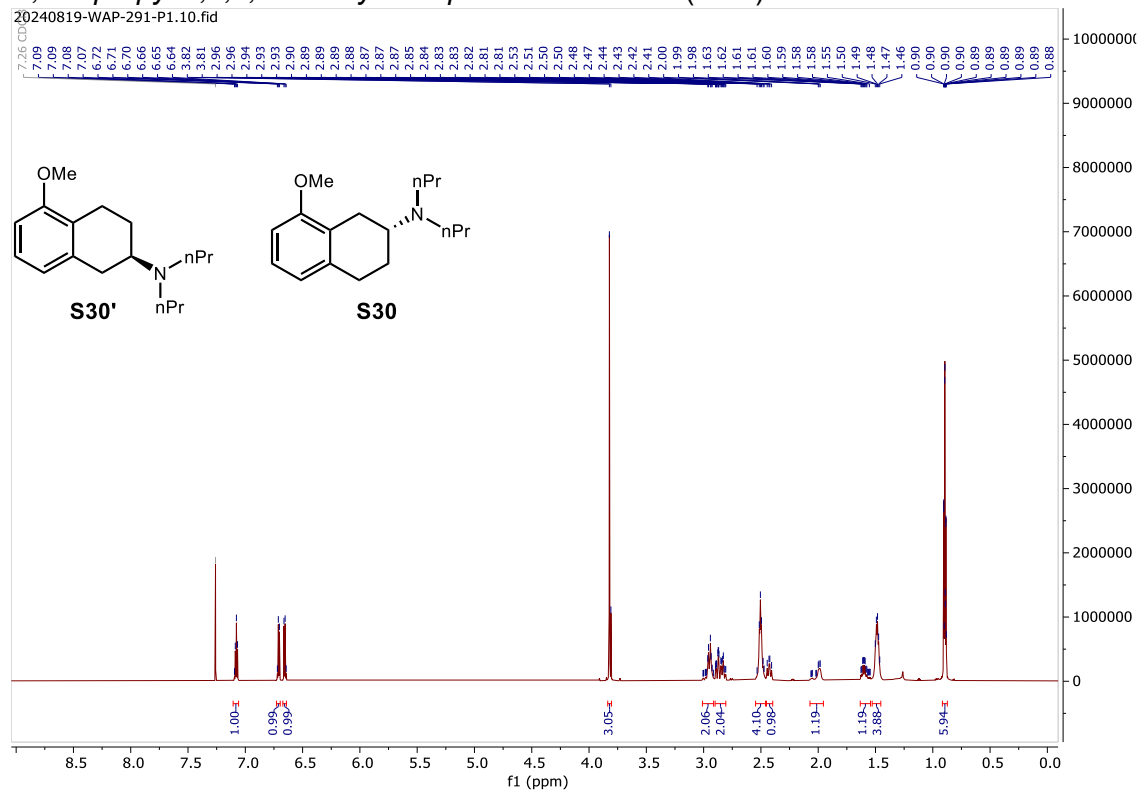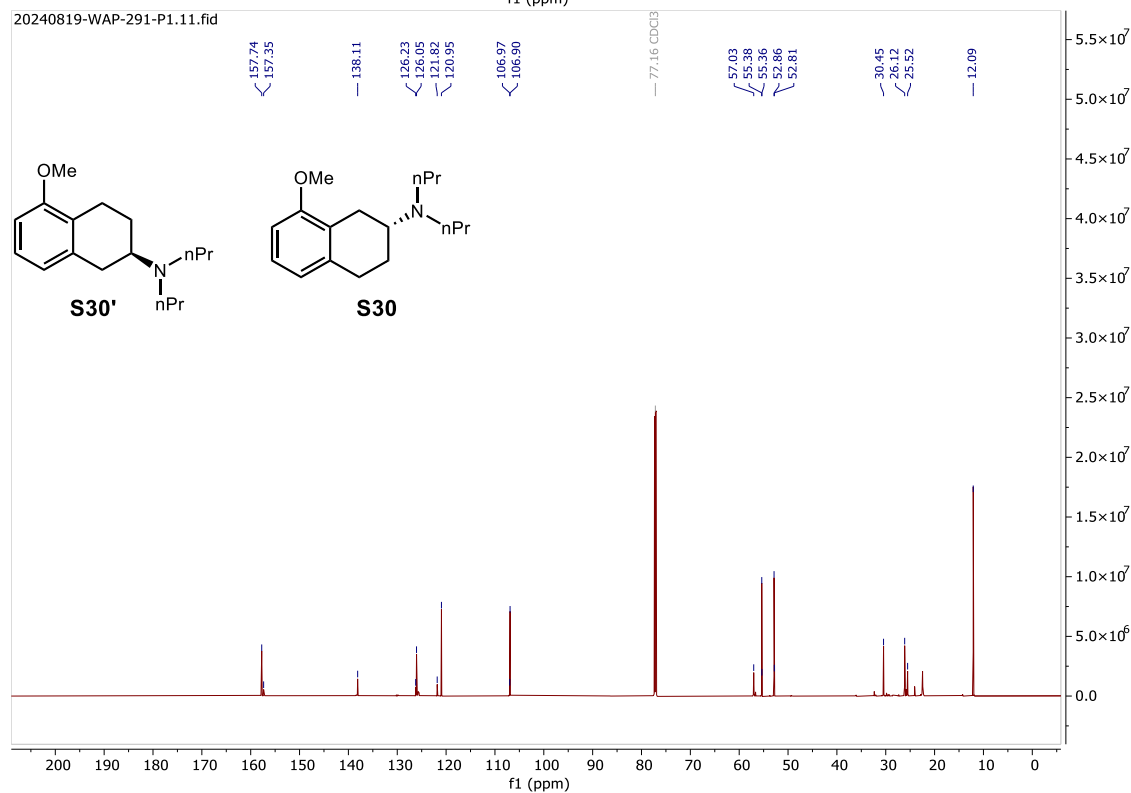

(*R*)-7-(dipropylamino)-5,6,7,8-tetrahydronaphthalen-1-ol (**1**) and (*R*)-6-(dipropylamino)-5,6,7,8-tetrahydronaphthalen-1-ol (**1'**):

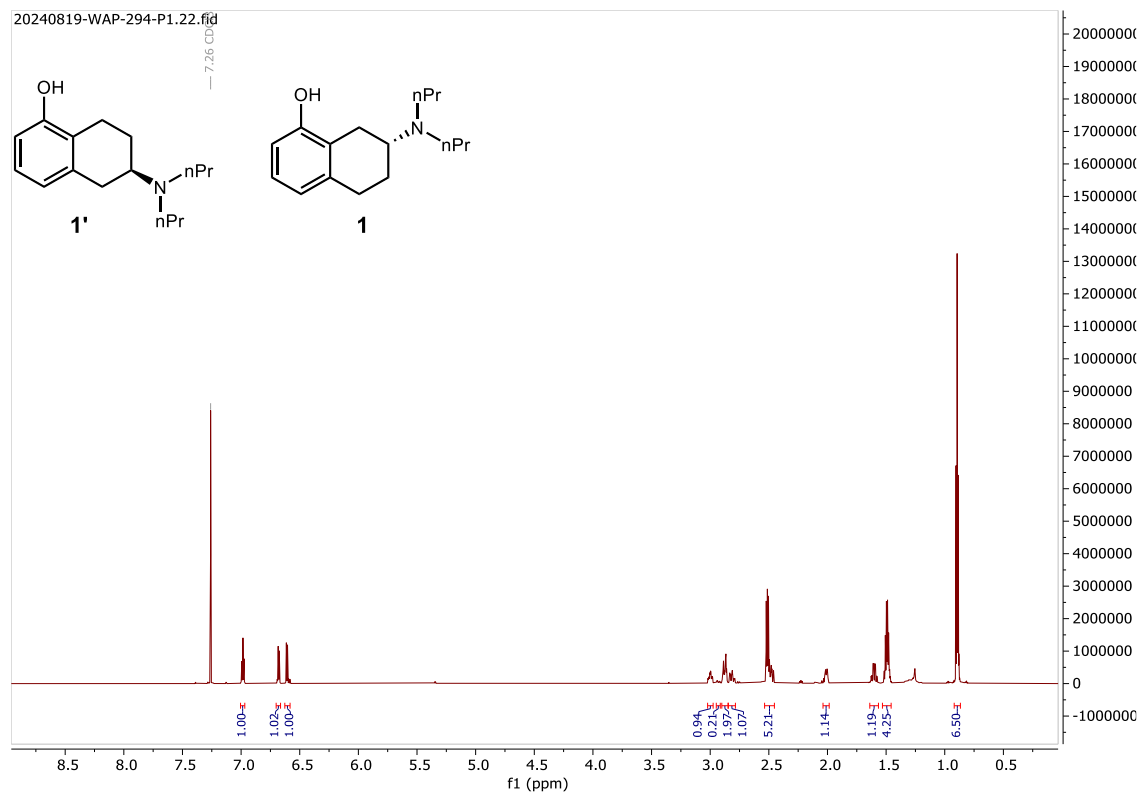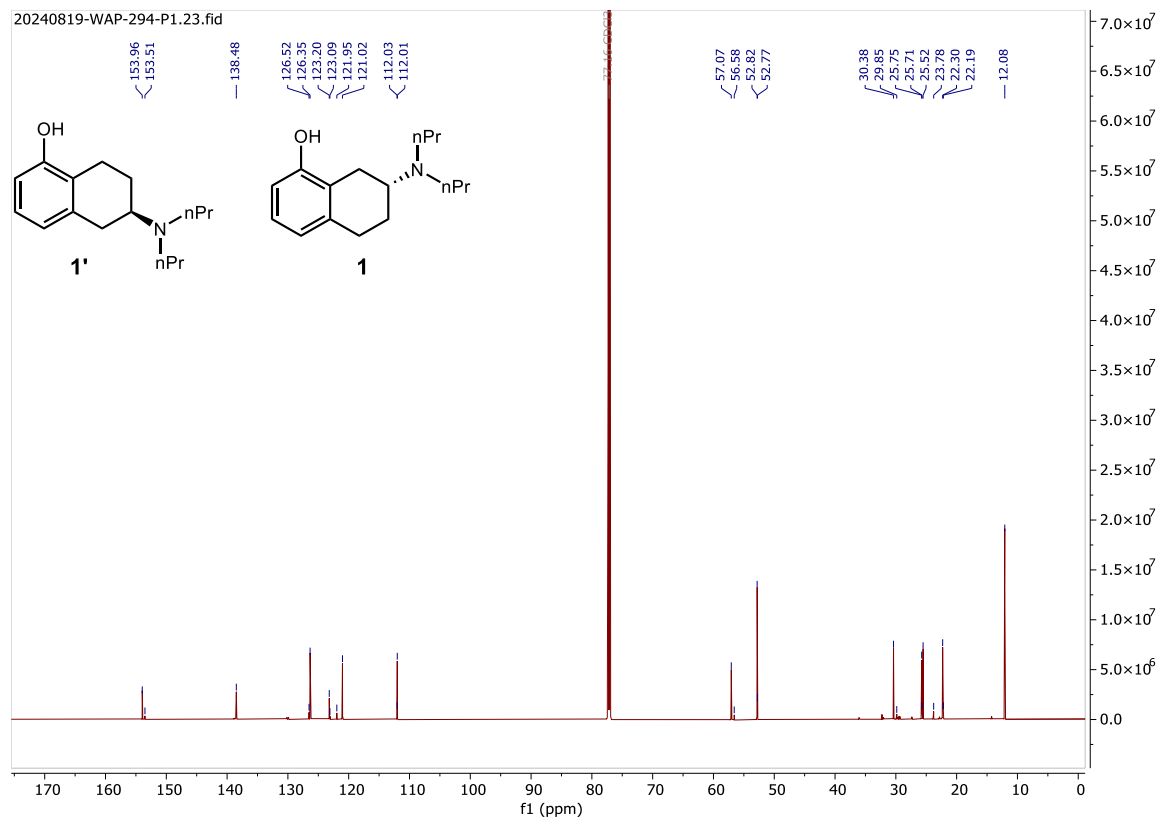

4-(but-3-en-1-yl)-1-tosyl-1H-indole (S10):

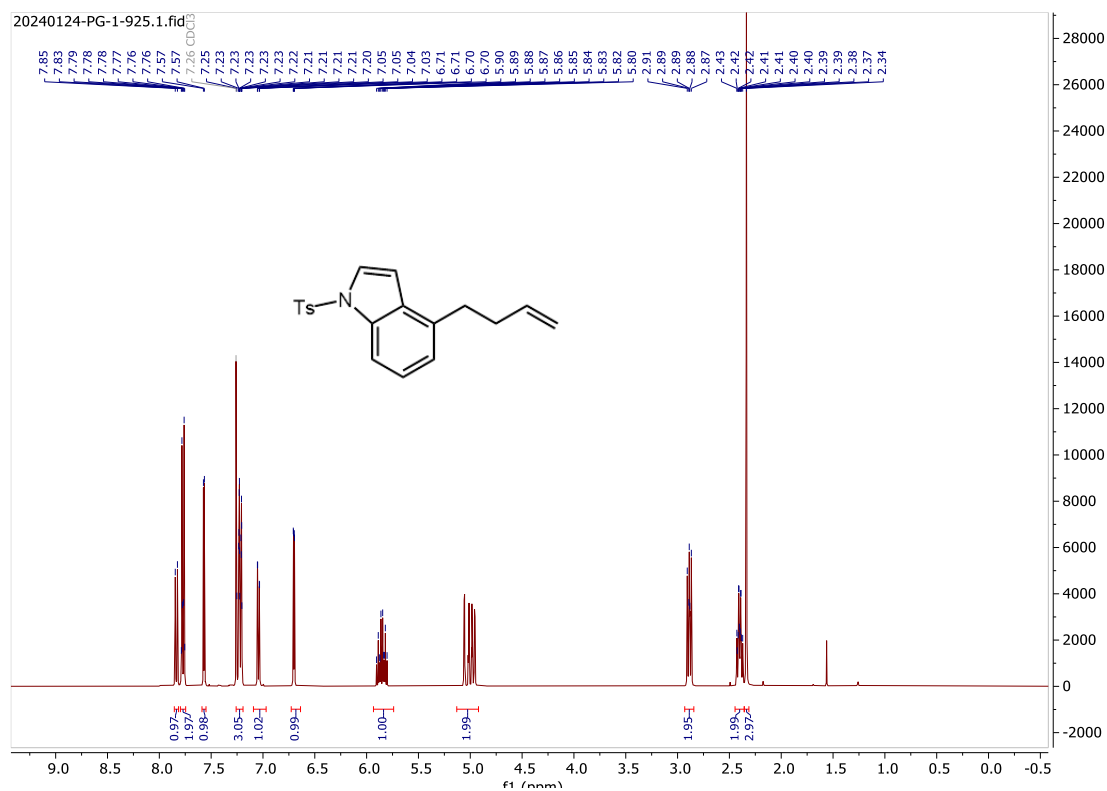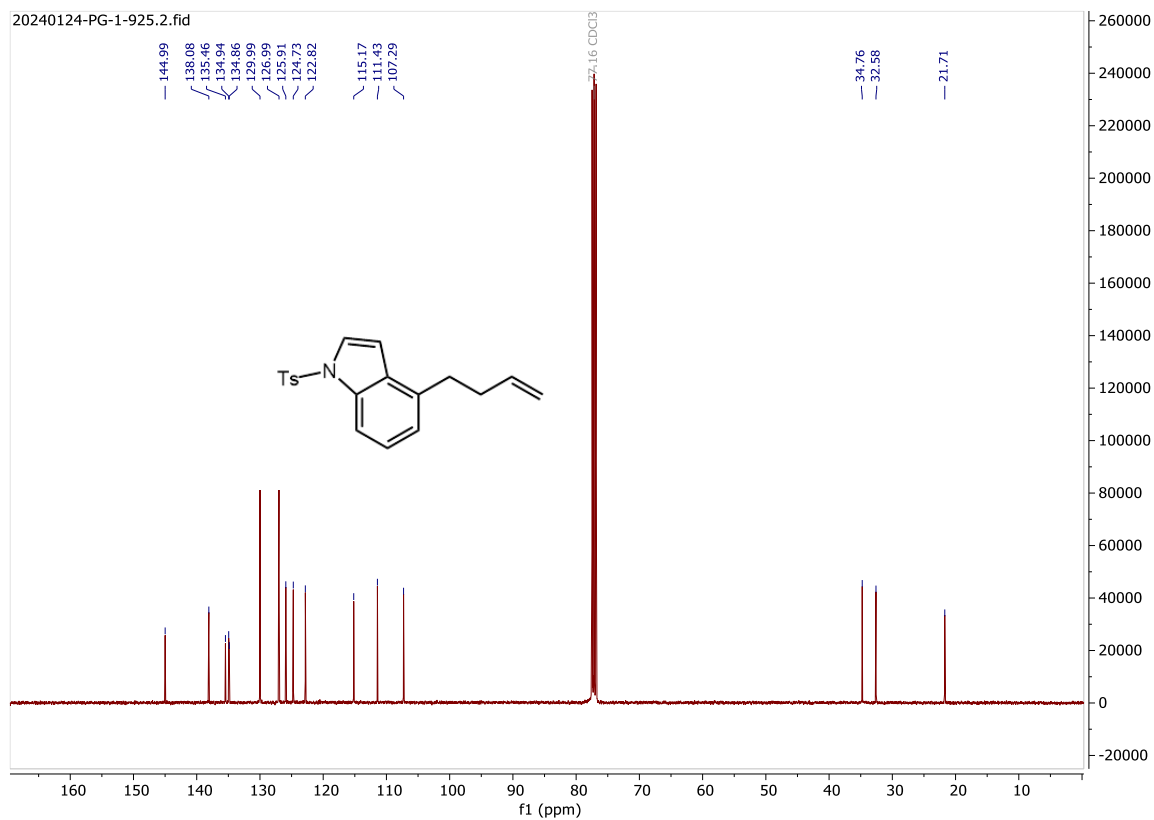

(4-(but-3-en-1-yl)phenoxy)trimethylsilane (**S12**):

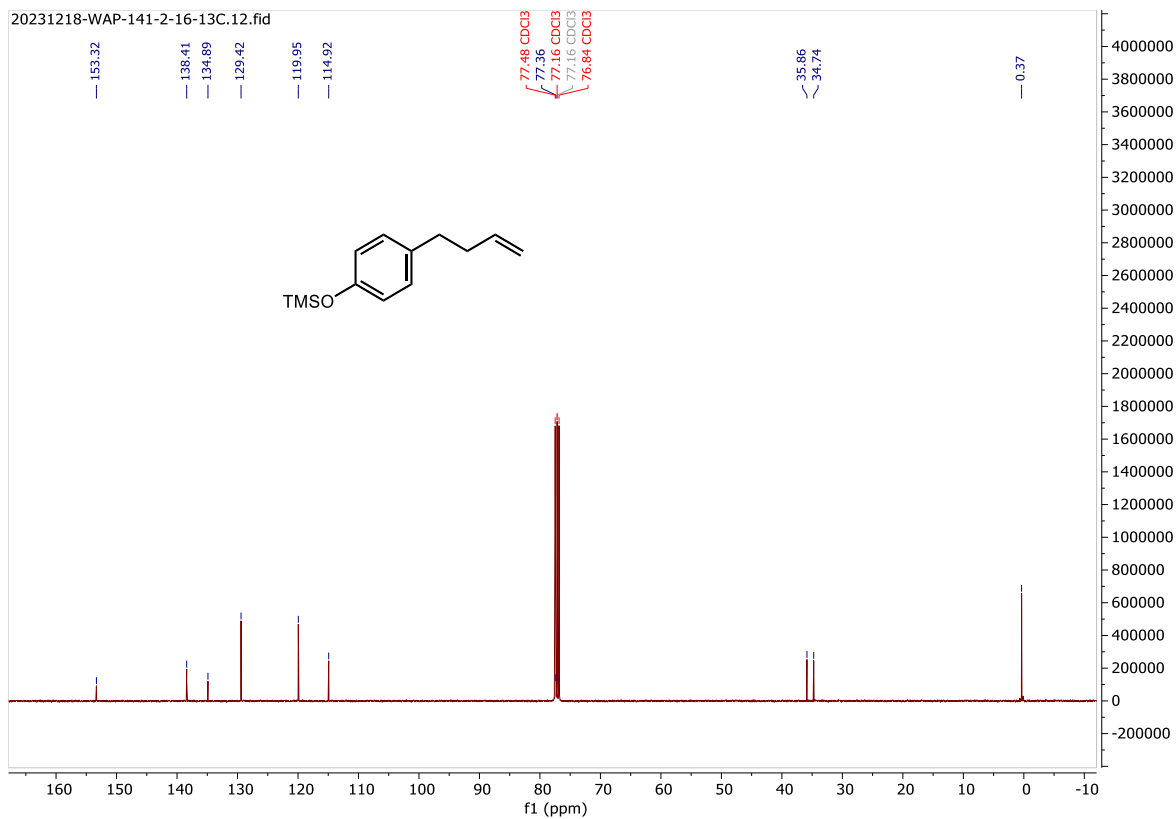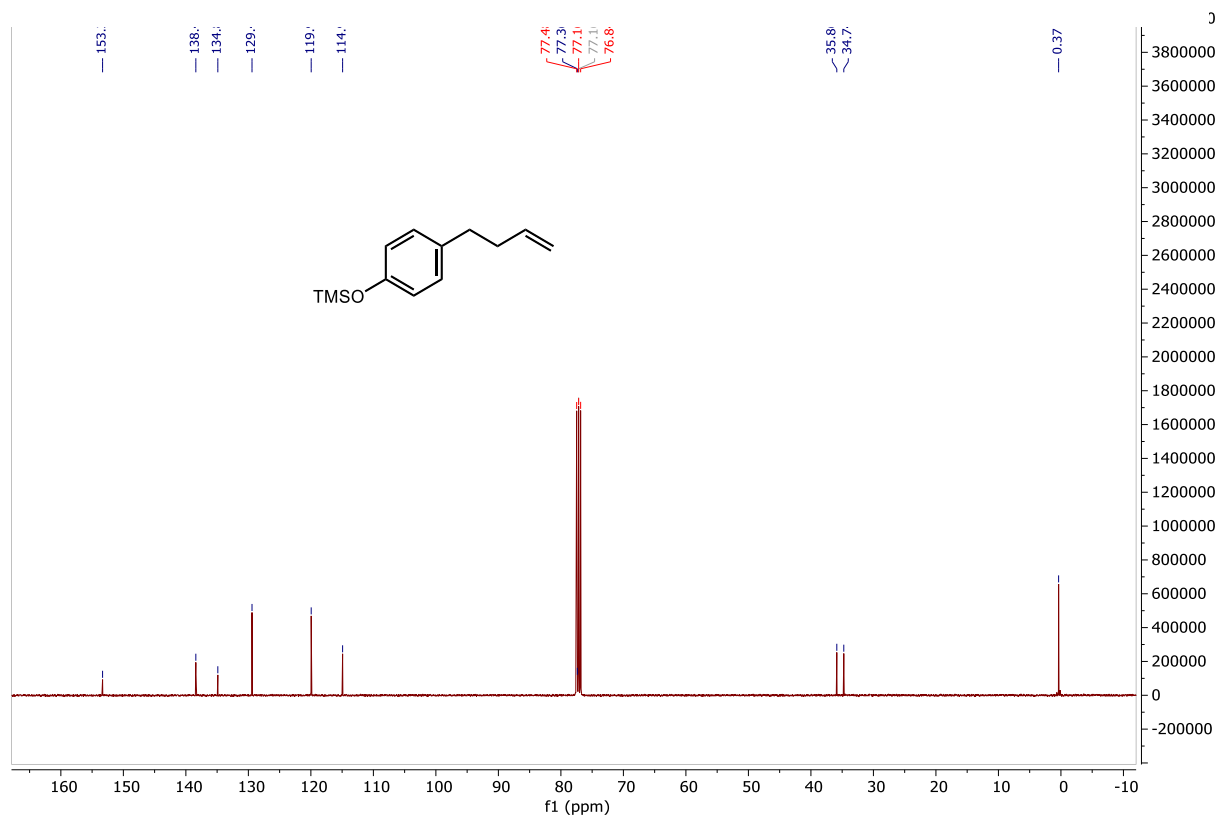

((1-(but-3-en-1-yl)naphthalen-2-yl)oxy)(tert-butyl)dimethylsilane (S13):

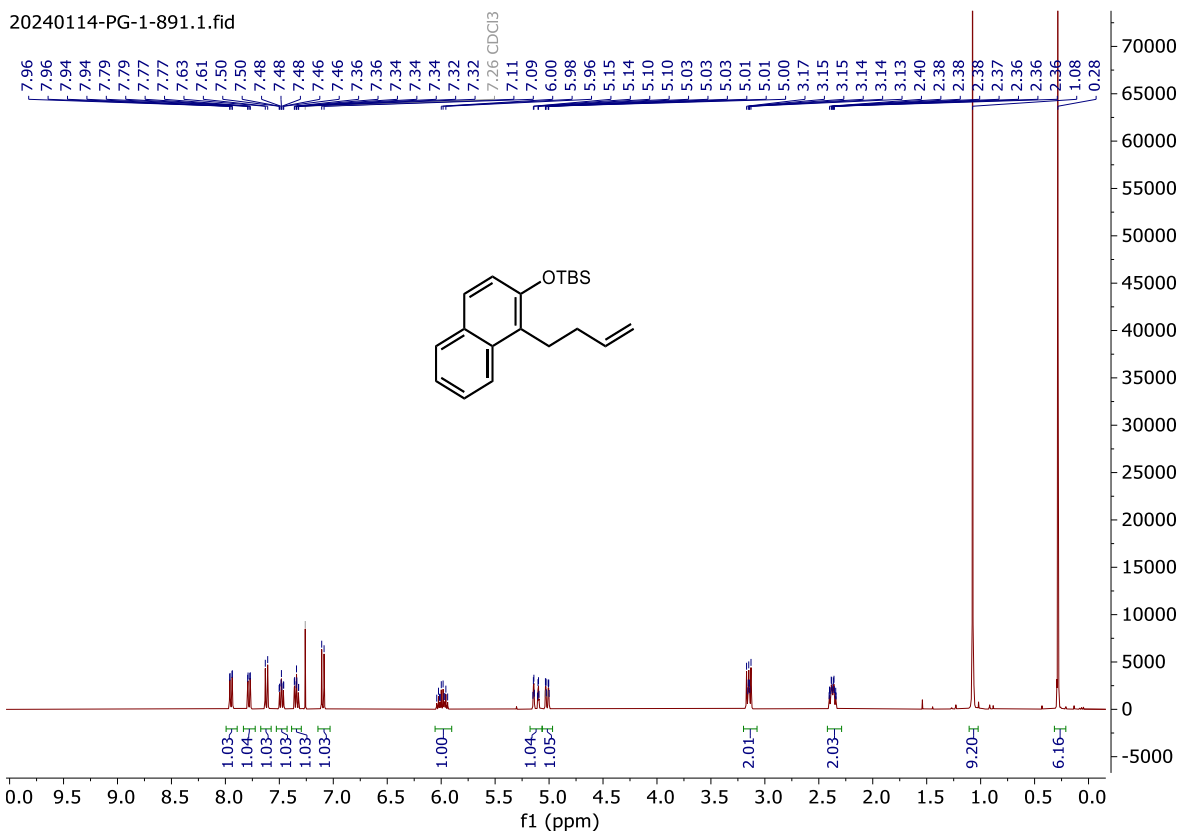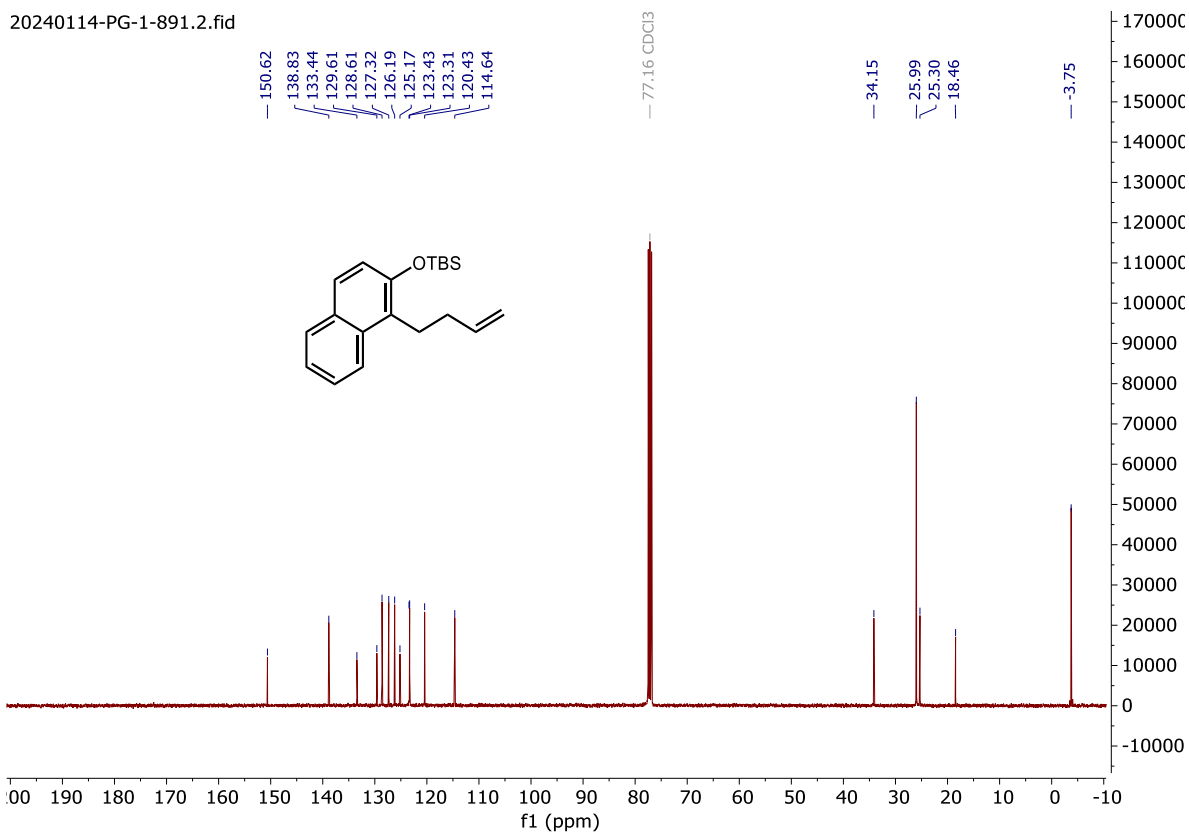

trimethyl(4-(pent-4-en-1-yl)phenoxy)silane (S14):

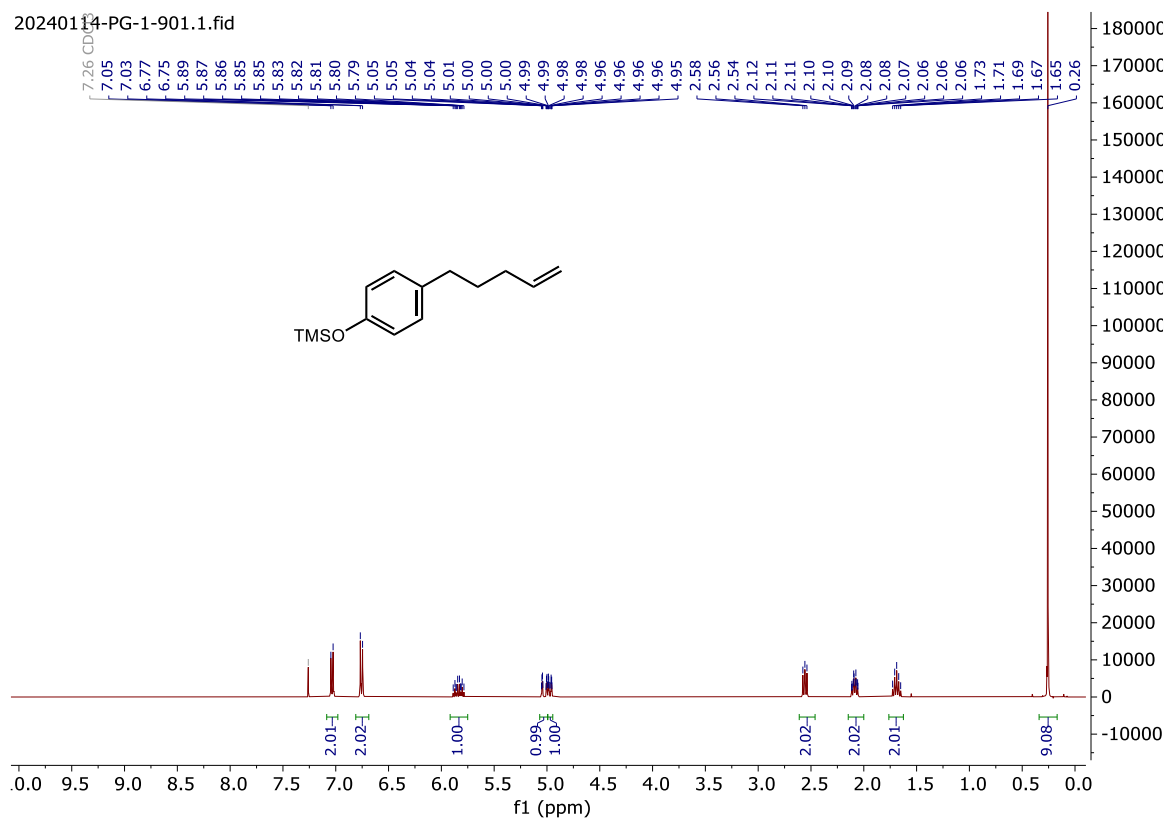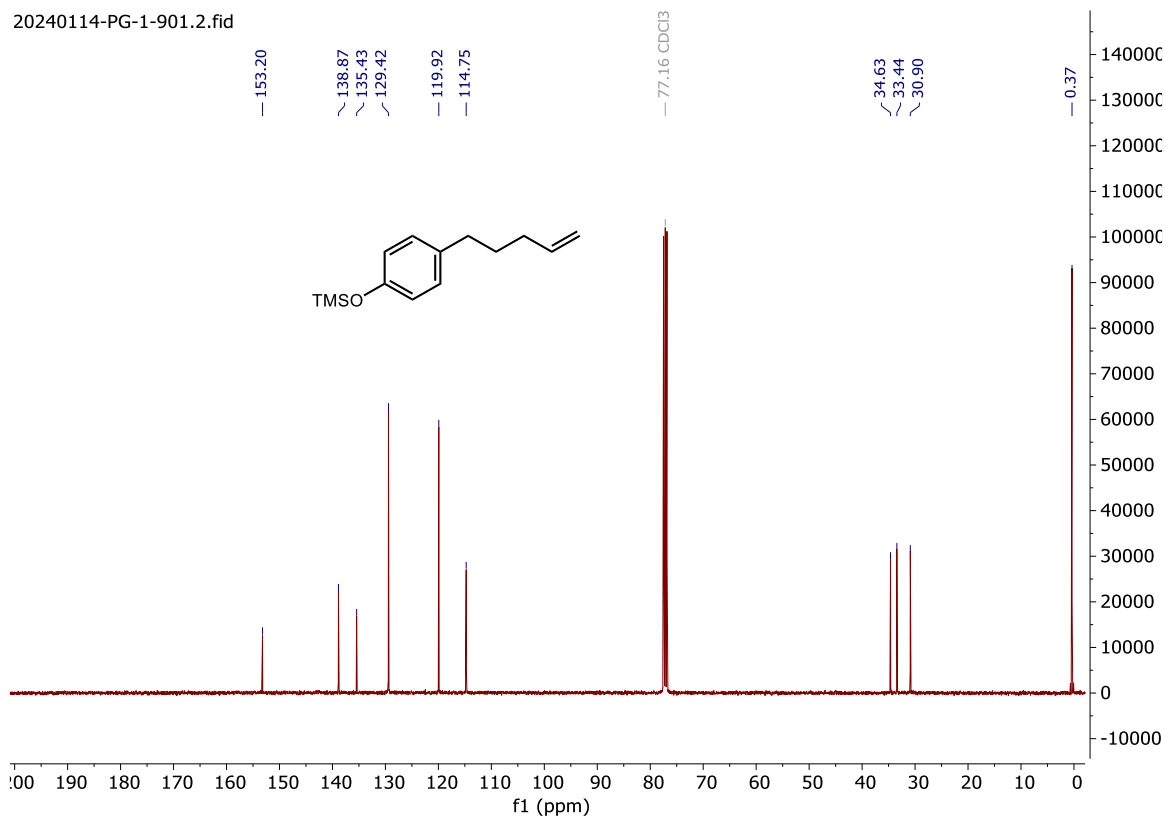

***tert*-butyldimethyl((1-(pent-4-en-1-yl)naphthalen-2-yl)oxy)silane (S15):**

20231202-PG-1-1004-1-24.1.fid

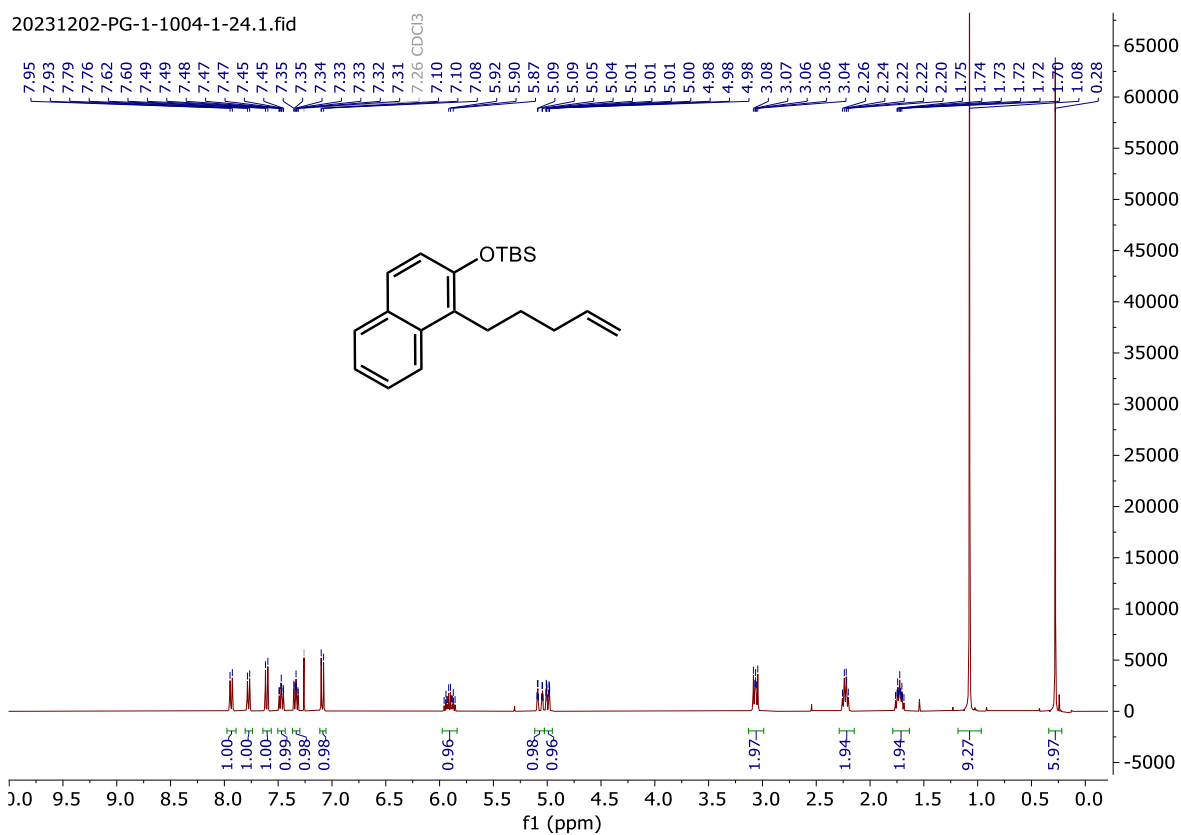

20231202-PG-1-1004-1-24.2.fid

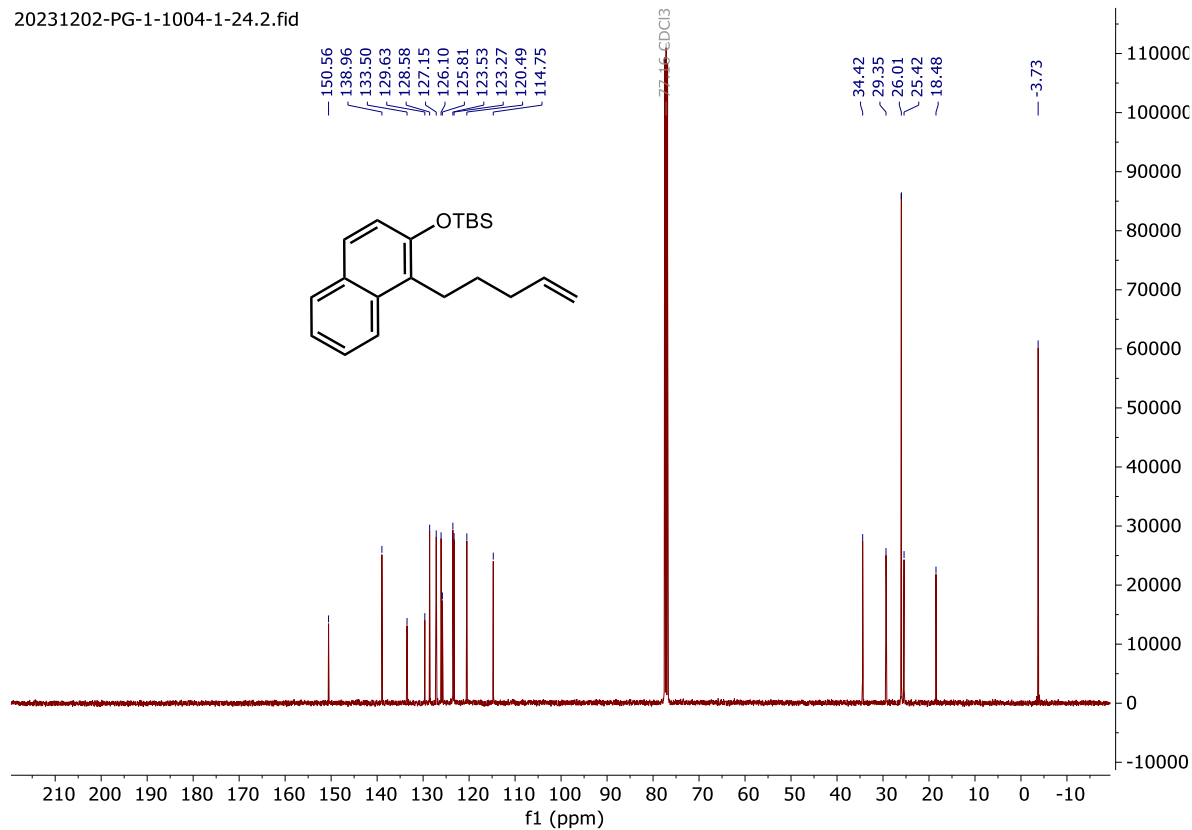

***tert*-butyl(2-methoxy-4-(pent-4-en-1-yl)phenoxy)dimethylsilane (S16):**

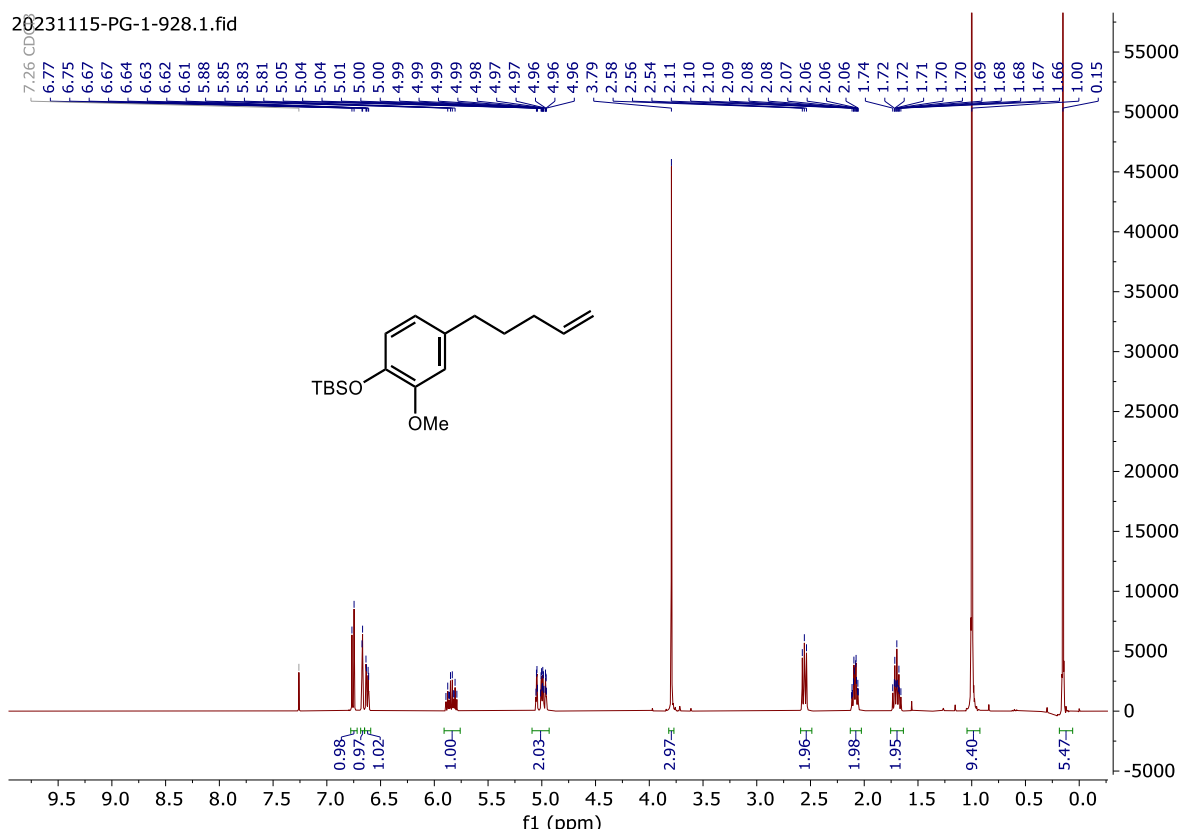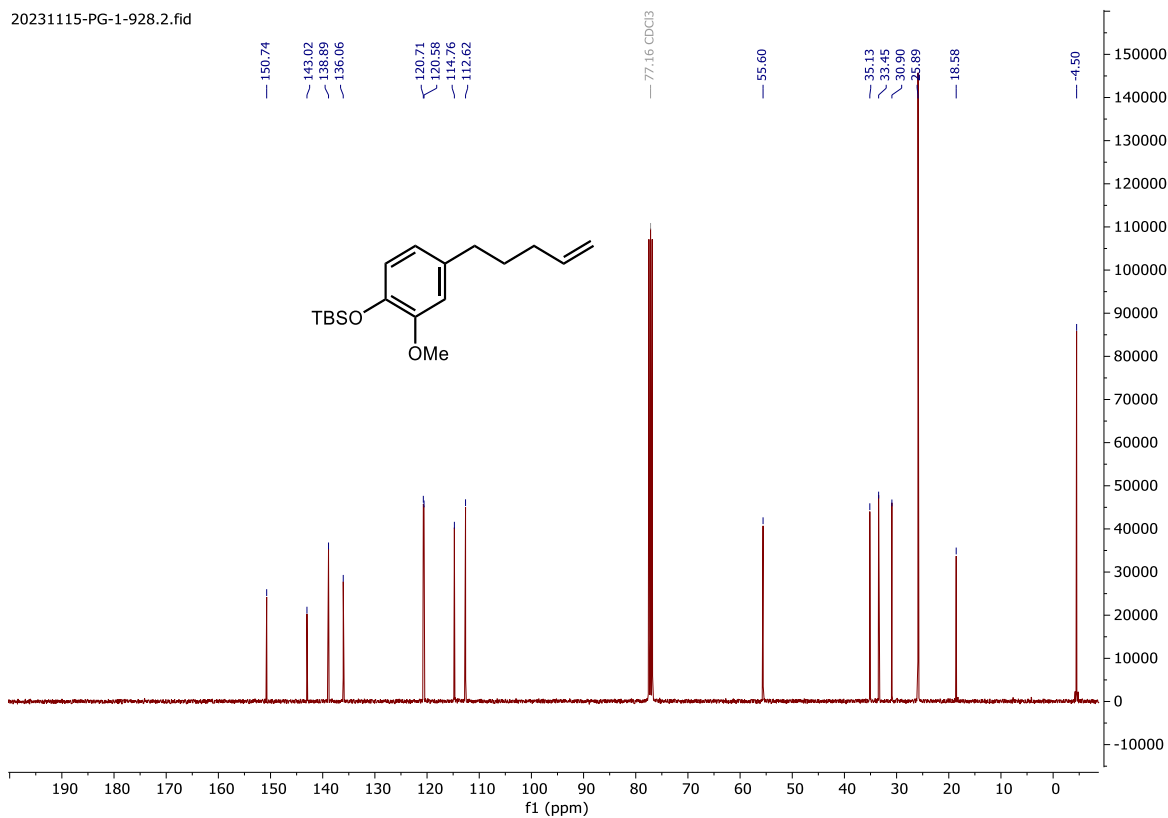

tert-butyl(3-methoxy-4-(pent-4-en-1-yl)phenoxy)dimethylsilane (**S17**):

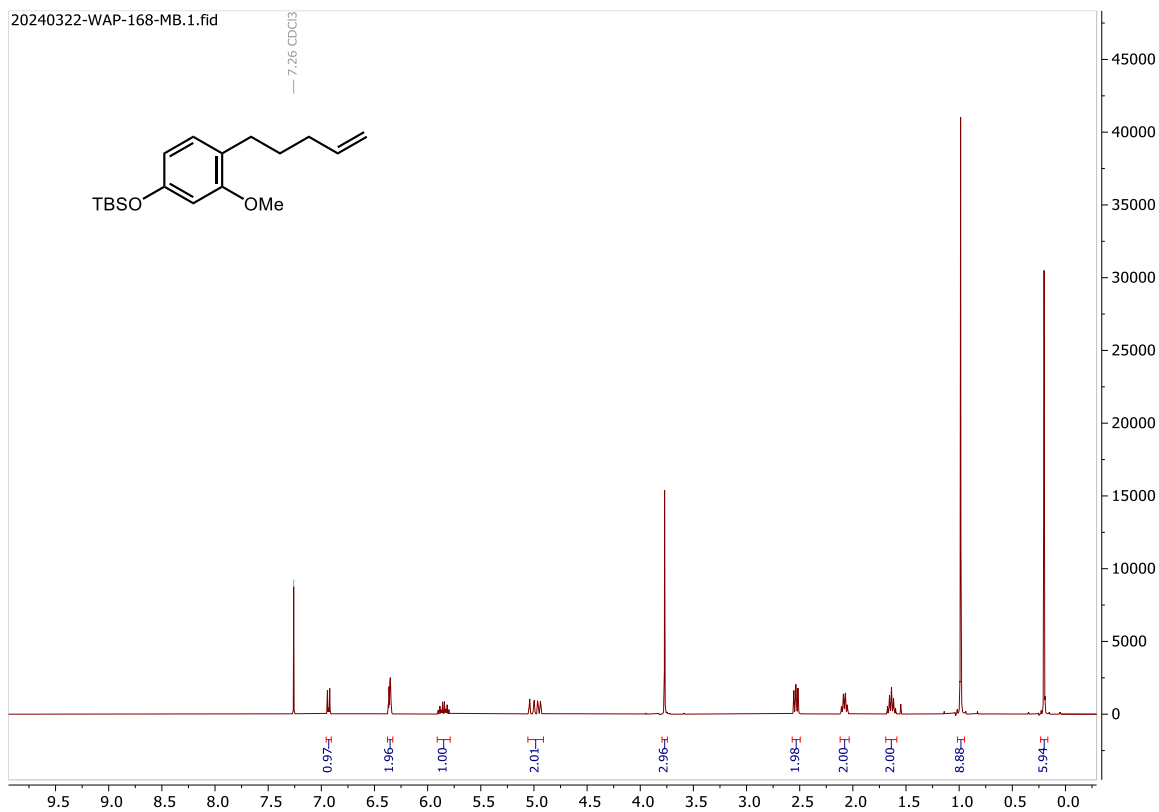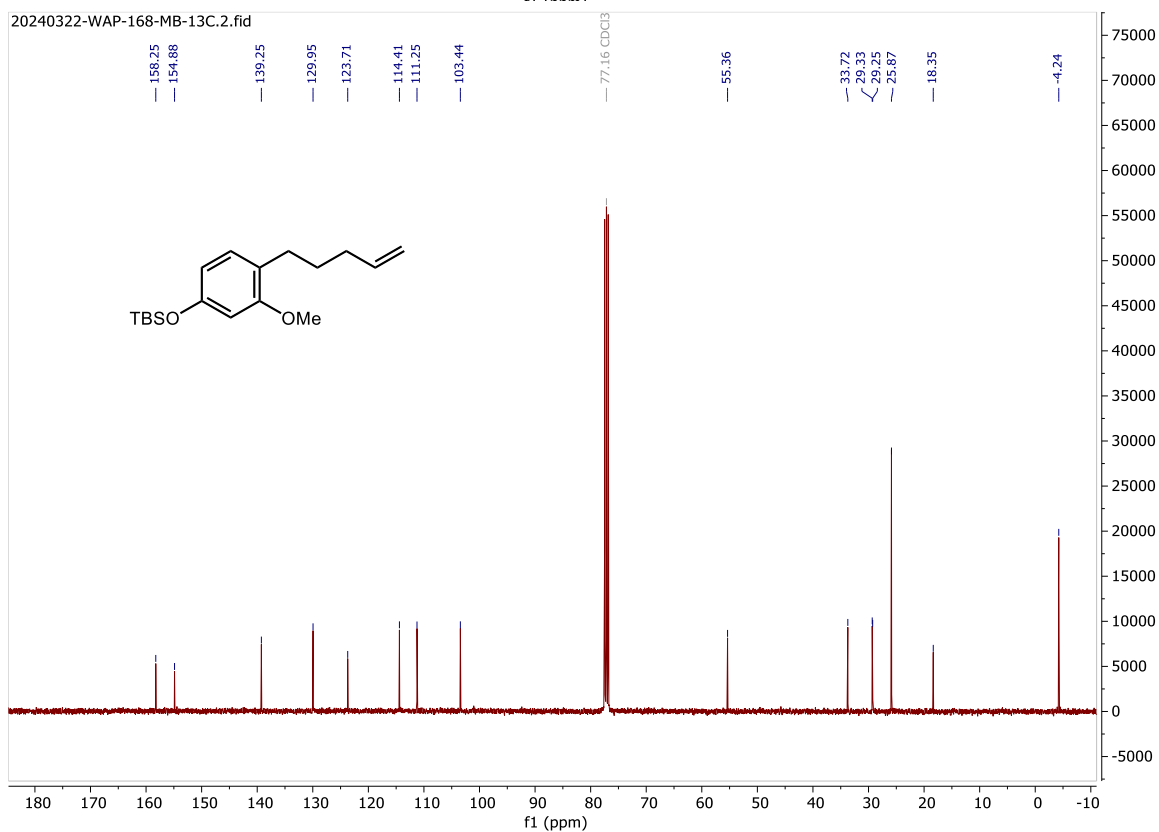

2,2,2-trichloroethyl acetoxycarbamate (S23):

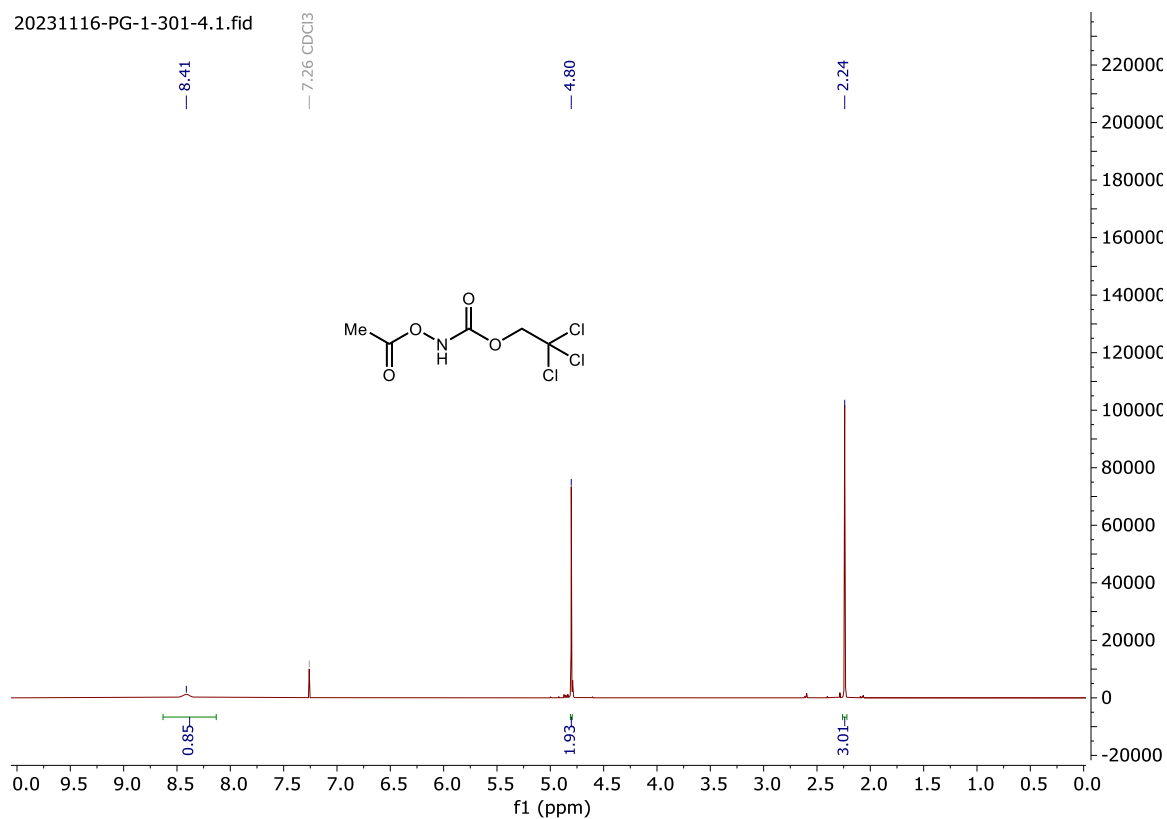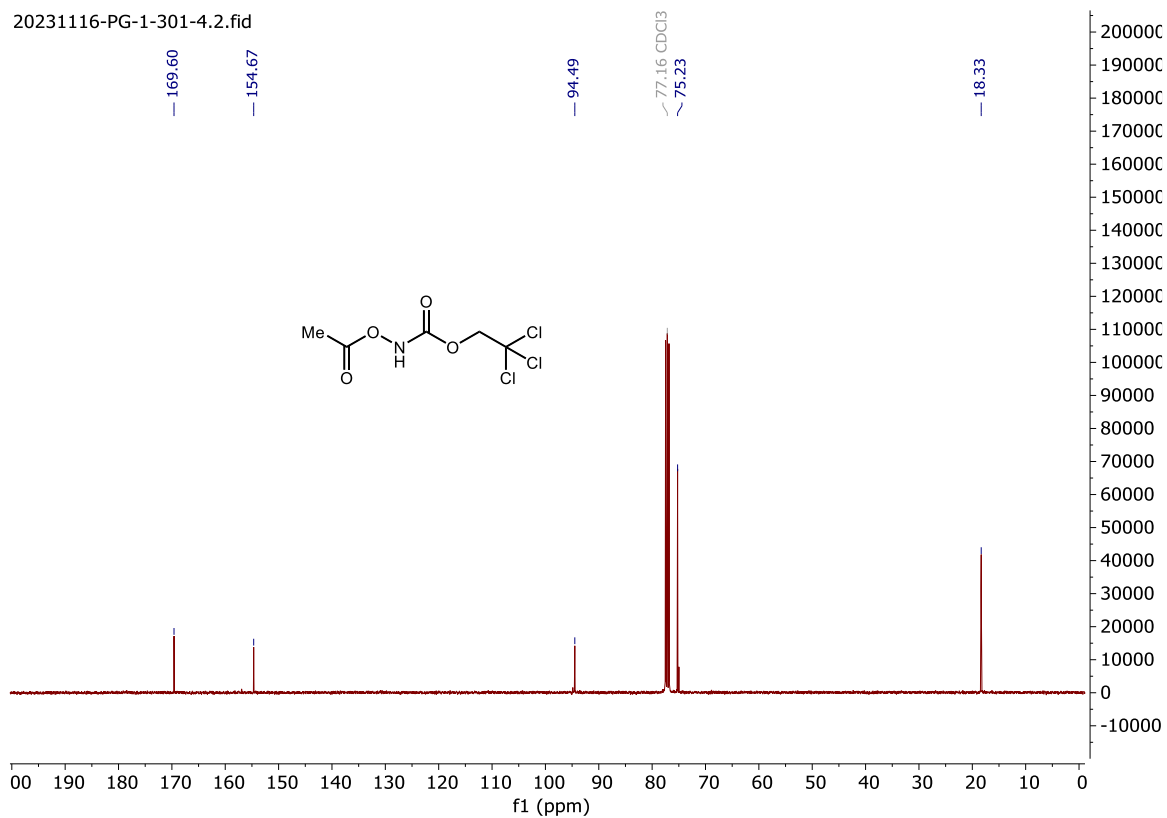

1-(4-((*tert*-butyldimethylsilyl)oxy)-3-methoxyphenyl)pent-4-en-1-ol (**S24**):

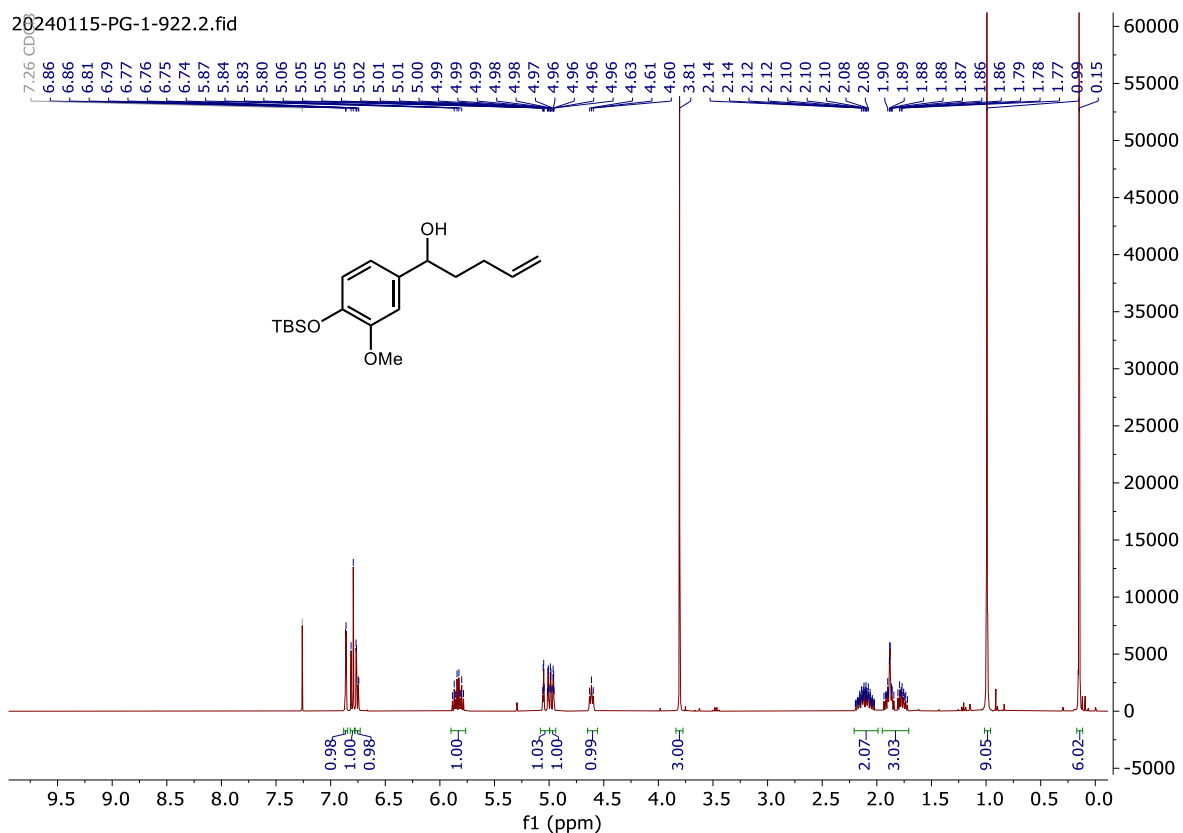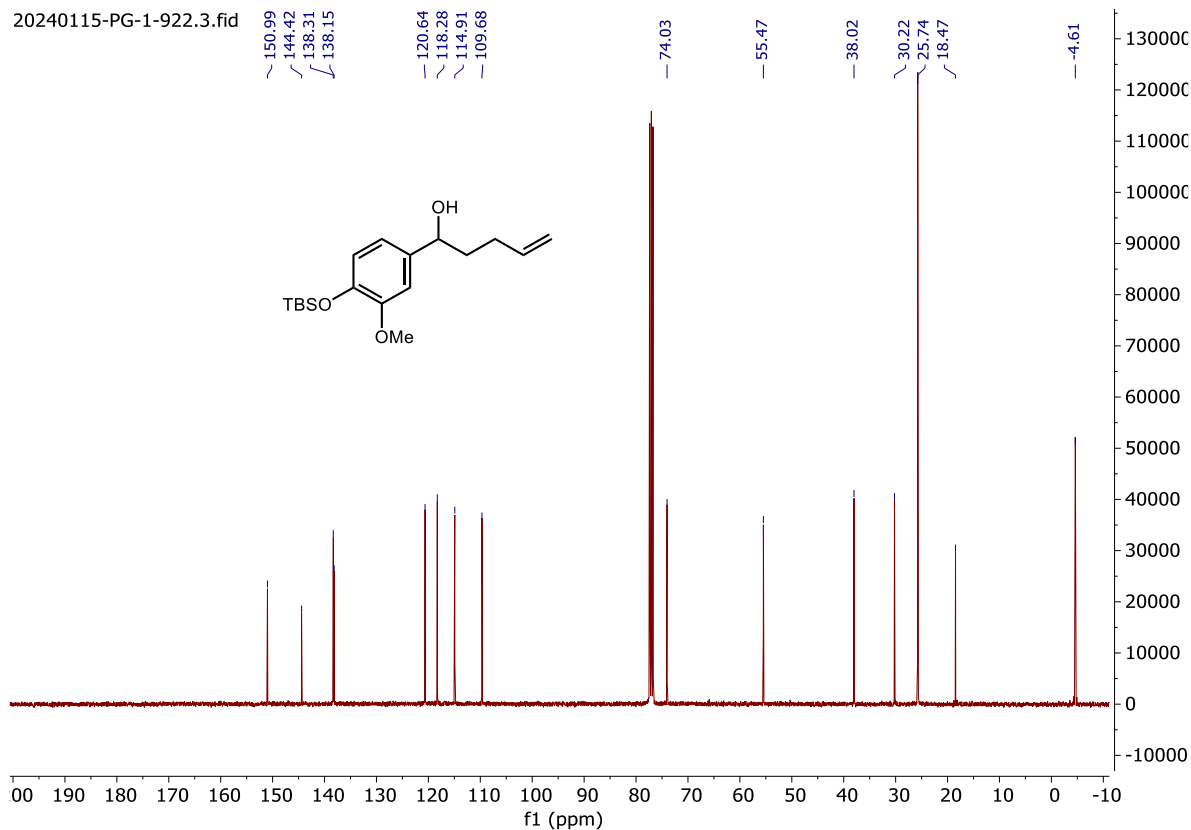

1-(4-((*tert*-butyldimethylsilyl)oxy)-2-methoxyphenyl)pent-4-en-1-ol (S25):

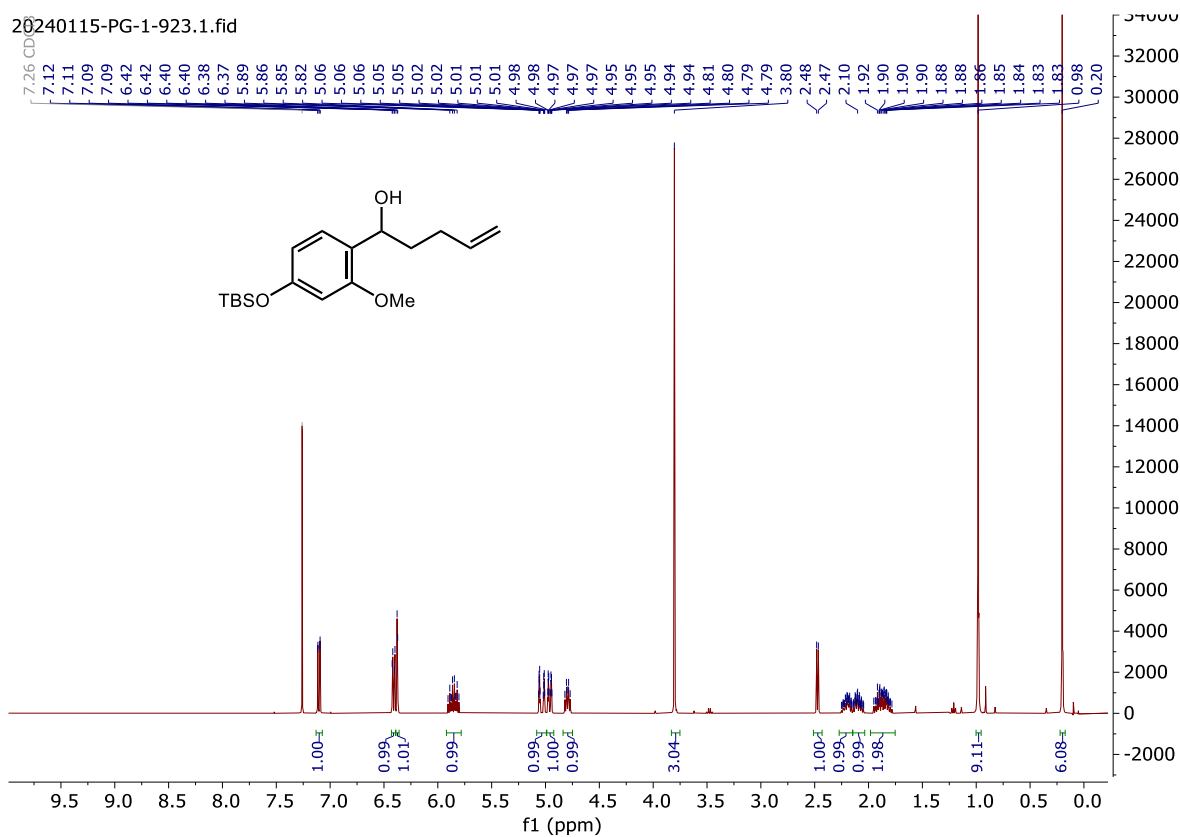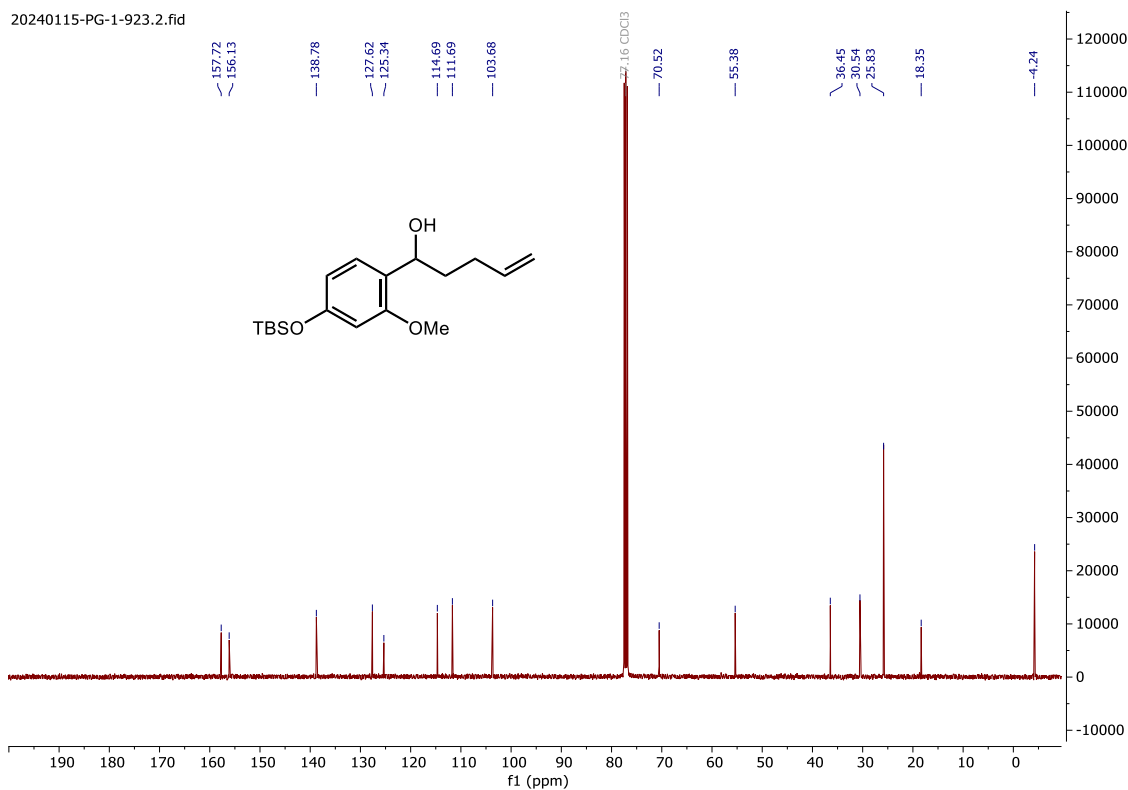

20231026-PG-1-964.1.fid

Chemical structure: CC(C)(C)C(C)C1=CC=C(C=C1)C2=CC=C(C=C2)C3=CC=CC=C3C4=CC=CC=C4C5=CC=CC=C5C6=CC=CC=C6C7=CC=CC=C7C8=CC=CC=C8C9=CC=CC=C9C10=CC=CC=C10C11=CC=CC=C11C12=CC=CC=C12C13=CC=CC=C13C14=CC=CC=C14C15=CC=CC=C15C16=CC=CC=C16C17=CC=CC=C17C18=CC=CC=C18C19=CC=CC=C19C20=CC=CC=C20C21=CC=CC=C21C22=CC=CC=C22C23=CC=CC=C23C24=CC=CC=C24C25=CC=CC=C25C26=CC=CC=C26C27=CC=CC=C27C28=CC=CC=C28C29=CC=CC=C29C30=CC=CC=C30C31=CC=CC=C31C32=CC=CC=C32C33=CC=CC=C33C34=CC=CC=C34C35=CC=CC=C35C36=CC=CC=C36C37=CC=CC=C37C38=CC=CC=C38C39=CC=CC=C39C40=CC=CC=C40C41=CC=CC=C41C42=CC=CC=C42C43=CC=CC=C43C44=CC=CC=C44C45=CC=CC=C45C46=CC=CC=C46C47=CC=CC=C47C48=CC=CC=C48C49=CC=CC=C49C50=CC=CC=C50C51=CC=CC=C51C52=CC=CC=C52C53=CC=CC=C53C54=CC=CC=C54C55=CC=CC=C55C56=CC=CC=C56C57=CC=CC=C57C58=CC=CC=C58C59=CC=CC=C59C60=CC=CC=C60C61=CC=CC=C61C62=CC=CC=C62C63=CC=CC=C63C64=CC=CC=C64C65=CC=CC=C65C66=CC=CC=C66C67=CC=CC=C67C68=CC=CC=C68C69=CC=CC=C69C70=CC=CC=C70C71=CC=CC=C71C72=CC=CC=C72C73=CC=CC=C73C74=CC=CC=C74C75=CC=CC=C75C76=CC=CC=C76C77=CC=CC=C77C78=CC=CC=C78C79=CC=CC=C79C80=CC=CC=C80C81=CC=CC=C81C82=CC=CC=C82C83=CC=CC=C83C84=CC=CC=C84C85=CC=CC=C85C86=CC=CC=C86C87=CC=CC=C87C88=CC=CC=C88C89=CC=CC=C89C90=CC=CC=C90C91=CC=CC=C91C92=CC=CC=C92C93=CC=CC=C93C94=CC=CC=C94C95=CC=CC=C95C96=CC=CC=C96C97=CC=CC=C97C98=CC=CC=C98C99=CC=CC=C99C100=CC=CC=C100

1H NMR spectrum (f1 (ppm)) showing peaks and integration values:

Chemical Shift (ppm)	Integration
8.28	0.94
8.25	1.09
7.79	1.05
7.78	1.05
7.77	1.04
7.76	1.04
7.68	1.04
7.66	1.04
7.49	1.04
7.47	1.04
7.47	1.04
7.47	1.04
7.45	1.04
7.45	1.04
7.38	1.04
7.37	1.04
7.36	1.04
7.35	1.04
7.34	1.04
7.34	1.04
7.26	1.04
7.10	1.04
7.08	1.04
5.93	1.04
5.91	1.04
5.89	1.04
5.86	1.04
5.62	1.04
5.61	1.04
5.10	1.04
5.09	1.04
5.06	1.04
5.05	1.04
5.01	1.04
5.00	1.04
4.98	1.04
4.98	1.04
2.28	1.04
2.27	1.04
2.26	1.04
2.24	1.04
2.22	1.04
2.22	1.04
2.22	1.04
2.20	1.04
1.97	1.04
1.96	1.04
1.08	1.04
0.38	1.04
0.33	1.04

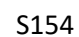

2,2,2-trichloroethyl 2-phenethylaziridine-1-carboxylate (12):

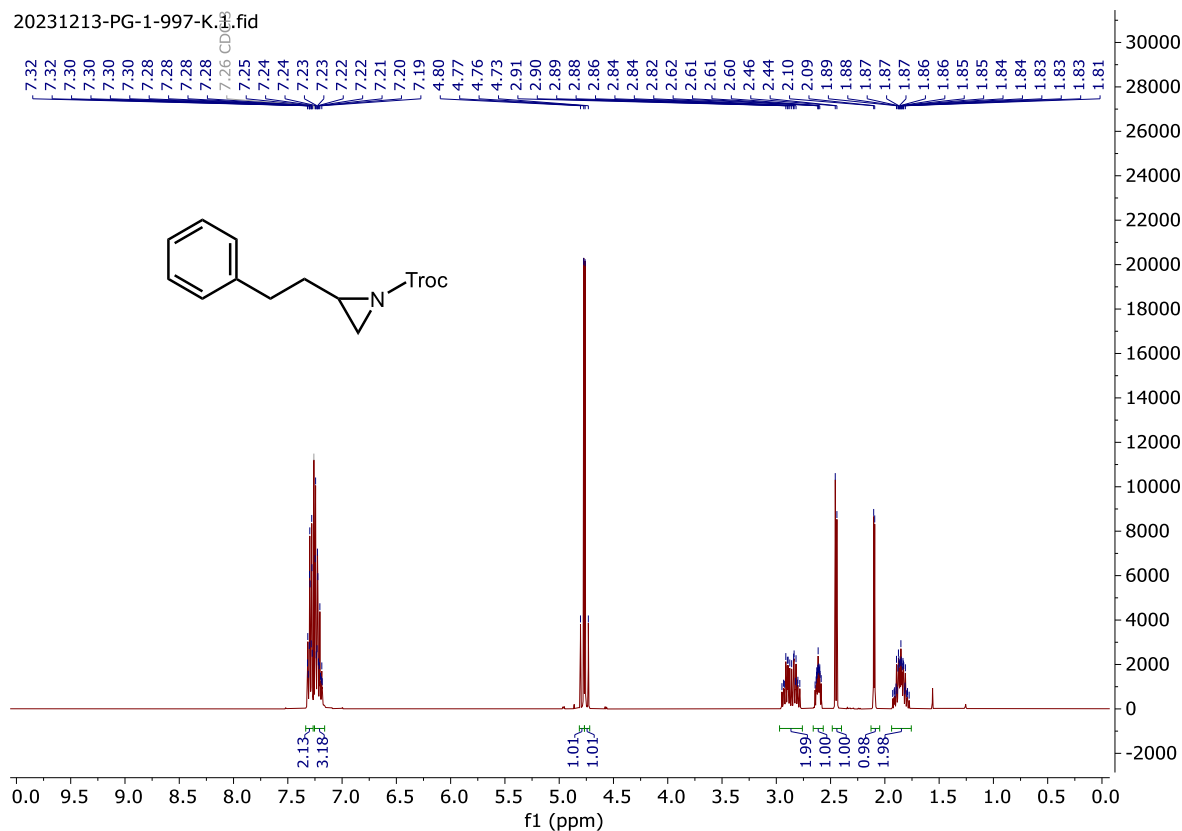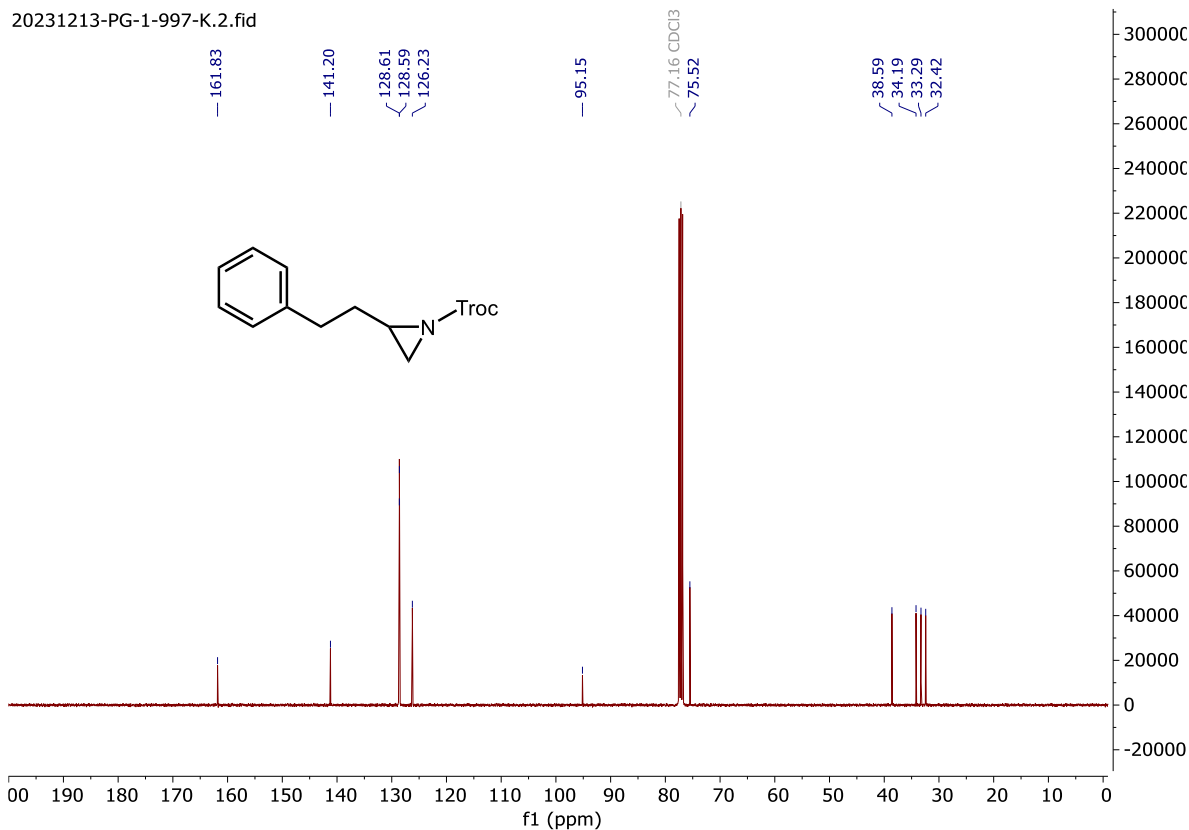

(but-3-en-1-yl-2,2-d₂)benzene (**6-d₂**):

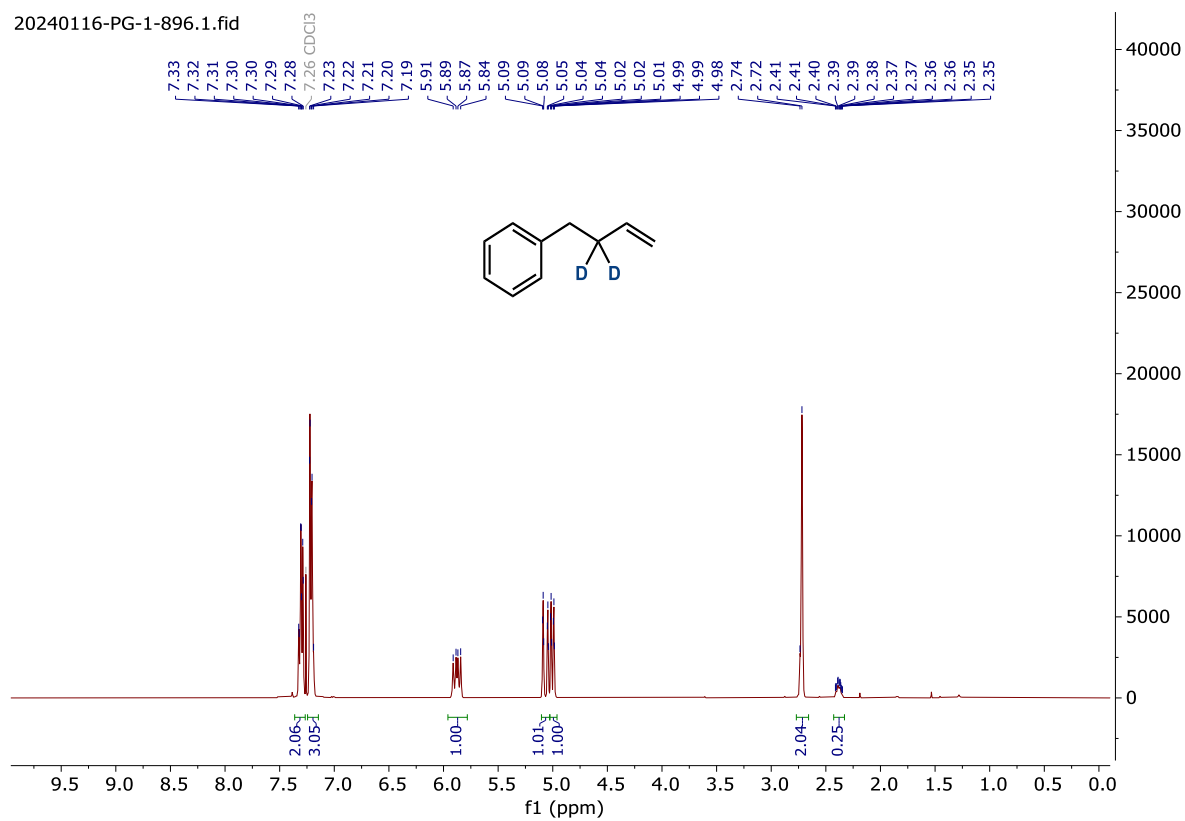

(E)-(but-3-en-1-yl-4-d)benzene (E-6-d₁):

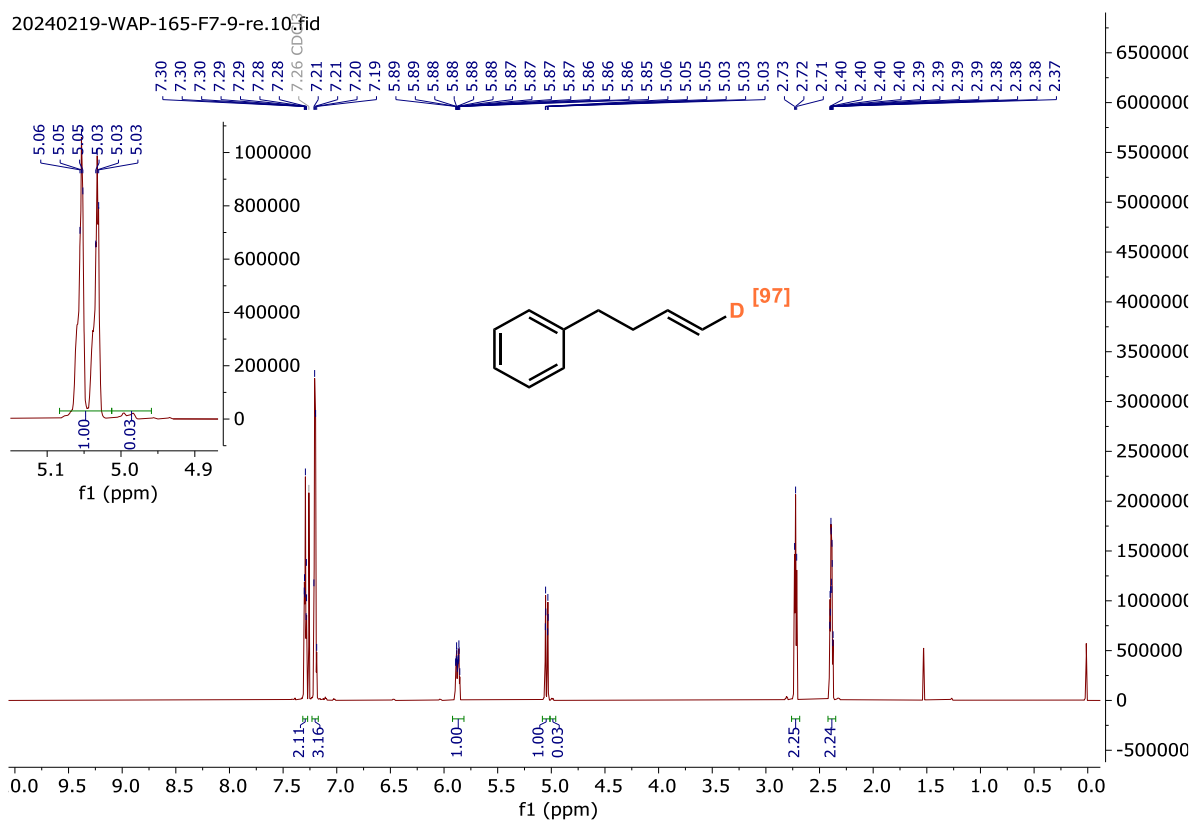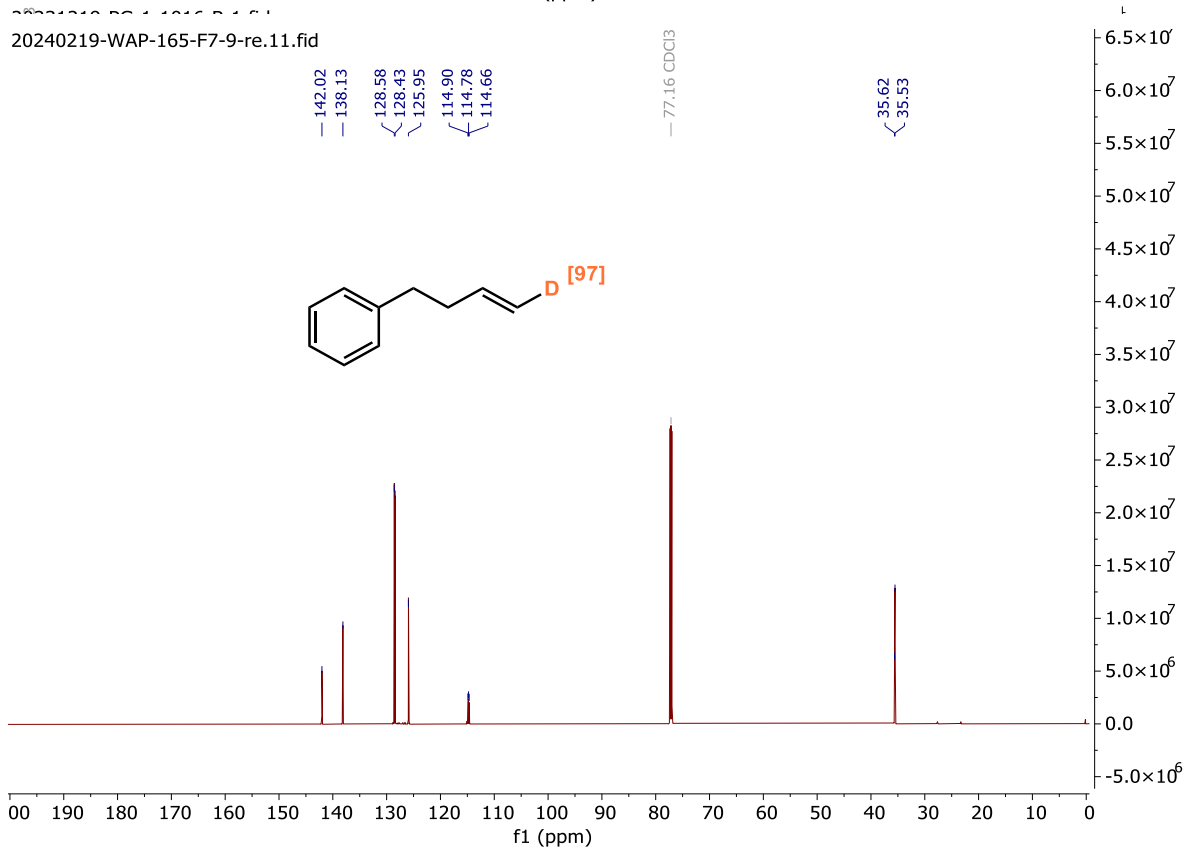

2,2,2-trichloroethyl (1,2,3,4-tetrahydronaphthalen-2-yl-3,3-d₂)carbamate (10-d₂**):**

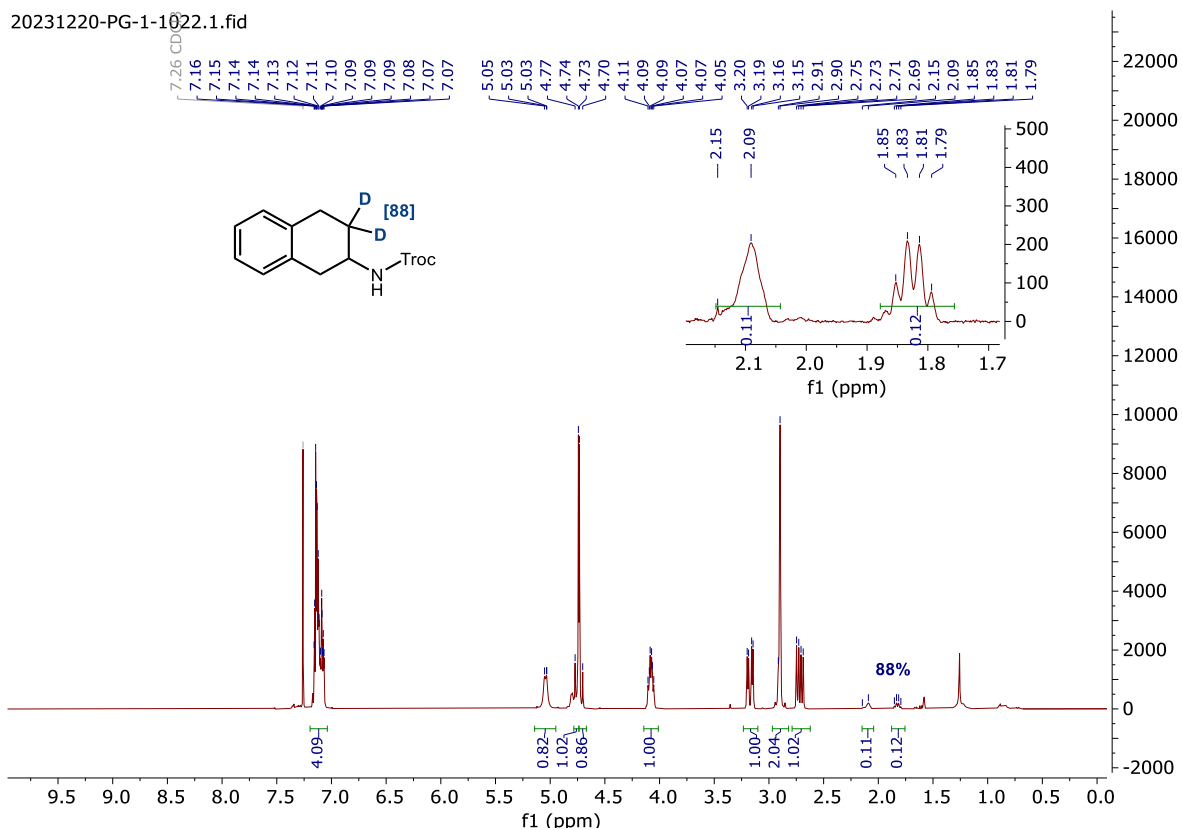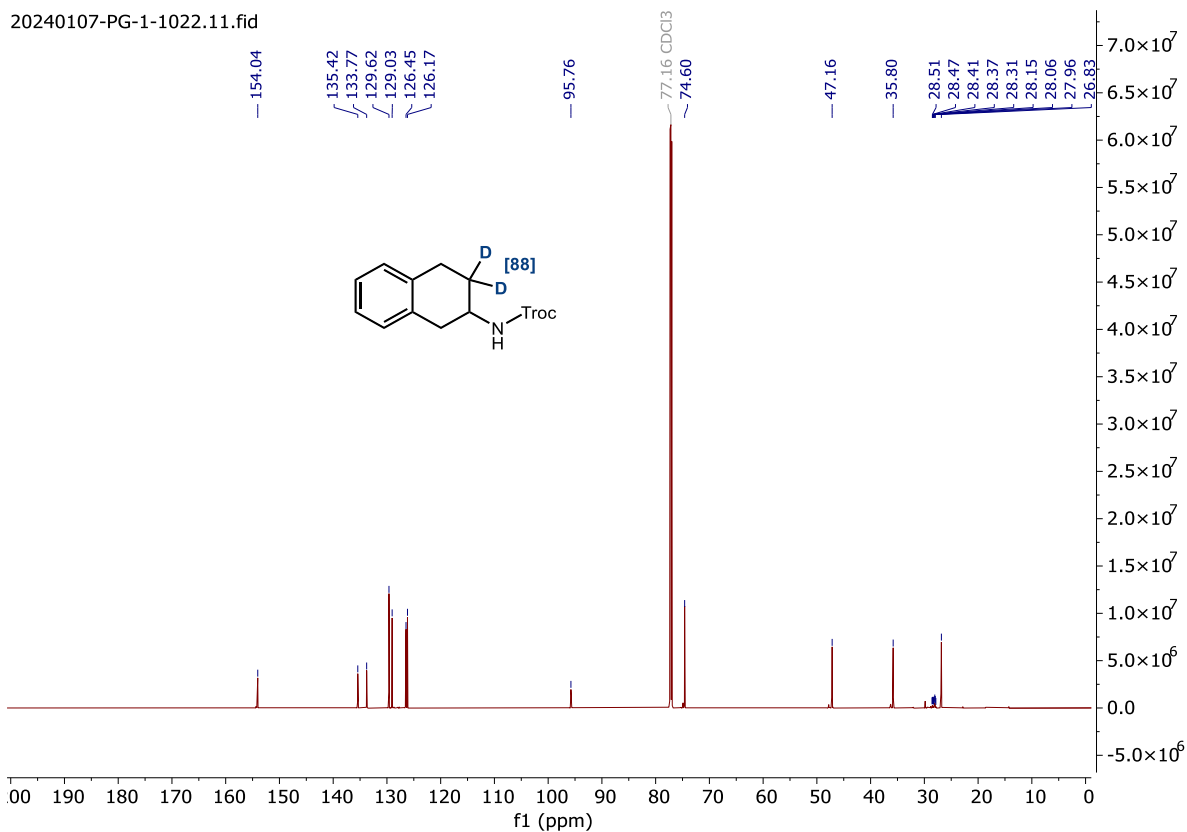

22,2,2-trichloroethyl (1,2,3,4-tetrahydronaphthalen-2-yl-1-d)carbamate (**10-d₁**):

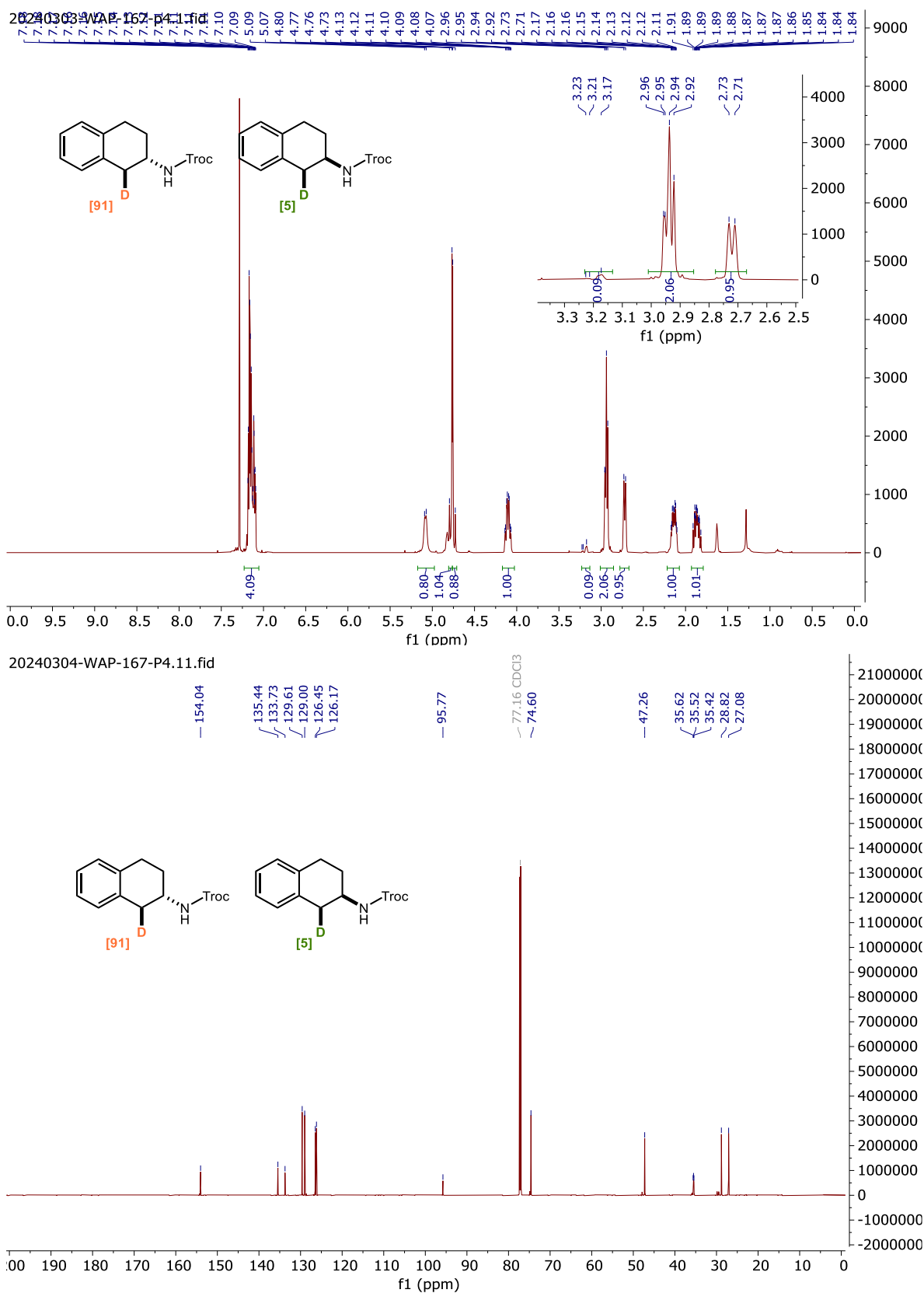

1-(phenyl-*d*₅)but-3-en-1-ol (S33-*d*₅):

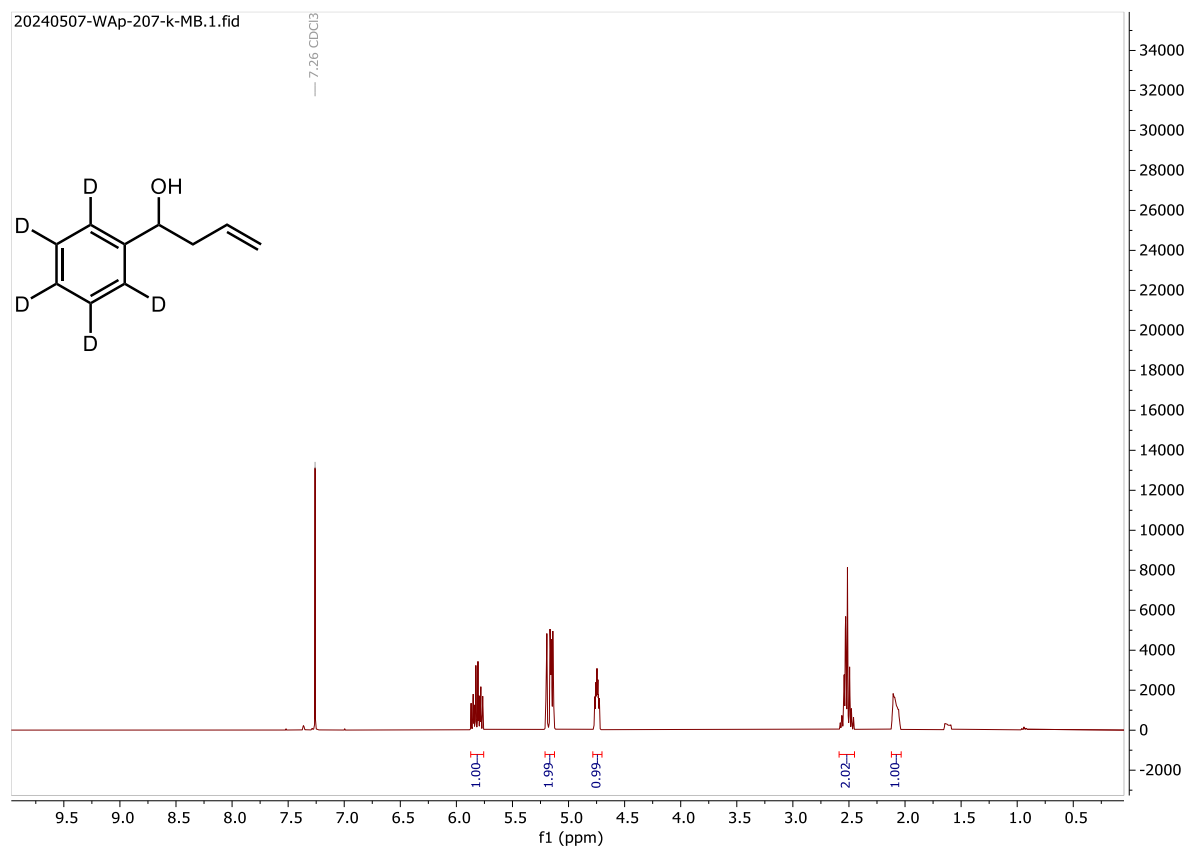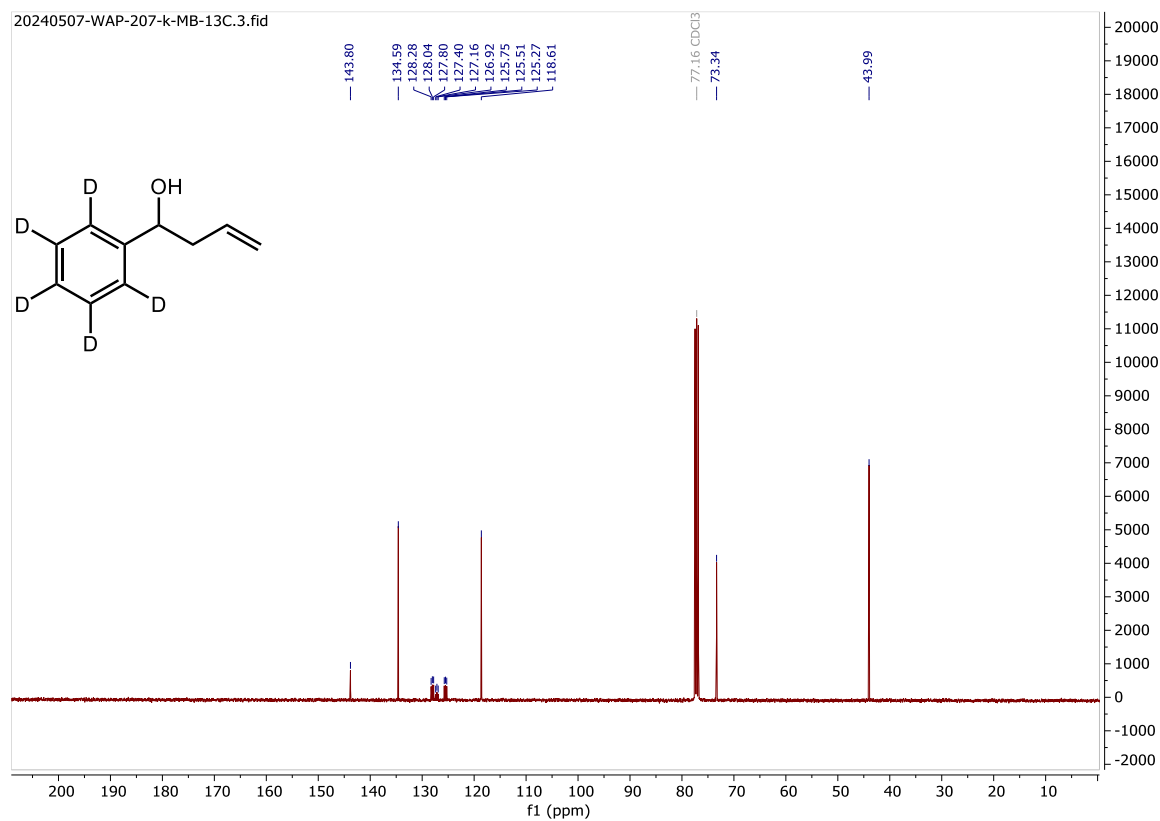

1-(but-3-en-1-yl)benzene-2,3,4,5,6-d₅ (**6-d₅**):

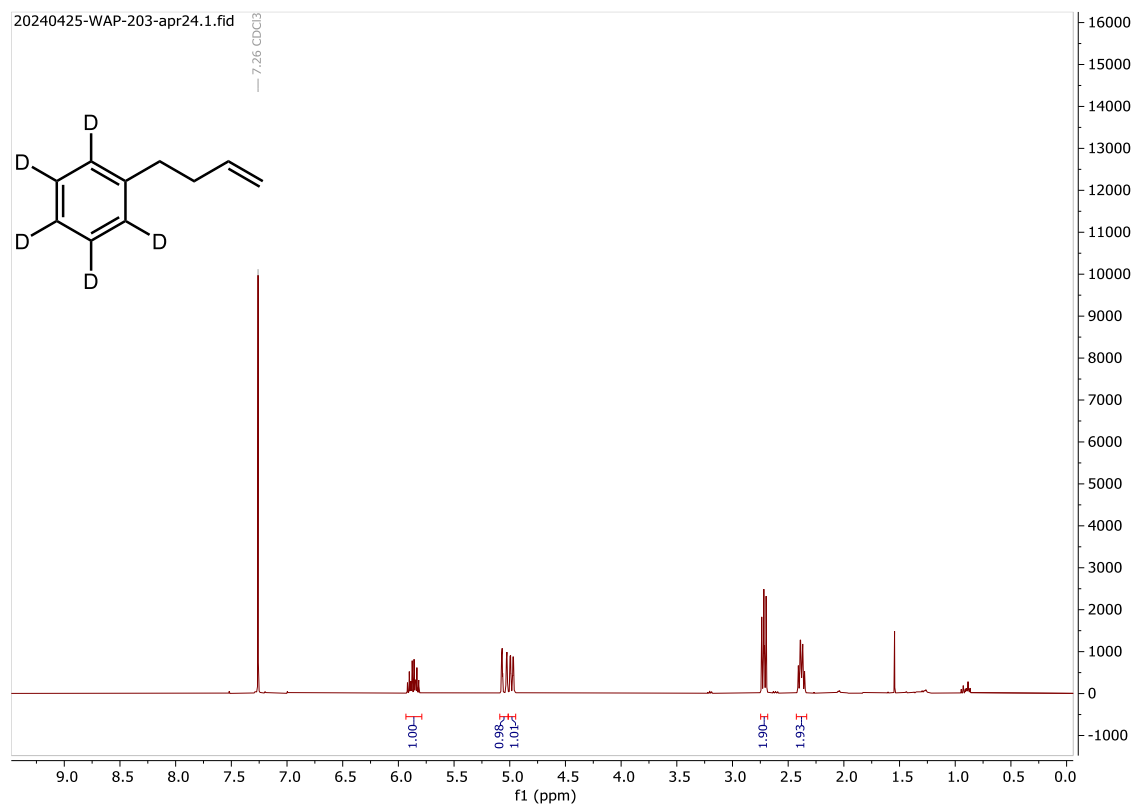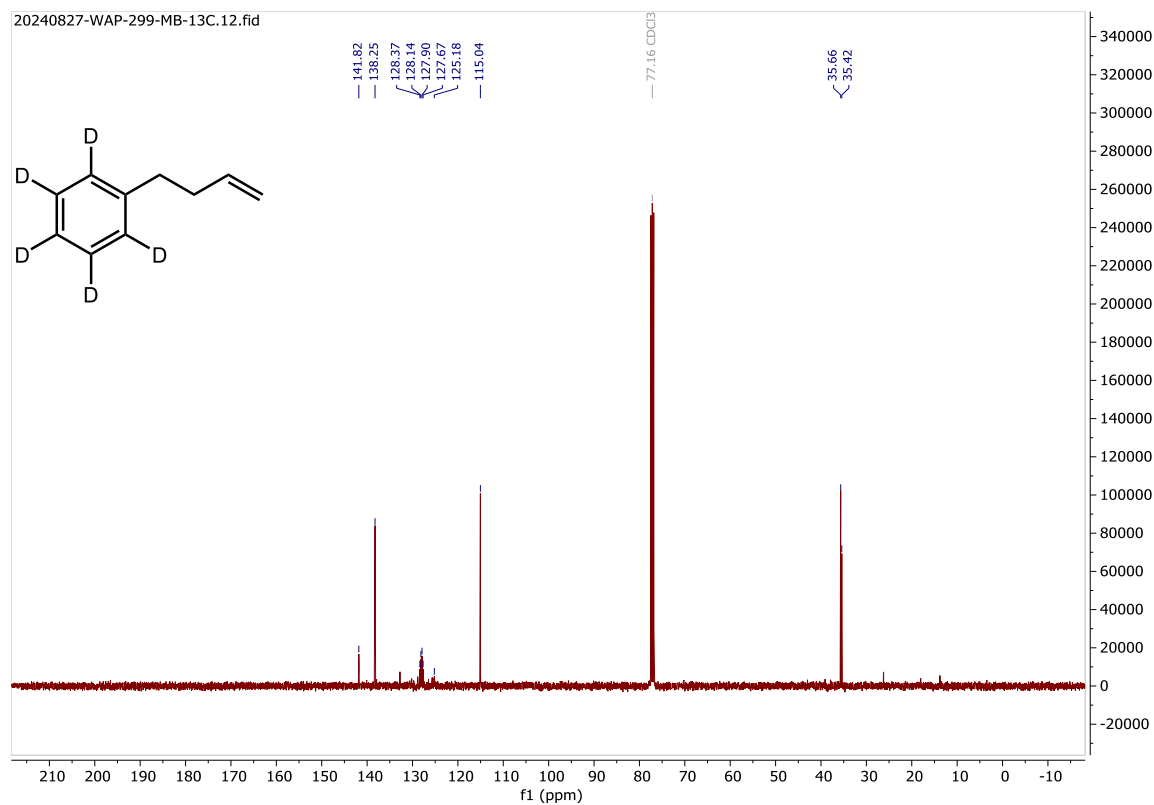

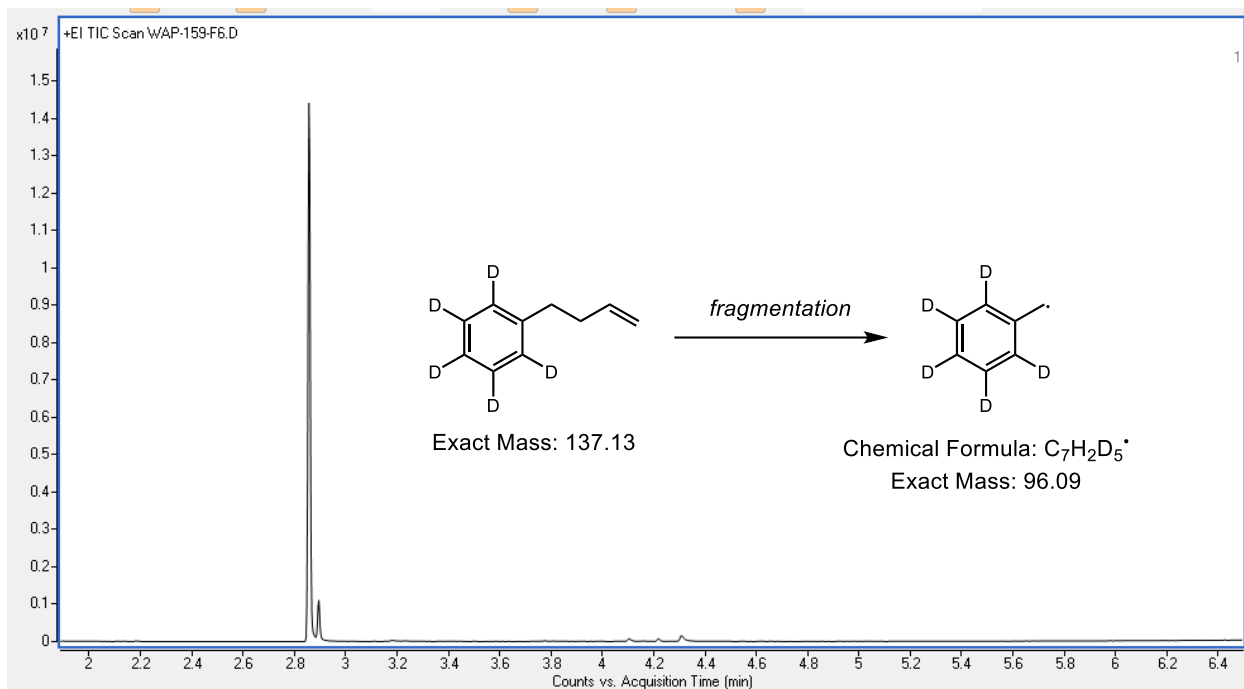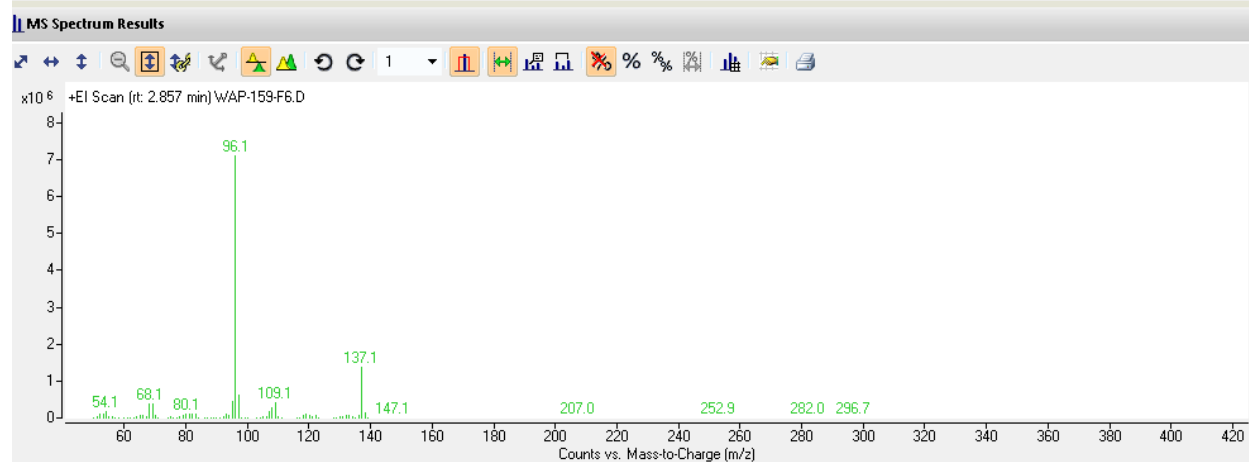

2,2,2-trichloroethyl (1,2,3,4-tetrahydronaphthalen-2-yl-5,6,7,8-d₄)carbamate (10-d₄)

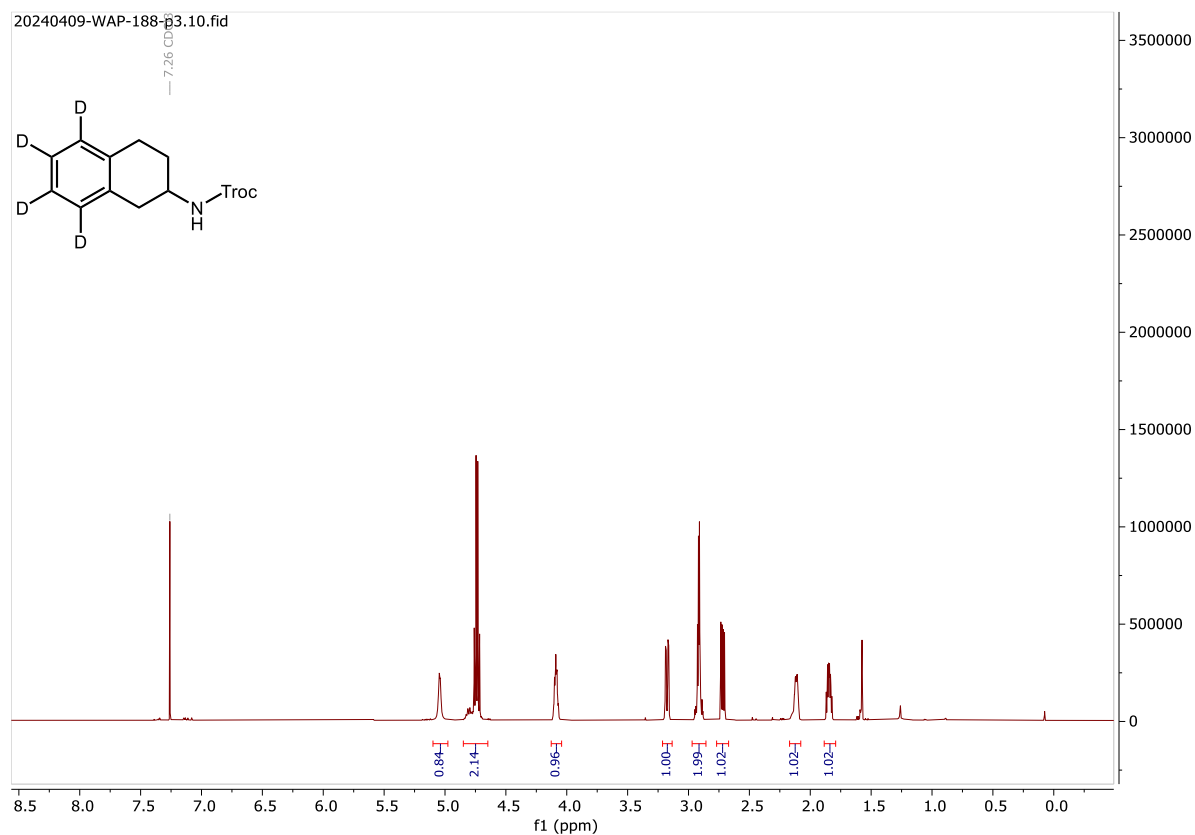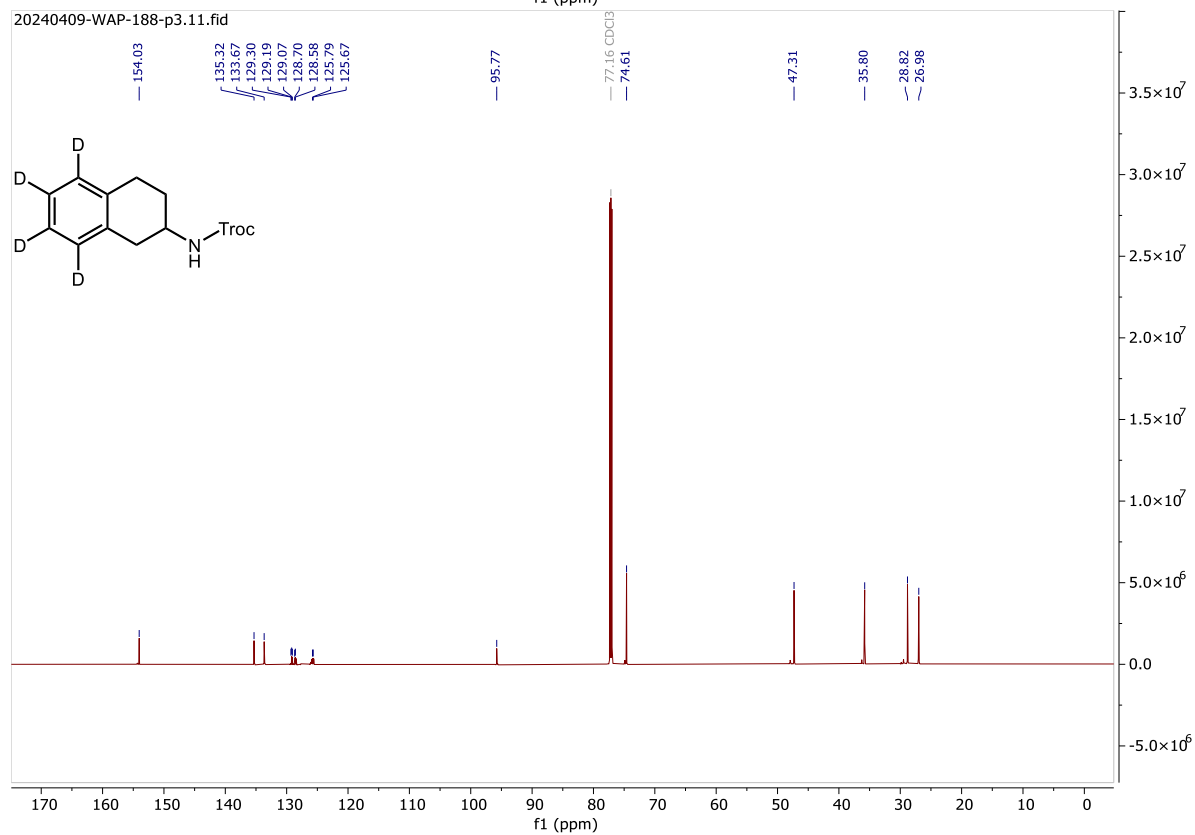

Supplement: Supplementary file 1 [file cs5c07589_si_001.pdf]
